# Dwarfism of high‐monolignol Arabidopsis plants is rescued by ectopic LACCASE overexpression

**DOI:** 10.1002/pld3.265

**Published:** 2020-09-28

**Authors:** Mendel L. Perkins, Mathias Schuetz, Faride Unda, Rebecca A. Smith, Richard Sibout, Natalie J. Hoffmann, Darren C. J. Wong, Simone D. Castellarin, Shawn D. Mansfield, Lacey Samuels

**Affiliations:** ^1^ Department of Botany University of British Columbia Vancouver Canada; ^2^ Department of Wood Science University of British Columbia Vancouver Canada; ^3^ Department of Energy's Great Lakes Bioenergy Research Center Department of Biochemistry University of Wisconsin‐Madison Madison WI USA; ^4^ UR1268 BIA (Biopolymères Interactions Assemblages) INRA Nantes France; ^5^ Wine Research Centre University of British Columbia Vancouver Canada

## Abstract

Lignin is a key secondary cell wall chemical constituent, and is both a barrier to biomass utilization and a potential source of bioproducts. The Arabidopsis transcription factors *MYB58* and *MYB63* have been shown to upregulate gene expression of the general phenylpropanoid and monolignol biosynthetic pathways. The overexpression of these genes also results in dwarfism. The vascular integrity, soluble phenolic profiles, cell wall lignin, and transcriptomes associated with these MYB‐overexpressing lines were characterized. Plants with high expression of *MYB58* and *MYB63* had increased ectopic lignin and the xylem vessels were regular and open, suggesting that the stunted growth is not associated with loss of vascular conductivity. *MYB58* and *MYB63* overexpression lines had characteristic soluble phenolic profiles with large amounts of monolignol glucosides and sinapoyl esters, but decreased flavonoids. Because loss of function *lac4 lac17* mutants also accumulate monolignol glucosides, we hypothesized that LACCASE overexpression might decrease monolignol glucoside levels in the MYB‐overexpressing plant lines. When laccases related to lignification (*LAC4* or *LAC17*) were co‐overexpressed with *MYB63* or *MYB58*, the dwarf phenotype was rescued. Moreover, the overexpression of either *LAC4* or *LAC17* led to wild‐type monolignol glucoside levels, as well as wild‐type lignin levels in the rescued plants. Transcriptomes of the rescued double *MYB63‐OX/LAC17‐OX* overexpression lines showed elevated, but attenuated, expression of the *MYB63* gene itself and the direct transcriptional targets of MYB63. Contrasting the dwarfism from overabundant monolignol production with dwarfism from lignin mutants provides insight into some of the proposed mechanisms of lignin modification‐induced dwarfism.

## 1 INTRODUCTION

As society increasingly seeks to transition toward renewable resources, plant biomass is increasingly viewed as a potential source of commodity chemicals and novel bioproducts (Ralph, Lapierre, & Boerjan, 2019). Lignin is a key chemical component of plant biomass, conferring strength to cell walls, and resistance to degradation. For uses of plant biomass in forage and potential bioenergy applications, efforts have largely focused on reducing or altering lignin content, as it generally impedes accessibility to the carbohydrate cell wall fractions (DeMeester, de Vries, & Özparpucu, 2018). More recently, lignin is viewed positively as a valuable potential source of chemical feedstocks, and engineering lignin modification has been a major target (Mahon & Mansfield, 2019; Mottiar, Vanholme, Boerjan, Ralph, & Mansfield, 2016; Umezawa, 2018). In studies where lignin has been modified with the aim of biotechnological applications, and in mutant studies where monolignol biosynthesis has been perturbed, unexpected stunted growth phenotypes, termed ligninmodification‐induced dwarfism (LMID), have been observed (reviewed by (Muro‐Villanueva, Mao, & Chapple, 2019). Understanding why modifications in lignin content affect plant growth represents an important challenge.

Lignin is a polymer primarily made up of hydroxycinnamyl alcohols. Lignin also has a remarkable capacity to incorporate a wide range of other components including ferulic acid, tricin, and coniferyaldehyde (Mottiar et al., 2016). Hydroxycinnamyl alcohols are derived from the core phenylpropanoid pathway, commencing with the amino acid phenylalanine and resulting in the production of lignin monomers, or monolignols (Ralph et al., 2019). *p*‐coumaroyl‐CoA represents a branch point between lignin biosynthesis and the biosynthesis of other phenolic compounds such as flavonoids (Dixon & Barros, 2019; Liu, Luo, & Zheng, 2018; Vanholme, De Meester, Ralph, & Boerjan, 2019). From *p*‐coumaroyl‐CoA, a series of subsequent hydroxylations, methylations, and reductions results in the formation of the three canonical monolignols (*p*‐coumaryl‐, coniferyl‐, and sinapyl alcohols). The enzymes catalyzing this series of reactions have been biochemically characterized, and their functions inferred through mutant analysis in *Arabidopsis thaliana* (Arabidopsis; Van‐Acker et al., 2013; Vanholme, Storme, & Vanholme, 2012) and gene knockdowns in *Medicago sativa* (alfalfa; Chen & Dixon, 2007) and poplar (Coleman, Park, Nair, Chapple, & Mansfield, 2008a. In addition to elucidating the biosynthetic pathways, the regulation of lignin production has also been studied in Arabidopsis. Both the general phenylpropanoid pathway and the monolignol‐specific pathway are positively regulated by two MYB transcription factors, MYB58 and MYB63 (Zhou, Lee, Zhong, & Ye, 2009). These transcription factors are themselves controlled within the context of vessel and fiber development by upstream master transcription factors (Ohtani & Demura, 2019).

When lignin deposition is triggered, monolignols are synthesized in the cytoplasm of both the lignifying, and in some cases, neighboring cells (Blokhina, Laitinen, & Hatakeyama, 2019; Smith et al., 2013). It remains uncertain exactly how monomers move from their site of synthesis to the wall where they are polymerized. Several possible mechanisms have been proposed, but there is no genetic evidence for transporter‐mediated export of the major monolignols, coniferyl alcohol and sinapyl alcohol (Perkins, Smith, & Samuels, 2019). This may reflect a non‐transporter‐mediated mechanism, such as diffusion (Vermaas et al., 2019). Alternatively, active transport of monolignols by ABC transporters (Alejandro, Lee, & Tohge, 2012; Miao & Liu, 2010) or transport of monolignol glucosides have also been proposed (Tsuyama et al., 2013).

Once in the wall, the monomers are oxidized by secreted laccase and/or peroxidase to form monolignol radicals that ultimately polymerize into lignin. Simultaneous mutation of the two most highly expressed laccases in Arabidopsis inflorescence stems, LAC4 and LAC17, resulted in irregular xylem and reduced Klason lignin phenotypes (Berthet, Demont‐Caulet, & Pollet, 2011). The mutation of a third laccase, *lac11*, in addition to *lac4* and *lac17* resulted in severe dwarfism and nearly absent lignin, suggesting these laccases play a major role in lignification (Zhao, Nakashima, & Chen, 2013). In the absence of polymerization machinery in the laccase triple mutant, excess monomers led to the formation of monolignol glucosides (Zhao et al., 2013), likely as a detoxification product (Väisänen et al., 2015). These monolignol glucosides are thought to be sequestered in plant vacuoles. Both nonspecific monolignol export and activity of oxidative enzymes in the cell wall confer flexibility in a plants’ ability to incorporate non‐canonical monomers into lignin (Mottiar et al., 2016). These factors suggest that manipulation of lignin content should be possible, but the deleterious effects on plant growth due to LMID remain an issue.

The mechanisms underlying LMID are currently the topic of intense interest (DeMeester et al., 2018; Panda, Li, Wager, Chen, & Li, 2020). Some mechanisms underlying LMID have been proposed, and then challenged. For example, *HCT^RNAi^* (*HYDROXYCINNAMOYL COENZYMEA:SHIKIMATE HYDROXYCINNAMOYL TRANSFERASE*) mutants (Hoffmann et al., 2005) were proposed to be dwarf due to flavonoid‐induced inhibition of growth and auxin transport (Besseau et al., 2007). This model was not supported by the observation that loss of flavonoids in chalcone synthase mutants did not result in a dwarf phenotype (Li, Bonawitz, Weng, & Chapple, 2010). In *CINNAMOYL‐COENZYME A REDUCTASE1 (ccr1‐4)* mutants, dwarf growth was reported to be due to ferulic acid accumulation creating disruption of the cell cycle during leaf development (Xue et al., 2015). This model is not supported by the observation that wild‐type growth is possible in *ccr1* mutants when a wild‐type copy of the CCR1 gene is expressed exclusively in xylem cells (DeMeester et al., 2018). Other possible mechanisms leading to LMID include loss of vascular integrity, accumulation of pathway intermediates or derivatives, or triggering cell wall integrity sensing (Bonawitz & Chapple, 2013; Gallego‐Giraldo, Liu, & Pose‐Albacete, 2020; Muro‐Villanueva et al., 2019). Forward genetic screens have identified that LMID requires subunits of the transcriptional coregulator Mediator (Bonawitz et al., 2014), as well as an importin‐beta protein required to bring the MYB4 transcriptional repressor into the nucleus (Panda et al., 2020). These results highlight components of pathways that regulate the interaction between lignin defects and associated phenotypes, but leave large gaps in our understanding of the interaction between the pathways and the majority of the other factors involved.

Most studies examining LMID concern mutants with perturbations in the monolignol biosynthesis pathway, leading to phenotypes such as irregular xylem (DeMeester et al., 2018) or lower lignin levels (Van‐Acker et al., 2013). Paradoxically, overexpression of the regulatory *MYB58* and *MYB63* transcription factors that led to increased gene expression in the phenylpropanoid and monolignol biosynthetic pathways also led to impaired plant growth (Zhou et al., 2009). These plants with overabundant monolignol production and impaired growth provide opportunities to examine some of the proposed mechanisms of LMID. The first objective of this study was to examine the phenotypes of *Pro35S::MYB58* (*MYB58‐OX*) and *Pro35S::MYB63* (*MYB63‐OX*) plants that are relevant to LMID, such as vascular integrity, soluble phenolic profiles, and transcriptomes. One striking result was the high monolignol glucoside levels in these plants. As high monolignol glucosides are also found in loss of function *lac4 lac11 lac17* triple mutants (Zhao et al., 2013), we hypothesized that additional copies of the lignin‐related genes *LAC4* or *LAC17* might rescue the LMID in *MYB58‐OX* or *MYB63‐OX* lines. Co‐expression of *LAC4* or *LAC17* was able to rescue the LMID growth phenotypes of *MYB58‐OX* or *MYB63‐OX*, in addition to reducing monolignol glucoside levels and transcriptome changes away from “stress‐related” genes. These data have interesting implications for understanding potential causes of LMID (Bonawitz & Chapple, 2013), arguing against dwarfism from the loss of a monolignol‐derived growth‐promoting molecules or the loss of lignin in vascular bundles, because *MYB58‐OX/MYB63‐OX* lines have high levels of monolignols and open xylem vessels. Comparing these plants with monolignol biosynthetic mutants (Bonawitz & Chapple, 2013; Vanholme et al., 2012) and engineered plants (DeMeester et al., 2018; Yang, Mitra, & Zhang, 2013) provides further insights into how modification of lignin can produce modified growth phenotypes.

## 2 MATERIALS AND METHODS

### 2.1 Plant growth

Arabidopsis seeds were sown on ½ MS agar plates, were vernalized at 4°C for 2–3 days, and were transferred to soil after 7 days of growth together with WT seedlings. Growth conditions for all plants were set to 21°C, 16 hr light/8 hr dark, and 210 μmol m^−2^ s^−1^ light intensity.

### 2.2 Microscopy

A Leica DMR epifluorescence microscope using 350/50 excitation and 455  nm longpass emission filter sets was used to document ultraviolet autofluorescence of Arabidopsis leaves and stems. A Perkin‐Elmer UltraView VoX spinning disk confocal mounted on a Leica DMI6000 inverted microscope and a Hamamatsu 9100‐02 CCD camera were used to image fluorescent proteins in living plant cells using the following excitation and emission filters: GFP (488 and 525), YFP (514 and 540), and RFP/m‐Cherry (561 and 595). To study cell wall localization of LAC4, surface sterilized seeds were plated on GM media (MS media supplemented with 1% Sucrose and 1x Gamborg's Vitamin mix; Phytotechnology labs), vernalized at 4ºC for 2–3 days before being transferred to growth chambers. Seeds were induced to germinate in 8 hr of light, then wrapped in foil and kept in darkness for 7 days. 7‐day‐old etiolated cotyledons were plasmolyzed in 0.4M D‐mannitol (Sigma‐Aldrich) for 1 hr. Three seedlings were imaged as above for three independent lines of each construct for two independent experiments. Hand sections of Arabidopsis stem were Mäule stained (Mitra and Loqué, 2014). To assess cell wall localisation of Lac4‐mCherry, 7 day old etiolated hypocotyls were plasmolyzed in 0.4M mannitol for 1 hour. A line of *prUBQ10‐sec‐mCherry* (Chou et al 2018) was used as a control for cell wall localized signal.

### 2.3 Molecular biology

Genomic sequences containing the MYB58 or MYB63 coding sequences were amplified using Phusion® High‐Fidelity DNA Polymerase (New England Biolabs) using the primers listed in Table S4a. These PCR fragments were cloned using gateway cloning methodology (Invitrogen) using the pDONR221 vector as an entry clone and subsequently shuttled into the pK2GW7 binary vector (Karimi, Inzé, & Depicker, 2002).

### 2.4 Real‐time quantitative PCR

Gene‐specific primers amplifying 190‐240bp amplicons of were designed using primer 3 software (Koressaar & Remm, 2007) and are listed in Table S4b. The *APT1* (AT1G27450) gene was used as a reference gene as described in Guénin et al. (2009). Real‐time PCR was performed using iQ SYBR Green supermix (Biorad) and CFX connect real‐time PCR detection system (Biorad). Efficiencies of PCR amplification and quantifications were performed according to the manufacturer's specification, as described in Schmittgen and Livak (2008). Total RNA was isolated from 5‐week‐old plants using TRIzol reagent (Invitrogen) and cDNA was synthesized using SuperScript III reverse transcriptase (Invitrogen) according to the manufacturer's instructions.

### 2.5 Soluble phenolics extraction and analysis

Three‐ to four‐week‐old plants were harvested into liquid nitrogen and ground using a mortar and pestle. Fifty to 100 milligrams of ground tissue was combined with 1 ml of “methanol water” solution (49.5% methanol:1% acetic acid in water) and incubated 45°C for 4 hr to extract soluble phenolic compounds. The samples were centrifuged at 15 000 rpm for 15 min and the supernatant was transferred to glass vials. Phase partitioning with 1 ml ethyl ether was performed three times, with the upper layer transferred to new glass vials. The lower phase water‐soluble phase was also transferred to separate glass vials. Samples were allowed to dry out overnight before resuspension in 50% methanol, sonication for 15–20 min and incubation at 35°C for 1 hr. Samples were filter‐sterilized into HPLC vials and soluble phenolic compounds were separated by running approximately 10 μL of sample on an LC30 Chromatography Oven HPLC fitted with a Symmetry C14 column and PDA‐100 Photodiode Array Detector (Dionex). The samples were examined at the wavelengths 280, 320, and 510 nm. Methanol extracts were eluted from the column over a gradient from 95% A (100% water:0.1% trifluoroacetic acid (TFA)) to 45% B (75% acetonitrile:25% methanol: 0.1% TFA) over 50 min, followed by a 10 min wash with 75% B and re‐acclimation of the column with 95% A for 10 min. The flow rate was 1 ml/minute; the column temperature was set to 40°C. Coniferin and syringin HPLC standards were prepared at a concentration of 0.05 mg/ml methanol and run on the HPLC using the conditions listed above.

To analyze the soluble phenolic phase by liquid chromatography/mass spectroscopy, 10 μL of the same samples was run through an Agilent Zorbax Eclipse XDB C18 column (4.6 × 70 mm, particle size 1.8 μm) with a flow rate of 0.7 ml/min at 30°C. The samples were eluted with an increasing concentration of acetonitrile in 5% formic acid from 10% to 25% over 16 min, and from 25% to 100% over 9 min. The detection and analysis of metabolites was performed using a Bruker maXis Impact Ultra‐High Resolution tandem TOF (UHR‐Qq‐TOF) mass spectrometer in positive electrospray ionization mode, temperature 220°C, drying gas flow rate 10 L/min, nebulizer pressure 4 bars, capillary voltage 3800 V, and using sodium formate as a calibrant.

### 2.6 Structural chemistry analysis

Arabidopsis stems from 8‐ to 10‐week‐old plants were used to determine lignin and carbohydrate content following a modified Klason method (Cullis, Saddler, & Mansfield, 2004). Samples were ground in a Wiley mill to pass a 40 mesh screen, treated with acetone overnight using a Soxhlet, and then dried for 48 hr at 50°C. Approximately 150 mg of dried extractive‐free tissue was treated with 72% sulfuric acid for 2 hr, diluted to ~3% with 112 ml DI water and autoclaved at 121°C for 60 min. The mixture was filtered through a medium coarseness crucible and the retentate dried at 105°C. The acid‐insoluble lignin was determined by weighing the retentate, while the acid‐soluble lignin was measured from an aliquot of the filtrate using an UV spectrophotometer at 205 nm. Carbohydrate contents were determined by HPLC analysis of the filtrate. Glucose, xylose, mannose, galactose, arabinose, and rhamnose were analyzed using a Dx‐600 anion‐exchange HPLC (Dionex) fitted with a CarboPac PA1 column (Dionex) at 1 ml/min and post column detection (100 mM NaOH min^−1^). Sugar concentrations were calculated from standard curves created from external standards.

### 2.7 RNA extraction, RNAseq analysis, and defining MYB63 target genes

Total RNA was isolated from three replicated samples of several pooled leaves each of 4‐week‐old plants using TRIzol reagent (Life technologies). RNAseq was performed using an Ion AmpliSeq Transcriptome Gene Expression Kit on an ion torrent (Life Technologies) sequencing platform at the Next Generation Sequencing Centre at the University of British Columbia (http://ngs.med.ubc.ca/). Trimming and filtering of raw FASTQ reads were performed with trimmomatic v0.35 (Bolger, Lohse, & Usadel, 2014) using the following parameters; LEADING:20 TRAILING:20 AVGQUAL:20 MINLEN:40. Other parameters were kept at default. Surviving reads were aligned against the *A. thaliana* reference genome (TAIR10 genome release; Swarbreck, Wilks, & Lamesch, 2008) with bowtie2 v2.2.7 using the very sensitive‐ local parameter (Langmead & Salzberg, 2012). Count matrices were obtained with htseq‐count v0.6.1 with default parameters (Anders, Pyl, & Huber, 2015). Differential analysis and normalization of count data were performed with DESeq2 (Love, Huber, & Anders, 2014). False discovery rate (FDR) < 0.05 and an absolute log2 fold change >1.5 define differentially expressed (DE) genes between comparisons (*Pro35S:MYB63*/WT and *Pro35S:MYB63xPro35S:LAC17/WT*). Gene Ontology (GO) enrichment analysis was performed with agriGO with default settings (Du, Zhou, Ling, Zhang, & Su, 2010). GO terms having FDR < 0.05 were considered significantly enriched. DNA affinity purification sequencing motif peaks (fraction of reads in peaks, FRiP ≥5%) for MYB63 were obtained from O’Malley et al. (2016). Differentially expressed genes whose promoter region (1.5 kb upstream of transcription start site) contains MYB63 motif peaks are defined as high‐confidence MYB63 target genes in this study. The raw sequence reads were deposited in NCBI Sequence Read Archive (http://www.ncbi.nlm.nih.gov/sra).

## 3 RESULTS

### 3.1 Overexpression of MYB58 and MYB63 produces dwarf plants with intact xylem

**FIGURE 1:**
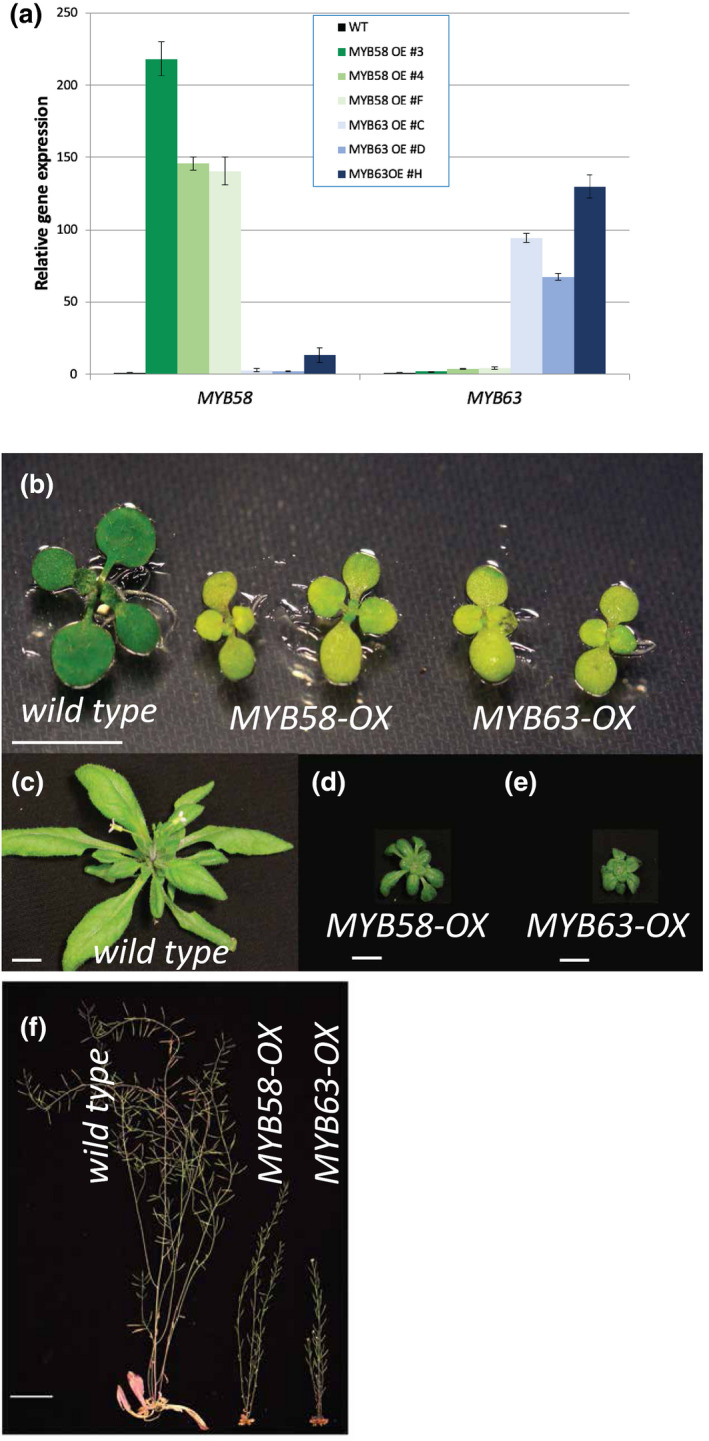
*Pro35S::MYB58* (*MYB58‐OX*) and *Pro35S::MYB63* (MYB63‐OX) plants display growth and development defects. (a) Real‐time quantitative PCR of the *MYB58* and *MYB63* genes from WT, *MYB58‐OX*, and *MYB63‐OX* genotypes. Error bars represent the standard error of three technical replicates. (b) 10‐day‐old seedlings of indicated genotypes. (c) 5‐week‐old wild‐type, (d) *MYB58‐OX* and (e) *MYB63‐OX* lines (d). (f) Mature, 10‐week‐old wild‐type, *MYB58‐OX*, and *MYB63‐OX* lines. Scale bars are 1 cm in (b–e), and 5 cm in F.

Wild‐type Columbia‐0 Arabidopsis plants (WT) were transformed with overexpression constructs consisting of the cauliflower mosaic virus *35S* promoter driving *MYB58* (*At1g16490*) or *MYB63* (*At1g79180*). Several lines with high levels of MYB gene expression were identified and quantitative real‐time PCR of these lines quantified the specific overexpression of either *MYB58* or *MYB63* in each respective line (Figure 1a). The overexpression of *MYB58* did not affect the levels of *MYB63*, and conversely overexpression of *MYB63* did not affect *MYB58* levels (Figure 1a). *MYB58‐OX* and *MYB63‐OX* lines were severely dwarfed during all stages of development (Figure 1b–f). The leaves of young seedlings that germinated on plates were pale and chlorotic, compared to WT, but pigmentation recovered after continued development on soil for several weeks (Figure 1c–e). At maturity, the *MYB58‐OX* and *MYB63‐OX* lines produced inflorescence stems that were shorter than the WT (Figure 1f). These data are consistent with the dramatic changes in growth and development, as well as ectopic deposition of lignin reported by Zhou et al. (2009), who discovered these monolignol‐associated transcription factors.

**FIGURE 2:**
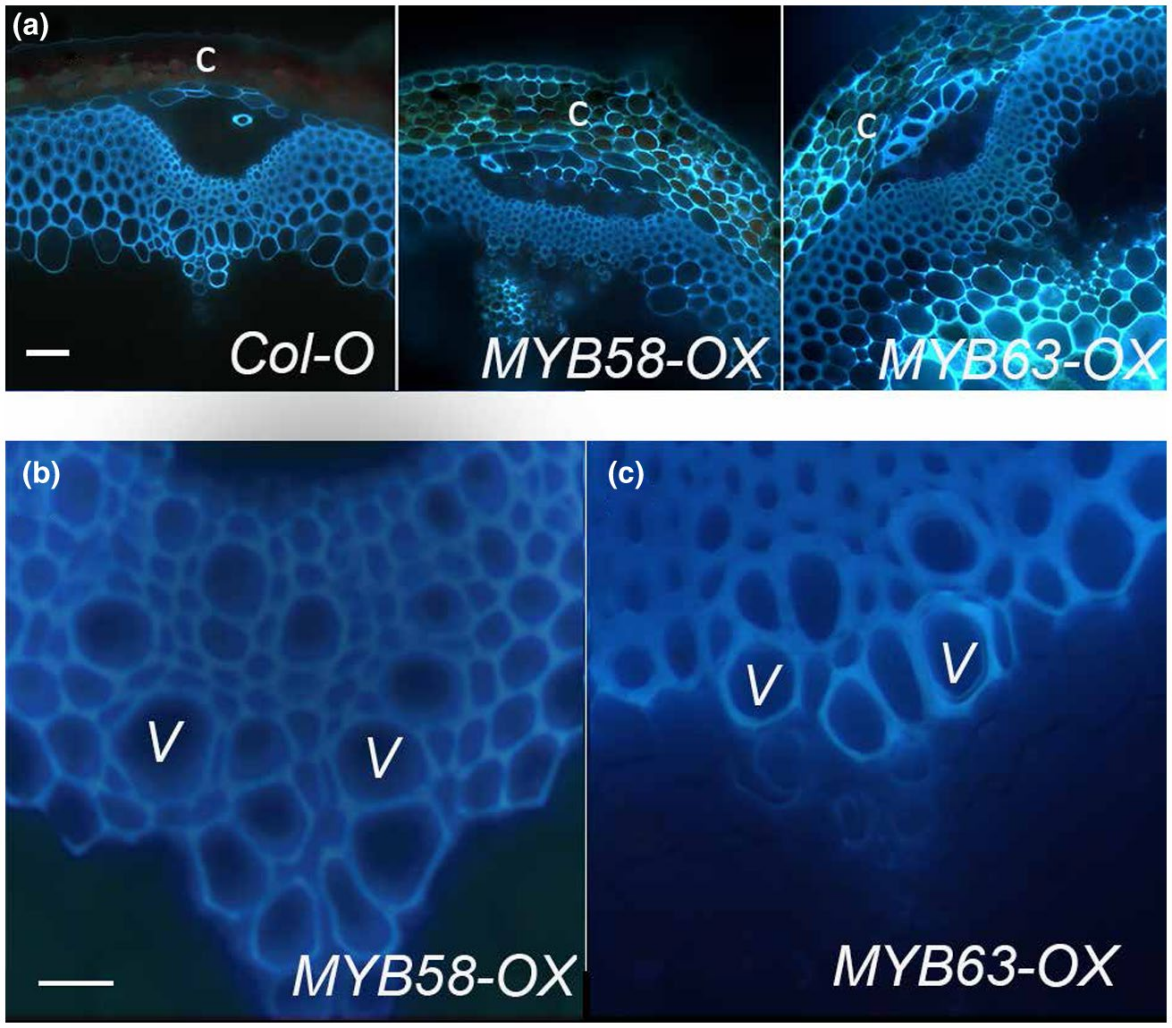
*Pro35S::MYB58 (MYB58‐OX)* and *Pro35S::MYB63 (MYB63‐OX)* inflorescence stems have regular xylem vessels and ectopic lignin. (a) UV autofluorescence of sections from the base of WT, *MYB58‐OX*, and *MYB63‐OX* inflorescence stems. Autofluorescence in wild‐type stems was observed in xylem, interfascicular fibers, and phloem fibers, while the autofluorescence in *MYB58‐OX* and *MYB63‐OX* spreads to the cortex (c), epidermis, and pith. (b) Vascular bundle of *MYB58‐OX* stem showing open xylem vessels. (c) *MYB63‐OX* stem showing open xylem vessels. V indicates xylem vessels. Scale bars are 50 µm.

In studies examining dwarfism associated with downregulation of lignin, one of the proposed mechanisms leading to decreased growth was insufficient reinforcement of the xylem vessels (Bonawitz & Chapple, 2013; DeMeester et al., 2018). In this study, the mature stems of WT, *MYB58‐OX*, and *MYB63‐OX* plants were examined using the intrinsic fluorescence of the lignin (Figure 2) or using Mäule staining (Figure S1). Ectopic lignin in the epidermis, cortex, and pith was observed in the *MYB58‐OX* and *MYB63‐OX* lines using autofluorescence (Figure 2a). Some lignification in the cortex of Mäule‐stained sections of *MYB63‐OX* was also observed (Figure S1). In all cases, the xylem vessels were regular, with open, round profiles (Figure 2b,c), unlike the irregular xylem of lignin‐deficient mutants (Bonawitz et al., 2014; Li et al., 2010). This suggests that the stunted growth of *MYB58‐OX* and *MYB63‐OX* is due to factors other than irregular xylem.

### 3.2 Increased monolignol glucosides and sinapoyl esters, but decreased flavonoids, in MYB58‐OX and MYB63‐OX lines

**FIGURE 3:**
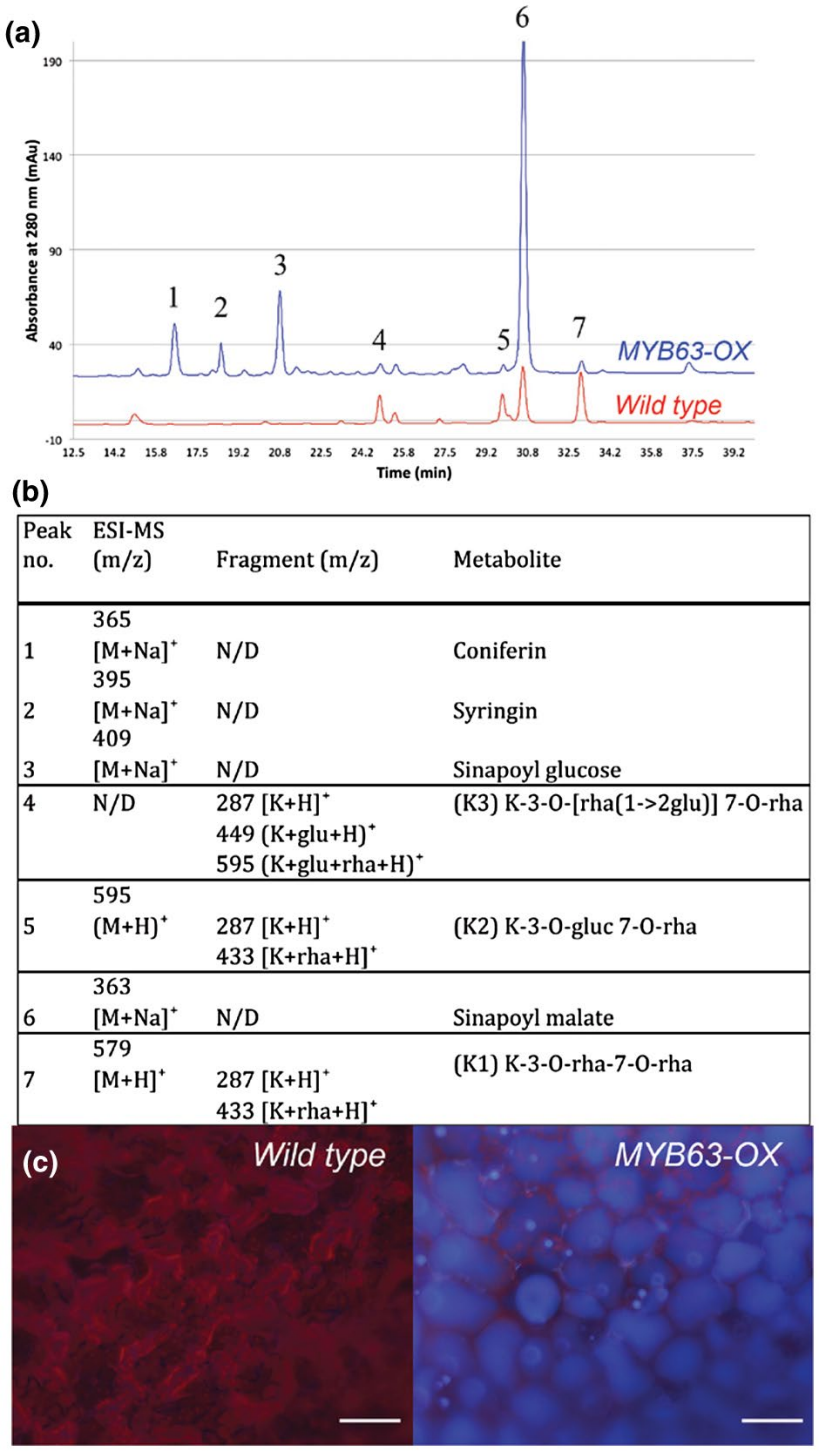
*Pro35S::MYB63 (MYB63‐OX)* plants accumulate increased monolignol glucosides and sinapoyl esters, in blue autofluorescent vacuoles. (a) HPLC chromatograph of methanol leaf extracts at 280 nm indicating the major phenolic metabolites accumulating in the *MYB63‐OX* overexpression lines. (b) LC‐MS identification of the soluble phenolic peak shown in A. K1, K2, and K3 indicate Kaempferol glycosides. (c) UV autofluorescence of chlorophyll wild‐type leaf mesophyll is red. UV autofluorescence of *MYB63*‐OX leaf mesophyll with blue vacuoles. Scale bars: 50 μm in (c).

**FIGURE 4:**
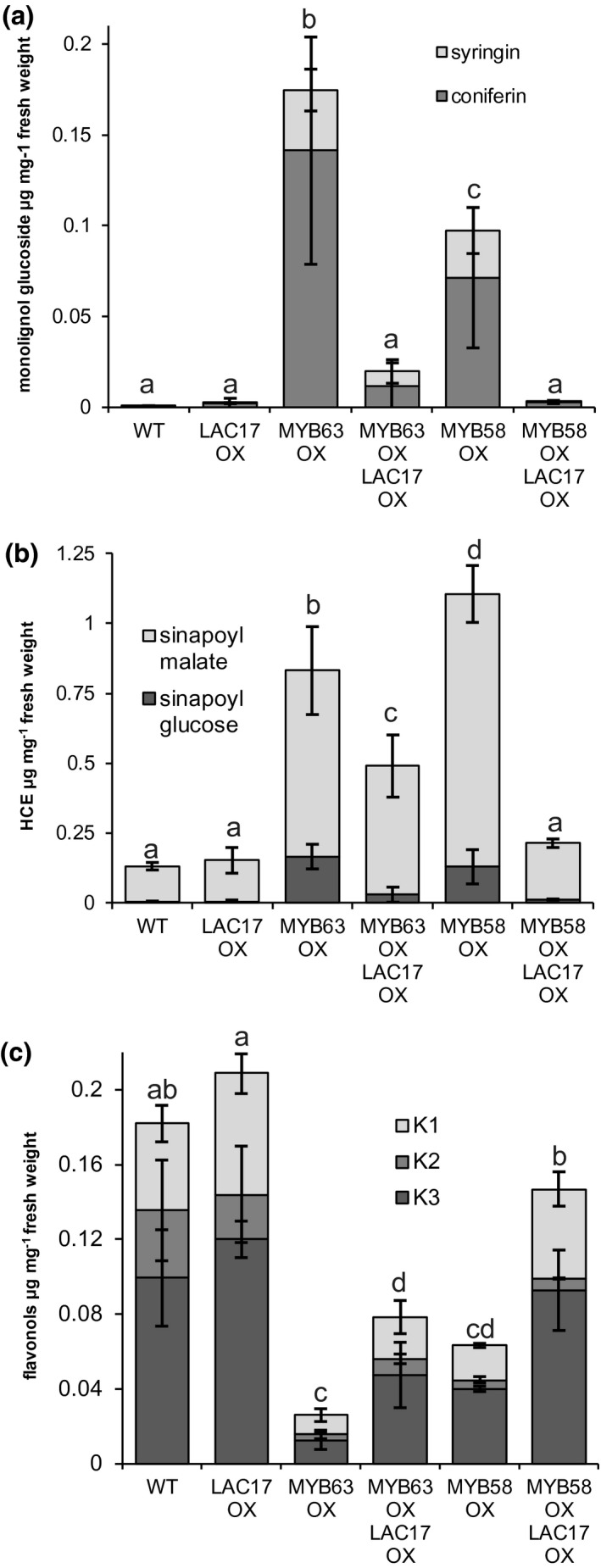
*Pro35S::MYB68* (*MYB58‐OX)* and *Pro35S::MYB63* (*MYB63‐OX)* plants disrupted soluble phenolic phenotype reversed with LAC17 co‐overexpression. Quantification of the soluble phenolics from Arabidopsis leaves of *MYB58‐OX* and *MYB63‐OX* overexpression lines, as well as *MYB58‐OX* or *MYB63‐OX* that co‐overexpress *Pro35S::LAC17* (*LAC17‐OX*). (a) monolignol glucosides, coniferin and syringin; (b) hydroxycinnamate esters (HCE), sinapoyl glucose and sinapoyl malate; and (c) kaempferol glycosides. Bars indicate standard deviation. Three replicate experiments. *n* = 3–6 pooled samples of five leaves from five plants. Means with different letters represent statistically significant differences of total monolignol glucosides, HCEs, or flavonols (Tukey's pairwise comparison, *p* < .01).

The consequences of *MYB58* or *MYB63* overexpression on the soluble phenolic metabolites were not previously examined. Based on their ectopic lignification, we hypothesized that these lines also have elevated levels of monolignols, which could manifest in high monolignol glucoside levels (LeRoy, Huss, Creach, Hawkins, & Neutelings, 2016). The appearance of monolignol glucosides is proposed to be a homeostatic mechanism used by plant cells to balance monolignol metabolism (Lin et al., 2016), analogous to hexosylation of xenobiotics (Vanholme et al., 2012). HPLC‐MS of extracted soluble phenolics from *MYB63‐OX* leaves was used to examine the major peaks of soluble phenolics (Figure 3a) and identify them based on mass spectroscopy profiles (Figure 3b). In addition to altered phenolic profiles, the leaves of *MYB63‐OX* exhibit bright blue vacuolar autofluorescence (Figure 3c). Qualitatively, it was clear that there were increased accumulations of monolignol glucosides (Figure 3a), and this was confirmed quantitatively for both *MYB58‐OX* and *MYB63‐OX* (Figure 4a). Other phenylpropanoid‐related pathways were also upregulated, with corresponding increases in soluble hydroxycinnamate esters such as sinapoyl glucose and sinapoyl malate (Figures 3a and 4b). As predicted by the upregulation of genes of the phenylpropanoid and monolignol biosynthetic pathways, monolignol glucosides and sinapoyl esters were strongly upregulated in the accumulation in the *MYB58‐OX* and *MYB63‐OX* lines.

While monolignol and hydroxycinnamoyl esters increased, kaempferol glucosides decreased in *MYB58‐OX* and *MYB63‐OX* lines, as might be predicted for a competing metabolic pathway for flavonol biosynthesis (Figures 3a and 4c). Decreased flavonoid production is consistent with the observation that *MYB58‐OX* and *MYB63‐OX* lines did not produce anthocyanin pigments under light stress (Figure S2a). In order to quantify anthocyanins, we used photometric quantification of anthocyanin extracts from 3‐week‐old seedlings, as previously described (Mehrtens, Kranz, Bednarek, & Weisshaar, 2005). Anthocyanin quantification of methanol extracts from these plants showed a 50% reduction of anthocyanin content in *MYB58‐OX* and *MYB63‐OX* lines compared to WT, and extremely high anthocyanin content in *MYB75^PAP1D^* plants (Figure S2b–d). The *MYB75^PAP1D^* mutants were a positive control for the accumulation of anthocyanins (Borevitz, Xia, Blount, Dixon, & Lamb, 2000). Overall, there were decreased flavonol levels in both *MYB58‐OX* and *MYB63‐OX* lines.

In summary, the soluble phenolic changes detected in the *MYB58‐OX* and *MYB63‐OX* lines were consistent with strong, specific upregulation of the general phenylpropanoid and monolignol biosynthetic pathways, leading to monolignol glucoside and hydroxycinnamate ester accumulation in the vacuoles, accompanied by a concomitant decrease in flavonoids.

### 3.3 Co‐overexpression of lignin‐related LACCASES in MYB58‐OX and MYB63‐OX lines makes soluble phenolic pools similar to wild type

Accumulations of monolignol glucosides, seen here in the *MYB58‐OX* and *MYB63‐OX* lines, were also observed in *lac11 lac4 lac17* triple mutants (Zhao et al., 2013), which were severely impaired in lignification and dwarfed. We hypothesized that overexpression of lignin‐related laccases such as *LAC4* or *LAC17* could lead to reduction of monolignol glucosides in the *MYB58‐OX* and *MYB63‐OX* lines. Plants were transformed with a construct containing the *35S* promoter driving overexpression of either *LAC17* (*Pro35S::LAC17*, *LAC17‐OX*) or *LAC4* (*Pro35S::LAC4*, *LAC4‐OX)*, with overexpression confirmed by RT‐PCR (data not shown). These plants were crossed with the *MYB63‐OX* lines, and RT‐PCR identified plants that co‐overexpressed both the laccase and the transcription factor, for example, *Pro35S::MYB58 (MYB58‐OX/LAC17‐OX)* and *Pro35S::MYB63 Pro35S:LAC17 (MYB63‐OX/LAC17‐OX)*. Quantification of the soluble phenolics by HPLC‐MS indicated that the monolignol glucosides were reduced to WT levels in the double overexpression lines compared to the single overexpression lines (Figure 4a). In both *MYB58‐OX/LAC17‐OX* and *MYB63‐OX/LAC17‐OX* lines, the sinapoyl ester levels were also reduced compared to the single overexpression lines (Figure 4b). Interestingly, in the *MYB63‐OX* background, sinapoyl ester levels were elevated above WT levels, indicating continued increased flux through the phenylpropanoid pathway. The suppression of the kaempferol glycosides observed in the *MYB58‐OX* and *MYB63‐OX* lines was also reversed in the *MYB58‐OX/LAC17‐OX* and *MYB63‐OX/LAC17‐OX* lines (Figure 4c). Identical results were observed when *LAC4* or *LAC17* was co‐overexpressed with *MYB63* (Figure S3). Clearly, the co‐overexpression of either lignin‐related *LAC4* or *LAC17* in the *MYB58‐OX* and *MYB63‐OX* lines had a strong impact on the soluble phenolics, shifting them toward a WT profile.

### 3.4 Cell wall composition is restored by co‐overexpression of LAC17 in MYB58‐OX and MYB63‐OX backgrounds

**FIGURE 5:**
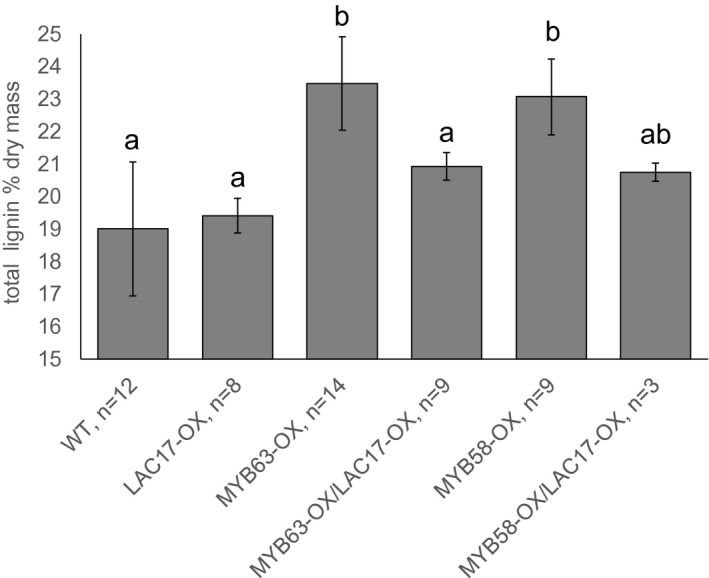
Total lignin levels are high in *Pro35S::MYB58* (*MYB58‐OX)* and *Pro35S::MYB63* (*MYB63‐OX)* stems, but similar to wild type when LAC17 is co‐overexpressed. Stem lignin was determined from the following overexpression lines: *MYB58‐OX*, *MYB63‐OX*, *LAC17‐OX*; and the co‐overexpression lines: *MYB63‐OX/LAC17‐OX* and *MYB58‐OX/LAC17‐OX*. Bars indicate standard deviation. Means with different letters represent statistically significant differences. One to three batches of ~90 plants were split into two to four technical replicates each for total *n* = 3–14 (Dunn's pairwise comparison, *p* < .05).

In addition to reverting the phenolic profiles toward WT levels, ectopic lignin (detected by Mäule staining) found in dwarf *MYB63‐OX* line disappeared in the double *MYB63‐OX/LAC17‐OX* overexpression lines (Figure S1). This was consistent with the cell wall lignin content, as *MYB63‐OX* and *MYB58‐OX* lines had higher total lignin levels than WT, but the double *MYB63‐OX/LAC17‐OX* and *MYB58‐OX/LAC17‐OX* overexpression lines were similar to WT levels (Figure 5). This result seems counterintuitive, as the upregulation of monolignol biosynthesis, paired with increased oxidative enzymes ectopically expressed throughout the plant, would be predicted to increase, not decrease, total cell wall lignin levels. The wild‐type cell wall phenotype in the rescued lines, compared to the dwarf *MYB63‐OX* lines, was also observed in the structural cell wall polysaccharides (Figure S4).The severely dwarfed *MYB63‐OX* line had the largest deviations in carbohydrate monomer composition, compared to WT, with significantly elevated arabinose and galactose and reduced mannose, glucose, and xylose. The *LAC17‐OX*, *MYB58‐OX*, *MYB58‐OX/LAC17‐OX*, and *MYB63‐OX/LAC17‐OX* were similar or indistinguishable from WT (Figure S4). Thus, for both cell wall lignin and carbohydrates, co‐overexpression of *LAC17* in *MYB63‐OX* lines led to cell wall composition similar to wild type.

### 3.5 Co‐overexpression of lignin‐related LACCASES rescues growth in MYB58‐OX and MYB63‐OX lines

**FIGURE 6:**
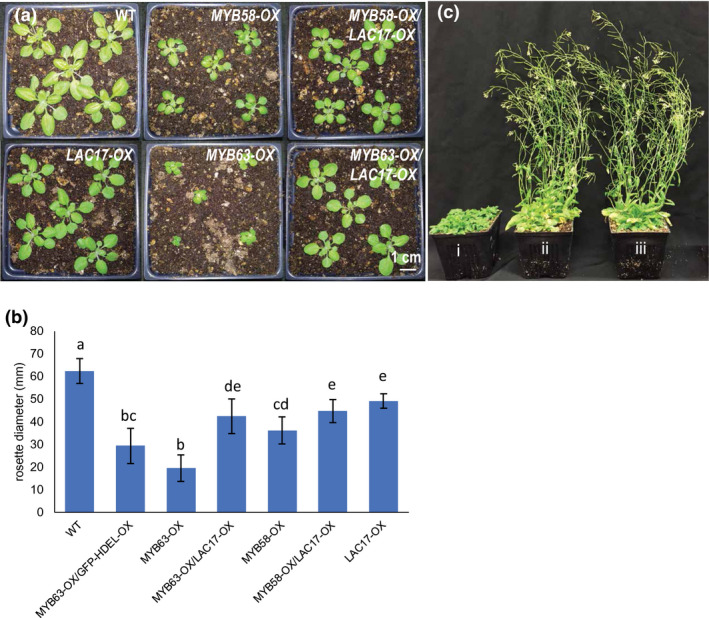
*Pro35S::MYB58* (*MYB58‐OX)* and *Pro35S::MYB63* (*MYB63‐OX)* dwarfism can be rescued by concurrent ectopic LAC17 overexpression. (a) Rosettes of wild type, *MYB58‐OX*, *MYB63‐OX*, *MYB58‐OX/LAC17‐OX*, and *MYB63‐OX/LAC17‐OX* Arabidopsis lines at 17 days old. (b) Quantification of rosette diameters at 17 days old (*n* = 9 plants except *MYB63‐OX GFP‐HDEL‐OX* *n* = 6 plants). Bars indicate standard deviation. Means with different letters represent statistically significant differences (Tukey's pairwise comparison, *p* < .01) 3 replicated experiments. (c) After 5 weeks of growth, while *MYB63‐OX* are at rosette stage (i), *MYB63‐OX/LAC17‐OX* double overexpression lines (ii) and wild type (iii) are flowering.

**FIGURE 7:**
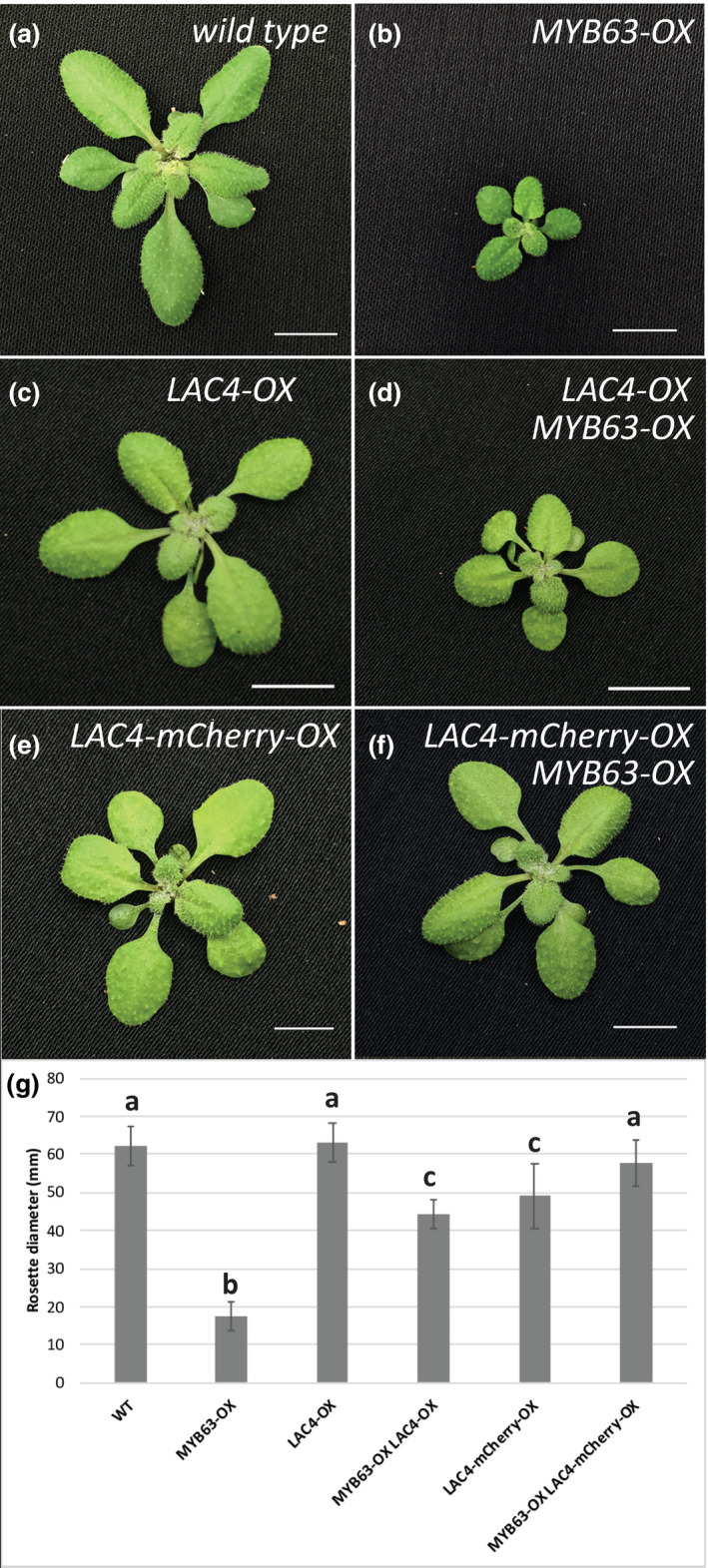
LAC4 overexpression rescues *Pro35S::MYB63 (MYB63‐OX*) growth phenotype. (a,c,e) Wild‐type and *MYB63‐OX* control Arabidopsis rosettes have similar growth to single overexpression *Pro35S::LAC4 (LAC4‐OX)* and *ProUBQ10::LAC4‐mCherry (LAC4‐mCherry‐OX)*. *MYB63‐OX* dwarfism (B) was rescued in (d) double overexpression lines *LAC4‐OX/MYB63‐OX* and (e) *LAC4‐mCherry‐OX/MYB63‐OX* (scale bar is 1 cm). (g) Quantified rosette diameters of the above lines. Bars indicate standard deviation. Means with different letters represent statistically significant differences (*n* = 12 rosettes, Tukey's pairwise comparison, *p* < .05).

**FIGURE 8:**
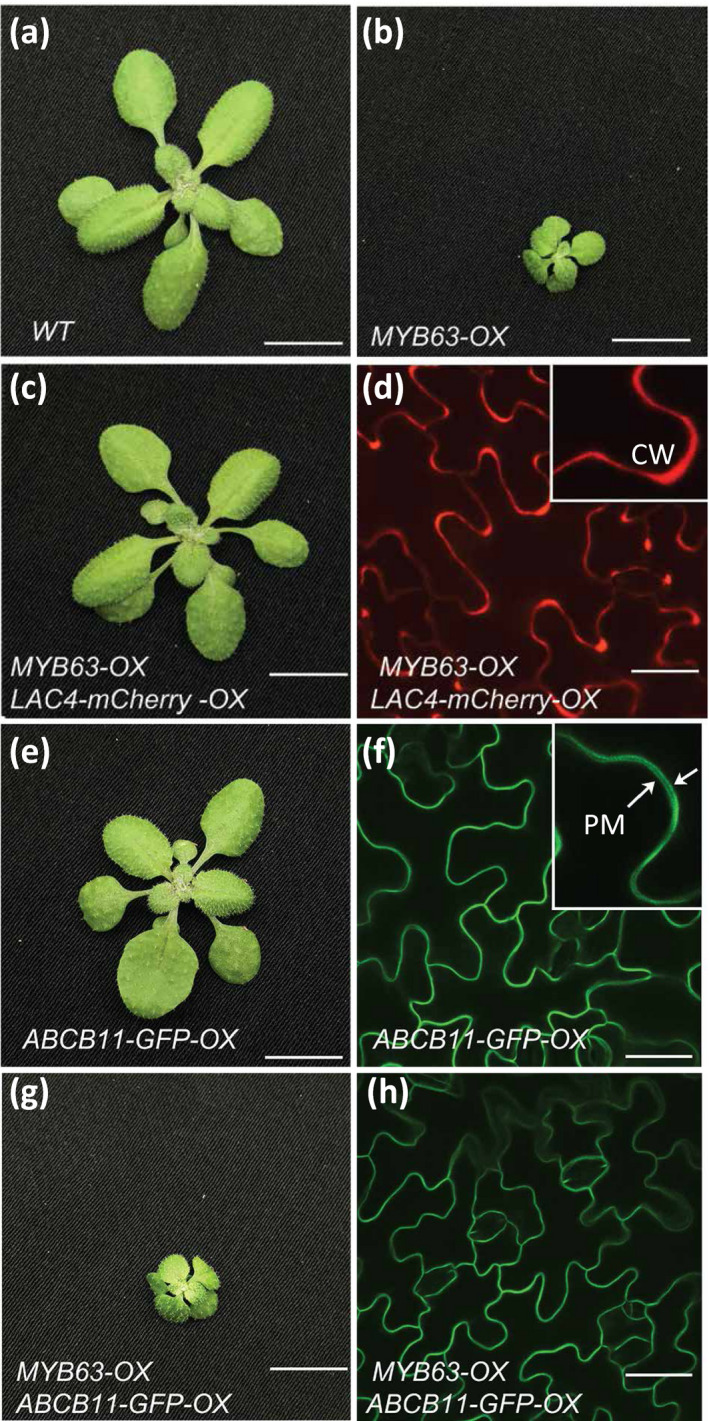
LAC4‐mCherry localized in the cell wall in rescued *Pro35S::MYB63 (MYB63‐OX*) lines. (a) Wild‐type Arabidopsis rosette. (b) Dwarf *MYB63‐OX* Arabidopsis rosette. (c) Wild‐type growth phenotype in double overexpression lines expressing *ProUBQ10::LAC4‐mCherry* (*LAC4‐mCherry‐OX*) in the *MYB63‐OX* background. (d) LAC4‐mCherry localized to the cell walls in *LAC4‐mCherry‐OX MYB63‐OX* lines. (e) Wild‐type growth phenotype in *Pro35S::ABCB11‐GFP* (*ABCB11‐GFP‐OX*) expressed in Col‐0 background. (f) Plasma membrane localization of ABCB11‐GFP. Arrows indicate plasma membrane of two cells separated by cell wall. (g) Dwarf *MYB63‐OX/ABCB11‐GFP‐OX lines*. (h) Plasma membrane localization of ABCB11‐GFP. Scale bars are 1 cm (a,b,c,e,g), 30 µm (d,f,h).

In addition to restoring soluble phenolic and cell wall composition toward the WT levels, growth was dramatically altered when *LAC4* or *LAC17* was co‐overexpressed in the *MYB58‐OX* and *MYB63‐OX* lines. *MYB63‐OX/LAC17‐OX* and *MYB58‐OX/LAC17‐OX* double overexpression lines displayed rosette diameters that were statistically significantly larger than the single overexpression lines (Figure 6a,b). When the *MYB63‐OX/LAC17‐OX* double overexpression plants were bolting and setting seed, the dwarf *MYB63‐OX* lines were not yet forming inflorescence stems (Figure 6c). As a control for the possibility that the introduction of an additional *35S* expression construct interfered with the *MYB63‐*induced dwarfism, *MYB63‐OX* lines were also transformed with *Pro35S::GFP‐HDEL*, which localizes GFP to the endomembrane system, and the LMID of plants was not rescued (Figure 6b). In addition to rescue by *LAC17* overexpression, when *LAC4* was co‐overexpressed (*Pro35S::LAC4*) in the *MYB63‐OX* lines, growth was significantly increased compared to the dwarf *MYB63‐OX* lines (Figure 7). This was also the case when LAC4, tagged with a red fluorescent protein (*ProUbiquitin10::LAC4‐mCherry*), was co‐overexpressed in the *MYB63‐OX* lines, where it rescued the growth back to WT (Figure 7e–g). In this line, LAC4‐mCherry was localized via spinning disk confocal microscopy to the cell walls of leaf epidermal cells and seedlings (Figure 8), demonstrating that the laccase protein was produced and targeted to its correct cell wall location (Figure 8d). The cell wall localization was confirmed with plasmolysis: treatment with 0.4 M mannitol led to shrinkage of the protoplast from the cell wall, yet the LAC4‐mCherry signal remained associated with the wall (Figure S5). As a control, the plasma membrane marker ABCB11‐GFP (*Pro35S::ABCB11‐GFP*; Figure 8e–h) was introduced into the *MYB63‐OX* background. ABCB11‐GFP expression and localization to the plasma membrane were confirmed using spinning disk confocal microscopy, and the resulting co‐overexpression lines remained severely dwarfed as predicted (Figure 8g‐h). Using the ABCB11‐GFP to outline the cell diameter, the difference in cell sizes in the dwarf co‐overexpression lines is apparent (Figure 8h). The impaired growth phenotype associated with upregulation of the phenylpropanoid and monolignol pathways was rescued exclusively by extracellular laccases, suggesting that by the presence of the oxidative cell wall enzyme changed intracellular conditions associated with the observed impaired plant growth.

Other hypotheses related to LMID plants from previous studies were tested using the more severe dwarf growth phenotype of MYB63‐OX lines. To test the potential contribution of flavonoids to the reduced growth of MYB63‐OX lines, flavonoid biosynthetic mutant *transparent testa4*, *tt4‐2*, deficient in chalcone synthase (Burbulis, Lacobucci, & Shirley, 1996), were transformed with the *35S::MYB63* overexpression construct. These plants showed the same dwarf growth habit and blue fluorescent vacuoles as the *35S::MYB63* in the Col‐0 WT background (Figure S6a). These data provide an independent line of evidence that the decreased growth in Arabidopsis associated with altered phenolics is independent of flavonoids, as demonstrated for *HCT‐RNAi* and *c3’h* mutants by Li et al. (2010). To test if the Mediator complex subunits 5A and 5B are required for the dwarf phenotype (Bonawitz et al., 2014), *med5a/5b* mutants were transformed with the *35S::MYB63* overexpression construct. The resulting transformants were identical to dwarf *MYB63‐OX* lines (Figure S6b). These data demonstrate that the dwarf growth of *MYB63‐OX* plants is independent of flavonoid‐mediated growth modulation proposed for loss of function *hct* lines, and the Mediator 5a5b‐dependent mechanisms that rescue *c3’h* mutants.

The rescue of the growth phenotype was not due to co‐suppression of *MYB63*, as transcript levels of *MYB63* remain high in the double overexpression lines. When *MYB63* expression was measured with quantitative RT‐PCR, both *MYB63‐OX/LAC4‐OX* and *MYB63‐OX/LAC17‐OX* lines had *MYB63* gene expression ranging from 21‐ to 1683‐fold greater than WT levels (gray boxes in Figure S7). While there was a clear correlation between rosette size and *MYB63* gene expression in the single *MYB63‐OX* lines (yellow triangle in Figure S7) and WT (yellow circles in Figure S7), there was no correlation between rosette size and *MYB63* transcript levels in the *MYB63‐OX/LAC17‐OX* or *MYB63‐OX/LAC4‐OX* double overexpression plants (gray and black squares in Figure S7).

**FIGURE 9:**
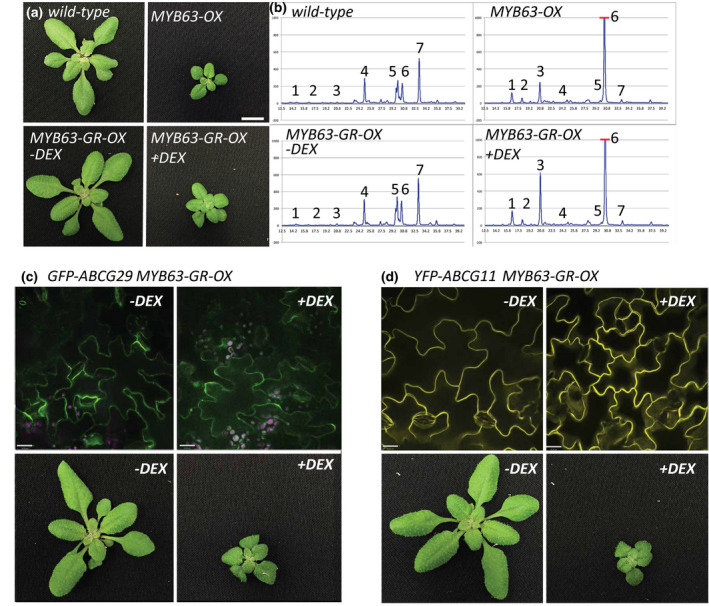
Co‐overexpression of ABC transporters does not suppress the dwarf growth phenotype of *Pro35S::MYB63 (MYB63‐OX*) lines. (a) Control wild‐type and dwarf *MYB63‐OX* Arabidopsis rosettes (top) compared to plants overexpressing an inducible MYB63 construct, where MYB63 is fused to the VP16 activation domain and glucocorticoid receptor (*Pro35S::MYB63‐VP16‐GR*). Uninduced plants, *MYB63‐GR‐OX* without dexamethasone (−DEX) resemble wild type; plants with MYB63 activity induced by spraying dexamethasone (+DEX) have reduced growth similar to *MYB63‐OX*. After 5 days of growth on soil dexamethasone at a concentration of 10 μM in water was sprayed every 3–4 days. (b) Representative HPLC of soluble phenolic profiles show a wild‐type pattern for the uninduced MYB63‐GR‐OX and the characteristic high monolignol glucoside pattern from *MYB63‐OX* and the induced *MYB63‐GR + DEX* plants. (c) When ABCG29 (*Pro35S::GFP‐ABCG29*) is co‐overexpressed with MYB63 by crossing it with the inducible *MYB63‐GR‐OX* lines, growth is wild type in *‐*DEX controls and dwarf in + DEX‐induced plants. Spinning disk confocal of these lines demonstrates the GFP‐ABCG29 in the plasma membrane of leaf cells. (d) When the YFP‐ABCG11 (*Pro35S::YFP‐ABCG11*) is crossed into the inducible *MYB63‐GR‐OX* lines, growth is wild type in *‐DEX* controls and dwarf in + *DEX*‐induced plants. Spinning disk confocal of plasma membrane localization of GFP‐ABCG11. Scale bars for rosette images are 1 cm (a,c,d), and 30 µm for confocal images (c,d).

Several ATP‐binding cassette (ABC) transporters have been correlated with monolignol production (Kaneda et al., 2011; Takeuchi, Kegasa, Watanabe, Tamura, & Tsutsumi, 2018), and *ABCG29* was reported to transport *p*‐coumaryl alcohol (Alejandro et al., 2012). To test if putative monolignol exporters were able to rescue the growth phenotype of *MYB63‐OX* lines, we co‐overexpressed *ABCB11* and *ABCG29* with *MYB63*. Due to difficulty recovering *ABCG29* transporter/*MYB63* double overexpression lines, plants carrying an inducible *MYB63* overexpression construct were also generated by exchanging the *VND7* coding sequence with the *MYB63* coding sequence in the *Pro35::VND7‐VP16‐GR* vector described in Yamaguchi et al. (2010; *MYB63‐VP16‐GR*). When young seedlings were sprayed with dexamethasone to activate the inducible system, a dwarf phenotype, and accumulation of monolignol glucosides, which were identical to those found in *Pro35S::MYB63* lines, was observed (Figure 9a–b). When the inducible *MYB63‐VP16‐GR* construct was introduced into plants overexpressing *Pro35S::GFP‐ABCG29* (Figure 9c), there was no rescue of the dwarf growth phenotype induced by the dexamethasone treatment, although the ABC transporters were properly localized to the plasma membrane (Figure 9c). Overexpression of the lipid‐transport ABC transporter, *ABCG11* (*Pro35S::YFP‐ABCG11*; (Bird, Beisson, & Brigham, 2007), was similarly unable to rescue the *MYB63‐OX* dwarfism in the inducible lines (Figure 9d).

Thus, of all the ectopic proteins that we co‐overexpressed in *MYB63‐OX* lines, only LAC4 and LAC17 (with or without fluorescent tags) were capable of reversing the dwarf phenotype of *MYB63‐OX*. Conversely, loss of function of *LAC4* and *LAC17* did not modify the *MYB63‐OX* phenotype. When the laccase double *lac4 lac17* mutant (Berthet et al., 2011) was transformed with the *35S::MYB63* overexpression construct, the transformants also showed dwarf growth similar to *MYB63‐OX* lines (Figure S6c).

### 3.6 Co‐overexpression of lignin‐related LACCASES in MYB58‐OX and MYB63‐OX lines shifts the transcriptome from stressed to wild‐type

**FIGURE 10:**
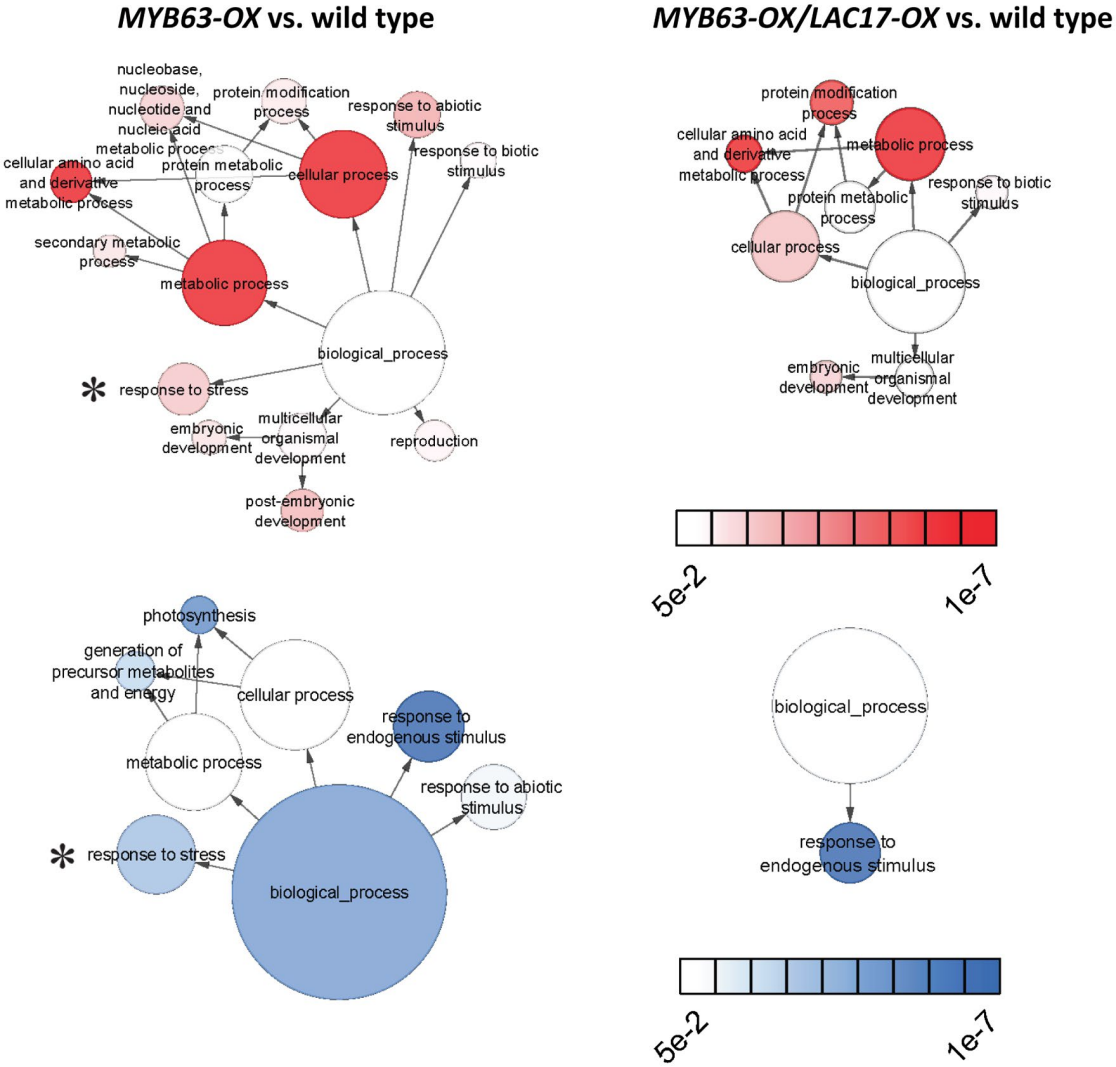
Gene Ontology (GO) enrichment analysis in the transcriptome of *MYB63‐OX* compared to wild‐type Arabidopsis rosette leaves or *MYB63‐OX*/*LAC17‐OX* double overexpression lines compared to wild type. Gene Ontology (GO) enrichment analysis, with GO SLIM terms having FDR < 0.05 considered significantly enriched. Dwarf *MYB63‐OX* (left) have more up‐ and downregulated GO terms than rescued *MYB63‐OX/LAC17‐OX* (right) lines, details in Table S2. Response to stress indicated by a star.

**FIGURE 11:**
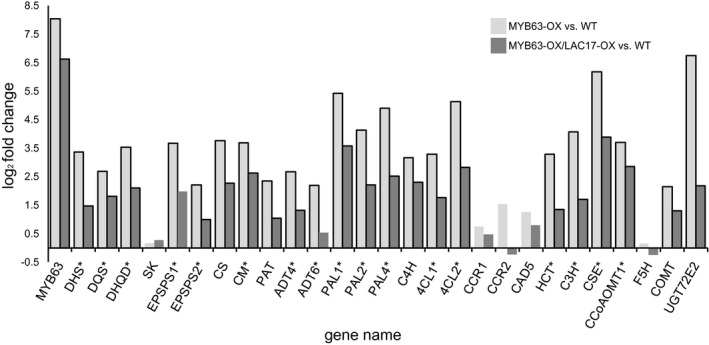
Transcriptomic analysis of key monolignol biosynthetic genes in *MYB63‐OX* and *MYB63‐ OX/LAC17‐OX* leaves compared to wild type. Expression of genes encoding the Arabidopsis monolignol biosynthetic pathway in MYB63‐OX (light gray) and in double overexpression, MYB63‐OX/LAC17‐OX (dark gray), lines. Outlined bars indicate transcript levels significantly different from wild type (*p* < .05). Genes that are direct MYB63 targets according to O’Malley et al., 2016 are demarcated by a star.

Transcriptomic analysis was used to obtain a global view of the changes in gene expression underlying the dwarf phenotype in *MYB63*‐*OX* lines compared to wild type. The overexpression of the *MYB63* in both the *MYB63‐OX* overexpression and the *MYB63‐OX/LAC17‐OX* co‐overexpression lines was verified in the RNA‐seq data (Figure S8a). Over 600 genes were upregulated and almost 1,600 downregulated in the dwarf *MYB63‐OX* lines compared to WT plants (Figure S8b, Table S2). Overall, the transcriptomic analysis highlights a significant upregulation of gene ontology terms relating to phenylpropanoid metabolic processes, including aromatic amino acid biosynthesis (*p* < 5E‐12; Figure 10, Table S3). There were also increases in expression of genes in GO categories “response to stress” and “response to abiotic stimulus” in the dwarf *MYB63‐OX* line compared to the rescued double *MYB63‐OX/LAC17‐OX* lines (Figure 10, Table S2). The ability of purified MYB63 to bind to sites in the Arabidopsis genome was previously mapped by O’Malley et al. (2016) using in vitro DNA affinity purification sequencing. There is strong overlap between that *MYB63* target dataset and the genes modulated in the *MYB63‐OX* line in this study (see starred genes in Figure 11; column A in Table S1). Transcriptomes of the rescued double *MYB63‐OX/LAC17‐OX* overexpression lines against WT showed that all of the direct target genes for MYB63 had significantly higher expression than WT (Figure 11, Table S3). This analysis confirmed MYB63 target genes continued to be upregulated, within the context of dramatic shifts in the transcriptome away from stress‐responsive genes. The ability of additional laccase outside the cell membrane to trigger dramatic transcriptional shifts suggests monolignol oxidation outside the membrane changes the phenolic pools inside the cells.

## 4 DISCUSSION

This study demonstrates that when plants have constitutive expression of phenylpropanoid and monolignol pathway genes due to MYB58 or MYB63 overexpression, there is a strong effect on the soluble phenolic metabolites and stress‐related gene expression in the transcriptome. The plants were dwarfed (Figure 1), with ectopic lignification (Figure 2), as described by Zhao et al. (2013), who identified these monolignol pathway regulatory transcription factors. In this work, *MYB58‐OX* and *MYB63‐OX* lines are used as a tool to probe the consequences of upregulation of monolignol biosynthesis. In *MYB63‐OX* and *MYB58‐OX* lines, bright blue autofluorescence in the vacuoles (Figure 3) was correlated with increased soluble phenolic profiles, with a characteristic upregulation of monolignol glucosides (Figure 4). Given that increased monolignol glucoside levels are correlated with increases in the monolignol pools (LeRoy et al., 2016; Väisänen et al., 2015), we hypothesized that consumption of monolignols by lignin‐related laccases would lead to decrease monolignol glucoside levels in these plants. When either *LAC4* or *LAC17* were overexpressed in the high monolignol‐containing lines (*MYB58‐OX* or *MYB63‐OX*), not only were monolignol glucoside levels decreased (Figure 4) but also the impaired plant growth was rescued (Figures 6 and 7). Using RNA‐seq, we captured a more complete picture of the complex transcriptional changes elicited by *MYB63* overexpression and the co‐expression of *MYB63* and *LAC17*. While there were many groups of differentially expressed genes, the stress‐associated genes were strongly differentially expressed in dwarfed lines compared to rescued lines (Figure 10). The addition of LAC17 seems to broadly alleviate stress and dwarfism, and also alleviates the overaccumulation of soluble phenolics. It is difficult, however, to disentangle the possible effect(s) of the various stress‐induced signaling pathways from those of the direct effects of LAC17 in the cell wall. Taken together, the transcriptional landscape of both lines suggests the potential involvement of many pathways, whose connection to lignin‐associated dwarfism is yet to be elucidated fully. How the overexpression of a cell wall oxidative enzyme changes the cellular phenolic profiles to have an impact on LMID is unclear, but it does permit us to critically evaluate some of the hypotheses proposed to explain LMID.

It has been suggested that one factor contributing to LIMD could be the disruption of the normal geometry and physiological functioning of xylem vessels (Bonawitz & Chapple, 2013; DeMeester et al., 2018). However, *MYB58* and *MYB63* overexpression lines do not have collapsed or irregularly shaped xylem vessels (Figure 2). As with some severe monolignol biosynthetic mutants (Panda et al., 2020), very young seedlings of *MYB63* and *MYB58* overexpressing plants are dwarfed even when grown inside highly moist sterile petri dishes where water should be readily available to all cells of the plant (Figure 1b), which also supports the conclusion that dwarfism in this case is due to other factors. In poplar, downregulation of C3’H resulted in irregular xylem, but measurement of physiological parameters such as xylem pressure potential and water‐use efficiency was not consistent with a simple explanation of water stress (Coleman, Samuels, et al., 2008b). As the obvious explanation of water stress is not sufficient, we considered a constellation of other correlated factors among dwarfed and rescued lines.

In our dwarfed and rescued lines, plant size and monolignol glucosides are inversely proportional, with soluble phenolic analyses showing the highest coniferin and syringin in the most dwarf MYB63 overexpression lines. The more modestly dwarfed MYB58 overexpression lines also have modestly elevated coniferin and syringin levels (Figure 4). In the double MYB and LAC17 overexpression lines, where growth was near WT (Figure 6), so too were the monolignol glucoside levels (Figure 4). Monolignol glucosides can accumulate as a method of detoxifying cytoplasmic accumulations of monolignols (Väisänen et al., 2015). When exogenous coniferyl alcohol was applied to BY‐2 cell cultures, levels of coniferin and other related phenolics were elevated (Väisänen et al., 2015). Considering that, and the generally very low levels of free monolignols in cells (Jaini, Wang, Dudareva, Chapple, & Morgan, 2017), one interpretation is that monolignols glucosides in the *MYB63‐OX* and *MYB58‐OX* lines are a reflection of elevated intracellular monolignols due to the upregulation of the lignin biosynthesis pathway. If abundant coniferin in the dwarf plants reflects high coniferyl alcohol levels in the cytoplasm, then lines with most severe dwarfism have the most abundant accumulations of G‐type soluble phenolics. This does not support the hypothesis that coniferyl alcohol‐derived growth‐promoting substances, such as dehydro‐diconiferyl glucosides (Binns, Chen, Wood, & Lynn, 1987; Lynn, Chen, Manning, & Wood, 1987), are playing a role in dwarfism in this case. Alternatively, it could be the case that the increase in glucosylation of monolignols limits the monolignols that could be oxidized and undergo combinatorial coupling to form dimers or oligolignols. Feeding experiments in Arabidopsis leaves have demonstrated the cytoplasmic oxidation and coupling of monolignols into oligomers in the cytoplasm (Dima et al., 2015). In the dwarf plants, tying up monolignols in the glucosylated form could occur at the expense of unknown dimers or oligolignols with growth‐promoting properties.

Transcriptional analysis points to monolignols and monolignol glucosides as key factors involved in dwarfism. MYB58 and MYB63 regulate a suite of genes responsible for monolignol biosynthesis, and our RNAseq transcriptomic data are consistent with earlier RT‐PCR data (Zhou et al., 2009). The RNAseq data also validate the in vitro DAP‐seq analysis of MYB63 transcription factor binding to gene promoters (O’Malley et al., 2016), which identified the core monolignol biosynthetic genes as direct binding targets of MYB63 (Figure 11). We see a significant upregulation of many of the core components of the monolignol biosynthetic pathway, as well as a glucosyltransferase (UGT72E2) implicated in the formation of coniferin (Lanot et al., 2006). Although they did not accumulate monolignol glucosides, there was a similar upregulation of the monolignol biosynthetic pathway and UGT72E2 in the double *MYB63‐OX LAC17‐OX* overexpression lines. This demonstrates that the upregulation of these genes by MYB63 was not abolished by LAC17 co‐overexpression and that the production of monolignols could continue at a level elevated above WT. This level was not high enough to trigger either dwarf growth or monolignol glucoside accumulation, suggesting the presence of the laccase led to lower cytoplasmic monolignol levels.

The drop in monolignol glucoside accumulation in *LAC17‐OX MYB63‐OX* lines is consistent with the established relationship between increased monolignol glucoside accumulation when laccases are knocked out (Zhao et al., 2013). However, the impact of an extracellular oxidative enzyme on the intracellular phenolics in the overexpression plants implies export of the cytoplasmic monolignols to the wall where the laccases are found (Figure 8). Given the recent in silico modeling indicating that diffusion of monolignols across lipid bilayers is a chemically and energetically plausible mechanism of transport (Vermaas et al., 2019), one mechanism explaining the drop of monolignols glucoside levels in the double overexpression plants would be the consumption of monolignols by laccases in the cell wall, creating a concentration gradients across the cell membrane followed by passive diffusion (Perkins et al., 2019). The elevated monolignols in the single *MYB63*‐*OX* lines overpowers the ability of cell wall‐localized oxidative enzymes to consume them, resulting in a buildup, glucosylation by UGT72E2, and their storage as glucosides to mitigate their toxicity. In the *MYB63‐OX LAC17‐OX* double overexpression lines, the additional copies of laccase may increase the strength of the extracellular sink shifting the balance of monolignols to primarily flowing out of the cell, alleviating the cytoplasmic toxicity. An alternative to this sink‐driven diffusion model is that monolignol transporters compete with the UGT72E2 for monolignol substrate in the *LAC17‐OX MYB63‐OX* lines, but no candidate transporters were identified in our transcriptome, and overexpression of several different ABC transporters did not rescue the *MYB63‐OX* dwarfism (Figure 9).

Alterations to the monolignol levels did not happen in isolation with respect to other soluble phenolics. In lines where monolignol glucosides were most elevated relative to WT, the levels of flavonols were depleted (Figure 4). Flavonol biosynthesis branches from the core monolignol biosynthesis pathway at *p*‐coumaroyl‐CoA (Fraser & Chapple, 2011). The inverse relationship between the two suggests that when monolignol production is favored by upregulation of the downstream monolignol pathway, the flux of *p*‐coumaroyl‐CoA is preferentially directed toward monolignols at the expense of flavonols. The intermediate levels of flavonols in the *MYB58‐OX LAC17‐OX* or *MYB63‐OX LAC17‐OX* lines compared to the single *MYB58‐OX* or *MYB63‐OX* lines mirror the other intermediate phenotypes in the double overexpressing lines. It appears that the hydroxycinnamate esters, sinapoyl malate and sinapoyl glucose, move in concert with the monolignol glucosides. Interestingly, this was also observed in *lac4 lac11 lac17* triple mutants, where accumulation of monolignol glucosides was associated with elevated hydroxycinnamoyl esters (Zhao et al., 2013). These consistent patterns support the view that complex cross‐talk exists in the phenylpropanoid and monolignol biosynthetic pathways as well as lignin polymerization mechanisms (Zhou et al., 2009).

The complexity of the soluble phenylpropanoid response was reflected in the unexpected result that lignin levels in the rescued double *MYB58‐OX LAC17‐OX* or *MYB63‐OX LAC17‐OX* lines were similar to wild type, not higher than the single overexpression lines (Figure 5). Given the presence of ectopic lignin (Figure 2, Figure S1), the MYB63 and MYB58 overexpressing lines were predicted to have increased levels of lignin above wild type. We observed greater ectopic lignin in the cortex, pith, and epidermis using intrinsic autofluorescence compared with Mäule stained sections. We ascribe this to the greater sensitivity of the intrinsic fluorescence that we observe, particularly with respect to lignified primary walls. The hypothesis that increased laccase in the wall increases the consumption of monolignols, thus normalizing the intracellular soluble phenolic profile, leads to the prediction that there would be a corresponding increase in lignin. The lignin levels found in the double overexpressing lines were indistinguishable from wild type. The combination of transcriptional changes and the lignin levels suggests that the presence of additional laccase puts the system into some kind of equilibrium that results in wild‐type lignin levels in spite of changes to the transcriptome, most notably the sustained upregulation of the lignin biosynthetic pathway. This is similar to the case of the *c3’h* mutants with decreased lignin levels, which could be rescued by the disruption of Mediator components *MED5a* and *MED5b* (Bonawitz et al., 2014). The quantity of lignin was restored to wild‐type levels, even though the composition of the lignin remained altered, consisting almost exclusively of H subunits (Bonawitz et al., 2014). This demonstrated that the plant was chemically and physiologically capable of tolerating the *C3’H* mutation, but that the severe phenotype was due to other signals dependent on MED5a/b. It may be that, in the case of *MYB58‐OX* and *MYB63‐OX*, the cause and alleviation of severe dwarf phenotypes may be similarly dependent on intracellular or cell wall homeostatic mechanisms, and independent from the bulk lignification effects of laccase.

Our study of LMID in the context of *MYB63*, *MYB58*, and *MYB/LAC* co‐overexpressing lines has directly answered some questions about dwarfism related to mis‐regulation of the lignin pathway, and raised the possibility of connections to other pathways and mechanisms worthy of further inquiry. We have ruled out the possibility that collapsed xylem is the cause of dwarfism in these lines. MYB/LAC4 or LAC17 co‐overexpression was able to normalize the hyperaccumulation of monolignol glucosides and restore growth, which is not consistent with dwarfism being due to loss of a monolignol‐derived growth factor. Transcriptional analysis suggests that broad transcriptional changes are elicited by both the overexpression of *MYB63* and the co‐overexpression of *MYB63* and *LAC17*, with the most striking difference between the two being stress‐associated transcripts associated with dwarfism. We have not fully determined the mechanisms and signaling pathways responsible for the dramatic changes in phenotype that we observe. It is likely that other factors are involved, which may overlap with unknown factors implicated in other related studies of LMID‐associated phenotypic rescues (Bonawitz et al., 2014; Gallego‐Giraldo et al., 2020; Panda et al., 2020).

## AUTHOR CONTRIBUTIONS

M.L.P, M.S, and L.S. designed the research. M.L.P, M.S., F.U., R.A.S., R.S., and N.J.H. conducted experiments and analyzed the data. D.C.J.W. performed bioinformatic analysis. S.D.C, S.D.M, and L.S. supervised experiments. M.L.P. and L.S. wrote the article with contributions from all authors.

## Accession Numbers

Sequence data from this article can be found in the GenBank data library under accession numbers *MYB63*, *AT1G79180*; *MYB58*, *AT1G16490*; *LAC4*, *AT2G38080*; *LAC17*, *AT5G60020*. The BioProject and SRA accession for the RNAseq data are PRJNA345391 and SRP090868, respectively.

## Supporting information

Fig S1‐S8

Table S1‐S4

Supplementary Material

## References

[pld3265-bib-0001] Alejandro, S. , Lee, Y. , Tohge, T. et al (2012). AtABCG29 is a monolignol transporter involved in lignin biosynthesis. Current Biology, 22, 1207–1212. 22704988 10.1016/j.cub.2012.04.064

[pld3265-bib-0002] Anders, S. , Pyl, P. T. , & Huber, W. (2015). HTSeq–a Python framework to work with high‐throughput sequencing data. Bioinformatics, 31, 166–169. 10.1093/bioinformatics/btu638 25260700 PMC4287950

[pld3265-bib-0003] Berthet, S. , Demont‐Caulet, N. , Pollet, B. et al (2011). Disruption of LACCASE4 and 17 results in tissue‐specific alterations to lignification of Arabidopsis thaliana stems. The Plant Cell, 23, 1124–1137. 21447792 10.1105/tpc.110.082792 PMC3082258

[pld3265-bib-0004] Besseau, S. , Hoffmann, L. , Geoffroy, P. , Lapierre, C. , Pollet, B. , & Legrand, M. (2007). Flavonoid accumulation in Arabidopsis repressed in lignin synthesis affects auxin transport and plant growth. The Plant Cell, 19, 148–162. 17237352 10.1105/tpc.106.044495 PMC1820963

[pld3265-bib-0005] Binns, A. N. , Chen, R. H. , Wood, H. N. , & Lynn, D. G. (1987). Cell division promoting activity of naturally occurring dehydrodiconiferyl glucosides: Do cell wall components control cell division? Proceedings of the National Academy of Sciences of the United Science of America, 84, 980–984. 10.1073/pnas.84.4.980 PMC304345 3469655

[pld3265-bib-0006] Bird, D. , Beisson, F. , Brigham, A. et al (2007). Characterization of Arabidopsis ABCG11/WBC11, an ATP binding cassette (ABC) transporter that is required for cuticular lipid secretion. The Plant Journal, 52, 485–498. 17727615 10.1111/j.1365-313X.2007.03252.x

[pld3265-bib-0007] Blokhina, O. , Laitinen, T. , Hatakeyama, Y. et al (2019). Ray parenchymal cells contribute to lignification of tracheids in developing xylem of Norway SpRuce. Plant Physiology, 181, 1552–1572. 31558578 10.1104/pp.19.00743 PMC6878020

[pld3265-bib-0008] Bolger, A. M. , Lohse, M. , & Usadel, B. (2014). Trimmomatic: A flexible trimmer for Illumina sequence data. Bioinformatics, 30, 2114–2120. 10.1093/bioinformatics/btu170 24695404 PMC4103590

[pld3265-bib-0009] Bonawitz, N. D. , & Chapple, C. (2013). Can genetic engineering of lignin deposition be accomplished without an unacceptable yield penalty? Current Opinion in Biotechnology, 24, 336–343. 23228388 10.1016/j.copbio.2012.11.004

[pld3265-bib-0010] Bonawitz, N. D. , Kim, J. I. , Tobimatsu, Y. , Ciesielski, P. N. , Anderson, N. A. , Ximenes, E. , … Chapple, C. (2014). Disruption of Mediator rescues the stunted growth of a lignin‐deficient Arabidopsis mutant. Nature, 509, 376–380. 10.1038/nature13084 24670657

[pld3265-bib-0011] Borevitz, J. O. , Xia, Y. , Blount, J. , Dixon, R. A. , & Lamb, C. (2000). Activation tagging identifies a conserved MYB regulator of phenylpropanoid biosynthesis. The Plant Cell, 12, 2383–2393. 10.1105/tpc.12.12.2383 11148285 PMC102225

[pld3265-bib-0012] Burbulis, L. E. , Lacobucci, M. , & Shirley, B. W. (1996). A null mutation in the first enzyme of flavonoid biosynthesis does not affect male fertility in Arabidopsis. The Plant Cell, 8, 1013–1025. 8672888 10.1105/tpc.8.6.1013 PMC161155

[pld3265-bib-0013] Chen, F. , & Dixon, R. A. (2007). Lignin modification improves fermentable sugar yields for biofuel production. Nature Biotechnology, 25, 759–761. 10.1038/nbt1316 17572667

[pld3265-bib-0014] Chou, E. , Schuetz, M. , Hoffmann, N. et al (2018). Distribution, Mobility and Anchoring of Lignin‐Related Oxidative Enzymes in Arabidopsis Secondary Cell Walls. Journal of Experimental Botany, 69, 1849–1859. 29481639 10.1093/jxb/ery067 PMC6018803

[pld3265-bib-0015] Coleman, H. D. , Park, J. Y. , Nair, R. , Chapple, C. , & Mansfield, S. D. (2008a). RNAi‐mediated suppression of p‐coumaroyl‐CoA 3′‐hydroxylase in hybrid poplar impacts lignin deposition and soluble secondary metabolism. Proceedings of the National Academy of Sciences of the United States of America, 105, 4501–4506. 18316744 10.1073/pnas.0706537105 PMC2393750

[pld3265-bib-0016] Coleman, H. D. , Samuels, A. L. , Guy, R. D. , & Mansfield, S. D. (2008b). Perturbed lignification impacts tree growth in hybrid poplar ‐ A function of sink strength, vascular integrity, and photosynthetic assimilation. Plant Physiology, 148, 1229–1237. 18805953 10.1104/pp.108.125500 PMC2577275

[pld3265-bib-0017] Cullis, I. F. , Saddler, J. N. , & Mansfield, S. D. (2004). Effect of initial moisture content and chip size on the bioconversion efficiency of softwood lignocellulosics. Biotechnology and Bioengineering, 85, 413–421. 14755559 10.1002/bit.10905

[pld3265-bib-0018] De Meester, B. , de Vries, L. , Özparpucu, M. et al (2018). Vessel‐specific reintroduction of CINNAMOYL‐COA REDUCTASE1 (CCR1) in Dwarfed ccr1 mutants restores vessel and xylary fiber integrity and increases biomass. Plant Physiology, 176, 611–633. 29158331 10.1104/pp.17.01462 PMC5761799

[pld3265-bib-0019] Dima, O. , Ralph, J. , Vanholme, B. , Boerjan, W. , Kim, H. , & Morreel, K. (2015). Small glycosylated lignin oligomers are stored in arabidopsis leaf vacuoles. The Plant Cell, 27, 695–710. 10.1105/tpc.114.134643 25700483 PMC4558659

[pld3265-bib-0020] Dixon, R. A. , & Barros, J. (2019). Lignin biosynthesis: Old roads revisited and new roads explored. Open Biology, 9, 190215 10.1098/rsob.190215 31795915 PMC6936255

[pld3265-bib-0021] Du, Z. , Zhou, X. , Ling, Y. , Zhang, Z. , & Su, Z. (2010). agriGO: A GO analysis toolkit for the agricultural community. Nucleic Acids Research, 38, W64–W70. 10.1093/nar/gkq310 20435677 PMC2896167

[pld3265-bib-0022] Fraser, C. M. , & Chapple, C. (2011). The phenylpropanoid pathway in Arabidopsis. The Arabidopsis Book, 9, e0152 10.1199/tab.0152 22303276 PMC3268504

[pld3265-bib-0023] Gallego‐Giraldo, L. , Liu, C. , Pose‐Albacete, S. et al (2020). ARABIDOPSIS DEHISCENCE ZONE POLYGALACTURONASE 1 (ADPG1) releases latent defense signals in stems with reduced lignin content. Proceedings of the National Academy of Sciences of the United States of America, 117, 3281–3290. 31974310 10.1073/pnas.1914422117 PMC7022211

[pld3265-bib-0024] Guénin, S. , Mauriat, M. , Pelloux, J. , Wuytswinkel, O. V. , Bellini, C. , & Gutierrez, L. (2009). Normalization of qRT‐PCR data: The necessity of adopting a systematic, experimental conditions‐specific, validation of references. Journal of Experimental Botany, 60, 487–493. 10.1093/jxb/ern305 19264760

[pld3265-bib-0025] Hoffmann, L. , Besseau, S. , Geoffroy, P. , Ritzenthaler, C. , Meyer, D. , Lapierre, C. , … Legrand, M. (2005). Acyltransferase‐catalysed p‐coumarate ester formation is a committed step of lignin biosynthesis. Plant Biosystems, 139, 50–53.

[pld3265-bib-0026] Jaini, R. , Wang, P. , Dudareva, N. , Chapple, C. , & Morgan, J. A. (2017). Targeted metabolomics of the phenylpropanoid pathway in arabidopsis thaliana using reversed phase liquid chromatography coupled with tandem mass spectrometry. Phytochemical Analysis, 28, 267–276. 28146307 10.1002/pca.2672

[pld3265-bib-0027] Kaneda, M. , Schuetz, M. , Lin, B. S. P. , Chanis, C. , Hamberger, B. , Western, T. L. , … Samuels, A. L. (2011). ABC transporters coordinately expressed during lignification of Arabidopsis stems include a set of ABCBs associated with auxin transport. Journal of Experimental Botany, 62, 2063–2077. 21239383 10.1093/jxb/erq416 PMC3060696

[pld3265-bib-0028] Karimi, M. , Inzé, D. , & Depicker, A. (2002). GATEWAY^TM^ vectors for Agrobacterium‐mediated plant transformation. Trends in Plant Science, 7, 193–195. 11992820 10.1016/s1360-1385(02)02251-3

[pld3265-bib-0029] Koressaar, T. , & Remm, M. (2007). Enhancements and modifications of primer design program Primer3. Bioinformatics, 23, 1289–1291. 10.1093/bioinformatics/btm091 17379693

[pld3265-bib-0030] Langmead, B. , & Salzberg, S. L. (2012). Fast gapped‐read alignment with Bowtie 2. Nature Methods, 9, 357–359. 22388286 10.1038/nmeth.1923 PMC3322381

[pld3265-bib-0031] Lanot, A. , Hodge, D. , Jackson, R. G. , George, G. L. , Elias, L. , Lim, E. K. , … Bowles, D. J. (2006). The glucosyltransferase UGT72E2 is responsible for monolignol 4‐O‐glucoside production in Arabidopsis thaliana. The Plant Journal, 48, 286–295. 16995900 10.1111/j.1365-313X.2006.02872.x

[pld3265-bib-0032] LeRoy, J. , Huss, B. , Creach, A. , Hawkins, S. , & Neutelings, G. (2016). Glycosylation Is a major regulator of phenylpropanoid availability and biological activity in plants. Frontiers in Plant Science, 7, 735. 27303427 10.3389/fpls.2016.00735 PMC4880792

[pld3265-bib-0033] Li, X. , Bonawitz, N. D. , Weng, J.‐K.‐J.‐K. , & Chapple, C. (2010). The growth reduction associated with repressed lignin biosynthesis in arabidopsis thaliana is independent of flavonoids. The Plant Cell, 22, 1620–1632. 20511296 10.1105/tpc.110.074161 PMC2899864

[pld3265-bib-0034] Lin, J. S. , Huang, X. X. , Li, Q. , Cao, Y. , Bao, Y. , Meng, X. F. , … Hou, B. K. (2016). UDP‐glycosyltransferase 72B1 catalyzes the glucose conjugation of monolignols and is essential for the normal cell wall lignification in Arabidopsis thaliana. The Plant Journal, 88, 26–42. 27273756 10.1111/tpj.13229

[pld3265-bib-0035] Liu, Q. , Luo, L. , & Zheng, L. (2018). Lignins: Biosynthesis and Biological Functions in Plants. International Journal of Molecular Sciences, 19, 335. 10.3390/ijms19020335 PMC5855557 29364145

[pld3265-bib-0036] Love, M. I. , Huber, W. , Anders, S. et al (2014). Moderated estimation of fold change and dispersion for RNA‐seq data with DESeq2. Genome Biology, 15, 550. 25516281 10.1186/s13059-014-0550-8 PMC4302049

[pld3265-bib-0037] Lynn, D. G. , Chen, R. H. , Manning, K. S. , & Wood, H. N. (1987). The structural characterization of endogenous factors from Vinca rosea crown gall tumors that promote cell division of tobacco cells. Proceedings of the National Academy of Sciences of the United States of America, 84, 615–619. 3468501 10.1073/pnas.84.3.615 PMC304264

[pld3265-bib-0038] Mahon, E. L. , & Mansfield, S. D. (2019). Tailor‐made trees: Engineering lignin for ease of processing and tomorrow’s bioeconomy. Current Opinion in Biotechnology, 56, 147–155. 30529238 10.1016/j.copbio.2018.10.014

[pld3265-bib-0039] Mehrtens, F. , Kranz, H. , Bednarek, P. , & Weisshaar, B. (2005). The Arabidopsis transcription factor MYB12 is a flavonol‐specific regulator of phenylpropanoid biosynthesis. Plant Physiology, 138, 1083–1096. 15923334 10.1104/pp.104.058032 PMC1150422

[pld3265-bib-0040] Miao, Y.‐C. , & Liu, C.‐J. (2010). ATP‐binding cassette‐like transporters are involved in the transport of lignin precursors across plasma and vacuolar membranes. Proceedings of the National Academy of Sciences of the United States of America, 107, 22728–22733. 21149736 10.1073/pnas.1007747108 PMC3012465

[pld3265-bib-0041] Mitra, P. P. , & Loqué, D. (2014). Histochemical staining of arabidopsis thaliana secondary cell wall elements. Journal of Visualized Experiments: Jove, 1–11. 10.3791/51381 PMC4186213 24894795

[pld3265-bib-0042] Mottiar, Y. , Vanholme, R. , Boerjan, W. , Ralph, J. , & Mansfield, S. D. (2016). Designer lignins: Harnessing the plasticity of lignification. Current Opinion in Biotechnology, 37, 190–200. 26775114 10.1016/j.copbio.2015.10.009

[pld3265-bib-0043] Muro‐Villanueva, F. , Mao, X. , & Chapple, C. (2019). Linking phenylpropanoid metabolism, lignin deposition, and plant growth inhibition. Current Opinion in Biotechnology, 56, 202–208. 30677701 10.1016/j.copbio.2018.12.008

[pld3265-bib-0044] O’Malley, R. C. , Huang, S. C. , Song, L. , Lewsey, M. G. , Bartlett, A. , Nery, J. R. , … Ecker, J. R. (2016). Cistrome and Epicistrome features shape the regulatory DNA landscape. Cell, 166, 1598 10.1016/j.cell.2016.08.063 27610578

[pld3265-bib-0045] Ohtani, M. , & Demura, T. (2019). The quest for transcriptional hubs of lignin biosynthesis: Beyond the NAC‐MYB‐gene regulatory network model. Current Opinion in Biotechnology, 56, 82–87. 30390602 10.1016/j.copbio.2018.10.002

[pld3265-bib-0046] Panda, C. , Li, X. , Wager, A. , Chen, H. , & Li, X. U. (2020). An importin‐beta‐like protein mediates lignin‐modification‐induced dwarfism in Arabidopsis. The Plant Journal, 102(6), 1281–1293. 10.1111/tpj.14701 31972869

[pld3265-bib-0047] Perkins, M. , Smith, R. A. , & Samuels, L. (2019). The transport of monomers during lignification in plants: Anything goes but how? Current Opinion in Biotechnology, 56, 69–74. 30347315 10.1016/j.copbio.2018.09.011

[pld3265-bib-0048] Ralph, J. , Lapierre, C. , & Boerjan, W. (2019). Lignin structure and its engineering. Current Opinion in Biotechnology, 56, 240–249. 30921563 10.1016/j.copbio.2019.02.019

[pld3265-bib-0049] Schmittgen, T. D. , & Livak, K. J. (2008). Analyzing real‐time PCR data by the comparative CT method. Nature Protocols, 3, 1101–1108. 18546601 10.1038/nprot.2008.73

[pld3265-bib-0050] Smith, R. A. , Schuetz, M. , Roach, M. , Mansfield, S. D. , Ellis, B. , & Samuels, L. (2013). Neighboring parenchyma cells contribute to arabidopsis xylem lignification, while lignification of interfascicular fibers is cell autonomous. The Plant Cell, 25, 3988–3999. 24096341 10.1105/tpc.113.117176 PMC3877792

[pld3265-bib-0051] Swarbreck, D. , Wilks, C. , Lamesch, P. et al (2008). The Arabidopsis Information Resource (TAIR): Gene structure and function annotation. Nucleic Acids Research, 36, D1009–D1014. 17986450 10.1093/nar/gkm965 PMC2238962

[pld3265-bib-0052] Takeuchi, M. , Kegasa, T. , Watanabe, A. , Tamura, M. , & Tsutsumi, Y. (2018). Expression analysis of transporter genes for screening candidate monolignol transporters using Arabidopsis thaliana cell suspensions during tracheary element differentiation. Journal of Plant Research, 131, 297–305. 10.1007/s10265-017-0979-4 28921082

[pld3265-bib-0053] Tsuyama, T. , Kawai, R. , Shitan, N. , Matoh, T. , Sugiyama, J. , Yoshinaga, A. , … Yazaki, K. (2013). Proton‐dependent coniferin transport, a common major transport event in differentiating xylem tissue of woody plants. Plant Physiology, 162, 918–926. 23585651 10.1104/pp.113.214957 PMC3668080

[pld3265-bib-0054] Umezawa, T. (2018). Lignin modification in planta for valorization. Phytochemistry Reviews, 17, 1305–1327.

[pld3265-bib-0055] Väisänen, E. E. , Smeds, A. I. , Fagerstedt, K. V. , Teeri, T. H. , Willför, S. M. , & Kärkönen, A. (2015). Coniferyl alcohol hinders the growth of tobacco BY‐2 cells and Nicotiana benthamiana seedlings. Planta, 242, 747–760. 10.1007/s00425-015-2348-7 26108783

[pld3265-bib-0056] Van‐Acker, R. , Vanholme, R. , Storme, V. , Mortimer, J. C. , Dupree, P. , & Boerjan, W. (2013). Lignin biosynthesis perturbations affect secondary cell wall composition and saccharification yield in Arabidopsis thaliana. Biotechnology for Biofuels, 6, 46 10.1186/1754-6834-6-46 23622268 PMC3661393

[pld3265-bib-0057] Vanholme, R. , De Meester, B. , Ralph, J. , & Boerjan, W. (2019). Lignin biosynthesis and its integration into metabolism. Current Opinion in Biotechnology, 56, 230–239. 30913460 10.1016/j.copbio.2019.02.018

[pld3265-bib-0058] Vanholme, R. , Storme, V. , Vanholme, B. et al (2012). A systems biology view of responses to lignin biosynthesis perturbations in arabidopsis. The Plant Cell, 24, 3506–3529. 23012438 10.1105/tpc.112.102574 PMC3480285

[pld3265-bib-0059] Vermaas, J. V. , Dixon, R. A. , Chen, F. , Mansfield, S. D. , Boerjan, W. , Ralph, J. , … Beckham, G. T. (2019). Passive membrane transport of lignin‐related compounds. Proceedings of the National Academy of Sciences of the United States of America, 116, 23117–23123. 31659054 10.1073/pnas.1904643116 PMC6859372

[pld3265-bib-0060] Xue, J. , Luo, D. , Xu, D. , Zeng, M. , Cui, X. , Li, L. , & Huang, H. (2015). CCR1, an enzyme required for lignin biosynthesis in Arabidopsis, mediates cell proliferation exit for leaf development. The Plant Journal, 83, 375–387. 26058952 10.1111/tpj.12902

[pld3265-bib-0061] Yamaguchi, M. , Goué, N. , Igarashi, H. , Ohtani, M. , Nakano, Y. , Mortimer, J. C. , … Demura, T. (2010). VASCULAR‐RELATED NAC‐DOMAIN6 and VASCULAR‐RELATED NAC‐DOMAIN7 effectively induce transdifferentiation into xylem vessel elements under control of an induction system. Plant Physiology, 153, 906–914. 10.1104/pp.110.154013 20488898 PMC2899931

[pld3265-bib-0062] Yang, F. , Mitra, P. , Zhang, L. et al (2013). Engineering secondary cell wall deposition in plants. Plant Biotechnology Journal, 11, 325–335. 23140549 10.1111/pbi.12016 PMC3644865

[pld3265-bib-0063] Zhao, Q. , Nakashima, J. , Chen, F. et al (2013). LACCASE Is Necessary and Nonredundant with PEROXIDASE for Lignin Polymerization during Vascular Development in Arabidopsis. The Plant Cell, 25, 3976–3987. 24143805 10.1105/tpc.113.117770 PMC3877815

[pld3265-bib-0064] Zhou, J. , Lee, C. , Zhong, R. , & Ye, Z.‐H. (2009). MYB58 and MYB63 are transcriptional activators of the lignin biosynthetic pathway during secondary cell wall formation in arabidopsis. Plant Cell Online, 21, 248–266. 10.1105/tpc.108.063321 PMC2648072 19122102

