# Supplementary material for: Dwarfism of high‐monolignol Arabidopsis plants is rescued by ectopic LACCASE overexpression
Source: Plant Direct. 2020 Sep 28;4(9):e00265. doi: 10.1002/pld3.265 (PMC7520647; doi:10.1002/pld3.265)
Supplement: Supplementary file 1 — Fig S1‐S8 [file PLD3-4-e00265-s001.pdf]

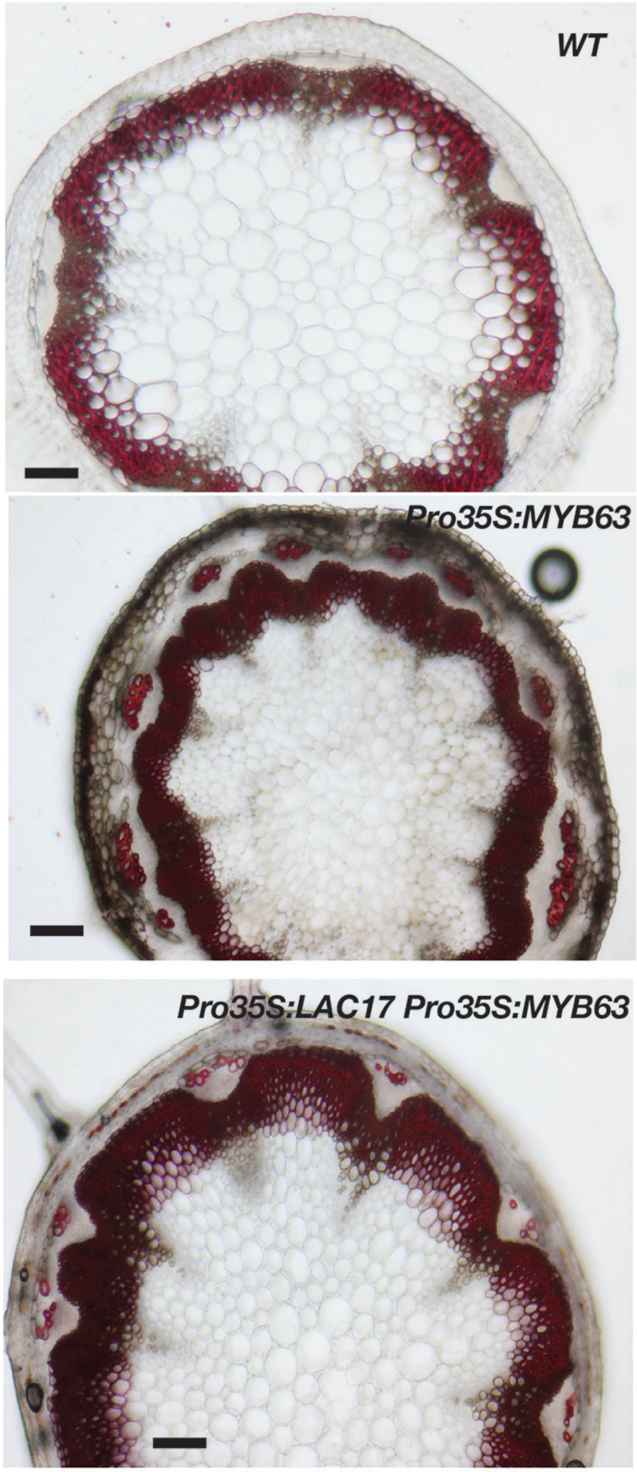

Supplemental Figure 1: Mäule staining of lignin in inflorescence stems of Arabidopsis. WT dwarf *MYB63-OX* and rescued *MYB63-OX/LAC17-OX lines.* bar = 60 μm. A Leica DMR epifluorescence microscope was used to capture images of hand sectioned Arabidopsis stems that were Mäule stained as per (Mitra and Loqué, 2014).

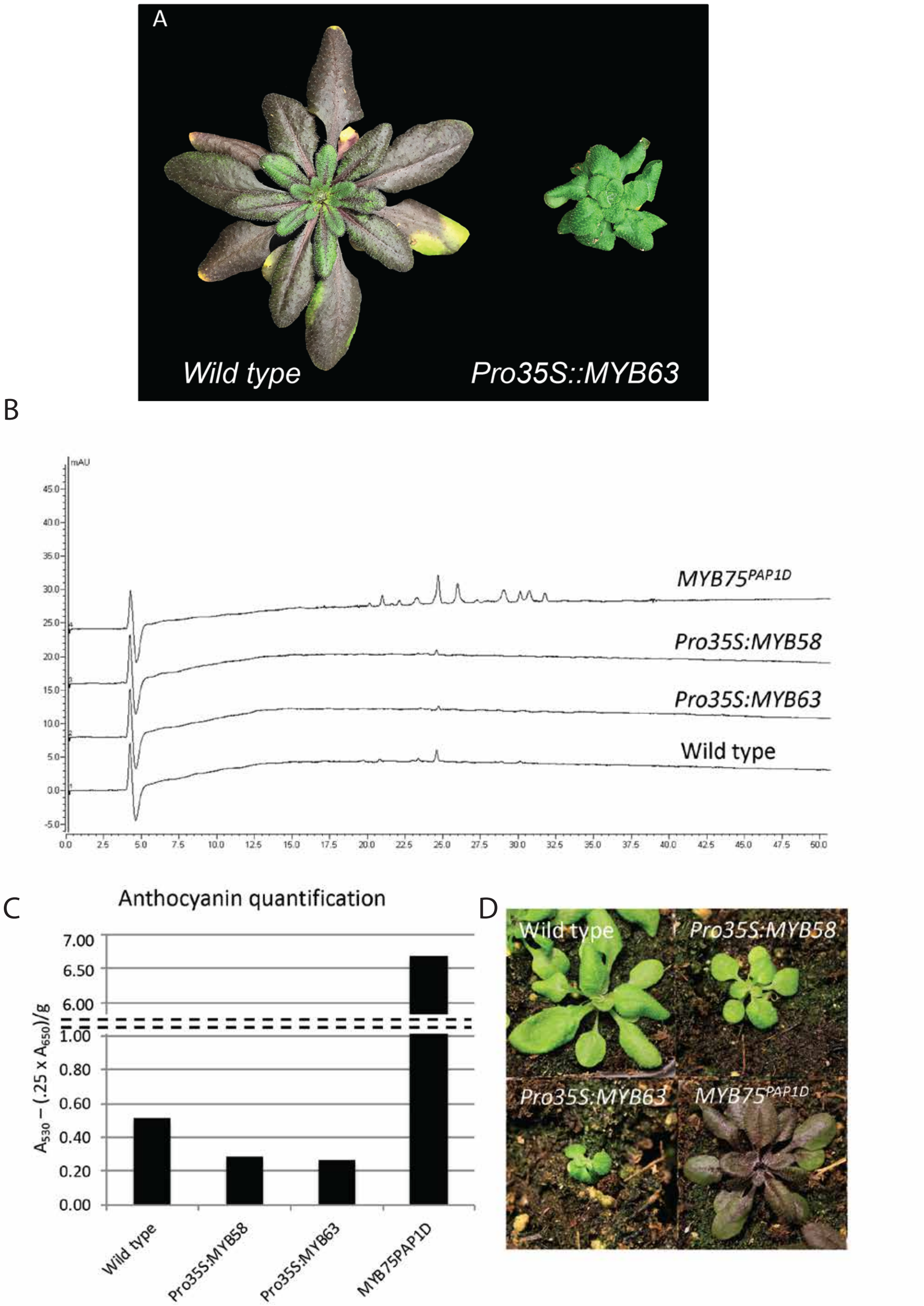

Supplemental Figure 2: MYB58 and MYB63 overexpression lines lack anthocyanins induced by light stress. (A) Wild-type Arabidopsis plants grown in high light (210 µmol m^{-2} s^{-1}) accumulate purple anthocyanin pigments in their leaves while *Pro35S::MYB63* plants lack anthocyanins under identical conditions. (B) HPLC chromatograph of methanol leaf extracts at 510 nm indicating the presence of several anthocyanin peaks in *MYB75^{PAP1D}* extracts which are not observed in *Pro35S::MYB63*, *Pro35S::MYB58* or wild type. (C) Spectrophotometric quantification of anthocyanin content, based on absorbance at 530nm of methanol extracts from the indicated genotypes, calculated as $Q_{anthocyanins}$ = A530 –(.25 x A650) x $mass^{-1}$. (D) Images of four-week-old plants that show an obvious accumulation of purple anthocyanins in *MYB75^{PAP1D}* plants compared to wild type, *Pro35S::MYB58* and *Pro35S::MYB63* plants.

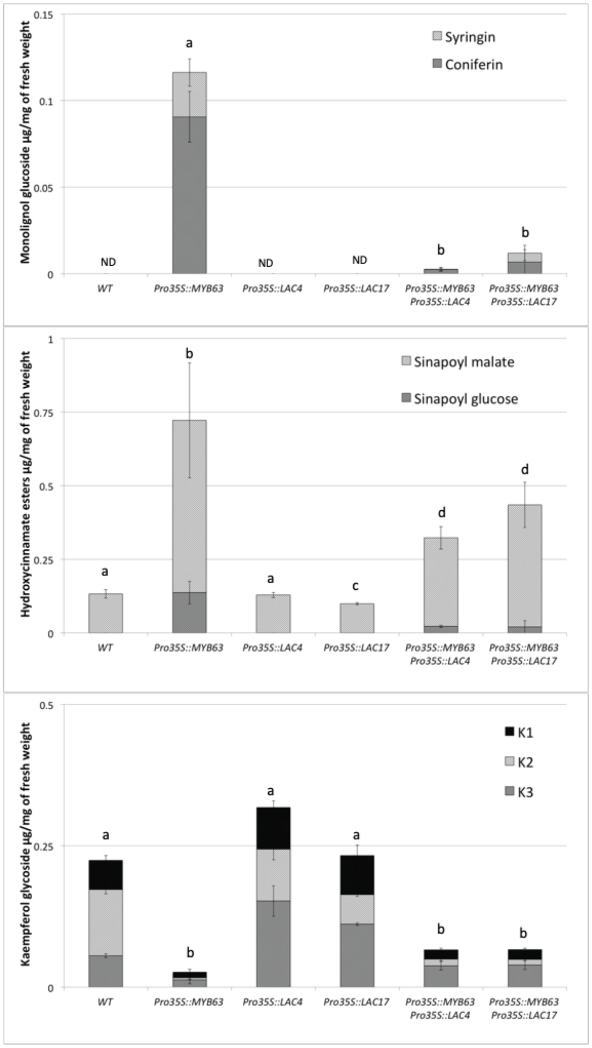

Supplemental Figure 3: Both LAC4 and LAC17 overexpression alter the soluble phenolics in *MYB63-OX* lines. Quantification of the soluble phenolics from Arabidopsis leaves of *Pro35S::MYB63* (*MYB63-OX*) overexpression lines, as well as *MYB63-OX* lines that co-overexpress either *Pro35S::LAC4* (*LAC4-OX*) or *Pro35S::LAC17* (*LAC17-OX*). (A) monolignol glucosides, coniferin and syringin; (B) hydroxycinnamate esters (HCE), sinapoyl glucose and sinapoyl malate; and (C) kaempferol glycosides. Bars indicate standard deviation. n = 3-6 pooled samples of 5 leaves from 5 plants. Means with different letters represent statistically significant differences of total monolignol glucosides, HCEs or flavonols (Tukey's pairwise comparison, P < 0.01).

A

B

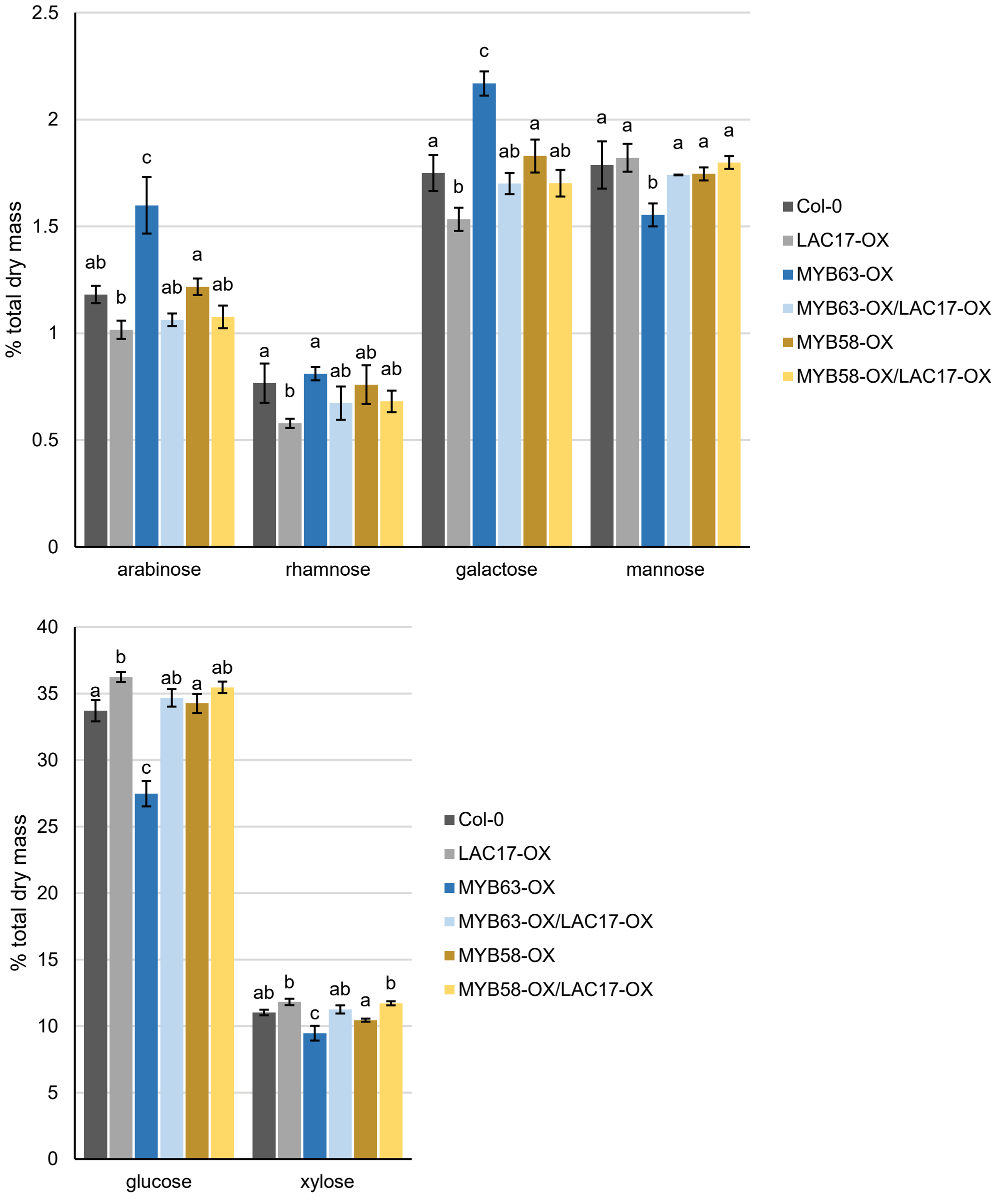

Supplemental Figure 4: Cell wall carbohydrate content of *LAC17-OX, MYB63-OX, MYB63-OX/LAC17-OX, MYB58-OX* and *MYB58-OX/LAC17-OX* compared to *Col-0* control expressed as a percentage of total stem dry mass. (A) Levels of arabinose, rhamnose, galactose and mannose. (B) Levels of glucose and xylose. Bars indicate standard deviation, n=3, letters indicate statistically significant differences (Tukey's pairwise comparison, $P < 0.05$).

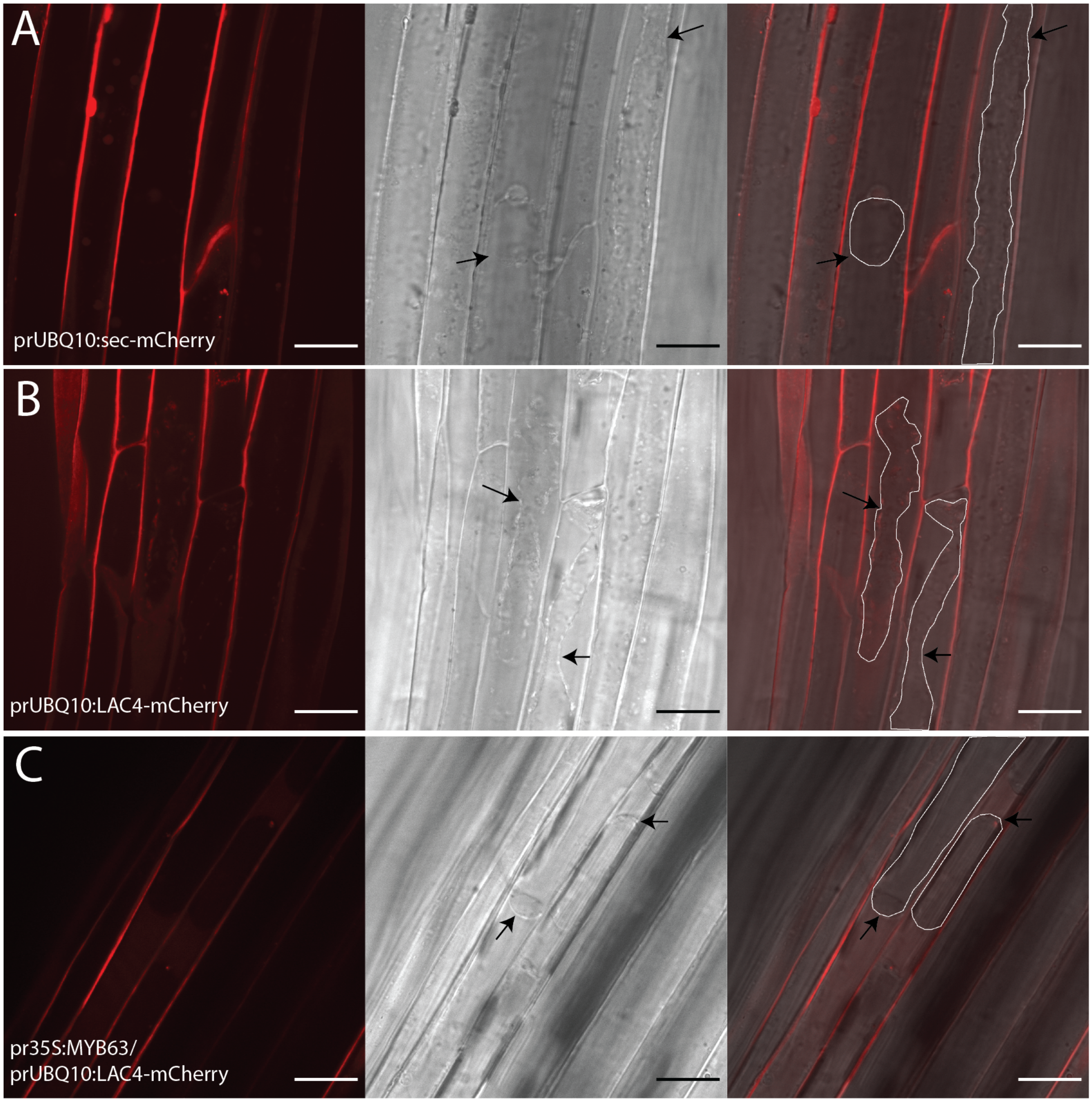

Supplemental Figure 5: Confirmation of cell wall localization of LAC4-mCherry by plasmolysis. 7 day old etiolated cotyledons were plasmolyzed in 0.4M mannitol for 1 hour. LAC4-mCherry tagged protein in red followed by brightfield image and merged image. Plasmolyzed plama membrane indicated by black arrows and outlined in white in merged image. A) A cell wall-secreted control mCherry protein localizes to the cell wall in *prUBQ10-sec-mCherry* lines (Chou *et al.*, 2018). B) Wall label *in ProUBQ10::LAC4-mCherry*. C) Wall label in *Pro35S::MYB63/prUBQ10:LAC4-mCherry*. Scale bar is 20 µm.

A. *35S:MYB63* in *tt4* mutant (deficient in chalcone synthase).

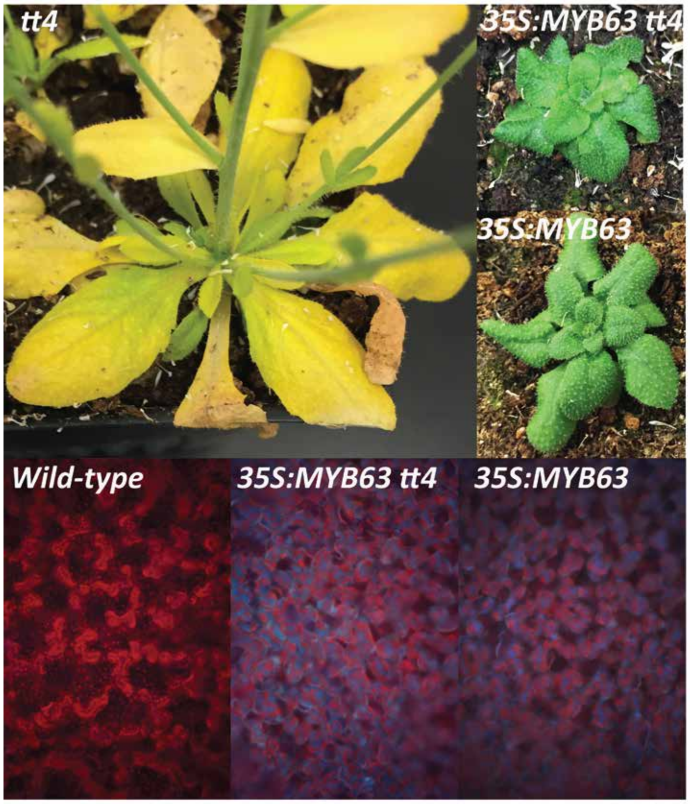

B. *35S:MYB63* in *med5a 5b* mutant.

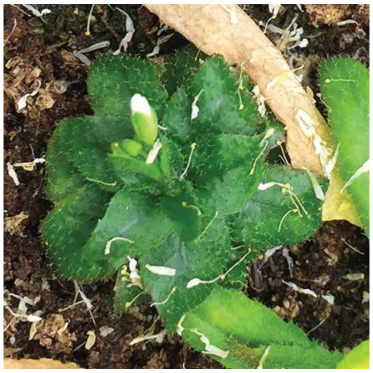

C. *35S:MYB63* in *lac4 lac17* mutant.

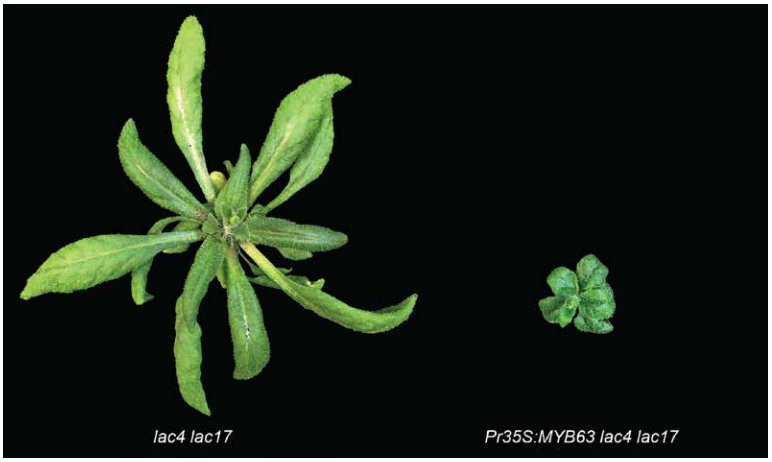

Supplemental Figure 6: Reduced growth in MYB63-OX lines is not dependent on flavonoids (chalcone synthase), Mediator 5a 5b, or LACCASE4 LACCASE17. (A) Chalcone synthase knockout mutant *tt4-2* (Burbulis et al., 1996) lacks anthocyanin pigments but has wild-type plant biomass phenotype. When *tt4-2* was transformed with *Pro35S::MYB63*, the growth of the *Pro35S::MYB63 tt4-2* plants is similar to *Pro35S::MYB63* in Col-0. Autofluorescence of wild-type leaves shows red autofluorescence from chlorophyll, while the *Pro35S::MYB63 tt4-2* and *Pro35S::MYB63* show blue autofluorescence from vacuolar phenolics. 6 week old plants. (B) Primary transformants of *Pro35S::MYB63* in the *mediator5a mediator5b* (*med5a/5b*) background. While *med5a/5b* mutants had wild-type growth (Bonawitz et al., 2014), when transformed with *Pro35S::MYB63,* they resemble *Pro35S::MYB63* in wild type. 6 week old plants. (C) Primary transformants of *Pro35S::MYB63* in the *laccase4 laccase17* (*lac4 lac17*) double mutant background. While *lac4 lac17* mutants had growth similar to wild type, *Pro35S::MYB63* in *lac4 lac17* background resemble *Pro35S::MYB63* in Col-0. 4 week old plants.

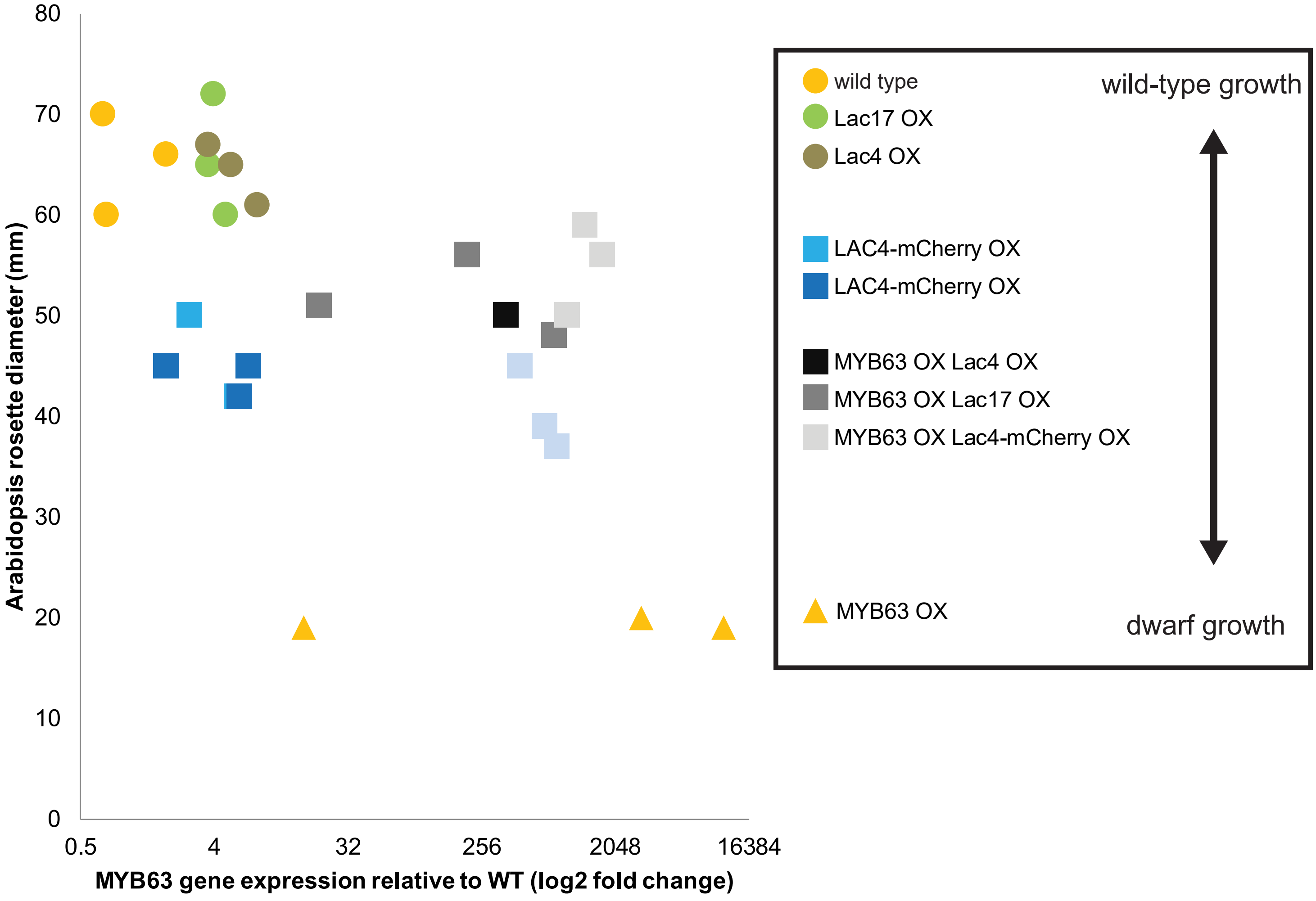

Supplemental Figure 7: Comparison of *MYB63* gene expression and plant size. The log2 fold expression as determined by real time quantitative PCR plotted against the rosette diameter at ~ 3 weeks in age of *MYB63-OX, LAC17-OX, LAC4-OX, LAC4-mCherry-OX, MYB63-OX/LAC4-OX, MYB63-OX/LAC17-OX,* and *MYB63-OX/LAC4-mCherry-OX.*

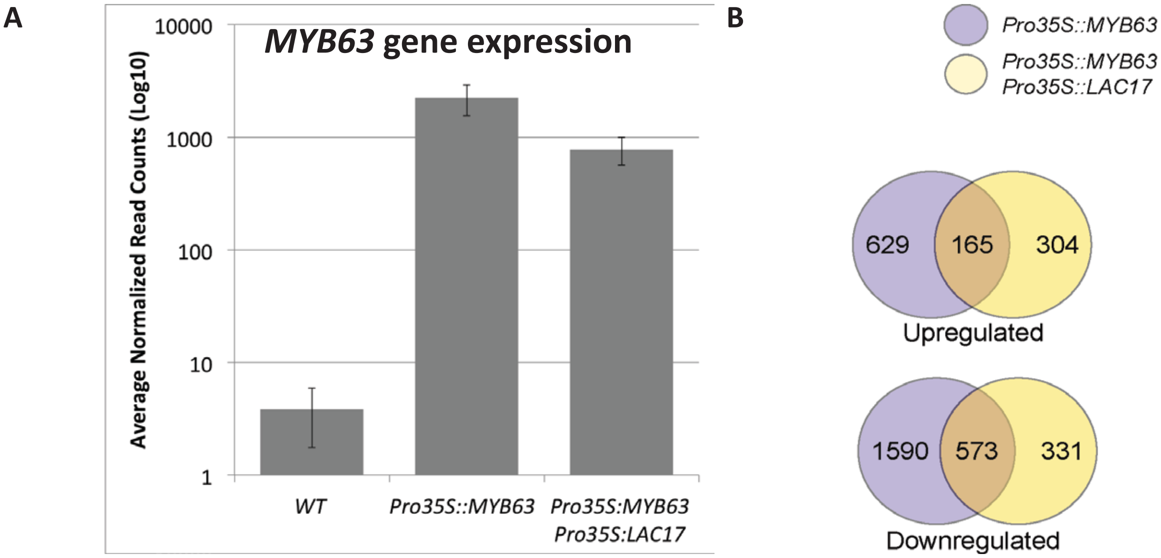

Supplemental Figure 8: (A) MYB63 gene expression in wild type compared to single *MYB63-OX* and double *MYB63-OX LAC17-OX*. Bars indicate standard deviation, n = 3 (B) Venn diagram of the number of differentially expressed genes that upregulated or downregulated in dwarf *MYB63-OX* (purple) or the rescued *MYB63-OX*/*LAC17-OX* (yellow) lines.
