# Supplementary material for: Dwarfism of high‐monolignol Arabidopsis plants is rescued by ectopic LACCASE overexpression
Source: Plant Direct. 2020 Sep 28;4(9):e00265. doi: 10.1002/pld3.265 (PMC7520647; doi:10.1002/pld3.265)
Supplement: Supplementary file 2 — Table S1‐S4 [file PLD3-4-e00265-s002.pdf]

Supplemental Table 1-RNA seq data comparing Pro35S::MYB63 with wild type.

DAP.MYB63.target(1.5kb upstream TSS)	Gene	baseMean	log2FoldChange	lfcMLE	lfcSE	stat	pvalue	padj (FDR)	TAIR10 Symbol	TAIR10 Annotation (Short)
YES	AT1G79180	1057	8.00	9.15	0.67	11.86	0.00000	0.00000	ATMYB63	MYB DOMAIN PROTEIN 63
	AT3G05727	224	7.79	13.44	0.87	9.00	0.00000	0.00000	NA	NA
	AT4G22513	96	6.85	12.22	0.89	7.67	0.00000	0.00000	NA	NA
	AT5G66690	77	6.62	11.89	0.90	7.37	0.00000	0.00000	UGT72E2	0
	AT4G30140	117	6.60	9.32	0.86	7.67	0.00000	0.00000	CDEF1	CUTICLE DESTRUCTING FACTOR 1
	AT4G22517	75	6.38	11.87	0.92	6.93	0.00000	0.00000	NA	NA
YES	AT1G62333	45	6.19	11.12	0.90	6.88	0.00000	0.00000	NA	NA
YES	AT1G52760	3050	6.16	6.56	0.51	12.01	0.00000	0.00000	LysoPL2	lysophospholipase 2
	AT5G64110	38	5.80	10.88	0.93	6.24	0.00000	0.00000	NA	NA
	AT1G11080	55	5.67	7.24	0.81	7.00	0.00000	0.00000	scpl31	serine carboxypeptidase-like 31
	AT3G32030	27	5.52	10.41	0.94	5.90	0.00000	0.00000	NA	NA
YES	AT2G37040	6734	5.41	5.74	0.50	10.83	0.00000	0.00000	ATPAL1	0
	AT5G66985	97	5.32	6.41	0.77	6.90	0.00000	0.00000	NA	NA
	AT3G13960	32	5.24	7.45	0.88	5.92	0.00000	0.00000	AtGRF5	growth-regulating factor 5
	AT4G22510	31	5.19	10.57	0.98	5.32	0.00000	0.00001	NA	NA
YES	AT2G45290	357	5.13	5.64	0.61	8.39	0.00000	0.00000	TKL2	transketolase 2
YES	AT3G21240	830	5.12	5.58	0.59	8.74	0.00000	0.00000	4CL2	4-coumarate:CoA ligase 2
	AT1G13710	29	5.10	7.28	0.89	5.75	0.00000	0.00000	CYP78A5	cytochrome P450, family 78, subfamily A, polypeptide 5
YES	AT5G12330	20	4.91	9.92	0.98	5.04	0.00000	0.00003	LRP1	LATERAL ROOT PRIMORDIUM 1
YES	AT3G10340	619	4.89	5.33	0.59	8.28	0.00000	0.00000	PAL4	phenylalanine ammonia-lyase 4
	AT2G44480	386	4.86	5.35	0.61	7.96	0.00000	0.00000	BGLU17	beta glucosidase 17
	AT1G04660	274	4.85	5.43	0.66	7.40	0.00000	0.00000	NA	NA
	AT1G43800	44	4.81	6.26	0.85	5.66	0.00000	0.00000	FTM1	FLORAL TRANSITION AT THE MERISTEM1
	AT4G36430	155	4.78	5.45	0.69	6.93	0.00000	0.00000	NA	NA
	AT2G38390	15	4.68	9.55	0.98	4.77	0.00000	0.00009	NA	NA
	AT1G10480	16	4.66	9.61	0.99	4.72	0.00000	0.00010	ZFP5	zinc finger protein 5
YES	AT5G40760	743	4.64	4.98	0.54	8.60	0.00000	0.00000	G6PD6	glucose-6-phosphate dehydrogenase 6
	AT4G22490	186	4.51	4.91	0.58	7.72	0.00000	0.00000	NA	NA
	AT5G66940	14	4.47	9.45	1.00	4.48	0.00001	0.00025	NA	NA
YES	AT5G24760	705	4.44	4.89	0.62	7.18	0.00000	0.00000	NA	NA
	AT4G21760	147	4.43	5.17	0.74	5.96	0.00000	0.00000	BGLU47	beta-glucosidase 47
	AT4G22520	35	4.39	5.92	0.89	4.92	0.00000	0.00004	NA	NA
	AT5G58860	13	4.31	9.30	1.01	4.28	0.00002	0.00053	CYP86	0
	AT5G57250	29	4.29	5.72	0.89	4.84	0.00000	0.00006	NA	NA
YES	AT1G06550	664	4.28	4.61	0.55	7.76	0.00000	0.00000	NA	NA
	AT2G30480	13	4.22	9.35	1.02	4.15	0.00003	0.00080	NA	NA
	AT1G78815	12	4.19	9.19	1.01	4.13	0.00004	0.00085	LSH7	LIGHT SENSITIVE HYPOCOTYLS 7
	AT4G14250	37	4.16	5.07	0.80	5.22	0.00000	0.00001	NA	NA
YES	AT1G09420	70	4.15	4.70	0.68	6.13	0.00000	0.00000	G6PD4	glucose-6-phosphate dehydrogenase 4
YES	AT3G53260	6784	4.12	4.43	0.55	7.51	0.00000	0.00000	ATPAL2	0
YES	AT1G61820	33	4.10	5.16	0.83	4.93	0.00000	0.00004	BGLU46	beta glucosidase 46
	AT3G58590	22	4.09	5.87	0.93	4.39	0.00001	0.00036	NA	NA
	AT4G22485	10	4.06	8.99	1.02	3.99	0.00007	0.00136	NA	NA
YES	AT2G40890	3165	4.05	4.40	0.58	7.04	0.00000	0.00000	CYP98A3	cytochrome P450, family 98, subfamily A, polypeptide 3
	AT4G21770	122	4.04	4.70	0.73	5.52	0.00000	0.00000	NA	NA
	AT3G59930	409	4.02	4.40	0.60	6.68	0.00000	0.00000	NA	NA
	AT3G63450	26	3.98	5.09	0.85	4.68	0.00000	0.00012	NA	NA
	AT4G38080	40	3.90	5.12	0.89	4.40	0.00001	0.00034	NA	NA
YES	AT2G36880	5793	3.85	4.07	0.48	8.03	0.00000	0.00000	MAT3	methionine adenosyltransferase 3
	AT1G61050	10	3.85	8.91	1.03	3.73	0.00019	0.00303	NA	NA
	AT1G17345	20	3.85	5.76	0.96	3.99	0.00007	0.00134	SAUR77	SMALL AUXIN UPREGULATED RNA 77
	AT1G74030	337	3.82	4.12	0.56	6.88	0.00000	0.00000	ENO1	enolase 1
YES	AT1G13420	16	3.80	5.40	0.93	4.09	0.00004	0.00098	ATST4B	ARABIDOPSIS THALIANA SULFOTRANSFERASE 4B
	AT2G24700	14	3.78	6.30	1.00	3.79	0.00015	0.00256	NA	NA
	AT4G14465	49	3.76	4.60	0.81	4.64	0.00000	0.00014	AHL20	AT-hook motif nuclear-localized protein 20
YES	AT1G48850	2377	3.75	3.98	0.50	7.55	0.00000	0.00000	EMB1144	embryo defective 1144
	AT1G13600	18	3.74	5.07	0.91	4.13	0.00004	0.00085	AtbZIP58	basic leucine-zipper 58
	AT1G03780	18	3.70	5.56	0.97	3.82	0.00013	0.00232	AtTPX2	0
	AT1G19540	127	3.70	4.07	0.61	6.01	0.00000	0.00000	NA	NA
YES	AT4G34050	28972	3.68	3.93	0.52	7.03	0.00000	0.00000	CCoAOMT1	caffeoyl coenzyme A O-methyltransferase 1
YES	AT3G29200	1697	3.67	3.93	0.53	6.87	0.00000	0.00000	ATCM1	ARABIDOPSIS THALIANA CHORISMATE MUTASE 1
	AT2G20980	22	3.66	4.84	0.89	4.13	0.00004	0.00086	MCM10	minichromosome maintenance 10

Supplemental Table 1-RNA seq data comparing Pro35S::MYB63 with wild type.

DAP.MYB63.target(1.5kb upstream TSS)	Gene	baseMean	log2FoldChange	lfcMLE	lfcSE	stat	pvalue	padj (FDR)	TAIR10 Symbol	TAIR10 Annotation (Short)
YES	AT2G45300	1093	3.66	3.90	0.52	7.07	0.00000	0.00000	NA	NA
	AT4G36610	17	3.66	5.43	0.96	3.81	0.00014	0.00237	NA	NA
	AT1G64470	21	3.63	4.82	0.89	4.08	0.00005	0.00103	NA	NA
	AT4G08150	14	3.63	5.23	0.94	3.86	0.00011	0.00206	BP	BREVIPEDICELLUS
	AT5G11780	16	3.61	5.44	0.97	3.73	0.00019	0.00302	NA	NA
	AT2G47950	22	3.61	4.53	0.84	4.32	0.00002	0.00047	NA	NA
YES	AT4G25810	925	3.58	3.89	0.58	6.17	0.00000	0.00000	XTH23	xyloglucan endotransglucosylase/hydrolase 23
	AT4G09500	340	3.57	3.82	0.53	6.80	0.00000	0.00000	NA	NA
YES	AT1G02500	6460	3.57	3.86	0.57	6.27	0.00000	0.00000	AtSAM1	0
	AT3G09260	147	3.54	4.15	0.76	4.68	0.00000	0.00012	BGLU23	0
	AT2G14580	10	3.53	8.99	1.05	3.36	0.00079	0.00867	ATPRB1	basic pathogenesis-related protein 1
YES	AT3G06350	683	3.52	3.77	0.53	6.68	0.00000	0.00000	EMB3004	EMBRYO DEFECTIVE 3004
	AT2G23540	8	3.51	8.58	1.05	3.36	0.00079	0.00868	NA	NA
	AT1G64060	210	3.51	3.86	0.61	5.72	0.00000	0.00000	ATRBOH F	respiratory burst oxidase protein F
	AT1G34460	7	3.51	8.53	1.05	3.35	0.00080	0.00873	CYC3	CYCLIN 3
YES	AT5G42830	545	3.49	3.74	0.53	6.56	0.00000	0.00000	NA	NA
	AT5G67100	11	3.46	9.06	1.06	3.27	0.00106	0.01076	ICU2	INCURVATA2
	AT5G01870	21	3.45	4.71	0.92	3.76	0.00017	0.00274	NA	NA
	AT3G03130	13	3.43	6.17	1.03	3.35	0.00082	0.00889	NA	NA
YES	AT5G12300	198	3.42	3.71	0.57	5.95	0.00000	0.00000	NA	NA
YES	AT1G68050	183	3.42	3.86	0.68	5.03	0.00000	0.00003	ADO3	0
	AT3G16450	9	3.40	8.82	1.06	3.22	0.00127	0.01220	JAL33	Jacalin-related lectin 33
	AT4G01970	9	3.39	5.64	1.00	3.38	0.00073	0.00818	AtSTS	stachyose synthase
	AT5G57390	16	3.39	5.41	1.00	3.39	0.00071	0.00802	AIL5	AINTEGUMENTA-like 5
	AT3G09370	58	3.38	3.99	0.76	4.43	0.00001	0.00030	AtMYB3R3	myb domain protein 3R3
YES	AT4G39980	1596	3.34	3.60	0.55	6.07	0.00000	0.00000	DHS1	3-deoxy-D-arabino-heptulosonate 7-phosphate synthase 1
	AT1G65880	7	3.34	8.50	1.05	3.17	0.00155	0.01424	BZO1	benzoyloxyglucosinolate 1
YES	AT5G06540	16	3.34	4.80	0.95	3.50	0.00046	0.00583	NA	NA
YES	AT1G78990	9	3.34	8.76	1.06	3.16	0.00160	0.01461	NA	NA
	AT4G11320	402	3.33	3.69	0.63	5.30	0.00000	0.00001	AtCP2	0
YES	AT1G71691	7	3.33	8.43	1.05	3.16	0.00157	0.01443	NA	NA
	AT5G62710	94	3.30	3.70	0.66	4.96	0.00000	0.00004	NA	NA
YES	AT1G51680	1753	3.28	3.47	0.49	6.72	0.00000	0.00000	4CL.1	4-COUMARATE:COA LIGASE 1
YES	AT5G48930	9809	3.28	3.52	0.54	6.03	0.00000	0.00000	HCT	hydroxycinnamoyl-CoA shikimate/quinate hydroxycinnamoyl transferase
	AT1G31770	39	3.27	3.98	0.81	4.05	0.00005	0.00113	ABCG14	ATP-binding cassette G14
	AT2G16890	123	3.25	3.57	0.62	5.28	0.00000	0.00001	NA	NA
	AT1G33700	34	3.22	3.78	0.75	4.31	0.00002	0.00049	NA	NA
	AT1G66860	12	3.22	4.97	0.99	3.26	0.00110	0.01105	NA	NA
	AT3G20370	22	3.21	4.03	0.85	3.79	0.00015	0.00253	NA	NA
	AT1G32415	11	3.20	4.91	0.99	3.25	0.00115	0.01141	NA	NA
	AT4G06744	18	3.20	4.24	0.90	3.55	0.00038	0.00510	NA	NA
	AT4G34160	62	3.19	3.61	0.68	4.67	0.00000	0.00012	CYCD3	0
	AT1G78650	28	3.18	4.18	0.89	3.56	0.00038	0.00506	POLD3	0
	AT5G38930	8	3.18	8.57	1.06	2.99	0.00276	0.02191	NA	NA
YES	AT2G16720	219	3.15	3.51	0.65	4.87	0.00000	0.00006	ATMYB7	ARABIDOPSIS THALIANA MYB DOMAIN PROTEIN 7
	AT2G39510	10	3.15	5.84	1.04	3.04	0.00238	0.01974	UMAMIT14	Usually multiple acids move in and out Transporters 14
YES	AT2G30490	5283	3.15	3.34	0.49	6.39	0.00000	0.00000	ATC4H	CINNAMATE 4-HYDROXYLASE
	AT1G65890	75	3.14	3.53	0.67	4.69	0.00000	0.00011	AAE12	acyl activating enzyme 12
	AT4G29560	16	3.13	4.32	0.93	3.37	0.00075	0.00840	NA	NA
	AT4G02290	98	3.12	3.57	0.71	4.42	0.00001	0.00031	AtGH9B13	glycosyl hydrolase 9B13
	AT1G11220	262	3.12	3.42	0.61	5.13	0.00000	0.00002	NA	NA
	AT1G67650	12	3.10	4.99	1.01	3.08	0.00205	0.01753	NA	NA
	AT1G13400	7	3.10	8.49	1.06	2.91	0.00356	0.02637	JGL	JAGGED-LIKE
	AT5G28490	70	3.10	3.65	0.76	4.09	0.00004	0.00099	LSH1	LIGHT-DEPENDENT SHORT HYPOCOTYLS 1
	AT2G29760	51	3.10	3.55	0.71	4.36	0.00001	0.00039	OTP81	ORGANELLE TRANSCRIPT PROCESSING 81
	AT2G43840	13	3.09	5.19	1.02	3.02	0.00249	0.02043	UGT74F1	UDP-glycosyltransferase 74 F1
	AT2G34180	22	3.09	4.53	0.97	3.18	0.00149	0.01385	ATWL2	WPL4-LIKE 2
YES	AT2G44160	2131	3.09	3.30	0.52	5.95	0.00000	0.00000	MTHFR2	methylenetetrahydrofolate reductase 2
	AT5G08110	19	3.09	4.30	0.94	3.28	0.00103	0.01057	NA	NA
	AT4G28690	32	3.09	4.04	0.90	3.45	0.00057	0.00683	NA	NA
	AT5G09570	49	3.08	3.87	0.85	3.61	0.00031	0.00435	NA	NA
	AT1G78930	29	3.07	3.80	0.83	3.69	0.00023	0.00346	NA	NA

Supplemental Table 1-RNA seq data comparing Pro35S::MYB63 with wild type.

DAP.MYB63.target(1.5kb upstream TSS)	Gene	baseMean	log2FoldChange	lfcMLE	lfcSE	stat	pvalue	padj (FDR)	TAIR10 Symbol	TAIR10 Annotation (Short)
	AT5G09480	30	3.06	4.07	0.91	3.36	0.00078	0.00860	NA	NA
	AT1G65710	23	3.04	3.72	0.81	3.73	0.00019	0.00297	NA	NA
	AT1G38131	10	3.04	5.82	1.05	2.90	0.00369	0.02702	NA	NA
	AT3G54630	22	3.04	3.79	0.84	3.63	0.00029	0.00408	NA	NA
	AT1G16520	34	3.02	3.60	0.77	3.91	0.00009	0.00176	NA	NA
YES	AT3G51240	316	3.01	3.29	0.59	5.09	0.00000	0.00002	F3'H	0
	AT1G17665	26	3.00	4.27	0.96	3.13	0.00177	0.01584	NA	NA
	AT2G18150	15	3.00	4.20	0.94	3.18	0.00146	0.01359	NA	NA
	AT4G14200	27	2.99	3.93	0.90	3.34	0.00083	0.00899	NA	NA
	AT1G66300	7	2.99	8.38	1.07	2.81	0.00501	0.03368	NA	NA
	AT1G05440	7	2.97	8.35	1.07	2.79	0.00531	0.03523	NA	NA
	AT4G04370	22	2.97	3.75	0.85	3.47	0.00051	0.00636	NA	NA
YES	AT3G03780	8233	2.97	3.17	0.53	5.58	0.00000	0.00000	ATMS2	methionine synthase 2
	AT1G56110	275	2.96	3.23	0.59	5.00	0.00000	0.00003	NOP56	homolog of nucleolar protein NOP56
	AT1G62310	28	2.96	3.86	0.89	3.31	0.00095	0.00987	NA	NA
	AT3G13670	35	2.95	3.71	0.85	3.46	0.00055	0.00664	NA	NA
	AT3G17690	20	2.95	4.12	0.95	3.11	0.00188	0.01647	ATCNGC19	CYCLIC NUCLEOTIDE-GATED CHANNEL 19
	AT1G26540	14	2.94	4.63	1.00	2.93	0.00343	0.02573	NA	NA
	AT3G15950	28	2.93	3.60	0.82	3.56	0.00037	0.00500	NAI2	0
	AT4G33150	235	2.92	3.13	0.53	5.49	0.00000	0.00000	LKR	0
	AT4G02150	46	2.92	3.38	0.73	3.98	0.00007	0.00138	ATIMPALPH	IMPORTIN ALPHA 3
	AT2G04880	97	2.92	3.28	0.67	4.37	0.00001	0.00038	ATWRKY1	0
	AT5G62730	21	2.91	3.55	0.81	3.61	0.00031	0.00433	NA	NA
	AT4G03340	36	2.91	3.34	0.71	4.10	0.00004	0.00095	NA	NA
	AT1G35910	31	2.91	3.57	0.82	3.53	0.00041	0.00535	TPPD	trehalose-6-phosphate phosphatase D
	AT3G19960	114	2.90	3.28	0.68	4.28	0.00002	0.00053	ATM1	myosin 1
	AT5G56300	36	2.90	3.72	0.87	3.32	0.00090	0.00956	GAMT2	gibberellic acid methyltransferase 2
	AT2G20710	9	2.90	4.57	1.00	2.90	0.00374	0.02721	NA	NA
	AT4G21530	51	2.89	3.60	0.84	3.43	0.00060	0.00718	APC4	anaphase promoting complex 4
	AT3G05190	15	2.89	4.04	0.95	3.05	0.00226	0.01890	NA	NA
	AT3G16430	6	2.89	8.26	1.07	2.70	0.00691	0.04264	JAL31	jacalin-related lectin 31
	AT5G03300	1675	2.88	3.04	0.48	6.03	0.00000	0.00000	ADK2	adenosine kinase 2
	AT3G01330	14	2.87	3.81	0.90	3.18	0.00148	0.01376	DEL3	DP-E2F-like protein 3
	AT1G20870	53	2.87	3.64	0.87	3.31	0.00094	0.00985	NA	NA
	AT3G23370	14	2.84	4.14	0.97	2.93	0.00338	0.02545	NA	NA
	AT1G75120	15	2.84	4.32	0.99	2.86	0.00428	0.02999	RRA1	REDUCED RESIDUAL ARABINOSE 1
	AT5G03870	25	2.84	3.51	0.83	3.42	0.00062	0.00735	NA	NA
	AT1G69060	96	2.83	3.11	0.62	4.58	0.00000	0.00018	NA	NA
	AT2G19590	77	2.82	3.22	0.70	4.01	0.00006	0.00129	ACO1	ACC oxidase 1
	AT2G21260	8	2.81	5.47	1.05	2.67	0.00753	0.04516	NA	NA
	AT4G05631	11	2.81	4.83	1.03	2.72	0.00649	0.04068	NA	NA
	AT1G19250	25	2.81	3.51	0.85	3.32	0.00091	0.00962	FMO1	flavin-dependent monooxygenase 1
	AT5G23020	337	2.80	3.01	0.55	5.13	0.00000	0.00002	IMS2	2-isopropylmalate synthase 2
	AT3G03970	37	2.79	3.19	0.70	3.99	0.00007	0.00136	NA	NA
	ATCG00340	343	2.79	3.37	0.81	3.47	0.00053	0.00650	PSAB	0
	AT2G01730	11	2.79	4.24	0.99	2.81	0.00503	0.03372	ATCPSF73-I	cleavage and polyadenylation specificity factor 73 kDa subunit-II
YES	AT4G25515	24	2.78	3.54	0.87	3.22	0.00129	0.01238	SLK3	SEUSS-like 3
	AT1G04050	25	2.78	3.84	0.94	2.95	0.00316	0.02426	SDG13	SET DOMAIN PROTEIN 13
	AT1G02290	40	2.77	3.31	0.78	3.55	0.00039	0.00517	NA	NA
	AT3G09730	17	2.77	3.58	0.88	3.15	0.00164	0.01488	NA	NA
	AT1G55040	8	2.77	5.39	1.05	2.63	0.00848	0.04867	NA	NA
YES	AT5G13420	1124	2.76	2.94	0.51	5.38	0.00000	0.00001	TRA2	transaldolase 2
YES	AT4G13930	2025	2.74	2.97	0.57	4.78	0.00000	0.00008	SHM4	serine hydroxymethyltransferase 4
	AT3G59510	10	2.74	4.18	0.99	2.76	0.00585	0.03778	NA	NA
	AT1G43910	91	2.73	3.09	0.68	4.00	0.00006	0.00131	NA	NA
	AT3G57600	41	2.73	3.29	0.80	3.42	0.00062	0.00735	NA	NA
	AT1G02970	21	2.72	3.41	0.85	3.21	0.00132	0.01259	ATWEE1	0
	AT5G61400	10	2.72	4.10	0.99	2.75	0.00600	0.03845	NA	NA
	AT2G45490	9	2.71	4.56	1.03	2.64	0.00831	0.04814	AtAUR3	ataurora3
	AT1G04730	25	2.71	3.36	0.83	3.26	0.00111	0.01114	CTF18	CHROMOSOME TRANSMISSION FIDELITY 18
	AT3G26782	30	2.71	3.27	0.80	3.39	0.00069	0.00791	NA	NA
	AT2G30370	15	2.71	3.85	0.96	2.83	0.00469	0.03211	CHAL	CHALLAH
	AT4G36260	13	2.70	3.75	0.94	2.87	0.00412	0.02919	SRS2	SHI RELATED SEQUENCE 2
	AT5G20600	96	2.69	3.10	0.72	3.74	0.00019	0.00295	NA	NA
	AT1G27680	115	2.69	2.94	0.60	4.49	0.00001	0.00024	APL2	ADPGLC-PPase large subunit
	AT1G73710	50	2.69	3.13	0.74	3.63	0.00029	0.00409	NA	NA
	AT2G23740	24	2.68	3.41	0.87	3.10	0.00192	0.01671	SUVR5	SU(VAR)3-9-RELATED protein 5

Supplemental Table 1-RNA seq data comparing Pro35S::MYB63 with wild type.

DAP.MYB63.target(1.5kb upstream TSS)	Gene	baseMean	log2FoldChange	lfcMLE	lfcSE	stat	pvalue	padj (FDR)	TAIR10 Symbol	TAIR10 Annotation (Short)
	AT4G34990	96	2.68	3.04	0.69	3.90	0.00010	0.00182	AtMYB32	myb domain protein 32
	AT5G40010	15	2.68	3.61	0.92	2.92	0.00355	0.02633	AATP1	AAA-ATPase 1
	AT5G20610	87	2.68	3.15	0.76	3.53	0.00042	0.00546	NA	NA
YES	AT5G66120	2089	2.68	2.82	0.47	5.72	0.00000	0.00000	NA	NA
	AT2G24300	10	2.67	4.12	1.00	2.68	0.00747	0.04487	NA	NA
	AT5G27120	195	2.67	2.95	0.63	4.25	0.00002	0.00059	NA	NA
YES	AT3G27570	323	2.67	2.83	0.50	5.36	0.00000	0.00001	NA	NA
	AT1G11600	33	2.67	3.30	0.83	3.20	0.00139	0.01307	CYP77B1	cytochrome P450, family 77, subfamily B, polypeptide 1
	AT4G39160	38	2.67	3.14	0.76	3.49	0.00048	0.00609	NA	NA
YES	AT3G44720	3064	2.66	2.81	0.48	5.57	0.00000	0.00000	ADT4	arogenate dehydratase 4
	AT1G34180	92	2.66	3.07	0.73	3.66	0.00025	0.00366	anac016	NAC domain containing protein 16
	AT1G72220	12	2.65	3.96	0.99	2.69	0.00721	0.04392	NA	NA
	AT3G54260	115	2.64	2.92	0.63	4.17	0.00003	0.00074	TBL36	TRICHOME BIREFRINGENCE-LIKE 36
YES	AT1G68710	30	2.64	3.20	0.81	3.27	0.00108	0.01092	NA	NA
	AT3G50040	21	2.62	3.24	0.83	3.15	0.00165	0.01494	NA	NA
	AT5G43020	71	2.61	2.92	0.65	4.00	0.00006	0.00131	NA	NA
	AT3G49240	61	2.60	3.01	0.73	3.58	0.00034	0.00466	emb1796	embryo defective 1796
YES	AT5G55340	28	2.60	3.26	0.85	3.06	0.00220	0.01855	NA	NA
	AT3G20260	45	2.60	2.94	0.69	3.78	0.00016	0.00259	NA	NA
	AT5G43500	88	2.59	2.93	0.68	3.81	0.00014	0.00239	ARP9	actin-related protein 9
	AT5G18580	963	2.59	2.99	0.73	3.55	0.00039	0.00516	EMB40	EMBRYO DEFECTIVE 40
YES	AT3G13100	28	2.59	3.11	0.80	3.25	0.00115	0.01142	ABCC7	ATP-binding cassette C7
	AT5G53440	273	2.58	3.09	0.79	3.26	0.00112	0.01118	NA	NA
	AT4G12870	22	2.58	3.25	0.86	3.01	0.00259	0.02097	NA	NA
	AT3G51480	13	2.58	3.51	0.93	2.77	0.00556	0.03638	ATGLR3.6	glutamate receptor 3.6
	AT3G55720	34	2.58	3.29	0.87	2.95	0.00316	0.02426	NA	NA
	ATCG00350	296	2.58	3.11	0.81	3.19	0.00141	0.01319	PSAA	0
	AT2G22410	12	2.57	3.82	0.98	2.62	0.00883	0.04999	SLO1	SLOW GROWTH 1
	AT1G71830	27	2.57	3.12	0.81	3.18	0.00148	0.01370	ATSERK1	SOMATIC EMBRYOGENESIS RECEPTOR-LIKE KINASE 1
	AT1G75170	118	2.57	2.79	0.58	4.46	0.00001	0.00027	NA	NA
	AT1G63640	32	2.57	3.13	0.82	3.14	0.00168	0.01519	NA	NA
	AT1G29170	51	2.56	3.08	0.80	3.21	0.00135	0.01277	ATSCAR3	WASP (WISKOTT-ALDRICH SYNDROME PROTEIN)-FAMILY VERPROLIN HOMOLOGOUS PROTEIN 2
	AT1G63160	64	2.56	2.87	0.67	3.83	0.00013	0.00225	EMB2811	EMBRYO DEFECTIVE 2811
	AT2G21140	503	2.56	2.87	0.66	3.85	0.00012	0.00209	ATPRP2	proline-rich protein 2
	AT5G28646	10	2.55	3.72	0.97	2.62	0.00881	0.04990	WVD2	WAVE-DAMPENED 2
	AT3G01770	82	2.54	2.84	0.66	3.87	0.00011	0.00196	ATBET10	bromodomain and extraterminal domain protein 10
	AT2G19190	23	2.54	3.13	0.83	3.06	0.00222	0.01863	FRK1	FLG22-induced receptor-like kinase 1
	AT5G45150	27	2.54	3.20	0.86	2.95	0.00315	0.02423	RTL3	RNAse THREE-like protein 3
	AT5G64000	158	2.53	2.76	0.58	4.37	0.00001	0.00039	ATSAL2	0
	AT5G17760	118	2.53	2.80	0.62	4.06	0.00005	0.00107	NA	NA
	AT1G64600	19	2.53	3.31	0.90	2.81	0.00492	0.03321	NA	NA
	AT5G22220	30	2.53	3.05	0.80	3.16	0.00160	0.01462	ATE2FB	0
	AT2G16250	38	2.53	3.29	0.90	2.82	0.00486	0.03294	NA	NA
	AT5G08000	190	2.52	2.73	0.57	4.44	0.00001	0.00029	E13L3	glucan endo-1,3-beta-glucosidase-like protein 3
	AT4G39230	149	2.52	2.83	0.67	3.79	0.00015	0.00253	NA	NA
	AT3G24660	39	2.52	2.96	0.76	3.31	0.00092	0.00968	TMKL1	transmembrane kinase-like 1
	AT4G34860	32	2.51	2.99	0.78	3.22	0.00127	0.01222	A/N-InvB	alkaline/neutral invertase B
	AT5G56580	58	2.51	3.01	0.79	3.16	0.00159	0.01453	ANQ1	ARABIDOPSIS NQK1
	AT3G27580	54	2.51	2.83	0.68	3.70	0.00021	0.00330	ATPK7	0
	AT5G13690	41	2.50	3.34	0.92	2.70	0.00692	0.04268	CYL1	CYCLOPS 1
YES	AT5G65710	77	2.49	2.72	0.59	4.22	0.00002	0.00063	HSL2	HAESA-like 2
	AT5G04895	18	2.49	3.12	0.85	2.92	0.00354	0.02629	ABO6	ABA overly sensitive 6
YES	AT1G22410	3663	2.47	2.62	0.49	5.03	0.00000	0.00003	NA	NA
	AT1G10780	27	2.47	2.89	0.75	3.29	0.00099	0.01016	NA	NA
	AT1G60700	21	2.46	3.01	0.82	3.01	0.00264	0.02124	NA	NA
	AT5G64960	45	2.46	2.98	0.81	3.03	0.00245	0.02021	CDKC2	cyclin dependent kinase group C2
	AT5G20850	21	2.45	2.96	0.80	3.05	0.00229	0.01906	ATRAD51	0
	AT5G62990	20	2.45	3.32	0.93	2.62	0.00867	0.04943	emb1692	embryo defective 1692
	AT5G37530	25	2.44	2.97	0.81	3.01	0.00261	0.02104	NA	NA
	AT1G73330	92	2.44	2.76	0.68	3.57	0.00035	0.00482	ATDR4	drought-repressed 4
	AT1G32610	21	2.43	2.96	0.81	3.00	0.00269	0.02149	NA	NA
	AT2G40340	18	2.43	3.02	0.84	2.89	0.00385	0.02777	AtERF48	0
	AT4G18300	18	2.43	2.98	0.82	2.96	0.00306	0.02370	NA	NA

Supplemental Table 1-RNA seq data comparing Pro35S::MYB63 with wild type.

DAP.MYB63.target(1.5kb upstream TSS)	Gene	baseMean	log2FoldChange	lfcMLE	lfcSE	stat	pvalue	padj (FDR)	TAIR10 Symbol	TAIR10 Annotation (Short)
	AT3G23810	5741	2.43	2.58	0.51	4.81	0.00000	0.00007	ATSAHH2	S-ADENOSYL-L-HOMOCYSTEINE (SAH) HYDROLASE 2
	AT2G04030	545	2.43	2.63	0.58	4.20	0.00003	0.00067	AtHsp90.5	HEAT SHOCK PROTEIN 90.5
	AT1G28130	33	2.42	2.93	0.81	3.01	0.00264	0.02121	GH3.17	0
	AT4G19430	73	2.42	2.66	0.61	3.96	0.00008	0.00148	NA	NA
	AT1G02630	19	2.42	3.15	0.90	2.70	0.00691	0.04261	NA	NA
	AT4G11160	121	2.42	2.80	0.73	3.30	0.00096	0.00997	NA	NA
	AT3G10530	34	2.41	2.95	0.82	2.93	0.00336	0.02532	NA	NA
	AT5G55600	72	2.41	2.70	0.66	3.63	0.00029	0.00412	NA	NA
	AT1G60860	30	2.41	3.03	0.86	2.80	0.00510	0.03414	AGD2	ARF-GAP domain 2
	AT5G16750	85	2.40	2.79	0.74	3.25	0.00114	0.01132	TOZ	TORMOZEMBRYO DEFECTIVE
	AT5G39900	45	2.40	2.97	0.84	2.87	0.00409	0.02905	NA	NA
	AT4G21270	31	2.40	2.97	0.84	2.87	0.00405	0.02887	ATK1	kinesin 1
	AT3G27220	24	2.40	3.02	0.86	2.80	0.00512	0.03421	NA	NA
	AT1G26410	47	2.40	2.71	0.69	3.50	0.00047	0.00597	NA	NA
	AT3G23290	131	2.39	2.63	0.61	3.94	0.00008	0.00158	LSH4	LIGHT SENSITIVE HYPOCOTYLS 4
	AT1G33790	22	2.38	2.89	0.81	2.95	0.00313	0.02413	NA	NA
	AT4G15417	31	2.38	2.91	0.82	2.91	0.00362	0.02667	ATRTL1	RNAse II-like 1
	AT1G69200	102	2.37	2.61	0.61	3.90	0.00010	0.00181	FLN2	fructokinase-like 2
	AT5G09290	28	2.37	2.97	0.86	2.77	0.00563	0.03669	NA	NA
	AT3G11340	19	2.37	2.90	0.82	2.88	0.00395	0.02829	UGT76B1	UDP-dependent glycosyltransferase 76B1
	AT1G24150	191	2.37	2.53	0.52	4.52	0.00001	0.00022	ATFH4	FORMIN HOMOLOGUE 4
	AT2G40650	271	2.37	2.84	0.80	2.97	0.00294	0.02304	NA	NA
	AT4G26300	65	2.36	2.94	0.85	2.77	0.00565	0.03677	emb1027	embryo defective 1027
	AT3G61250	23	2.36	2.88	0.82	2.88	0.00401	0.02858	AtMYB17	myb domain protein 17
	AT5G05980	124	2.35	2.58	0.61	3.88	0.00011	0.00193	ATDFB	DHFS-FPGS homolog B
	AT1G48310	33	2.35	2.84	0.80	2.93	0.00340	0.02555	CHA18	0
	AT4G01730	26	2.34	2.78	0.78	3.02	0.00249	0.02043	NA	NA
	AT5G22650	668	2.34	2.52	0.55	4.28	0.00002	0.00053	ATHD2	ARABIDOPSIS HISTONE DEACETYLASE 2
	AT5G54200	28	2.34	2.74	0.75	3.13	0.00178	0.01586	NA	NA
	AT2G07690	68	2.34	2.70	0.72	3.23	0.00125	0.01203	MCM5	MINICHROMOSOME MAINTENANCE 5
	AT1G49320	101	2.34	2.58	0.62	3.78	0.00015	0.00256	ATUSPL1	unknown seed protein like 1
	AT3G48670	92	2.33	2.71	0.74	3.16	0.00159	0.01454	IDN2	INVOLVED IN DE NOVO 2
	AT2G22250	630	2.33	2.49	0.52	4.46	0.00001	0.00028	AAT	aspartate aminotransferase
	AT3G06290	37	2.33	2.81	0.80	2.90	0.00373	0.02720	AtSAC3B	0
	AT3G21060	87	2.32	2.65	0.70	3.31	0.00094	0.00985	RBL	RbBP5 LIKE
	AT5G10060	57	2.32	2.65	0.71	3.27	0.00108	0.01088	NA	NA
	AT4G36150	31	2.32	2.83	0.82	2.82	0.00486	0.03290	NA	NA
	AT5G56050	21	2.32	2.92	0.86	2.69	0.00711	0.04351	NA	NA
	AT5G07830	142	2.32	2.51	0.57	4.08	0.00005	0.00103	AtGUS2	glucuronidase 2
	AT1G21250	655	2.31	2.60	0.67	3.46	0.00055	0.00664	AtWAK1	0
	AT3G03060	36	2.31	2.79	0.80	2.87	0.00410	0.02910	NA	NA
	AT4G28390	158	2.31	2.48	0.54	4.29	0.00002	0.00052	AAC3	ADP/ATP carrier 3
	AT5G22840	28	2.31	2.71	0.75	3.06	0.00220	0.01851	NA	NA
	AT1G71210	48	2.31	2.74	0.78	2.96	0.00308	0.02379	NA	NA
	AT2G07170	26	2.31	2.93	0.87	2.65	0.00800	0.04707	NA	NA
	AT1G14740	268	2.30	2.47	0.54	4.28	0.00002	0.00052	TTA1	TITANIA 1
	AT3G11760	90	2.30	2.61	0.70	3.31	0.00095	0.00987	NA	NA
YES	AT4G35880	79	2.30	2.54	0.63	3.67	0.00024	0.00357	NA	NA
	AT1G09710	40	2.30	2.61	0.70	3.29	0.00098	0.01015	NA	NA
	AT2G12550	56	2.29	2.72	0.77	2.96	0.00306	0.02370	NUB1	homolog of human NUB1
YES	AT1G76900	433	2.29	2.56	0.66	3.47	0.00052	0.00646	AtTLP1	tubby like protein 1
	AT2G28250	23	2.29	2.76	0.80	2.86	0.00428	0.02999	NCRK	0
	AT1G09470	22	2.29	2.83	0.84	2.73	0.00630	0.03977	NA	NA
	AT1G20950	767	2.28	2.43	0.51	4.49	0.00001	0.00024	NA	NA
	AT5G20510	61	2.28	2.54	0.65	3.50	0.00046	0.00580	AL5	alfin-like 5
YES	AT5G50370	279	2.28	2.43	0.51	4.43	0.00001	0.00030	NA	NA
	AT3G23890	34	2.27	2.86	0.86	2.63	0.00847	0.04865	ATTOPII	0
	AT4G26760	66	2.26	2.67	0.76	2.97	0.00299	0.02336	MAP65-2	microtubule-associated protein 65-2
	AT1G80150	22	2.26	2.69	0.78	2.91	0.00361	0.02661	NA	NA
	AT1G72040	189	2.25	2.48	0.61	3.68	0.00024	0.00354	AtdNK	0
	AT1G07050	230	2.25	2.46	0.59	3.79	0.00015	0.00251	NA	NA
	AT2G20310	69	2.25	2.65	0.76	2.95	0.00314	0.02418	RIN13	RPM1 interacting protein 13
YES	AT5G19300	52	2.25	2.70	0.80	2.83	0.00465	0.03184	NA	NA
	AT1G08560	114	2.25	2.46	0.60	3.75	0.00017	0.00280	ATSYP111	0
	AT4G24760	84	2.25	2.59	0.72	3.11	0.00188	0.01647	NA	NA
	AT4G13070	57	2.25	2.54	0.68	3.31	0.00095	0.00987	NA	NA
	AT5G18950	33	2.25	2.67	0.78	2.89	0.00391	0.02811	NA	NA

Supplemental Table 1-RNA seq data comparing Pro35S::MYB63 with wild type.

DAP.MYB63.target(1.5kb upstream TSS)	Gene	baseMean	log2FoldChange	lfcMLE	lfcSE	stat	pvalue	padj (FDR)	TAIR10 Symbol	TAIR10 Annotation (Short)
	AT3G26410	115	2.25	2.51	0.66	3.42	0.00063	0.00741	AtTRM11	0
	AT4G00480	35	2.24	2.55	0.70	3.22	0.00129	0.01238	ATMYC1	0
	AT1G29030	28	2.24	2.76	0.83	2.70	0.00685	0.04235	NA	NA
	AT1G27030	456	2.24	2.41	0.55	4.10	0.00004	0.00096	NA	NA
	AT5G58003	66	2.24	2.55	0.69	3.23	0.00123	0.01199	CPL4	C-terminal domain phosphatase-like 4
	AT2G20550	43	2.24	2.55	0.70	3.20	0.00139	0.01307	NA	NA
	AT1G17850	35	2.23	2.56	0.71	3.15	0.00164	0.01490	NA	NA
	AT5G25160	20	2.23	2.78	0.85	2.63	0.00842	0.04850	ZFP3	zinc finger protein 3
	AT2G43360	770	2.23	2.41	0.57	3.90	0.00010	0.00177	BIO2	BIOTIN AUXOTROPH 2
	AT3G09200	3108	2.23	2.40	0.56	4.00	0.00006	0.00131	NA	NA
	AT1G53720	51	2.23	2.49	0.66	3.37	0.00076	0.00850	ATCYP59	CYCLOPHILIN 59
YES	AT1G28680	225	2.23	2.47	0.63	3.51	0.00045	0.00578	NA	NA
	AT4G00026	120	2.23	2.40	0.56	3.98	0.00007	0.00141	SD3	SEGREGATION DISTORTION 3
	AT1G09970	839	2.22	2.45	0.62	3.60	0.00032	0.00444	LRR XI-23	0
YES	AT5G17770	2312	2.22	2.36	0.51	4.40	0.00001	0.00034	ATCBR	NADH:cytochrome B5 reductase 1
	AT2G35540	36	2.22	2.70	0.81	2.74	0.00615	0.03908	NA	NA
	AT5G10760	1266	2.22	2.37	0.52	4.30	0.00002	0.00049	NA	NA
	AT2G29890	24	2.22	2.67	0.80	2.76	0.00572	0.03711	ATVLN1	0
	AT3G20440	31	2.22	2.60	0.76	2.94	0.00332	0.02513	BE1	BRANCHING ENZYME 1
	AT4G26090	72	2.22	2.65	0.79	2.80	0.00506	0.03390	RPS2	RESISTANT TO P. SYRINGAE 2
	AT1G15440	85	2.21	2.52	0.70	3.16	0.00158	0.01446	ATPWP2	PERIODIC TRYPTOPHAN PROTEIN 2
	AT1G12050	649	2.21	2.33	0.47	4.72	0.00000	0.00010	AtFAH	0
	AT2G29140	117	2.21	2.50	0.68	3.26	0.00113	0.01125	APUM3	pumilio 3
	AT4G09140	70	2.21	2.52	0.70	3.14	0.00167	0.01505	ATMLH1	ARABIDOPSIS THALIANA MUTL-HOMOLOGUE 1
	AT1G33960	188	2.21	2.45	0.64	3.47	0.00052	0.00640	AIG1	AVRRPT2-INDUCED GENE 1
	AT1G13640	86	2.21	2.40	0.59	3.74	0.00018	0.00291	NA	NA
	AT2G37380	35	2.20	2.72	0.84	2.62	0.00867	0.04943	MAKR3	MEMBRANE-ASSOCIATED KINASE REGULATOR 3
YES	AT1G48860	531	2.20	2.33	0.50	4.42	0.00001	0.00031	NA	NA
	AT1G71850	26	2.20	2.70	0.83	2.64	0.00827	0.04805	NA	NA
	AT3G27740	794	2.19	2.33	0.50	4.37	0.00001	0.00038	CARA	carbamoyl phosphate synthetase A
	AT5G53770	73	2.19	2.65	0.80	2.73	0.00641	0.04031	NA	NA
	AT2G05120	45	2.19	2.53	0.73	3.00	0.00272	0.02163	NA	NA
YES	AT1G08250	393	2.19	2.33	0.51	4.26	0.00002	0.00056	ADT6	arogenate dehydratase 6
YES	AT1G09430	1375	2.19	2.30	0.47	4.63	0.00000	0.00014	ACLA-3	ATP-citrate lyase A-3
	AT1G23280	47	2.19	2.51	0.71	3.07	0.00216	0.01828	NA	NA
	AT1G63720	58	2.18	2.45	0.66	3.30	0.00097	0.01001	NA	NA
	AT3G13610	72	2.18	2.44	0.66	3.31	0.00094	0.00981	F6'H1	0
	AT2G16270	24	2.18	2.54	0.75	2.92	0.00347	0.02593	NA	NA
	AT5G52010	71	2.18	2.39	0.61	3.55	0.00039	0.00519	NA	NA
	AT5G12400	53	2.18	2.53	0.74	2.94	0.00331	0.02509	NA	NA
	AT5G37350	45	2.17	2.47	0.70	3.12	0.00181	0.01609	NA	NA
	AT5G67270	45	2.17	2.53	0.74	2.92	0.00348	0.02600	ATEB1C	MICROTUBULE END BINDING PROTEIN 1
	AT4G09510	185	2.17	2.36	0.57	3.78	0.00016	0.00259	A/N-InvI	alkaline/neutral invertase I
	AT1G08600	83	2.17	2.55	0.76	2.87	0.00413	0.02922	ATRX	0
	AT5G09590	41	2.17	2.58	0.78	2.79	0.00523	0.03483	HSC70-5	HEAT SHOCK COGNATE
	AT1G51790	57	2.17	2.53	0.74	2.93	0.00342	0.02571	NA	NA
	AT3G21560	235	2.17	2.36	0.59	3.69	0.00023	0.00345	BRT1	Bright Trichomes 1
	AT1G32340	68	2.17	2.42	0.65	3.32	0.00091	0.00957	NHL8	NDR1/HIN1-like 8
	AT1G70140	41	2.17	2.47	0.70	3.10	0.00192	0.01671	ATFH8	formin 8
	AT3G57940	36	2.16	2.53	0.75	2.89	0.00380	0.02752	NA	NA
	AT5G01100	138	2.16	2.35	0.57	3.77	0.00017	0.00271	FRB1	FRIABLE 1
	AT5G63970	30	2.16	2.64	0.82	2.64	0.00827	0.04805	RGLG3	RING DOMAIN LIGASE 3
	AT2G42650	27	2.16	2.57	0.78	2.78	0.00547	0.03596	NA	NA
	AT4G24200	44	2.16	2.61	0.81	2.68	0.00744	0.04479	NA	NA
	AT4G01870	114	2.16	2.37	0.61	3.52	0.00044	0.00562	NA	NA
	AT4G14510	63	2.16	2.57	0.78	2.76	0.00581	0.03758	ATCFM3B	0
	AT4G14690	131	2.15	2.36	0.60	3.57	0.00035	0.00481	ELIP2	EARLY LIGHT-INDUCIBLE PROTEIN 2
	AT3G10630	50	2.15	2.49	0.74	2.92	0.00345	0.02581	NA	NA
	AT1G63810	65	2.15	2.55	0.78	2.75	0.00591	0.03801	NA	NA
	AT4G27970	21	2.15	2.61	0.81	2.64	0.00826	0.04801	SLAH2	SLAC1 homologue 2
	AT5G24670	102	2.15	2.35	0.60	3.60	0.00032	0.00445	EMB2820	EMBRYO DEFECTIVE 2820
YES	AT2G17630	144	2.15	2.37	0.62	3.44	0.00057	0.00690	PSAT2	phosphoserine aminotransferase 2
	AT1G54385	68	2.14	2.36	0.62	3.43	0.00060	0.00716	NA	NA
	AT1G57700	30	2.14	2.54	0.78	2.74	0.00612	0.03897	NA	NA
	AT5G54160	3584	2.14	2.28	0.52	4.07	0.00005	0.00105	ATOMT1	O-methyltransferase 1
	AT1G23380	22	2.14	2.57	0.80	2.68	0.00731	0.04428	KNAT6	KNOTTED1-like homeobox gene 6
	AT3G19670	75	2.13	2.55	0.79	2.69	0.00724	0.04402	ATPRP40B	pre-mRNA-processing protein 40B

Supplemental Table 1-RNA seq data comparing Pro35S::MYB63 with wild type.

DAP.MYB63.target(1.5kb upstream TSS)	Gene	baseMean	log2FoldChange	lfcMLE	lfcSE	stat	pvalue	padj (FDR)	TAIR10 Symbol	TAIR10 Annotation (Short)
	AT3G20120	67	2.13	2.36	0.63	3.35	0.00079	0.00873	CYP705A21	cytochrome P450, family 705, subfamily A, polypeptide 21
	AT5G65750	115	2.13	2.47	0.74	2.86	0.00420	0.02964	NA	NA
	AT2G24120	191	2.12	2.33	0.60	3.51	0.00044	0.00564	PDE319	PIGMENT DEFECTIVE 319
	AT3G48870	290	2.12	2.37	0.66	3.21	0.00131	0.01250	ATCLPC	0
	AT5G37370	141	2.12	2.39	0.68	3.11	0.00186	0.01637	ATSRL1	0
	AT5G13120	1303	2.12	2.23	0.46	4.61	0.00000	0.00015	ATCYP20-2	ARABIDOPSIS THALIANA CYCLOPHILIN 20-2
	AT1G08610	30	2.12	2.47	0.75	2.84	0.00452	0.03121	NA	NA
	AT4G14770	42	2.12	2.41	0.70	3.02	0.00254	0.02066	ATTCX2	TESMIN/TSO1-LIKE CXC 2
	AT1G04980	126	2.11	2.28	0.56	3.77	0.00017	0.00271	ATPDI10	ARABIDOPSIS THALIANA PROTEIN DISULFIDE ISOMERASE 10
	AT1G54010	62	2.11	2.35	0.65	3.22	0.00128	0.01231	GLL23	GDSL-like lipase 23
	AT1G76930	905	2.11	2.36	0.66	3.17	0.00150	0.01389	ATEXT1	EXTENSIN 1
	AT1G01630	1015	2.10	2.21	0.47	4.51	0.00001	0.00023	NA	NA
	AT3G03640	345	2.10	2.23	0.51	4.12	0.00004	0.00088	BGLU25	beta glucosidase 25
	AT1G21240	61	2.10	2.37	0.68	3.09	0.00203	0.01743	WAK3	wall associated kinase 3
	AT5G47500	108	2.10	2.36	0.67	3.12	0.00183	0.01619	PME5	pectin methylesterase 5
	AT4G03100	44	2.09	2.35	0.67	3.14	0.00168	0.01518	NA	NA
	AT4G19860	699	2.09	2.23	0.51	4.07	0.00005	0.00106	NA	NA
	AT4G14310	45	2.09	2.41	0.73	2.88	0.00395	0.02829	NA	NA
	AT3G21770	56	2.09	2.36	0.68	3.06	0.00220	0.01855	NA	NA
	AT4G34910	67	2.09	2.37	0.69	3.02	0.00249	0.02043	NA	NA
	AT1G72090	64	2.09	2.40	0.72	2.89	0.00385	0.02777	NA	NA
	AT4G28650	32	2.09	2.47	0.78	2.68	0.00731	0.04428	NA	NA
YES	AT1G48570	105	2.08	2.28	0.60	3.46	0.00054	0.00659	NA	NA
	AT1G75040	2711	2.08	2.24	0.55	3.80	0.00015	0.00247	PR-5	0
	AT3G15590	83	2.08	2.35	0.69	3.02	0.00253	0.02060	NA	NA
	AT3G13310	2740	2.08	2.24	0.55	3.76	0.00017	0.00275	DJC66	DNA J protein C66
	AT5G58450	144	2.08	2.28	0.61	3.43	0.00061	0.00721	NA	NA
	AT2G32680	514	2.07	2.37	0.71	2.93	0.00344	0.02575	AtRLP23	receptor like protein 23
	AT2G38310	978	2.07	2.29	0.63	3.29	0.00100	0.01030	PYL4	PYR1-like 4
	AT2G21710	26	2.07	2.42	0.75	2.75	0.00604	0.03855	EMB2219	embryo defective 2219
	AT3G16620	45	2.07	2.39	0.73	2.83	0.00461	0.03166	ATTOC120	ARABIDOPSIS THALIANA TRANSLOCON OUTER COMPLEX PROTEIN 120
	AT5G56650	233	2.07	2.26	0.59	3.48	0.00050	0.00626	ILL1	IAA-leucine resistant (ILR)-like 1
	AT5G18370	22	2.06	2.44	0.77	2.68	0.00741	0.04467	NA	NA
	AT5G19950	40	2.06	2.40	0.74	2.79	0.00534	0.03537	NA	NA
	AT5G21040	166	2.06	2.27	0.62	3.32	0.00089	0.00948	FBX2	F-box protein 2
	AT3G12170	55	2.06	2.36	0.71	2.91	0.00366	0.02689	NA	NA
	AT4G04500	37	2.06	2.34	0.70	2.95	0.00318	0.02435	CRK37	cysteine-rich RLK (RECEPTOR-like protein kinase) 37
	AT3G07520	75	2.06	2.36	0.71	2.90	0.00378	0.02747	ATGLR1.4	0
	AT5G53890	45	2.06	2.32	0.68	3.03	0.00243	0.02009	AtPSKR2	0
	AT1G73620	104	2.06	2.25	0.59	3.46	0.00054	0.00661	NA	NA
	AT1G72730	85	2.06	2.27	0.63	3.29	0.00101	0.01040	NA	NA
	AT2G05940	454	2.06	2.18	0.49	4.19	0.00003	0.00070	RIPK	RPM1-induced protein kinase
	AT3G16260	135	2.06	2.26	0.62	3.32	0.00090	0.00952	TRZ4	tRNAse Z4
	AT4G13980	71	2.06	2.27	0.63	3.27	0.00107	0.01084	AT-HSFA5	0
	AT4G30490	454	2.05	2.20	0.53	3.85	0.00012	0.00211	NA	NA
	AT1G71350	47	2.05	2.30	0.66	3.11	0.00186	0.01638	NA	NA
	AT3G14980	50	2.05	2.37	0.73	2.82	0.00483	0.03278	IDM1	increased DNA methylation 1
	AT3G05830	85	2.05	2.34	0.70	2.93	0.00342	0.02571	NA	NA
	AT4G35850	135	2.05	2.23	0.59	3.45	0.00056	0.00677	NA	NA
	AT4G31270	61	2.04	2.35	0.72	2.85	0.00438	0.03049	NA	NA
	AT2G24690	35	2.04	2.40	0.76	2.70	0.00703	0.04321	NA	NA
	AT2G41890	31	2.04	2.39	0.76	2.70	0.00696	0.04279	NA	NA
	AT5G37010	48	2.04	2.34	0.71	2.85	0.00438	0.03049	NA	NA
	AT5G19320	104	2.04	2.29	0.67	3.04	0.00239	0.01975	RANGAP2	RAN GTPase activating protein 2
	AT1G58110	171	2.04	2.31	0.69	2.94	0.00323	0.02462	NA	NA
	AT1G66510	44	2.04	2.29	0.67	3.04	0.00233	0.01935	NA	NA
	AT5G58270	107	2.03	2.27	0.65	3.14	0.00171	0.01540	ABCB25	ATP-binding cassette B25
	AT3G05050	78	2.03	2.27	0.65	3.11	0.00190	0.01655	NA	NA
	AT1G07370	102	2.03	2.24	0.62	3.27	0.00107	0.01085	ATPCNA1	PROLIFERATING CELLULAR NUCLEAR ANTIGEN 1
	AT1G68620	66	2.03	2.39	0.77	2.64	0.00818	0.04775	NA	NA
	AT1G79080	81	2.03	2.23	0.62	3.28	0.00103	0.01057	NA	NA
	AT3G57430	77	2.02	2.28	0.68	2.99	0.00282	0.02226	OTP84	ORGANELLE TRANSCRIPT PROCESSING 84
	AT1G04510	306	2.02	2.15	0.50	4.02	0.00006	0.00122	MAC3A	MOS4-associated complex 3A

Supplemental Table 1-RNA seq data comparing Pro35S::MYB63 with wild type.

DAP.MYB63.target(1.5kb upstream TSS)	Gene	baseMean	log2FoldChange	lfcMLE	lfcSE	stat	pvalue	padj (FDR)	TAIR10 Symbol	TAIR10 Annotation (Short)
	AT5G49930	113	2.02	2.30	0.70	2.88	0.00400	0.02852	emb1441	embryo defective 1441
	AT5G03200	112	2.02	2.20	0.58	3.46	0.00054	0.00657	LUL1	LOG2-LIKE UBIQUITIN LIGASE1
	AT5G10920	266	2.02	2.16	0.52	3.85	0.00012	0.00211	NA	NA
	AT1G65540	106	2.02	2.25	0.65	3.09	0.00203	0.01743	AtLETM2	0
	AT3G02065	58	2.01	2.28	0.69	2.91	0.00360	0.02660	NA	NA
	AT3G17450	39	2.01	2.37	0.77	2.62	0.00870	0.04954	NA	NA
	AT1G48210	150	2.00	2.18	0.59	3.43	0.00061	0.00725	NA	NA
	AT5G01740	65	2.00	2.30	0.72	2.79	0.00521	0.03476	NA	NA
	AT5G39550	28	2.00	2.30	0.72	2.79	0.00521	0.03478	ORTH1	ORTHRUS 1
	AT2G25740	61	2.00	2.24	0.66	3.01	0.00259	0.02094	NA	NA
	AT1G47056	127	2.00	2.16	0.56	3.55	0.00039	0.00515	VFB1	VIER F-box proteine 1
	AT5G43060	194	2.00	2.16	0.56	3.54	0.00040	0.00522	RD21B	esponsive to dehydration 21B
	AT3G58660	84	2.00	2.31	0.73	2.73	0.00629	0.03974	NA	NA
	AT4G26455	106	2.00	2.29	0.71	2.80	0.00507	0.03394	AtWIP1	Arabidopsis thaliana WPP domain interacting protein 1
	AT3G16310	63	1.99	2.28	0.71	2.83	0.00469	0.03211	NA	NA
	AT2G30320	62	1.99	2.21	0.63	3.15	0.00163	0.01480	NA	NA
	AT5G58300	78	1.99	2.19	0.62	3.24	0.00121	0.01184	NA	NA
	AT4G11450	29	1.99	2.31	0.73	2.72	0.00654	0.04086	NA	NA
	AT4G30990	94	1.99	2.28	0.71	2.82	0.00480	0.03265	NA	NA
	AT3G47890	92	1.99	2.23	0.67	2.99	0.00280	0.02216	NA	NA
	AT3G10500	163	1.99	2.12	0.52	3.80	0.00014	0.00247	ANAC053	NAC domain containing protein 53
	AT5G14310	86	1.99	2.17	0.60	3.33	0.00087	0.00933	AtCXE16	carboxyesterase 16
	AT1G17110	118	1.99	2.30	0.73	2.72	0.00650	0.04073	UBP15	ubiquitin-specific protease 15
	AT5G44080	77	1.99	2.18	0.61	3.27	0.00109	0.01095	NA	NA
	AT4G31420	160	1.98	2.11	0.52	3.80	0.00015	0.00247	REIL1	REI1-LIKE 1
	AT2G17580	51	1.97	2.22	0.67	2.96	0.00309	0.02388	NA	NA
	AT3G02550	84	1.97	2.15	0.59	3.35	0.00081	0.00885	LBD41	LOB domain-containing protein 41
	AT4G34370	144	1.97	2.14	0.58	3.40	0.00068	0.00780	ARI1	ARIADNE 1
	AT4G29360	119	1.97	2.11	0.54	3.68	0.00023	0.00349	NA	NA
	AT1G80080	144	1.97	2.16	0.60	3.27	0.00107	0.01080	AtRLP17	Receptor Like Protein 17
	AT5G11240	84	1.97	2.17	0.62	3.20	0.00139	0.01307	NA	NA
	AT4G33980	71	1.97	2.20	0.65	3.01	0.00260	0.02100	NA	NA
	AT3G07650	61	1.97	2.23	0.69	2.87	0.00413	0.02922	BBX7	B-box domain protein 7
	AT2G24990	36	1.97	2.29	0.75	2.64	0.00840	0.04841	NA	NA
	AT1G58290	3395	1.97	2.13	0.57	3.42	0.00062	0.00735	AtHEMA1	Arabidopsis thaliana hemA 1
	AT1G22730	60	1.96	2.19	0.65	3.05	0.00233	0.01932	NA	NA
	AT1G13730	86	1.96	2.18	0.64	3.06	0.00225	0.01881	NA	NA
YES	AT1G12000	745	1.96	2.10	0.52	3.76	0.00017	0.00274	NA	NA
	AT2G21130	59	1.96	2.22	0.69	2.86	0.00429	0.03002	NA	NA
	AT3G20540	84	1.96	2.20	0.66	2.97	0.00297	0.02321	POLGAMM.	polymerase gamma 1
	AT4G32730	54	1.96	2.27	0.73	2.69	0.00718	0.04383	ATMYB3R-1	0
YES	AT5G17920	11371	1.96	2.08	0.51	3.85	0.00012	0.00210	ATCIMS	COBALAMIN-INDEPENDENT METHIONINE SYNTHASE
	AT5G04540	76	1.96	2.15	0.60	3.24	0.00119	0.01171	AtMTM2	0
YES	AT1G20780	166	1.96	2.19	0.65	3.00	0.00271	0.02162	ATPUB44	ARABIDOPSIS THALIANA PLANT U-BOX 44
	AT1G31920	102	1.96	2.20	0.67	2.92	0.00352	0.02620	NA	NA
	AT3G57150	450	1.96	2.08	0.51	3.85	0.00012	0.00209	AtCBF5	0
	AT2G30470	39	1.95	2.21	0.69	2.84	0.00455	0.03136	HSI2	high-level expression of sugar-inducible gene 2
	AT1G26270	188	1.95	2.13	0.59	3.29	0.00099	0.01023	NA	NA
	AT3G50110	64	1.95	2.27	0.74	2.62	0.00871	0.04960	ATPEN3	Arabidopsis thaliana phosphatase and TENsin homolog deleted on chromosome ten 3
	AT5G24470	556	1.95	2.24	0.71	2.74	0.00617	0.03922	APRR5	pseudo-response regulator 5
	AT4G26110	285	1.95	2.09	0.54	3.59	0.00033	0.00456	ATNAP1;1	ARABIDOPSIS THALIANA NUCLEOSOME ASSEMLY PROTEIN 1;1
	AT3G18600	108	1.95	2.17	0.65	3.01	0.00264	0.02122	NA	NA
	AT4G27560	382	1.95	2.07	0.52	3.77	0.00017	0.00271	NA	NA
	AT5G02550	155	1.95	2.13	0.60	3.23	0.00125	0.01203	NA	NA
	AT5G58210	38	1.94	2.22	0.71	2.75	0.00595	0.03821	NA	NA
	AT5G66820	107	1.94	2.17	0.66	2.95	0.00323	0.02459	NA	NA
	AT5G39020	143	1.94	2.15	0.63	3.09	0.00203	0.01743	NA	NA
	AT5G23310	235	1.94	2.06	0.50	3.92	0.00009	0.00168	FSD3	Fe superoxide dismutase 3
	AT4G39140	94	1.94	2.17	0.65	2.97	0.00300	0.02342	NA	NA
	AT5G51100	131	1.94	2.15	0.63	3.07	0.00217	0.01836	FSD2	Fe superoxide dismutase 2
	AT5G14590	72	1.94	2.21	0.71	2.73	0.00633	0.03997	NA	NA
	AT2G38180	57	1.94	2.19	0.69	2.81	0.00502	0.03371	NA	NA
	AT3G12120	2483	1.94	2.08	0.55	3.51	0.00046	0.00580	AtFAD2	0

Supplemental Table 1-RNA seq data comparing Pro35S::MYB63 with wild type.

DAP.MYB63.target(1.5kb upstream TSS)	Gene	baseMean	log2FoldChange	lfcMLE	lfcSE	stat	pvalue	padj (FDR)	TAIR10 Symbol	TAIR10 Annotation (Short)
YES	AT5G49910	1213	1.94	2.09	0.56	3.47	0.00051	0.00637	cpHsc70-2	chloroplast heat shock protein 70-2
	AT1G80830	274	1.94	2.07	0.53	3.66	0.00026	0.00373	ATNRAMP1	0
	AT3G23000	726	1.93	2.10	0.58	3.34	0.00082	0.00895	ATSR2	0
	AT4G30720	157	1.93	2.11	0.59	3.26	0.00111	0.01114	PDE327	PIGMENT DEFECTIVE 327
	AT1G10640	96	1.93	2.23	0.73	2.65	0.00801	0.04707	NA	NA
	AT3G51800	1004	1.93	2.13	0.63	3.05	0.00227	0.01891	ATEBP1	A. THALIANA ERBB-3 BINDING PROTEIN 1
	AT5G55180	114	1.92	2.07	0.54	3.54	0.00041	0.00534	NA	NA
	AT3G19870	181	1.92	2.14	0.66	2.92	0.00356	0.02637	NA	NA
	AT3G18010	30	1.92	2.20	0.72	2.67	0.00765	0.04568	WOX1	WUSCHEL related homeobox 1
	AT5G49580	63	1.92	2.16	0.67	2.85	0.00443	0.03078	NA	NA
	AT2G30990	203	1.92	2.06	0.55	3.50	0.00046	0.00586	NA	NA
	AT2G24180	413	1.92	2.02	0.48	4.01	0.00006	0.00127	CYP71B6	cytochrome p450 71b6
	AT5G08510	39	1.92	2.18	0.70	2.74	0.00615	0.03910	NA	NA
	AT1G31690	66	1.91	2.12	0.63	3.02	0.00254	0.02068	NA	NA
	AT1G06760	1759	1.91	2.05	0.53	3.58	0.00035	0.00477	NA	NA
	AT3G61490	84	1.91	2.12	0.63	3.02	0.00253	0.02060	NA	NA
	AT4G20110	179	1.91	2.04	0.52	3.70	0.00021	0.00330	BP80-3;1	binding protein of 80 kDa 3;1
	AT5G09300	37	1.91	2.20	0.72	2.65	0.00801	0.04707	NA	NA
	AT1G61620	216	1.91	2.09	0.60	3.20	0.00136	0.01287	NA	NA
YES	AT3G16340	51	1.91	2.14	0.67	2.86	0.00430	0.03009	ABCG29	ATP-binding cassette G29
	AT5G62530	159	1.91	2.13	0.66	2.87	0.00407	0.02896	ALDH12A1	aldehyde dehydrogenase 12A1
	AT4G19010	57	1.90	2.18	0.71	2.68	0.00744	0.04479	NA	NA
	AT3G18440	100	1.90	2.14	0.67	2.82	0.00480	0.03265	ALMT9	aluminum-activated malate transporter 9
	AT1G18250	159	1.90	2.05	0.56	3.38	0.00072	0.00813	ATLP-1	0
	AT3G10040	36	1.90	2.19	0.72	2.64	0.00836	0.04828	NA	NA
	AT3G13070	173	1.90	2.03	0.53	3.58	0.00035	0.00478	NA	NA
YES	AT1G03495	39	1.89	2.18	0.72	2.64	0.00832	0.04821	NA	NA
	AT1G21750	476	1.89	2.08	0.61	3.12	0.00184	0.01622	ATPDI5	ARABIDOPSIS THALIANA PROTEIN DISULFIDE ISOMERASE 5
	AT1G55850	241	1.89	2.02	0.53	3.58	0.00034	0.00474	ATCSLE1	0
	AT3G58600	126	1.89	2.11	0.66	2.88	0.00392	0.02815	NA	NA
	AT2G23350	761	1.88	2.00	0.51	3.70	0.00022	0.00335	PAB4	poly(A) binding protein 4
	AT5G14520	197	1.88	2.03	0.56	3.34	0.00083	0.00902	PES	PESCADILLO
	AT1G02330	178	1.88	2.13	0.69	2.72	0.00651	0.04076	NA	NA
	AT4G13660	39	1.88	2.12	0.67	2.79	0.00528	0.03510	ATPRR2	0
	AT5G54630	446	1.88	2.07	0.62	3.01	0.00257	0.02085	NA	NA
	AT5G53000	184	1.87	2.07	0.63	2.95	0.00321	0.02448	TAP46	2A phosphatase associated protein of 46 kD
	AT4G34340	45	1.87	2.12	0.70	2.68	0.00740	0.04464	TAF8	TBP-associated factor 8
	AT5G64580	286	1.87	2.03	0.58	3.21	0.00134	0.01276	EMB3144	EMBRYO DEFECTIVE 3144
	AT1G77130	184	1.86	2.02	0.57	3.25	0.00114	0.01135	GUX3	glucuronic acid substitution of xylan 3
	AT1G19860	123	1.86	2.09	0.67	2.78	0.00550	0.03611	NA	NA
	AT1G12480	47	1.86	2.12	0.70	2.66	0.00777	0.04612	CDI3	CARBON DIOXIDE INSENSITIVE 3
	AT1G21880	133	1.86	2.05	0.62	2.98	0.00284	0.02236	LYM1	lysm domain GPI-anchored protein 1 precursor
	AT2G21520	284	1.86	2.01	0.57	3.23	0.00122	0.01188	NA	NA
	AT1G18485	41	1.85	2.10	0.69	2.69	0.00705	0.04330	NA	NA
	AT2G13840	57	1.85	2.04	0.62	2.96	0.00304	0.02363	NA	NA
	AT3G22660	74	1.85	2.05	0.64	2.91	0.00367	0.02693	NA	NA
	AT5G54510	183	1.85	2.05	0.64	2.90	0.00368	0.02697	DFL1	DWARF IN LIGHT 1
	AT2G33170	167	1.85	2.06	0.65	2.83	0.00472	0.03218	NA	NA
	AT4G13250	810	1.85	1.96	0.50	3.73	0.00019	0.00299	NYC1	NON-YELLOW COLORING 1
	AT5G50280	133	1.85	1.99	0.56	3.32	0.00091	0.00961	EMB1006	embryo defective 1006
	AT4G32070	51	1.85	2.09	0.69	2.67	0.00765	0.04568	Phox4	Phox4
	AT4G29000	47	1.84	2.10	0.70	2.64	0.00824	0.04797	NA	NA
	AT2G17020	83	1.84	2.08	0.67	2.73	0.00625	0.03956	NA	NA
	AT1G52420	160	1.84	2.05	0.65	2.84	0.00453	0.03127	NA	NA
	AT5G46840	98	1.84	2.00	0.57	3.23	0.00122	0.01188	NA	NA
	AT5G56500	139	1.84	1.98	0.55	3.37	0.00074	0.00833	Cpn60beta3	chaperonin-60beta3
	AT5G09220	536	1.84	2.07	0.68	2.72	0.00655	0.04094	AAP2	amino acid permease 2
	AT1G25510	39	1.84	2.08	0.69	2.66	0.00790	0.04662	NA	NA
	AT5G39040	196	1.84	2.01	0.60	3.06	0.00219	0.01850	ABCB27	ATP-binding cassette B27
	AT1G34120	80	1.84	2.06	0.67	2.76	0.00576	0.03728	AT5P1	0
	AT3G13380	54	1.84	2.09	0.70	2.64	0.00833	0.04823	BRL3	BRI1-like 3
	AT4G39030	103	1.83	1.98	0.55	3.31	0.00092	0.00968	EDS5	ENHANCED DISEASE SUSCEPTIBILITY 5
	AT4G33680	1081	1.83	1.92	0.47	3.89	0.00010	0.00184	AGD2	ABERRANT GROWTH AND DEATH 2
	AT3G18770	97	1.82	2.04	0.66	2.78	0.00550	0.03612	NA	NA

Supplemental Table 1-RNA seq data comparing Pro35S::MYB63 with wild type.

DAP.MYB63.target(1.5kb upstream TSS)	Gene	baseMean	log2FoldChange	lfcMLE	lfcSE	stat	pvalue	padj (FDR)	TAIR10 Symbol	TAIR10 Annotation (Short)
	AT1G28050	89	1.82	2.01	0.63	2.91	0.00361	0.02661	BBX13	B-box domain protein 13
YES	AT4G28450	117	1.82	2.01	0.62	2.95	0.00318	0.02435	NA	NA
YES	AT1G19680	76	1.82	2.04	0.66	2.76	0.00572	0.03711	NA	NA
	AT5G66910	160	1.82	1.95	0.53	3.42	0.00062	0.00731	NA	NA
	AT1G17140	121	1.82	2.01	0.62	2.93	0.00343	0.02573	ICR1	interactor of constitutive active rops 1
	AT1G15670	218	1.82	2.00	0.61	2.97	0.00298	0.02326	KFB01	Kelch repeat F-box 1
	AT5G25930	150	1.81	2.00	0.62	2.91	0.00360	0.02660	NA	NA
	AT2G02220	376	1.81	1.92	0.50	3.66	0.00025	0.00368	ATPSKR1	PHYTOSULFOKIN RECEPTOR 1
	AT2G05440	1987	1.81	1.94	0.53	3.44	0.00058	0.00696	ATGRP9	ARABIDOPSIS GLYCINE RICH PROTEIN 9
	AT1G01260	99	1.81	2.00	0.63	2.89	0.00384	0.02771	JAM2	Jasmonate Associated MYC2 LIKE 2
	AT3G02120	65	1.81	2.03	0.67	2.71	0.00677	0.04197	NA	NA
	AT1G06390	343	1.80	1.92	0.51	3.53	0.00042	0.00549	ATGSK1	0
	AT1G11820	192	1.80	1.96	0.58	3.12	0.00179	0.01591	NA	NA
YES	AT3G54820	338	1.80	1.94	0.55	3.25	0.00116	0.01143	PIP2;5	plasma membrane intrinsic protein 2;5
	AT1G45000	555	1.80	1.90	0.49	3.69	0.00023	0.00346	NA	NA
	AT5G14270	112	1.79	2.01	0.66	2.71	0.00682	0.04224	ATBET9	bromodomain and extraterminal domain protein 9
	AT1G31817	150	1.79	1.97	0.61	2.94	0.00330	0.02502	NFD3	NUCLEAR FUSION DEFECTIVE 3
	AT3G07930	76	1.79	1.97	0.61	2.93	0.00341	0.02562	NA	NA
	AT5G02620	116	1.79	1.96	0.59	3.02	0.00254	0.02064	ANK1	ankyrin-like1
	AT1G76270	39	1.79	2.01	0.67	2.65	0.00803	0.04715	NA	NA
	AT1G60140	389	1.79	1.91	0.53	3.40	0.00068	0.00783	ATTPS10	trehalose phosphate synthase
	AT5G20890	378	1.79	1.90	0.52	3.45	0.00056	0.00677	NA	NA
	AT5G23240	137	1.78	1.95	0.60	2.98	0.00285	0.02242	DJC76	DNA J protein C76
	AT1G14900	208	1.78	1.91	0.53	3.37	0.00074	0.00833	HMGA	high mobility group A
	AT2G17480	135	1.78	2.00	0.66	2.69	0.00704	0.04326	ATMLO8	MILDEW RESISTANCE LOCUS O 8
	AT1G67850	85	1.78	1.99	0.65	2.72	0.00654	0.04089	NA	NA
	AT5G22020	226	1.78	1.96	0.62	2.87	0.00410	0.02911	NA	NA
	AT5G28500	531	1.78	1.90	0.52	3.39	0.00070	0.00795	NA	NA
YES	AT3G05290	357	1.78	1.91	0.55	3.23	0.00123	0.01195	AtPNC1	0
	AT3G05060	200	1.78	1.91	0.55	3.20	0.00136	0.01287	NA	NA
	AT3G20250	124	1.77	1.92	0.57	3.09	0.00199	0.01717	APUM5	pumilio 5
	AT4G14580	107	1.77	1.93	0.59	3.03	0.00249	0.02042	CIPK4	CBL-interacting protein kinase 4
	AT5G15730	132	1.77	1.95	0.61	2.88	0.00395	0.02829	AtCRLK2	0
	AT1G79550	2090	1.77	1.87	0.48	3.69	0.00023	0.00344	PGK	phosphoglycerate kinase
	AT5G65950	247	1.77	1.88	0.52	3.42	0.00063	0.00736	NA	NA
	AT3G11830	369	1.76	1.87	0.49	3.57	0.00036	0.00491	NA	NA
	AT1G15470	114	1.76	1.91	0.57	3.09	0.00198	0.01707	NA	NA
	AT3G63250	252	1.76	1.89	0.53	3.30	0.00097	0.01001	ATHMT-2	HOMOCYSTEINE METHYLTRANSFERASE-2
	AT2G38960	53	1.76	1.97	0.66	2.67	0.00767	0.04575	AERO2	endoplasmic reticulum oxidoreductins 2
	AT5G63570	655	1.76	1.87	0.51	3.45	0.00057	0.00683	GSA1	glutamate-1-semialdehyde-2,1-aminomutase
	AT3G18190	211	1.76	1.89	0.55	3.18	0.00147	0.01366	NA	NA
	AT5G64420	137	1.76	1.96	0.65	2.68	0.00728	0.04414	NA	NA
	AT3G25230	248	1.76	1.94	0.63	2.79	0.00530	0.03522	ATFKBP62	0
	AT5G41000	66	1.76	1.92	0.60	2.94	0.00327	0.02483	AtYSL4	0
	AT4G34500	61	1.76	1.93	0.61	2.89	0.00385	0.02778	NA	NA
YES	AT1G48320	607	1.75	1.88	0.53	3.30	0.00095	0.00990	DHNAT1	DHNA-CoA thioesterase 1
	AT2G47470	949	1.75	1.91	0.59	2.99	0.00282	0.02227	ATPDI11	ARABIDOPSIS THALIANA PROTEIN DISULFIDE ISOMERASE 11
	AT2G43180	72	1.75	1.92	0.61	2.87	0.00406	0.02891	NA	NA
	AT3G16180	197	1.75	1.88	0.54	3.24	0.00119	0.01169	NRT1.12	nitrate transporter 1.12
	AT1G28110	108	1.75	1.90	0.57	3.06	0.00225	0.01882	SCPL45	serine carboxypeptidase-like 45
	AT4G01037	196	1.75	1.87	0.52	3.34	0.00084	0.00912	AtWTF1	0
	AT3G17410	424	1.75	1.90	0.58	3.04	0.00239	0.01975	NA	NA
	AT5G13320	159	1.74	1.93	0.63	2.78	0.00544	0.03582	AtGH3.12	0
	AT5G10200	43	1.74	1.96	0.66	2.63	0.00864	0.04934	NA	NA
	AT3G12570	108	1.74	1.93	0.63	2.78	0.00535	0.03544	FYD	0
	AT1G48920	1485	1.74	1.86	0.51	3.39	0.00071	0.00805	ATNUC-L1	nucleolin like 1
	AT1G69420	226	1.74	1.85	0.51	3.40	0.00068	0.00783	NA	NA
	AT5G50320	223	1.74	1.88	0.55	3.14	0.00170	0.01532	AtELP3	Elongator protein 3
	AT2G18850	57	1.74	1.94	0.65	2.68	0.00739	0.04459	NA	NA
	AT1G32780	91	1.74	1.90	0.59	2.95	0.00316	0.02426	NA	NA
	AT1G10070	145	1.74	1.90	0.59	2.92	0.00345	0.02580	ATBCAT-2	branched-chain amino acid transaminase 2
YES	AT1G14180	79	1.74	1.94	0.65	2.66	0.00783	0.04633	NA	NA

Supplemental Table 1-RNA seq data comparing Pro35S::MYB63 with wild type.

DAP.MYB63.target(1.5kb upstream TSS)	Gene	baseMean	log2FoldChange	lfcMLE	lfcSE	stat	pvalue	padj (FDR)	TAIR10 Symbol	TAIR10 Annotation (Short)
	AT3G22440	476	1.74	1.94	0.65	2.66	0.00790	0.04662	NA	NA
	AT4G35580	73	1.74	1.94	0.65	2.67	0.00764	0.04568	CBNAC	calmodulin-binding NAC protein
	AT3G55450	318	1.73	1.86	0.54	3.19	0.00144	0.01345	PBL1	PBS1-like 1
YES	AT1G21270	679	1.73	1.87	0.56	3.11	0.00188	0.01645	WAK2	wall-associated kinase 2
YES	AT1G03870	1264	1.73	1.87	0.56	3.09	0.00200	0.01720	FLA9	FASCICLIN-like arabinoogalactan 9
	AT3G13030	99	1.73	1.89	0.60	2.88	0.00397	0.02837	NA	NA
	AT1G54270	1322	1.73	1.89	0.60	2.90	0.00374	0.02721	EIF4A-2	eif4a-2
YES	AT5G10740	75	1.73	1.88	0.59	2.90	0.00369	0.02699	NA	NA
	AT5G18070	58	1.72	1.92	0.65	2.65	0.00812	0.04751	DRT101	DNA-DAMAGE-REPAIR/TOLERATION 101
	AT4G37520	213	1.72	1.85	0.55	3.12	0.00179	0.01591	NA	NA
	AT3G56720	104	1.72	1.91	0.64	2.68	0.00726	0.04406	NA	NA
	AT2G16780	77	1.72	1.87	0.58	2.94	0.00325	0.02468	MSI02	0
	AT4G11860	151	1.72	1.83	0.52	3.33	0.00088	0.00941	NA	NA
	AT5G62550	57	1.72	1.92	0.66	2.62	0.00880	0.04990	NA	NA
	AT4G30200	331	1.72	1.87	0.58	2.94	0.00324	0.02462	VEL1	vernalization5/VIN3-like 1
	AT2G40090	74	1.72	1.88	0.60	2.86	0.00426	0.02994	ATATH9	ABC2 homolog 9
	AT4G06598	54	1.71	1.90	0.64	2.68	0.00733	0.04434	NA	NA
	AT3G25010	116	1.71	1.90	0.64	2.66	0.00788	0.04658	AtRLP41	receptor like protein 41
	AT5G51430	176	1.71	1.87	0.60	2.86	0.00419	0.02958	EYE	EMBRYO YELLOW
YES	AT3G57290	614	1.71	1.83	0.53	3.23	0.00124	0.01199	ATEIF3E-1	0
	AT1G73980	177	1.71	1.87	0.60	2.85	0.00443	0.03073	NA	NA
	AT1G23030	196	1.71	1.82	0.52	3.30	0.00096	0.00993	NA	NA
	AT3G15980	186	1.70	1.90	0.65	2.63	0.00865	0.04938	NA	NA
	AT3G25150	111	1.70	1.88	0.63	2.70	0.00693	0.04270	NA	NA
YES	AT1G49580	114	1.70	1.83	0.55	3.06	0.00218	0.01842	NA	NA
	AT4G24290	294	1.70	1.82	0.53	3.23	0.00122	0.01193	NA	NA
	AT5G07710	76	1.70	1.86	0.60	2.82	0.00480	0.03265	NA	NA
	AT4G21540	152	1.70	1.85	0.60	2.84	0.00457	0.03148	SPHK1	sphingosine kinase 1
	AT3G57260	1158	1.69	1.80	0.50	3.40	0.00067	0.00774	AtBG2	0
	AT4G24530	63	1.69	1.85	0.60	2.83	0.00470	0.03215	NA	NA
	AT5G04885	164	1.69	1.84	0.58	2.92	0.00353	0.02620	NA	NA
	AT3G12080	188	1.69	1.87	0.62	2.71	0.00676	0.04195	emb2738	embryo defective 2738
	AT2G41370	94	1.69	1.87	0.63	2.66	0.00770	0.04587	BOP2	BLADE ON PETIOLE2
	AT2G21385	367	1.69	1.79	0.48	3.49	0.00049	0.00611	NA	NA
	AT3G59110	132	1.69	1.87	0.63	2.66	0.00771	0.04587	NA	NA
	AT1G64740	175	1.69	1.82	0.55	3.07	0.00211	0.01793	TUA1	alpha-1 tubulin
	AT3G58570	142	1.69	1.81	0.54	3.11	0.00188	0.01647	NA	NA
	AT2G44760	91	1.69	1.87	0.64	2.64	0.00820	0.04782	NA	NA
	AT4G00650	52	1.69	1.86	0.63	2.69	0.00712	0.04355	FLA	FLOWERING LOCUS A
	AT4G17720	243	1.69	1.82	0.56	3.03	0.00245	0.02021	NA	NA
	AT5G22040	82	1.69	1.87	0.63	2.67	0.00769	0.04585	NA	NA
	AT3G06930	187	1.68	1.79	0.52	3.26	0.00111	0.01113	ATPRMT4B	ARABIDOPSIS THALIANA PROTEIN ARGININE METHYLTRANSFERASE 4B
	AT4G02230	787	1.68	1.86	0.64	2.62	0.00878	0.04987	NA	NA
	AT5G63710	117	1.68	1.84	0.61	2.74	0.00612	0.03897	NA	NA
	AT4G30780	77	1.68	1.84	0.61	2.75	0.00603	0.03853	NA	NA
	AT3G53110	219	1.67	1.80	0.54	3.11	0.00186	0.01638	LOS4	LOW EXPRESSION OF OSMOTICALLY RESPONSIVE GENES 4
	AT5G56630	190	1.67	1.79	0.52	3.20	0.00139	0.01305	PFK7	phosphofructokinase 7
	AT5G37600	1597	1.67	1.76	0.48	3.48	0.00051	0.00635	ATGLN1;1	ARABIDOPSIS GLUTAMINE SYNTHASE 1;1
	AT1G05350	118	1.67	1.84	0.62	2.67	0.00755	0.04520	NA	NA
	AT3G51850	214	1.66	1.82	0.61	2.74	0.00619	0.03928	CPK13	calcium-dependent protein kinase 13
	AT3G29180	207	1.66	1.78	0.54	3.08	0.00208	0.01777	NA	NA
	AT1G67440	93	1.66	1.81	0.58	2.84	0.00450	0.03114	emb1688	embryo defective 1688
	AT1G73720	123	1.66	1.82	0.60	2.75	0.00603	0.03853	SMU1	SUPPRESSORS OF MEC-8 AND UNC-52 1
	AT5G62700	561	1.66	1.83	0.62	2.66	0.00774	0.04599	TUB3	tubulin beta chain 3
	AT1G19700	148	1.66	1.80	0.58	2.85	0.00436	0.03039	BEL10	BEL1-like homeodomain 10
	AT4G00340	193	1.65	1.81	0.61	2.73	0.00628	0.03970	RLK4	receptor-like protein kinase 4
	AT5G24060	122	1.65	1.81	0.60	2.75	0.00604	0.03857	NA	NA
	AT1G21400	324	1.65	1.75	0.50	3.31	0.00092	0.00968	NA	NA
	AT2G28100	56	1.65	1.82	0.62	2.67	0.00749	0.04496	ATFUC1	alpha-L-fucosidase 1
	AT3G03310	170	1.65	1.77	0.53	3.10	0.00190	0.01658	ATLCAT3	ARABIDOPSIS LECITHIN:CHOLESTEROL ACYLTRANSFERASE 3
	AT3G51950	751	1.65	1.74	0.49	3.38	0.00073	0.00818	NA	NA
	AT1G74850	181	1.65	1.80	0.59	2.78	0.00543	0.03581	PDE343	PIGMENT DEFECTIVE 343
	AT1G49760	458	1.64	1.74	0.50	3.29	0.00101	0.01034	PAB8	poly(A) binding protein 8

Supplemental Table 1-RNA seq data comparing Pro35S::MYB63 with wild type.

DAP MYB63.target(1.5kb upstream TSS)	Gene	baseMean	log2FoldChange	lfcMLE	lfcSE	stat	pvalue	padj (FDR)	TAIR10 Symbol	TAIR10 Annotation (Short)
	AT3G20560	137	1.64	1.79	0.60	2.75	0.00600	0.03845	ATPDI12	ARABIDOPSIS THALIANA PROTEIN DISULFIDE ISOMERASE 12
	AT2G26330	334	1.64	1.80	0.62	2.65	0.00806	0.04724	ER	ERECTA
	AT3G22330	216	1.64	1.74	0.51	3.20	0.00139	0.01306	ATRH53	0
	AT3G03740	144	1.64	1.76	0.55	2.95	0.00316	0.02426	ATBPM4	BTB-POZ AND MATH DOMAIN 4
	AT3G63270	69	1.64	1.79	0.60	2.71	0.00671	0.04170	NA	NA
	AT3G49880	74	1.63	1.80	0.62	2.64	0.00838	0.04836	NA	NA
	AT1G47290	135	1.63	1.76	0.55	2.95	0.00321	0.02448	3BETAHSD/	3beta-hydroxysteroid-dehydrogenase/decarboxylase isoform 1
	AT2G25920	108	1.63	1.78	0.59	2.79	0.00533	0.03534	NA	NA
	AT4G14990	151	1.63	1.76	0.55	2.98	0.00292	0.02291	NA	NA
	AT3G52210	91	1.63	1.77	0.58	2.83	0.00462	0.03171	NA	NA
	AT5G64560	85	1.63	1.79	0.61	2.68	0.00738	0.04455	ATMGT9	0
	AT5G42720	162	1.63	1.79	0.62	2.64	0.00826	0.04801	NA	NA
	AT5G56660	321	1.63	1.73	0.51	3.19	0.00140	0.01313	ILL2	IAA-leucine resistant (ILR)-like 2
	AT1G75370	354	1.63	1.73	0.50	3.27	0.00106	0.01076	NA	NA
	AT2G03220	135	1.63	1.75	0.55	2.95	0.00318	0.02435	ATFT1	ARABIDOPSIS THALIANA FUCOSYLTRANSFERASE 1
	AT5G39990	244	1.63	1.78	0.60	2.71	0.00667	0.04153	GlcAT14A	beta-glucuronosyltransferase 14A
	AT4G03260	354	1.62	1.78	0.61	2.66	0.00781	0.04627	NA	NA
	AT1G25580	89	1.62	1.78	0.60	2.70	0.00686	0.04240	ANAC008	Arabidopsis NAC domain containing protein 8
	AT5G26742	2837	1.62	1.75	0.56	2.88	0.00396	0.02834	AtRH3	0
	AT1G74710	236	1.62	1.73	0.53	3.07	0.00215	0.01820	ATICS1	ARABIDOPSIS ISOCHORISMATE SYNTHASE 1
	AT4G35830	1625	1.62	1.76	0.59	2.75	0.00594	0.03817	ACO1	aconitase 1
	AT3G07140	89	1.61	1.76	0.59	2.74	0.00622	0.03944	NA	NA
	AT5G11760	228	1.61	1.77	0.61	2.66	0.00775	0.04604	NA	NA
	AT5G19350	170	1.61	1.74	0.55	2.92	0.00352	0.02616	NA	NA
	AT4G17080	258	1.61	1.74	0.55	2.92	0.00350	0.02608	NA	NA
	AT1G80480	231	1.61	1.74	0.57	2.82	0.00475	0.03241	PTAC17	plastid transcriptionally active 17
	AT2G29550	329	1.61	1.72	0.52	3.08	0.00205	0.01754	TUB7	tubulin beta-7 chain
	AT5G06050	63	1.61	1.76	0.60	2.68	0.00744	0.04479	NA	NA
	AT2G47610	1363	1.61	1.70	0.48	3.33	0.00087	0.00934	NA	NA
	AT1G59900	477	1.61	1.73	0.54	2.95	0.00314	0.02418	AT-E1 ALPH	pyruvate dehydrogenase complex E1 alpha subunit
	AT1G20010	1593	1.60	1.70	0.50	3.20	0.00136	0.01286	TUB5	tubulin beta-5 chain
	AT2G22360	173	1.60	1.71	0.52	3.06	0.00223	0.01870	DJA6	DNA J protein A6
	AT2G04450	153	1.60	1.72	0.55	2.93	0.00340	0.02556	ATNUDT6	nudix hydrolase homolog 6
	AT4G37920	148	1.60	1.73	0.56	2.86	0.00430	0.03009	NA	NA
	AT3G13230	193	1.60	1.73	0.57	2.80	0.00512	0.03421	NA	NA
	AT1G02780	2928	1.60	1.72	0.54	2.96	0.00307	0.02377	emb2386	embryo defective 2386
	AT2G33620	107	1.60	1.72	0.55	2.90	0.00374	0.02721	AHL10	AT-hook motif nuclear localized protein 10
	AT5G02270	261	1.59	1.74	0.59	2.71	0.00664	0.04135	ABCI20	ATP-binding cassette I20
YES	AT4G32400	201	1.59	1.71	0.56	2.86	0.00428	0.02999	ATBT1	ARABIDOPSIS THALIANA BRITTLE 1
	AT3G58970	123	1.58	1.71	0.57	2.80	0.00507	0.03395	MGT6	magnesium transporter 6
YES	AT3G10840	105	1.58	1.73	0.59	2.67	0.00750	0.04504	NA	NA
	AT3G19000	186	1.58	1.71	0.57	2.76	0.00571	0.03710	NA	NA
	AT1G21670	448	1.58	1.72	0.58	2.72	0.00644	0.04038	NA	NA
	AT3G60300	212	1.58	1.71	0.58	2.74	0.00614	0.03903	NA	NA
	AT1G13470	389	1.57	1.66	0.49	3.23	0.00124	0.01203	NA	NA
	AT4G35600	131	1.57	1.70	0.57	2.75	0.00603	0.03853	CST	CAST AWAY
	AT2G35190	95	1.57	1.70	0.58	2.70	0.00702	0.04315	ATNPSN11	0
	AT5G63040	119	1.56	1.69	0.57	2.75	0.00589	0.03796	NA	NA
	AT1G72500	169	1.56	1.68	0.54	2.91	0.00366	0.02692	NA	NA
	AT3G62870	1703	1.56	1.68	0.54	2.90	0.00378	0.02747	NA	NA
	AT5G35430	154	1.56	1.68	0.54	2.89	0.00383	0.02768	NA	NA
	AT3G60190	638	1.56	1.68	0.55	2.86	0.00427	0.02999	ADL1E	DYNAMIN-like 1E
	AT3G44990	369	1.56	1.68	0.55	2.82	0.00483	0.03278	AtXTH31	0
	AT2G01910	155	1.56	1.70	0.59	2.65	0.00800	0.04707	ATMAP65-6	0
	AT4G31810	127	1.56	1.68	0.55	2.84	0.00455	0.03137	NA	NA
	AT5G03160	196	1.56	1.66	0.51	3.05	0.00231	0.01921	ATP58IPK	homolog of mamallian P58IPK
	AT2G17220	215	1.56	1.66	0.51	3.05	0.00230	0.01915	Kin3	kinase 3
	AT1G11790	573	1.56	1.70	0.59	2.65	0.00801	0.04707	ADT1	arogenate dehydratase 1
	AT2G45510	81	1.56	1.69	0.58	2.66	0.00774	0.04599	CYP704A2	cytochrome P450, family 704, subfamily A, polypeptide 2
	AT2G19470	103	1.56	1.68	0.56	2.78	0.00539	0.03562	ckl5	casein kinase I-like 5
	AT5G23540	521	1.56	1.65	0.49	3.16	0.00160	0.01462	NA	NA
	AT5G03740	254	1.55	1.67	0.54	2.86	0.00428	0.02999	HD2C	histone deacetylase 2C

Supplemental Table 1-RNA seq data comparing Pro35S::MYB63 with wild type.

DAP.MYB63.target(1.5kb upstream TSS)	Gene	baseMean	log2FoldChange	lfcMLE	lfcSE	stat	pvalue	padj (FDR)	TAIR10 Symbol	TAIR10 Annotation (Short)
	AT1G73950	96	1.55	1.69	0.58	2.66	0.00780	0.04620	NA	NA
YES	AT3G17390	6238	1.55	1.63	0.47	3.32	0.00090	0.00953	MAT4	METHIONINE ADENOSYLTRANSFERASE 4
	AT2G41900	154	1.55	1.68	0.58	2.69	0.00715	0.04368	OXS2	OXIDATIVE STRESS 2
	AT1G68020	133	1.55	1.68	0.59	2.64	0.00828	0.04808	ATTPS6	0
	AT2G19450	94	1.55	1.69	0.59	2.62	0.00878	0.04987	ABX45	0
	AT3G15355	133	1.55	1.66	0.55	2.82	0.00483	0.03278	PFU1	PHO2 FAMILY UBIQUITIN CONJUGATION ENZYME 1
	AT4G32610	177	1.54	1.67	0.58	2.66	0.00773	0.04599	NA	NA
	AT1G21380	173	1.54	1.66	0.55	2.80	0.00514	0.03437	NA	NA
	AT1G19170	123	1.54	1.65	0.55	2.80	0.00517	0.03453	NA	NA
	AT5G61000	106	1.54	1.66	0.57	2.70	0.00692	0.04268	ATRPA70D	0
	AT3G12560	132	1.53	1.67	0.58	2.64	0.00820	0.04782	ATTBP2	TELOMERIC DNA-BINDING PROTEIN 2
	AT3G56580	97	1.53	1.66	0.57	2.69	0.00725	0.04404	AtRZF1	0
	AT4G31820	181	1.53	1.65	0.55	2.78	0.00543	0.03580	ENP	ENHANCER OF PINOID
	AT1G23180	166	1.53	1.67	0.58	2.63	0.00847	0.04865	NA	NA
	AT1G21680	695	1.53	1.64	0.53	2.89	0.00387	0.02783	NA	NA
	AT3G53130	261	1.53	1.63	0.51	3.02	0.00250	0.02049	CYP97C1	CYTOCHROME P450 97C1
	AT5G03940	323	1.53	1.66	0.57	2.68	0.00736	0.04447	54CP	54 CHLOROPLAST PROTEIN
	AT3G48200	254	1.53	1.64	0.53	2.87	0.00413	0.02922	NA	NA
	AT2G31660	251	1.53	1.64	0.54	2.85	0.00438	0.03049	EMA1	enhanced miRNA activity 1
	AT4G08690	146	1.51	1.63	0.55	2.78	0.00547	0.03597	NA	NA
	AT1G63680	139	1.51	1.63	0.55	2.77	0.00553	0.03626	APG13	ALBINO OR PALE-GREEN 13
	AT4G38430	192	1.51	1.63	0.56	2.72	0.00660	0.04112	ATROPGEF1	0
	AT3G03950	191	1.51	1.61	0.52	2.89	0.00379	0.02751	ECT1	evolutionarily conserved C-terminal region 1
YES	AT1G65930	2531	1.51	1.59	0.48	3.14	0.00169	0.01523	cICDH	cytosolic NADP+-dependent isocitrate dehydrogenase
	AT1G73170	213	1.51	1.62	0.54	2.76	0.00571	0.03710	NA	NA
	AT1G12120	354	1.50	1.60	0.50	3.01	0.00260	0.02100	NA	NA
YES	AT1G28610	379	1.50	1.62	0.55	2.73	0.00639	0.04022	NA	NA
	AT1G04870	164	1.50	1.61	0.54	2.81	0.00503	0.03374	ATPRMT10	0
	AT1G34300	145	1.50	1.62	0.55	2.75	0.00592	0.03805	NA	NA
	AT1G23290	8352	-1.50	-1.60	0.51	-2.95	0.00322	0.02458	RPL27A	RIBOSOMAL PROTEIN L27A
	AT3G56810	154	-1.50	-1.62	0.56	-2.69	0.00721	0.04392	NA	NA
	AT2G03690	604	-1.50	-1.61	0.54	-2.81	0.00502	0.03372	NA	NA
	AT5G55990	747	-1.51	-1.63	0.56	-2.69	0.00715	0.04368	ATCBL2	0
	AT1G12310	2097	-1.51	-1.62	0.54	-2.78	0.00540	0.03566	NA	NA
	AT5G06980	1134	-1.51	-1.61	0.53	-2.84	0.00456	0.03141	LNK4	night light-inducible and clock-regulated 4
	AT1G11475	1332	-1.51	-1.62	0.55	-2.72	0.00657	0.04105	NRPB10	0
	AT1G74970	7103	-1.51	-1.60	0.51	-2.93	0.00334	0.02522	RPS9	ribosomal protein S9
	AT5G67300	3883	-1.51	-1.61	0.52	-2.92	0.00345	0.02585	ATMYB44	ARABIDOPSIS THALIANA MYB DOMAIN PROTEIN 44
	AT4G39780	479	-1.51	-1.62	0.54	-2.78	0.00544	0.03582	NA	NA
	AT5G20165	1098	-1.51	-1.62	0.54	-2.79	0.00529	0.03514	NA	NA
	AT1G01990	762	-1.51	-1.62	0.53	-2.83	0.00460	0.03162	NA	NA
	AT3G07460	2176	-1.51	-1.63	0.55	-2.78	0.00549	0.03606	NA	NA
	AT5G50100	271	-1.51	-1.62	0.53	-2.85	0.00441	0.03063	NA	NA
	AT2G18740	999	-1.52	-1.63	0.55	-2.75	0.00596	0.03828	NA	NA
	AT4G35770	1469	-1.52	-1.65	0.58	-2.64	0.00837	0.04830	ATSEN1	ARABIDOPSIS THALIANA SENESCENCE 1
	AT1G24350	593	-1.52	-1.64	0.55	-2.75	0.00597	0.03828	NA	NA
	AT2G27720	11335	-1.52	-1.63	0.55	-2.77	0.00553	0.03627	NA	NA
	AT1G12840	3254	-1.52	-1.62	0.52	-2.90	0.00374	0.02721	ATVHA-C	ARABIDOPSIS THALIANA VACUOLAR ATP SYNTHASE SUBUNIT C
	AT4G28190	149	-1.52	-1.65	0.58	-2.64	0.00825	0.04801	ULT	ULTRAPETALA
	AT1G78620	1923	-1.52	-1.63	0.54	-2.82	0.00480	0.03265	NA	NA
	AT4G26860	1077	-1.52	-1.65	0.57	-2.69	0.00721	0.04392	NA	NA
	AT2G33810	433	-1.52	-1.63	0.54	-2.81	0.00489	0.03311	SPL3	squamosa promoter binding protein-like 3
	AT5G65730	2721	-1.52	-1.62	0.50	-3.02	0.00256	0.02074	XTH6	xyloglucan endotransglucosylase/hydrolase 6
	AT5G35732	221	-1.52	-1.64	0.55	-2.77	0.00560	0.03654	NA	NA
	AT2G37920	516	-1.52	-1.64	0.54	-2.83	0.00464	0.03179	emb1513	embryo defective 1513
YES	AT3G06310	883	-1.53	-1.64	0.55	-2.79	0.00530	0.03522	NA	NA
	AT2G33740	740	-1.53	-1.62	0.51	-3.02	0.00255	0.02072	CUTA	0
	AT2G44740	823	-1.53	-1.64	0.53	-2.88	0.00395	0.02829	CYCP4;1	cyclin p4;1
	AT3G01740	772	-1.53	-1.64	0.54	-2.85	0.00433	0.03023	NA	NA
	AT3G17100	783	-1.53	-1.63	0.52	-2.96	0.00306	0.02370	AIF3	ATBS1 Interacting Factor 3

Supplemental Table 1-RNA seq data comparing Pro35S::MYB63 with wild type.

DAP.MYB63.target(1.5kb upstream TSS)	Gene	baseMean	log2FoldChange	lfcMLE	lfcSE	stat	pvalue	padj (FDR)	TAIR10 Symbol	TAIR10 Annotation (Short)
	AT5G47190	5773	-1.53	-1.65	0.55	-2.79	0.00523	0.03485	NA	NA
	AT1G13930	16926	-1.53	-1.65	0.54	-2.82	0.00480	0.03265	NA	NA
	AT1G79160	299	-1.53	-1.64	0.52	-2.94	0.00328	0.02483	NA	NA
	AT5G10570	104	-1.53	-1.66	0.58	-2.65	0.00795	0.04685	NA	NA
	AT2G46580	414	-1.53	-1.66	0.58	-2.66	0.00790	0.04662	NA	NA
	AT2G06850	6712	-1.53	-1.64	0.53	-2.91	0.00356	0.02640	EXGT-A1	endoxyloglucan transferase A1
	AT3G07200	274	-1.53	-1.65	0.54	-2.84	0.00456	0.03139	NA	NA
	AT4G18230	381	-1.54	-1.65	0.54	-2.85	0.00437	0.03044	NA	NA
	AT5G41920	269	-1.54	-1.67	0.57	-2.68	0.00730	0.04425	NA	NA
	AT2G39705	681	-1.54	-1.67	0.58	-2.65	0.00811	0.04746	DVL11	DEVIL 11
	AT1G29660	2420	-1.54	-1.63	0.49	-3.15	0.00165	0.01494	NA	NA
	AT1G04340	434	-1.54	-1.67	0.58	-2.66	0.00788	0.04658	NA	NA
	AT5G52750	1784	-1.54	-1.67	0.58	-2.67	0.00753	0.04516	NA	NA
	AT4G02530	5896	-1.54	-1.65	0.53	-2.90	0.00370	0.02704	NA	NA
	AT5G13560	406	-1.54	-1.67	0.58	-2.67	0.00767	0.04575	NA	NA
	AT1G55910	575	-1.54	-1.65	0.54	-2.87	0.00416	0.02942	ZIP11	zinc transporter 11 precursor
	AT4G31985	8421	-1.54	-1.67	0.56	-2.74	0.00621	0.03936	NA	NA
	AT5G11280	706	-1.54	-1.66	0.54	-2.85	0.00438	0.03049	NA	NA
	AT3G19030	3752	-1.55	-1.68	0.58	-2.66	0.00776	0.04604	NA	NA
	AT1G15820	154753	-1.55	-1.66	0.54	-2.86	0.00428	0.02999	CP24	0
	AT1G64065	311	-1.55	-1.68	0.58	-2.66	0.00776	0.04608	NA	NA
	AT5G49210	869	-1.55	-1.68	0.57	-2.72	0.00652	0.04080	NA	NA
	AT5G41910	213	-1.55	-1.66	0.54	-2.85	0.00433	0.03023	MED10A	0
YES	AT5G42060	319	-1.55	-1.67	0.56	-2.77	0.00559	0.03654	NA	NA
	AT1G67280	3899	-1.55	-1.69	0.58	-2.66	0.00790	0.04662	NA	NA
	AT5G43850	2212	-1.55	-1.65	0.51	-3.01	0.00259	0.02097	ARD4	0
	AT5G27390	1738	-1.55	-1.68	0.58	-2.67	0.00749	0.04498	NA	NA
	AT4G00165	1810	-1.55	-1.67	0.55	-2.80	0.00505	0.03381	NA	NA
	AT5G41600	1432	-1.55	-1.67	0.54	-2.88	0.00400	0.02854	BTI3	VIRB2-interacting protein 3
	AT5G51110	3717	-1.55	-1.64	0.47	-3.30	0.00097	0.01001	NA	NA
	AT3G26520	35903	-1.55	-1.67	0.54	-2.88	0.00394	0.02824	GAMMA-TI	0
	AT5G06790	244	-1.55	-1.66	0.53	-2.95	0.00313	0.02413	NA	NA
	AT5G48335	874	-1.56	-1.68	0.56	-2.77	0.00554	0.03628	NA	NA
	AT1G65845	828	-1.56	-1.66	0.51	-3.06	0.00224	0.01879	NA	NA
	AT5G47370	291	-1.56	-1.67	0.53	-2.93	0.00344	0.02579	HAT2	0
YES	AT3G22600	886	-1.56	-1.67	0.54	-2.90	0.00367	0.02693	LTPG5	glycosylphosphatidylinositol-anchored lipid protein transfer 5
	AT5G54750	956	-1.56	-1.70	0.59	-2.63	0.00861	0.04920	NA	NA
	AT5G27770	6218	-1.56	-1.66	0.52	-3.01	0.00265	0.02130	NA	NA
	AT2G24590	588	-1.56	-1.68	0.56	-2.79	0.00532	0.03528	At-RSZ22a	RS-containing zinc finger protein 22a
	AT3G15640	1757	-1.56	-1.67	0.53	-2.96	0.00306	0.02370	NA	NA
	AT5G46160	395	-1.56	-1.69	0.58	-2.70	0.00701	0.04312	NA	NA
	AT5G67070	2410	-1.56	-1.67	0.52	-2.99	0.00277	0.02193	RALFL34	ralf-like 34
	AT5G57685	320	-1.56	-1.70	0.58	-2.67	0.00763	0.04560	ATGDU3	ARABIDOPSIS THALIANA GLUTAMINE DUMPER 3
	AT3G52760	752	-1.56	-1.67	0.52	-2.98	0.00293	0.02295	NA	NA
	AT4G34700	1922	-1.56	-1.67	0.52	-3.01	0.00260	0.02097	AtCIB22	0
	AT1G68490	1278	-1.56	-1.67	0.53	-2.95	0.00318	0.02435	NA	NA
	AT5G61220	545	-1.56	-1.68	0.54	-2.90	0.00372	0.02718	NA	NA
	AT3G50920	344	-1.56	-1.71	0.59	-2.63	0.00861	0.04919	LPPepsilon1	lipid phosphate phosphatase epsilon 1
	AT1G03730	336	-1.56	-1.69	0.56	-2.78	0.00546	0.03591	NA	NA
	AT5G67370	1312	-1.56	-1.68	0.55	-2.87	0.00413	0.02922	CGLD27	CONSERVED IN THE GREEN LINEAGE AND DIATOMS 27
	AT5G18400	1006	-1.56	-1.70	0.57	-2.73	0.00637	0.04012	AtDRE2	homolog of yeast DRE2
	AT5G54855	929	-1.56	-1.70	0.58	-2.71	0.00679	0.04204	NA	NA
	AT5G53560	5653	-1.57	-1.68	0.54	-2.90	0.00369	0.02699	ATB5-A	0
	AT4G26840	2020	-1.57	-1.67	0.52	-3.03	0.00245	0.02018	ATSUMO1	ARABIDOPSIS THALIANA SMALL UBIQUITIN-LIKE MODIFIER 1
	AT3G44010	5769	-1.57	-1.71	0.59	-2.67	0.00765	0.04569	NA	NA
	AT3G15850	5384	-1.57	-1.68	0.54	-2.91	0.00367	0.02693	ADS3	0
	AT5G16650	871	-1.57	-1.67	0.52	-3.00	0.00268	0.02142	NA	NA
	AT1G69570	129	-1.57	-1.70	0.58	-2.72	0.00649	0.04071	NA	NA
	AT1G21550	415	-1.57	-1.69	0.55	-2.83	0.00461	0.03166	NA	NA
	AT1G13245	4029	-1.57	-1.71	0.58	-2.70	0.00694	0.04273	DVL4	DEVIL 4
	AT5G11340	995	-1.58	-1.70	0.55	-2.86	0.00417	0.02948	NA	NA
	AT2G02760	4746	-1.58	-1.72	0.60	-2.64	0.00841	0.04845	ATUBC2	ubiquiting-conjugating enzyme 2
	AT4G33467	150	-1.58	-1.71	0.57	-2.78	0.00541	0.03573	NA	NA
	AT5G11690	465	-1.58	-1.71	0.57	-2.77	0.00554	0.03629	ATTIM17-3	ARABIDOPSIS THALIANA TRANSLOCASE INNER MEMBRANE SUBUNIT 17-3

Supplemental Table 1-RNA seq data comparing Pro35S::MYB63 with wild type.

DAP.MYB63.target(1.5kb upstream TSS)	Gene	baseMean	log2FoldChange	lfcMLE	lfcSE	stat	pvalue	padj (FDR)	TAIR10 Symbol	TAIR10 Annotation (Short)
	AT4G16008	258	-1.58	-1.71	0.57	-2.78	0.00543	0.03580	NA	NA
	AT3G05180	456	-1.58	-1.69	0.52	-3.02	0.00256	0.02074	NA	NA
	AT5G63670	859	-1.58	-1.68	0.51	-3.11	0.00185	0.01627	SPT42	SPT4 homolog 2
	AT4G24660	684	-1.58	-1.71	0.56	-2.83	0.00464	0.03180	ATHB22	HOMEOBOX PROTEIN 22
YES	AT4G27740	1150	-1.58	-1.71	0.56	-2.82	0.00479	0.03265	NA	NA
	AT1G05785	212	-1.58	-1.73	0.60	-2.65	0.00806	0.04724	NA	NA
	AT3G03120	397	-1.58	-1.70	0.54	-2.90	0.00367	0.02693	ARFB1C	ADP-ribosylation factor B1C
	AT3G28930	1126	-1.58	-1.69	0.51	-3.11	0.00189	0.01653	AIG2	AVRRPT2-INDUCED GENE 2
	AT2G22540	3490	-1.58	-1.70	0.54	-2.92	0.00348	0.02600	AGL22	AGAMOUS-like 22
	AT1G69935	651	-1.58	-1.70	0.55	-2.91	0.00367	0.02693	SHW1	SHORT HYPOCOTYL IN WHITE LIGHT1
	AT5G60340	1600	-1.59	-1.73	0.58	-2.71	0.00664	0.04137	AAK6	Arabidopsis adenylate kinase 6
	AT5G55160	2979	-1.59	-1.69	0.51	-3.12	0.00184	0.01622	ATSUMO2	0
	AT5G46730	237	-1.59	-1.74	0.60	-2.64	0.00822	0.04789	NA	NA
	AT1G08900	869	-1.59	-1.73	0.58	-2.75	0.00596	0.03828	NA	NA
	AT2G44600	155	-1.59	-1.73	0.58	-2.74	0.00612	0.03897	NA	NA
	AT2G36830	9189	-1.59	-1.71	0.55	-2.90	0.00373	0.02720	GAMMA-TI	gamma tonoplast intrinsic protein
	AT3G45160	1476	-1.60	-1.70	0.53	-3.03	0.00244	0.02012	NA	NA
	AT2G24390	287	-1.60	-1.71	0.54	-2.93	0.00335	0.02529	NA	NA
	AT5G39250	328	-1.60	-1.73	0.57	-2.81	0.00503	0.03372	NA	NA
YES	AT4G30630	198	-1.60	-1.74	0.59	-2.71	0.00682	0.04224	NA	NA
	AT1G52590	574	-1.60	-1.70	0.52	-3.10	0.00195	0.01683	NA	NA
	AT1G04800	1274	-1.60	-1.72	0.54	-2.97	0.00296	0.02316	NA	NA
	AT5G10810	1031	-1.60	-1.74	0.58	-2.75	0.00596	0.03828	ATER	ARABIDOPSIS THALIANA ENHANCER OF RUDIMENTARY HOMOLOGUE
	AT1G20030	585	-1.60	-1.72	0.55	-2.90	0.00375	0.02727	NA	NA
	AT2G41110	1152	-1.60	-1.74	0.58	-2.77	0.00568	0.03698	ATCAL5	0
	AT1G04555	367	-1.60	-1.72	0.55	-2.93	0.00336	0.02533	NA	NA
	AT5G40080	306	-1.60	-1.72	0.53	-3.00	0.00271	0.02162	NA	NA
	AT4G22820	735	-1.60	-1.71	0.52	-3.07	0.00215	0.01820	NA	NA
	AT5G52450	151	-1.60	-1.73	0.55	-2.92	0.00352	0.02616	NA	NA
	AT5G27830	441	-1.60	-1.72	0.54	-2.96	0.00305	0.02370	NA	NA
	AT5G44820	371	-1.61	-1.74	0.58	-2.77	0.00559	0.03651	NA	NA
	AT1G54770	341	-1.61	-1.72	0.53	-3.03	0.00244	0.02012	NA	NA
	AT2G39400	995	-1.61	-1.73	0.56	-2.87	0.00417	0.02945	NA	NA
	AT5G62920	343	-1.61	-1.72	0.54	-2.99	0.00277	0.02198	ARR6	response regulator 6
	AT1G51660	603	-1.61	-1.72	0.54	-3.00	0.00270	0.02156	ATMEK4	ARABIDOPSIS THALIANA MITOGEN-ACTIVATED PROTEIN KINASE KINASE 4
	AT2G46735	286	-1.61	-1.73	0.55	-2.94	0.00325	0.02473	NA	NA
	AT5G09250	476	-1.61	-1.74	0.57	-2.83	0.00471	0.03218	KIWI	0
	AT3G28130	703	-1.61	-1.73	0.55	-2.92	0.00352	0.02616	UMAMIT44	Usually multiple acids move in and out Transporters 44
	AT5G06870	571	-1.61	-1.72	0.52	-3.08	0.00207	0.01771	ATPGIP2	ARABIDOPSIS POLYGALACTURONASE INHIBITING PROTEIN 2
	AT5G67510	1918	-1.61	-1.73	0.55	-2.95	0.00317	0.02430	NA	NA
	AT2G32080	1921	-1.61	-1.75	0.59	-2.75	0.00602	0.03853	PUR ALPHA	purin-rich alpha 1
	AT5G17000	308	-1.61	-1.73	0.54	-2.96	0.00312	0.02405	NA	NA
	AT1G30845	198	-1.61	-1.76	0.60	-2.69	0.00705	0.04326	NA	NA
	AT5G03120	620	-1.61	-1.72	0.52	-3.07	0.00213	0.01811	NA	NA
	AT3G63140	7108	-1.61	-1.77	0.61	-2.65	0.00796	0.04688	CSP41A	chloroplast stem-loop binding protein of 41 kDa
	AT5G67420	7010	-1.61	-1.72	0.51	-3.15	0.00163	0.01480	ASL39	ASYMMETRIC LEAVES2-LIKE 39
	AT2G26580	727	-1.61	-1.75	0.57	-2.84	0.00448	0.03103	YAB5	YABBY5
	AT4G40040	14655	-1.61	-1.72	0.52	-3.12	0.00183	0.01618	H3.3	histone 3.3
	AT1G19740	5033	-1.61	-1.74	0.55	-2.91	0.00361	0.02661	NA	NA
	AT5G59070	218	-1.62	-1.75	0.56	-2.87	0.00414	0.02930	NA	NA
	AT5G46710	589	-1.62	-1.73	0.53	-3.05	0.00228	0.01902	NA	NA
	AT4G16146	371	-1.62	-1.74	0.55	-2.95	0.00322	0.02456	NA	NA
	AT3G50970	196	-1.62	-1.77	0.61	-2.65	0.00813	0.04757	LTI30	LOW TEMPERATURE-INDUCED 30
	AT3G19520	1033	-1.62	-1.75	0.58	-2.80	0.00503	0.03375	NA	NA
	AT3G51970	85	-1.62	-1.78	0.62	-2.63	0.00858	0.04913	ASAT1	acyl-CoA sterol acyl transferase 1
	AT1G73885	7921	-1.62	-1.74	0.56	-2.91	0.00367	0.02693	NA	NA
	AT3G58100	115	-1.62	-1.77	0.59	-2.73	0.00643	0.04035	PDCB5	plasmodesmata callose-binding protein 5
	AT1G01230	796	-1.62	-1.73	0.52	-3.10	0.00191	0.01664	NA	NA
	AT5G17710	2116	-1.62	-1.75	0.56	-2.91	0.00365	0.02684	EMB1241	embryo defective 1241
	AT2G45695	262	-1.62	-1.76	0.59	-2.76	0.00571	0.03710	URM11	ubiquitin-related modifier 11
	AT1G70890	1241	-1.62	-1.74	0.54	-2.99	0.00281	0.02220	MLP43	MLP-like protein 43
	AT3G20600	691	-1.62	-1.73	0.52	-3.09	0.00200	0.01720	NDR1	non race-specific disease resistance 1
	AT5G43700	1128	-1.62	-1.75	0.55	-2.95	0.00318	0.02435	ATAUX2-11	AUXIN INDUCIBLE 2-11

Supplemental Table 1-RNA seq data comparing Pro35S::MYB63 with wild type.

DAP.MYB63.target(1.5kb upstream TSS)	Gene	baseMean	log2FoldChange	lfcMLE	lfcSE	stat	pvalue	padj (FDR)	TAIR10 Symbol	TAIR10 Annotation (Short)
	AT4G39950	257	-1.62	-1.76	0.57	-2.84	0.00446	0.03091	CYP79B2	cytochrome P450, family 79, subfamily B, polypeptide 2
	AT4G21110	1274	-1.62	-1.75	0.55	-2.95	0.00322	0.02458	NA	NA
	AT4G18370	1161	-1.62	-1.78	0.61	-2.65	0.00808	0.04733	DEG5	degradation of periplasmic proteins 5
	AT4G36410	173	-1.62	-1.75	0.56	-2.91	0.00362	0.02667	UBC17	ubiquitin-conjugating enzyme 17
	AT1G64850	1379	-1.63	-1.73	0.52	-3.15	0.00163	0.01484	NA	NA
	AT4G25700	231	-1.63	-1.74	0.53	-3.05	0.00226	0.01890	B1	0
	AT5G26800	547	-1.63	-1.79	0.61	-2.65	0.00801	0.04707	NA	NA
	AT5G11630	439	-1.63	-1.75	0.55	-2.96	0.00303	0.02360	NOXY2	nonresponding to oxylipins 2
	AT1G56420	129	-1.63	-1.79	0.62	-2.63	0.00844	0.04855	NA	NA
	AT4G13575	475	-1.63	-1.76	0.56	-2.90	0.00373	0.02721	NA	NA
	AT5G51620	608	-1.63	-1.77	0.57	-2.85	0.00441	0.03066	NA	NA
	AT1G01210	193	-1.63	-1.75	0.55	-2.97	0.00300	0.02343	NA	NA
	AT1G14880	8769	-1.63	-1.73	0.49	-3.33	0.00085	0.00922	AtPCR1	0
	AT3G12710	109	-1.63	-1.76	0.55	-2.96	0.00312	0.02407	NA	NA
YES	AT5G51960	457	-1.63	-1.75	0.54	-3.02	0.00250	0.02048	NA	NA
	AT3G51080	474	-1.63	-1.76	0.56	-2.90	0.00375	0.02724	GATA6	GATA transcription factor 6
YES	AT1G06010	350	-1.63	-1.75	0.53	-3.06	0.00223	0.01872	NA	NA
	AT5G12390	570	-1.63	-1.75	0.53	-3.08	0.00210	0.01785	FIS1B	FISSION 1B
	AT5G13500	458	-1.63	-1.80	0.62	-2.62	0.00879	0.04989	NA	NA
	AT2G15050	2645	-1.64	-1.77	0.57	-2.86	0.00426	0.02995	LTP	lipid transfer protein
	AT1G12440	4046	-1.64	-1.78	0.59	-2.75	0.00589	0.03795	NA	NA
	AT5G03230	770	-1.64	-1.77	0.56	-2.92	0.00353	0.02620	NA	NA
	AT5G36290	1549	-1.64	-1.77	0.56	-2.91	0.00356	0.02640	NA	NA
	AT3G12587	649	-1.64	-1.79	0.60	-2.72	0.00643	0.04038	NA	NA
	AT1G66240	2046	-1.64	-1.76	0.54	-3.01	0.00265	0.02130	ATATX1	0
	AT5G64350	12817	-1.64	-1.78	0.58	-2.84	0.00451	0.03116	ATFKBP12	ARABIDOPSIS THALIANA FK506-BINDING PROTEIN 12
	AT4G27270	350	-1.64	-1.78	0.58	-2.81	0.00490	0.03312	NA	NA
	AT1G05340	157	-1.64	-1.77	0.57	-2.90	0.00373	0.02720	NA	NA
	AT4G16520	5125	-1.64	-1.75	0.52	-3.15	0.00163	0.01480	ATG8F	autophagy 8f
	AT2G24762	574	-1.64	-1.77	0.56	-2.94	0.00325	0.02469	AtGDU4	glutamine dumper 4
	AT1G32120	306	-1.64	-1.78	0.57	-2.86	0.00426	0.02994	NA	NA
	AT5G62540	784	-1.64	-1.76	0.53	-3.10	0.00196	0.01697	UBC3	ubiquitin-conjugating enzyme 3
	AT5G47120	3870	-1.64	-1.76	0.53	-3.11	0.00187	0.01642	ATBI-1	ARABIDOPSIS BAX INHIBITOR 1
	AT3G53630	698	-1.64	-1.77	0.55	-2.96	0.00307	0.02377	NA	NA
YES	AT5G26667	1719	-1.64	-1.76	0.53	-3.10	0.00192	0.01671	PYR6	0
	AT4G24350	627	-1.64	-1.76	0.53	-3.08	0.00204	0.01749	NA	NA
	AT5G59880	8914	-1.64	-1.75	0.52	-3.16	0.00159	0.01455	ADF3	actin depolymerizing factor 3
	AT5G15520	546	-1.64	-1.78	0.57	-2.88	0.00403	0.02873	NA	NA
	AT1G62250	489	-1.64	-1.77	0.56	-2.95	0.00319	0.02440	NA	NA
	AT2G31200	2148	-1.65	-1.75	0.51	-3.21	0.00132	0.01261	ADF6	actin depolymerizing factor 6
	AT3G52500	5688	-1.65	-1.77	0.54	-3.03	0.00243	0.02004	NA	NA
	AT2G41200	295	-1.65	-1.79	0.59	-2.81	0.00494	0.03337	NA	NA
	AT4G24210	561	-1.65	-1.77	0.55	-3.01	0.00262	0.02113	SLY1	SLEEPY1
	AT1G12244	238	-1.65	-1.78	0.56	-2.95	0.00316	0.02426	NA	NA
	AT1G34000	4036	-1.65	-1.74	0.49	-3.35	0.00080	0.00873	OHP2	one-helix protein 2
	AT3G53420	28298	-1.65	-1.78	0.57	-2.88	0.00393	0.02822	AtPIP2;1	0
	AT4G20360	22188	-1.65	-1.78	0.56	-2.93	0.00338	0.02542	ATRAB8D	0
	AT3G10090	1461	-1.65	-1.79	0.58	-2.83	0.00469	0.03209	NA	NA
	ATCG00540	412	-1.65	-1.82	0.63	-2.62	0.00873	0.04970	PETA	photosynthetic electron transfer A
	AT3G60260	250	-1.65	-1.82	0.62	-2.64	0.00828	0.04807	NA	NA
	AT4G31870	186	-1.65	-1.80	0.59	-2.77	0.00554	0.03628	ATGPX7	GLUTATHIONE PEROXIDASE 7
	AT1G80040	1294	-1.65	-1.77	0.53	-3.11	0.00188	0.01647	NA	NA
	AT5G21274	2411	-1.65	-1.76	0.52	-3.17	0.00153	0.01409	ACAM-6	0
	AT5G57000	358	-1.65	-1.79	0.56	-2.93	0.00340	0.02555	NA	NA
	AT1G46768	283	-1.65	-1.80	0.59	-2.78	0.00537	0.03549	RAP2.1	related to AP2 1
	AT5G20130	1194	-1.65	-1.80	0.58	-2.83	0.00471	0.03218	NA	NA
	AT5G14700	205	-1.65	-1.78	0.54	-3.05	0.00228	0.01898	NA	NA
	AT3G54826	728	-1.65	-1.77	0.53	-3.14	0.00167	0.01505	ZR3	zinc ribbon 3
YES	AT5G63480	345	-1.65	-1.82	0.62	-2.68	0.00725	0.04405	NA	NA
	AT2G35520	716	-1.65	-1.77	0.52	-3.18	0.00146	0.01361	DAD2	DEFENDER AGAINST CELL DEATH 2
	AT5G05990	315	-1.66	-1.77	0.52	-3.17	0.00153	0.01410	NA	NA
	AT5G19260	705	-1.66	-1.79	0.56	-2.97	0.00302	0.02354	FAF3	FANTASTIC FOUR 3
	AT5G39670	737	-1.66	-1.78	0.54	-3.07	0.00213	0.01811	NA	NA
	AT1G63460	1179	-1.66	-1.80	0.58	-2.86	0.00424	0.02985	ATGPX8	0
	AT4G28270	953	-1.66	-1.81	0.59	-2.83	0.00471	0.03218	ATRMA2	0
	AT1G14600	254	-1.66	-1.78	0.54	-3.05	0.00231	0.01921	NA	NA
	AT3G15518	150	-1.66	-1.81	0.59	-2.83	0.00471	0.03218	NA	NA
	AT5G60400	1370	-1.66	-1.81	0.58	-2.85	0.00433	0.03022	NA	NA

Supplemental Table 1-RNA seq data comparing Pro35S::MYB63 with wild type.

DAP MYB63.target(1.5kb upstream TSS)	Gene	baseMean	log2FoldChange	lfcMLE	lfcSE	stat	pvalue	padj (FDR)	TAIR10 Symbol	TAIR10 Annotation (Short)
	AT4G17410	304	-1.66	-1.81	0.59	-2.84	0.00446	0.03095	NA	NA
	AT1G53542	281	-1.67	-1.83	0.61	-2.72	0.00651	0.04076	NA	NA
	AT3G28100	391	-1.67	-1.79	0.54	-3.09	0.00202	0.01733	UMAMIT45	Usually multiple acids move in and out Transporters 45
	AT1G29960	224	-1.67	-1.81	0.57	-2.91	0.00357	0.02645	NA	NA
YES	AT4G02970	3716	-1.67	-1.81	0.58	-2.86	0.00423	0.02978	AT7SL-1	7SL RNA1
YES	AT5G13190	2323	-1.67	-1.77	0.51	-3.29	0.00101	0.01036	AtGILP	0
	AT1G78790	264	-1.67	-1.78	0.52	-3.22	0.00130	0.01241	NA	NA
	AT2G33830	12450	-1.67	-1.81	0.57	-2.94	0.00323	0.02462	AtDRM2	0
	AT5G65590	132	-1.67	-1.82	0.59	-2.84	0.00452	0.03123	SCAP1	STOMATAL CARPENTER 1
	AT1G12450	291	-1.67	-1.85	0.63	-2.66	0.00774	0.04599	NA	NA
	AT5G45010	1004	-1.67	-1.81	0.56	-2.96	0.00304	0.02365	ATDSS1(V)	DSS1 homolog on chromosome V
	AT5G01650	2113	-1.67	-1.80	0.54	-3.07	0.00214	0.01815	NA	NA
	AT1G27330	2820	-1.67	-1.80	0.56	-2.99	0.00276	0.02191	NA	NA
	AT1G14700	337	-1.67	-1.79	0.53	-3.15	0.00165	0.01494	ATPAP3	0
	AT4G17170	2373	-1.67	-1.78	0.52	-3.22	0.00127	0.01218	AT-RAB2	0
	AT1G16916	585	-1.67	-1.84	0.61	-2.73	0.00625	0.03956	NA	NA
	AT2G47710	3950	-1.68	-1.80	0.54	-3.12	0.00182	0.01611	NA	NA
	AT1G49140	1626	-1.68	-1.81	0.55	-3.02	0.00251	0.02053	NA	NA
	AT4G11175	1789	-1.68	-1.79	0.52	-3.25	0.00114	0.01129	NA	NA
	AT3G22530	475	-1.68	-1.80	0.54	-3.08	0.00207	0.01771	NA	NA
	AT2G46600	8684	-1.68	-1.78	0.50	-3.33	0.00086	0.00924	NA	NA
	AT4G14410	1036	-1.68	-1.83	0.60	-2.81	0.00495	0.03340	bHLH104	basic Helix-Loop-Helix 104
	AT2G26340	1892	-1.68	-1.80	0.53	-3.17	0.00154	0.01414	NA	NA
	AT3G44100	3551	-1.68	-1.80	0.53	-3.15	0.00161	0.01465	NA	NA
	AT3G55170	1292	-1.68	-1.80	0.53	-3.19	0.00141	0.01319	NA	NA
	AT2G42210	4287	-1.68	-1.80	0.53	-3.18	0.00149	0.01381	ATOEP16-3	0
	AT1G71050	352	-1.68	-1.82	0.56	-3.00	0.00266	0.02130	HIPP20	heavy metal associated isoprenylated plant protein 20
	AT1G66180	737	-1.68	-1.81	0.54	-3.13	0.00172	0.01545	NA	NA
	AT1G19400	1126	-1.68	-1.81	0.54	-3.12	0.00180	0.01597	NA	NA
YES	AT3G24030	295	-1.68	-1.85	0.61	-2.76	0.00569	0.03702	NA	NA
	AT1G22750	2538	-1.68	-1.82	0.57	-2.96	0.00310	0.02397	NA	NA
	AT3G26990	79	-1.68	-1.87	0.64	-2.65	0.00815	0.04764	NA	NA
	AT3G24730	295	-1.69	-1.86	0.62	-2.73	0.00636	0.04009	NA	NA
	AT3G12685	296	-1.69	-1.83	0.57	-2.93	0.00336	0.02533	NA	NA
	AT5G41520	6844	-1.69	-1.79	0.51	-3.33	0.00086	0.00929	RPS10B	ribosomal protein S10e B
	AT5G58375	2007	-1.69	-1.81	0.54	-3.14	0.00169	0.01519	NA	NA
	AT5G42530	68233	-1.69	-1.79	0.50	-3.38	0.00071	0.00808	NA	NA
	AT1G21065	3579	-1.69	-1.82	0.55	-3.07	0.00215	0.01817	NA	NA
	AT5G42980	17340	-1.69	-1.83	0.56	-3.00	0.00274	0.02180	ATH3	thioredoxin H-type 3
	AT4G38840	2806	-1.69	-1.86	0.61	-2.77	0.00561	0.03662	SAUR14	SMALL AUXIN UPREGULATED RNA 14
	AT1G80500	2277	-1.69	-1.83	0.56	-3.00	0.00268	0.02142	NA	NA
	AT1G09575	191	-1.69	-1.83	0.56	-3.01	0.00260	0.02099	NA	NA
	AT3G53850	390	-1.69	-1.84	0.58	-2.90	0.00375	0.02724	NA	NA
	AT2G46100	2361	-1.69	-1.84	0.59	-2.89	0.00379	0.02751	NA	NA
	AT1G04270	14178	-1.70	-1.81	0.52	-3.28	0.00104	0.01063	RPS15	cytosolic ribosomal protein S15
	AT2G21530	3144	-1.70	-1.88	0.64	-2.67	0.00760	0.04549	NA	NA
	AT5G01430	453	-1.70	-1.84	0.56	-3.02	0.00252	0.02056	NA	NA
	AT3G17790	787	-1.70	-1.83	0.56	-3.05	0.00232	0.01929	ATACP5	0
	AT2G01660	122	-1.70	-1.85	0.58	-2.94	0.00330	0.02502	PDLP6	plasmodesmata-located protein 6
	AT3G23760	429	-1.70	-1.85	0.58	-2.96	0.00308	0.02384	NA	NA
	AT5G17190	2576	-1.70	-1.82	0.53	-3.24	0.00121	0.01184	NA	NA
	AT3G60200	437	-1.70	-1.83	0.55	-3.12	0.00182	0.01611	NA	NA
	AT4G16566	326	-1.70	-1.83	0.54	-3.17	0.00152	0.01404	HINT4	histidine triad nucleotide-binding 4
	AT3G26030	2050	-1.70	-1.85	0.57	-3.00	0.00269	0.02151	ATB' DELTA	serine/threonine protein phosphatase 2A 55 kDa regulatory subunit B prime delta
	AT5G47630	718	-1.71	-1.83	0.55	-3.11	0.00187	0.01642	mtACP3	mitochondrial acyl carrier protein 3
	AT5G35680	1805	-1.71	-1.82	0.52	-3.30	0.00098	0.01015	NA	NA
	AT3G63390	274	-1.71	-1.83	0.53	-3.21	0.00132	0.01261	NA	NA
	AT3G43980	1227	-1.71	-1.87	0.60	-2.84	0.00455	0.03137	NA	NA
	AT3G11200	1581	-1.71	-1.85	0.57	-2.99	0.00278	0.02200	AL2	alfin-like 2
	AT1G50740	2232	-1.71	-1.86	0.60	-2.86	0.00425	0.02992	NA	NA
	AT3G20830	118	-1.71	-1.87	0.61	-2.81	0.00490	0.03312	AGC2-4	AGC2 kinase 4
	AT1G50020	2206	-1.71	-1.84	0.56	-3.04	0.00238	0.01974	NA	NA
	AT2G25190	548	-1.71	-1.85	0.57	-3.00	0.00268	0.02145	NA	NA
	AT1G52230	70461	-1.71	-1.82	0.51	-3.32	0.00090	0.00952	PSAH-2	PHOTOSYSTEM I SUBUNIT H-2
	AT3G05160	478	-1.71	-1.84	0.54	-3.15	0.00162	0.01471	NA	NA
	AT2G19690	126	-1.71	-1.89	0.64	-2.69	0.00713	0.04363	PLA2-BETA	phospholipase A2-beta
	AT2G23130	1188	-1.71	-1.85	0.57	-3.02	0.00255	0.02073	AGP17	arabinogalactan protein 17

Supplemental Table 1-RNA seq data comparing Pro35S::MYB63 with wild type.

DAP.MYB63.target(1.5kb upstream TSS)	Gene	baseMean	log2FoldChange	lfcMLE	lfcSE	stat	pvalue	padj (FDR)	TAIR10 Symbol	TAIR10 Annotation (Short)
	AT5G12240	983	-1.71	-1.84	0.55	-3.10	0.00193	0.01671	NA	NA
	AT4G26230	4893	-1.71	-1.84	0.55	-3.13	0.00175	0.01571	NA	NA
	AT4G08230	1328	-1.71	-1.83	0.53	-3.21	0.00134	0.01268	NA	NA
	AT2G39270	548	-1.71	-1.89	0.62	-2.76	0.00585	0.03778	NA	NA
	AT4G30760	1810	-1.72	-1.84	0.55	-3.15	0.00165	0.01494	NA	NA
	AT1G75750	9982	-1.72	-1.85	0.56	-3.07	0.00212	0.01799	GASA1	GAST1 protein homolog 1
	AT5G45550	1831	-1.72	-1.86	0.58	-2.96	0.00303	0.02362	MOB1-like	MOB1-like
YES	AT3G01500	49687	-1.72	-1.83	0.51	-3.34	0.00084	0.00911	ATBCA1	BETA CARBONIC ANHYDRASE 1
	AT4G21560	975	-1.72	-1.84	0.53	-3.26	0.00112	0.01118	VPS28	vacuolar protein sorting 28
	AT3G09500	10189	-1.72	-1.82	0.50	-3.43	0.00059	0.00708	NA	NA
	AT5G26990	389	-1.72	-1.89	0.61	-2.82	0.00485	0.03288	NA	NA
	AT3G55330	2709	-1.72	-1.86	0.56	-3.05	0.00230	0.01916	PPL1	PsbP-like protein 1
	AT1G20816	416	-1.72	-1.86	0.56	-3.07	0.00216	0.01827	NA	NA
	AT2G44650	7043	-1.72	-1.85	0.54	-3.19	0.00141	0.01320	CHL-CPN10	chloroplast chaperonin 10
	AT3G04890	638	-1.72	-1.88	0.60	-2.88	0.00395	0.02829	NA	NA
	AT4G00585	2105	-1.72	-1.86	0.55	-3.11	0.00187	0.01643	NA	NA
	AT3G57785	1491	-1.72	-1.85	0.53	-3.23	0.00124	0.01202	NA	NA
	AT5G50240	212	-1.72	-1.89	0.61	-2.85	0.00438	0.03049	AtPIMT2	Arabidopsis thaliana protein-l-isoaspartate methyltransferase 2
	AT5G02230	98	-1.73	-1.90	0.61	-2.81	0.00495	0.03342	NA	NA
	AT5G06280	401	-1.73	-1.87	0.58	-2.99	0.00281	0.02220	NA	NA
	AT1G09490	1073	-1.73	-1.89	0.61	-2.83	0.00461	0.03168	NA	NA
	AT5G03050	312	-1.73	-1.87	0.56	-3.06	0.00222	0.01868	NA	NA
	AT3G11410	1520	-1.73	-1.87	0.56	-3.06	0.00218	0.01838	AHG3	0
YES	AT1G31020	221	-1.73	-1.92	0.64	-2.68	0.00733	0.04434	ATO2	thioredoxin O2
	AT4G15765	129	-1.73	-1.87	0.56	-3.06	0.00221	0.01855	NA	NA
	AT1G12020	341	-1.73	-1.85	0.53	-3.25	0.00114	0.01135	NA	NA
	AT4G24920	2569	-1.73	-1.88	0.59	-2.95	0.00317	0.02435	NA	NA
YES	AT2G44380	140	-1.73	-1.87	0.57	-3.06	0.00223	0.01871	NA	NA
	AT2G47400	18253	-1.73	-1.85	0.53	-3.26	0.00111	0.01114	CP12	CP12 DOMAIN-CONTAINING PROTEIN 1
	AT1G69410	2838	-1.73	-1.85	0.52	-3.36	0.00079	0.00873	ATELF5A-3	EUKARYOTIC ELONGATION FACTOR 5A-3
	AT2G20930	860	-1.73	-1.84	0.51	-3.39	0.00069	0.00790	NA	NA
	AT4G13200	1241	-1.73	-1.89	0.60	-2.89	0.00386	0.02780	NA	NA
	AT4G23760	666	-1.73	-1.87	0.56	-3.08	0.00209	0.01780	NA	NA
	AT3G12490	5152	-1.73	-1.89	0.59	-2.94	0.00323	0.02462	ATCYS6	ARABIDOPSIS THALIANA PHYTOCYSTATIN 6
	AT4G37450	416	-1.73	-1.84	0.50	-3.50	0.00047	0.00596	AGP18	arabinogalactan protein 18
	AT5G20190	2269	-1.74	-1.85	0.52	-3.36	0.00078	0.00865	NA	NA
	AT4G19390	496	-1.74	-1.88	0.58	-3.01	0.00261	0.02104	NA	NA
YES	AT2G38540	24500	-1.74	-1.88	0.56	-3.09	0.00199	0.01717	ATLTP1	ARABIDOPSIS THALIANA LIPID TRANSFER PROTEIN 1
	AT2G14910	4654	-1.74	-1.89	0.58	-2.99	0.00278	0.02200	NA	NA
	AT1G49000	139	-1.74	-1.90	0.59	-2.93	0.00339	0.02552	NA	NA
	AT3G13437	207	-1.74	-1.93	0.64	-2.72	0.00659	0.04109	NA	NA
	AT3G27100	302	-1.74	-1.87	0.54	-3.20	0.00136	0.01288	NA	NA
	AT5G57860	802	-1.74	-1.86	0.54	-3.24	0.00118	0.01161	NA	NA
	AT2G44620	3053	-1.74	-1.85	0.51	-3.38	0.00073	0.00818	MTACP-1	mitochondrial acyl carrier protein 1
	AT2G34430	100170	-1.74	-1.87	0.54	-3.23	0.00123	0.01198	LHB1B1	light-harvesting chlorophyll-protein complex II subunit B1
	AT2G28060	356	-1.74	-1.90	0.59	-2.95	0.00320	0.02448	NA	NA
	AT1G70350	306	-1.74	-1.88	0.57	-3.07	0.00212	0.01799	NA	NA
	AT3G29350	803	-1.74	-1.86	0.53	-3.27	0.00106	0.01077	AHP2	histidine-containing phosphotransmitter 2
	AT4G25260	397	-1.74	-1.88	0.56	-3.10	0.00195	0.01683	NA	NA
	AT5G04760	644	-1.74	-1.92	0.62	-2.81	0.00490	0.03312	NA	NA
	AT2G38130	768	-1.74	-1.87	0.54	-3.22	0.00128	0.01228	ATMAK3	0
	AT4G20150	5895	-1.75	-1.86	0.51	-3.43	0.00061	0.00720	NA	NA
	AT4G18580	188	-1.75	-1.88	0.56	-3.14	0.00170	0.01533	NA	NA
	AT3G62650	1872	-1.75	-1.87	0.53	-3.30	0.00096	0.00995	NA	NA
	AT2G46030	1217	-1.75	-1.87	0.54	-3.24	0.00121	0.01181	UBC6	ubiquitin-conjugating enzyme 6
	AT3G22230	9161	-1.75	-1.87	0.53	-3.32	0.00088	0.00943	NA	NA
	AT2G27330	390	-1.75	-1.92	0.61	-2.88	0.00398	0.02840	NA	NA
	AT2G39440	372	-1.75	-1.88	0.55	-3.16	0.00158	0.01446	NA	NA
	AT3G52800	1774	-1.75	-1.87	0.52	-3.34	0.00083	0.00899	NA	NA
	AT5G14730	216	-1.75	-1.91	0.59	-2.96	0.00306	0.02371	NA	NA
	AT3G58730	5398	-1.75	-1.88	0.55	-3.21	0.00133	0.01265	NA	NA
	AT3G61970	297	-1.75	-1.87	0.52	-3.35	0.00081	0.00885	NGA2	NGATHA2
	AT2G23430	250	-1.75	-1.89	0.56	-3.11	0.00187	0.01641	ICK1	0
	AT5G49610	175	-1.75	-1.94	0.64	-2.75	0.00598	0.03833	NA	NA

Supplemental Table 1-RNA seq data comparing Pro35S::MYB63 with wild type.

DAP.MYB63.target(1.5kb upstream TSS)	Gene	baseMean	log2FoldChange	lfcMLE	lfcSE	stat	pvalue	padj (FDR)	TAIR10 Symbol	TAIR10 Annotation (Short)
	AT1G25230	649	-1.75	-1.89	0.56	-3.12	0.00184	0.01622	NA	NA
	AT5G62360	443	-1.75	-1.88	0.53	-3.32	0.00090	0.00953	NA	NA
	AT2G40765	2482	-1.76	-1.88	0.53	-3.31	0.00093	0.00979	NA	NA
	AT2G37120	1285	-1.76	-1.89	0.55	-3.21	0.00133	0.01261	NA	NA
	AT1G80180	3056	-1.76	-1.88	0.54	-3.27	0.00107	0.01080	NA	NA
	AT4G26710	4408	-1.76	-1.86	0.49	-3.59	0.00033	0.00457	NA	NA
	AT1G22050	315	-1.76	-1.90	0.56	-3.13	0.00172	0.01543	MUB6	membrane-anchored ubiquitin-fold protein 6 precursor
	AT3G60290	166	-1.76	-1.95	0.64	-2.73	0.00634	0.04001	NA	NA
	AT5G62180	130	-1.76	-1.97	0.66	-2.68	0.00737	0.04453	AtCXE20	carboxyesterase 20
	AT2G36090	163	-1.76	-1.91	0.58	-3.05	0.00226	0.01886	NA	NA
	AT2G29290	1029	-1.76	-1.89	0.53	-3.30	0.00098	0.01008	NA	NA
	AT3G13882	955	-1.76	-1.94	0.62	-2.86	0.00426	0.02994	NA	NA
	AT4G37240	780	-1.76	-1.89	0.54	-3.24	0.00121	0.01186	NA	NA
	AT1G47830	1117	-1.76	-1.87	0.51	-3.48	0.00050	0.00629	NA	NA
	AT1G65870	70	-1.76	-1.96	0.64	-2.74	0.00623	0.03945	NA	NA
	AT3G17860	454	-1.76	-1.95	0.63	-2.79	0.00527	0.03507	JAI3	JASMONATE-INSENSITIVE 3
	AT5G50915	500	-1.76	-1.91	0.58	-3.04	0.00235	0.01952	NA	NA
	AT3G08990	94	-1.76	-1.92	0.58	-3.02	0.00250	0.02048	NA	NA
	AT4G17650	557	-1.76	-1.89	0.53	-3.33	0.00088	0.00939	NA	NA
	AT5G14910	5403	-1.76	-1.91	0.57	-3.08	0.00208	0.01773	NA	NA
	AT5G15770	119	-1.76	-1.98	0.67	-2.62	0.00877	0.04986	AtGNA1	glucose-6-phosphate acetyltransferase 1
	AT3G15040	1263	-1.76	-1.88	0.51	-3.44	0.00059	0.00707	NA	NA
	AT1G09995	112	-1.76	-1.98	0.67	-2.63	0.00857	0.04907	NA	NA
	AT5G05090	439	-1.77	-1.90	0.55	-3.23	0.00125	0.01207	NA	NA
	AT1G15350	603	-1.77	-1.98	0.66	-2.67	0.00762	0.04559	NA	NA
	AT4G00850	145	-1.77	-1.94	0.61	-2.89	0.00390	0.02806	GIF3	GRF1-interacting factor 3
	AT5G58005	925	-1.77	-1.89	0.53	-3.32	0.00088	0.00943	NA	NA
	AT5G37055	178	-1.77	-1.95	0.62	-2.83	0.00465	0.03183	ATSWC6	0
	AT5G59550	1132	-1.77	-1.90	0.54	-3.27	0.00106	0.01077	AtRDUF2	Arabidopsis thaliana RING and Domain of Unknown Function 1117 2
YES	AT3G11100	610	-1.77	-1.94	0.61	-2.91	0.00360	0.02661	NA	NA
	AT5G49550	432	-1.77	-1.89	0.53	-3.32	0.00091	0.00957	BLOS2	BLOC subunit 2
	AT1G44000	2212	-1.77	-1.91	0.56	-3.17	0.00150	0.01390	NA	NA
	AT3G61110	7668	-1.77	-1.94	0.61	-2.89	0.00380	0.02755	ARS27A	ribosomal protein S27
	AT1G51950	896	-1.77	-1.90	0.54	-3.30	0.00096	0.00996	IAA18	indole-3-acetic acid inducible 18
	AT5G24610	1640	-1.77	-1.96	0.63	-2.81	0.00496	0.03344	NA	NA
	AT3G06420	409	-1.77	-1.92	0.57	-3.10	0.00194	0.01683	ATG8H	autophagy 8h
	AT2G45310	346	-1.77	-1.97	0.64	-2.76	0.00574	0.03719	GAE4	UDP-D-glucuronate 4-epimerase 4
	AT5G61290	87	-1.77	-1.98	0.65	-2.72	0.00658	0.04106	NA	NA
	AT1G68660	3621	-1.77	-1.90	0.53	-3.35	0.00081	0.00885	NA	NA
	AT4G22570	2221	-1.77	-1.90	0.53	-3.36	0.00079	0.00873	APT3	adenine phosphoribosyl transferase 3
	AT1G32690	94	-1.78	-1.98	0.65	-2.75	0.00603	0.03854	NA	NA
	AT2G04900	1439	-1.78	-1.92	0.56	-3.17	0.00152	0.01406	NA	NA
	AT4G22830	739	-1.78	-1.92	0.56	-3.17	0.00151	0.01398	NA	NA
	AT3G28140	443	-1.78	-1.92	0.55	-3.21	0.00133	0.01266	NA	NA
	AT1G22370	81	-1.78	-1.93	0.58	-3.05	0.00226	0.01886	AtUGT85A5	UDP-glucosyl transferase 85A5
YES	AT1G29250	1866	-1.78	-1.92	0.55	-3.23	0.00123	0.01195	NA	NA
	AT2G31710	362	-1.78	-1.93	0.58	-3.07	0.00213	0.01807	NA	NA
	AT5G17560	2459	-1.78	-1.90	0.52	-3.42	0.00063	0.00740	BolA4	homolog of E.coli BolA 4
	AT1G63970	4680	-1.78	-1.91	0.55	-3.26	0.00111	0.01113	ISPF	isoprenoid F
	AT4G22160	381	-1.78	-1.93	0.57	-3.13	0.00175	0.01564	NA	NA
	AT4G27230	1370	-1.78	-1.90	0.53	-3.39	0.00070	0.00794	HTA2	histone H2A 2
	AT4G04800	1506	-1.78	-1.92	0.55	-3.25	0.00117	0.01151	ATMSRB3	methionine sulfoxide reductase B3
	AT1G65420	445	-1.78	-1.92	0.55	-3.27	0.00109	0.01098	NPQ7	NONPHOTOCHEMICAL QUENCHING 7
	AT5G53650	2150	-1.78	-1.92	0.55	-3.25	0.00116	0.01144	NA	NA
	AT5G65520	354	-1.78	-1.90	0.52	-3.46	0.00055	0.00666	NA	NA
	AT3G22550	395	-1.79	-1.93	0.57	-3.12	0.00179	0.01596	NA	NA
	AT5G50460	3072	-1.79	-1.92	0.55	-3.24	0.00119	0.01167	NA	NA
	AT5G44190	4325	-1.79	-1.92	0.54	-3.33	0.00087	0.00933	ATGLK2	0
	AT4G09550	995	-1.79	-1.92	0.54	-3.28	0.00104	0.01057	ATGIP1	ARABIDOPSIS ATGCP3 INTERACTING PROTEIN 1
	AT4G32915	1513	-1.79	-1.96	0.61	-2.93	0.00334	0.02522	NA	NA
	AT2G32380	461	-1.79	-1.93	0.56	-3.21	0.00135	0.01277	NA	NA
YES	AT3G53470	1166	-1.79	-1.90	0.51	-3.50	0.00047	0.00594	NA	NA
	AT2G33800	8844	-1.79	-1.94	0.57	-3.13	0.00178	0.01586	EMB3113	EMBRYO DEFECTIVE 3113
	AT5G10695	1343	-1.79	-1.97	0.62	-2.90	0.00374	0.02721	NA	NA

Supplemental Table 1-RNA seq data comparing Pro35S::MYB63 with wild type.

DAP MYB63.target(1.5kb upstream TSS)	Gene	baseMean	log2FoldChange	lfcMLE	lfcSE	stat	pvalue	padj (FDR)	TAIR10 Symbol	TAIR10 Annotation (Short)
	AT4G00880	527	-1.79	-1.97	0.61	-2.94	0.00326	0.02477	SAUR31	SMALL AUXIN UPREGULATED RNA 31
	AT5G63150	883	-1.79	-1.95	0.58	-3.08	0.00209	0.01780	NA	NA
	AT3G23325	976	-1.79	-1.92	0.53	-3.37	0.00076	0.00843	NA	NA
	AT1G26670	2999	-1.79	-1.92	0.54	-3.35	0.00081	0.00885	ATVTI12	0
	AT2G01870	771	-1.79	-1.91	0.52	-3.45	0.00056	0.00679	NA	NA
	AT4G32590	1030	-1.79	-1.91	0.52	-3.47	0.00052	0.00644	NA	NA
	AT3G44280	764	-1.79	-1.93	0.55	-3.26	0.00109	0.01100	NA	NA
	AT1G71910	59	-1.79	-1.98	0.62	-2.89	0.00381	0.02761	NA	NA
	AT1G05835	334	-1.80	-2.00	0.65	-2.76	0.00580	0.03754	NA	NA
YES	AT4G24972	447	-1.80	-1.99	0.64	-2.83	0.00472	0.03219	TPD1	TAPETUM DETERMINANT 1
	AT5G36925	194	-1.80	-2.01	0.66	-2.74	0.00622	0.03940	NA	NA
	AT5G66440	395	-1.80	-1.94	0.56	-3.23	0.00125	0.01205	NA	NA
	AT2G46690	986	-1.80	-1.94	0.56	-3.23	0.00125	0.01207	SAUR32	SMALL AUXIN UPREGULATED RNA 32
	AT5G62150	66	-1.80	-1.98	0.62	-2.90	0.00369	0.02701	NA	NA
	AT5G11000	852	-1.80	-2.02	0.67	-2.69	0.00707	0.04333	NA	NA
	AT3G46430	3497	-1.80	-1.92	0.53	-3.37	0.00074	0.00833	NA	NA
	AT4G14870	2556	-1.80	-1.92	0.53	-3.42	0.00063	0.00741	SECE1	0
	AT3G15395	337	-1.80	-1.97	0.60	-3.01	0.00260	0.02097	NA	NA
	AT1G21520	1117	-1.80	-1.93	0.53	-3.37	0.00076	0.00845	NA	NA
	AT1G47820	342	-1.80	-1.94	0.55	-3.29	0.00101	0.01036	NA	NA
	AT5G06210	597	-1.80	-1.93	0.53	-3.37	0.00075	0.00840	NA	NA
	AT1G08890	195	-1.80	-1.96	0.58	-3.09	0.00199	0.01714	NA	NA
	AT1G49980	538	-1.80	-1.94	0.55	-3.29	0.00101	0.01036	NA	NA
	AT3G43740	1072	-1.80	-1.97	0.60	-3.02	0.00256	0.02079	NA	NA
	AT1G22330	275	-1.80	-1.93	0.53	-3.43	0.00061	0.00720	NA	NA
	AT3G09735	1978	-1.80	-1.94	0.56	-3.25	0.00116	0.01144	NA	NA
	AT1G13190	1412	-1.80	-1.94	0.55	-3.28	0.00104	0.01063	NA	NA
	AT4G21870	439	-1.80	-1.96	0.58	-3.12	0.00180	0.01598	NA	NA
	AT1G17330	221	-1.80	-1.98	0.61	-2.94	0.00326	0.02477	NA	NA
	AT1G72800	169	-1.80	-1.96	0.58	-3.11	0.00186	0.01639	NA	NA
	AT1G72230	428	-1.80	-1.97	0.59	-3.04	0.00235	0.01946	NA	NA
	AT4G21445	942	-1.80	-1.95	0.56	-3.22	0.00126	0.01216	NA	NA
	AT4G23710	1700	-1.81	-1.92	0.51	-3.52	0.00043	0.00561	VAG2	vacuolar ATP synthase subunit G2
	AT1G28540	328	-1.81	-1.96	0.57	-3.20	0.00140	0.01311	NA	NA
	AT4G26810	277	-1.81	-1.94	0.54	-3.35	0.00080	0.00880	NA	NA
	AT1G21770	1006	-1.81	-1.92	0.49	-3.68	0.00023	0.00349	NA	NA
	AT5G40710	313	-1.81	-2.01	0.65	-2.81	0.00500	0.03366	NA	NA
	AT4G14342	2478	-1.81	-1.95	0.55	-3.27	0.00108	0.01090	NA	NA
	AT3G56030	41	-1.81	-2.04	0.68	-2.67	0.00760	0.04549	NA	NA
	AT5G45350	1574	-1.81	-1.94	0.53	-3.42	0.00064	0.00742	NA	NA
	AT1G56060	192	-1.81	-2.05	0.68	-2.66	0.00779	0.04619	NA	NA
	AT1G55160	848	-1.81	-1.94	0.53	-3.40	0.00067	0.00774	NA	NA
	ATCG00680	1528	-1.81	-1.99	0.61	-2.99	0.00282	0.02225	PSBB	photosystem II reaction center protein B
	AT3G45740	265	-1.81	-2.00	0.62	-2.94	0.00332	0.02510	NA	NA
	AT1G49410	2133	-1.81	-1.95	0.55	-3.30	0.00097	0.01001	TOM6	translocase of the outer mitochondrial membrane 6
	AT1G57630	255	-1.81	-2.02	0.65	-2.81	0.00493	0.03330	NA	NA
	AT4G14270	3468	-1.82	-1.97	0.58	-3.14	0.00166	0.01503	NA	NA
	AT1G67060	567	-1.82	-2.00	0.62	-2.94	0.00327	0.02483	NA	NA
	AT3G16530	1705	-1.82	-1.96	0.56	-3.26	0.00113	0.01125	NA	NA
	AT2G41750	97	-1.82	-1.99	0.60	-3.02	0.00253	0.02060	NA	NA
	AT1G56200	2992	-1.82	-1.94	0.52	-3.53	0.00042	0.00546	EMB1303	embryo defective 1303
	AT5G09960	1074	-1.82	-1.95	0.53	-3.42	0.00063	0.00736	NA	NA
	AT3G52230	5162	-1.82	-1.95	0.54	-3.35	0.00080	0.00873	NA	NA
	AT4G34870	14093	-1.82	-1.95	0.52	-3.47	0.00051	0.00636	ATCYP1	ARABIDOPSIS THALIANA CYCLOPHILIN 1
	AT5G55290	1136	-1.82	-1.96	0.55	-3.33	0.00086	0.00928	NA	NA
	AT3G15480	2341	-1.82	-1.94	0.51	-3.55	0.00038	0.00512	NA	NA
	AT3G12650	607	-1.82	-1.97	0.56	-3.24	0.00118	0.01160	NA	NA
	AT2G38025	726	-1.82	-2.00	0.61	-2.99	0.00277	0.02193	NA	NA
	AT5G41810	483	-1.82	-1.96	0.55	-3.34	0.00083	0.00901	NA	NA
	AT5G53030	314	-1.82	-2.04	0.66	-2.77	0.00560	0.03654	NA	NA
	AT5G48655	1065	-1.82	-1.99	0.59	-3.09	0.00198	0.01706	NA	NA
	AT5G51830	626	-1.83	-2.06	0.69	-2.66	0.00782	0.04629	NA	NA
	AT1G61065	217	-1.83	-1.99	0.59	-3.07	0.00211	0.01794	NA	NA
	AT5G66450	510	-1.83	-2.05	0.67	-2.75	0.00603	0.03854	LPPepsilon2	lipid phosphate phosphatase epsilon 2
	AT2G40935	379	-1.83	-2.07	0.69	-2.64	0.00834	0.04823	NA	NA
	AT4G11010	5339	-1.83	-1.96	0.54	-3.36	0.00077	0.00857	NDPK3	nucleoside diphosphate kinase 3
	AT4G27040	580	-1.83	-2.01	0.61	-2.97	0.00293	0.02298	VPS22	0

Supplemental Table 1-RNA seq data comparing Pro35S::MYB63 with wild type.

DAP.MYB63.target(1.5kb upstream TSS)	Gene	baseMean	log2FoldChange	lfcMLE	lfcSE	stat	pvalue	padj (FDR)	TAIR10 Symbol	TAIR10 Annotation (Short)
	AT2G23810	5376	-1.83	-1.97	0.55	-3.32	0.00089	0.00943	TET8	tetraspanin8
	AT2G05520	58512	-1.83	-1.96	0.54	-3.40	0.00068	0.00783	ATGRP-3	GLYCINE-RICH PROTEIN 3
	AT1G52825	164	-1.83	-2.00	0.60	-3.04	0.00240	0.01982	NA	NA
	AT5G03850	6680	-1.83	-1.98	0.56	-3.26	0.00112	0.01117	NA	NA
	AT4G33040	173	-1.83	-1.99	0.58	-3.18	0.00146	0.01359	NA	NA
	AT2G37035	218	-1.83	-1.96	0.53	-3.47	0.00052	0.00640	NA	NA
	AT5G57170	791	-1.83	-1.99	0.58	-3.18	0.00145	0.01353	NA	NA
	AT1G01620	8634	-1.83	-1.98	0.57	-3.21	0.00132	0.01255	PIP1;3	PLASMA MEMBRANE INTRINSIC PROTEIN 1;3
	AT1G55260	606	-1.83	-1.95	0.52	-3.55	0.00039	0.00519	LTPG6	glycosylphosphatidylinositol-anchored lipid protein transfer 6
	AT1G12970	420	-1.83	-2.00	0.59	-3.12	0.00182	0.01611	PIRL3	plant intracellular ras group-related LRR 3
	AT3G12630	5932	-1.83	-2.03	0.63	-2.89	0.00386	0.02780	SAP5	stress associated protein 5
	AT3G11530	824	-1.83	-1.97	0.54	-3.39	0.00070	0.00796	NA	NA
	AT1G14200	1155	-1.83	-2.01	0.61	-3.02	0.00252	0.02054	NA	NA
	AT4G00895	2656	-1.84	-1.95	0.50	-3.66	0.00025	0.00372	NA	NA
	AT5G02600	446	-1.84	-1.96	0.52	-3.52	0.00043	0.00561	NAKR1	SODIUM POTASSIUM ROOT DEFECTIVE 1
	ATMG01320	65	-1.84	-2.03	0.62	-2.95	0.00315	0.02423	NAD2	NADH DEHYDROGENASE 2
	AT3G55740	521	-1.84	-1.98	0.56	-3.26	0.00110	0.01100	ATPROT2	PROLINE TRANSPORTER 2
	AT3G23480	371	-1.84	-1.99	0.57	-3.25	0.00116	0.01143	NA	NA
	AT3G15900	772	-1.84	-2.04	0.64	-2.88	0.00395	0.02829	NA	NA
	AT2G41945	319	-1.84	-2.07	0.67	-2.74	0.00613	0.03902	NA	NA
	AT1G02340	187	-1.84	-2.01	0.60	-3.05	0.00230	0.01913	FBI1	0
	AT3G60080	507	-1.84	-2.00	0.58	-3.17	0.00150	0.01392	NA	NA
	AT2G16600	23305	-1.84	-1.95	0.50	-3.65	0.00026	0.00383	AtCYP19-1	0
	AT5G47430	625	-1.84	-1.98	0.55	-3.35	0.00079	0.00873	NA	NA
	AT3G59600	2175	-1.84	-1.98	0.55	-3.36	0.00079	0.00868	NRPB8B	0
	AT2G43460	7768	-1.84	-1.98	0.54	-3.42	0.00062	0.00730	NA	NA
	AT2G02180	842	-1.84	-1.98	0.55	-3.35	0.00080	0.00875	TOM3	tobamovirus multiplication protein 3
	AT1G51650	6791	-1.84	-1.97	0.52	-3.54	0.00040	0.00530	NA	NA
	AT3G59900	60	-1.84	-2.08	0.68	-2.73	0.00639	0.04022	ARGOS	AUXIN-REGULATED GENE INVOLVED IN ORGAN SIZE
	AT2G42570	332	-1.84	-1.97	0.53	-3.45	0.00055	0.00669	TBL39	TRICHOME BIREFRINGENCE-LIKE 39
	AT2G36145	720	-1.84	-2.01	0.59	-3.10	0.00194	0.01681	NA	NA
	AT1G01490	1836	-1.84	-1.97	0.52	-3.56	0.00037	0.00494	NA	NA
	AT2G03870	951	-1.84	-1.97	0.52	-3.54	0.00040	0.00522	EMB2816	EMBRYO DEFECTIVE 2816
	AT5G14360	155	-1.84	-2.00	0.57	-3.22	0.00130	0.01241	NA	NA
	AT2G37060	115	-1.84	-2.02	0.60	-3.06	0.00218	0.01838	NF-YB8	nuclear factor Y, subunit B8
	AT3G52770	66	-1.84	-2.04	0.64	-2.90	0.00370	0.02703	ZPR3	LITTLE ZIPPER 3
	AT5G18800	1961	-1.84	-1.97	0.53	-3.51	0.00045	0.00570	NA	NA
	AT1G54580	3237	-1.84	-1.98	0.54	-3.42	0.00064	0.00742	ACP2	acyl carrier protein 2
	AT3G48720	580	-1.84	-1.98	0.54	-3.40	0.00067	0.00769	DCF	DEFICIENT IN CUTIN FERULATE
	AT4G13615	7748	-1.85	-1.98	0.55	-3.38	0.00072	0.00817	NA	NA
	AT3G60210	2270	-1.85	-2.01	0.58	-3.16	0.00158	0.01446	NA	NA
	AT2G37678	650	-1.85	-1.97	0.52	-3.54	0.00039	0.00519	FHY1	far-red elongated hypocotyl 1
	AT1G74520	1236	-1.85	-1.98	0.53	-3.46	0.00053	0.00653	ATHVA22A	HVA22 homologue A
YES	AT3G01980	751	-1.85	-1.99	0.55	-3.36	0.00077	0.00857	NA	NA
	AT2G22500	1147	-1.85	-1.98	0.53	-3.52	0.00044	0.00562	ATPUMP5	PLANT UNCOUPLING MITOCHONDRIAL PROTEIN 5
	AT2G34770	1250	-1.85	-1.98	0.54	-3.43	0.00061	0.00723	ATFAH1	ARABIDOPSIS FATTY ACID HYDROXYLASE 1
	AT5G53940	880	-1.85	-1.99	0.55	-3.38	0.00072	0.00809	NA	NA
	AT3G01830	348	-1.85	-2.05	0.63	-2.92	0.00350	0.02608	NA	NA
	AT1G73790	429	-1.85	-1.99	0.54	-3.46	0.00054	0.00657	AtGIP2	0
	AT1G74458	162	-1.85	-2.04	0.61	-3.02	0.00251	0.02053	NA	NA
	AT4G28770	1254	-1.85	-2.02	0.59	-3.14	0.00171	0.01535	NA	NA
	AT5G13470	561	-1.85	-2.04	0.61	-3.04	0.00239	0.01976	NA	NA
	AT5G53160	1836	-1.86	-1.99	0.54	-3.46	0.00053	0.00653	PYL8	PYR1-like 8
	AT1G69523	196	-1.86	-2.01	0.57	-3.28	0.00103	0.01056	NA	NA
	AT5G17610	677	-1.86	-1.98	0.52	-3.55	0.00038	0.00512	NA	NA
	AT1G30380	92127	-1.86	-2.00	0.55	-3.38	0.00072	0.00809	PSAK	photosystem I subunit K
	AT4G21980	1818	-1.86	-1.99	0.53	-3.51	0.00044	0.00564	APG8A	AUTOPHAGY 8A
	AT3G16100	1068	-1.86	-1.98	0.52	-3.61	0.00031	0.00437	ATRAB7D	0
	AT1G22520	868	-1.86	-2.00	0.55	-3.36	0.00077	0.00857	NA	NA
	AT1G26800	989	-1.86	-2.01	0.55	-3.36	0.00078	0.00865	NA	NA
	AT1G10550	69	-1.86	-2.11	0.69	-2.70	0.00687	0.04242	XET	XYLOGLUCAN:XYLOGLUCOSYL TRANSFERASE 33
	AT4G29190	6833	-1.86	-2.00	0.54	-3.43	0.00061	0.00720	AtC3H49	0
	AT2G13820	458	-1.86	-2.00	0.54	-3.48	0.00051	0.00631	AtXYP2	0

Supplemental Table 1-RNA seq data comparing Pro35S::MYB63 with wild type.

DAP.MYB63.target(1.5kb upstream TSS)	Gene	baseMean	log2FoldChange	lfcMLE	lfcSE	stat	pvalue	padj (FDR)	TAIR10 Symbol	TAIR10 Annotation (Short)
	AT4G04200	452	-1.86	-2.00	0.54	-3.43	0.00060	0.00717	NA	NA
	AT5G40460	98	-1.86	-2.13	0.71	-2.64	0.00830	0.04814	NA	NA
	AT2G47690	2497	-1.86	-2.00	0.55	-3.42	0.00063	0.00741	NA	NA
	AT1G63220	282	-1.86	-1.99	0.53	-3.51	0.00044	0.00566	NA	NA
	AT4G15680	618	-1.86	-2.00	0.53	-3.49	0.00048	0.00607	NA	NA
	AT5G19510	11480	-1.86	-2.10	0.67	-2.77	0.00563	0.03669	NA	NA
	AT5G23820	2627	-1.87	-2.00	0.54	-3.47	0.00052	0.00643	ML3	MD2-related lipid recognition 3
	AT1G22270	1906	-1.87	-1.99	0.52	-3.58	0.00035	0.00479	NA	NA
	AT3G62070	106	-1.87	-2.06	0.62	-3.00	0.00272	0.02165	NA	NA
	AT4G11370	334	-1.87	-2.00	0.54	-3.47	0.00052	0.00639	RHA1A	RING-H2 finger A1A
	AT5G57040	4753	-1.87	-2.03	0.58	-3.23	0.00124	0.01200	NA	NA
	ATCG00150	244	-1.87	-2.10	0.67	-2.79	0.00522	0.03483	ATPI	0
	AT1G68310	839	-1.87	-2.00	0.53	-3.51	0.00044	0.00564	AE7	AS1/2 ENHANCER7
	AT2G17230	1700	-1.87	-2.00	0.53	-3.54	0.00040	0.00523	EXL5	EXORDIUM like 5
	AT1G30250	92	-1.87	-2.05	0.60	-3.11	0.00184	0.01622	NA	NA
	AT2G14285	423	-1.87	-2.03	0.58	-3.23	0.00123	0.01195	NA	NA
	AT5G40690	352	-1.87	-2.02	0.57	-3.31	0.00094	0.00981	NA	NA
	AT1G34350	449	-1.87	-2.00	0.52	-3.56	0.00037	0.00496	NA	NA
	AT5G67350	309	-1.87	-2.07	0.64	-2.93	0.00343	0.02573	NA	NA
	AT2G33585	179	-1.87	-2.14	0.71	-2.65	0.00814	0.04761	NA	NA
	AT5G43970	2936	-1.87	-2.02	0.55	-3.38	0.00074	0.00828	ATTOM22-\	0
	AT2G33510	276	-1.87	-2.02	0.56	-3.33	0.00088	0.00940	NA	NA
	AT4G34881	61	-1.87	-2.13	0.70	-2.67	0.00748	0.04494	NA	NA
	AT5G60200	233	-1.87	-2.03	0.58	-3.25	0.00115	0.01141	TMO6	TARGET OF MONOPTEROS 6
	AT1G65290	3697	-1.87	-2.00	0.53	-3.55	0.00038	0.00507	mtACP2	mitochondrial acyl carrier protein 2
	AT4G18590	600	-1.87	-2.01	0.54	-3.45	0.00056	0.00675	NA	NA
	AT2G16060	275	-1.88	-2.09	0.65	-2.87	0.00412	0.02919	AHB1	hemoglobin 1
	AT5G41960	398	-1.88	-2.03	0.56	-3.32	0.00090	0.00953	NA	NA
	AT1G77370	2071	-1.88	-2.03	0.57	-3.29	0.00102	0.01042	NA	NA
	AT2G39720	1096	-1.88	-2.04	0.58	-3.26	0.00113	0.01126	RHC2A	RING-H2 finger C2A
	AT3G51660	3999	-1.88	-2.01	0.52	-3.60	0.00032	0.00443	NA	NA
	AT5G64880	733	-1.88	-2.05	0.59	-3.20	0.00139	0.01305	NA	NA
	AT2G24290	703	-1.88	-2.01	0.52	-3.61	0.00031	0.00436	NA	NA
	AT1G07170	761	-1.88	-2.02	0.54	-3.46	0.00054	0.00660	NA	NA
	AT1G56045	8776	-1.88	-2.02	0.54	-3.49	0.00049	0.00611	NA	NA
	AT2G32150	590	-1.89	-2.02	0.53	-3.57	0.00036	0.00490	NA	NA
	AT1G07950	708	-1.89	-2.07	0.60	-3.12	0.00180	0.01601	MED22B	0
	AT5G24314	2948	-1.89	-2.03	0.54	-3.48	0.00050	0.00623	PDE225	PIGMENT DEFECTIVE 225
	AT1G30890	666	-1.89	-2.04	0.55	-3.41	0.00064	0.00745	NA	NA
	AT4G34560	198	-1.89	-2.11	0.66	-2.87	0.00408	0.02900	NA	NA
	AT4G11521	242	-1.89	-2.11	0.65	-2.92	0.00353	0.02620	NA	NA
	AT3G07568	1370	-1.89	-2.04	0.55	-3.42	0.00064	0.00742	NA	NA
	AT5G57910	203	-1.89	-2.05	0.57	-3.32	0.00090	0.00952	NA	NA
	AT4G38680	6285	-1.89	-2.02	0.53	-3.57	0.00035	0.00481	ATCSP2	ARABIDOPSIS THALIANA COLD SHOCK PROTEIN 2
	AT3G01130	1941	-1.89	-2.04	0.55	-3.45	0.00056	0.00675	NA	NA
	AT5G58570	370	-1.89	-2.11	0.65	-2.89	0.00382	0.02761	NA	NA
	AT5G22920	4705	-1.90	-2.03	0.53	-3.56	0.00037	0.00500	NA	NA
	AT3G48030	816	-1.90	-2.05	0.56	-3.39	0.00069	0.00793	NA	NA
	AT3G10080	993	-1.90	-2.10	0.63	-3.00	0.00272	0.02165	NA	NA
	AT3G08920	2134	-1.90	-2.05	0.56	-3.41	0.00066	0.00765	NA	NA
	AT5G15802	564	-1.90	-2.05	0.57	-3.34	0.00083	0.00902	NA	NA
	AT4G02725	1342	-1.90	-2.03	0.54	-3.53	0.00042	0.00546	NA	NA
	AT4G10300	3101	-1.90	-2.03	0.53	-3.60	0.00032	0.00444	NA	NA
	AT2G46390	5382	-1.90	-2.06	0.58	-3.27	0.00108	0.01086	SDH8	succinate dehydrogenase 8
	AT4G18800	899	-1.90	-2.05	0.57	-3.33	0.00087	0.00930	ATHSGBP	0
	AT3G07195	268	-1.90	-2.04	0.55	-3.44	0.00059	0.00707	NA	NA
	AT4G01410	512	-1.90	-2.04	0.54	-3.51	0.00044	0.00565	NA	NA
	AT3G24770	1086	-1.90	-2.09	0.62	-3.07	0.00215	0.01820	CLE41	CLAVATA3/ESR-RELATED 41
	AT5G57560	7042	-1.90	-2.08	0.60	-3.16	0.00160	0.01459	TCH4	Touch 4
	AT3G25717	905	-1.90	-2.08	0.59	-3.21	0.00135	0.01279	DVL6	DEVIL 6
	AT4G39800	849	-1.90	-2.01	0.49	-3.88	0.00010	0.00192	ATIPS1	INOSITOL 3-PHOSPHATE SYNTHASE 1
	AT1G45010	273	-1.90	-2.05	0.55	-3.44	0.00059	0.00705	NA	NA
	AT4G34770	522	-1.90	-2.06	0.57	-3.36	0.00078	0.00860	SAUR1	SMALL AUXIN UPREGULATED RNA 1
	AT5G65925	190	-1.90	-2.05	0.55	-3.47	0.00052	0.00645	NA	NA
	AT1G43790	253	-1.91	-2.06	0.56	-3.43	0.00061	0.00725	TED6	tracheary element differentiation-related 6
	AT1G31335	367	-1.91	-2.06	0.56	-3.39	0.00070	0.00793	NA	NA
	AT2G31090	642	-1.91	-2.08	0.58	-3.28	0.00105	0.01065	NA	NA
	AT4G40042	707	-1.91	-2.07	0.56	-3.39	0.00070	0.00799	NA	NA
	AT1G52740	7639	-1.91	-2.05	0.53	-3.60	0.00032	0.00441	HTA9	histone H2A protein 9

Supplemental Table 1-RNA seq data comparing Pro35S::MYB63 with wild type.

DAP.MYB63.target(1.5kb upstream TSS)	Gene	baseMean	log2FoldChange	lfcMLE	lfcSE	stat	pvalue	padj (FDR)	TAIR10 Symbol	TAIR10 Annotation (Short)
	AT5G19151	130	-1.91	-2.08	0.58	-3.31	0.00094	0.00985	NA	NA
	AT2G21600	2367	-1.91	-2.04	0.52	-3.69	0.00023	0.00345	ATRER1B	endoplasmatic reticulum retrieval protein 1B
	AT1G23710	831	-1.92	-2.05	0.52	-3.68	0.00024	0.00352	NA	NA
	AT2G24395	897	-1.92	-2.07	0.56	-3.44	0.00058	0.00699	NA	NA
	AT3G20510	1096	-1.92	-2.07	0.56	-3.42	0.00063	0.00740	NA	NA
	AT2G29995	277	-1.92	-2.11	0.62	-3.09	0.00202	0.01736	NA	NA
	AT2G02050	2880	-1.92	-2.11	0.61	-3.12	0.00178	0.01590	NA	NA
	AT3G27340	536	-1.92	-2.11	0.61	-3.12	0.00179	0.01594	NA	NA
	AT4G18372	221	-1.92	-2.09	0.58	-3.28	0.00102	0.01046	NA	NA
	AT1G74270	2830	-1.92	-2.06	0.54	-3.56	0.00037	0.00500	NA	NA
	AT5G03500	311	-1.92	-2.11	0.62	-3.12	0.00182	0.01611	NA	NA
	AT4G25225	275	-1.92	-2.08	0.57	-3.37	0.00076	0.00849	NA	NA
	AT5G24890	2402	-1.92	-2.08	0.57	-3.37	0.00075	0.00840	NA	NA
	AT4G04630	300	-1.92	-2.06	0.53	-3.61	0.00031	0.00435	NA	NA
	AT5G46720	79	-1.92	-2.13	0.64	-3.03	0.00247	0.02033	NA	NA
	AT2G45180	94205	-1.92	-2.06	0.54	-3.57	0.00036	0.00490	NA	NA
	AT2G20890	15049	-1.92	-2.09	0.58	-3.30	0.00096	0.00994	PSB29	photosystem II reaction center PSB29 protein
YES	AT4G36800	4511	-1.92	-2.08	0.56	-3.44	0.00059	0.00707	RCE1	RUB1 conjugating enzyme 1
	AT5G02060	72	-1.93	-2.16	0.66	-2.91	0.00359	0.02653	NA	NA
	AT1G45474	5105	-1.93	-2.12	0.61	-3.13	0.00172	0.01545	Lhca5	photosystem I light harvesting complex gene 5
	AT3G28080	901	-1.93	-2.11	0.60	-3.19	0.00141	0.01317	UMAMIT47	Usually multiple acids move in and out Transporters 47
	AT1G02610	1129	-1.93	-2.07	0.55	-3.52	0.00042	0.00550	NA	NA
	AT5G57340	566	-1.93	-2.07	0.54	-3.54	0.00040	0.00530	NA	NA
	AT3G28940	7622	-1.93	-2.06	0.53	-3.65	0.00026	0.00383	NA	NA
	AT2G35810	557	-1.93	-2.08	0.57	-3.41	0.00065	0.00754	NA	NA
	AT2G30942	237	-1.93	-2.10	0.59	-3.25	0.00116	0.01146	NA	NA
	AT3G27880	483	-1.93	-2.08	0.55	-3.51	0.00044	0.00564	NA	NA
	AT3G03590	338	-1.93	-2.15	0.65	-2.99	0.00278	0.02200	NA	NA
	AT2G36220	2427	-1.93	-2.07	0.53	-3.67	0.00025	0.00364	NA	NA
	AT5G22390	1072	-1.93	-2.06	0.51	-3.76	0.00017	0.00274	NA	NA
	AT4G20870	1469	-1.93	-2.07	0.54	-3.61	0.00031	0.00434	ATFAH2	ARABIDOPSIS FATTY ACID HYDROXYLASE 2
	AT3G18710	167	-1.93	-2.12	0.61	-3.17	0.00152	0.01404	ATPUB29	ARABIDOPSIS THALIANA PLANT U-BOX 29
	AT5G41650	69	-1.94	-2.16	0.65	-2.97	0.00294	0.02302	NA	NA
	AT2G03710	64	-1.94	-2.14	0.62	-3.11	0.00189	0.01651	AGL3	AGAMOUS-like 3
YES	AT3G04790	11582	-1.94	-2.11	0.57	-3.39	0.00069	0.00791	EMB3119	EMBRYO DEFECTIVE 3119
YES	AT3G61980	466	-1.94	-2.11	0.58	-3.37	0.00074	0.00830	NA	NA
	AT1G10500	1949	-1.94	-2.25	0.73	-2.65	0.00804	0.04720	ATCPISCA	chloroplast-localized ISCA-like protein
	AT2G18240	212	-1.94	-2.13	0.61	-3.20	0.00139	0.01307	NA	NA
	AT3G62790	1900	-1.94	-2.07	0.52	-3.77	0.00017	0.00272	NA	NA
	AT1G61780	1897	-1.94	-2.08	0.54	-3.61	0.00031	0.00434	NA	NA
	ATCG00470	55969	-1.94	-2.12	0.59	-3.29	0.00099	0.01020	ATPE	ATP synthase epsilon chain
	AT1G65510	169	-1.94	-2.17	0.66	-2.96	0.00306	0.02370	NA	NA
	AT4G02080	3929	-1.95	-2.08	0.53	-3.65	0.00026	0.00384	ASAR1	0
	AT1G23730	69	-1.95	-2.25	0.72	-2.68	0.00727	0.04410	ATBCA3	BETA CARBONIC ANHYDRASE 3
	AT5G23280	2051	-1.95	-2.10	0.56	-3.48	0.00049	0.00619	NA	NA
YES	AT5G47050	254	-1.95	-2.08	0.53	-3.69	0.00023	0.00344	NA	NA
	AT1G01470	3107	-1.95	-2.07	0.50	-3.88	0.00011	0.00193	LEA14	LATE EMBRYOGENESIS ABUNDANT 14
	AT2G23090	13186	-1.95	-2.09	0.53	-3.65	0.00026	0.00379	NA	NA
	AT5G13100	1363	-1.95	-2.11	0.58	-3.39	0.00071	0.00805	NA	NA
	AT1G75770	136	-1.95	-2.13	0.60	-3.24	0.00119	0.01167	NA	NA
	AT4G35950	1060	-1.95	-2.14	0.62	-3.17	0.00154	0.01417	ARAC6	RAC-like 6
	AT4G28025	1868	-1.95	-2.08	0.52	-3.75	0.00017	0.00279	NA	NA
	AT1G66230	191	-1.95	-2.08	0.52	-3.75	0.00017	0.00280	AtMYB20	myb domain protein 20
	AT5G14710	192	-1.95	-2.12	0.58	-3.34	0.00083	0.00902	NA	NA
	AT2G20480	28	-1.95	-2.26	0.74	-2.66	0.00791	0.04665	NA	NA
	AT5G01520	484	-1.95	-2.15	0.62	-3.16	0.00156	0.01429	AIRP2	ABA Insensitive RING Protein 2
	AT1G03610	1266	-1.95	-2.09	0.52	-3.73	0.00019	0.00300	NA	NA
	AT5G45340	256	-1.95	-2.10	0.55	-3.58	0.00034	0.00468	CYP707A3	cytochrome P450, family 707, subfamily A, polypeptide 3
	AT3G62950	1426	-1.96	-2.08	0.51	-3.83	0.00013	0.00225	NA	NA
	AT5G44575	213	-1.96	-2.12	0.57	-3.40	0.00067	0.00774	NA	NA
	AT2G22980	627	-1.96	-2.19	0.66	-2.95	0.00318	0.02435	SCPL13	serine carboxypeptidase-like 13
	AT1G73650	1655	-1.96	-2.09	0.53	-3.71	0.00021	0.00321	NA	NA

Supplemental Table 1-RNA seq data comparing Pro35S::MYB63 with wild type.

DAP.MYB63.target(1.5kb upstream TSS)	Gene	baseMean	log2FoldChange	lfcMLE	lfcSE	stat	pvalue	padj (FDR)	TAIR10 Symbol	TAIR10 Annotation (Short)
	AT2G16740	347	-1.96	-2.14	0.60	-3.27	0.00108	0.01089	UBC29	ubiquitin-conjugating enzyme 29
	AT2G22425	960	-1.96	-2.13	0.58	-3.36	0.00077	0.00853	NA	NA
	AT1G54650	200	-1.96	-2.13	0.58	-3.39	0.00071	0.00805	NA	NA
	AT3G11500	1371	-1.96	-2.09	0.52	-3.75	0.00017	0.00280	NA	NA
	AT4G24275	255	-1.96	-2.17	0.63	-3.10	0.00194	0.01681	NA	NA
	AT1G70490	8016	-1.96	-2.10	0.54	-3.66	0.00025	0.00368	ARFA1D	0
	AT3G01210	308	-1.96	-2.16	0.62	-3.19	0.00142	0.01327	NA	NA
	AT3G27770	2002	-1.97	-2.27	0.73	-2.71	0.00677	0.04197	NA	NA
	AT1G64750	2502	-1.97	-2.11	0.54	-3.61	0.00031	0.00434	ATDSS1(I)	deletion of SUV3 suppressor 1(I)
	AT4G31310	638	-1.97	-2.15	0.60	-3.27	0.00108	0.01087	NA	NA
	AT2G44940	222	-1.97	-2.15	0.60	-3.26	0.00111	0.01110	NA	NA
	AT2G38870	2490	-1.97	-2.10	0.52	-3.76	0.00017	0.00275	NA	NA
	AT4G11360	2524	-1.97	-2.11	0.54	-3.65	0.00026	0.00379	RHA1B	RING-H2 finger A1B
	AT4G12040	2540	-1.97	-2.13	0.57	-3.47	0.00052	0.00640	AtSAP7	0
	AT1G22740	359	-1.97	-2.11	0.54	-3.67	0.00024	0.00361	ATRABG3B	0
	AT3G52560	3354	-1.97	-2.12	0.54	-3.62	0.00030	0.00424	MMZ4	MMS2 ZWEI HOMOLOGUE 4
	AT5G59300	1213	-1.97	-2.10	0.53	-3.74	0.00018	0.00288	ATUBC7	ARABIDOPSIS THALIANA UBIQUITIN CARRIER PROTEIN 7
	AT2G36835	1821	-1.97	-2.11	0.54	-3.67	0.00024	0.00362	NA	NA
	AT5G54148	45	-1.97	-2.27	0.72	-2.73	0.00628	0.03969	NA	NA
	AT4G08700	300	-1.97	-2.13	0.57	-3.49	0.00049	0.00611	ATPUP13	0
	AT3G09940	71	-1.97	-2.15	0.58	-3.38	0.00072	0.00813	ATMDAR3	ARABIDOPSIS THALIANA MONODEHYDROASCORBATE REDUCTASE 3
	AT2G36120	1011	-1.97	-2.15	0.58	-3.39	0.00069	0.00789	DOT1	DEFECTIVELY ORGANIZED TRIBUTARIES 1
	AT3G26900	2061	-1.97	-2.16	0.59	-3.32	0.00090	0.00952	ATSKL1	Arabidopsis thaliana shikimate kinase-like 1
	AT5G59613	7308	-1.97	-2.12	0.54	-3.63	0.00029	0.00409	NA	NA
	AT1G71880	1318	-1.98	-2.18	0.63	-3.13	0.00176	0.01571	ATSUC1	ARABIDOPSIS THALIANA SUCROSE-PROTON SYMPORTER 1
	AT3G48550	284	-1.98	-2.15	0.59	-3.35	0.00082	0.00890	NA	NA
	AT1G75690	6260	-1.98	-2.10	0.50	-3.97	0.00007	0.00141	LQY1	LOW QUANTUM YIELD OF PHOTOSYSTEM II 1
	AT5G54490	814	-1.98	-2.17	0.61	-3.22	0.00129	0.01238	PBP1	pinoid-binding protein 1
	AT3G49100	330	-1.98	-2.23	0.68	-2.91	0.00357	0.02643	NA	NA
	AT1G08480	2954	-1.98	-2.14	0.57	-3.48	0.00050	0.00626	SDH6	succinate dehydrogenase 6
	AT1G78040	20931	-1.98	-2.10	0.50	-3.93	0.00009	0.00163	NA	NA
	AT5G49540	832	-1.98	-2.15	0.58	-3.39	0.00069	0.00791	NA	NA
	AT3G63540	3655	-1.98	-2.12	0.52	-3.77	0.00016	0.00266	NA	NA
	AT3G47820	626	-1.98	-2.18	0.62	-3.21	0.00131	0.01252	PUB39	PLANT U-BOX 39
	AT3G46000	6014	-1.98	-2.14	0.56	-3.53	0.00042	0.00549	ADF2	actin depolymerizing factor 2
	AT1G14687	97	-1.98	-2.22	0.67	-2.97	0.00293	0.02298	AtHB32	homeobox protein 32
	AT1G20430	1240	-1.98	-2.21	0.65	-3.04	0.00238	0.01970	NA	NA
	AT5G62140	650	-1.98	-2.13	0.54	-3.64	0.00027	0.00395	NA	NA
	AT4G28150	93	-1.98	-2.23	0.68	-2.93	0.00334	0.02522	NA	NA
	AT1G65500	511	-1.98	-2.12	0.54	-3.70	0.00022	0.00337	NA	NA
	AT2G24860	1395	-1.98	-2.11	0.52	-3.85	0.00012	0.00211	NA	NA
	AT1G72930	6215	-1.98	-2.12	0.53	-3.73	0.00020	0.00305	AtTN10	0
	AT2G37750	103	-1.98	-2.30	0.74	-2.69	0.00721	0.04392	NA	NA
	AT5G08320	646	-1.98	-2.13	0.54	-3.68	0.00024	0.00355	NA	NA
	AT1G29420	93	-1.99	-2.29	0.72	-2.74	0.00607	0.03874	SAUR61	SMALL AUXIN UPREGULATED RNA 61
	AT2G17972	754	-1.99	-2.13	0.53	-3.73	0.00019	0.00305	NA	NA
	AT5G44568	1444	-1.99	-2.14	0.55	-3.62	0.00030	0.00422	NA	NA
	AT2G37970	622	-1.99	-2.15	0.57	-3.52	0.00044	0.00564	AtHBP2	0
	AT1G23130	10239	-1.99	-2.14	0.56	-3.57	0.00035	0.00481	NA	NA
	AT4G10360	794	-1.99	-2.15	0.57	-3.52	0.00044	0.00564	NA	NA
	AT1G20696	3607	-1.99	-2.16	0.59	-3.39	0.00069	0.00790	HMGB3	high mobility group B3
	AT3G23050	2384	-1.99	-2.12	0.52	-3.79	0.00015	0.00254	AXR2	AUXIN RESISTANT 2
	AT1G32410	615	-1.99	-2.18	0.60	-3.32	0.00090	0.00957	NA	NA
	AT3G16140	31644	-1.99	-2.30	0.73	-2.73	0.00636	0.04008	PSAH-1	photosystem I subunit H-1
	AT1G71950	3323	-1.99	-2.15	0.56	-3.55	0.00039	0.00516	NA	NA
	AT1G68680	1386	-1.99	-2.15	0.55	-3.60	0.00032	0.00448	NA	NA
	AT1G60010	1845	-1.99	-2.12	0.52	-3.86	0.00011	0.00206	NA	NA
	AT2G16630	220	-1.99	-2.13	0.52	-3.84	0.00012	0.00216	NA	NA
	AT4G33780	626	-1.99	-2.12	0.52	-3.87	0.00011	0.00199	NA	NA
	AT2G30395	34	-1.99	-2.28	0.71	-2.81	0.00490	0.03312	ATOFP17	0
	AT5G42146	231	-1.99	-2.19	0.61	-3.27	0.00106	0.01077	NA	NA
	AT1G25400	792	-1.99	-2.12	0.51	-3.89	0.00010	0.00187	NA	NA
	AT3G59840	2132	-2.00	-2.14	0.53	-3.73	0.00019	0.00299	NA	NA
	AT4G12980	3093	-2.00	-2.20	0.63	-3.18	0.00146	0.01362	NA	NA

Supplemental Table 1-RNA seq data comparing Pro35S::MYB63 with wild type.

DAP MYB63.target(1.5kb upstream TSS)	Gene	baseMean	log2FoldChange	lfcMLE	lfcSE	stat	pvalue	padj (FDR)	TAIR10 Symbol	TAIR10 Annotation (Short)
	AT3G62530	7521	-2.00	-2.27	0.70	-2.84	0.00445	0.03085	NA	NA
	AT1G08180	323	-2.00	-2.20	0.62	-3.25	0.00117	0.01152	NA	NA
	AT5G65430	5014	-2.00	-2.16	0.56	-3.56	0.00037	0.00499	14-3-3KAPP	14-3-3 PROTEIN G-BOX FACTOR14 KAPPA
	AT1G61470	119	-2.00	-2.18	0.60	-3.34	0.00083	0.00901	NA	NA
	AT4G16442	489	-2.00	-2.15	0.55	-3.63	0.00028	0.00401	NA	NA
	AT3G12260	4727	-2.00	-2.14	0.54	-3.72	0.00020	0.00307	NA	NA
	AT5G03460	1482	-2.00	-2.14	0.53	-3.74	0.00019	0.00294	NA	NA
	AT3G06750	1410	-2.00	-2.15	0.54	-3.70	0.00022	0.00332	NA	NA
	AT2G01505	136	-2.00	-2.30	0.72	-2.78	0.00539	0.03562	CLE16	CLAVATA3/ESR-RELATED 16
	AT2G32560	989	-2.00	-2.13	0.52	-3.86	0.00011	0.00200	NA	NA
	AT5G52760	1272	-2.00	-2.20	0.61	-3.25	0.00114	0.01135	NA	NA
	AT2G21185	1197	-2.00	-2.16	0.56	-3.56	0.00038	0.00504	NA	NA
	AT1G15885	272	-2.00	-2.19	0.59	-3.37	0.00075	0.00840	NA	NA
	AT2G27130	463	-2.00	-2.14	0.53	-3.80	0.00014	0.00243	NA	NA
	AT4G30996	1911	-2.00	-2.16	0.55	-3.63	0.00028	0.00405	NKS1	NA(+)- AND K(+)-SENSITIVE 1
	AT3G48570	624	-2.00	-2.17	0.56	-3.55	0.00038	0.00507	NA	NA
	AT3G54290	821	-2.00	-2.15	0.54	-3.69	0.00023	0.00345	NA	NA
	AT3G10860	6083	-2.00	-2.16	0.55	-3.63	0.00028	0.00401	NA	NA
	AT2G01850	4851	-2.00	-2.17	0.56	-3.55	0.00038	0.00507	ATXTH27	0
	AT5G07960	1462	-2.00	-2.18	0.58	-3.46	0.00054	0.00658	NA	NA
	AT5G52120	89	-2.00	-2.18	0.59	-3.41	0.00065	0.00751	AtPP2-A14	phloem protein 2-A14
	AT2G44920	3036	-2.00	-2.16	0.55	-3.61	0.00030	0.00426	NA	NA
	AT5G14105	505	-2.01	-2.20	0.60	-3.33	0.00087	0.00933	NA	NA
	AT2G14460	172	-2.01	-2.20	0.60	-3.33	0.00087	0.00933	NA	NA
	AT5G18250	443	-2.01	-2.17	0.57	-3.51	0.00045	0.00575	NA	NA
	AT2G28710	120	-2.01	-2.27	0.68	-2.93	0.00337	0.02538	NA	NA
	AT3G22950	842	-2.01	-2.18	0.58	-3.48	0.00050	0.00626	ARFC1	ADP-ribosylation factor C1
	AT4G38920	8486	-2.01	-2.15	0.53	-3.79	0.00015	0.00254	ATVHA-C3	vacuolar-type H(+)-ATPase C3
	AT3G62880	321	-2.01	-2.20	0.60	-3.32	0.00089	0.00943	ATOEP16-4	0
	AT2G20740	1290	-2.01	-2.18	0.57	-3.52	0.00044	0.00564	NA	NA
	AT1G21010	489	-2.01	-2.26	0.67	-2.98	0.00286	0.02249	NA	NA
	AT1G01120	329	-2.01	-2.14	0.51	-3.91	0.00009	0.00173	KCS1	3-ketoacyl-CoA synthase 1
	AT2G45710	1045	-2.01	-2.18	0.57	-3.53	0.00041	0.00534	NA	NA
	AT4G31875	99	-2.01	-2.25	0.66	-3.06	0.00220	0.01855	NA	NA
	AT4G17085	473	-2.01	-2.19	0.59	-3.44	0.00058	0.00699	NA	NA
	AT1G76860	1341	-2.01	-2.16	0.54	-3.72	0.00020	0.00313	LSM3B	SM-like 3B
	AT5G05440	1631	-2.01	-2.24	0.64	-3.13	0.00177	0.01586	PYL5	PYRABACTIN RESISTANCE 1-LIKE 5
	AT1G09645	1182	-2.01	-2.17	0.55	-3.65	0.00026	0.00381	NA	NA
	AT1G57980	203	-2.02	-2.21	0.61	-3.33	0.00087	0.00935	NA	NA
	AT3G51600	20223	-2.02	-2.17	0.55	-3.65	0.00026	0.00383	LTP5	lipid transfer protein 5
	AT1G13280	1101	-2.02	-2.21	0.60	-3.35	0.00081	0.00883	AOC4	allene oxide cyclase 4
	AT5G19370	3250	-2.02	-2.22	0.62	-3.23	0.00124	0.01199	NA	NA
	AT1G16430	228	-2.02	-2.21	0.61	-3.32	0.00091	0.00962	NA	NA
	AT5G47455	474	-2.02	-2.22	0.62	-3.25	0.00116	0.01143	NA	NA
	AT2G34585	2262	-2.02	-2.18	0.56	-3.61	0.00030	0.00430	NA	NA
	AT2G01990	241	-2.02	-2.18	0.57	-3.55	0.00039	0.00516	NA	NA
	AT1G18680	279	-2.02	-2.17	0.55	-3.67	0.00025	0.00366	NA	NA
	AT4G39235	293	-2.02	-2.23	0.63	-3.22	0.00130	0.01243	NA	NA
	AT5G67180	120	-2.02	-2.22	0.62	-3.27	0.00106	0.01077	TOE3	target of early activation tagged (EAT) 3
	AT5G05690	5934	-2.02	-2.17	0.54	-3.75	0.00018	0.00287	CBB3	CABBAGE 3
	AT3G23605	428	-2.02	-2.27	0.68	-2.98	0.00286	0.02249	NA	NA
	AT2G34160	674	-2.02	-2.17	0.54	-3.76	0.00017	0.00277	NA	NA
	AT1G12400	244	-2.02	-2.22	0.61	-3.32	0.00092	0.00964	NA	NA
	AT1G62370	45	-2.02	-2.30	0.70	-2.87	0.00411	0.02916	NA	NA
	AT4G14100	346	-2.02	-2.27	0.67	-3.02	0.00255	0.02074	NA	NA
	AT4G14430	1613	-2.02	-2.19	0.57	-3.55	0.00038	0.00512	ATECI2	ARABIDOPSIS THALIANA DELTA(3), DELTA(2)-ENOYL COA ISOMERASE 2
	AT1G72940	363	-2.02	-2.18	0.55	-3.69	0.00023	0.00345	NA	NA
	AT4G10330	379	-2.02	-2.17	0.54	-3.72	0.00020	0.00315	NA	NA
	AT4G36570	342	-2.02	-2.21	0.60	-3.40	0.00067	0.00774	ATRL3	RAD-like 3
	AT2G01930	1473	-2.02	-2.22	0.61	-3.33	0.00086	0.00929	ATBPC1	BASIC PENTACYSTEINE1
	AT3G01390	5042	-2.02	-2.18	0.54	-3.72	0.00020	0.00312	AVMA10	0
	AT1G79070	200	-2.03	-2.31	0.71	-2.87	0.00412	0.02919	NA	NA
	AT4G12690	143	-2.03	-2.36	0.74	-2.73	0.00641	0.04031	NA	NA
	AT5G03440	373	-2.03	-2.21	0.59	-3.42	0.00063	0.00739	NA	NA
	AT5G66005	304	-2.03	-2.24	0.63	-3.23	0.00125	0.01207	NA	NA
	AT3G11510	10401	-2.03	-2.18	0.54	-3.77	0.00016	0.00267	NA	NA
	AT5G09990	129	-2.03	-2.22	0.61	-3.35	0.00080	0.00873	PROPEP5	elicitor peptide 5 precursor
	AT5G14320	14779	-2.03	-2.18	0.54	-3.74	0.00018	0.00290	EMB3137	EMBRYO DEFECTIVE 3137

Supplemental Table 1-RNA seq data comparing Pro35S::MYB63 with wild type.

DAP MYB63.target(1.5kb upstream TSS)	Gene	baseMean	log2FoldChange	lfcMLE	lfcSE	stat	pvalue	padj (FDR)	TAIR10 Symbol	TAIR10 Annotation (Short)
	AT2G21960	4065	-2.03	-2.24	0.62	-3.26	0.00111	0.01110	NA	NA
	AT1G07770	12336	-2.03	-2.17	0.53	-3.87	0.00011	0.00196	RPS15A	ribosomal protein S15A
	AT3G62920	157	-2.03	-2.23	0.60	-3.37	0.00075	0.00839	NA	NA
	AT2G15970	7575	-2.03	-2.20	0.56	-3.63	0.00028	0.00402	ATCOR413-	ARABIDOPSIS THALIANA COLD-REGULATED413 PLASMA MEMBRANE 1
	AT4G15930	1724	-2.03	-2.18	0.54	-3.76	0.00017	0.00274	NA	NA
	AT4G17790	1171	-2.04	-2.19	0.55	-3.69	0.00022	0.00342	NA	NA
	AT3G48140	21622	-2.04	-2.17	0.51	-3.98	0.00007	0.00140	NA	NA
	AT1G53030	577	-2.04	-2.23	0.60	-3.39	0.00070	0.00794	NA	NA
	AT1G55190	393	-2.04	-2.19	0.55	-3.69	0.00023	0.00345	PRA1.F2	PRENYLATED RAB ACCEPTOR 1.F2
	AT3G26740	35421	-2.04	-2.20	0.56	-3.65	0.00026	0.00379	CCL	CCR-like
	AT3G08710	814	-2.04	-2.23	0.60	-3.42	0.00062	0.00735	ATH9	thioredoxin H-type 9
	AT1G76960	5034	-2.04	-2.21	0.58	-3.51	0.00045	0.00568	NA	NA
	AT5G64130	4560	-2.04	-2.18	0.54	-3.81	0.00014	0.00241	NA	NA
	AT1G15860	1278	-2.04	-2.21	0.58	-3.52	0.00043	0.00559	NA	NA
	AT1G75335	134	-2.04	-2.23	0.60	-3.42	0.00062	0.00730	NA	NA
	AT3G08600	417	-2.04	-2.39	0.76	-2.69	0.00723	0.04398	NA	NA
	AT5G18970	305	-2.04	-2.26	0.63	-3.23	0.00122	0.01193	NA	NA
	AT2G38080	52	-2.04	-2.26	0.64	-3.19	0.00142	0.01329	ATLMCO4	ARABIDOPSIS LACCASE-LIKE MULTICOPPER OXIDASE 4
	AT5G02280	824	-2.04	-2.28	0.66	-3.09	0.00198	0.01706	NA	NA
	AT3G28007	55	-2.04	-2.29	0.66	-3.08	0.00206	0.01765	AtSWEET4	0
	AT1G03430	556	-2.04	-2.36	0.73	-2.78	0.00536	0.03549	AHP5	histidine-containing phosphotransfer factor 5
	AT5G27700	9190	-2.04	-2.22	0.58	-3.53	0.00041	0.00535	NA	NA
	AT4G38860	2520	-2.04	-2.24	0.61	-3.34	0.00083	0.00900	SAUR16	SMALL AUXIN UPREGULATED RNA 16
	AT1G03410	504	-2.04	-2.20	0.54	-3.76	0.00017	0.00275	2A6	0
	AT4G14550	145	-2.05	-2.28	0.65	-3.15	0.00163	0.01483	IAA14	indole-3-acetic acid inducible 14
	AT5G47700	8186	-2.05	-2.21	0.57	-3.62	0.00030	0.00422	NA	NA
	AT2G43810	916	-2.05	-2.24	0.61	-3.37	0.00076	0.00843	LSM6B	SM-like 6B
	AT4G24805	458	-2.05	-2.19	0.53	-3.88	0.00011	0.00192	NA	NA
	AT5G16010	3171	-2.05	-2.19	0.53	-3.87	0.00011	0.00195	NA	NA
	AT3G22680	540	-2.05	-2.26	0.62	-3.31	0.00094	0.00981	RDM1	RNA-DIRECTED DNA METHYLATION 1
	AT1G29970	981	-2.05	-2.20	0.55	-3.75	0.00018	0.00285	RPL18AA	60S ribosomal protein L18A-1
	AT3G25030	205	-2.05	-2.27	0.63	-3.26	0.00112	0.01117	NA	NA
	AT4G35980	567	-2.05	-2.20	0.54	-3.79	0.00015	0.00250	NA	NA
	AT3G57040	1124	-2.05	-2.20	0.54	-3.81	0.00014	0.00236	ARR9	response regulator 9
	AT4G35905	497	-2.05	-2.24	0.59	-3.49	0.00048	0.00607	NA	NA
	AT2G22430	6961	-2.05	-2.20	0.54	-3.81	0.00014	0.00240	ATHB6	homeobox protein 6
	AT2G36320	7845	-2.05	-2.21	0.56	-3.68	0.00023	0.00349	NA	NA
	AT3G59980	1626	-2.05	-2.25	0.60	-3.40	0.00067	0.00774	NA	NA
	AT2G32690	13586	-2.05	-2.24	0.59	-3.46	0.00053	0.00653	ATGRP23	GLYCINE-RICH PROTEIN 23
	AT1G56220	14604	-2.05	-2.20	0.53	-3.87	0.00011	0.00195	NA	NA
	AT2G42310	4791	-2.05	-2.22	0.56	-3.64	0.00027	0.00395	NA	NA
	AT5G08050	7492	-2.05	-2.18	0.51	-4.05	0.00005	0.00111	NA	NA
YES	AT1G66480	190	-2.06	-2.23	0.57	-3.60	0.00032	0.00449	NA	NA
	AT2G45640	1491	-2.06	-2.22	0.57	-3.63	0.00028	0.00401	ATSAP18	SIN3 ASSOCIATED POLYPEPTIDE 18
	AT3G15420	84	-2.06	-2.30	0.66	-3.10	0.00191	0.01663	NA	NA
	AT4G30010	4941	-2.06	-2.21	0.55	-3.75	0.00018	0.00285	NA	NA
	AT2G25310	1698	-2.06	-2.22	0.56	-3.68	0.00023	0.00352	NA	NA
	AT1G29520	381	-2.06	-2.23	0.57	-3.60	0.00032	0.00448	NA	NA
	AT5G13220	167	-2.06	-2.23	0.57	-3.61	0.00031	0.00432	JAS1	JASMONATE-ASSOCIATED 1
	AT1G68760	424	-2.06	-2.22	0.55	-3.72	0.00020	0.00313	ATNUDT1	ARABIDOPSIS THALIANA NUDIX HYDROLASE HOMOLOG 1
	AT1G16640	97	-2.06	-2.28	0.63	-3.29	0.00099	0.01023	NA	NA
	AT4G35320	213	-2.06	-2.26	0.60	-3.44	0.00057	0.00689	NA	NA
	AT1G20460	139	-2.06	-2.46	0.79	-2.62	0.00868	0.04944	NA	NA
	AT1G31170	1810	-2.07	-2.24	0.57	-3.61	0.00031	0.00430	ATSRX	SULFIREDOXIN
	AT2G01080	567	-2.07	-2.23	0.56	-3.67	0.00025	0.00366	NA	NA
	AT2G01890	98	-2.07	-2.37	0.71	-2.91	0.00366	0.02689	ATPAP8	PURPLE ACID PHOSPHATASE 8
	AT3G62450	387	-2.07	-2.44	0.77	-2.69	0.00716	0.04376	NA	NA
	AT2G06925	1939	-2.07	-2.26	0.59	-3.51	0.00044	0.00565	ATSPLA2-AI	PHOSPHOLIPASE A2-ALPHA
	AT4G17870	2000	-2.07	-2.24	0.56	-3.69	0.00023	0.00344	PYR1	PYRABACTIN RESISTANCE 1
	AT2G30540	140	-2.07	-2.32	0.66	-3.13	0.00178	0.01586	NA	NA
	AT3G03840	370	-2.07	-2.32	0.66	-3.15	0.00166	0.01500	SAUR27	SMALL AUXIN UP RNA 27
	AT4G15630	1046	-2.07	-2.24	0.56	-3.67	0.00024	0.00358	NA	NA
	AT2G02510	4698	-2.08	-2.21	0.51	-4.10	0.00004	0.00095	NA	NA
	AT1G50040	223	-2.08	-2.27	0.60	-3.47	0.00052	0.00642	NA	NA
	AT3G18740	12137	-2.08	-2.23	0.54	-3.83	0.00013	0.00226	RLK902	receptor-like kinase 902

Supplemental Table 1-RNA seq data comparing Pro35S::MYB63 with wild type.

DAP.MYB63.target(1.5kb upstream TSS)	Gene	baseMean	log2FoldChange	lfcMLE	lfcSE	stat	pvalue	padj (FDR)	TAIR10 Symbol	TAIR10 Annotation (Short)
	AT1G16060	289	-2.08	-2.22	0.52	-3.99	0.00007	0.00136	ADAP	ARIA-interacting double AP2 domain protein
	AT3G53890	8789	-2.08	-2.22	0.52	-3.97	0.00007	0.00144	NA	NA
	AT1G13920	45	-2.08	-2.48	0.79	-2.63	0.00844	0.04855	NA	NA
	AT4G03240	536	-2.08	-2.23	0.53	-3.94	0.00008	0.00156	ATFH	0
	AT5G47130	36	-2.08	-2.47	0.78	-2.68	0.00740	0.04463	NA	NA
	AT3G13110	2096	-2.08	-2.20	0.48	-4.32	0.00002	0.00047	ATSERAT2;2	serine acetyltransferase 2;2
	AT2G42380	205	-2.08	-2.25	0.56	-3.75	0.00018	0.00285	ATBZIP34	0
	AT1G20310	41	-2.09	-2.38	0.70	-2.98	0.00291	0.02284	NA	NA
	AT5G60680	8476	-2.09	-2.24	0.54	-3.89	0.00010	0.00184	NA	NA
	AT5G62880	862	-2.09	-2.27	0.59	-3.56	0.00037	0.00500	ARAC10	RAC-like 10
	AT4G14540	1043	-2.09	-2.25	0.56	-3.75	0.00018	0.00285	NF-YB3	nuclear factor Y, subunit B3
	ATCG00430	6404	-2.09	-2.31	0.64	-3.28	0.00103	0.01055	PSBG	photosystem II reaction center protein G
	AT3G29170	742	-2.09	-2.28	0.59	-3.54	0.00040	0.00524	NA	NA
	AT3G13845	457	-2.09	-2.26	0.56	-3.72	0.00020	0.00311	NA	NA
	AT5G28442	75	-2.09	-2.48	0.78	-2.68	0.00739	0.04460	NA	NA
	AT3G08660	70	-2.09	-2.45	0.76	-2.75	0.00588	0.03792	NA	NA
	AT4G05590	737	-2.09	-2.26	0.57	-3.67	0.00024	0.00357	NA	NA
	AT3G55970	85	-2.09	-2.34	0.66	-3.19	0.00144	0.01342	ATJRG21	0
	AT1G22140	1428	-2.09	-2.26	0.57	-3.69	0.00022	0.00338	NA	NA
	AT5G03210	157	-2.09	-2.38	0.70	-2.99	0.00277	0.02193	AtDIP2	0
	AT4G21105	7997	-2.09	-2.24	0.53	-3.94	0.00008	0.00158	NA	NA
	AT2G27500	997	-2.09	-2.28	0.58	-3.60	0.00032	0.00441	NA	NA
	AT2G13660	304	-2.09	-2.36	0.68	-3.10	0.00194	0.01678	NA	NA
	AT2G46540	1691	-2.09	-2.24	0.54	-3.91	0.00009	0.00174	NA	NA
	AT3G18530	51	-2.09	-2.36	0.67	-3.12	0.00182	0.01611	NA	NA
	AT5G07220	2669	-2.10	-2.25	0.54	-3.85	0.00012	0.00209	ATBAG3	BCL-2-associated athanogene 3
	AT3G03160	1821	-2.10	-2.25	0.55	-3.82	0.00014	0.00234	NA	NA
	AT3G20490	137	-2.10	-2.30	0.61	-3.44	0.00059	0.00702	NA	NA
	AT3G07910	1704	-2.10	-2.26	0.56	-3.75	0.00018	0.00283	NA	NA
	AT5G46080	302	-2.10	-2.29	0.59	-3.55	0.00039	0.00515	NA	NA
	AT4G12000	210	-2.10	-2.27	0.57	-3.65	0.00026	0.00379	NA	NA
	AT1G75440	191	-2.10	-2.29	0.59	-3.57	0.00035	0.00482	UBC16	ubiquitin-conjugating enzyme 16
	AT3G47430	680	-2.10	-2.26	0.55	-3.80	0.00014	0.00243	PEX11B	peroxin 11B
	AT2G39470	2852	-2.10	-2.25	0.54	-3.88	0.00011	0.00192	PnsL1	Photosynthetic NDH subcomplex L 1
	AT5G64850	2045	-2.10	-2.26	0.54	-3.87	0.00011	0.00199	NA	NA
	AT3G20310	3084	-2.10	-2.26	0.54	-3.86	0.00011	0.00203	ATERF-7	0
	AT5G38660	6332	-2.10	-2.32	0.62	-3.38	0.00071	0.00806	APE1	ACCLIMATION OF PHOTOSYNTHESIS TO ENVIRONMENT
	AT2G19580	94	-2.10	-2.49	0.77	-2.72	0.00653	0.04084	TET2	tetraspanin2
	AT5G03905	721	-2.10	-2.34	0.64	-3.27	0.00106	0.01077	NA	NA
	AT2G31940	41	-2.11	-2.53	0.80	-2.64	0.00824	0.04796	NA	NA
	AT2G34340	44	-2.11	-2.50	0.78	-2.70	0.00683	0.04228	NA	NA
	AT4G34680	319	-2.11	-2.30	0.60	-3.51	0.00044	0.00567	GATA3	GATA transcription factor 3
	AT2G29320	812	-2.11	-2.25	0.52	-4.03	0.00006	0.00119	NA	NA
	AT2G47380	1361	-2.11	-2.26	0.53	-3.96	0.00007	0.00147	NA	NA
	AT1G78210	1757	-2.11	-2.26	0.53	-3.98	0.00007	0.00141	NA	NA
	AT4G12990	129	-2.11	-2.51	0.79	-2.69	0.00719	0.04389	NA	NA
	AT3G14430	1017	-2.11	-2.29	0.58	-3.66	0.00025	0.00370	NA	NA
	AT5G43750	3277	-2.11	-2.26	0.53	-4.02	0.00006	0.00124	NDH18	NAD(P)H dehydrogenase 18
	AT5G57060	559	-2.11	-2.28	0.56	-3.77	0.00016	0.00267	NA	NA
YES	AT5G26960	429	-2.11	-2.30	0.58	-3.62	0.00030	0.00424	NA	NA
	AT3G04400	37964	-2.11	-2.30	0.59	-3.56	0.00037	0.00497	emb2171	embryo defective 2171
YES	AT3G51920	4244	-2.11	-2.25	0.52	-4.10	0.00004	0.00095	ATCML9	0
	AT4G27960	6902	-2.11	-2.27	0.54	-3.89	0.00010	0.00188	UBC9	ubiquitin conjugating enzyme 9
	AT2G23680	391	-2.11	-2.32	0.62	-3.44	0.00059	0.00705	NA	NA
	AT5G65495	481	-2.11	-2.31	0.60	-3.52	0.00044	0.00562	NA	NA
	AT3G58120	470	-2.11	-2.26	0.53	-3.96	0.00008	0.00147	ATBZIP61	0
	AT5G03490	472	-2.12	-2.29	0.56	-3.76	0.00017	0.00279	NA	NA
	AT2G01590	2755	-2.12	-2.29	0.56	-3.77	0.00016	0.00266	CRR3	CHLORORESPIRATORY REDUCTION 3
	AT5G54300	166	-2.12	-2.33	0.62	-3.44	0.00059	0.00707	NA	NA
	AT1G16960	351	-2.12	-2.29	0.57	-3.71	0.00020	0.00316	NA	NA
	AT3G10520	4119	-2.12	-2.27	0.55	-3.88	0.00010	0.00190	AHB2	haemoglobin 2
	AT2G04550	953	-2.12	-2.28	0.55	-3.85	0.00012	0.00211	DSPTP1E	DUAL SPECIFICITY PROTEIN PHOSPHATASE 1E
	AT1G77710	3945	-2.12	-2.27	0.54	-3.95	0.00008	0.00150	AtCCP2	0
	AT3G47490	1420	-2.12	-2.33	0.61	-3.45	0.00057	0.00682	NA	NA
	AT1G56260	194	-2.12	-2.36	0.65	-3.25	0.00116	0.01143	MDO1	MERISTEM DISORGANIZATION 1
	AT1G02335	780	-2.12	-2.42	0.70	-3.01	0.00257	0.02085	GL22	germin-like protein subfamily 2 member 2 precursor

Supplemental Table 1-RNA seq data comparing Pro35S::MYB63 with wild type.

DAP.MYB63.target(1.5kb upstream TSS)	Gene	baseMean	log2FoldChange	lfcMLE	lfcSE	stat	pvalue	padj (FDR)	TAIR10 Symbol	TAIR10 Annotation (Short)
	AT3G61900	35	-2.12	-2.39	0.68	-3.12	0.00180	0.01601	SAUR33	SMALL AUXIN UPREGULATED RNA 33
	AT4G29430	279	-2.12	-2.37	0.65	-3.25	0.00115	0.01141	rps15ae	ribosomal protein S15A E
	AT3G60480	405	-2.12	-2.31	0.58	-3.66	0.00025	0.00369	NA	NA
YES	AT3G55420	147	-2.12	-2.47	0.74	-2.85	0.00433	0.03023	NA	NA
	AT2G23340	227	-2.12	-2.32	0.60	-3.57	0.00036	0.00487	DEAR3	DREB and EAR motif protein 3
	AT1G45145	3985	-2.12	-2.25	0.50	-4.26	0.00002	0.00055	ATH5	THIOREDOXIN H-TYPE 5
YES	AT2G44310	1485	-2.12	-2.27	0.53	-4.01	0.00006	0.00126	NA	NA
	AT4G31510	963	-2.13	-2.32	0.59	-3.62	0.00029	0.00413	NA	NA
	AT1G54630	4577	-2.13	-2.30	0.57	-3.72	0.00020	0.00311	ACP3	acyl carrier protein 3
	AT5G65380	886	-2.13	-2.30	0.57	-3.76	0.00017	0.00277	NA	NA
	AT1G66740	652	-2.13	-2.28	0.53	-4.03	0.00006	0.00118	ASF1A	ANTI- SILENCING FUNCTION 1A
YES	AT1G15120	4676	-2.13	-2.32	0.59	-3.63	0.00028	0.00404	NA	NA
	AT3G04720	13430	-2.13	-2.31	0.57	-3.74	0.00018	0.00290	AtPR4	0
	AT3G46630	2738	-2.13	-2.29	0.54	-3.96	0.00008	0.00148	NA	NA
	AT4G01680	62	-2.13	-2.41	0.68	-3.12	0.00181	0.01609	AtMYB55	myb domain protein 55
	AT3G25597	218	-2.13	-2.31	0.56	-3.78	0.00016	0.00262	NA	NA
	AT3G62400	2700	-2.13	-2.30	0.55	-3.88	0.00010	0.00192	NA	NA
	AT5G06190	90	-2.13	-2.52	0.77	-2.77	0.00555	0.03632	NA	NA
	AT4G39300	1056	-2.14	-2.27	0.51	-4.19	0.00003	0.00071	NA	NA
	AT1G19730	375	-2.14	-2.35	0.62	-3.47	0.00053	0.00647	ATH4	thioredoxin H-type 4
	AT3G50685	5106	-2.14	-2.32	0.58	-3.70	0.00021	0.00330	NA	NA
	AT4G04745	179	-2.14	-2.42	0.68	-3.14	0.00170	0.01531	NA	NA
	AT4G12880	1393	-2.14	-2.34	0.61	-3.52	0.00043	0.00558	AtENODL19	0
	AT1G54217	140	-2.14	-2.52	0.77	-2.79	0.00530	0.03520	NA	NA
	AT1G14450	4616	-2.14	-2.30	0.54	-3.95	0.00008	0.00152	NA	NA
	AT4G34970	136	-2.14	-2.38	0.65	-3.30	0.00097	0.01004	ADF9	actin depolymerizing factor 9
	AT3G01050	481	-2.14	-2.35	0.61	-3.53	0.00041	0.00535	MUB1	membrane-anchored ubiquitin-fold protein 1 precursor
	AT1G57765	1031	-2.14	-2.34	0.59	-3.60	0.00032	0.00446	NA	NA
	AT2G24040	388	-2.14	-2.45	0.71	-3.02	0.00251	0.02051	NA	NA
	AT1G52618	369	-2.14	-2.35	0.60	-3.57	0.00035	0.00481	NA	NA
	AT3G48590	1581	-2.14	-2.36	0.62	-3.45	0.00056	0.00678	ATHAP5A	0
	AT3G55250	3598	-2.14	-2.32	0.57	-3.79	0.00015	0.00253	PDE329	PIGMENT DEFECTIVE 329
	AT5G62575	985	-2.15	-2.30	0.54	-4.00	0.00006	0.00131	SDH7	succinate dehydrogenase 7
	AT1G10030	682	-2.15	-2.31	0.56	-3.85	0.00012	0.00211	ERG28	homolog of yeast ergosterol28
	AT2G28370	1595	-2.15	-2.31	0.55	-3.91	0.00009	0.00175	NA	NA
	AT1G35210	730	-2.15	-2.39	0.65	-3.30	0.00095	0.00990	NA	NA
	AT5G44582	181	-2.15	-2.39	0.65	-3.32	0.00089	0.00943	NA	NA
	AT3G57160	78	-2.15	-2.46	0.71	-3.00	0.00266	0.02130	NA	NA
	AT2G36060	4749	-2.15	-2.30	0.53	-4.05	0.00005	0.00110	MMZ3	MMS ZWEI homologue 3
	AT1G20470	108	-2.15	-2.38	0.63	-3.42	0.00062	0.00730	SAUR60	SMALL AUXIN UPREGULATED RNA 60
	AT5G11650	558	-2.15	-2.39	0.65	-3.31	0.00094	0.00981	NA	NA
	AT2G30570	182714	-2.15	-2.28	0.50	-4.29	0.00002	0.00051	PSBW	photosystem II reaction center W
	AT5G15320	3031	-2.15	-2.31	0.54	-3.97	0.00007	0.00145	NA	NA
	AT5G23760	489	-2.15	-2.31	0.55	-3.92	0.00009	0.00167	NA	NA
	AT4G16500	1802	-2.15	-2.30	0.52	-4.14	0.00004	0.00084	AtCYS4	0
	AT4G14305	32	-2.15	-2.60	0.81	-2.66	0.00779	0.04619	NA	NA
	AT2G27490	524	-2.15	-2.39	0.64	-3.35	0.00080	0.00877	ATCOAE	0
	AT5G08760	2199	-2.15	-2.39	0.64	-3.36	0.00078	0.00860	NA	NA
	AT5G02020	596	-2.15	-2.30	0.52	-4.13	0.00004	0.00085	SIS	Salt Induced Serine rich
	AT4G22290	1315	-2.15	-2.42	0.67	-3.21	0.00134	0.01270	NA	NA
	AT1G28230	208	-2.16	-2.56	0.78	-2.76	0.00572	0.03711	ATPUP1	0
	AT3G20450	126	-2.16	-2.35	0.59	-3.66	0.00025	0.00370	NA	NA
	AT5G16410	161	-2.16	-2.55	0.77	-2.81	0.00497	0.03348	NA	NA
	AT1G13390	1535	-2.16	-2.34	0.57	-3.80	0.00015	0.00247	NA	NA
	AT2G31570	3394	-2.16	-2.29	0.50	-4.31	0.00002	0.00049	ATGPX2	glutathione peroxidase 2
	AT5G05950	610	-2.16	-2.35	0.58	-3.71	0.00021	0.00319	MEE60	maternal effect embryo arrest 60
	AT3G43430	283	-2.16	-2.35	0.59	-3.69	0.00022	0.00338	NA	NA
	AT1G25560	10998	-2.16	-2.34	0.56	-3.84	0.00013	0.00220	AtTEM1	0
	AT5G62220	187	-2.17	-2.39	0.62	-3.49	0.00049	0.00611	ATGT18	glycosyltransferase 18
	AT3G08610	8450	-2.17	-2.31	0.51	-4.22	0.00002	0.00063	NA	NA
	AT4G16695	1727	-2.17	-2.37	0.60	-3.62	0.00029	0.00413	NA	NA
	AT4G37770	47	-2.17	-2.60	0.80	-2.71	0.00673	0.04179	ACS8	1-amino-cyclopropane-1-carboxylate synthase 8
	AT4G16510	497	-2.17	-2.39	0.62	-3.52	0.00043	0.00559	NA	NA
	AT2G35830	2882	-2.17	-2.33	0.55	-3.96	0.00008	0.00147	NA	NA
	AT5G37480	823	-2.17	-2.37	0.59	-3.68	0.00023	0.00352	NA	NA
	AT4G14010	1055	-2.17	-2.45	0.67	-3.22	0.00127	0.01221	RALFL32	ralf-like 32
	AT1G48770	128	-2.17	-2.42	0.64	-3.39	0.00069	0.00793	NA	NA
	AT5G63160	1209	-2.17	-2.32	0.52	-4.20	0.00003	0.00069	BT1	BTB and TAZ domain protein 1
	AT1G36980	5029	-2.17	-2.36	0.58	-3.74	0.00018	0.00289	NA	NA

Supplemental Table 1-RNA seq data comparing Pro35S::MYB63 with wild type.

DAP.MYB63.target(1.5kb upstream TSS)	Gene	baseMean	log2FoldChange	lfcMLE	lfcSE	stat	pvalue	padj (FDR)	TAIR10 Symbol	TAIR10 Annotation (Short)
	AT5G24980	1271	-2.18	-2.37	0.59	-3.69	0.00023	0.00344	NA	NA
	AT3G50350	219	-2.18	-2.36	0.58	-3.76	0.00017	0.00274	NA	NA
	AT1G68590	12282	-2.18	-2.33	0.54	-4.05	0.00005	0.00110	PSRP3/1	plastid-speciﬁc ribosomal protein 3/1
	AT2G01918	260	-2.18	-2.36	0.58	-3.78	0.00016	0.00259	PQL3	PsbQ-like 3
	AT1G78030	253	-2.18	-2.44	0.67	-3.27	0.00107	0.01080	NA	NA
	AT1G56500	2497	-2.18	-2.33	0.53	-4.11	0.00004	0.00092	SOQ1	suppressor of quenching 1
	AT1G21500	13367	-2.18	-2.34	0.53	-4.09	0.00004	0.00099	NA	NA
	AT1G29910	157469	-2.18	-2.32	0.51	-4.24	0.00002	0.00060	AB180	0
	AT2G24550	3181	-2.18	-2.34	0.54	-4.03	0.00006	0.00119	NA	NA
	AT5G13080	47	-2.18	-2.62	0.80	-2.74	0.00618	0.03926	ATWRKY75	ARABIDOPSIS THALIANA WRKY DNA-BINDING PROTEIN 75
	AT3G03000	533	-2.18	-2.36	0.56	-3.87	0.00011	0.00199	NA	NA
	AT2G05620	4032	-2.18	-2.31	0.49	-4.44	0.00001	0.00029	AtPGR5	0
	AT3G06145	576	-2.19	-2.36	0.57	-3.86	0.00011	0.00205	NA	NA
	AT1G34010	436	-2.19	-2.39	0.60	-3.66	0.00025	0.00370	NA	NA
	AT1G60660	611	-2.19	-2.35	0.55	-3.99	0.00007	0.00133	ATCB5LP	ARABIDOPSIS CYTOCHROME B5-LIKE PROTEIN
	AT1G53830	26	-2.19	-2.58	0.77	-2.84	0.00458	0.03152	ATPME2	pectin methylesterase 2
	AT3G05800	1083	-2.19	-2.34	0.53	-4.10	0.00004	0.00095	AIF1	AtBS1(activation-tagged BRI1 suppressor 1)-interacting factor 1
	AT1G78895	538	-2.19	-2.41	0.61	-3.57	0.00035	0.00481	NA	NA
	AT1G49245	360	-2.19	-2.37	0.57	-3.85	0.00012	0.00212	NA	NA
	AT4G34750	766	-2.19	-2.43	0.64	-3.44	0.00059	0.00707	SAUR49	SMALL AUXIN UPREGULATED RNA 49
	AT3G14630	30	-2.19	-2.68	0.83	-2.65	0.00803	0.04715	CYP72A9	cytochrome P450, family 72, subfamily A, polypeptide 9
	AT5G65480	2009	-2.19	-2.34	0.53	-4.15	0.00003	0.00080	CCI1	Clavata complex interactor 1
	AT1G35680	20541	-2.19	-2.41	0.61	-3.57	0.00036	0.00491	RPL21C	chloroplast ribosomal protein L21
	AT2G04790	387	-2.20	-2.38	0.57	-3.82	0.00013	0.00230	NA	NA
	AT3G11745	85	-2.20	-2.51	0.71	-3.11	0.00190	0.01656	NA	NA
	AT5G12050	321	-2.20	-2.41	0.60	-3.64	0.00027	0.00393	NA	NA
	AT3G62930	173	-2.20	-2.38	0.57	-3.83	0.00013	0.00227	NA	NA
	AT2G40420	1011	-2.20	-2.43	0.63	-3.50	0.00047	0.00598	NA	NA
	AT3G13275	124	-2.20	-2.43	0.62	-3.52	0.00043	0.00552	NA	NA
	AT2G39850	152	-2.20	-2.37	0.56	-3.95	0.00008	0.00151	NA	NA
	AT2G32190	145	-2.20	-2.40	0.59	-3.74	0.00018	0.00288	NA	NA
	AT2G24090	11721	-2.20	-2.35	0.52	-4.21	0.00003	0.00066	PRPL35	plastid ribosomal protein L35
	AT5G52050	105	-2.20	-2.45	0.65	-3.41	0.00066	0.00762	NA	NA
	AT3G15300	70	-2.20	-2.59	0.76	-2.88	0.00399	0.02846	NA	NA
	AT3G13480	445	-2.20	-2.40	0.59	-3.75	0.00017	0.00280	NA	NA
	AT1G11530	1379	-2.20	-2.40	0.59	-3.76	0.00017	0.00277	ATCXXS1	C-terminal cysteine residue is changed to a serine 1
	AT1G75630	3779	-2.20	-2.37	0.54	-4.04	0.00005	0.00114	AVA-P4	vacuolar H+-pumping ATPase 16 kDa proteolipid subunit 4
	AT1G14870	314	-2.20	-2.37	0.55	-4.01	0.00006	0.00126	AtPCR2	0
	AT5G62520	33	-2.20	-2.64	0.79	-2.78	0.00543	0.03581	SRO5	similar to RCD one 5
	AT3G15352	817	-2.21	-2.36	0.53	-4.17	0.00003	0.00074	ATCOX17	ARABIDOPSIS THALIANA CYTOCHROME C OXIDASE 17
	AT3G27940	109	-2.21	-2.42	0.60	-3.66	0.00025	0.00369	LBD26	LOB domain-containing protein 26
	AT5G11740	28946	-2.21	-2.37	0.54	-4.06	0.00005	0.00109	AGP15	arabinogalactan protein 15
	AT3G11650	379	-2.21	-2.50	0.69	-3.19	0.00141	0.01319	NHL2	NDR1/HIN1-like 2
	AT4G09890	1013	-2.21	-2.43	0.61	-3.61	0.00031	0.00436	NA	NA
	AT4G12800	252226	-2.21	-2.41	0.59	-3.73	0.00019	0.00296	PSAL	photosystem I subunit l
	AT5G07020	11509	-2.21	-2.39	0.56	-3.97	0.00007	0.00144	NA	NA
	AT1G53560	388	-2.21	-2.36	0.52	-4.22	0.00002	0.00065	NA	NA
	AT5G10930	331	-2.21	-2.39	0.57	-3.89	0.00010	0.00182	CIPK5	CBL-interacting protein kinase 5
	AT3G61190	356	-2.21	-2.39	0.56	-3.96	0.00008	0.00147	BAP1	BON association protein 1
	AT5G40370	7286	-2.21	-2.35	0.51	-4.34	0.00001	0.00042	GRXC2	glutaredoxin C2
	AT1G68000	1462	-2.21	-2.38	0.55	-4.02	0.00006	0.00123	ATPIS	PHOSPHATIDYLINOSITOL SYNTHASE
	AT5G19190	4498	-2.22	-2.36	0.52	-4.26	0.00002	0.00055	NA	NA
	AT5G03455	2824	-2.22	-2.41	0.58	-3.79	0.00015	0.00253	ACR2	ARSENATE REDUCTASE 2
	AT5G16660	4057	-2.22	-2.41	0.58	-3.79	0.00015	0.00254	NA	NA
	AT3G11090	831	-2.22	-2.36	0.52	-4.29	0.00002	0.00051	LBD21	LOB domain-containing protein 21
	AT5G09830	993	-2.22	-2.41	0.58	-3.82	0.00014	0.00234	BolA2	homolog of E.coli BolA 2
	AT2G30410	2222	-2.22	-2.41	0.58	-3.82	0.00013	0.00228	KIS	KIESEL
	AT2G27402	691	-2.22	-2.38	0.53	-4.17	0.00003	0.00076	NA	NA
	AT1G64980	1265	-2.22	-2.40	0.56	-3.95	0.00008	0.00151	CDI	cadmium 2+ induced
YES	AT3G52110	509	-2.22	-2.41	0.57	-3.89	0.00010	0.00186	NA	NA
	AT5G57290	9061	-2.22	-2.38	0.54	-4.11	0.00004	0.00091	NA	NA
	AT5G03370	456	-2.22	-2.39	0.55	-4.02	0.00006	0.00124	NA	NA
	AT5G22270	1664	-2.22	-2.40	0.57	-3.93	0.00009	0.00163	NA	NA

Supplemental Table 1-RNA seq data comparing Pro35S::MYB63 with wild type.

DAP.MYB63.target(1.5kb upstream TSS)	Gene	baseMean	log2FoldChange	lfcMLE	lfcSE	stat	pvalue	padj (FDR)	TAIR10 Symbol	TAIR10 Annotation (Short)
	AT5G04190	178	-2.22	-2.38	0.53	-4.22	0.00002	0.00065	PKS4	phytochrome kinase substrate 4
	AT3G05890	117	-2.22	-2.49	0.66	-3.36	0.00079	0.00873	RCI2B	RARE-COLD-INDUCIBLE 2B
	AT1G27290	3831	-2.22	-2.39	0.55	-4.05	0.00005	0.00111	NA	NA
	AT5G08391	29	-2.22	-2.72	0.83	-2.69	0.00706	0.04332	NA	NA
	AT2G20875	103	-2.22	-2.62	0.76	-2.91	0.00360	0.02661	EPF1	EPIDERMAL PATTERNING FACTOR 1
	AT3G23550	329	-2.23	-2.40	0.56	-3.96	0.00008	0.00148	NA	NA
	AT4G01575	47	-2.23	-2.65	0.78	-2.84	0.00453	0.03127	NA	NA
	AT1G73965	54	-2.23	-2.54	0.70	-3.19	0.00143	0.01339	CLE13	CLAVATA3/ESR-RELATED 13
	AT4G02075	1035	-2.23	-2.40	0.56	-3.99	0.00007	0.00136	PIT1	pitchoun 1
	AT5G56600	1890	-2.23	-2.42	0.59	-3.81	0.00014	0.00242	PFN3	PROFILIN 3
	AT1G08315	712	-2.23	-2.39	0.54	-4.14	0.00003	0.00082	NA	NA
	AT5G52960	2392	-2.23	-2.38	0.52	-4.30	0.00002	0.00050	NA	NA
	AT1G53670	3215	-2.23	-2.37	0.50	-4.46	0.00001	0.00027	ATMSRB1	0
	AT1G18690	197	-2.23	-2.60	0.75	-2.96	0.00304	0.02363	XXT4	xyloglucan xylosyltransferase 4
YES	AT3G47070	18813	-2.23	-2.40	0.55	-4.06	0.00005	0.00108	NA	NA
	AT1G21460	442	-2.23	-2.39	0.53	-4.20	0.00003	0.00067	AtSWEET1	0
	AT2G38790	444	-2.23	-2.38	0.52	-4.29	0.00002	0.00050	NA	NA
	AT3G06070	4327	-2.23	-2.39	0.53	-4.18	0.00003	0.00072	NA	NA
	AT1G57680	3011	-2.23	-2.48	0.65	-3.45	0.00055	0.00669	Cand1	candidate G-protein Coupled Receptor 1
	AT5G16480	258	-2.23	-2.48	0.64	-3.50	0.00047	0.00596	AtPFA-DSP5	0
	AT4G18205	2407	-2.23	-2.41	0.56	-3.97	0.00007	0.00141	NA	NA
	AT1G04070	566	-2.24	-2.46	0.62	-3.62	0.00029	0.00412	ATTOM22-I	0
	AT3G23255	217	-2.24	-2.51	0.67	-3.36	0.00078	0.00862	NA	NA
	AT2G29340	3170	-2.24	-2.43	0.58	-3.85	0.00012	0.00211	NA	NA
	AT3G19800	2125	-2.24	-2.40	0.53	-4.19	0.00003	0.00071	NA	NA
	AT1G70000	353	-2.24	-2.45	0.60	-3.74	0.00018	0.00289	NA	NA
	AT4G34620	54271	-2.24	-2.42	0.57	-3.93	0.00008	0.00161	SSR16	small subunit ribosomal protein 16
	AT2G22370	416	-2.24	-2.43	0.58	-3.84	0.00012	0.00216	MED18	mediator 18
	AT2G17300	145	-2.24	-2.43	0.57	-3.92	0.00009	0.00169	NA	NA
	AT4G29350	19192	-2.24	-2.39	0.51	-4.35	0.00001	0.00041	PFN2	profilin 2
	AT3G15770	950	-2.24	-2.42	0.56	-3.99	0.00007	0.00136	NA	NA
	AT5G60890	235	-2.24	-2.58	0.72	-3.11	0.00189	0.01652	ATMYB34	0
	AT4G15802	3527	-2.24	-2.39	0.52	-4.35	0.00001	0.00041	AtHSBP	Arabidopsis thaliana heat shock factor binding protein
	AT5G49300	42	-2.24	-2.65	0.78	-2.89	0.00382	0.02761	GATA16	GATA transcription factor 16
	AT2G41410	7818	-2.24	-2.43	0.57	-3.96	0.00008	0.00147	NA	NA
	AT1G65000	325	-2.24	-2.43	0.57	-3.92	0.00009	0.00165	NA	NA
	AT4G00810	8453	-2.25	-2.42	0.55	-4.09	0.00004	0.00098	NA	NA
	AT4G10100	1251	-2.25	-2.42	0.56	-4.00	0.00006	0.00130	CNX7	co-factor for nitrate, reductase and xanthine dehydrogenase 7
	AT1G10460	58	-2.25	-2.50	0.65	-3.48	0.00050	0.00629	GLP7	germin-like protein 7
	AT1G67785	2431	-2.25	-2.44	0.58	-3.85	0.00012	0.00209	NA	NA
	AT1G62820	1421	-2.25	-2.41	0.54	-4.18	0.00003	0.00073	NA	NA
	AT5G17280	1591	-2.25	-2.42	0.54	-4.13	0.00004	0.00087	NA	NA
	AT3G03020	550	-2.25	-2.45	0.59	-3.80	0.00014	0.00245	NA	NA
	AT4G29735	3244	-2.25	-2.43	0.57	-3.98	0.00007	0.00141	NA	NA
	AT1G24170	1520	-2.25	-2.42	0.55	-4.10	0.00004	0.00094	GATL8	GALACTURONOSYLTRANSFERASE-LIKE 8
	AT4G15670	789	-2.25	-2.42	0.55	-4.11	0.00004	0.00093	NA	NA
	AT1G62040	2176	-2.25	-2.44	0.58	-3.90	0.00010	0.00182	ATG8C	autophagy 8c
	AT5G60850	1531	-2.25	-2.47	0.61	-3.67	0.00024	0.00356	OBP4	OBF binding protein 4
	AT4G20030	900	-2.25	-2.48	0.62	-3.61	0.00030	0.00426	NA	NA
	AT3G10400	236	-2.25	-2.48	0.61	-3.68	0.00023	0.00352	U11/U12-3	U11/U12-31K
	AT1G75950	14177	-2.25	-2.42	0.55	-4.11	0.00004	0.00091	ASK1	ARABIDOPSIS SKP1 HOMOLOGUE 1
	AT1G07610	1913	-2.25	-2.49	0.62	-3.62	0.00029	0.00413	MT1C	metallothionein 1C
	AT5G10450	22001	-2.26	-2.45	0.58	-3.91	0.00009	0.00173	14-3-3lamb	14-3-3 PROTEIN G-BOX FACTOR14 LAMBDA
	AT4G01080	85	-2.26	-2.64	0.76	-2.99	0.00283	0.02230	TBL26	TRICHOME BIREFRINGENCE-LIKE 26
	AT1G75890	34	-2.26	-2.71	0.80	-2.82	0.00478	0.03255	NA	NA
	AT2G39445	210	-2.26	-2.46	0.59	-3.82	0.00014	0.00234	NA	NA
	AT3G29575	407	-2.26	-2.43	0.55	-4.10	0.00004	0.00095	AFP3	ABI five binding protein 3
	AT3G62190	843	-2.26	-2.47	0.59	-3.80	0.00015	0.00247	NA	NA
	AT4G29905	10147	-2.26	-2.43	0.54	-4.17	0.00003	0.00075	NA	NA
	ATCG01120	67	-2.26	-2.53	0.66	-3.41	0.00064	0.00750	RPS15	chloroplast ribosomal protein S15
	AT5G64770	4287	-2.26	-2.49	0.61	-3.68	0.00023	0.00349	CLEL 9	CLE-like 9
	AT1G17420	261	-2.26	-2.49	0.62	-3.65	0.00026	0.00382	ATLOX3	Arabidopsis thaliana lipoxygenase 3
	AT3G14060	116	-2.26	-2.49	0.62	-3.66	0.00025	0.00366	NA	NA
	AT3G17210	3629	-2.26	-2.43	0.55	-4.12	0.00004	0.00087	ATHS1	A. THALIANA HEAT STABLE PROTEIN 1
	AT4G16450	6496	-2.26	-2.45	0.57	-3.99	0.00007	0.00133	NA	NA

Supplemental Table 1-RNA seq data comparing Pro35S::MYB63 with wild type.

DAP.MYB63.target(1.5kb upstream TSS)	Gene	baseMean	log2FoldChange	lfcMLE	lfcSE	stat	pvalue	padj (FDR)	TAIR10 Symbol	TAIR10 Annotation (Short)
	AT4G01510	161	-2.27	-2.54	0.67	-3.40	0.00067	0.00774	ARV2	0
	AT4G39925	84	-2.27	-2.50	0.62	-3.65	0.00026	0.00382	NA	NA
	AT2G37530	98	-2.27	-2.52	0.64	-3.55	0.00038	0.00510	NA	NA
	AT1G11380	259	-2.27	-2.51	0.63	-3.57	0.00035	0.00481	NA	NA
	AT1G76690	32	-2.27	-2.70	0.79	-2.89	0.00390	0.02804	ATOPR2	ARABIDOPSIS 12-OXOPHYTODIENOATE REDUCTASE 2
	AT1G55475	180	-2.27	-2.70	0.79	-2.89	0.00384	0.02774	NA	NA
	AT2G22000	54	-2.27	-2.67	0.77	-2.96	0.00305	0.02368	PROPEP6	elicitor peptide 6 precursor
	AT3G13910	787	-2.27	-2.45	0.56	-4.04	0.00005	0.00116	NA	NA
	AT1G31300	1496	-2.27	-2.49	0.61	-3.73	0.00019	0.00304	NA	NA
	AT2G36900	1200	-2.27	-2.50	0.61	-3.70	0.00022	0.00334	ATMEMB11	0
	AT5G41590	25	-2.27	-2.86	0.86	-2.63	0.00845	0.04858	NA	NA
	AT3G61770	1300	-2.28	-2.48	0.59	-3.86	0.00011	0.00201	NA	NA
	AT2G46820	30082	-2.28	-2.41	0.49	-4.62	0.00000	0.00015	CURT1B	CURVATURE THYLAKOID 1B
	AT1G32210	3325	-2.28	-2.47	0.57	-3.99	0.00007	0.00136	ATDAD1	DEFENDER AGAINST APOPTOTIC DEATH 1
	AT3G15630	6806	-2.28	-2.49	0.61	-3.76	0.00017	0.00274	NA	NA
	AT4G04972	33	-2.28	-2.74	0.80	-2.83	0.00459	0.03159	NA	NA
	AT1G01180	1059	-2.28	-2.45	0.55	-4.17	0.00003	0.00074	NA	NA
	AT3G44735	557	-2.28	-2.50	0.61	-3.74	0.00018	0.00291	ATPSK3	PHYTOSULFOKINE 3 PRECURSOR
	AT3G05936	638	-2.28	-2.51	0.62	-3.66	0.00025	0.00373	NA	NA
	AT2G01540	867	-2.28	-2.53	0.64	-3.58	0.00035	0.00476	NA	NA
	AT2G15960	7864	-2.28	-2.46	0.56	-4.05	0.00005	0.00111	NA	NA
	AT3G10300	1596	-2.28	-2.59	0.69	-3.28	0.00104	0.01063	NA	NA
	AT1G17200	2913	-2.28	-2.45	0.55	-4.15	0.00003	0.00081	NA	NA
	AT2G23780	1652	-2.28	-2.49	0.59	-3.84	0.00012	0.00215	NA	NA
	AT1G01725	662	-2.28	-2.46	0.56	-4.08	0.00004	0.00100	NA	NA
	AT4G24990	1917	-2.28	-2.45	0.55	-4.17	0.00003	0.00074	ATGP4	0
	AT3G56910	20696	-2.28	-2.48	0.58	-3.90	0.00009	0.00177	PSRP5	plastid-specific 50S ribosomal protein 5
YES	AT5G44170	532	-2.28	-2.46	0.55	-4.15	0.00003	0.00080	NA	NA
	AT5G63905	516	-2.28	-2.50	0.61	-3.76	0.00017	0.00272	NA	NA
	AT1G32310	770	-2.28	-2.45	0.54	-4.20	0.00003	0.00068	SAMBA	SAMBA
	AT3G13677	184	-2.28	-2.51	0.61	-3.74	0.00018	0.00291	NA	NA
	AT4G16840	265	-2.29	-2.46	0.55	-4.13	0.00004	0.00087	NA	NA
	AT3G09860	4846	-2.29	-2.45	0.54	-4.23	0.00002	0.00063	NA	NA
	AT2G42530	955	-2.29	-2.48	0.57	-4.00	0.00006	0.00132	COR15B	cold regulated 15b
	AT2G28430	1984	-2.29	-2.48	0.57	-4.03	0.00006	0.00119	NA	NA
	AT1G54290	690	-2.29	-2.48	0.57	-4.04	0.00005	0.00115	NA	NA
	AT1G25275	7500	-2.29	-2.50	0.59	-3.88	0.00011	0.00193	NA	NA
	AT5G47890	4034	-2.29	-2.44	0.51	-4.53	0.00001	0.00021	NA	NA
	AT2G29490	34	-2.29	-2.67	0.74	-3.09	0.00203	0.01743	ATGSTU1	glutathione S-transferase TAU 1
YES	AT3G49940	4465	-2.29	-2.46	0.55	-4.19	0.00003	0.00070	LBD38	LOB domain-containing protein 38
	AT1G32070	1939	-2.29	-2.47	0.56	-4.12	0.00004	0.00087	ATNSI	nuclear shuttle interacting
	AT2G39030	122	-2.29	-2.73	0.79	-2.91	0.00361	0.02665	NATA1	N-acetyltransferase activity 1
YES	AT5G59470	492	-2.30	-2.57	0.67	-3.45	0.00056	0.00676	NA	NA
	AT4G18970	639	-2.30	-2.45	0.52	-4.45	0.00001	0.00029	NA	NA
	AT1G73260	2230	-2.30	-2.76	0.80	-2.86	0.00421	0.02967	ATKTI1	ARABIDOPSIS THALIANA KUNITZ TRYPSIN INHIBITOR 1
	AT5G62020	378	-2.30	-2.49	0.57	-4.00	0.00006	0.00131	AT-HSFB2A	ARABIDOPSIS THALIANA HEAT SHOCK TRANSCRIPTION FACTOR B2A
	ATCG00640	255	-2.30	-2.50	0.58	-3.97	0.00007	0.00144	RPL33	ribosomal protein L33
	AT2G40610	2061	-2.30	-2.44	0.50	-4.62	0.00000	0.00015	ATEXP8	0
	AT2G20260	49739	-2.30	-2.53	0.62	-3.72	0.00020	0.00308	PSAE-2	photosystem I subunit E-2
	AT5G45630	87	-2.30	-2.72	0.77	-2.99	0.00275	0.02188	NA	NA
	AT3G06760	696	-2.30	-2.50	0.58	-3.96	0.00007	0.00146	NA	NA
	AT4G15690	1417	-2.30	-2.48	0.55	-4.17	0.00003	0.00074	NA	NA
	AT1G19020	1337	-2.30	-2.56	0.64	-3.58	0.00034	0.00474	NA	NA
	AT4G14860	25	-2.30	-2.82	0.83	-2.78	0.00543	0.03580	AtOFP11	ovate family protein 11
	AT1G16825	199	-2.30	-2.52	0.60	-3.83	0.00013	0.00225	NA	NA
	AT5G44050	105	-2.30	-2.55	0.63	-3.64	0.00027	0.00395	NA	NA
	AT1G06080	318	-2.30	-2.56	0.64	-3.60	0.00031	0.00439	ADS1	delta 9 desaturase 1
	AT2G22640	1267	-2.30	-2.48	0.55	-4.17	0.00003	0.00075	ATBRK1	0
	AT1G36622	269	-2.30	-2.51	0.59	-3.88	0.00010	0.00191	NA	NA
	AT4G39710	3600	-2.30	-2.49	0.57	-4.07	0.00005	0.00105	FKBP16-2	FK506-binding protein 16-2
	AT1G32583	37	-2.31	-2.77	0.80	-2.88	0.00392	0.02815	NA	NA
	AT3G14770	570	-2.31	-2.51	0.58	-3.96	0.00008	0.00147	AtSWEET2	0
	AT2G31680	463	-2.31	-2.57	0.65	-3.55	0.00038	0.00512	AtRABA5d	RAB GTPase homolog A5D
	AT3G62550	12409	-2.31	-2.50	0.57	-4.07	0.00005	0.00104	NA	NA
	AT3G25600	1972	-2.31	-2.51	0.59	-3.94	0.00008	0.00158	NA	NA

Supplemental Table 1-RNA seq data comparing Pro35S::MYB63 with wild type.

DAP MYB63.target(1.5kb upstream TSS)	Gene	baseMean	log2FoldChange	lfcMLE	lfcSE	stat	pvalue	padj (FDR)	TAIR10 Symbol	TAIR10 Annotation (Short)
	AT4G23870	390	-2.31	-2.48	0.55	-4.23	0.00002	0.00062	NA	NA
	AT1G24575	569	-2.31	-2.51	0.58	-4.00	0.00006	0.00131	NA	NA
	AT3G15760	1264	-2.31	-2.49	0.56	-4.12	0.00004	0.00091	NA	NA
	AT3G15356	1544	-2.31	-2.50	0.57	-4.07	0.00005	0.00104	NA	NA
	AT3G08690	1875	-2.31	-2.52	0.59	-3.92	0.00009	0.00167	ATUBC11	0
	AT1G44350	276	-2.31	-2.52	0.60	-3.88	0.00011	0.00192	ILL6	IAA-leucine resistant (ILR)-like gene 6
	AT1G80920	20666	-2.31	-2.49	0.55	-4.20	0.00003	0.00068	AtJ8	0
	AT5G24780	110	-2.31	-2.72	0.77	-3.01	0.00265	0.02130	ATVSP1	0
	AT5G08410	4682	-2.31	-2.50	0.57	-4.09	0.00004	0.00099	FTRA2	ferredoxin/thioredoxin reductase subunit A (variable subunit) 2
	AT3G57320	660	-2.31	-2.49	0.56	-4.16	0.00003	0.00076	NA	NA
	AT1G61930	22	-2.31	-2.88	0.85	-2.72	0.00658	0.04106	NA	NA
	AT5G67490	2302	-2.31	-2.50	0.56	-4.10	0.00004	0.00095	NA	NA
	AT5G57345	9072	-2.32	-2.49	0.55	-4.21	0.00003	0.00066	NA	NA
	AT1G13370	20	-2.32	-2.91	0.86	-2.69	0.00725	0.04404	NA	NA
	AT1G52905	336	-2.32	-2.52	0.58	-3.99	0.00007	0.00134	NA	NA
YES	AT1G66820	1572	-2.32	-2.48	0.53	-4.36	0.00001	0.00040	NA	NA
	AT1G73630	430	-2.32	-2.52	0.58	-3.98	0.00007	0.00139	NA	NA
	AT4G02380	20466	-2.32	-2.58	0.65	-3.59	0.00032	0.00450	AtLEA5	Arabidopsis thaliana late embryogenensis abundant like 5
	AT2G27030	5320	-2.32	-2.49	0.55	-4.24	0.00002	0.00061	ACAM-2	0
	AT1G53625	62	-2.32	-2.75	0.78	-2.98	0.00291	0.02286	NA	NA
	AT5G53045	494	-2.32	-2.52	0.57	-4.06	0.00005	0.00109	NA	NA
	AT3G46890	38	-2.32	-2.83	0.81	-2.87	0.00412	0.02919	NA	NA
	AT1G75810	538	-2.33	-2.55	0.60	-3.86	0.00011	0.00204	NA	NA
	AT2G25964	349	-2.33	-2.57	0.63	-3.68	0.00023	0.00346	NA	NA
	AT5G49100	646	-2.33	-2.49	0.54	-4.33	0.00001	0.00045	NA	NA
	AT1G26470	1564	-2.33	-2.52	0.56	-4.12	0.00004	0.00090	SNS1	SnRK2-substrate 1
	AT5G61660	1557	-2.33	-2.50	0.54	-4.32	0.00002	0.00047	NA	NA
	AT3G63160	13997	-2.33	-2.51	0.56	-4.19	0.00003	0.00070	OEP6	outer envelope protein 6
	AT3G47675	186	-2.33	-2.67	0.72	-3.24	0.00120	0.01179	NA	NA
	AT5G42110	555	-2.33	-2.53	0.58	-4.05	0.00005	0.00113	NA	NA
	AT2G21210	1788	-2.33	-2.54	0.59	-3.92	0.00009	0.00165	SAUR6	SMALL AUXIN UPREGULATED RNA 6
	AT3G49870	2858	-2.33	-2.53	0.58	-4.00	0.00006	0.00131	ARLA1C	ADP-ribosylation factor-like A1C
	AT3G61560	141	-2.33	-2.56	0.61	-3.82	0.00013	0.00232	NA	NA
	AT5G42825	2627	-2.33	-2.57	0.62	-3.78	0.00016	0.00264	NA	NA
	AT5G04330	141	-2.33	-2.75	0.77	-3.02	0.00249	0.02044	CYP84A4	CYTOCHROME P450 84A4
	AT4G31560	2311	-2.33	-2.49	0.52	-4.46	0.00001	0.00028	HCF153	high chlorophyll fluorescence 153
	AT1G47278	577	-2.33	-2.54	0.59	-3.94	0.00008	0.00154	NA	NA
	AT5G24920	99	-2.33	-2.73	0.76	-3.08	0.00205	0.01754	AtGDU5	glutamine dumper 5
	AT5G11970	905	-2.33	-2.54	0.59	-3.97	0.00007	0.00141	NA	NA
	AT3G51380	19	-2.33	-2.99	0.88	-2.64	0.00832	0.04818	IQD20	IQ-domain 20
	AT4G02620	3693	-2.33	-2.51	0.54	-4.32	0.00002	0.00047	NA	NA
	AT1G63245	36	-2.33	-2.86	0.83	-2.82	0.00483	0.03279	CLE14	CLAVATA3/ESR-RELATED 14
	AT3G22210	2395	-2.33	-2.53	0.57	-4.07	0.00005	0.00104	NA	NA
	AT1G68840	9612	-2.34	-2.53	0.58	-4.05	0.00005	0.00113	AtRAV2	0
	AT1G23980	114	-2.34	-2.70	0.73	-3.20	0.00138	0.01297	NA	NA
	AT2G29420	255	-2.34	-2.49	0.52	-4.53	0.00001	0.00021	ATGSTU7	glutathione S-transferase tau 7
	AT1G62660	142	-2.34	-2.58	0.62	-3.75	0.00018	0.00287	NA	NA
	AT3G29000	664	-2.34	-2.72	0.74	-3.15	0.00165	0.01494	NA	NA
	AT1G14340	809	-2.34	-2.54	0.57	-4.07	0.00005	0.00104	NA	NA
	AT3G57540	29	-2.34	-2.81	0.80	-2.92	0.00348	0.02600	NA	NA
	AT1G64230	13893	-2.34	-2.51	0.54	-4.31	0.00002	0.00049	UBC28	ubiquitin-conjugating enzyme 28
	AT1G07410	65	-2.34	-2.78	0.78	-3.00	0.00266	0.02131	ATRAB-A2B	ARABIDOPSIS RAB GTPASE HOMOLOG A2B
	AT3G15680	1037	-2.34	-2.54	0.57	-4.09	0.00004	0.00099	NA	NA
YES	AT4G39970	6554	-2.34	-2.61	0.65	-3.58	0.00034	0.00469	NA	NA
	AT3G05400	15	-2.34	-3.01	0.89	-2.64	0.00831	0.04818	NA	NA
	AT5G42850	2912	-2.34	-2.55	0.59	-4.00	0.00006	0.00129	NA	NA
	AT1G33475	95	-2.34	-2.56	0.60	-3.91	0.00009	0.00172	NA	NA
	AT5G41160	76	-2.35	-2.79	0.79	-2.99	0.00282	0.02225	ATPUP12	ARABIDOPSIS THALIANA PURINE PERMEASE 12
	AT1G12250	1848	-2.35	-2.55	0.58	-4.07	0.00005	0.00105	NA	NA
	AT3G54990	22	-2.35	-2.90	0.84	-2.78	0.00543	0.03580	SMZ	SCHLAFMUTZE
	AT1G06515	653	-2.35	-2.52	0.54	-4.36	0.00001	0.00039	NA	NA
	AT5G20110	130	-2.35	-2.54	0.56	-4.17	0.00003	0.00075	NA	NA
	AT1G28250	1098	-2.35	-2.51	0.53	-4.42	0.00001	0.00031	NA	NA
	AT3G22930	96	-2.35	-2.86	0.82	-2.86	0.00418	0.02950	AtCML11	0
	AT5G53880	1696	-2.35	-2.51	0.53	-4.42	0.00001	0.00031	NA	NA
	AT1G18773	114	-2.35	-2.77	0.77	-3.06	0.00221	0.01857	NA	NA
	AT5G50800	54	-2.35	-2.78	0.78	-3.02	0.00254	0.02068	AtSWEET13	0

Supplemental Table 1-RNA seq data comparing Pro35S::MYB63 with wild type.

DAP.MYB63.target(1.5kb upstream TSS)	Gene	baseMean	log2FoldChange	lfcMLE	lfcSE	stat	pvalue	padj (FDR)	TAIR10 Symbol	TAIR10 Annotation (Short)
	AT1G75590	91	-2.35	-2.71	0.73	-3.23	0.00124	0.01202	SAUR52	SMALL AUXIN UPREGULATED RNA 52
	AT2G44080	865	-2.35	-2.53	0.56	-4.21	0.00002	0.00065	ARL	ARGOS-like
	AT3G53232	260	-2.35	-2.63	0.66	-3.54	0.00040	0.00528	DVL20	DEVIL 20
	AT4G39930	61	-2.35	-2.85	0.81	-2.90	0.00379	0.02751	NA	NA
	AT1G47655	98	-2.35	-2.76	0.76	-3.10	0.00191	0.01663	NA	NA
	AT5G42200	327	-2.35	-2.76	0.76	-3.10	0.00197	0.01700	NA	NA
	AT1G45201	2822	-2.35	-2.54	0.56	-4.23	0.00002	0.00062	ATTLL1	ARABIDOPSIS THALIANA TRIACYLGLYCEROL LIPASE-LIKE 1
	AT3G47836	3064	-2.35	-2.54	0.56	-4.19	0.00003	0.00069	NA	NA
	AT1G72020	6612	-2.35	-2.54	0.56	-4.22	0.00002	0.00065	NA	NA
	AT4G15990	61	-2.35	-2.79	0.78	-3.03	0.00248	0.02039	NA	NA
	AT5G19855	2133	-2.35	-2.56	0.59	-4.01	0.00006	0.00128	AtRbcX2	0
	AT1G61667	152	-2.36	-2.66	0.69	-3.43	0.00060	0.00714	NA	NA
	AT2G33380	364	-2.36	-2.73	0.74	-3.20	0.00137	0.01294	AtCLO3	Arabidopsis thaliana caleosin 3
	AT1G22030	139	-2.36	-2.68	0.70	-3.37	0.00076	0.00843	NA	NA
	AT2G23320	4195	-2.36	-2.59	0.62	-3.83	0.00013	0.00222	AtWRKY15	0
	AT1G22630	3588	-2.36	-2.55	0.56	-4.19	0.00003	0.00071	NA	NA
	AT2G23755	88	-2.36	-2.81	0.79	-3.00	0.00270	0.02153	NA	NA
	AT5G25760	1518	-2.36	-2.65	0.67	-3.50	0.00046	0.00580	PEX4	peroxin4
	AT1G67350	3257	-2.36	-2.53	0.54	-4.40	0.00001	0.00034	NA	NA
YES	AT4G32240	775	-2.36	-2.59	0.61	-3.87	0.00011	0.00199	NA	NA
	AT5G09976	22	-2.36	-2.97	0.86	-2.73	0.00632	0.03994	NA	NA
	AT5G23160	25	-2.36	-2.90	0.83	-2.83	0.00462	0.03171	NA	NA
	AT1G75550	31	-2.36	-2.89	0.83	-2.85	0.00432	0.03022	NA	NA
	AT1G49500	17705	-2.36	-2.55	0.56	-4.23	0.00002	0.00062	NA	NA
	AT3G07470	4115	-2.36	-2.58	0.60	-3.96	0.00007	0.00146	NA	NA
	AT2G28105	81	-2.37	-2.82	0.79	-3.00	0.00272	0.02163	NA	NA
	AT2G26695	196	-2.37	-2.55	0.55	-4.29	0.00002	0.00051	NA	NA
	AT3G47833	1756	-2.37	-2.54	0.53	-4.43	0.00001	0.00031	SDH7	succinate dehydrogenase 7
	AT5G43260	1972	-2.37	-2.55	0.55	-4.29	0.00002	0.00051	NA	NA
	AT5G42650	7846	-2.37	-2.51	0.50	-4.73	0.00000	0.00010	AOS	allene oxide synthase
	AT1G04290	879	-2.37	-2.56	0.57	-4.17	0.00003	0.00075	NA	NA
	AT4G31330	167	-2.37	-2.62	0.63	-3.79	0.00015	0.00256	NA	NA
	AT1G66400	297	-2.37	-2.59	0.60	-3.97	0.00007	0.00142	CML23	calmodulin like 23
YES	AT5G23230	41	-2.37	-2.82	0.78	-3.03	0.00241	0.01994	NIC2	nicotinamidase 2
	AT5G08040	1839	-2.37	-2.54	0.53	-4.46	0.00001	0.00027	TOM5	mitochondrial import receptor subunit TOM5 homolog
	AT3G52730	5470	-2.38	-2.57	0.56	-4.20	0.00003	0.00067	NA	NA
	AT2G41475	1064	-2.38	-2.53	0.51	-4.70	0.00000	0.00011	NA	NA
	AT5G09240	188	-2.38	-2.81	0.77	-3.08	0.00206	0.01758	NA	NA
	AT1G73530	1758	-2.38	-2.60	0.59	-4.01	0.00006	0.00128	NA	NA
	AT5G54530	219	-2.38	-2.60	0.60	-3.96	0.00007	0.00147	NA	NA
	AT4G39070	167	-2.38	-2.66	0.66	-3.63	0.00029	0.00410	BBX20	B-box domain protein 20
	AT5G57815	1650	-2.38	-2.57	0.56	-4.28	0.00002	0.00053	NA	NA
	AT1G54530	26	-2.39	-3.00	0.86	-2.77	0.00553	0.03627	NA	NA
	AT4G28290	853	-2.39	-2.60	0.59	-4.03	0.00006	0.00120	NA	NA
	AT5G59130	71	-2.39	-2.81	0.77	-3.11	0.00190	0.01655	NA	NA
	AT4G23530	880	-2.39	-2.73	0.70	-3.40	0.00067	0.00769	NA	NA
	AT5G17600	438	-2.39	-2.65	0.63	-3.79	0.00015	0.00254	NA	NA
	AT1G01170	6447	-2.39	-2.63	0.62	-3.88	0.00010	0.00191	NA	NA
	AT2G46490	3133	-2.39	-2.58	0.56	-4.31	0.00002	0.00048	NA	NA
	AT4G14020	714	-2.40	-2.58	0.56	-4.30	0.00002	0.00050	NA	NA
	AT5G39340	754	-2.40	-2.61	0.58	-4.11	0.00004	0.00093	AHP3	histidine-containing phosphotransmitter 3
	AT5G16110	5743	-2.40	-2.58	0.55	-4.34	0.00001	0.00043	NA	NA
	AT1G77855	40	-2.40	-2.89	0.80	-2.98	0.00289	0.02273	NA	NA
	AT1G76520	1787	-2.40	-2.60	0.57	-4.22	0.00002	0.00064	NA	NA
	AT2G29950	42	-2.40	-2.83	0.77	-3.13	0.00178	0.01586	ELF4-L1	ELF4-like 1
	AT4G08555	187	-2.40	-2.73	0.69	-3.47	0.00051	0.00636	NA	NA
	AT1G49975	3185	-2.40	-2.60	0.57	-4.23	0.00002	0.00063	NA	NA
	AT1G79660	1038	-2.41	-2.64	0.61	-3.96	0.00008	0.00147	NA	NA
	ATCG01020	61392	-2.41	-2.64	0.61	-3.94	0.00008	0.00154	RPL32	ribosomal protein L32
	AT2G19350	614	-2.41	-2.64	0.61	-3.95	0.00008	0.00149	NA	NA
	AT1G71100	647	-2.41	-2.66	0.63	-3.85	0.00012	0.00211	RSW10	RADIAL SWELLING 10
	AT2G20562	99	-2.41	-2.69	0.65	-3.69	0.00023	0.00345	NA	NA
	AT2G32030	174	-2.41	-2.64	0.61	-3.98	0.00007	0.00138	NA	NA
	AT2G13360	40062	-2.41	-2.64	0.60	-4.01	0.00006	0.00128	AGT	alanine:glyoxylate aminotransferase
	AT2G25605	906	-2.41	-2.59	0.55	-4.42	0.00001	0.00032	NA	NA
	AT1G07985	18	-2.41	-3.03	0.86	-2.81	0.00496	0.03344	NA	NA
	AT5G20180	1219	-2.42	-2.59	0.54	-4.48	0.00001	0.00025	NA	NA

Supplemental Table 1-RNA seq data comparing Pro35S::MYB63 with wild type.

DAP.MYB63.target(1.5kb upstream TSS)	Gene	baseMean	log2FoldChange	lfcMLE	lfcSE	stat	pvalue	padj (FDR)	TAIR10 Symbol	TAIR10 Annotation (Short)
	AT2G31230	600	-2.42	-2.62	0.58	-4.20	0.00003	0.00068	ATERF15	ethylene-responsive element binding factor 15
	AT4G15920	2725	-2.42	-2.61	0.56	-4.35	0.00001	0.00041	AtSWEET17	0
	AT3G27630	60	-2.42	-2.76	0.70	-3.44	0.00057	0.00688	SMR7	SIAMESE-RELATED 7
	AT3G05000	1233	-2.42	-2.69	0.64	-3.76	0.00017	0.00274	NA	NA
	AT1G12845	1108	-2.42	-2.59	0.53	-4.58	0.00000	0.00017	NA	NA
	AT4G25890	4176	-2.42	-2.62	0.57	-4.27	0.00002	0.00055	NA	NA
	AT2G33175	35	-2.42	-2.92	0.80	-3.01	0.00257	0.02085	NA	NA
	AT5G49015	442	-2.42	-2.63	0.58	-4.18	0.00003	0.00072	NA	NA
	AT2G47270	120	-2.42	-2.65	0.60	-4.05	0.00005	0.00113	UPB1	UPBEAT1
YES	AT3G23805	836	-2.42	-2.60	0.55	-4.44	0.00001	0.00029	RALFL24	ralf-like 24
	AT1G22890	927	-2.42	-2.62	0.56	-4.30	0.00002	0.00049	NA	NA
	AT2G29180	1402	-2.42	-2.61	0.55	-4.40	0.00001	0.00034	NA	NA
	AT1G52550	169	-2.42	-2.84	0.76	-3.20	0.00136	0.01288	NA	NA
	AT1G76610	72	-2.42	-2.64	0.59	-4.11	0.00004	0.00093	NA	NA
	AT2G19810	2830	-2.43	-2.67	0.62	-3.91	0.00009	0.00172	AtOZF1	0
	AT5G19230	356	-2.43	-2.71	0.66	-3.70	0.00021	0.00330	NA	NA
	AT1G08165	65	-2.43	-2.94	0.81	-3.01	0.00263	0.02116	NA	NA
	AT5G20120	610	-2.43	-2.70	0.64	-3.77	0.00016	0.00269	NA	NA
	AT5G42070	2399	-2.43	-2.64	0.57	-4.24	0.00002	0.00060	NA	NA
	AT5G02540	253	-2.43	-2.87	0.77	-3.17	0.00152	0.01406	NA	NA
	AT2G19310	2199	-2.43	-2.62	0.55	-4.43	0.00001	0.00030	NA	NA
	AT2G34920	32	-2.43	-2.86	0.77	-3.18	0.00149	0.01385	EDA18	embryo sac development arrest 18
	AT5G05430	53	-2.43	-2.93	0.80	-3.03	0.00243	0.02009	NA	NA
	AT1G74340	1012	-2.43	-2.60	0.53	-4.63	0.00000	0.00014	DPMS2	dolichol phosphate mannose synthase 2
	AT1G35430	317	-2.44	-2.66	0.60	-4.08	0.00004	0.00100	NA	NA
	AT5G67140	407	-2.44	-2.64	0.57	-4.30	0.00002	0.00050	NA	NA
	AT3G56160	1201	-2.44	-2.80	0.72	-3.40	0.00068	0.00785	NA	NA
	AT5G60220	15	-2.44	-3.21	0.91	-2.67	0.00754	0.04520	TET4	tetraspanin4
	AT1G62422	1015	-2.44	-2.66	0.60	-4.10	0.00004	0.00096	NA	NA
	AT1G12600	15	-2.44	-3.28	0.93	-2.63	0.00861	0.04918	NA	NA
	AT3G45050	3236	-2.44	-2.65	0.58	-4.21	0.00003	0.00067	NA	NA
YES	AT5G44210	42	-2.44	-2.86	0.76	-3.22	0.00130	0.01240	ATERF-9	ERF DOMAIN PROTEIN- 9
	AT5G08150	47	-2.44	-2.86	0.76	-3.21	0.00132	0.01261	SOB5	SUPPRESSOR OF PHYTOCHROME B 5
	AT3G52520	20	-2.44	-3.04	0.85	-2.88	0.00392	0.02815	NA	NA
	AT1G50732	2321	-2.44	-2.64	0.56	-4.38	0.00001	0.00037	NA	NA
	AT3G44220	178	-2.45	-2.91	0.78	-3.13	0.00174	0.01556	NA	NA
	AT4G32930	1550	-2.45	-2.67	0.59	-4.13	0.00004	0.00085	NA	NA
	AT1G29300	51	-2.45	-2.85	0.75	-3.26	0.00111	0.01111	UNE1	unfertilized embryo sac 1
	AT2G01150	116	-2.45	-2.66	0.58	-4.19	0.00003	0.00071	RHA2B	RING-H2 finger protein 2B
	AT5G39800	594	-2.45	-2.63	0.54	-4.55	0.00001	0.00020	NA	NA
	AT1G17380	59	-2.45	-2.87	0.76	-3.23	0.00124	0.01202	JAZ5	jasmonate-zim-domain protein 5
	AT3G46880	77	-2.45	-2.79	0.69	-3.53	0.00042	0.00547	NA	NA
	AT2G21195	796	-2.45	-2.69	0.61	-4.04	0.00005	0.00117	NA	NA
	AT5G25240	193	-2.45	-2.68	0.60	-4.11	0.00004	0.00091	NA	NA
	AT2G45830	43	-2.45	-2.92	0.79	-3.11	0.00186	0.01638	DTA2	downstream target of AGL15 2
	AT1G27695	1072	-2.45	-2.68	0.60	-4.07	0.00005	0.00106	NA	NA
	AT1G32760	95	-2.45	-2.72	0.64	-3.85	0.00012	0.00213	NA	NA
	AT1G06475	47	-2.45	-2.93	0.79	-3.10	0.00193	0.01677	NA	NA
	AT1G29390	2213	-2.45	-2.67	0.58	-4.22	0.00002	0.00064	COR314-TM	cold regulated 314 thylakoid membrane 2
	AT2G23985	275	-2.45	-2.72	0.64	-3.85	0.00012	0.00211	NA	NA
	AT3G47650	11693	-2.45	-2.65	0.55	-4.43	0.00001	0.00030	NA	NA
	AT1G57990	4506	-2.46	-2.73	0.65	-3.80	0.00015	0.00248	ATPUP18	purine permease 18
	AT5G35920	12	-2.46	-3.32	0.93	-2.63	0.00842	0.04850	CYP79A4P	cytochrome P450, family 79, subfamily A, polypeptide 4 pseudogene
	AT5G23460	417	-2.46	-2.77	0.67	-3.65	0.00026	0.00377	NA	NA
	AT5G54145	936	-2.46	-2.65	0.55	-4.50	0.00001	0.00024	NA	NA
	AT2G27580	1604	-2.46	-2.66	0.56	-4.37	0.00001	0.00038	NA	NA
	AT3G51750	160	-2.46	-2.71	0.62	-3.99	0.00007	0.00133	NA	NA
	AT3G53990	7320	-2.46	-2.63	0.52	-4.73	0.00000	0.00010	NA	NA
	AT5G54940	8688	-2.46	-2.67	0.57	-4.31	0.00002	0.00048	NA	NA
	AT3G16070	23	-2.46	-3.03	0.83	-2.97	0.00301	0.02344	NA	NA
	AT1G76955	482	-2.47	-2.70	0.61	-4.07	0.00005	0.00106	NA	NA
	AT5G03345	2783	-2.47	-2.68	0.58	-4.25	0.00002	0.00058	NA	NA
	AT1G07440	520	-2.47	-2.64	0.54	-4.61	0.00000	0.00015	NA	NA
	AT2G18938	12	-2.47	-3.24	0.90	-2.74	0.00616	0.03911	NA	NA
	AT2G39500	720	-2.47	-2.66	0.56	-4.44	0.00001	0.00029	NA	NA
	AT4G21740	804	-2.47	-2.67	0.57	-4.35	0.00001	0.00041	NA	NA

Supplemental Table 1-RNA seq data comparing Pro35S::MYB63 with wild type.

DAP MYB63.target(1.5kb upstream TSS)	Gene	baseMean	log2FoldChange	lfcMLE	lfcSE	stat	pvalue	padj (FDR)	TAIR10 Symbol	TAIR10 Annotation (Short)
	AT4G32260	50214	-2.47	-2.69	0.58	-4.25	0.00002	0.00057	PDE334	PIGMENT DEFECTIVE 334
	AT5G09980	42	-2.47	-2.86	0.73	-3.41	0.00066	0.00764	PROPEP4	elicitor peptide 4 precursor
	AT5G19030	582	-2.47	-2.79	0.68	-3.66	0.00025	0.00368	NA	NA
	AT1G53160	280	-2.48	-2.69	0.58	-4.26	0.00002	0.00056	FTM6	FLORAL TRANSITION AT THE MERISTEM6
	AT1G19910	9404	-2.48	-2.69	0.58	-4.28	0.00002	0.00053	ATVHA-C2	VACUOLAR-TYPE H+ ATPASE C2
	AT3G56360	16978	-2.48	-2.70	0.59	-4.21	0.00003	0.00067	NA	NA
	AT5G16170	173	-2.48	-2.71	0.60	-4.15	0.00003	0.00080	NA	NA
	AT2G28605	2069	-2.48	-2.69	0.57	-4.34	0.00001	0.00043	NA	NA
	AT4G03500	760	-2.48	-2.70	0.59	-4.23	0.00002	0.00062	NA	NA
	AT4G12970	476	-2.48	-2.70	0.59	-4.23	0.00002	0.00062	EPFL9	0
	AT1G02470	109	-2.48	-2.91	0.76	-3.27	0.00109	0.01097	NA	NA
	AT5G58580	127	-2.48	-2.72	0.60	-4.13	0.00004	0.00085	ATL63	TOXICOS EN LEVADURA 63
	AT3G05685	45	-2.48	-3.10	0.85	-2.92	0.00346	0.02590	NA	NA
	AT1G60870	3272	-2.49	-2.68	0.55	-4.48	0.00001	0.00026	MEE9	maternal effect embryo arrest 9
	AT3G54900	6547	-2.49	-2.67	0.54	-4.61	0.00000	0.00015	ATGRXCP	GLUTAREDOXIN
	AT5G40970	345	-2.49	-2.71	0.59	-4.22	0.00002	0.00064	NA	NA
	AT2G27930	24	-2.49	-3.16	0.87	-2.85	0.00439	0.03049	NA	NA
	AT3G09162	45	-2.49	-2.89	0.74	-3.36	0.00078	0.00860	NA	NA
	AT4G21620	2212	-2.49	-2.67	0.55	-4.56	0.00001	0.00019	NA	NA
YES	AT3G49330	38	-2.49	-2.97	0.79	-3.16	0.00159	0.01451	NA	NA
	AT5G11070	7913	-2.49	-2.66	0.52	-4.76	0.00000	0.00009	NA	NA
	AT2G19760	5559	-2.49	-2.65	0.51	-4.89	0.00000	0.00005	PFN1	PROFILIN 1
	AT4G16000	466	-2.49	-2.75	0.63	-3.97	0.00007	0.00144	NA	NA
	AT3G01960	58	-2.49	-2.91	0.75	-3.31	0.00094	0.00983	NA	NA
	AT1G69690	1176	-2.49	-2.74	0.61	-4.06	0.00005	0.00107	AtTCP15	0
	AT1G02816	782	-2.49	-2.79	0.66	-3.77	0.00017	0.00271	NA	NA
	AT4G14450	14	-2.49	-3.25	0.90	-2.76	0.00583	0.03768	NA	NA
	AT5G54600	12118	-2.49	-2.69	0.55	-4.51	0.00001	0.00023	RPL24	plastid ribosomal protein L24
	AT4G00360	3560	-2.49	-2.69	0.56	-4.47	0.00001	0.00026	ATT1	ABERRANT INDUCTION OF TYPE THREE 1
	AT1G03820	388	-2.50	-2.69	0.56	-4.47	0.00001	0.00027	NA	NA
	AT5G56550	4590	-2.50	-2.69	0.56	-4.47	0.00001	0.00026	ATOXS3	OXIDATIVE STRESS 3
	AT1G17455	498	-2.50	-2.72	0.58	-4.27	0.00002	0.00053	ELF4-L4	ELF4-like 4
	AT4G28330	57	-2.50	-2.95	0.77	-3.26	0.00112	0.01114	NA	NA
	AT1G14400	13956	-2.50	-2.75	0.62	-4.06	0.00005	0.00109	ATUBC1	0
	AT4G37140	15	-2.50	-3.39	0.93	-2.68	0.00733	0.04434	ATMES20	ARABIDOPSIS THALIANA METHYL ESTERASE 20
	AT1G10470	9748	-2.50	-2.72	0.58	-4.29	0.00002	0.00051	ARR4	response regulator 4
	AT2G20500	109	-2.50	-2.73	0.59	-4.22	0.00002	0.00064	NA	NA
	AT1G70670	607	-2.50	-2.79	0.65	-3.83	0.00013	0.00225	AtCLO4	Arabidopsis thaliana caleosin 4
	AT5G02160	42093	-2.50	-2.71	0.56	-4.44	0.00001	0.00029	NA	NA
	AT5G67600	3967	-2.50	-2.69	0.55	-4.56	0.00001	0.00019	WIH1	WINDHOSE 1
	AT2G30930	2938	-2.50	-2.73	0.59	-4.23	0.00002	0.00062	NA	NA
	AT4G14560	188	-2.51	-2.74	0.59	-4.23	0.00002	0.00061	AXR5	AUXIN RESISTANT 5
	AT5G44574	12	-2.51	-3.49	0.95	-2.64	0.00837	0.04830	NA	NA
	AT1G51355	131	-2.51	-2.84	0.68	-3.67	0.00025	0.00366	NA	NA
	AT3G29030	1018	-2.51	-2.81	0.66	-3.78	0.00016	0.00262	ATEXP5	ARABIDOPSIS THALIANA EXPANSIN 5
	AT5G56100	1246	-2.51	-2.70	0.55	-4.54	0.00001	0.00020	NA	NA
YES	AT4G40045	1139	-2.51	-2.70	0.54	-4.63	0.00000	0.00014	NA	NA
	AT1G05810	294	-2.51	-2.72	0.57	-4.45	0.00001	0.00029	ARA	0
	AT4G32030	2323	-2.51	-2.82	0.67	-3.78	0.00016	0.00263	NA	NA
	AT4G36515	193	-2.52	-2.78	0.63	-4.01	0.00006	0.00128	NA	NA
	AT5G49525	130	-2.52	-2.88	0.71	-3.56	0.00037	0.00501	NA	NA
	AT3G15450	36296	-2.52	-2.74	0.59	-4.25	0.00002	0.00057	NA	NA
	AT5G21940	21699	-2.52	-2.72	0.56	-4.46	0.00001	0.00027	NA	NA
	AT5G53750	132	-2.52	-2.70	0.54	-4.63	0.00000	0.00014	NA	NA
	AT1G35140	2868	-2.52	-2.83	0.67	-3.74	0.00018	0.00292	EXL1	EXORDIUM like 1
	AT5G18310	977	-2.52	-2.77	0.62	-4.06	0.00005	0.00106	NA	NA
	AT1G16170	1308	-2.52	-2.75	0.59	-4.27	0.00002	0.00054	NA	NA
	AT4G21745	12	-2.52	-3.52	0.96	-2.63	0.00854	0.04893	NA	NA
	AT5G49320	52	-2.52	-3.02	0.79	-3.17	0.00150	0.01390	NA	NA
	AT1G80745	14	-2.52	-3.33	0.91	-2.77	0.00565	0.03679	NA	NA
	AT1G65520	398	-2.52	-2.81	0.65	-3.87	0.00011	0.00199	ATECI1	ARABIDOPSIS THALIANA DELTA(3), DELTA(2)-ENOYL COA ISOMERASE 1
	AT1G22250	248	-2.52	-2.75	0.59	-4.25	0.00002	0.00058	NA	NA
	AT3G60966	59	-2.52	-2.94	0.75	-3.39	0.00071	0.00806	NA	NA
	AT3G25400	131	-2.52	-2.97	0.76	-3.31	0.00092	0.00968	NA	NA
	AT4G37800	6616	-2.53	-2.71	0.55	-4.62	0.00000	0.00015	XTH7	xyloglucan endotransglucosylase/hydrolase 7
	AT1G18730	3424	-2.53	-2.74	0.57	-4.40	0.00001	0.00034	NDF6	NDH dependent flow 6

Supplemental Table 1-RNA seq data comparing Pro35S::MYB63 with wild type.

DAP.MYB63.target(1.5kb upstream TSS)	Gene	baseMean	log2FoldChange	lfcMLE	lfcSE	stat	pvalue	padj (FDR)	TAIR10 Symbol	TAIR10 Annotation (Short)
	AT5G18920	66	-2.53	-3.01	0.78	-3.23	0.00124	0.01202	NA	NA
	AT4G28703	336	-2.53	-2.88	0.70	-3.60	0.00032	0.00448	NA	NA
	AT5G02370	960	-2.53	-2.70	0.53	-4.82	0.00000	0.00007	NA	NA
	AT3G61113	88	-2.53	-3.07	0.82	-3.10	0.00192	0.01669	URM12	ubiquitin-related modifier 12
	AT3G04860	344	-2.53	-2.79	0.62	-4.07	0.00005	0.00105	NA	NA
	AT1G80555	13	-2.53	-3.34	0.91	-2.77	0.00552	0.03624	NA	NA
	AT4G27435	334	-2.53	-2.80	0.63	-4.00	0.00006	0.00130	NA	NA
	AT1G12940	11	-2.53	-3.44	0.94	-2.70	0.00690	0.04261	ATNRT2.5	nitrate transporter2.5
	AT2G46640	80	-2.53	-3.01	0.78	-3.23	0.00123	0.01197	TAC1	Tiller Angle Control 1
	AT3G04640	2621	-2.53	-2.77	0.60	-4.24	0.00002	0.00061	NA	NA
	AT3G45600	2652	-2.53	-2.72	0.53	-4.74	0.00000	0.00009	TET3	tetraspanin3
	AT1G68500	88	-2.54	-3.01	0.78	-3.25	0.00114	0.01133	NA	NA
	AT5G66580	578	-2.54	-2.73	0.55	-4.62	0.00000	0.00015	NA	NA
	AT5G05365	87	-2.54	-2.85	0.67	-3.81	0.00014	0.00235	NA	NA
	ATCG01010	647	-2.54	-2.80	0.62	-4.09	0.00004	0.00099	NDHF	0
	AT1G02400	54	-2.54	-2.88	0.69	-3.68	0.00023	0.00346	ATGA2OX4	Arabidopsis thaliana gibberellin 2-oxidase 4
	AT2G36895	1317	-2.54	-2.71	0.52	-4.85	0.00000	0.00006	NA	NA
	AT4G13520	7229	-2.54	-2.73	0.54	-4.68	0.00000	0.00012	SMAP1	small acidic protein 1
	AT3G16570	7288	-2.54	-2.75	0.57	-4.45	0.00001	0.00029	ATRALF23	ARABIDOPSIS RAPID ALKALINIZATION FACTOR 23
	AT5G59410	444	-2.54	-2.82	0.63	-4.02	0.00006	0.00122	NA	NA
	AT1G63295	72	-2.54	-2.96	0.74	-3.42	0.00064	0.00742	NA	NA
	AT5G16450	911	-2.55	-2.79	0.61	-4.21	0.00003	0.00067	NA	NA
	AT4G14640	13	-2.55	-3.62	0.96	-2.64	0.00834	0.04823	AtCML8	calmodulin-like 8
	AT5G37670	44	-2.55	-2.99	0.76	-3.34	0.00084	0.00910	NA	NA
	AT1G72750	2586	-2.55	-2.76	0.57	-4.45	0.00001	0.00029	ATTIM23-2	translocase inner membrane subunit 23-2
YES	AT2G35760	295	-2.55	-2.78	0.59	-4.32	0.00002	0.00047	NA	NA
	AT1G32520	623	-2.55	-2.79	0.60	-4.27	0.00002	0.00055	NA	NA
	AT5G18404	41	-2.55	-3.16	0.84	-3.03	0.00246	0.02024	NA	NA
	AT1G65486	663	-2.56	-2.73	0.53	-4.85	0.00000	0.00006	NA	NA
	AT1G29490	43	-2.56	-3.12	0.81	-3.16	0.00160	0.01463	SAUR68	SMALL AUXIN UPREGULATED 68
	AT4G04925	622	-2.56	-2.83	0.63	-4.07	0.00005	0.00105	NA	NA
	AT3G48100	775	-2.56	-2.78	0.58	-4.40	0.00001	0.00034	ARR5	response regulator 5
	AT1G59970	394	-2.56	-2.88	0.68	-3.79	0.00015	0.00256	NA	NA
	AT5G15970	10304	-2.56	-2.77	0.57	-4.51	0.00001	0.00022	AtCor6.6	0
	AT5G19790	9	-2.56	-3.67	0.97	-2.64	0.00819	0.04778	RAP2.11	related to AP2 11
	AT4G21510	471	-2.56	-2.75	0.54	-4.74	0.00000	0.00009	AtFBS2	0
	AT1G29395	1272	-2.57	-2.82	0.61	-4.19	0.00003	0.00070	COR413-TM	COLD REGULATED 314 THYLAKOID MEMBRANE 1
	AT4G28730	751	-2.57	-2.79	0.58	-4.43	0.00001	0.00031	GrxC5	glutaredoxin C5
	AT1G72030	1802	-2.57	-2.81	0.60	-4.25	0.00002	0.00059	NA	NA
YES	AT1G24600	34	-2.57	-3.04	0.78	-3.31	0.00092	0.00968	NA	NA
	AT2G05540	1023	-2.57	-2.77	0.55	-4.65	0.00000	0.00013	NA	NA
	AT2G38140	23090	-2.57	-2.78	0.56	-4.56	0.00001	0.00019	PSRP4	plastid-specific ribosomal protein 4
	AT1G19510	245	-2.57	-2.85	0.64	-4.04	0.00005	0.00117	ATRL5	RAD-like 5
	AT3G09390	21916	-2.57	-2.80	0.59	-4.35	0.00001	0.00041	ATMT-1	ARABIDOPSIS THALIANA METALLOTHIONEIN-1
	AT3G03870	6191	-2.57	-2.81	0.60	-4.30	0.00002	0.00050	NA	NA
	AT5G05370	4635	-2.57	-2.79	0.58	-4.42	0.00001	0.00031	NA	NA
	AT2G43290	2368	-2.57	-2.78	0.56	-4.62	0.00000	0.00015	MSS3	multicopy suppressors of snf4 deficiency in yeast 3
	AT5G17220	229	-2.57	-2.82	0.60	-4.27	0.00002	0.00055	ATGSTF12	ARABIDOPSIS THALIANA GLUTATHIONE S-TRANSFERASE PHI 12
	AT5G64040	93507	-2.57	-2.74	0.51	-5.09	0.00000	0.00002	PSAN	0
	AT3G48185	163	-2.58	-3.11	0.80	-3.20	0.00137	0.01296	NA	NA
	AT5G51720	1173	-2.58	-2.71	0.47	-5.48	0.00000	0.00000	At-NEET	0
	AT3G45730	1186	-2.58	-2.79	0.57	-4.49	0.00001	0.00024	NA	NA
	AT1G14250	586	-2.58	-2.78	0.55	-4.66	0.00000	0.00013	NA	NA
	AT2G26500	42738	-2.58	-2.77	0.54	-4.79	0.00000	0.00008	NA	NA
	AT3G16690	187	-2.58	-3.07	0.78	-3.30	0.00097	0.01006	AtSWEET16	0
	AT5G63225	42	-2.58	-3.04	0.77	-3.35	0.00081	0.00885	NA	NA
	AT4G05070	11778	-2.58	-2.82	0.60	-4.31	0.00002	0.00047	NA	NA
	AT3G60540	314	-2.58	-2.85	0.63	-4.10	0.00004	0.00095	NA	NA
	AT3G08860	23	-2.58	-3.26	0.87	-2.97	0.00301	0.02348	PYD4	PYRIMIDINE 4
	AT3G29639	39	-2.58	-3.14	0.81	-3.17	0.00153	0.01408	NA	NA
	AT3G24535	26	-2.58	-3.17	0.83	-3.12	0.00184	0.01622	NA	NA
	AT4G19200	16672	-2.59	-2.79	0.56	-4.58	0.00000	0.00017	NA	NA
	AT1G68238	830	-2.59	-2.84	0.62	-4.20	0.00003	0.00069	NA	NA

Supplemental Table 1-RNA seq data comparing Pro35S::MYB63 with wild type.

DAP MYB63.target(1.5kb upstream TSS)	Gene	baseMean	log2FoldChange	lfcMLE	lfcSE	stat	pvalue	padj (FDR)	TAIR10 Symbol	TAIR10 Annotation (Short)
	AT5G01445	37	-2.59	-3.08	0.78	-3.31	0.00092	0.00968	NA	NA
	AT4G34720	7889	-2.59	-2.78	0.54	-4.81	0.00000	0.00007	ATVHA-C1	0
	AT4G27657	191	-2.59	-2.88	0.64	-4.04	0.00005	0.00115	NA	NA
	AT5G36220	140	-2.59	-2.98	0.72	-3.60	0.00032	0.00448	CYP81D1	cytochrome P450, family 81, subfamily D, polypeptide 1
	AT1G22160	324	-2.59	-2.79	0.55	-4.75	0.00000	0.00009	NA	NA
	AT2G38465	140	-2.59	-2.81	0.57	-4.55	0.00001	0.00020	NA	NA
	AT4G25050	25263	-2.60	-2.79	0.55	-4.71	0.00000	0.00011	ACP4	acyl carrier protein 4
	AT1G74890	145	-2.60	-2.93	0.68	-3.80	0.00015	0.00248	ARR15	response regulator 15
	AT2G15830	184	-2.60	-2.79	0.54	-4.77	0.00000	0.00008	NA	NA
	AT1G52855	436	-2.60	-2.81	0.57	-4.53	0.00001	0.00021	NA	NA
	AT4G27460	42	-2.60	-3.09	0.79	-3.30	0.00096	0.00997	CBSX5	CBS domain containing protein 5
	AT1G66890	909	-2.60	-2.76	0.51	-5.13	0.00000	0.00002	NA	NA
	AT5G25450	31	-2.60	-3.23	0.85	-3.07	0.00214	0.01815	NA	NA
	AT1G49475	43	-2.60	-3.13	0.80	-3.24	0.00120	0.01176	NA	NA
YES	AT1G67050	1224	-2.60	-2.77	0.51	-5.09	0.00000	0.00002	NA	NA
	AT4G21200	8	-2.60	-3.89	0.99	-2.63	0.00866	0.04939	ATGA2OX8	ARABIDOPSIS THALIANA GIBBERELLIN 2-OXIDASE 8
	AT4G08330	750	-2.60	-3.09	0.78	-3.33	0.00086	0.00924	NA	NA
	AT5G46871	288	-2.60	-2.87	0.62	-4.21	0.00003	0.00067	NA	NA
	AT4G01897	1193	-2.60	-2.81	0.56	-4.65	0.00000	0.00013	NA	NA
	AT5G56540	156	-2.61	-2.84	0.58	-4.46	0.00001	0.00027	AGP14	arabinogalactan protein 14
	AT4G17030	103	-2.61	-3.04	0.75	-3.49	0.00048	0.00604	AT-EXPR	0
	AT2G24790	17128	-2.61	-2.84	0.59	-4.44	0.00001	0.00029	ATCOL3	0
	AT1G71340	1408	-2.61	-2.86	0.61	-4.28	0.00002	0.00053	AtGDPD4	0
	AT2G03440	15371	-2.61	-2.80	0.54	-4.80	0.00000	0.00008	ATNRP1	0
	AT1G76180	13950	-2.61	-2.82	0.56	-4.66	0.00000	0.00013	ERD14	EARLY RESPONSE TO DEHYDRATION 14
	AT1G21050	533	-2.61	-2.82	0.56	-4.68	0.00000	0.00012	NA	NA
	AT4G36791	21	-2.62	-3.43	0.91	-2.89	0.00386	0.02780	NA	NA
	AT5G21950	55	-2.62	-2.96	0.69	-3.81	0.00014	0.00235	NA	NA
	AT1G05065	124	-2.62	-2.85	0.59	-4.46	0.00001	0.00028	CLE20	CLAVATA3/ESR-RELATED 20
	AT2G44360	1302	-2.62	-2.84	0.58	-4.54	0.00001	0.00020	NA	NA
	AT1G64770	2962	-2.62	-2.85	0.58	-4.50	0.00001	0.00024	NDF2	NDH-dependent cyclic electron flow 1
	AT2G37950	416	-2.62	-2.86	0.59	-4.46	0.00001	0.00028	NA	NA
	AT5G02090	229	-2.62	-2.96	0.68	-3.87	0.00011	0.00200	NA	NA
	AT2G35290	119	-2.62	-2.95	0.67	-3.90	0.00010	0.00181	SAUR79	SMALL AUXIN UPREGULATED RNA 79
	AT2G22820	23	-2.63	-3.35	0.88	-3.00	0.00270	0.02156	NA	NA
	AT2G40880	9621	-2.63	-2.80	0.51	-5.11	0.00000	0.00002	ATCYSA	cystatin A
YES	AT4G15140	247	-2.63	-2.95	0.67	-3.92	0.00009	0.00166	NA	NA
	AT5G59890	6922	-2.63	-2.84	0.55	-4.76	0.00000	0.00009	ADF4	actin depolymerizing factor 4
	AT5G52740	27	-2.63	-3.23	0.83	-3.18	0.00145	0.01350	NA	NA
	AT3G17120	992	-2.64	-2.96	0.67	-3.95	0.00008	0.00154	NA	NA
	AT3G56800	14499	-2.64	-2.82	0.53	-5.00	0.00000	0.00003	ACAM-3	CALMODULIN 3
	AT3G48970	91	-2.64	-3.07	0.75	-3.54	0.00040	0.00530	NA	NA
	AT4G12080	127	-2.64	-3.06	0.74	-3.56	0.00037	0.00497	AHL1	AT-hook motif nuclear-localized protein 1
	AT3G21270	802	-2.64	-2.86	0.57	-4.67	0.00000	0.00012	ADOF2	DOF zinc finger protein 2
	AT1G10560	17	-2.64	-3.33	0.86	-3.06	0.00222	0.01862	ATPUB18	ARABIDOPSIS THALIANA PLANT U-BOX 18
YES	AT2G41420	3490	-2.64	-2.86	0.57	-4.66	0.00000	0.00013	WIH2	WINDHOSE 2
	AT5G66110	10	-2.64	-3.89	0.98	-2.69	0.00721	0.04392	HIPP27	heavy metal associated isoprenylated plant protein 27
	AT3G47620	3039	-2.64	-2.96	0.66	-4.00	0.00006	0.00130	AtTCP14	TEOSINTE BRANCHED, cycloidea and PCF (TCP) 14
	AT4G27280	2706	-2.64	-2.84	0.54	-4.89	0.00000	0.00005	NA	NA
	AT1G01250	300	-2.65	-2.86	0.57	-4.65	0.00000	0.00013	NA	NA
	AT1G52220	19161	-2.65	-2.89	0.59	-4.47	0.00001	0.00026	CURT1C	CURVATURE THYLAKOID 1C
	AT5G15960	76	-2.65	-2.95	0.65	-4.09	0.00004	0.00099	KIN1	0
	AT5G02120	7916	-2.65	-2.87	0.57	-4.61	0.00000	0.00015	OHP	one helix protein
	AT3G20680	1799	-2.65	-2.86	0.56	-4.70	0.00000	0.00011	NA	NA
	AT3G46460	2007	-2.65	-2.90	0.61	-4.37	0.00001	0.00038	UBC13	ubiquitin-conjugating enzyme 13
	AT5G18020	796	-2.65	-2.98	0.67	-3.98	0.00007	0.00138	SAUR20	SMALL AUXIN UP RNA 20
	AT5G64816	812	-2.66	-2.94	0.63	-4.20	0.00003	0.00068	NA	NA
	AT1G64380	364	-2.66	-2.89	0.58	-4.56	0.00001	0.00019	NA	NA
	AT4G00305	22	-2.66	-3.30	0.84	-3.15	0.00164	0.01490	NA	NA
	AT4G16590	11	-2.66	-3.81	0.97	-2.73	0.00642	0.04032	ATCSLA01	cellulose synthase-like A01
	AT2G18350	513	-2.66	-2.87	0.56	-4.73	0.00000	0.00010	AtHB24	homeobox protein 24
	AT3G24065	27	-2.66	-3.25	0.82	-3.23	0.00123	0.01195	NA	NA
	AT5G65207	2810	-2.66	-2.93	0.62	-4.29	0.00002	0.00052	NA	NA

Supplemental Table 1-RNA seq data comparing Pro35S::MYB63 with wild type.

DAP.MYB63.target(1.5kb upstream TSS)	Gene	baseMean	log2FoldChange	lfcMLE	lfcSE	stat	pvalue	padj (FDR)	TAIR10 Symbol	TAIR10 Annotation (Short)
	AT1G70700	6362	-2.66	-2.98	0.66	-4.04	0.00005	0.00114	JAZ9	JASMONATE-ZIM-DOMAIN PROTEIN 9
	AT3G25905	32	-2.66	-3.26	0.83	-3.23	0.00125	0.01207	CLE27	CLAVATA3/ESR-RELATED 27
	AT1G75250	524	-2.67	-2.88	0.56	-4.76	0.00000	0.00009	ATRL6	RAD-like 6
	AT5G38770	35	-2.67	-3.35	0.86	-3.11	0.00187	0.01641	AtGDU7	glutamine dumper 7
	AT5G27760	3402	-2.67	-2.92	0.59	-4.49	0.00001	0.00024	NA	NA
	AT5G67390	644	-2.67	-2.89	0.57	-4.69	0.00000	0.00011	NA	NA
	AT3G25780	240	-2.67	-3.15	0.77	-3.47	0.00052	0.00641	AOC3	allene oxide cyclase 3
	AT2G01818	92	-2.67	-3.09	0.73	-3.65	0.00027	0.00385	NA	NA
	AT4G34800	27	-2.67	-3.36	0.86	-3.12	0.00183	0.01618	SAUR4	SMALL AUXIN UPREGULATED RNA 4
	AT3G27030	1062	-2.67	-2.95	0.63	-4.28	0.00002	0.00053	NA	NA
	AT1G07135	2896	-2.68	-2.96	0.63	-4.27	0.00002	0.00055	NA	NA
	AT4G16740	19	-2.68	-3.43	0.89	-3.02	0.00249	0.02042	ATTPS03	terpene synthase 03
	AT5G56795	14	-2.68	-3.49	0.90	-2.99	0.00281	0.02222	MT1B	metallothionein 1B
YES	AT1G11785	10	-2.68	-3.80	0.96	-2.79	0.00526	0.03500	NA	NA
	AT5G64230	80	-2.68	-3.03	0.69	-3.91	0.00009	0.00173	NA	NA
	AT4G10150	10	-2.68	-3.83	0.97	-2.78	0.00551	0.03614	NA	NA
	AT1G78000	409	-2.69	-2.91	0.57	-4.70	0.00000	0.00011	SEL1	SELENATE RESISTANT 1
	AT1G55152	824	-2.69	-2.94	0.60	-4.47	0.00001	0.00027	NA	NA
	AT2G45450	605	-2.69	-2.96	0.62	-4.31	0.00002	0.00048	ZPR1	LITTLE ZIPPER 1
	AT5G66080	65	-2.69	-2.98	0.64	-4.20	0.00003	0.00068	APD9	Arabidopsis Pp2c clade D 9
	AT5G22210	1543	-2.69	-2.89	0.55	-4.89	0.00000	0.00005	NA	NA
	AT4G08930	1223	-2.69	-3.02	0.67	-4.02	0.00006	0.00122	APRL6	APR-like 6
	AT4G37980	755	-2.69	-2.85	0.49	-5.46	0.00000	0.00000	ATCAD7	0
	AT1G14150	2526	-2.69	-2.91	0.56	-4.76	0.00000	0.00009	PnsL2	Photosynthetic NDH subcomplex L 2
YES	AT5G56230	63	-2.69	-3.18	0.77	-3.49	0.00048	0.00604	PRA1.G2	prenylated RAB acceptor 1.G2
	AT2G26975	2702	-2.69	-2.87	0.52	-5.17	0.00000	0.00002	COPT6	copper transporter 6
	AT1G58235	1929	-2.69	-3.03	0.67	-4.00	0.00006	0.00132	NA	NA
	AT4G17470	90	-2.70	-3.20	0.78	-3.46	0.00055	0.00664	NA	NA
	AT4G08685	2894	-2.70	-2.89	0.53	-5.10	0.00000	0.00002	SAH7	0
	AT1G14540	47	-2.70	-3.32	0.83	-3.24	0.00118	0.01160	PER4	peroxidase 4
	AT2G25625	62	-2.70	-3.19	0.77	-3.48	0.00049	0.00619	NA	NA
	AT1G51402	57	-2.70	-3.12	0.73	-3.69	0.00022	0.00343	NA	NA
	AT1G61795	445	-2.70	-2.96	0.61	-4.42	0.00001	0.00031	NA	NA
	AT1G67340	1030	-2.70	-2.94	0.58	-4.66	0.00000	0.00013	NA	NA
	AT5G44005	1014	-2.70	-2.92	0.56	-4.83	0.00000	0.00007	NA	NA
	AT5G18850	604	-2.70	-2.89	0.52	-5.18	0.00000	0.00001	NA	NA
	AT2G27310	986	-2.70	-2.93	0.57	-4.74	0.00000	0.00009	NA	NA
	AT3G13720	1976	-2.71	-2.90	0.54	-5.05	0.00000	0.00003	PRA1.F3	PRENYLATED RAB ACCEPTOR 1.F3
	AT4G12382	37	-2.71	-3.28	0.81	-3.33	0.00085	0.00922	NA	NA
	AT5G18600	3930	-2.71	-2.89	0.53	-5.16	0.00000	0.00002	NA	NA
	AT5G50950	1370	-2.71	-2.90	0.53	-5.09	0.00000	0.00002	FUM2	FUMARASE 2
	AT1G16730	236	-2.71	-3.05	0.68	-4.01	0.00006	0.00128	UP6	unknown protein 6
	AT3G25770	9316	-2.71	-2.96	0.59	-4.56	0.00001	0.00019	AOC2	allene oxide cyclase 2
	AT5G65220	37482	-2.71	-2.96	0.59	-4.62	0.00000	0.00015	NA	NA
	AT2G32210	231	-2.71	-3.02	0.64	-4.21	0.00003	0.00065	NA	NA
YES	AT3G22540	17	-2.71	-3.46	0.87	-3.11	0.00190	0.01656	NA	NA
	AT2G40670	240	-2.71	-2.97	0.60	-4.52	0.00001	0.00022	ARR16	response regulator 16
	AT2G17710	427	-2.72	-3.00	0.62	-4.40	0.00001	0.00034	NA	NA
	AT1G22910	960	-2.72	-3.01	0.63	-4.34	0.00001	0.00043	NA	NA
	AT1G44414	26	-2.72	-3.53	0.89	-3.06	0.00224	0.01875	NA	NA
	AT5G15190	45	-2.73	-3.29	0.81	-3.37	0.00075	0.00839	NA	NA
	AT1G72630	2089	-2.73	-2.92	0.53	-5.14	0.00000	0.00002	ELF4-L2	ELF4-like 2
	AT3G22235	41134	-2.73	-3.27	0.80	-3.43	0.00060	0.00718	NA	NA
	AT5G16360	39	-2.73	-3.32	0.82	-3.34	0.00083	0.00902	NA	NA
	AT2G22810	31	-2.73	-3.25	0.79	-3.46	0.00054	0.00661	ACC4	1-AMINOCYCLOPROPANE-1-CARBOXYLIC ACID SYNTHASE POLYPEPTIDE
	AT1G26762	48	-2.73	-3.10	0.69	-3.96	0.00007	0.00147	NA	NA
	AT5G02640	24	-2.73	-3.32	0.82	-3.34	0.00083	0.00900	NA	NA
	AT5G50760	14	-2.73	-3.66	0.92	-2.96	0.00304	0.02363	SAUR55	SMALL AUXIN UPREGULATED RNA 55
	AT1G29460	871	-2.73	-2.96	0.57	-4.75	0.00000	0.00009	SAUR65	SMALL AUXIN UPREGULATED RNA 65
	AT2G17450	9027	-2.73	-2.94	0.55	-4.99	0.00000	0.00003	RHA3A	RING-H2 finger A3A
	AT1G61165	12	-2.73	-3.96	0.97	-2.81	0.00500	0.03363	NA	NA
	AT1G68585	883	-2.73	-2.96	0.57	-4.81	0.00000	0.00007	NA	NA
	AT1G21830	365	-2.73	-2.91	0.51	-5.39	0.00000	0.00001	NA	NA
	AT5G15160	253	-2.73	-2.95	0.56	-4.90	0.00000	0.00005	BHLH134	BASIC HELIX-LOOP-HELIX PROTEIN 134
	AT5G12140	8338	-2.73	-2.94	0.55	-4.97	0.00000	0.00004	ATCYS1	cystatin-1
	AT5G43150	1276	-2.74	-2.97	0.58	-4.74	0.00000	0.00009	NA	NA
	AT3G01940	75	-2.74	-3.24	0.77	-3.54	0.00041	0.00533	NA	NA

Supplemental Table 1-RNA seq data comparing Pro35S::MYB63 with wild type.

DAP.MYB63.target(1.5kb upstream TSS)	Gene	baseMean	log2FoldChange	lfcMLE	lfcSE	stat	pvalue	padj (FDR)	TAIR10 Symbol	TAIR10 Annotation (Short)
YES	AT4G01480	664	-2.74	-3.00	0.60	-4.53	0.00001	0.00021	AtPPa5	pyrophosphorylase 5
YES	AT1G28480	260	-2.74	-3.04	0.64	-4.31	0.00002	0.00048	GRX480	0
	AT1G14290	1525	-2.74	-3.00	0.60	-4.55	0.00001	0.00019	SBH2	sphingoid base hydroxylase 2
	AT1G69880	38	-2.74	-3.30	0.80	-3.42	0.00063	0.00736	ATH8	thioredoxin H-type 8
	AT4G37990	116	-2.74	-3.20	0.75	-3.66	0.00026	0.00374	ATCAD8	ARABIDOPSIS THALIANA CINNAMYL-ALCOHOL DEHYDROGENASE 8
	AT1G22190	3552	-2.74	-3.00	0.60	-4.56	0.00001	0.00019	RAP2.4	related to AP2 4
	AT2G05310	6563	-2.74	-2.97	0.58	-4.76	0.00000	0.00009	NA	NA
	AT4G20970	31	-2.74	-3.25	0.78	-3.53	0.00042	0.00548	NA	NA
	AT5G66650	168	-2.74	-3.20	0.75	-3.65	0.00027	0.00385	NA	NA
	AT1G21840	250	-2.74	-3.24	0.77	-3.57	0.00036	0.00492	UREF	urease accessory protein F
	AT1G24405	16	-2.75	-3.62	0.91	-3.02	0.00251	0.02052	NA	NA
	AT1G58280	169	-2.75	-3.06	0.65	-4.25	0.00002	0.00059	NA	NA
	AT4G24370	1074	-2.75	-2.99	0.59	-4.68	0.00000	0.00012	NA	NA
	AT1G47510	32	-2.75	-3.29	0.79	-3.49	0.00048	0.00610	5PTASE11	inositol polyphosphate 5-phosphatase 11
	AT1G24210	66	-2.75	-3.25	0.77	-3.57	0.00035	0.00481	NA	NA
	AT3G47100	7	-2.76	-4.26	1.00	-2.74	0.00609	0.03884	NA	NA
	AT5G65080	12	-2.76	-3.88	0.96	-2.87	0.00412	0.02921	AGL68	AGAMOUS-like 68
	AT5G59030	3987	-2.76	-2.96	0.53	-5.17	0.00000	0.00002	COPT1	copper transporter 1
	AT3G22961	26	-2.76	-3.39	0.83	-3.32	0.00089	0.00948	NA	NA
	AT5G17870	20784	-2.76	-2.99	0.57	-4.86	0.00000	0.00006	PSRP6	plastid-specific 50S ribosomal protein 6
	AT5G55850	5997	-2.76	-2.98	0.55	-5.01	0.00000	0.00003	NOI	0
	AT1G25425	184	-2.76	-2.97	0.54	-5.09	0.00000	0.00002	CLE43	CLAVATA3/ESR-RELATED 43
YES	AT5G40500	2423	-2.77	-3.03	0.60	-4.57	0.00000	0.00018	NA	NA
	AT2G27290	4847	-2.77	-2.98	0.56	-4.95	0.00000	0.00004	NA	NA
	AT1G19200	89	-2.77	-3.30	0.79	-3.52	0.00043	0.00559	NA	NA
	AT3G29140	112	-2.77	-3.23	0.75	-3.70	0.00022	0.00336	NA	NA
	AT4G34410	28	-2.78	-3.34	0.80	-3.46	0.00053	0.00655	RRTF1	redox responsive transcription factor 1
	AT5G24510	8	-2.78	-4.51	1.02	-2.71	0.00672	0.04174	NA	NA
	AT5G05630	70	-2.78	-3.21	0.73	-3.79	0.00015	0.00251	PUT3	POLYAMINE UPTAKE TRANSPORTER 3
YES	AT1G31812	26666	-2.78	-2.97	0.53	-5.28	0.00000	0.00001	ACBP	ACYL-COA-BINDING PROTEIN
	AT5G12235	12	-2.78	-3.77	0.93	-2.98	0.00290	0.02280	CLE22	CLAVATA3/ESR-RELATED 22
	AT2G21970	3468	-2.78	-3.02	0.58	-4.76	0.00000	0.00009	42249	stress enhanced protein 2
	AT5G03995	194	-2.78	-3.07	0.62	-4.47	0.00001	0.00026	NA	NA
	AT5G38120	165	-2.79	-2.98	0.53	-5.22	0.00000	0.00001	4CL8	0
	AT2G43540	809	-2.79	-3.07	0.62	-4.53	0.00001	0.00021	NA	NA
	AT5G63500	1075	-2.79	-3.02	0.57	-4.93	0.00000	0.00004	NA	NA
	AT3G45960	54	-2.79	-3.34	0.79	-3.51	0.00045	0.00569	ATEXLA3	expansin-like A3
	AT3G27025	29	-2.79	-3.41	0.82	-3.40	0.00068	0.00785	NA	NA
	AT2G30432	97	-2.79	-3.11	0.64	-4.35	0.00001	0.00041	TCL1	TRICHOMELESS1
	AT5G41050	1806	-2.80	-3.01	0.55	-5.12	0.00000	0.00002	NA	NA
	AT5G52900	946	-2.80	-2.98	0.51	-5.44	0.00000	0.00000	MAKR6	MEMBRANE-ASSOCIATED KINASE REGULATOR 6
	AT2G18969	24	-2.80	-3.44	0.83	-3.36	0.00078	0.00864	NA	NA
	AT3G21055	114669	-2.80	-3.02	0.56	-5.03	0.00000	0.00003	PSBTN	photosystem II subunit T
	AT1G23965	18	-2.80	-3.59	0.88	-3.16	0.00156	0.01430	NA	NA
	AT2G34925	90	-2.80	-3.27	0.74	-3.76	0.00017	0.00273	CLE42	CLAVATA3/ESR-RELATED 42
	AT3G06890	87	-2.80	-3.09	0.62	-4.50	0.00001	0.00024	NA	NA
	AT1G29450	1125	-2.80	-3.05	0.59	-4.78	0.00000	0.00008	SAUR64	SMALL AUXIN UPREGULATED RNA 64
	AT1G65490	3338	-2.80	-3.10	0.63	-4.43	0.00001	0.00030	NA	NA
	AT1G19380	2651	-2.80	-3.04	0.58	-4.87	0.00000	0.00006	NA	NA
	AT1G70760	2669	-2.80	-3.15	0.67	-4.17	0.00003	0.00074	CRR23	CHLORORESPIRATORY REDUCTION 23
	AT2G29170	372	-2.80	-3.07	0.61	-4.63	0.00000	0.00014	NA	NA
	AT3G22231	27949	-2.80	-3.01	0.54	-5.21	0.00000	0.00001	PCC1	PATHOGEN AND CIRCADIAN CONTROLLED 1
	AT4G23410	67	-2.81	-3.17	0.68	-4.14	0.00004	0.00085	TET5	tetraspanin5
	AT2G38480	601	-2.81	-3.03	0.55	-5.10	0.00000	0.00002	NA	NA
	AT1G34640	510	-2.81	-3.31	0.76	-3.68	0.00023	0.00350	NA	NA
	AT3G19550	347	-2.81	-3.04	0.56	-4.99	0.00000	0.00003	NA	NA
	AT2G20835	129	-2.82	-3.10	0.61	-4.58	0.00000	0.00017	NA	NA
	AT4G34190	11930	-2.82	-3.05	0.57	-4.98	0.00000	0.00003	42248	stress enhanced protein 1
	AT4G23600	469	-2.82	-3.02	0.53	-5.31	0.00000	0.00001	CORI3	CORONATINE INDUCED 1
	AT1G22690	4350	-2.83	-3.06	0.57	-4.94	0.00000	0.00004	NA	NA
	AT4G36900	403	-2.83	-3.04	0.55	-5.17	0.00000	0.00002	DEAR4	DREB AND EAR MOTIF PROTEIN 4
	AT3G54363	84	-2.83	-3.29	0.74	-3.80	0.00014	0.00244	NA	NA
	AT3G18690	1381	-2.83	-3.09	0.59	-4.80	0.00000	0.00007	MKS1	MAP kinase substrate 1

Supplemental Table 1-RNA seq data comparing Pro35S::MYB63 with wild type.

DAP.MYB63.target(1.5kb upstream TSS)	Gene	baseMean	log2FoldChange	lfcMLE	lfcSE	stat	pvalue	padj (FDR)	TAIR10 Symbol	TAIR10 Annotation (Short)
	AT1G28070	417	-2.83	-3.10	0.60	-4.71	0.00000	0.00011	NA	NA
	AT1G77540	1352	-2.83	-3.05	0.55	-5.12	0.00000	0.00002	NA	NA
	AT5G45680	4721	-2.83	-3.09	0.59	-4.81	0.00000	0.00007	ATFKBP13	FK506 BINDING PROTEIN 13
	AT5G47960	49	-2.83	-3.40	0.80	-3.56	0.00037	0.00502	ATRABA4C	RAB GTPase homolog A4C
	AT3G52105	2051	-2.83	-3.08	0.58	-4.91	0.00000	0.00005	NA	NA
	AT5G65870	496	-2.84	-3.09	0.59	-4.82	0.00000	0.00007	ATPSK5	phytosulfokine 5 precursor
	AT5G18080	585	-2.84	-3.09	0.58	-4.88	0.00000	0.00005	SAUR24	small auxin up RNA 24
	AT5G60460	728	-2.84	-3.28	0.73	-3.91	0.00009	0.00174	NA	NA
	AT1G80520	79	-2.84	-3.37	0.78	-3.66	0.00025	0.00370	NA	NA
	AT1G71890	22	-2.84	-3.56	0.85	-3.33	0.00088	0.00942	ATSUC5	SUCROSE-PROTON SYMPORTER 5
	AT1G60190	28	-2.84	-3.46	0.82	-3.48	0.00051	0.00631	AtPUB19	0
YES	AT4G15210	57	-2.84	-3.50	0.82	-3.46	0.00054	0.00658	AT-BETA-AI	0
	AT1G06160	532	-2.84	-3.02	0.50	-5.68	0.00000	0.00000	ORA59	octadecanoid-responsive Arabidopsis AP2/ERF 59
	AT1G07590	43729	-2.84	-3.05	0.54	-5.22	0.00000	0.00001	NA	NA
	AT2G04039	6124	-2.84	-3.11	0.60	-4.78	0.00000	0.00008	NA	NA
	AT5G66040	14332	-2.84	-3.11	0.60	-4.72	0.00000	0.00010	STR16	sulfurtransferase protein 16
	AT5G25190	1356	-2.85	-3.06	0.54	-5.27	0.00000	0.00001	ESE3	ethylene and salt inducible 3
	AT3G63050	17	-2.85	-3.73	0.90	-3.17	0.00152	0.01404	NA	NA
	AT5G24155	28	-2.85	-3.52	0.84	-3.41	0.00065	0.00754	NA	NA
	AT4G25470	572	-2.85	-3.10	0.58	-4.92	0.00000	0.00004	ATCBF2	0
	AT4G13500	4309	-2.85	-3.08	0.56	-5.12	0.00000	0.00002	NA	NA
	AT3G14810	104	-2.85	-3.28	0.72	-3.95	0.00008	0.00152	MSL5	mechanosensitive channel of small conductance-like 5
	AT1G72510	2094	-2.85	-3.05	0.53	-5.39	0.00000	0.00001	NA	NA
	AT2G28305	822	-2.85	-3.20	0.67	-4.29	0.00002	0.00051	ATLOG1	0
	AT1G59950	12	-2.85	-4.20	0.99	-2.89	0.00382	0.02761	NA	NA
YES	AT5G48940	20	-2.85	-3.53	0.84	-3.41	0.00065	0.00753	NA	NA
YES	AT1G29500	739	-2.85	-3.12	0.60	-4.79	0.00000	0.00008	SAUR66	SMALL AUXIN UPREGULATED RNA 66
	AT2G23120	14801	-2.86	-3.07	0.55	-5.22	0.00000	0.00001	NA	NA
	AT2G26070	559	-2.86	-3.20	0.67	-4.29	0.00002	0.00051	AtRTE1	0
	AT3G19660	529	-2.86	-3.13	0.61	-4.71	0.00000	0.00010	NA	NA
	AT2G46780	255	-2.86	-3.13	0.60	-4.77	0.00000	0.00008	NA	NA
YES	AT3G16720	3808	-2.87	-3.17	0.63	-4.56	0.00001	0.00019	ATL2	TOXICOS EN LEVADURA 2
	AT3G21351	28	-2.87	-3.46	0.81	-3.55	0.00038	0.00507	NA	NA
	AT1G75880	168	-2.87	-3.26	0.70	-4.10	0.00004	0.00095	NA	NA
	AT3G28420	58	-2.87	-3.35	0.75	-3.82	0.00014	0.00235	NA	NA
	AT3G15115	217	-2.87	-3.19	0.64	-4.45	0.00001	0.00028	NA	NA
	AT3G14395	14	-2.87	-3.75	0.90	-3.20	0.00137	0.01294	NA	NA
	AT5G38610	186	-2.87	-3.37	0.76	-3.77	0.00016	0.00267	NA	NA
	AT3G61930	12	-2.87	-4.13	0.97	-2.97	0.00300	0.02342	NA	NA
YES	AT1G07450	28	-2.87	-3.44	0.79	-3.62	0.00029	0.00418	NA	NA
	AT3G06780	1436	-2.87	-3.12	0.57	-5.02	0.00000	0.00003	NA	NA
	AT3G11690	580	-2.88	-3.08	0.53	-5.43	0.00000	0.00000	NA	NA
	AT4G03380	13	-2.88	-4.26	0.99	-2.92	0.00350	0.02607	NA	NA
	AT1G76190	38	-2.88	-3.42	0.78	-3.69	0.00022	0.00338	SAUR56	SMALL AUXIN UPREGULATED RNA 56
	AT5G43580	108	-2.88	-3.43	0.79	-3.67	0.00025	0.00366	UPI	UNUSUAL SERINE PROTEASE INHIBITOR
	AT2G17705	961	-2.88	-3.14	0.59	-4.88	0.00000	0.00005	NA	NA
	AT5G67520	116	-2.88	-3.37	0.75	-3.82	0.00013	0.00229	APK4	adenosine-5'-phosphosulfate (APS) kinase 4
	AT1G19210	7	-2.88	-5.94	1.06	-2.72	0.00647	0.04061	NA	NA
	AT2G45170	5213	-2.89	-3.16	0.60	-4.77	0.00000	0.00008	ATATG8E	AUTOPHAGY 8E
	AT1G16022	21	-2.89	-3.57	0.83	-3.47	0.00053	0.00648	NA	NA
	AT3G45970	1043	-2.89	-3.17	0.60	-4.81	0.00000	0.00007	ATEXLA1	expansin-like A1
	AT2G18320	7	-2.89	-4.73	1.02	-2.84	0.00449	0.03108	NA	NA
	AT1G52270	157	-2.89	-3.39	0.75	-3.83	0.00013	0.00221	NA	NA
	AT1G66690	26	-2.90	-3.52	0.81	-3.57	0.00036	0.00484	NA	NA
	AT1G66500	471	-2.90	-3.18	0.60	-4.80	0.00000	0.00007	NA	NA
	AT5G55250	22	-2.90	-3.52	0.81	-3.56	0.00036	0.00494	AtIAMT1	0
	AT5G56980	874	-2.90	-3.11	0.53	-5.45	0.00000	0.00000	NA	NA
	AT5G01880	141	-2.90	-3.37	0.74	-3.94	0.00008	0.00156	DAFL2	DAF-Like gene 2
	AT3G03190	407	-2.91	-3.11	0.53	-5.51	0.00000	0.00000	ATGSTF11	glutathione S-transferase F11
	AT1G19670	2857	-2.91	-3.17	0.59	-4.90	0.00000	0.00005	ATCLH1	chlorophyllase 1
	AT4G05010	150	-2.91	-3.26	0.67	-4.36	0.00001	0.00039	AtFBS3	0
	AT1G68765	39	-2.91	-3.61	0.84	-3.45	0.00056	0.00674	IDA	INFLORESCENCE DEFICIENT IN ABSCISSION
	AT5G21020	24278	-2.91	-3.34	0.72	-4.05	0.00005	0.00113	NA	NA
	AT3G15500	306	-2.91	-3.16	0.58	-5.04	0.00000	0.00003	ANAC055	NAC domain containing protein 55
YES	AT2G32090	2798	-2.91	-3.15	0.57	-5.14	0.00000	0.00002	NA	NA
	AT4G12410	8	-2.91	-4.38	0.99	-2.94	0.00325	0.02468	SAUR35	SMALL AUXIN UPREGULATED RNA 35

Supplemental Table 1-RNA seq data comparing Pro35S::MYB63 with wild type.

DAP.MYB63.target(1.5kb upstream TSS)	Gene	baseMean	log2FoldChange	lfcMLE	lfcSE	stat	pvalue	padj (FDR)	TAIR10 Symbol	TAIR10 Annotation (Short)
	AT2G30560	8	-2.92	-4.86	1.02	-2.85	0.00434	0.03031	NA	NA
YES	AT5G14410	485	-2.92	-3.16	0.57	-5.15	0.00000	0.00002	NA	NA
	AT4G37220	14	-2.92	-3.93	0.92	-3.16	0.00157	0.01443	NA	NA
	AT1G06980	78	-2.92	-3.36	0.73	-4.02	0.00006	0.00123	NA	NA
	AT4G30430	14	-2.92	-3.79	0.89	-3.27	0.00106	0.01077	TET9	tetraspanin9
	AT2G43340	430	-2.92	-3.20	0.61	-4.83	0.00000	0.00007	NA	NA
	AT5G09225	1050	-2.92	-3.16	0.56	-5.22	0.00000	0.00001	NA	NA
	AT2G46650	4935	-2.92	-3.13	0.54	-5.45	0.00000	0.00000	ATCB5-C	ARABIDOPSIS CYTOCHROME B5 ISOFORM C
	AT3G15210	5450	-2.92	-3.17	0.57	-5.09	0.00000	0.00002	ATERF-4	ETHYLENE RESPONSIVE ELEMENT BINDING FACTOR 4
	AT5G12880	12	-2.93	-4.08	0.95	-3.09	0.00198	0.01706	NA	NA
	AT4G15700	1779	-2.93	-3.19	0.59	-4.96	0.00000	0.00004	NA	NA
	AT1G09180	49	-2.93	-3.47	0.77	-3.79	0.00015	0.00254	ATSAR1	SECRETION-ASSOCIATED RAS 1
	AT4G15800	4396	-2.93	-3.19	0.58	-5.02	0.00000	0.00003	RALFL33	ralf-like 33
	AT3G05880	2715	-2.93	-3.20	0.60	-4.92	0.00000	0.00004	RCI2A	RARE-COLD-INDUCIBLE 2A
	AT3G05937	246	-2.94	-3.16	0.55	-5.35	0.00000	0.00001	NA	NA
	AT3G23170	1196	-2.94	-3.17	0.56	-5.27	0.00000	0.00001	NA	NA
	AT1G10770	13	-2.94	-3.89	0.91	-3.22	0.00127	0.01219	NA	NA
	AT1G65295	765	-2.94	-3.18	0.56	-5.22	0.00000	0.00001	NA	NA
	AT5G14110	61	-2.94	-3.39	0.72	-4.11	0.00004	0.00093	NA	NA
	AT5G53980	8	-2.94	-4.80	1.01	-2.90	0.00370	0.02703	ATHB52	homeobox protein 52
	AT3G16900	10	-2.94	-4.20	0.96	-3.07	0.00211	0.01797	NA	NA
	AT2G02130	3758	-2.95	-3.16	0.54	-5.47	0.00000	0.00000	LCR68	low-molecular-weight cysteine-rich 68
	AT4G01200	157	-2.95	-3.44	0.75	-3.93	0.00008	0.00161	NA	NA
YES	AT1G72240	177	-2.95	-3.23	0.60	-4.92	0.00000	0.00005	NA	NA
	AT3G10985	7589	-2.95	-3.38	0.71	-4.16	0.00003	0.00076	ATWI-12	ARABIDOPSIS THALIANA WOUND-INDUCED PROTEIN 12
	AT2G46940	52	-2.96	-3.65	0.84	-3.54	0.00040	0.00530	NA	NA
	AT3G22415	164	-2.96	-3.32	0.66	-4.48	0.00001	0.00026	NA	NA
	AT1G11500	30	-2.96	-3.52	0.78	-3.79	0.00015	0.00254	NA	NA
	AT2G40530	357	-2.96	-3.28	0.63	-4.70	0.00000	0.00011	NA	NA
	AT1G55675	2818	-2.96	-3.23	0.59	-4.98	0.00000	0.00003	NA	NA
	AT3G05490	871	-2.96	-3.24	0.60	-4.97	0.00000	0.00004	RALFL22	ralf-like 22
YES	AT3G60550	37	-2.97	-3.64	0.83	-3.58	0.00034	0.00472	CYCP3;2	cyclin p3;2
	AT1G52342	2007	-2.97	-3.20	0.56	-5.31	0.00000	0.00001	NA	NA
	AT5G16490	125	-2.97	-3.48	0.76	-3.91	0.00009	0.00173	RIC4	ROP-interactive CRIB motif-containing protein 4
	AT2G43530	2774	-2.97	-3.20	0.55	-5.37	0.00000	0.00001	NA	NA
	AT3G44020	1405	-2.97	-3.26	0.61	-4.84	0.00000	0.00006	NA	NA
	AT1G79040	439722	-2.97	-3.21	0.56	-5.31	0.00000	0.00001	PSBR	photosystem II subunit R
	AT1G11850	1097	-2.97	-3.16	0.50	-5.94	0.00000	0.00000	NA	NA
	AT5G59080	4632	-2.97	-3.20	0.55	-5.44	0.00000	0.00000	NA	NA
	AT4G15440	130	-2.98	-3.45	0.74	-4.03	0.00006	0.00119	CYP74B2	0
	AT4G34790	439	-2.98	-3.24	0.58	-5.10	0.00000	0.00002	SAUR3	SMALL AUXIN UPREGULATED RNA 3
	AT4G36110	231	-2.98	-3.50	0.76	-3.91	0.00009	0.00172	SAUR9	SMALL AUXIN UPREGULATED RNA 9
	AT4G26288	145	-2.98	-3.30	0.63	-4.69	0.00000	0.00011	NA	NA
	AT1G01453	7	-2.98	-4.76	1.01	-2.96	0.00304	0.02363	NA	NA
	AT5G04150	234	-2.99	-3.53	0.77	-3.87	0.00011	0.00199	BHLH101	0
	AT4G28700	17	-2.99	-3.82	0.88	-3.41	0.00065	0.00751	AMT1;4	ammonium transporter 1;4
	AT5G47240	1397	-2.99	-3.23	0.56	-5.31	0.00000	0.00001	atnudt8	nudix hydrolase homolog 8
	AT2G37330	272	-2.99	-3.51	0.76	-3.93	0.00008	0.00162	ALS3	ALUMINUM SENSITIVE 3
	AT3G57450	4270	-2.99	-3.28	0.61	-4.92	0.00000	0.00004	NA	NA
	AT3G02730	25143	-2.99	-3.28	0.61	-4.92	0.00000	0.00005	ATF1	0
	AT3G13520	3879	-2.99	-3.20	0.53	-5.61	0.00000	0.00000	AGP12	arabinogalactan protein 12
	AT5G60660	179	-2.99	-3.48	0.74	-4.04	0.00005	0.00114	PIP2;4	plasma membrane intrinsic protein 2;4
	AT4G30660	1946	-2.99	-3.29	0.62	-4.87	0.00000	0.00006	NA	NA
	AT5G65610	1118	-3.00	-3.21	0.53	-5.61	0.00000	0.00000	NA	NA
	AT1G58420	275	-3.00	-3.35	0.65	-4.62	0.00000	0.00015	NA	NA
	AT2G26020	472	-3.00	-3.23	0.55	-5.47	0.00000	0.00000	PDF1.2b	plant defensin 1.2b
	AT5G66800	84	-3.01	-3.51	0.75	-3.99	0.00007	0.00135	NA	NA
	AT3G61640	1480	-3.01	-3.37	0.67	-4.51	0.00001	0.00023	AGP20	arabinogalactan protein 20
	AT1G19610	520	-3.01	-3.40	0.68	-4.40	0.00001	0.00034	LCR78	LOW-MOLECULAR-WEIGHT CYSTEINE-RICH 78
	AT1G11570	47	-3.01	-3.61	0.80	-3.78	0.00015	0.00257	NTL	NTF2-like
	AT5G43620	280	-3.01	-3.34	0.64	-4.73	0.00000	0.00010	NA	NA
	AT3G21260	210	-3.01	-3.53	0.76	-3.98	0.00007	0.00138	GLTP3	GLYCOLIPID TRANSFER PROTEIN 3
	AT2G27960	1762	-3.01	-3.30	0.60	-5.02	0.00000	0.00003	CKS1	cyclin-dependent kinase-subunit 1
	AT5G14550	755	-3.02	-3.26	0.56	-5.39	0.00000	0.00001	NA	NA

Supplemental Table 1-RNA seq data comparing Pro35S::MYB63 with wild type.

DAP.MYB63.target(1.5kb upstream TSS)	Gene	baseMean	log2FoldChange	lfcMLE	lfcSE	stat	pvalue	padj (FDR)	TAIR10 Symbol	TAIR10 Annotation (Short)
	AT4G13195	200	-3.02	-3.25	0.55	-5.53	0.00000	0.00000	CLE44	CLAVATA3/ESR-RELATED 44
	AT2G05632	62	-3.02	-3.61	0.79	-3.83	0.00013	0.00226	NA	NA
	AT1G64360	1810	-3.03	-4.63	1.00	-3.02	0.00251	0.02049	NA	NA
	AT4G11211	792	-3.04	-3.29	0.57	-5.31	0.00000	0.00001	NA	NA
	AT2G27180	25	-3.04	-3.75	0.84	-3.64	0.00028	0.00397	NA	NA
	AT1G76600	3032	-3.04	-3.25	0.53	-5.74	0.00000	0.00000	NA	NA
	AT4G24380	201	-3.04	-3.36	0.63	-4.86	0.00000	0.00006	NA	NA
	AT5G49480	944	-3.04	-3.24	0.52	-5.89	0.00000	0.00000	ATCP1	Ca2+-binding protein 1
YES	AT1G09500	79	-3.05	-3.61	0.78	-3.93	0.00009	0.00164	NA	NA
	AT5G44580	9820	-3.05	-3.30	0.57	-5.34	0.00000	0.00001	NA	NA
	AT5G23730	146	-3.05	-3.43	0.67	-4.54	0.00001	0.00020	EFO2	EARLY FLOWERING BY OVEREXPRESSION 2
	AT5G52190	937	-3.05	-3.38	0.63	-4.82	0.00000	0.00007	NA	NA
	AT1G75030	490	-3.05	-3.28	0.55	-5.59	0.00000	0.00000	ATLP-3	thaumatin-like protein 3
	AT4G18280	458	-3.05	-3.26	0.53	-5.77	0.00000	0.00000	NA	NA
	AT1G67860	6314	-3.05	-3.30	0.56	-5.42	0.00000	0.00000	NA	NA
	AT2G27830	5667	-3.05	-3.28	0.54	-5.65	0.00000	0.00000	NA	NA
	AT4G17490	1131	-3.05	-3.27	0.54	-5.69	0.00000	0.00000	ATERF6	ethylene responsive element binding factor 6
	AT4G36500	9300	-3.06	-3.33	0.58	-5.23	0.00000	0.00001	NA	NA
	AT5G04000	16	-3.06	-4.01	0.90	-3.42	0.00063	0.00741	NA	NA
	AT3G28310	18	-3.07	-4.02	0.90	-3.41	0.00065	0.00751	NA	NA
	AT3G49340	31	-3.07	-3.83	0.85	-3.62	0.00029	0.00412	NA	NA
	AT3G14560	208	-3.07	-3.34	0.58	-5.31	0.00000	0.00001	NA	NA
	AT1G52880	719	-3.08	-3.33	0.57	-5.37	0.00000	0.00001	ANAC018	Arabidopsis NAC domain containing protein 18
	AT5G16600	19	-3.08	-3.94	0.87	-3.52	0.00043	0.00559	AtMYB43	myb domain protein 43
	AT2G15760	45	-3.08	-3.58	0.74	-4.15	0.00003	0.00080	NA	NA
	AT3G24420	523	-3.08	-3.37	0.60	-5.14	0.00000	0.00002	NA	NA
	ATCG00630	14351	-3.09	-3.32	0.55	-5.61	0.00000	0.00000	PSAJ	0
	AT5G37770	6242	-3.09	-3.32	0.55	-5.60	0.00000	0.00000	CML24	CALMODULIN-LIKE 24
	AT2G27229	7	-3.09	-5.57	1.03	-3.00	0.00274	0.02175	NA	NA
	AT1G19780	31	-3.09	-3.67	0.78	-3.96	0.00008	0.00147	ATCNGC8	cyclic nucleotide gated channel 8
	AT2G27385	14194	-3.09	-3.39	0.60	-5.12	0.00000	0.00002	NA	NA
	AT1G02350	334	-3.10	-3.39	0.60	-5.15	0.00000	0.00002	NA	NA
	AT1G77870	101	-3.10	-3.60	0.74	-4.19	0.00003	0.00070	MUB5	membrane-anchored ubiquitin-fold protein 5 precursor
	AT5G44578	106	-3.10	-3.50	0.68	-4.56	0.00001	0.00019	NA	NA
	AT5G06480	144	-3.10	-3.65	0.77	-4.05	0.00005	0.00113	NA	NA
	AT1G07500	7	-3.10	-5.58	1.03	-3.01	0.00263	0.02117	SMR5	SIAMESE-RELATED 5
	AT2G46330	16805	-3.10	-3.47	0.66	-4.71	0.00000	0.00010	AGP16	arabinogalactan protein 16
	AT5G55010	19	-3.10	-4.00	0.88	-3.51	0.00045	0.00573	NA	NA
	AT5G24165	10052	-3.11	-3.36	0.57	-5.50	0.00000	0.00000	NA	NA
	AT5G46950	6	-3.11	-8.55	1.06	-2.92	0.00346	0.02589	NA	NA
	AT5G44430	634	-3.11	-3.34	0.53	-5.83	0.00000	0.00000	PDF1.2c	plant defensin 1.2C
	AT1G48745	387	-3.11	-3.35	0.54	-5.73	0.00000	0.00000	NA	NA
	AT5G65300	89	-3.11	-3.56	0.71	-4.39	0.00001	0.00035	NA	NA
	AT4G01830	10	-3.12	-5.08	1.01	-3.08	0.00207	0.01768	ABCB5	ATP-binding cassette B5
	AT1G78410	814	-3.12	-3.42	0.61	-5.15	0.00000	0.00002	NA	NA
	AT1G07901	81	-3.12	-3.61	0.73	-4.29	0.00002	0.00051	NA	NA
	AT5G44420	1289	-3.13	-3.40	0.58	-5.42	0.00000	0.00000	LCR77	LOW-MOLECULAR-WEIGHT CYSTEINE-RICH 77
	AT1G06830	1014	-3.13	-3.36	0.54	-5.81	0.00000	0.00000	NA	NA
	AT2G47780	88	-3.13	-3.50	0.65	-4.79	0.00000	0.00008	NA	NA
	AT1G56650	52	-3.13	-3.66	0.75	-4.16	0.00003	0.00076	ATMYB75	MYB DOMAIN PROTEIN 75
	AT3G03820	872	-3.13	-3.36	0.54	-5.77	0.00000	0.00000	SAUR29	SMALL AUXIN UP RNA 29
	AT3G63088	80	-3.14	-3.65	0.74	-4.23	0.00002	0.00063	DVL14	DEVIL 14
	AT3G10150	16	-3.14	-4.08	0.89	-3.53	0.00042	0.00546	ATPAP16	0
	AT2G18328	2600	-3.15	-3.40	0.57	-5.54	0.00000	0.00000	ATRL4	RAD-like 4
	AT1G32920	10550	-3.15	-3.42	0.58	-5.46	0.00000	0.00000	NA	NA
	AT1G24148	1403	-3.16	-3.48	0.62	-5.08	0.00000	0.00002	NA	NA
	AT5G04080	486	-3.16	-3.38	0.53	-5.96	0.00000	0.00000	NA	NA
	AT3G43720	4857	-3.16	-3.42	0.57	-5.59	0.00000	0.00000	LTPG2	glycosylphosphatidylinositol-anchored lipid protein transfer 2
	AT3G16240	71455	-3.16	-3.42	0.57	-5.59	0.00000	0.00000	AQP1	0
	AT2G25735	3781	-3.16	-3.43	0.57	-5.55	0.00000	0.00000	NA	NA
	AT1G23350	10	-3.17	-4.69	0.98	-3.24	0.00118	0.01161	NA	NA
	AT2G22122	224	-3.17	-3.65	0.72	-4.38	0.00001	0.00037	NA	NA
	AT3G55646	509	-3.17	-3.40	0.53	-5.94	0.00000	0.00000	NA	NA
	AT3G47295	2405	-3.17	-3.41	0.54	-5.83	0.00000	0.00000	NA	NA
	AT1G69970	45	-3.18	-3.73	0.76	-4.18	0.00003	0.00072	CLE26	CLAVATA3/ESR-RELATED 26

Supplemental Table 1-RNA seq data comparing Pro35S::MYB63 with wild type.

DAP MYB63.target(1.5kb upstream TSS)	Gene	baseMean	log2FoldChange	lfcMLE	lfcSE	stat	pvalue	padj (FDR)	TAIR10 Symbol	TAIR10 Annotation (Short)
	AT1G29510	876	-3.18	-3.50	0.62	-5.14	0.00000	0.00002	SAUR67	SMALL AUXIN UPREGULATED RNA 67
	AT3G54120	149	-3.18	-3.69	0.74	-4.31	0.00002	0.00048	NA	NA
	AT1G66370	8	-3.18	-4.96	0.99	-3.20	0.00136	0.01287	AtMYB113	myb domain protein 113
	AT2G43510	991	-3.19	-3.51	0.62	-5.15	0.00000	0.00002	ATTI1	trypsin inhibitor protein 1
	AT3G46490	106	-3.19	-3.70	0.74	-4.34	0.00001	0.00042	NA	NA
	AT5G40630	19	-3.19	-4.09	0.87	-3.67	0.00024	0.00363	NA	NA
	AT5G49170	298	-3.20	-3.52	0.62	-5.16	0.00000	0.00002	NA	NA
	AT1G78020	8270	-3.20	-3.44	0.55	-5.83	0.00000	0.00000	NA	NA
	AT2G30760	64	-3.20	-3.77	0.77	-4.17	0.00003	0.00074	NA	NA
	AT1G73500	3072	-3.20	-3.41	0.52	-6.16	0.00000	0.00000	ATMKK9	0
	AT4G25490	240	-3.20	-3.55	0.63	-5.05	0.00000	0.00003	ATCBF1	0
	AT5G22580	3419	-3.20	-3.42	0.52	-6.13	0.00000	0.00000	NA	NA
	AT2G18010	42	-3.21	-3.77	0.76	-4.21	0.00003	0.00066	SAUR10	SMALL AUXIN UPREGULATED RNA 10
	AT4G38850	236	-3.21	-3.52	0.60	-5.33	0.00000	0.00001	ATSAUR15	ARABIDOPSIS THALIANA SMALL AUXIN UPREGULATED 15
	AT1G20190	2094	-3.21	-3.53	0.61	-5.23	0.00000	0.00001	ATEXP11	0
	AT4G37230	11	-3.21	-4.72	0.97	-3.31	0.00092	0.00968	NA	NA
	AT3G56290	2496	-3.21	-3.47	0.56	-5.75	0.00000	0.00000	NA	NA
	AT4G35783	86	-3.22	-3.78	0.76	-4.23	0.00002	0.00062	DVL17	DEVIL 17
	AT1G07485	8	-3.23	-5.73	1.03	-3.15	0.00165	0.01494	NA	NA
	AT1G18710	59	-3.24	-3.70	0.70	-4.60	0.00000	0.00016	AtMYB47	myb domain protein 47
	AT1G30757	195	-3.24	-3.86	0.79	-4.12	0.00004	0.00089	NA	NA
	AT3G28925	6	-3.24	-8.53	1.06	-3.06	0.00225	0.01881	NA	NA
	AT2G30360	497	-3.24	-3.48	0.54	-5.96	0.00000	0.00000	CIPK11	CBL-INTERACTING PROTEIN KINASE 11
	AT1G49780	1642	-3.24	-3.57	0.62	-5.24	0.00000	0.00001	PUB26	plant U-box 26
	AT5G58650	1493	-3.24	-3.51	0.57	-5.66	0.00000	0.00000	PSY1	plant peptide containing sulfated tyrosine 1
	AT3G10930	1282	-3.25	-3.59	0.63	-5.17	0.00000	0.00002	NA	NA
	AT1G13609	675	-3.25	-3.68	0.69	-4.72	0.00000	0.00010	NA	NA
	AT1G31350	648	-3.25	-3.58	0.62	-5.25	0.00000	0.00001	KUF1	KAR-UP F-box 1
	AT1G74929	47	-3.25	-3.86	0.78	-4.18	0.00003	0.00072	NA	NA
	AT1G29440	782	-3.26	-3.49	0.53	-6.13	0.00000	0.00000	SAUR63	SMALL AUXIN UP RNA 63
	AT3G27027	122	-3.26	-3.77	0.73	-4.46	0.00001	0.00027	NA	NA
	AT4G27652	730	-3.26	-3.59	0.62	-5.28	0.00000	0.00001	NA	NA
	AT3G28857	66	-3.27	-3.88	0.78	-4.18	0.00003	0.00072	PRE5	Paclobutrazol Resistance 5
	AT5G64660	1558	-3.28	-3.59	0.61	-5.41	0.00000	0.00001	ATCMPG2	0
	AT3G47965	211	-3.28	-3.87	0.77	-4.29	0.00002	0.00051	NA	NA
	AT1G53690	31	-3.29	-3.94	0.79	-4.16	0.00003	0.00077	NA	NA
	AT3G50900	203	-3.29	-3.84	0.75	-4.40	0.00001	0.00035	NA	NA
	AT2G06255	229	-3.29	-3.55	0.55	-5.98	0.00000	0.00000	ELF4-L3	ELF4-like 3
	AT5G24570	532	-3.30	-3.57	0.57	-5.83	0.00000	0.00000	NA	NA
	AT5G22545	31	-3.30	-3.95	0.79	-4.20	0.00003	0.00067	NA	NA
	AT2G42975	766	-3.30	-3.57	0.56	-5.86	0.00000	0.00000	NA	NA
	AT2G26010	400	-3.31	-3.87	0.76	-4.38	0.00001	0.00037	PDF1.3	plant defensin 1.3
	AT5G25990	245	-3.31	-3.63	0.61	-5.42	0.00000	0.00000	NA	NA
	AT3G21460	1303	-3.31	-3.56	0.54	-6.11	0.00000	0.00000	NA	NA
YES	AT1G55330	20561	-3.32	-3.53	0.51	-6.48	0.00000	0.00000	AGP21	arabinogalactan protein 21
	AT5G17460	720	-3.33	-3.59	0.55	-6.00	0.00000	0.00000	NA	NA
	AT1G61340	220	-3.33	-3.96	0.78	-4.26	0.00002	0.00055	AtFBS1	0
	AT3G56970	121	-3.35	-3.84	0.72	-4.67	0.00000	0.00012	BHLH038	0
	AT1G74670	24434	-3.35	-3.61	0.56	-6.00	0.00000	0.00000	GASA6	GA-stimulated Arabidopsis 6
	AT5G62280	89	-3.35	-3.79	0.69	-4.86	0.00000	0.00006	NA	NA
	AT4G20780	1244	-3.35	-3.65	0.59	-5.70	0.00000	0.00000	CML42	calmodulin like 42
	AT4G13395	1513	-3.36	-3.66	0.58	-5.77	0.00000	0.00000	DVL10	DEVIL 10
	AT5G05600	795	-3.36	-3.62	0.55	-6.13	0.00000	0.00000	NA	NA
	AT4G16515	519	-3.37	-3.61	0.54	-6.28	0.00000	0.00000	CLEL 6	CLE-like 6
	AT2G43550	2797	-3.37	-3.62	0.55	-6.15	0.00000	0.00000	NA	NA
	AT3G05935	829	-3.37	-3.60	0.52	-6.44	0.00000	0.00000	NA	NA
	AT4G17460	935	-3.37	-3.61	0.53	-6.32	0.00000	0.00000	HAT1	0
	AT3G25760	1612	-3.37	-3.68	0.59	-5.72	0.00000	0.00000	AOC1	allene oxide cyclase 1
	AT3G03180	232	-3.37	-3.92	0.74	-4.56	0.00001	0.00019	NA	NA
	AT5G48490	7034	-3.39	-3.62	0.53	-6.43	0.00000	0.00000	NA	NA
YES	AT5G35480	7630	-3.41	-3.73	0.60	-5.67	0.00000	0.00000	NA	NA
	AT1G17147	924	-3.41	-3.73	0.60	-5.69	0.00000	0.00000	NA	NA
	AT1G33102	119	-3.42	-3.77	0.62	-5.48	0.00000	0.00000	NA	NA
	AT4G24570	2669	-3.42	-3.70	0.57	-6.05	0.00000	0.00000	DIC2	dicarboxylate carrier 2
	AT2G41800	23	-3.43	-4.21	0.83	-4.15	0.00003	0.00081	NA	NA
	AT4G18980	27	-3.43	-4.14	0.80	-4.28	0.00002	0.00053	AtS40-3	AtS40-3
	AT3G49620	52	-3.43	-3.95	0.72	-4.76	0.00000	0.00009	DIN11	DARK INDUCIBLE 11
	AT1G31750	37	-3.44	-4.22	0.82	-4.19	0.00003	0.00070	NA	NA

Supplemental Table 1-RNA seq data comparing Pro35S::MYB63 with wild type.

DAP.MYB63.target(1.5kb upstream TSS)	Gene	baseMean	log2FoldChange	lfcMLE	lfcSE	stat	pvalue	padj (FDR)	TAIR10 Symbol	TAIR10 Annotation (Short)
	AT1G19960	6537	-3.44	-3.72	0.57	-6.06	0.00000	0.00000	NA	NA
	AT5G47230	741	-3.45	-3.72	0.55	-6.25	0.00000	0.00000	ATERF-5	ETHYLENE RESPONSIVE ELEMENT BINDING FACTOR- 5
	AT1G43160	6	-3.45	-8.58	1.05	-3.29	0.00101	0.01034	RAP2.6	related to AP2 6
	AT4G11780	8	-3.46	-6.05	1.02	-3.38	0.00071	0.00808	TRM10	TON1 Recruiting Motif 10
	AT5G14330	91	-3.46	-3.99	0.73	-4.75	0.00000	0.00009	NA	NA
	AT3G02480	76	-3.48	-3.98	0.71	-4.88	0.00000	0.00005	NA	NA
	AT1G35560	1324	-3.48	-3.88	0.65	-5.35	0.00000	0.00001	AtTCP23	0
	AT4G30280	86	-3.48	-4.06	0.75	-4.67	0.00000	0.00012	ATXTH18	XYLOGLUCAN ENDOTRANSGLUCOSYLASE/HYDROLASE 18
	AT5G59510	19	-3.48	-4.50	0.88	-3.95	0.00008	0.00154	DVL18	DEVIL 18
	AT3G18560	112	-3.49	-4.01	0.72	-4.88	0.00000	0.00005	NA	NA
	AT3G23230	39	-3.50	-4.09	0.75	-4.67	0.00000	0.00012	AtERF98	0
	AT4G39250	7	-3.50	-8.78	1.05	-3.33	0.00087	0.00932	ATRL1	RAD-like 1
	AT5G37760	12	-3.50	-4.93	0.94	-3.72	0.00020	0.00311	NA	NA
	AT5G01015	820	-3.50	-3.77	0.54	-6.45	0.00000	0.00000	NA	NA
	AT5G59320	284	-3.51	-4.16	0.78	-4.52	0.00001	0.00022	LTP3	lipid transfer protein 3
	AT5G54585	1235	-3.51	-3.79	0.56	-6.29	0.00000	0.00000	NA	NA
	AT1G24147	4842	-3.52	-3.85	0.60	-5.87	0.00000	0.00000	NA	NA
	AT3G19380	1916	-3.52	-3.85	0.60	-5.89	0.00000	0.00000	PUB25	plant U-box 25
	AT1G76650	670	-3.53	-3.97	0.67	-5.27	0.00000	0.00001	CML38	calmodulin-like 38
	AT3G61090	7	-3.54	-8.66	1.05	-3.37	0.00074	0.00830	NA	NA
	AT5G51680	10	-3.54	-6.19	1.02	-3.48	0.00049	0.00619	NA	NA
	AT5G52390	93	-3.54	-4.08	0.72	-4.89	0.00000	0.00005	NA	NA
	AT1G59920	63	-3.54	-4.00	0.68	-5.19	0.00000	0.00001	NA	NA
	AT3G28840	57	-3.54	-4.15	0.75	-4.73	0.00000	0.00010	NA	NA
	AT2G41240	50	-3.55	-4.15	0.75	-4.73	0.00000	0.00010	BHLH100	basic helix-loop-helix protein 100
	AT2G28400	919	-3.56	-3.88	0.60	-5.97	0.00000	0.00000	NA	NA
	AT1G53633	67	-3.56	-4.11	0.73	-4.87	0.00000	0.00006	NA	NA
	AT5G28630	333	-3.56	-3.89	0.60	-5.98	0.00000	0.00000	NA	NA
	AT3G30720	71	-3.56	-4.17	0.75	-4.73	0.00000	0.00010	QQS	QUA-QUINE STARCH
	AT1G18265	97	-3.57	-3.94	0.63	-5.66	0.00000	0.00000	NA	NA
	AT1G61890	1194	-3.57	-3.95	0.63	-5.64	0.00000	0.00000	NA	NA
	AT3G48520	54	-3.57	-4.15	0.74	-4.81	0.00000	0.00007	CYP94B3	cytochrome P450, family 94, subfamily B, polypeptide 3
	AT1G70985	168	-3.58	-4.12	0.72	-4.94	0.00000	0.00004	NA	NA
	AT5G51580	145	-3.58	-3.89	0.58	-6.20	0.00000	0.00000	NA	NA
	AT1G75490	32	-3.59	-4.26	0.77	-4.64	0.00000	0.00014	NA	NA
	AT5G17350	266	-3.59	-3.97	0.63	-5.74	0.00000	0.00000	NA	NA
	AT5G18060	481	-3.61	-3.94	0.59	-6.10	0.00000	0.00000	SAUR23	SMALL AUXIN UP RNA 23
	AT3G15353	93403	-3.61	-4.05	0.66	-5.44	0.00000	0.00000	ATMT3	0
	AT3G43850	42	-3.62	-4.20	0.74	-4.89	0.00000	0.00005	NA	NA
	AT1G77885	258	-3.62	-4.07	0.67	-5.39	0.00000	0.00001	NA	NA
	AT5G20670	675	-3.63	-4.02	0.63	-5.72	0.00000	0.00000	NA	NA
	AT5G05965	11	-3.63	-5.38	0.96	-3.78	0.00016	0.00262	NA	NA
	AT3G25290	54	-3.63	-4.29	0.77	-4.74	0.00000	0.00009	NA	NA
	AT1G75050	17	-3.63	-4.99	0.93	-3.93	0.00009	0.00163	NA	NA
	AT3G22820	70	-3.64	-4.25	0.75	-4.86	0.00000	0.00006	CLL1	CHALLAH-LIKE 1
	AT5G24770	254	-3.64	-4.25	0.75	-4.86	0.00000	0.00006	ATVSP2	0
	AT2G35960	1179	-3.65	-4.01	0.61	-5.94	0.00000	0.00000	NHL12	NDR1/HIN1-like 12
	AT5G39860	618	-3.65	-3.89	0.51	-7.09	0.00000	0.00000	BHLH136	BASIC HELIX-LOOP-HELIX PROTEIN 136
	AT4G33550	31	-3.66	-4.38	0.79	-4.64	0.00000	0.00013	NA	NA
	AT5G45920	27	-3.67	-4.65	0.86	-4.27	0.00002	0.00054	NA	NA
	AT5G50720	208	-3.67	-4.01	0.59	-6.18	0.00000	0.00000	ATHVA22E	ARABIDOPSIS THALIANA HVA22 HOMOLOGUE E
	AT4G32280	358	-3.67	-4.00	0.58	-6.31	0.00000	0.00000	IAA29	indole-3-acetic acid inducible 29
	AT5G05300	772	-3.68	-4.20	0.70	-5.23	0.00000	0.00001	NA	NA
	AT1G22590	419	-3.68	-4.01	0.59	-6.25	0.00000	0.00000	AGL87	AGAMOUS-like 87
	AT5G46295	77	-3.68	-4.43	0.80	-4.61	0.00000	0.00015	NA	NA
	AT1G02820	1282	-3.69	-3.98	0.56	-6.62	0.00000	0.00000	AtLEA3	0
	AT1G52990	26	-3.70	-4.54	0.82	-4.50	0.00001	0.00024	NA	NA
	AT1G67865	40980	-3.70	-4.18	0.68	-5.43	0.00000	0.00000	NA	NA
	AT2G17660	18	-3.70	-5.06	0.92	-4.02	0.00006	0.00122	NA	NA
	AT5G13181	7	-3.70	-8.82	1.04	-3.55	0.00038	0.00507	NA	NA
	AT4G22470	32	-3.71	-4.45	0.79	-4.70	0.00000	0.00011	NA	NA
	AT5G35525	18	-3.73	-4.89	0.87	-4.26	0.00002	0.00055	NA	NA
	AT5G04340	4766	-3.73	-4.10	0.61	-6.08	0.00000	0.00000	C2H2	0
	AT4G28040	28	-3.78	-4.64	0.82	-4.62	0.00000	0.00015	UMAMIT33	Usually multiple acids move in and out Transporters 33

Supplemental Table 1-RNA seq data comparing Pro35S::MYB63 with wild type.

DAP.MYB63.target(1.5kb upstream TSS)	Gene	baseMean	log2FoldChange	lfcMLE	lfcSE	stat	pvalue	padj (FDR)	TAIR10 Symbol	TAIR10 Annotation (Short)
	AT2G02990	274	-3.79	-4.38	0.73	-5.18	0.00000	0.00001	ATRNS1	RIBONUCLEASE 1
	AT5G41761	508	-3.80	-4.07	0.54	-7.08	0.00000	0.00000	NA	NA
	AT4G39403	14	-3.80	-5.64	0.96	-3.97	0.00007	0.00142	PLS	POLARIS
	AT5G57760	157	-3.80	-4.39	0.73	-5.23	0.00000	0.00001	NA	NA
	AT5G16980	24	-3.80	-4.89	0.87	-4.38	0.00001	0.00037	NA	NA
	AT3G49570	252	-3.81	-4.20	0.62	-6.11	0.00000	0.00000	LSU3	RESPONSE TO LOW SULFUR 3
	AT2G34655	1460	-3.81	-4.14	0.57	-6.67	0.00000	0.00000	NA	NA
	AT4G27360	10	-3.82	-6.33	1.00	-3.83	0.00013	0.00224	NA	NA
	AT2G29310	1278	-3.82	-4.09	0.54	-7.10	0.00000	0.00000	NA	NA
	AT5G35490	2146	-3.82	-4.16	0.58	-6.55	0.00000	0.00000	ATMRU1	ARABIDOPSIS MTO 1 RESPONDING UP 1
	AT1G22065	43	-3.83	-4.49	0.75	-5.09	0.00000	0.00002	NA	NA
	AT1G26945	784	-3.83	-4.42	0.73	-5.29	0.00000	0.00001	KDR	KIDARI
	AT2G33775	8	-3.85	-8.92	1.03	-3.72	0.00020	0.00307	RALFL19	ralf-like 19
	AT5G59000	414	-3.85	-4.19	0.59	-6.58	0.00000	0.00000	NA	NA
	AT5G21960	21	-3.86	-5.20	0.90	-4.27	0.00002	0.00054	NA	NA
	AT1G24580	30	-3.86	-4.74	0.82	-4.70	0.00000	0.00011	NA	NA
	AT1G30260	1046	-3.86	-4.26	0.63	-6.17	0.00000	0.00000	NA	NA
	AT5G67080	104	-3.86	-4.43	0.72	-5.37	0.00000	0.00001	MAPKKK19	mitogen-activated protein kinase kinase kinase 19
	AT5G36910	474	-3.86	-4.50	0.75	-5.17	0.00000	0.00002	THI2.2	thionin 2.2
	AT3G47510	366	-3.89	-4.19	0.55	-7.07	0.00000	0.00000	NA	NA
	AT1G29290	223	-3.89	-4.29	0.62	-6.29	0.00000	0.00000	NA	NA
	AT5G12940	1267	-3.89	-4.18	0.54	-7.25	0.00000	0.00000	NA	NA
	AT5G11140	22	-3.90	-4.90	0.84	-4.62	0.00000	0.00015	NA	NA
	AT3G58850	1454	-3.91	-4.41	0.68	-5.72	0.00000	0.00000	HLH2	0
	AT5G60910	145	-3.92	-4.38	0.65	-6.06	0.00000	0.00000	AGL8	AGAMOUS-like 8
	AT3G03341	43	-3.92	-4.60	0.75	-5.23	0.00000	0.00001	NA	NA
	AT1G68240	36	-3.93	-4.73	0.79	-4.98	0.00000	0.00004	NA	NA
	AT1G18400	336	-3.95	-4.27	0.56	-6.99	0.00000	0.00000	BEE1	BR enhanced expression 1
	AT3G56980	97	-3.98	-4.69	0.76	-5.24	0.00000	0.00001	BHLH039	0
	AT5G44260	516	-4.00	-4.32	0.56	-7.11	0.00000	0.00000	AtTZF5	0
	AT1G33760	278	-4.00	-4.73	0.77	-5.19	0.00000	0.00001	NA	NA
	AT2G37430	261	-4.01	-4.41	0.61	-6.54	0.00000	0.00000	ZAT11	zinc finger of Arabidopsis thaliana 11
	AT5G22250	1078	-4.04	-4.42	0.60	-6.72	0.00000	0.00000	AtCAF1b	CCR4- associated factor 1b
	AT5G51390	342	-4.06	-4.37	0.55	-7.39	0.00000	0.00000	NA	NA
	AT4G10290	18	-4.07	-5.64	0.93	-4.39	0.00001	0.00035	NA	NA
YES	AT1G36675	40	-4.08	-4.79	0.75	-5.43	0.00000	0.00000	NA	NA
	AT2G01300	159	-4.09	-4.67	0.70	-5.83	0.00000	0.00000	NA	NA
	AT3G44260	3267	-4.10	-4.59	0.66	-6.22	0.00000	0.00000	AtCAF1a	CCR4- associated factor 1a
	AT1G52830	53	-4.11	-4.77	0.73	-5.62	0.00000	0.00000	IAA6	indole-3-acetic acid 6
	AT1G28370	339	-4.11	-4.78	0.74	-5.56	0.00000	0.00000	ATERF11	ERF DOMAIN PROTEIN 11
	AT1G09950	26	-4.11	-5.12	0.83	-4.94	0.00000	0.00004	RAS1	RESPONSE TO ABA AND SALT 1
	AT3G46900	69	-4.12	-4.66	0.68	-6.01	0.00000	0.00000	COPT2	copper transporter 2
	AT5G55620	2525	-4.12	-4.50	0.59	-6.98	0.00000	0.00000	NA	NA
	AT4G14695	9	-4.13	-9.19	1.02	-4.05	0.00005	0.00113	NA	NA
	AT3G53600	19	-4.13	-5.52	0.89	-4.65	0.00000	0.00013	NA	NA
	AT3G02410	56	-4.13	-4.92	0.78	-5.32	0.00000	0.00001	ICME-LIKE2	Isoprenylcysteine methylesterase-like 2
	AT3G26960	2565	-4.16	-7.16	1.02	-4.06	0.00005	0.00107	NA	NA
	AT1G59930	151	-4.21	-4.76	0.68	-6.17	0.00000	0.00000	NA	NA
	AT3G53250	23	-4.23	-5.75	0.90	-4.70	0.00000	0.00011	SAUR57	SMALL AUXIN UPREGULATED RNA 57
	AT2G32487	642	-4.23	-4.57	0.56	-7.53	0.00000	0.00000	NA	NA
	AT3G60140	36	-4.24	-5.37	0.84	-5.05	0.00000	0.00003	BGLU30	BETA GLUCOSIDASE 30
	AT5G44565	296	-4.24	-4.91	0.73	-5.81	0.00000	0.00000	NA	NA
	AT2G32200	173	-4.25	-5.00	0.76	-5.59	0.00000	0.00000	NA	NA
	AT2G44840	347	-4.26	-4.61	0.56	-7.56	0.00000	0.00000	ATERF13	ETHYLENE-RESPONSIVE ELEMENT BINDING FACTOR 13
	AT4G28085	207	-4.26	-4.60	0.56	-7.65	0.00000	0.00000	NA	NA
	AT1G47370	246	-4.27	-4.82	0.68	-6.30	0.00000	0.00000	NA	NA
	AT1G22110	18	-4.27	-6.08	0.93	-4.61	0.00000	0.00015	NA	NA
	AT5G59310	53	-4.29	-5.33	0.83	-5.16	0.00000	0.00002	LTP4	lipid transfer protein 4
	AT2G44578	66	-4.30	-5.09	0.76	-5.63	0.00000	0.00000	NA	NA
	AT5G55410	27	-4.34	-6.00	0.91	-4.75	0.00000	0.00009	NA	NA
	AT1G05575	1705	-4.36	-4.72	0.57	-7.67	0.00000	0.00000	NA	NA
	AT1G10586	11	-4.36	-9.41	1.01	-4.33	0.00001	0.00044	NA	NA
	AT2G14247	1273	-4.40	-4.89	0.64	-6.82	0.00000	0.00000	NA	NA
	AT2G45570	18	-4.40	-6.21	0.92	-4.77	0.00000	0.00009	CYP76C2	cytochrome P450, family 76, subfamily C, polypeptide 2
	AT1G74930	1769	-4.46	-4.76	0.52	-8.62	0.00000	0.00000	ORA47	0
	AT4G22620	72	-4.48	-5.24	0.74	-6.02	0.00000	0.00000	SAUR34	SMALL AUXIN UPREGULATED RNA 34

Supplemental Table 1-RNA seq data comparing Pro35S::MYB63 with wild type.

DAP.MYB63.target(1.5kb upstream TSS)	Gene	baseMean	log2FoldChange	lfcMLE	lfcSE	stat	pvalue	padj (FDR)	TAIR10 Symbol	TAIR10 Annotation (Short)
	AT1G73325	211	-4.49	-5.21	0.74	-6.11	0.00000	0.00000	NA	NA
	AT3G53980	36	-4.50	-5.47	0.79	-5.68	0.00000	0.00000	NA	NA
	AT1G22810	58	-4.52	-5.25	0.72	-6.26	0.00000	0.00000	NA	NA
	AT3G20470	700	-4.53	-4.86	0.54	-8.39	0.00000	0.00000	ATGRP-5	0
	AT2G37870	34	-4.53	-5.62	0.81	-5.57	0.00000	0.00000	NA	NA
	AT1G04330	23	-4.54	-6.36	0.91	-5.00	0.00000	0.00003	NA	NA
	AT2G42540	2109	-4.56	-5.12	0.67	-6.84	0.00000	0.00000	COR15	0
	AT5G03130	101	-4.56	-5.14	0.68	-6.75	0.00000	0.00000	NA	NA
	AT2G22880	431	-4.60	-5.35	0.74	-6.19	0.00000	0.00000	NA	NA
YES	AT5G02580	52	-4.60	-5.36	0.73	-6.31	0.00000	0.00000	NA	NA
	AT1G72620	20	-4.65	-7.18	0.94	-4.93	0.00000	0.00004	NA	NA
	AT3G11480	42	-4.65	-5.90	0.85	-5.48	0.00000	0.00000	ATBSMT1	0
	AT3G09870	56	-4.68	-5.50	0.74	-6.28	0.00000	0.00000	SAUR48	SMALL AUXIN UPREGULATED RNA 48
	AT5G18010	215	-4.73	-5.33	0.68	-6.99	0.00000	0.00000	SAUR19	small auxin up RNA 19
	AT3G19350	70	-4.73	-5.69	0.78	-6.05	0.00000	0.00000	MPC	maternally expressed pab C-terminal
	AT1G52827	481	-4.75	-5.23	0.61	-7.76	0.00000	0.00000	ATCDT1	0
	AT1G12610	43	-4.76	-5.92	0.81	-5.84	0.00000	0.00000	DDF1	DWARF AND DELAYED FLOWERING 1
	AT1G10585	56	-4.82	-5.62	0.73	-6.59	0.00000	0.00000	NA	NA
	AT1G15580	67	-4.82	-5.57	0.71	-6.76	0.00000	0.00000	ATAUX2-27	0
	AT2G47880	651	-4.90	-5.41	0.62	-7.85	0.00000	0.00000	NA	NA
	AT4G30460	265	-4.96	-5.63	0.69	-7.17	0.00000	0.00000	NA	NA
	AT1G66100	15512	-4.96	-5.36	0.57	-8.76	0.00000	0.00000	NA	NA
	AT1G65480	55	-4.96	-5.82	0.74	-6.68	0.00000	0.00000	FT	FLOWERING LOCUS T
	AT1G11740	90	-5.04	-5.74	0.69	-7.32	0.00000	0.00000	NA	NA
	AT2G18210	347	-5.36	-6.36	0.77	-6.98	0.00000	0.00000	NA	NA
YES	AT4G33790	34	-5.36	-7.03	0.84	-6.37	0.00000	0.00000	CER4	ECERIFERUM 4
	AT1G70270	62	-5.39	-6.56	0.78	-6.88	0.00000	0.00000	NA	NA
	AT4G27654	122	-5.50	-6.60	0.78	-7.06	0.00000	0.00000	NA	NA
	AT4G30450	569	-5.58	-6.38	0.70	-7.99	0.00000	0.00000	NA	NA
	AT4G37290	122	-5.64	-6.65	0.74	-7.58	0.00000	0.00000	NA	NA
	AT1G30135	78	-5.66	-6.84	0.77	-7.38	0.00000	0.00000	JAZ8	jasmonate-zim-domain protein 8
	AT3G29370	331	-5.71	-6.55	0.71	-8.07	0.00000	0.00000	P1R3	P1R3
	AT5G50335	865	-5.80	-6.26	0.55	-10.51	0.00000	0.00000	NA	NA
	AT4G30290	136	-6.03	-6.94	0.70	-8.57	0.00000	0.00000	ATXTH19	XYLOGLUCAN ENDOTRANSGLUCOSYLASE/HYDROLASE 19
	AT2G11520	110	-6.05	-7.06	0.71	-8.48	0.00000	0.00000	CRCK3	calmodulin-binding receptor-like cytoplasmic kinase 3
	AT5G24105	315	-6.24	-7.08	0.68	-9.13	0.00000	0.00000	AGP41	arabinogalactan protein 41
	AT3G28770	92	-6.34	-7.84	0.78	-8.15	0.00000	0.00000	NA	NA
	AT2G37030	45	-6.50	-11.46	0.89	-7.31	0.00000	0.00000	SAUR46	SMALL AUXIN UPREGULATED RNA 46
	AT2G34600	388	-6.57	-7.45	0.68	-9.72	0.00000	0.00000	JAZ7	jasmonate-zim-domain protein 7
	AT3G49580	281	-6.83	-7.73	0.67	-10.17	0.00000	0.00000	LSU1	RESPONSE TO LOW SULFUR 1
	AT5G13170	259	-8.02	-13.97	0.87	-9.20	0.00000	0.00000	AtSWEET15	0

Supplemental Table 2A-GO terms from *Pro35S::MYB63* compared to WT

GO term	Ontology	Description	Number in input list	Number in BG/Ref	p-value	FDR	Notes
GO:0008152	P	metabolic process	529	13633	1.40E-25	7.90E-22	MYB63.up
GO:0044281	P	small molecule metabolic pro	220	4065	5.10E-22	8.70E-19	MYB63.up
GO:0044238	P	primary metabolic process	464	11717	5.90E-22	8.70E-19	MYB63.up
GO:0044237	P	cellular metabolic process	459	11509	3.10E-22	8.70E-19	MYB63.up
GO:0009987	P	cellular process	533	14419	2.70E-20	3.20E-17	MYB63.up
GO:0006807	P	nitrogen compound metabol	262	5675	1.70E-17	1.70E-14	MYB63.up
GO:0006730	P	one-carbon metabolic proces	61	578	2.10E-17	1.70E-14	MYB63.up
GO:0032259	P	methylation	59	548	3.10E-17	2.00E-14	MYB63.up
GO:0043412	P	macromolecule modification	174	3199	3.00E-17	2.00E-14	MYB63.up
GO:0043414	P	macromolecule methylation	58	540	6.30E-17	3.70E-14	MYB63.up
GO:0050896	P	response to stimulus	279	6292	1.50E-16	8.10E-14	MYB63.up
GO:0019438	P	aromatic compound biosynth	62	680	6.00E-15	2.90E-12	MYB63.up
GO:0009791	P	post-embryonic developmen	125	2188	9.10E-14	4.10E-11	MYB63.up
GO:0043687	P	post-translational protein mo	120	2075	1.40E-13	5.90E-11	MYB63.up
GO:0048608	P	reproductive structure devel	105	1722	2.90E-13	1.10E-10	MYB63.up
GO:0032501	P	multicellular organismal proc	190	4020	4.00E-13	1.50E-10	MYB63.up
GO:0000911	P	cytokinesis by cell plate form	31	204	4.30E-13	1.50E-10	MYB63.up
GO:0000910	P	cytokinesis	33	235	5.40E-13	1.70E-10	MYB63.up
GO:0007275	P	multicellular organismal deve	184	3864	5.70E-13	1.80E-10	MYB63.up
GO:0033205	P	cytokinesis during cell cycle	31	208	6.80E-13	2.00E-10	MYB63.up
GO:0006725	P	cellular aromatic compound	74	1022	7.60E-13	2.10E-10	MYB63.up
GO:0050789	P	regulation of biological proce	230	5235	8.30E-13	2.10E-10	MYB63.up
GO:0043170	P	macromolecule metabolic pr	356	9246	8.40E-13	2.10E-10	MYB63.up
GO:0006464	P	protein modification process	144	2773	9.90E-13	2.40E-10	MYB63.up
GO:0034968	P	histone lysine methylation	33	243	1.20E-12	2.90E-10	MYB63.up
GO:0032502	P	developmental process	190	4094	2.00E-12	4.40E-10	MYB63.up
GO:0051301	P	cell division	40	363	2.20E-12	4.90E-10	MYB63.up
GO:0006139	P	nucleobase, nucleoside, nucl	213	4798	3.40E-12	7.10E-10	MYB63.up
GO:0044260	P	cellular macromolecule meta	334	8616	3.90E-12	7.70E-10	MYB63.up
GO:0051239	P	regulation of multicellular or	57	694	4.00E-12	7.80E-10	MYB63.up
GO:0016570	P	histone modification	40	371	4.20E-12	7.90E-10	MYB63.up
GO:0046483	P	heterocycle metabolic proce	72	1023	5.50E-12	9.70E-10	MYB63.up
GO:0009805	P	coumarin biosynthetic proce	17	54	5.40E-12	9.70E-10	MYB63.up
GO:0009804	P	coumarin metabolic process	17	55	6.90E-12	1.20E-09	MYB63.up
GO:0003006	P	reproductive developmental	113	2050	1.50E-11	2.40E-09	MYB63.up
GO:0044249	P	cellular biosynthetic process	304	7737	1.80E-11	3.00E-09	MYB63.up
GO:0016568	P	chromatin modification	43	447	2.00E-11	3.10E-09	MYB63.up
GO:0065007	P	biological regulation	256	6222	2.30E-11	3.60E-09	MYB63.up
GO:0009058	P	biosynthetic process	310	7962	2.90E-11	4.30E-09	MYB63.up
GO:0042221	P	response to chemical stimulu	181	3978	4.00E-11	5.80E-09	MYB63.up
GO:0006950	P	response to stress	184	4089	6.30E-11	9.00E-09	MYB63.up
GO:0009220	P	pyrimidine ribonucleotide bio	23	140	1.20E-10	1.60E-08	MYB63.up
GO:0048856	P	anatomical structure develop	159	3396	1.20E-10	1.60E-08	MYB63.up
GO:0016569	P	covalent chromatin modifica	40	419	1.20E-10	1.70E-08	MYB63.up
GO:0016571	P	histone methylation	33	296	1.50E-10	1.90E-08	MYB63.up
GO:0009908	P	flower development	65	948	1.70E-10	2.20E-08	MYB63.up
GO:0009627	P	systemic acquired resistance	41	445	2.00E-10	2.40E-08	MYB63.up
GO:0006996	P	organelle organization	109	2044	2.20E-10	2.70E-08	MYB63.up
GO:0008283	P	cell proliferation	31	269	2.50E-10	3.00E-08	MYB63.up
GO:0009218	P	pyrimidine ribonucleotide me	23	147	2.70E-10	3.20E-08	MYB63.up
GO:0006479	P	protein amino acid methylati	33	305	2.90E-10	3.30E-08	MYB63.up

Supplemental Table 2A-GO terms from *Pro35S::MYB63* compared to WT

GO:0008213	P	protein amino acid alkylatior	33	305	2.90E-10	3.30E-08	MYB63.up
GO:0022414	P	reproductive process	115	2224	3.80E-10	4.20E-08	MYB63.up
GO:0006221	P	pyrimidine nucleotide biosyn	23	150	3.90E-10	4.20E-08	MYB63.up
GO:0009699	P	phenylpropanoid biosyntheti	34	327	4.10E-10	4.40E-08	MYB63.up
GO:0006220	P	pyrimidine nucleotide metab	23	154	6.10E-10	6.40E-08	MYB63.up
GO:0000003	P	reproduction	116	2272	6.40E-10	6.50E-08	MYB63.up
GO:0006325	P	chromatin organization	46	566	6.90E-10	6.90E-08	MYB63.up
GO:0045087	P	innate immune response	62	930	1.30E-09	1.30E-07	MYB63.up
GO:0050793	P	regulation of developmental	58	844	1.60E-09	1.60E-07	MYB63.up
GO:0002376	P	immune system process	64	984	1.70E-09	1.60E-07	MYB63.up
GO:0006955	P	immune response	64	984	1.70E-09	1.60E-07	MYB63.up
GO:0007017	P	microtubule-based process	32	313	2.00E-09	1.80E-07	MYB63.up
GO:0051567	P	histone H3-K9 methylation	24	180	2.00E-09	1.80E-07	MYB63.up
GO:0007010	P	cytoskeleton organization	42	507	2.30E-09	2.00E-07	MYB63.up
GO:0009962	P	regulation of flavonoid biosy	20	124	2.50E-09	2.20E-07	MYB63.up
GO:0009165	P	nucleotide biosynthetic proc	33	336	2.80E-09	2.40E-07	MYB63.up
GO:0006260	P	DNA replication	33	337	3.00E-09	2.50E-07	MYB63.up
GO:0051276	P	chromosome organization	53	752	3.70E-09	3.20E-07	MYB63.up
GO:0050794	P	regulation of cellular process	193	4595	4.50E-09	3.70E-07	MYB63.up
GO:0007049	P	cell cycle	55	802	4.60E-09	3.70E-07	MYB63.up
GO:0048731	P	system development	106	2083	4.60E-09	3.70E-07	MYB63.up
GO:0048513	P	organ development	106	2083	4.60E-09	3.70E-07	MYB63.up
GO:0009963	P	positive regulation of flavonc	18	103	5.10E-09	4.00E-07	MYB63.up
GO:0022402	P	cell cycle process	45	586	5.40E-09	4.20E-07	MYB63.up
GO:0009698	P	phenylpropanoid metabolic p	36	405	6.00E-09	4.60E-07	MYB63.up
GO:0019222	P	regulation of metabolic proc	145	3186	5.90E-09	4.60E-07	MYB63.up
GO:0006306	P	DNA methylation	23	180	9.00E-09	6.60E-07	MYB63.up
GO:0006305	P	DNA alkylation	23	180	9.00E-09	6.60E-07	MYB63.up
GO:0000226	P	microtubule cytoskeleton org	27	246	9.20E-09	6.70E-07	MYB63.up
GO:0031323	P	regulation of cellular metabc	135	2928	1.10E-08	7.70E-07	MYB63.up
GO:0006304	P	DNA modification	23	183	1.20E-08	8.50E-07	MYB63.up
GO:0042180	P	cellular ketone metabolic prc	106	2123	1.20E-08	8.50E-07	MYB63.up
GO:0009909	P	regulation of flower develop	33	359	1.20E-08	8.50E-07	MYB63.up
GO:0048580	P	regulation of post-embryonic	38	459	1.40E-08	9.20E-07	MYB63.up
GO:0009259	P	ribonucleotide metabolic prc	31	323	1.40E-08	9.20E-07	MYB63.up
GO:0043436	P	oxoacid metabolic process	105	2103	1.40E-08	9.50E-07	MYB63.up
GO:0019752	P	carboxylic acid metabolic prc	105	2103	1.40E-08	9.50E-07	MYB63.up
GO:0006082	P	organic acid metabolic proce	105	2105	1.50E-08	9.80E-07	MYB63.up
GO:0044283	P	small molecule biosynthetic p	96	1865	1.60E-08	1.00E-06	MYB63.up
GO:0043455	P	regulation of secondary meta	21	157	1.90E-08	1.20E-06	MYB63.up
GO:0055086	P	nucleobase, nucleoside and r	50	726	2.10E-08	1.30E-06	MYB63.up
GO:0009813	P	flavonoid biosynthetic proce:	25	225	2.60E-08	1.60E-06	MYB63.up
GO:0009814	P	defense response, incompati	41	536	2.90E-08	1.80E-06	MYB63.up
GO:0051716	P	cellular response to stimulus	113	2355	2.90E-08	1.80E-06	MYB63.up
GO:0006275	P	regulation of DNA replicatior	19	135	4.40E-08	2.70E-06	MYB63.up
GO:0006519	P	cellular amino acid and deriv	73	1324	8.20E-08	4.90E-06	MYB63.up
GO:0031326	P	regulation of cellular biosynt	121	2631	8.20E-08	4.90E-06	MYB63.up
GO:0019538	P	protein metabolic process	207	5220	8.70E-08	5.10E-06	MYB63.up
GO:0009889	P	regulation of biosynthetic pr	121	2634	8.70E-08	5.10E-06	MYB63.up
GO:0010564	P	regulation of cell cycle proce	19	144	1.10E-07	6.40E-06	MYB63.up
GO:0009886	P	post-embryonic morphogene	38	503	1.20E-07	7.10E-06	MYB63.up
GO:0009117	P	nucleotide metabolic proces:	46	685	1.50E-07	8.70E-06	MYB63.up
GO:0006753	P	nucleoside phosphate metab	46	687	1.70E-07	9.30E-06	MYB63.up
GO:0009812	P	flavonoid metabolic process	25	251	1.80E-07	1.00E-05	MYB63.up
GO:0016070	P	RNA metabolic process	157	3734	1.90E-07	1.00E-05	MYB63.up

Supplemental Table 2A-GO terms from *Pro35S::MYB63* compared to WT

GO:0046686	P	response to cadmium ion	36	470	1.90E-07	1.10E-05	MYB63.up
GO:0009887	P	organ morphogenesis	43	629	2.50E-07	1.30E-05	MYB63.up
GO:0051726	P	regulation of cell cycle	27	293	2.50E-07	1.30E-05	MYB63.up
GO:0010038	P	response to metal ion	42	610	2.90E-07	1.50E-05	MYB63.up
GO:0009696	P	salicylic acid metabolic proce	23	222	2.90E-07	1.50E-05	MYB63.up
GO:0044267	P	cellular protein metabolic pr	190	4775	2.90E-07	1.50E-05	MYB63.up
GO:0009260	P	ribonucleotide biosynthetic p	23	229	4.80E-07	2.50E-05	MYB63.up
GO:0042398	P	cellular amino acid derivative	38	534	5.00E-07	2.50E-05	MYB63.up
GO:0048519	P	negative regulation of biolog	67	1243	6.20E-07	3.10E-05	MYB63.up
GO:0051052	P	regulation of DNA metabolic	21	198	6.70E-07	3.40E-05	MYB63.up
GO:0009451	P	RNA modification	32	418	9.30E-07	4.60E-05	MYB63.up
GO:0006605	P	protein targeting	53	906	1.00E-06	5.00E-05	MYB63.up
GO:0051707	P	response to other organism	73	1421	1.00E-06	5.10E-05	MYB63.up
GO:0040029	P	regulation of gene expressio	36	508	1.10E-06	5.30E-05	MYB63.up
GO:0016043	P	cellular component organiza	133	3141	1.40E-06	6.80E-05	MYB63.up
GO:0009697	P	salicylic acid biosynthetic pro	21	209	1.50E-06	7.10E-05	MYB63.up
GO:0080090	P	regulation of primary metab	120	2761	1.50E-06	7.20E-05	MYB63.up
GO:0048507	P	meristem development	35	498	1.80E-06	8.60E-05	MYB63.up
GO:0010389	P	regulation of G2/M transitio	12	69	1.90E-06	8.90E-05	MYB63.up
GO:0051225	P	spindle assembly	10	45	2.00E-06	9.40E-05	MYB63.up
GO:0046907	P	intracellular transport	72	1423	2.10E-06	9.50E-05	MYB63.up
GO:0006468	P	protein amino acid phosphor	61	1134	2.10E-06	9.70E-05	MYB63.up
GO:0000086	P	G2/M transition of mitotic ce	12	70	2.20E-06	9.90E-05	MYB63.up
GO:0034613	P	cellular protein localization	58	1059	2.30E-06	0.0001	MYB63.up
GO:0010016	P	shoot morphogenesis	36	529	2.60E-06	0.00012	MYB63.up
GO:0006259	P	DNA metabolic process	47	790	2.70E-06	0.00012	MYB63.up
GO:0006886	P	intracellular protein transpor	57	1044	3.00E-06	0.00013	MYB63.up
GO:0051329	P	interphase of mitotic cell cyc	12	73	3.20E-06	0.00014	MYB63.up
GO:0048367	P	shoot development	44	723	3.30E-06	0.00014	MYB63.up
GO:0022621	P	shoot system development	44	723	3.30E-06	0.00014	MYB63.up
GO:0007051	P	spindle organization	10	48	3.40E-06	0.00014	MYB63.up
GO:0048518	P	positive regulation of biologi	51	896	3.40E-06	0.00014	MYB63.up
GO:0001510	P	RNA methylation	19	185	3.50E-06	0.00015	MYB63.up
GO:0009751	P	response to salicylic acid stin	33	470	3.60E-06	0.00015	MYB63.up
GO:0051325	P	interphase	12	74	3.70E-06	0.00015	MYB63.up
GO:0000278	P	mitotic cell cycle	25	302	4.00E-06	0.00017	MYB63.up
GO:0007389	P	pattern specification process	27	345	4.50E-06	0.00018	MYB63.up
GO:0070727	P	cellular macromolecule local	58	1093	5.60E-06	0.00023	MYB63.up
GO:0034976	P	response to endoplasmic ret	27	352	6.30E-06	0.00025	MYB63.up
GO:0031347	P	regulation of defense respon	35	529	6.30E-06	0.00025	MYB63.up
GO:0009891	P	positive regulation of biosyn	38	600	6.60E-06	0.00026	MYB63.up
GO:0031328	P	positive regulation of cellula	38	600	6.60E-06	0.00026	MYB63.up
GO:0006270	P	DNA replication initiation	11	66	7.30E-06	0.00029	MYB63.up
GO:0035266	P	meristem growth	18	179	8.20E-06	0.00032	MYB63.up
GO:0006261	P	DNA-dependent DNA replica	22	256	8.80E-06	0.00034	MYB63.up
GO:0006952	P	defense response	78	1653	8.90E-06	0.00034	MYB63.up
GO:0009628	P	response to abiotic stimulus	112	2635	9.40E-06	0.00036	MYB63.up
GO:0016310	P	phosphorylation	67	1354	9.50E-06	0.00036	MYB63.up
GO:0009607	P	response to biotic stimulus	79	1687	1.00E-05	0.00038	MYB63.up
GO:0016458	P	gene silencing	31	450	1.00E-05	0.00038	MYB63.up
GO:0080134	P	regulation of response to str	35	544	1.10E-05	0.00041	MYB63.up
GO:0031325	P	positive regulation of cellula	38	615	1.10E-05	0.00041	MYB63.up
GO:0051649	P	establishment of localization	74	1554	1.10E-05	0.00042	MYB63.up
GO:0006575	P	cellular amino acid derivative	42	714	1.20E-05	0.00044	MYB63.up
GO:0048583	P	regulation of response to stir	40	667	1.30E-05	0.00046	MYB63.up

Supplemental Table 2A-GO terms from *Pro35S::MYB63* compared to WT

GO:0070925	P	organelle assembly	10	57	1.30E-05	0.00046	MYB63.up
GO:0009893	P	positive regulation of metabo	38	623	1.50E-05	0.00052	MYB63.up
GO:0009863	P	salicylic acid mediated signal	26	349	1.50E-05	0.00053	MYB63.up
GO:0006558	P	L-phenylalanine metabolic pr	6	15	1.50E-05	0.00054	MYB63.up
GO:0071446	P	cellular response to salicylic a	26	351	1.60E-05	0.00057	MYB63.up
GO:0010075	P	regulation of meristem grow	17	171	1.70E-05	0.00058	MYB63.up
GO:0048645	P	organ formation	18	192	2.00E-05	0.00068	MYB63.up
GO:0009862	P	systemic acquired resistance	21	251	2.00E-05	0.0007	MYB63.up
GO:0048449	P	floral organ formation	17	174	2.00E-05	0.0007	MYB63.up
GO:0033554	P	cellular response to stress	70	1473	2.10E-05	0.00072	MYB63.up
GO:0051641	P	cellular localization	75	1613	2.10E-05	0.00073	MYB63.up
GO:0009888	P	tissue development	53	1015	2.20E-05	0.00073	MYB63.up
GO:0016572	P	histone phosphorylation	10	61	2.20E-05	0.00073	MYB63.up
GO:0010629	P	negative regulation of gene e	37	611	2.20E-05	0.00073	MYB63.up
GO:0010605	P	negative regulation of macro	38	636	2.20E-05	0.00075	MYB63.up
GO:0048827	P	phyllome development	32	497	2.60E-05	0.00085	MYB63.up
GO:0048366	P	leaf development	30	451	2.60E-05	0.00085	MYB63.up
GO:0009611	P	response to wounding	25	340	2.60E-05	0.00086	MYB63.up
GO:0006796	P	phosphate metabolic process	73	1571	2.80E-05	0.00091	MYB63.up
GO:0006793	P	phosphorus metabolic proce	73	1572	2.90E-05	0.00093	MYB63.up
GO:0008104	P	protein localization	60	1216	3.00E-05	0.00097	MYB63.up
GO:0045934	P	negative regulation of nucleo	28	413	3.50E-05	0.0011	MYB63.up
GO:0046394	P	carboxylic acid biosynthetic p	56	1116	3.60E-05	0.0011	MYB63.up
GO:0016053	P	organic acid biosynthetic pro	56	1116	3.60E-05	0.0011	MYB63.up
GO:0010033	P	response to organic substanc	113	2754	3.70E-05	0.0012	MYB63.up
GO:0006612	P	protein targeting to membra	27	392	3.70E-05	0.0012	MYB63.up
GO:0006342	P	chromatin silencing	20	242	3.70E-05	0.0012	MYB63.up
GO:0045088	P	regulation of innate immune	28	415	3.80E-05	0.0012	MYB63.up
GO:0051172	P	negative regulation of nitrog	28	415	3.80E-05	0.0012	MYB63.up
GO:0048509	P	regulation of meristem devel	19	223	3.90E-05	0.0012	MYB63.up
GO:0048437	P	floral organ development	36	605	4.00E-05	0.0012	MYB63.up
GO:0065001	P	specification of axis polarity	11	81	4.10E-05	0.0012	MYB63.up
GO:0010558	P	negative regulation of macro	28	418	4.30E-05	0.0013	MYB63.up
GO:0048569	P	post-embryonic organ develo	36	608	4.40E-05	0.0013	MYB63.up
GO:0009072	P	aromatic amino acid family m	20	245	4.40E-05	0.0013	MYB63.up
GO:0002682	P	regulation of immune system	28	419	4.40E-05	0.0013	MYB63.up
GO:0050776	P	regulation of immune respon	28	419	4.40E-05	0.0013	MYB63.up
GO:0022403	P	cell cycle phase	25	352	4.50E-05	0.0013	MYB63.up
GO:0009892	P	negative regulation of metab	38	659	4.60E-05	0.0013	MYB63.up
GO:0010310	P	regulation of hydrogen perox	17	187	4.70E-05	0.0014	MYB63.up
GO:0045814	P	negative regulation of gene e	20	247	4.80E-05	0.0014	MYB63.up
GO:0080010	P	regulation of oxygen and rea	17	188	5.00E-05	0.0014	MYB63.up
GO:0045184	P	establishment of protein loca	57	1160	5.30E-05	0.0015	MYB63.up
GO:0009626	P	plant-type hypersensitive res	27	401	5.40E-05	0.0015	MYB63.up
GO:0015031	P	protein transport	57	1160	5.30E-05	0.0015	MYB63.up
GO:0009890	P	negative regulation of biosyn	28	425	5.60E-05	0.0016	MYB63.up
GO:0034050	P	host programmed cell death	27	402	5.60E-05	0.0016	MYB63.up
GO:0009073	P	aromatic amino acid family b	12	100	5.50E-05	0.0016	MYB63.up
GO:0031327	P	negative regulation of cellula	28	425	5.60E-05	0.0016	MYB63.up
GO:0048522	P	positive regulation of cellular	43	793	5.80E-05	0.0016	MYB63.up
GO:0019748	P	secondary metabolic process	60	1247	6.00E-05	0.0016	MYB63.up
GO:0046417	P	chorismate metabolic proces	12	101	6.10E-05	0.0017	MYB63.up
GO:0012501	P	programmed cell death	29	451	6.20E-05	0.0017	MYB63.up
GO:0010035	P	response to inorganic substa	54	1086	6.30E-05	0.0017	MYB63.up
GO:0070887	P	cellular response to chemica	66	1417	6.50E-05	0.0017	MYB63.up

Supplemental Table 2A-GO terms from *Pro35S::MYB63* compared to WT

GO:0008219	P	cell death	31	500	6.70E-05	0.0018	MYB63.up
GO:0016265	P	death	31	500	6.70E-05	0.0018	MYB63.up
GO:0023052	P	signaling	99	2376	6.90E-05	0.0018	MYB63.up
GO:0045010	P	actin nucleation	12	103	7.20E-05	0.0019	MYB63.up
GO:0033036	P	macromolecule localization	70	1541	8.00E-05	0.0021	MYB63.up
GO:0009056	P	catabolic process	92	2182	8.50E-05	0.0022	MYB63.up
GO:0006800	P	oxygen and reactive oxygen s	24	347	9.30E-05	0.0024	MYB63.up
GO:0010363	P	regulation of plant-type hype	25	371	9.90E-05	0.0026	MYB63.up
GO:0048523	P	negative regulation of cellula	42	789	0.0001	0.0027	MYB63.up
GO:0003002	P	regionalization	19	241	0.0001	0.0027	MYB63.up
GO:0051258	P	protein polymerization	9	60	0.0001	0.0027	MYB63.up
GO:0043067	P	regulation of programmed ce	26	397	0.00011	0.0029	MYB63.up
GO:0010073	P	meristem maintenance	21	286	0.00012	0.0029	MYB63.up
GO:0051704	P	multi-organism process	79	1820	0.00012	0.0029	MYB63.up
GO:0009798	P	axis specification	11	92	0.00012	0.0029	MYB63.up
GO:0007346	P	regulation of mitotic cell cycl	12	109	0.00012	0.003	MYB63.up
GO:0009308	P	amine metabolic process	51	1034	0.00012	0.003	MYB63.up
GO:0045892	P	negative regulation of transc	26	400	0.00012	0.0031	MYB63.up
GO:0016481	P	negative regulation of transc	26	400	0.00012	0.0031	MYB63.up
GO:0051253	P	negative regulation of RNA m	26	400	0.00012	0.0031	MYB63.up
GO:0031324	P	negative regulation of cellula	28	449	0.00013	0.0033	MYB63.up
GO:0080135	P	regulation of cellular respons	25	379	0.00014	0.0033	MYB63.up
GO:0044248	P	cellular catabolic process	88	2095	0.00014	0.0034	MYB63.up
GO:0042743	P	hydrogen peroxide metabolic	23	335	0.00014	0.0035	MYB63.up
GO:0051171	P	regulation of nitrogen compo	102	2517	0.00014	0.0035	MYB63.up
GO:0006520	P	cellular amino acid metabolic	45	882	0.00015	0.0036	MYB63.up
GO:0005975	P	carbohydrate metabolic proc	93	2249	0.00015	0.0036	MYB63.up
GO:0010941	P	regulation of cell death	26	405	0.00015	0.0036	MYB63.up
GO:0009944	P	polarity specification of adax	10	79	0.00015	0.0037	MYB63.up
GO:0006457	P	protein folding	23	339	0.00017	0.004	MYB63.up
GO:0009943	P	adaxial/abaxial axis specifica	10	81	0.00018	0.0044	MYB63.up
GO:0044106	P	cellular amine metabolic pro	47	947	0.00019	0.0046	MYB63.up
GO:0006598	P	polyamine catabolic process	7	38	0.0002	0.0046	MYB63.up
GO:0051169	P	nuclear transport	17	213	0.0002	0.0047	MYB63.up
GO:0006913	P	nucleocytoplasmic transport	17	213	0.0002	0.0047	MYB63.up
GO:0032787	P	monocarboxylic acid metabo	66	1481	0.00022	0.005	MYB63.up
GO:0009965	P	leaf morphogenesis	23	347	0.00023	0.0053	MYB63.up
GO:0031048	P	chromatin silencing by small	12	118	0.00023	0.0053	MYB63.up
GO:0071310	P	cellular response to organic s	57	1234	0.00025	0.0057	MYB63.up
GO:0006096	P	glycolysis	17	219	0.00028	0.0063	MYB63.up
GO:0007166	P	cell surface receptor linked s	14	160	0.00031	0.007	MYB63.up
GO:0010468	P	regulation of gene expression	106	2695	0.00031	0.007	MYB63.up
GO:0009955	P	adaxial/abaxial pattern form	10	87	0.00031	0.007	MYB63.up
GO:0023060	P	signal transmission	75	1767	0.00033	0.0074	MYB63.up
GO:0023046	P	signaling process	75	1768	0.00033	0.0075	MYB63.up
GO:0008652	P	cellular amino acid biosynthe	29	503	0.00036	0.0078	MYB63.up
GO:0010556	P	regulation of macromolecule	99	2491	0.00035	0.0078	MYB63.up
GO:0043648	P	dicarboxylic acid metabolic p	13	143	0.00036	0.0078	MYB63.up
GO:0060255	P	regulation of macromolecule	110	2829	0.00035	0.0078	MYB63.up
GO:0019219	P	regulation of nucleobase, nu	99	2496	0.00038	0.0083	MYB63.up
GO:0070646	P	protein modification by smal	15	184	0.00038	0.0084	MYB63.up
GO:0009808	P	lignin metabolic process	9	74	0.00042	0.0092	MYB63.up
GO:0048438	P	floral whorl development	29	510	0.00044	0.0095	MYB63.up
GO:0010154	P	fruit development	37	721	0.0005	0.011	MYB63.up
GO:0010103	P	stomatal complex morphoge	13	149	0.00051	0.011	MYB63.up

Supplemental Table 2A-GO terms from *Pro35S::MYB63* compared to WT

GO:0048316	P	seed development	36	696	0.00052	0.011	MYB63.up
GO:0048563	P	post-embryonic organ morph	17	233	0.00054	0.011	MYB63.up
GO:0048444	P	floral organ morphogenesis	17	233	0.00054	0.011	MYB63.up
GO:0044271	P	cellular nitrogen compound l	40	804	0.00054	0.012	MYB63.up
GO:0009653	P	anatomical structure morpho	74	1783	0.00066	0.014	MYB63.up
GO:0034641	P	cellular nitrogen compound r	60	1373	0.00066	0.014	MYB63.up
GO:0009651	P	response to salt stress	39	788	0.0007	0.015	MYB63.up
GO:0006163	P	purine nucleotide metabolic	16	217	0.0007	0.015	MYB63.up
GO:0009309	P	amine biosynthetic process	29	527	0.00072	0.015	MYB63.up
GO:0007165	P	signal transduction	70	1670	0.00072	0.015	MYB63.up
GO:0006006	P	glucose metabolic process	30	554	0.00075	0.015	MYB63.up
GO:0006346	P	methylation-dependent chro	11	117	0.00077	0.016	MYB63.up
GO:0001666	P	response to hypoxia	10	100	0.00085	0.017	MYB63.up
GO:0051170	P	nuclear import	13	158	0.00085	0.017	MYB63.up
GO:0007015	P	actin filament organization	13	158	0.00085	0.017	MYB63.up
GO:0009790	P	embryonic development	33	638	0.00088	0.018	MYB63.up
GO:0007243	P	protein kinase cascade	16	223	0.00091	0.018	MYB63.up
GO:0006396	P	RNA processing	45	967	0.00095	0.019	MYB63.up
GO:0007242	P	intracellular signaling cascad	55	1252	0.00098	0.02	MYB63.up
GO:0009855	P	determination of bilateral sy	11	122	0.0011	0.021	MYB63.up
GO:0048439	P	flower morphogenesis	8	68	0.0011	0.021	MYB63.up
GO:0033365	P	protein localization in organe	18	272	0.0011	0.021	MYB63.up
GO:0031348	P	negative regulation of defens	18	273	0.0011	0.022	MYB63.up
GO:0009799	P	specification of symmetry	11	123	0.0011	0.022	MYB63.up
GO:0070482	P	response to oxygen levels	10	104	0.0011	0.022	MYB63.up
GO:0009314	P	response to radiation	55	1263	0.0012	0.023	MYB63.up
GO:0006970	P	response to osmotic stress	40	842	0.0012	0.024	MYB63.up
GO:0000165	P	MAPKKK cascade	15	209	0.0013	0.025	MYB63.up
GO:0048585	P	negative regulation of respor	21	349	0.0013	0.026	MYB63.up
GO:0044282	P	small molecule catabolic pro	43	932	0.0014	0.028	MYB63.up
GO:0009416	P	response to light stimulus	52	1188	0.0014	0.028	MYB63.up
GO:0009266	P	response to temperature stir	44	962	0.0015	0.028	MYB63.up
GO:0014070	P	response to organic cyclic su	12	148	0.0015	0.028	MYB63.up
GO:0048453	P	sepal formation	8	72	0.0015	0.028	MYB63.up
GO:0010228	P	vegetative to reproductive pl	25	451	0.0015	0.028	MYB63.up
GO:0048447	P	sepal morphogenesis	8	72	0.0015	0.028	MYB63.up
GO:0010583	P	response to cyclopentenone	12	148	0.0015	0.028	MYB63.up
GO:0006094	P	gluconeogenesis	13	169	0.0015	0.028	MYB63.up
GO:0046364	P	monosaccharide biosynthetic	14	191	0.0015	0.029	MYB63.up
GO:0048451	P	petal formation	8	73	0.0016	0.03	MYB63.up
GO:0016071	P	mRNA metabolic process	22	381	0.0017	0.032	MYB63.up
GO:0009605	P	response to external stimulu	48	1087	0.0018	0.034	MYB63.up
GO:0009793	P	embryonic development end	29	563	0.0019	0.034	MYB63.up
GO:0006606	P	protein import into nucleus	12	156	0.0022	0.042	MYB63.up
GO:0006595	P	polyamine metabolic process	7	60	0.0023	0.042	MYB63.up
GO:0048638	P	regulation of developmental	17	269	0.0023	0.043	MYB63.up
GO:0048464	P	flower calyx development	8	78	0.0024	0.043	MYB63.up
GO:0048442	P	sepal development	8	78	0.0024	0.043	MYB63.up
GO:0001708	P	cell fate specification	7	61	0.0025	0.045	MYB63.up
GO:0017038	P	protein import	21	368	0.0025	0.045	MYB63.up
GO:0019318	P	hexose metabolic process	30	602	0.0025	0.045	MYB63.up
GO:0009753	P	response to jasmonic acid sti	25	471	0.0026	0.046	MYB63.up
GO:0034504	P	protein localization in nucleu	12	159	0.0026	0.047	MYB63.up
GO:0009617	P	response to bacterium	29	577	0.0026	0.047	MYB63.up
GO:0042127	P	regulation of cell proliferatio	10	118	0.0027	0.048	MYB63.up

Supplemental Table 2A-GO terms from *Pro35S::MYB63* compared to WT

GO:0043900	P	regulation of multi-organism	10	118	0.0027	0.048	MYB63.up
GO:0031047	P	gene silencing by RNA	21	371	0.0027	0.048	MYB63.up
GO:0019319	P	hexose biosynthetic process	13	182	0.0028	0.049	MYB63.up
GO:0003824	F	catalytic activity	382	8787	2.00E-23	1.60E-20	MYB63.up
GO:0000166	F	nucleotide binding	175	3438	9.30E-15	3.70E-12	MYB63.up
GO:0032555	F	purine ribonucleotide bindin	130	2429	2.30E-12	3.60E-10	MYB63.up
GO:0032553	F	ribonucleotide binding	130	2429	2.30E-12	3.60E-10	MYB63.up
GO:0005524	F	ATP binding	114	1996	1.40E-12	3.60E-10	MYB63.up
GO:0005488	F	binding	412	11247	3.50E-12	4.00E-10	MYB63.up
GO:0016740	F	transferase activity	151	3012	3.30E-12	4.00E-10	MYB63.up
GO:0017076	F	purine nucleotide binding	135	2595	5.30E-12	5.40E-10	MYB63.up
GO:0032559	F	adenyl ribonucleotide bindin	118	2169	1.10E-11	9.50E-10	MYB63.up
GO:0001882	F	nucleoside binding	124	2341	1.50E-11	1.20E-09	MYB63.up
GO:0030554	F	adenyl nucleotide binding	123	2331	2.40E-11	1.70E-09	MYB63.up
GO:0001883	F	purine nucleoside binding	123	2333	2.50E-11	1.70E-09	MYB63.up
GO:0016787	F	hydrolase activity	134	3099	4.30E-07	2.60E-05	MYB63.up
GO:0016817	F	hydrolase activity, acting on	53	880	4.50E-07	2.60E-05	MYB63.up
GO:0016818	F	hydrolase activity, acting on	52	866	6.20E-07	3.30E-05	MYB63.up
GO:0016772	F	transferase activity, transfer	80	1590	6.70E-07	3.40E-05	MYB63.up
GO:0017111	F	nucleoside-triphosphatase a	50	826	8.30E-07	3.90E-05	MYB63.up
GO:0016462	F	pyrophosphatase activity	51	854	9.30E-07	4.20E-05	MYB63.up
GO:0004674	F	protein serine/threonine kin	53	940	2.80E-06	0.00012	MYB63.up
GO:0016773	F	phosphotransferase activity,	64	1275	9.90E-06	0.00038	MYB63.up
GO:0016301	F	kinase activity	67	1356	9.90E-06	0.00038	MYB63.up
GO:0016887	F	ATPase activity	32	488	1.80E-05	0.00068	MYB63.up
GO:0051082	F	unfolded protein binding	13	117	5.70E-05	0.002	MYB63.up
GO:0004672	F	protein kinase activity	55	1113	6.30E-05	0.0021	MYB63.up
GO:0005515	F	protein binding	109	2709	0.00011	0.0035	MYB63.up
GO:0016853	F	isomerase activity	18	237	0.00024	0.0074	MYB63.up
GO:0004553	F	hydrolase activity, hydrolyzir	26	423	0.00028	0.0085	MYB63.up
GO:0016864	F	intramolecular oxidoreducta	5	19	0.00041	0.011	MYB63.up
GO:0016862	F	intramolecular oxidoreducta	5	19	0.00041	0.011	MYB63.up
GO:0003756	F	protein disulfide isomerase a	5	19	0.00041	0.011	MYB63.up
GO:0016798	F	hydrolase activity, acting on	26	448	0.00064	0.017	MYB63.up
GO:0008026	F	ATP-dependent helicase acti	11	119	0.00087	0.021	MYB63.up
GO:0042623	F	ATPase activity, coupled	22	360	0.00087	0.021	MYB63.up
GO:0070035	F	purine NTP-dependent helica	11	119	0.00087	0.021	MYB63.up
GO:0003887	F	DNA-directed DNA polymera	5	24	0.001	0.023	MYB63.up
GO:0016840	F	carbon-nitrogen lyase activit	5	25	0.0012	0.027	MYB63.up
GO:0004386	F	helicase activity	13	167	0.0014	0.03	MYB63.up
GO:0044464	C	cell part	715	22455	2.50E-16	6.30E-14	MYB63.up
GO:0005623	C	cell	715	22455	2.50E-16	6.30E-14	MYB63.up
GO:0005886	C	plasma membrane	161	3740	2.80E-08	4.70E-06	MYB63.up
GO:0005829	C	cytosol	86	1644	5.00E-08	6.30E-06	MYB63.up
GO:0005737	C	cytoplasm	417	12233	8.40E-08	8.50E-06	MYB63.up
GO:0005622	C	intracellular	626	20042	1.20E-07	1.00E-05	MYB63.up
GO:0044424	C	intracellular part	623	19957	1.80E-07	1.30E-05	MYB63.up
GO:0044459	C	plasma membrane part	61	1065	3.00E-07	1.90E-05	MYB63.up
GO:0043228	C	non-membrane-bounded org	61	1109	1.10E-06	5.50E-05	MYB63.up
GO:0043232	C	intracellular non-membrane-	61	1109	1.10E-06	5.50E-05	MYB63.up
GO:0005911	C	cell-cell junction	50	854	2.00E-06	9.30E-05	MYB63.up
GO:0030054	C	cell junction	50	857	2.20E-06	9.40E-05	MYB63.up
GO:0044444	C	cytoplasmic part	342	9995	3.70E-06	0.00014	MYB63.up
GO:0030312	C	external encapsulating struct	39	621	6.00E-06	0.00022	MYB63.up
GO:0055044	C	symplast	48	852	8.50E-06	0.00024	MYB63.up

Supplemental Table 2A-GO terms from *Pro35S::MYB63* compared to WT

GO:0044428	C	nuclear part	40	650	7.20E-06	0.00024	MYB63.up
GO:0044446	C	intracellular organelle part	137	3379	8.50E-06	0.00024	MYB63.up
GO:0009506	C	plasmodesma	48	852	8.50E-06	0.00024	MYB63.up
GO:0044422	C	organelle part	137	3385	9.30E-06	0.00025	MYB63.up
GO:0005618	C	cell wall	38	618	1.20E-05	0.00031	MYB63.up
GO:0016020	C	membrane	222	6122	1.60E-05	0.0004	MYB63.up
GO:0005730	C	nucleolus	23	303	3.50E-05	0.0008	MYB63.up
GO:0015630	C	microtubule cytoskeleton	13	122	8.40E-05	0.0019	MYB63.up
GO:0009570	C	chloroplast stroma	34	603	0.00017	0.0036	MYB63.up
GO:0009532	C	plastid stroma	35	637	0.00022	0.0044	MYB63.up
GO:0031981	C	nuclear lumen	28	467	0.00025	0.0048	MYB63.up
GO:0044430	C	cytoskeletal part	14	161	0.00033	0.0061	MYB63.up
GO:0005874	C	microtubule	8	60	0.00051	0.0092	MYB63.up
GO:0009295	C	nucleoid	7	47	0.00063	0.011	MYB63.up
GO:0009524	C	phragmoplast	7	49	0.00078	0.013	MYB63.up
GO:0009536	C	plastid	146	4037	0.00079	0.013	MYB63.up
GO:0005856	C	cytoskeleton	15	199	0.00082	0.013	MYB63.up
GO:0005773	C	vacuole	41	855	0.00092	0.014	MYB63.up
GO:0009507	C	chloroplast	143	3959	0.00095	0.014	MYB63.up
GO:0070013	C	intracellular organelle lumen	32	622	0.0011	0.016	MYB63.up
GO:0043233	C	organelle lumen	32	622	0.0011	0.016	MYB63.up
GO:0031974	C	membrane-enclosed lumen	32	628	0.0013	0.018	MYB63.up
GO:0042646	C	plastid nucleoid	5	28	0.0019	0.024	MYB63.up
GO:0044454	C	nuclear chromosome part	6	42	0.0018	0.024	MYB63.up
GO:0043231	C	intracellular membrane-bour	525	17343	0.0021	0.027	MYB63.up
GO:0043227	C	membrane-bounded organel	525	17349	0.0022	0.027	MYB63.up
GO:0043229	C	intracellular organelle	530	17570	0.0027	0.033	MYB63.up
GO:0043226	C	organelle	530	17578	0.0029	0.034	MYB63.up
GO:0044434	C	chloroplast part	51	1211	0.0034	0.039	MYB63.up
GO:0044435	C	plastid part	52	1252	0.004	0.045	MYB63.up
GO:0006661	P	phosphatidylinositol biosyntl	65	98	1.40E-32	9.30E-29	MYB63.down
GO:0046488	P	phosphatidylinositol metabo	65	112	4.20E-30	9.70E-27	MYB63.down
GO:0046489	P	phosphoinositide biosynthet	65	112	4.20E-30	9.70E-27	MYB63.down
GO:0009719	P	response to endogenous stin	253	1615	8.70E-25	1.50E-21	MYB63.down
GO:0010033	P	response to organic substanc	367	2754	3.50E-24	4.50E-21	MYB63.down
GO:0030384	P	phosphoinositide metabolic	66	159	4.00E-24	4.50E-21	MYB63.down
GO:0046474	P	glycerophospholipid biosyntl	69	183	3.00E-23	2.60E-20	MYB63.down
GO:0045017	P	glycerolipid biosynthetic pro	72	200	3.00E-23	2.60E-20	MYB63.down
GO:0009725	P	response to hormone stimulu	213	1375	2.10E-20	1.60E-17	MYB63.down
GO:0042221	P	response to chemical stimulu	460	3978	3.40E-19	2.30E-16	MYB63.down
GO:0006650	P	glycerophospholipid metabo	70	240	1.70E-18	1.10E-15	MYB63.down
GO:0046486	P	glycerolipid metabolic proces	73	262	2.60E-18	1.50E-15	MYB63.down
GO:0008654	P	phospholipid biosynthetic pr	90	405	7.40E-17	3.90E-14	MYB63.down
GO:0010200	P	response to chitin	86	421	2.40E-14	1.20E-11	MYB63.down
GO:0006644	P	phospholipid metabolic proc	91	472	7.90E-14	3.60E-11	MYB63.down
GO:0019637	P	organophosphate metabolic	92	483	1.10E-13	4.30E-11	MYB63.down
GO:0008610	P	lipid biosynthetic process	167	1159	1.10E-13	4.30E-11	MYB63.down
GO:0050896	P	response to stimulus	628	6292	1.40E-13	5.30E-11	MYB63.down
GO:0044255	P	cellular lipid metabolic proce	190	1395	2.10E-13	7.60E-11	MYB63.down
GO:0009611	P	response to wounding	68	340	2.80E-11	9.50E-09	MYB63.down
GO:0009743	P	response to carbohydrate sti	120	812	9.00E-11	2.90E-08	MYB63.down
GO:0006629	P	lipid metabolic process	212	1772	4.20E-10	1.30E-07	MYB63.down
GO:0009753	P	response to jasmonic acid sti	78	471	2.50E-09	7.40E-07	MYB63.down
GO:0009692	P	ethylene metabolic process	35	131	4.00E-09	1.10E-06	MYB63.down
GO:0009693	P	ethylene biosynthetic proces	35	131	4.00E-09	1.10E-06	MYB63.down

Supplemental Table 2A-GO terms from *Pro35S::MYB63* compared to WT

GO:0009414	P	response to water deprivatio	71	416	4.30E-09	1.10E-06	MYB63.down
GO:0043449	P	cellular alkene metabolic pro	35	133	5.60E-09	1.40E-06	MYB63.down
GO:0043450	P	alkene biosynthetic process	35	133	5.60E-09	1.40E-06	MYB63.down
GO:0009733	P	response to auxin stimulus	72	431	7.40E-09	1.70E-06	MYB63.down
GO:0009415	P	response to water	71	424	8.60E-09	2.00E-06	MYB63.down
GO:0000160	P	two-component signal transc	38	165	2.70E-08	6.10E-06	MYB63.down
GO:0015979	P	photosynthesis	70	435	4.40E-08	9.40E-06	MYB63.down
GO:0006950	P	response to stress	404	4089	4.50E-08	9.40E-06	MYB63.down
GO:0009694	P	jasmonic acid metabolic proc	36	160	1.00E-07	2.10E-05	MYB63.down
GO:0031407	P	oxylipin metabolic process	37	168	1.10E-07	2.20E-05	MYB63.down
GO:0019684	P	photosynthesis, light reactio	57	333	1.30E-07	2.50E-05	MYB63.down
GO:0009723	P	response to ethylene stimulu	59	353	1.60E-07	3.00E-05	MYB63.down
GO:0006631	P	fatty acid metabolic process	84	587	2.10E-07	3.60E-05	MYB63.down
GO:0009741	P	response to brassinosteroid s	29	114	2.00E-07	3.60E-05	MYB63.down
GO:0006633	P	fatty acid biosynthetic proce:	52	303	4.20E-07	7.20E-05	MYB63.down
GO:0009695	P	jasmonic acid biosynthetic pr	31	136	6.10E-07	0.0001	MYB63.down
GO:0071495	P	cellular response to endogen	105	815	6.60E-07	0.00011	MYB63.down
GO:0009269	P	response to desiccation	16	39	7.30E-07	0.00012	MYB63.down
GO:0009853	P	photorespiration	34	161	8.20E-07	0.00013	MYB63.down
GO:0009737	P	response to abscisic acid stin	85	621	9.10E-07	0.00014	MYB63.down
GO:0009873	P	ethylene mediated signaling	29	125	1.00E-06	0.00015	MYB63.down
GO:0031408	P	oxylipin biosynthetic process	31	142	1.30E-06	0.0002	MYB63.down
GO:0009620	P	response to fungus	71	499	2.10E-06	0.0003	MYB63.down
GO:0006575	P	cellular amino acid derivative	92	714	3.20E-06	0.00045	MYB63.down
GO:0071369	P	cellular response to ethylene	30	144	4.50E-06	0.00061	MYB63.down
GO:0043094	P	cellular metabolic compounc	35	185	5.00E-06	0.00066	MYB63.down
GO:0006555	P	methionine metabolic proce:	41	236	5.20E-06	0.00068	MYB63.down
GO:0006301	P	postreplication repair	14	36	5.90E-06	0.00076	MYB63.down
GO:0019725	P	cellular homeostasis	51	328	6.70E-06	0.00084	MYB63.down
GO:0009607	P	response to biotic stimulus	180	1687	8.70E-06	0.0011	MYB63.down
GO:0000096	P	sulfur amino acid metabolic		65	465	9.30E-06	0.0011	MYB63.down
GO:0009612	P	response to mechanical stim	18	63	1.10E-05	0.0013	MYB63.down
GO:0045454	P	cell redox homeostasis	28	136	1.10E-05	0.0013	MYB63.down
GO:0006091	P	generation of precursor meta	91	730	1.20E-05	0.0014	MYB63.down
GO:0070838	P	divalent metal ion transport	35	195	1.30E-05	0.0015	MYB63.down
GO:0042398	P	cellular amino acid derivative	71	534	1.70E-05	0.0019	MYB63.down
GO:0022900	P	electron transport chain	27	133	2.00E-05	0.0022	MYB63.down
GO:0015824	P	proline transport	19	74	2.20E-05	0.0024	MYB63.down
GO:0009755	P	hormone-mediated signaling	77	600	2.20E-05	0.0024	MYB63.down
GO:0051707	P	response to other organism	153	1421	2.80E-05	0.0029	MYB63.down
GO:0015804	P	neutral amino acid transport	19	77	3.50E-05	0.0036	MYB63.down
GO:0032870	P	cellular response to hormone	80	641	3.70E-05	0.0038	MYB63.down
GO:0007242	P	intracellular signaling cascad	137	1252	3.70E-05	0.0038	MYB63.down
GO:0006790	P	sulfur metabolic process	84	683	3.80E-05	0.0038	MYB63.down
GO:0009767	P	photosynthetic electron tran	19	80	5.40E-05	0.0053	MYB63.down
GO:0002679	P	respiratory burst during defe	24	121	7.60E-05	0.0072	MYB63.down
GO:0045730	P	respiratory burst	24	121	7.60E-05	0.0072	MYB63.down
GO:0006007	P	glucose catabolic process	62	474	8.40E-05	0.0079	MYB63.down
GO:0019320	P	hexose catabolic process	62	476	9.30E-05	0.0086	MYB63.down
GO:0006098	P	pentose-phosphate shunt	33	200	9.90E-05	0.009	MYB63.down
GO:0042592	P	homeostatic process	63	488	0.0001	0.0093	MYB63.down
GO:0044283	P	small molecule biosynthetic		188	1865	0.0001	0.0093	MYB63.down
GO:0006740	P	NADPH regeneration	33	201	0.00011	0.0094	MYB63.down
GO:0044282	P	small molecule catabolic pro	105	932	0.00011	0.0095	MYB63.down
GO:0046365	P	monosaccharide catabolic pr	62	480	0.00011	0.0098	MYB63.down

Supplemental Table 2A-GO terms from *Pro35S::MYB63* compared to WT

GO:0046496	P	nicotinamide nucleotide met	34	212	0.00013	0.01	MYB63.down
GO:0006952	P	defense response	169	1653	0.00013	0.01	MYB63.down
GO:0006769	P	nicotinamide metabolic proc	34	212	0.00013	0.01	MYB63.down
GO:0009628	P	response to abiotic stimulus	253	2635	0.00013	0.01	MYB63.down
GO:0010207	P	photosystem II assembly	30	177	0.00013	0.011	MYB63.down
GO:0006739	P	NADP metabolic process	33	204	0.00014	0.011	MYB63.down
GO:0009773	P	photosynthetic electron tran	14	51	0.00014	0.011	MYB63.down
GO:0009066	P	aspartate family amino acid r	41	278	0.00014	0.011	MYB63.down
GO:0009266	P	response to temperature stir	107	962	0.00015	0.011	MYB63.down
GO:0019362	P	pyridine nucleotide metaboli	34	214	0.00015	0.011	MYB63.down
GO:0042538	P	hyperosmotic salinity respon	28	162	0.00016	0.012	MYB63.down
GO:0046164	P	alcohol catabolic process	62	491	0.0002	0.015	MYB63.down
GO:0009625	P	response to insect	17	76	0.00024	0.018	MYB63.down
GO:0006733	P	oxidoreduction coenzyme m	39	267	0.00024	0.018	MYB63.down
GO:0010286	P	heat acclimation	18	84	0.00026	0.018	MYB63.down
GO:0043603	P	cellular amide metabolic pro	34	222	0.00027	0.02	MYB63.down
GO:0042402	P	cellular biogenic amine catab	22	117	0.00029	0.02	MYB63.down
GO:0035304	P	regulation of protein amino a	24	135	0.00032	0.023	MYB63.down
GO:0009867	P	jasmonic acid mediated signa	40	282	0.00035	0.023	MYB63.down
GO:0009820	P	alkaloid metabolic process	34	225	0.00034	0.023	MYB63.down
GO:0071395	P	cellular response to jasmonic	40	282	0.00035	0.023	MYB63.down
GO:0006569	P	tryptophan catabolic process	17	79	0.00036	0.024	MYB63.down
GO:0009409	P	response to cold	74	629	0.00036	0.024	MYB63.down
GO:0046218	P	indolalkylamine catabolic pro	17	79	0.00036	0.024	MYB63.down
GO:0035303	P	regulation of dephosphorylat	24	137	0.00039	0.025	MYB63.down
GO:0015992	P	proton transport	25	147	0.00044	0.028	MYB63.down
GO:0009631	P	cold acclimation	10	31	0.00043	0.028	MYB63.down
GO:0006818	P	hydrogen transport	25	147	0.00044	0.028	MYB63.down
GO:0042436	P	indole derivative catabolic pr	17	81	0.00046	0.029	MYB63.down
GO:0070887	P	cellular response to chemica	144	1417	0.00049	0.031	MYB63.down
GO:0006972	P	hyperosmotic response	36	251	0.00056	0.034	MYB63.down
GO:0050832	P	defense response to fungus	45	342	0.00066	0.04	MYB63.down
GO:0015035	F	protein disulfide oxidoreduct	27	94	6.70E-08	8.00E-05	MYB63.down
GO:0015036	F	disulfide oxidoreductase acti	27	102	2.70E-07	0.00016	MYB63.down
GO:0030611	F	arsenate reductase activity	10	15	3.60E-06	0.0014	MYB63.down
GO:0030613	F	oxidoreductase activity, actir	9	14	1.40E-05	0.0027	MYB63.down
GO:0008794	F	arsenate reductase (glutared	9	14	1.40E-05	0.0027	MYB63.down
GO:0030614	F	oxidoreductase activity, actir	9	14	1.40E-05	0.0027	MYB63.down
GO:0005509	F	calcium ion binding	42	258	1.60E-05	0.0028	MYB63.down
GO:0005102	F	receptor binding	18	69	3.00E-05	0.0044	MYB63.down
GO:0003735	F	structural constituent of ribo	56	411	6.90E-05	0.0092	MYB63.down
GO:0015078	F	hydrogen ion transmembran	25	142	0.00028	0.033	MYB63.down
GO:0030414	F	peptidase inhibitor activity	12	44	0.00044	0.047	MYB63.down
GO:0034357	C	photosynthetic membrane	87	355	1.50E-18	5.70E-16	MYB63.down
GO:0044436	C	thylakoid part	92	388	9.60E-19	5.70E-16	MYB63.down
GO:0042651	C	thylakoid membrane	83	341	1.30E-17	3.30E-15	MYB63.down
GO:0009535	C	chloroplast thylakoid membr	78	322	1.60E-16	3.00E-14	MYB63.down
GO:0055035	C	plastid thylakoid membrane	78	324	2.10E-16	3.20E-14	MYB63.down
GO:0031976	C	plastid thylakoid	90	425	9.30E-16	1.00E-13	MYB63.down
GO:0009534	C	chloroplast thylakoid	90	425	9.30E-16	1.00E-13	MYB63.down
GO:0031984	C	organelle subcompartment	90	428	1.30E-15	1.30E-13	MYB63.down
GO:0009579	C	thylakoid	101	518	1.80E-15	1.50E-13	MYB63.down
GO:0044444	C	cytoplasmic part	915	9995	6.50E-12	4.90E-10	MYB63.down
GO:0070469	C	respiratory chain	37	112	9.70E-12	6.70E-10	MYB63.down
GO:0031090	C	organelle membrane	172	1327	1.10E-10	6.90E-09	MYB63.down

Supplemental Table 2A-GO terms from *Pro35S::MYB63* compared to WT

GO:0005746	C	mitochondrial respiratory ch	34	106	1.30E-10	7.40E-09	MYB63.down
GO:0044455	C	mitochondrial membrane pa	41	171	2.90E-09	1.60E-07	MYB63.down
GO:0009536	C	plastid	402	4037	2.20E-08	1.10E-06	MYB63.down
GO:0045271	C	respiratory chain complex I	24	72	3.50E-08	1.50E-06	MYB63.down
GO:0030964	C	NADH dehydrogenase compl	24	72	3.50E-08	1.50E-06	MYB63.down
GO:0009507	C	chloroplast	394	3959	3.30E-08	1.50E-06	MYB63.down
GO:0009521	C	photosystem	23	68	5.40E-08	2.20E-06	MYB63.down
GO:0005737	C	cytoplasm	1051	12233	1.00E-07	3.90E-06	MYB63.down
GO:0005747	C	mitochondrial respiratory ch	21	68	7.00E-07	2.50E-05	MYB63.down
GO:0005739	C	mitochondrion	350	3571	9.20E-07	3.20E-05	MYB63.down
GO:0005740	C	mitochondrial envelope	48	278	9.90E-07	3.30E-05	MYB63.down
GO:0031966	C	mitochondrial membrane	46	262	1.10E-06	3.50E-05	MYB63.down
GO:0005743	C	mitochondrial inner membra	42	229	1.20E-06	3.60E-05	MYB63.down
GO:0044429	C	mitochondrial part	51	328	6.70E-06	0.00019	MYB63.down
GO:0019866	C	organelle inner membrane	46	285	8.00E-06	0.00022	MYB63.down
GO:0044435	C	plastid part	139	1252	1.70E-05	0.00047	MYB63.down
GO:0044434	C	chloroplast part	134	1211	2.80E-05	0.00073	MYB63.down
GO:0044425	C	membrane part	241	2448	4.50E-05	0.0011	MYB63.down
GO:0009522	C	photosystem I	10	22	4.50E-05	0.0011	MYB63.down
GO:0033176	C	proton-transporting V-type A	9	18	6.10E-05	0.0014	MYB63.down
GO:0043229	C	intracellular organelle	1419	17570	7.10E-05	0.0016	MYB63.down
GO:0043226	C	organelle	1419	17578	7.90E-05	0.0018	MYB63.down
GO:0016469	C	proton-transporting two-sec	15	55	8.80E-05	0.0019	MYB63.down
GO:0031975	C	envelope	105	929	9.80E-05	0.002	MYB63.down
GO:0031967	C	organelle envelope	105	929	9.80E-05	0.002	MYB63.down
GO:0009523	C	photosystem II	13	46	0.00019	0.0038	MYB63.down
GO:0032991	C	macromolecular complex	201	2050	0.00024	0.0047	MYB63.down
GO:0009654	C	oxygen evolving complex	9	23	0.00026	0.005	MYB63.down
GO:0005750	C	mitochondrial respiratory ch	8	18	0.00029	0.0053	MYB63.down
GO:0045275	C	respiratory chain complex III	8	18	0.00029	0.0053	MYB63.down
GO:0043231	C	intracellular membrane-bour	1393	17343	0.00033	0.0058	MYB63.down
GO:0043227	C	membrane-bounded organel	1393	17349	0.00035	0.0061	MYB63.down
GO:0044446	C	intracellular organelle part	309	3379	0.0005	0.0085	MYB63.down
GO:0044422	C	organelle part	309	3385	0.00056	0.0093	MYB63.down
GO:0005840	C	ribosome	58	484	0.001	0.016	MYB63.down
GO:0031978	C	plastid thylakoid lumen	14	71	0.0024	0.036	MYB63.down
GO:0009543	C	chloroplast thylakoid lumen	14	71	0.0024	0.036	MYB63.down

Supplemental Table 2B-GO terms from *Pro35S::MYB63 Pro::LAC17* to WT

GO:0033179	C	proton-transporting V-type A	5	10	0.0028	0.043	MYB63.down
GO:0009627	P	systemic acquired resistance	42	445	1.10E-18	4.00E-15	MYB63::LAC17.up
GO:0009814	P	defense response, incompati	42	536	5.30E-16	9.50E-13	MYB63::LAC17.up
GO:0009696	P	salicylic acid metabolic proce	27	222	7.70E-15	9.20E-12	MYB63::LAC17.up
GO:0009697	P	salicylic acid biosynthetic prc	26	209	1.50E-14	1.10E-11	MYB63::LAC17.up
GO:0019438	P	aromatic compound biosynth	45	680	1.50E-14	1.10E-11	MYB63::LAC17.up
GO:0051707	P	response to other organism	62	1421	7.40E-12	3.80E-09	MYB63::LAC17.up
GO:0006725	P	cellular aromatic compound	51	1022	6.90E-12	3.80E-09	MYB63::LAC17.up
GO:0045087	P	innate immune response	48	930	9.90E-12	4.40E-09	MYB63::LAC17.up
GO:0044283	P	small molecule biosynthetic	73	1865	1.10E-11	4.60E-09	MYB63::LAC17.up
GO:0046394	P	carboxylic acid biosynthetic p	53	1116	1.50E-11	4.90E-09	MYB63::LAC17.up
GO:0016053	P	organic acid biosynthetic pro	53	1116	1.50E-11	4.90E-09	MYB63::LAC17.up
GO:0002376	P	immune system process	49	984	2.00E-11	5.40E-09	MYB63::LAC17.up
GO:0006955	P	immune response	49	984	2.00E-11	5.40E-09	MYB63::LAC17.up
GO:0034976	P	response to endoplasmic ret	28	352	3.30E-11	8.40E-09	MYB63::LAC17.up
GO:0006952	P	defense response	66	1653	5.80E-11	1.40E-08	MYB63::LAC17.up
GO:0050896	P	response to stimulus	165	6292	7.40E-11	1.70E-08	MYB63::LAC17.up
GO:0009607	P	response to biotic stimulus	66	1687	1.30E-10	2.80E-08	MYB63::LAC17.up
GO:0009863	P	salicylic acid mediated signal	26	349	6.20E-10	1.20E-07	MYB63::LAC17.up
GO:0071446	P	cellular response to salicylic a	26	351	6.90E-10	1.30E-07	MYB63::LAC17.up
GO:0043436	P	oxoacid metabolic process	74	2103	8.70E-10	1.50E-07	MYB63::LAC17.up
GO:0006082	P	organic acid metabolic proce	74	2105	9.00E-10	1.50E-07	MYB63::LAC17.up
GO:0019752	P	carboxylic acid metabolic prc	74	2103	8.70E-10	1.50E-07	MYB63::LAC17.up
GO:0042180	P	cellular ketone metabolic prc	74	2123	1.30E-09	2.00E-07	MYB63::LAC17.up
GO:0048585	P	negative regulation of respor	25	349	2.80E-09	4.20E-07	MYB63::LAC17.up
GO:0044281	P	small molecule metabolic prc	115	4065	3.40E-09	4.60E-07	MYB63::LAC17.up
GO:0031348	P	negative regulation of defens	22	273	3.50E-09	4.60E-07	MYB63::LAC17.up
GO:0031347	P	regulation of defense respon	31	529	3.40E-09	4.60E-07	MYB63::LAC17.up
GO:0009751	P	response to salicylic acid stin	29	470	3.70E-09	4.80E-07	MYB63::LAC17.up
GO:0048583	P	regulation of response to stir	35	667	4.80E-09	6.00E-07	MYB63::LAC17.up
GO:0080134	P	regulation of response to stre	31	544	6.30E-09	7.50E-07	MYB63::LAC17.up
GO:0009805	P	coumarin biosynthetic proce	11	54	7.10E-09	8.20E-07	MYB63::LAC17.up
GO:0009804	P	coumarin metabolic process	11	55	8.30E-09	9.40E-07	MYB63::LAC17.up
GO:0008152	P	metabolic process	285	13633	1.10E-08	1.20E-06	MYB63::LAC17.up
GO:0051704	P	multi-organism process	64	1820	1.50E-08	1.50E-06	MYB63::LAC17.up
GO:0006950	P	response to stress	113	4089	1.80E-08	1.80E-06	MYB63::LAC17.up
GO:0044238	P	primary metabolic process	251	11717	3.70E-08	3.70E-06	MYB63::LAC17.up
GO:0006468	P	protein amino acid phosphor	46	1134	3.90E-08	3.80E-06	MYB63::LAC17.up
GO:0016310	P	phosphorylation	51	1354	6.50E-08	6.20E-06	MYB63::LAC17.up
GO:0044237	P	cellular metabolic process	246	11509	7.80E-08	7.20E-06	MYB63::LAC17.up
GO:0006796	P	phosphate metabolic process	56	1571	8.40E-08	7.50E-06	MYB63::LAC17.up
GO:0006793	P	phosphorus metabolic proce	56	1572	8.50E-08	7.50E-06	MYB63::LAC17.up
GO:0045088	P	regulation of innate immune	24	415	2.60E-07	2.20E-05	MYB63::LAC17.up
GO:0002682	P	regulation of immune system	24	419	3.00E-07	2.50E-05	MYB63::LAC17.up
GO:0050776	P	regulation of immune respor	24	419	3.00E-07	2.50E-05	MYB63::LAC17.up
GO:0006612	P	protein targeting to membra	23	392	3.60E-07	2.90E-05	MYB63::LAC17.up
GO:0009862	P	systemic acquired resistance	18	251	4.60E-07	3.60E-05	MYB63::LAC17.up
GO:0034641	P	cellular nitrogen compound r	49	1373	5.60E-07	4.30E-05	MYB63::LAC17.up
GO:0012501	P	programmed cell death	24	451	1.00E-06	7.80E-05	MYB63::LAC17.up
GO:0043067	P	regulation of programmed ce	22	397	1.60E-06	0.00012	MYB63::LAC17.up
GO:0008219	P	cell death	25	500	1.80E-06	0.00013	MYB63::LAC17.up
GO:0016265	P	death	25	500	1.80E-06	0.00013	MYB63::LAC17.up
GO:0009626	P	plant-type hypersensitive res	22	401	1.80E-06	0.00013	MYB63::LAC17.up
GO:0034050	P	host programmed cell death	22	402	1.90E-06	0.00013	MYB63::LAC17.up
GO:0010363	P	regulation of plant-type hype	21	371	2.00E-06	0.00013	MYB63::LAC17.up

Supplemental Table 2B-GO terms from *Pro35S::MYB63 Pro::LAC17* to WT

GO:0010941	P	regulation of cell death	22	405	2.20E-06	0.00014	MYB63::LAC17.up
GO:0043687	P	post-translational protein mo	63	2075	2.70E-06	0.00017	MYB63::LAC17.up
GO:0080135	P	regulation of cellular respons	21	379	2.70E-06	0.00017	MYB63::LAC17.up
GO:0009987	P	cellular process	286	14419	3.20E-06	0.0002	MYB63::LAC17.up
GO:0000165	P	MAPKKK cascade	15	209	4.10E-06	0.00025	MYB63::LAC17.up
GO:0032787	P	monocarboxylic acid metabo	49	1481	4.30E-06	0.00026	MYB63::LAC17.up
GO:0007165	P	signal transduction	53	1670	5.50E-06	0.00032	MYB63::LAC17.up
GO:0023046	P	signaling process	55	1768	6.30E-06	0.00036	MYB63::LAC17.up
GO:0023060	P	signal transmission	55	1767	6.20E-06	0.00036	MYB63::LAC17.up
GO:0050789	P	regulation of biological proce	125	5235	6.50E-06	0.00037	MYB63::LAC17.up
GO:0009617	P	response to bacterium	26	577	6.80E-06	0.00038	MYB63::LAC17.up
GO:0044271	P	cellular nitrogen compound b	32	804	7.10E-06	0.00039	MYB63::LAC17.up
GO:0023052	P	signaling	68	2376	7.20E-06	0.00039	MYB63::LAC17.up
GO:0006984	P	ER-nuclear signaling pathway	14	193	7.70E-06	0.00041	MYB63::LAC17.up
GO:0050832	P	defense response to fungus	19	342	7.80E-06	0.00041	MYB63::LAC17.up
GO:0007243	P	protein kinase cascade	15	223	8.60E-06	0.00044	MYB63::LAC17.up
GO:0043069	P	negative regulation of progra	13	170	9.60E-06	0.00048	MYB63::LAC17.up
GO:0042221	P	response to chemical stimulu	100	3978	9.90E-06	0.00049	MYB63::LAC17.up
GO:0060548	P	negative regulation of cell de	13	174	1.20E-05	0.00059	MYB63::LAC17.up
GO:0023034	P	intracellular signaling pathwa	21	433	1.90E-05	0.00091	MYB63::LAC17.up
GO:0051716	P	cellular response to stimulus	66	2355	1.90E-05	0.00091	MYB63::LAC17.up
GO:0071310	P	cellular response to organic s	41	1234	2.50E-05	0.0012	MYB63::LAC17.up
GO:0006519	P	cellular amino acid and deriv	43	1324	2.60E-05	0.0012	MYB63::LAC17.up
GO:0023033	P	signaling pathway	23	519	3.00E-05	0.0014	MYB63::LAC17.up
GO:0007242	P	intracellular signaling cascad	41	1252	3.40E-05	0.0016	MYB63::LAC17.up
GO:0033554	P	cellular response to stress	46	1473	3.50E-05	0.0016	MYB63::LAC17.up
GO:0043412	P	macromolecule modification	82	3199	3.90E-05	0.0017	MYB63::LAC17.up
GO:0010033	P	response to organic substanc	73	2754	4.00E-05	0.0017	MYB63::LAC17.up
GO:0048511	P	rhythmic process	12	171	4.60E-05	0.002	MYB63::LAC17.up
GO:0007623	P	circadian rhythm	12	171	4.60E-05	0.002	MYB63::LAC17.up
GO:0009620	P	response to fungus	22	499	4.70E-05	0.002	MYB63::LAC17.up
GO:0070887	P	cellular response to chemica	44	1417	5.90E-05	0.0025	MYB63::LAC17.up
GO:0048519	P	negative regulation of biolog	40	1243	6.20E-05	0.0025	MYB63::LAC17.up
GO:0044106	P	cellular amine metabolic pro	33	947	6.60E-05	0.0027	MYB63::LAC17.up
GO:0050794	P	regulation of cellular process	108	4595	6.70E-05	0.0027	MYB63::LAC17.up
GO:0006598	P	polyamine catabolic process	6	38	7.20E-05	0.0029	MYB63::LAC17.up
GO:0065007	P	biological regulation	138	6222	7.30E-05	0.0029	MYB63::LAC17.up
GO:0009611	P	response to wounding	17	340	8.10E-05	0.0032	MYB63::LAC17.up
GO:0006464	P	protein modification process	72	2773	8.50E-05	0.0033	MYB63::LAC17.up
GO:0006520	P	cellular amino acid metabolic	31	882	9.40E-05	0.0036	MYB63::LAC17.up
GO:0030968	P	endoplasmic reticulum unfol	12	185	9.40E-05	0.0036	MYB63::LAC17.up
GO:0010310	P	regulation of hydrogen perox	12	187	0.0001	0.0038	MYB63::LAC17.up
GO:0034620	P	cellular response to unfolded	12	187	0.0001	0.0038	MYB63::LAC17.up
GO:0006986	P	response to unfolded protein	12	187	0.0001	0.0038	MYB63::LAC17.up
GO:0080010	P	regulation of oxygen and rea	12	188	0.00011	0.0039	MYB63::LAC17.up
GO:0010200	P	response to chitin	19	421	0.00011	0.0041	MYB63::LAC17.up
GO:0046942	P	carboxylic acid transport	15	283	0.00011	0.0041	MYB63::LAC17.up
GO:0015849	P	organic acid transport	15	289	0.00014	0.005	MYB63::LAC17.up
GO:0008652	P	cellular amino acid biosynthe	21	503	0.00014	0.005	MYB63::LAC17.up
GO:0071445	P	cellular response to protein s	12	194	0.00014	0.005	MYB63::LAC17.up
GO:0009308	P	amine metabolic process	34	1034	0.00015	0.0051	MYB63::LAC17.up
GO:0006605	P	protein targeting	31	906	0.00015	0.0051	MYB63::LAC17.up
GO:0071216	P	cellular response to biotic sti	12	197	0.00016	0.0055	MYB63::LAC17.up
GO:0043648	P	dicarboxylic acid metabolic p	10	143	0.0002	0.0067	MYB63::LAC17.up
GO:0006865	P	amino acid transport	14	266	0.00021	0.0068	MYB63::LAC17.up

Supplemental Table 2B-GO terms from *Pro35S::MYB63 Pro::LAC17* to WT

GO:0042743	P	hydrogen peroxide metaboli	16	335	0.00021	0.007	MYB63::LAC17.up
GO:0015837	P	amine transport	14	272	0.00026	0.0083	MYB63::LAC17.up
GO:0009309	P	amine biosynthetic process	21	527	0.00026	0.0085	MYB63::LAC17.up
GO:0006800	P	oxygen and reactive oxygen s	16	347	0.00031	0.0098	MYB63::LAC17.up
GO:0042398	P	cellular amino acid derivative	21	534	0.00031	0.0098	MYB63::LAC17.up
GO:0009962	P	regulation of flavonoid biosy	9	124	0.00032	0.01	MYB63::LAC17.up
GO:0006862	P	nucleotide transport	5	35	0.00044	0.014	MYB63::LAC17.up
GO:0009963	P	positive regulation of flavonc	8	103	0.00045	0.014	MYB63::LAC17.up
GO:0009791	P	post-embryonic developmen	57	2188	0.00046	0.014	MYB63::LAC17.up
GO:0009595	P	detection of biotic stimulus	8	104	0.00048	0.014	MYB63::LAC17.up
GO:0009699	P	phenylpropanoid biosyntheti	15	327	0.0005	0.015	MYB63::LAC17.up
GO:0009790	P	embryonic development	23	638	0.00053	0.016	MYB63::LAC17.up
GO:0009698	P	phenylpropanoid metabolic p	17	405	0.00057	0.017	MYB63::LAC17.up
GO:0009743	P	response to carbohydrate sti	27	812	0.0006	0.018	MYB63::LAC17.up
GO:0006595	P	polyamine metabolic process	6	60	0.00069	0.02	MYB63::LAC17.up
GO:0046777	P	protein amino acid autophos	9	146	0.00097	0.028	MYB63::LAC17.up
GO:0042402	P	cellular biogenic amine catab	8	117	0.00099	0.028	MYB63::LAC17.up
GO:0046483	P	heterocycle metabolic proce:	31	1023	0.0011	0.03	MYB63::LAC17.up
GO:0009867	P	jasmonic acid mediated signa	13	282	0.0011	0.031	MYB63::LAC17.up
GO:0071395	P	cellular response to jasmonic	13	282	0.0011	0.031	MYB63::LAC17.up
GO:0009753	P	response to jasmonic acid sti	18	471	0.0011	0.031	MYB63::LAC17.up
GO:0032502	P	developmental process	92	4094	0.0011	0.031	MYB63::LAC17.up
GO:0042742	P	defense response to bacteriu	16	394	0.0011	0.031	MYB63::LAC17.up
GO:0009310	P	amine catabolic process	11	214	0.0012	0.031	MYB63::LAC17.up
GO:0009628	P	response to abiotic stimulus	64	2635	0.0012	0.032	MYB63::LAC17.up
GO:0006886	P	intracellular protein transpor	31	1044	0.0015	0.039	MYB63::LAC17.up
GO:0043455	P	regulation of secondary meta	9	157	0.0016	0.041	MYB63::LAC17.up
GO:0006807	P	nitrogen compound metabol	120	5675	0.0016	0.043	MYB63::LAC17.up
GO:0009813	P	flavonoid biosynthetic proce:	11	225	0.0017	0.044	MYB63::LAC17.up
GO:0048608	P	reproductive structure devel	45	1722	0.0018	0.045	MYB63::LAC17.up
GO:0007166	P	cell surface receptor linked s	9	160	0.0018	0.046	MYB63::LAC17.up
GO:0034613	P	cellular protein localization	31	1059	0.0018	0.046	MYB63::LAC17.up
GO:0019748	P	secondary metabolic process	35	1247	0.0019	0.047	MYB63::LAC17.up
GO:0051789	P	response to protein stimulus	15	376	0.0019	0.048	MYB63::LAC17.up
GO:0003824	F	catalytic activity	222	8787	6.30E-14	3.40E-11	MYB63::LAC17.up
GO:0016740	F	transferase activity	100	3012	1.20E-11	3.10E-09	MYB63::LAC17.up
GO:0000166	F	nucleotide binding	101	3438	6.50E-09	1.10E-06	MYB63::LAC17.up
GO:0016772	F	transferase activity, transferr	58	1590	2.20E-08	2.90E-06	MYB63::LAC17.up
GO:0016301	F	kinase activity	52	1356	2.80E-08	2.90E-06	MYB63::LAC17.up
GO:0016773	F	phosphotransferase activity,	48	1275	1.70E-07	1.50E-05	MYB63::LAC17.up
GO:0017076	F	purine nucleotide binding	78	2595	2.10E-07	1.60E-05	MYB63::LAC17.up
GO:0004672	F	protein kinase activity	43	1113	3.90E-07	2.30E-05	MYB63::LAC17.up
GO:0005524	F	ATP binding	64	1996	3.70E-07	2.30E-05	MYB63::LAC17.up
GO:0032555	F	purine ribonucleotide bindin	73	2429	5.50E-07	2.70E-05	MYB63::LAC17.up
GO:0032553	F	ribonucleotide binding	73	2429	5.50E-07	2.70E-05	MYB63::LAC17.up
GO:0004674	F	protein serine/threonine kin	38	940	6.80E-07	3.00E-05	MYB63::LAC17.up
GO:0001883	F	purine nucleoside binding	70	2333	1.00E-06	4.00E-05	MYB63::LAC17.up
GO:0030554	F	adenyl nucleotide binding	70	2331	1.00E-06	4.00E-05	MYB63::LAC17.up
GO:0001882	F	nucleoside binding	70	2341	1.20E-06	4.20E-05	MYB63::LAC17.up
GO:0032559	F	adenyl ribonucleotide bindin	65	2169	2.80E-06	9.20E-05	MYB63::LAC17.up
GO:0050661	F	NADP or NADPH binding	8	66	2.60E-05	0.00081	MYB63::LAC17.up
GO:0050662	F	coenzyme binding	17	354	0.00013	0.0038	MYB63::LAC17.up
GO:0005488	F	binding	224	11247	0.00014	0.004	MYB63::LAC17.up
GO:0048037	F	cofactor binding	20	479	0.00021	0.0055	MYB63::LAC17.up
GO:0016829	F	lyase activity	15	347	0.00089	0.023	MYB63::LAC17.up

Supplemental Table 2B-GO terms from *Pro35S::MYB63 Pro::LAC17* to WT

GO:0044464	C	cell part	417	22455	2.40E-09	4.90E-07	MYB63::LAC17.up
GO:0005623	C	cell	417	22455	2.40E-09	4.90E-07	MYB63::LAC17.up
GO:0044424	C	intracellular part	367	19957	2.60E-05	0.0024	MYB63::LAC17.up
GO:0005622	C	intracellular	368	20042	3.00E-05	0.0024	MYB63::LAC17.up
GO:0005737	C	cytoplasm	246	12233	1.80E-05	0.0024	MYB63::LAC17.up
GO:0009570	C	chloroplast stroma	25	603	3.80E-05	0.0026	MYB63::LAC17.up
GO:0009536	C	plastid	98	4037	4.90E-05	0.0028	MYB63::LAC17.up
GO:0009532	C	plastid stroma	25	637	8.90E-05	0.0041	MYB63::LAC17.up
GO:0044444	C	cytoplasmic part	204	9995	9.10E-05	0.0041	MYB63::LAC17.up
GO:0009507	C	chloroplast	93	3959	0.00025	0.01	MYB63::LAC17.up
GO:0044434	C	chloroplast part	37	1211	0.00031	0.011	MYB63::LAC17.up
GO:0005886	C	plasma membrane	88	3740	0.00036	0.012	MYB63::LAC17.up
GO:0044435	C	plastid part	37	1252	0.00057	0.018	MYB63::LAC17.up
GO:0016020	C	membrane	128	6122	0.0016	0.047	MYB63::LAC17.up
GO:0009719	P	response to endogenous stin	134	1615	5.20E-23	2.20E-19	MYB63::LAC17.down
GO:0010033	P	response to organic substanc	182	2754	8.30E-21	1.70E-17	MYB63::LAC17.down
GO:0009725	P	response to hormone stimulu	116	1375	1.90E-20	2.60E-17	MYB63::LAC17.down
GO:0010200	P	response to chitin	58	421	4.60E-19	4.80E-16	MYB63::LAC17.down
GO:0071369	P	cellular response to ethylene	32	144	4.30E-16	3.60E-13	MYB63::LAC17.down
GO:0009873	P	ethylene mediated signaling	30	125	6.00E-16	4.20E-13	MYB63::LAC17.down
GO:0009743	P	response to carbohydrate sti	75	812	2.30E-15	1.40E-12	MYB63::LAC17.down
GO:0042221	P	response to chemical stimulu	214	3978	7.80E-15	4.10E-12	MYB63::LAC17.down
GO:0000160	P	two-component signal transc	31	165	6.60E-14	3.10E-11	MYB63::LAC17.down
GO:0009723	P	response to ethylene stimulu	43	353	8.10E-13	3.40E-10	MYB63::LAC17.down
GO:0009611	P	response to wounding	42	340	9.90E-13	3.80E-10	MYB63::LAC17.down
GO:0009733	P	response to auxin stimulus	45	431	2.80E-11	9.80E-09	MYB63::LAC17.down
GO:0009692	P	ethylene metabolic process	23	131	3.60E-10	1.10E-07	MYB63::LAC17.down
GO:0009693	P	ethylene biosynthetic proces	23	131	3.60E-10	1.10E-07	MYB63::LAC17.down
GO:0043449	P	cellular alkene metabolic pro	23	133	4.70E-10	1.20E-07	MYB63::LAC17.down
GO:0043450	P	alkene biosynthetic process	23	133	4.70E-10	1.20E-07	MYB63::LAC17.down
GO:0002679	P	respiratory burst during defe	21	121	2.50E-09	5.90E-07	MYB63::LAC17.down
GO:0045730	P	respiratory burst	21	121	2.50E-09	5.90E-07	MYB63::LAC17.down
GO:0050896	P	response to stimulus	272	6292	1.50E-08	3.30E-06	MYB63::LAC17.down
GO:0009753	P	response to jasmonic acid sti	40	471	7.40E-08	1.50E-05	MYB63::LAC17.down
GO:0009612	P	response to mechanical stim	14	63	8.10E-08	1.60E-05	MYB63::LAC17.down
GO:0042398	P	cellular amino acid derivative	43	534	9.60E-08	1.80E-05	MYB63::LAC17.down
GO:0006575	P	cellular amino acid derivative	51	714	2.10E-07	3.90E-05	MYB63::LAC17.down
GO:0032870	P	cellular response to hormone	47	641	3.20E-07	5.60E-05	MYB63::LAC17.down
GO:0071495	P	cellular response to endogen	55	815	3.90E-07	6.50E-05	MYB63::LAC17.down
GO:0009755	P	hormone-mediated signaling	43	600	1.80E-06	0.00028	MYB63::LAC17.down
GO:0071310	P	cellular response to organic s	71	1234	2.20E-06	0.00035	MYB63::LAC17.down
GO:0023034	P	intracellular signaling pathwa	34	433	3.50E-06	0.00052	MYB63::LAC17.down
GO:0006555	P	methionine metabolic proces	23	236	5.60E-06	0.00081	MYB63::LAC17.down
GO:0002252	P	immune effector process	25	273	6.10E-06	0.00086	MYB63::LAC17.down
GO:0023033	P	signaling pathway	36	519	2.30E-05	0.0031	MYB63::LAC17.down
GO:0070887	P	cellular response to chemica	74	1417	3.20E-05	0.0043	MYB63::LAC17.down
GO:0042538	P	hyperosmotic salinity respon	17	162	3.90E-05	0.005	MYB63::LAC17.down
GO:0009605	P	response to external stimulu	60	1087	4.30E-05	0.0053	MYB63::LAC17.down
GO:0009066	P	aspartate family amino acid r	23	278	6.20E-05	0.0074	MYB63::LAC17.down
GO:0007568	P	aging	15	145	0.00013	0.015	MYB63::LAC17.down
GO:0009741	P	response to brassinosteroid s	13	114	0.00015	0.017	MYB63::LAC17.down
GO:0006354	P	RNA elongation	14	133	0.00018	0.02	MYB63::LAC17.down
GO:0045333	P	cellular respiration	14	141	0.00031	0.033	MYB63::LAC17.down
GO:0009737	P	response to abscisic acid stin	37	621	0.00033	0.033	MYB63::LAC17.down
GO:0007242	P	intracellular signaling cascad	63	1252	0.00033	0.033	MYB63::LAC17.down

Supplemental Table 2B-GO terms from *Pro35S::MYB63 Pro::LAC17* to WT

GO:0009694	P	jasmonic acid metabolic proc	15	160	0.00034	0.034	MYB63::LAC17.down
GO:0000096	P	sulfur amino acid metabolic p	30	465	0.00035	0.034	MYB63::LAC17.down
GO:0006952	P	defense response	78	1653	0.00043	0.041	MYB63::LAC17.down
GO:0009620	P	response to fungus	31	499	0.00051	0.047	MYB63::LAC17.down
GO:0006661	P	phosphatidylinositol biosynth	11	98	0.00052	0.048	MYB63::LAC17.down
GO:0031407	P	oxylipin metabolic process	15	168	0.00054	0.048	MYB63::LAC17.down
GO:0005102	F	receptor binding	14	69	2.10E-07	0.00011	MYB63::LAC17.down

Supplemental Table 3-RNA seq data comparing *Pro35S::MYB63 Pro35S::LAC17* with wild type.

DAP.MYB63.target(1.5kb upstream TSS)	Gene	baseMean	log2FoldChange	lfcMLE	lfcSE	stat	pvalue	padj (FDR)	TAIR10 Symbol	TAIR10 Annotation (Short)
YES	AT1G79180	383.26139	6.5691074	7.7546842	0.4610905	14.246892	4.69E-46	8.45E-42	ATMYB63	MYB DOMAIN PROTEIN 63
YES	AT1G11230	126.30761	6.5572684	12.681496	0.5625121	11.657114	2.11E-31	7.61E-28	NA	NA
	AT3G05727	23.226736	4.145434	10.235076	0.6488355	6.3890369	1.67E-10	2.59E-08	NA	NA
	AT5G66985	46.029155	4.0496997	5.4106588	0.5661701	7.1527972	8.50E-13	2.10E-10	NA	NA
YES	AT5G24760	465.02489	3.9909155	4.3200166	0.36327	10.986085	4.46E-28	8.93E-25	NA	NA
YES	AT1G52760	570.54077	3.8655313	4.1374873	0.3396908	11.379557	5.29E-30	1.59E-26	LysoPL2	lysophospholipase 2
YES	AT2G37040	1714.4476	3.5609574	3.7467171	0.2985783	11.926377	8.62E-33	5.18E-29	ATPAL1	0
	AT4G33980	188.27714	3.4911839	3.8750182	0.4085956	8.5443509	1.29E-17	1.01E-14	NA	NA
YES	AT5G40760	328.67602	3.4632643	3.8040759	0.3910491	8.8563404	8.27E-19	7.10E-16	G6PD6	glucose-6-phosphate dehydrogenase 6
YES	AT4G25810	808.83992	3.4120268	3.7727664	0.4037605	8.4506203	2.90E-17	1.93E-14	XTH23	xyloglucan endotransglucosylase/hydrolase 23
	AT1G43910	140.97875	3.2529827	3.8101227	0.481377	6.7576609	1.40E-11	2.75E-09	NA	NA
	AT3G07650	125.21063	3.2019584	3.4813379	0.3715251	8.6184177	6.79E-18	5.56E-15	BBX7	B-box domain protein 7
	AT4G36430	51.741931	3.1778432	3.8395414	0.5090393	6.2428249	4.30E-10	5.91E-08	NA	NA
	AT1G33950	41.659331	3.1370237	3.719631	0.4900367	6.40161	1.54E-10	2.42E-08	NA	NA
YES	AT4G15330	9.5365794	3.1364458	8.9533221	0.6765044	4.636253	3.55E-06	9.86E-05	CYP705A1	cytochrome P450, family 705, subfamily A, polypeptide 1
YES	AT5G35550	18.226489	3.1249382	4.6351027	0.6180596	5.0560469	4.28E-07	1.88E-05	ATMYB123	MYB DOMAIN PROTEIN 123
	AT3G17690	18.857778	3.0468714	4.0943576	0.5782724	5.2689208	1.37E-07	7.59E-06	ATCNGC19	CYCLIC NUCLEOTIDE-GATED CHANNEL 19
	AT4G05130	20.021346	3.0456647	4.2310773	0.597799	5.0947974	3.49E-07	1.61E-05	ATENT4	0
	AT3G11340	34.829674	3.0230171	3.9567942	0.5702458	5.3012528	1.15E-07	6.52E-06	UGT76B1	UDP-dependent glycosyltransferase 76B1
	AT1G62590	7.5952979	2.9705282	8.6263266	0.6790935	4.3742549	1.22E-05	0.0002688	AtPPR-AC	0
YES	AT2G45290	72.12575	2.9574989	3.3087638	0.419648	7.047571	1.82E-12	4.10E-10	TKL2	transketolase 2
	AT2G44480	100.018	2.9312025	3.3952164	0.4692964	6.245951	4.21E-10	5.84E-08	BGLU17	beta glucosidase 17
	AT3G59350	998.68765	2.9207598	3.1924571	0.3837681	7.6107416	2.73E-14	1.04E-11	NA	NA
	AT1G75040	4204.2047	2.8436653	3.0385787	0.3372158	8.4327769	3.38E-17	2.17E-14	PR-5	0
	AT5G48250	154.93228	2.8414641	3.0662523	0.3570886	7.9573085	1.76E-15	8.56E-13	BBX8	B-box domain protein 8
	AT1G51620	61.193735	2.8314563	3.2140572	0.4407924	6.4235595	1.33E-10	2.14E-08	NA	NA
YES	AT4G34050	16096.88	2.8311143	3.0609916	0.3626991	7.8056836	5.92E-15	2.60E-12	CCoAOMT1	caffeoyl coenzyme A O-methyltransferase 1
YES	AT3G21240	148.30202	2.7973241	3.0242341	0.3612766	7.7428872	9.72E-15	3.89E-12	4CL2	4-coumarate:CoA ligase 2
	AT3G11010	370.76171	2.7933509	3.0624266	0.388814	7.1842851	6.76E-13	1.74E-10	AtRLP34	receptor like protein 34
	AT5G54520	11.512877	2.7148197	4.4491498	0.6508582	4.1711386	3.03E-05	0.0005478	NA	NA
	AT5G64000	169.26447	2.7096425	2.9558496	0.3794889	7.1402424	9.32E-13	2.27E-10	ATSAL2	0
	AT1G64060	113.43271	2.709457	2.9581592	0.3803176	7.1241952	1.05E-12	2.48E-10	ATRBOH F	respiratory burst oxidase protein F
	AT4G21760	45.09433	2.7054992	3.4116488	0.5495835	4.9228173	8.53E-07	3.27E-05	BGLU47	beta-glucosidase 47
YES	AT5G42830	306.2237	2.7050054	2.9045646	0.3478186	7.7770569	7.42E-15	3.11E-12	NA	NA
	AT3G13610	103.35553	2.7024943	3.0839343	0.4498655	6.0073381	1.89E-09	2.02E-07	F6'H1	0
	AT1G24150	230.7776	2.6902464	2.9029925	0.3578709	7.517366	5.59E-14	1.80E-11	ATFH4	FORMIN HOMOLOGUE 4
	AT4G14590	9.068264	2.662128	4.6885111	0.6615535	4.0240554	5.72E-05	0.0008956	emb2739	embryo defective 2739
	AT3G26540	13.167558	2.6441066	3.8420885	0.6186102	4.2742692	1.92E-05	0.0003777	NA	NA
	AT5G61920	7.6768723	2.6189057	8.6464712	0.6875971	3.8087795	0.0001397	0.0018041	FLL4	FLOWERING LOCUS C EXPRESSOR-LIKE 4
YES	AT3G29200	808.99315	2.6035208	2.8111367	0.3597712	7.2366005	4.60E-13	1.22E-10	ATCM1	ARABIDOPSIS THALIANA CHORISMATE MUTASE 1

Supplemental Table 3-RNA seq data comparing *Pro35S::MYB63 Pro35S::LAC17* with wild type.

DAP.MYB63.target(1.5kb upstream TSS)	Gene	baseMean	log2FoldChange	lfcMLE	lfcSE	stat	pvalue	padj (FDR)	TAIR10 Symbol	TAIR10 Annotation (Short)
	AT4G1189C	717.61067	2.6002673	2.7425511	0.3051632	8.5209073	1.58E-17	1.14E-14	ARCK1	ABA- AND OSMOTIC-STRESS-INDUCIBLE RECEPTOR-LIKE CYTOSOLIC KINASE1
	AT1G1122C	172.84399	2.6001538	2.8309825	0.3756434	6.921868	4.46E-12	9.34E-10	NA	NA
	AT1G1570C	16.750198	2.587334	3.6753992	0.612758	4.2224405	2.42E-05	0.0004594	ATPC2	0
	AT1G62914	11.527168	2.5775789	3.9807689	0.6385001	4.036928	5.42E-05	0.0008606	NA	NA
	AT5G5210C	100.04311	2.5707555	2.8797657	0.4239626	6.063637	1.33E-09	1.53E-07	CRR1	chlororespiration reduction 1
	AT5G2420C	323.81469	2.5207621	2.688544	0.3325729	7.5795767	3.47E-14	1.30E-11	NA	NA
	AT1G17665	17.048868	2.5106272	3.6682244	0.6245116	4.0201454	5.82E-05	0.0009059	NA	NA
	AT5G5169C	26.923208	2.5072206	3.0532561	0.5201862	4.8198525	1.44E-06	4.88E-05	ACS12	1-amino-cyclopropane-1-carboxylate synthase 12
	AT2G4549C	8.1030049	2.5030098	4.5204839	0.6675719	3.7494237	0.0001772	0.0021596	AtAUR3	ataurora3
YES	AT3G1034C	114.00661	2.4971183	2.7749349	0.4111713	6.073183	1.25E-09	1.45E-07	PAL4	phenylalanine ammonia-lyase 4
	AT1G4413C	8.3665958	2.4779697	4.5713812	0.6710726	3.692551	0.000222	0.0025632	NA	NA
	AT1G6447C	11.473631	2.460177	3.9297668	0.6485867	3.7931354	0.0001488	0.0018898	NA	NA
	AT4G2009C	23.410823	2.4442716	3.1724346	0.5706908	4.2830054	1.84E-05	0.0003681	EMB1025	embryo defective 1025
	AT1G2303C	294.85476	2.4150179	2.6154643	0.3656397	6.6049121	3.98E-11	6.96E-09	NA	NA
	AT1G3396C	195.9947	2.4090516	2.6113091	0.3671447	6.5615857	5.32E-11	9.14E-09	AIG1	AVRRPT2-INDUCED GENE 1
	AT5G0761C	44.785572	2.4030995	2.7309228	0.4438762	5.4138957	6.17E-08	3.93E-06	NA	NA
	AT5G0110C	154.62672	2.4019739	2.6229998	0.3811659	6.3016498	2.94E-10	4.21E-08	FRB1	FRIABLE 1
	AT3G3203C	5.7489065	2.4000969	8.2283706	0.6886525	3.4852079	0.0004918	0.0046519	NA	NA
	AT4G0995C	29.790959	2.3958541	2.8864584	0.5096897	4.7006135	2.59E-06	7.60E-05	NA	NA
	AT1G1474C	285.00785	2.3930505	2.6265097	0.3910983	6.1187957	9.43E-10	1.15E-07	TTA1	TITANIA 1
	AT1G7945C	16.849938	2.3731714	3.1427293	0.5809598	4.0849149	4.41E-05	0.0007347	ALIS5	ALA-interacting subunit 5
	AT2G4096C	50.425505	2.3531827	2.7013795	0.4573948	5.1447515	2.68E-07	1.32E-05	NA	NA
	AT3G2466C	33.524995	2.3472795	2.7748404	0.4923081	4.7679081	1.86E-06	5.90E-05	TMKL1	transmembrane kinase-like 1
YES	AT5G6571C	68.731702	2.3467838	2.6077019	0.4105296	5.7164787	1.09E-08	9.12E-07	HSL2	HAESA-like 2
	AT2G1381C	72.307385	2.3416483	2.6004282	0.4096221	5.7166061	1.09E-08	9.12E-07	ALD1	AGD2-like defense response protein 1
	AT1G7139C	12.647619	2.3281484	3.4678852	0.6322529	3.6823057	0.0002311	0.0026413	AtRLP11	receptor like protein 11
	AT2G3910C	42.158023	2.3265264	2.7775296	0.5033848	4.6217653	3.80E-06	0.0001042	NA	NA
	AT4G22513	6.1412512	2.3199817	8.3134667	0.6886702	3.3687849	0.000755	0.0064223	NA	NA
	AT2G2171C	30.923931	2.3166729	2.7786803	0.5074673	4.5651668	4.99E-06	0.00013	EMB2219	embryo defective 2219
	AT5G0532C	22.241219	2.3161581	3.0221566	0.5736484	4.0375916	5.40E-05	0.0008597	NA	NA
	AT4G1332C	13.044565	2.3142651	3.3123114	0.6188047	3.739896	0.0001841	0.0022296	NA	NA
	AT2G3818C	75.153703	2.3141094	2.7069137	0.4824154	4.7969233	1.61E-06	5.31E-05	NA	NA
YES	AT1G1342C	7.2958974	2.3060225	4.3379269	0.6748554	3.4170617	0.000633	0.0056473	ATST4B	ARABIDOPSIS THALIANA SULFOTRANSFERASE 4B
	AT1G1270C	22.382227	2.3008215	2.9889965	0.5723926	4.0196561	5.83E-05	0.000907	RPF1	RNA processing factor 1
	AT3G0778C	470.47383	2.2957089	2.4351311	0.3197204	7.1803631	6.95E-13	1.76E-10	OBE1	OBERON1
YES	AT2G3049C	2930.1837	2.2815967	2.4254499	0.3252012	7.015954	2.28E-12	5.08E-10	ATC4H	CINNAMATE 4-HYDROXYLASE
	AT4G1146C	7.5019941	2.2750079	4.3455498	0.6765695	3.3625635	0.0007722	0.0065377	CRK30	cysteine-rich RLK (RECEPTOR-like protein kinase) 30
	AT5G6299C	16.542329	2.2734234	3.0956552	0.5973283	3.8059864	0.0001412	0.0018181	emb1692	embryo defective 1692
	AT2G0445C	223.16871	2.2727787	2.4587119	0.3635016	6.2524591	4.04E-10	5.66E-08	ATNUDT6	nudix hydrolase homolog 6
	AT1G1925C	18.471022	2.2643548	3.0692141	0.5946405	3.8079391	0.0001401	0.0018076	FMO1	flavin-dependent monooxygenase 1

Supplemental Table 3-RNA seq data comparing *Pro35S::MYB63 Pro35S::LAC17* with wild type.

DAP.MYB63.target(1.5kb upstream TSS)	Gene	baseMean	log2FoldChange	lfcMLE	lfcSE	stat	pvalue	padj (FDR)	TAIR10 Symbol	TAIR10 Annotation (Short)
	AT1G0705C	226.22211	2.2629046	2.4850505	0.392046	5.7720378	7.83E-09	7.04E-07	NA	NA
YES	AT1G4885C	839.42817	2.2508085	2.3865328	0.3187664	7.0609966	1.65E-12	3.77E-10	EMB1144	embryo defective 1144
	AT5G6273C	14.479466	2.2456946	3.0251375	0.5901201	3.8054876	0.0001415	0.0018191	NA	NA
	AT5G4290C	152.42463	2.2438663	2.5917604	0.4682465	4.7920624	1.65E-06	5.40E-05	COR27	cold regulated gene 27
	AT2G0443C	78.869703	2.2193923	2.4318195	0.3868885	5.736516	9.66E-09	8.33E-07	atnudt5	nudix hydrolase homolog 5
	AT4G0450C	39.486056	2.2143154	2.5511485	0.4623429	4.7893356	1.67E-06	5.41E-05	CRK37	cysteine-rich RLK (RECEPTOR-like protein kinase) 37
	AT4G3100C	43.985679	2.2060245	2.5898044	0.4863429	4.5359449	5.73E-06	0.0001451	NA	NA
	AT3G26782	22.017669	2.2017088	2.8281771	0.5649422	3.8972282	9.73E-05	0.0013614	NA	NA
YES	AT2G4416C	1156.5323	2.1966415	2.3699914	0.3583788	6.1293854	8.82E-10	1.10E-07	MTHFR2	methylenetetrahydrofolate reductase 2
YES	AT5G1657C	359.42757	2.1964974	2.3072758	0.2941632	7.4669357	8.21E-14	2.55E-11	GLN1;4	glutamine synthetase 1;4
	AT5G3825C	11.378935	2.1921312	3.2671545	0.6329875	3.463151	0.0005339	0.0049587	NA	NA
	AT4G3748C	25.368538	2.1847242	2.790405	0.5618899	3.8881714	0.000101	0.0013958	NA	NA
YES	AT3G5326C	1708.2347	2.1838989	2.2999024	0.3013238	7.2476806	4.24E-13	1.14E-10	ATPAL2	0
YES	AT1G0942C	19.444055	2.1765039	2.7935854	0.5628593	3.86687	0.0001102	0.0015005	G6PD4	glucose-6-phosphate dehydrogenase 4
	AT1G6014C	485.14319	2.1746894	2.3553653	0.366138	5.9395356	2.86E-09	2.91E-07	ATTPS10	trehalose phosphate synthase
	AT4G3280C	316.3416	2.1739229	2.3796741	0.3865266	5.6242512	1.86E-08	1.42E-06	NA	NA
	AT4G0221C	36.008469	2.1729444	2.693352	0.538161	4.0377216	5.40E-05	0.0008597	NA	NA
	AT4G2177C	31.783715	2.1714875	2.6393964	0.5205541	4.1714924	3.03E-05	0.0005478	NA	NA
	AT1G1347C	537.45464	2.1620129	2.3190592	0.3458098	6.2520294	4.05E-10	5.66E-08	NA	NA
	AT1G5829C	3836.0589	2.1475659	2.3826462	0.4103841	5.2330632	1.67E-07	8.92E-06	AtHEMA1	Arabidopsis thaliana hemA 1
	AT3G5993C	187.19126	2.1472688	3.2809784	0.6494883	3.3060932	0.0009461	0.007601	NA	NA
	AT1G5372C	47.388448	2.1457411	2.4520691	0.4530491	4.7362225	2.18E-06	6.63E-05	ATCYP59	CYCLOPHILIN 59
	AT5G3864C	12.225114	2.1408778	3.2643624	0.6441444	3.323599	0.0008886	0.0072679	NA	NA
	AT1G1242C	76.89611	2.1368734	2.4410648	0.4524541	4.7228509	2.33E-06	6.98E-05	ACR8	ACT domain repeat 8
	AT4G1333C	28.971488	2.1227885	2.7376309	0.5701754	3.7230445	0.0001968	0.0023414	NA	NA
	AT1G7893C	16.209419	2.1209596	2.9623285	0.6126261	3.4620782	0.000536	0.0049664	NA	NA
	AT5G1076C	1183.084	2.1209186	2.3028809	0.3712721	5.7125717	1.11E-08	9.27E-07	NA	NA
	AT1G6554C	105.67891	2.1192679	2.3296122	0.393756	5.3821853	7.36E-08	4.46E-06	AtLETM2	0
	AT3G5911C	167.90489	2.1183965	2.361804	0.4178104	5.0702341	3.97E-07	1.79E-05	NA	NA
	AT4G2184C	140.6872	2.1100474	2.269225	0.3510605	6.0104954	1.85E-09	2.00E-07	ATMSRB8	methionine sulfoxide reductase B8
	AT4G0454C	15.592182	2.1065679	2.8074064	0.5860137	3.594742	0.0003247	0.0033982	CRK39	cysteine-rich RLK (RECEPTOR-like protein kinase) 39
YES	AT1G6805C	68.853973	2.1030968	2.3494096	0.4201311	5.0058105	5.56E-07	2.32E-05	ADO3	0
	AT5G6371C	148.02614	2.1030085	2.3377812	0.412969	5.092413	3.54E-07	1.63E-05	NA	NA
	AT2G2640C	230.02327	2.0891138	2.3571308	0.4361728	4.7896477	1.67E-06	5.41E-05	ARD	ACIREDUCTONE DIOXYGENASE
	AT4G3020C	414.19422	2.0879222	2.3131869	0.4077666	5.1203861	3.05E-07	1.46E-05	VEL1	vernalization5/VIN3-like 1
YES	AT3G0635C	259.46288	2.0767063	2.2616911	0.3769391	5.5093946	3.60E-08	2.53E-06	EMB3004	EMBRYO DEFECTIVE 3004
	AT2G3335C	9.0044554	2.0673841	3.2373649	0.6492263	3.184381	0.0014506	0.010411	NA	NA
	AT3G6094C	13.566296	2.0626385	2.7976352	0.5970343	3.4548072	0.0005507	0.0050762	NA	NA
	AT2G1748C	167.53331	2.0589521	2.4172843	0.4880403	4.2188158	2.46E-05	0.0004664	ATMLO8	MILDEW RESISTANCE LOCUS O 8
	AT2G3650C	15.742528	2.0569	2.6839791	0.5736404	3.5856961	0.0003362	0.0034859	NA	NA
	AT4G0437C	12.973337	2.0524592	3.0116251	0.6305379	3.2550928	0.0011336	0.0087513	NA	NA

Supplemental Table 3-RNA seq data comparing *Pro35S::MYB63 Pro35S::LAC17* with wild type.

DAP.MYB63.target(1.5kb upstream TSS)	Gene	baseMean	log2FoldChange	lfcMLE	lfcSE	stat	pvalue	padj (FDR)	TAIR10 Symbol	TAIR10 Annotation (Short)
YES	AT5G22630	1628.9412	2.0424729	2.145923	0.2949482	6.9248538	4.36E-12	9.25E-10	ADT5	arogenate dehydratase 5
	AT1G70980	116.89207	2.0356606	2.2549272	0.4074311	4.9963313	5.84E-07	2.42E-05	SYNC3	0
YES	AT3G11490	117.7762	2.0325051	2.2679053	0.4189338	4.8516142	1.22E-06	4.30E-05	NA	NA
	AT4G28630	104.18366	2.0300846	2.1965361	0.3636726	5.5821759	2.38E-08	1.76E-06	ABCB23	ATP-binding cassette B23
	AT4G13890	7.50735	2.0285641	3.7187345	0.6762799	2.9995926	0.0027034	0.0166047	EDA36	EMBRYO SAC DEVELOPMENT ARREST 37
	AT4G35110	103.35981	2.0172797	2.214802	0.3912668	5.1557644	2.53E-07	1.26E-05	NA	NA
	AT1G75170	82.244416	2.0157989	2.2413466	0.4126081	4.8855053	1.03E-06	3.79E-05	NA	NA
	AT5G57250	7.3598947	2.0157731	3.7850308	0.6798695	2.964941	0.0030274	0.0180473	NA	NA
	AT5G14080	56.917241	2.0155371	2.2856853	0.4423097	4.5568459	5.19E-06	0.0001336	NA	NA
	AT1G09970	701.74645	2.0120633	2.196443	0.381655	5.2719421	1.35E-07	7.53E-06	LRR XI-23	0
	AT3G03970	23.028671	2.0120358	2.4781275	0.5324513	3.7788163	0.0001576	0.0019665	NA	NA
	AT4G13980	69.477892	2.0093408	2.3010126	0.4560268	4.4061898	1.05E-05	0.0002388	AT-HSFA5	0
	AT2G46720	20.765567	2.0088333	2.6401537	0.5816698	3.453563	0.0005532	0.0050918	HIC	HIGH CARBON DIOXIDE
	AT5G47440	9.2322887	2.0023731	3.3518936	0.6662603	3.0053915	0.0026524	0.0163638	NA	NA
	AT1G65240	20.602165	1.9996241	2.5243462	0.5535189	3.6125671	0.0003032	0.0032277	NA	NA
	AT5G05750	423.25514	1.9976339	2.1627226	0.365411	5.4668134	4.58E-08	3.08E-06	NA	NA
	AT1G25320	71.572556	1.9931148	2.2270037	0.4203236	4.7418576	2.12E-06	6.49E-05	NA	NA
	AT1G61410	12.633199	1.9910736	2.8305428	0.6195792	3.2135902	0.0013109	0.0097175	NA	NA
	AT3G02140	34.792857	1.9860178	2.3227609	0.4811965	4.1272486	3.67E-05	0.0006337	AFP4	ABI FIVE BINDING PROTEIN 4
	AT4G17150	167.37164	1.9849283	2.1858502	0.3975121	4.9933781	5.93E-07	2.45E-05	NA	NA
	AT3G54850	126.08484	1.9825296	2.1499222	0.3684701	5.380436	7.43E-08	4.48E-06	ATPUB14	0
	AT4G35600	162.44364	1.9814012	2.1869671	0.4010193	4.9409126	7.78E-07	3.06E-05	CST	CAST AWAY
	AT2G38250	10.366133	1.9734682	2.960669	0.6396212	3.0853701	0.002033	0.0134151	NA	NA
	AT1G74030	95.909964	1.9708764	2.1663354	0.3933052	5.0110607	5.41E-07	2.29E-05	ENO1	enolase 1
	AT3G03640	305.26966	1.9667496	2.0969505	0.3320257	5.923487	3.15E-09	3.17E-07	BGLU25	beta glucosidase 25
	AT5G67480	978.02685	1.9639355	2.1776689	0.4094703	4.796283	1.62E-06	5.31E-05	ATBT4	0
	AT1G69060	53.069894	1.9620077	2.20199	0.4269562	4.5953369	4.32E-06	0.0001157	NA	NA
	AT3G48440	34.872803	1.956485	2.3520643	0.512499	3.8175392	0.0001348	0.0017576	NA	NA
	AT2G16250	22.053564	1.9542508	2.4638592	0.5531743	3.5327937	0.0004112	0.0040492	NA	NA
	AT4G03610	17.659518	1.9510838	2.5233516	0.5698197	3.4240373	0.000617	0.005538	NA	NA
	AT2G45800	46.943243	1.9503866	2.2926614	0.4887768	3.9903424	6.60E-05	0.0010008	PLIM2a	PLIM2a
	AT5G02860	41.322381	1.9470878	2.3461668	0.5148714	3.7816973	0.0001558	0.001952	NA	NA
	AT3G52030	45.489534	1.9445979	2.2448806	0.4671147	4.162999	3.14E-05	0.0005605	NA	NA
	AT1G26580	266.8427	1.943178	2.1049403	0.3665379	5.3014377	1.15E-07	6.52E-06	NA	NA
	AT1G14140	90.574217	1.9430643	2.1187067	0.3788665	5.1286247	2.92E-07	1.41E-05	UCP3	Uncoupling Protein 3
	AT5G64960	30.201762	1.9413827	2.4049629	0.5384822	3.6052865	0.0003118	0.0032937	CDKC2	cyclin dependent kinase group C2
	AT2G02810	169.86424	1.9336314	2.1432665	0.4085443	4.7329787	2.21E-06	6.71E-05	ATUTR1	UDP-GALACTOSE TRANSPORTER 1
	AT4G24290	320.99134	1.9297842	2.0575695	0.3321622	5.8097645	6.26E-09	5.84E-07	NA	NA
	AT2G26760	24.276638	1.9276384	2.317266	0.5100476	3.7793303	0.0001573	0.0019638	CYCB1;4	Cyclin B1;4
	AT5G36001	25.717489	1.9259197	2.4772252	0.5684045	3.3882906	0.0007033	0.0060875	NA	NA
	AT5G18500	502.50552	1.9244978	2.0537745	0.3343264	5.7563444	8.60E-09	7.56E-07	NA	NA
	AT3G55500	121.26125	1.9216759	2.0966349	0.3799978	5.0570707	4.26E-07	1.88E-05	ATEXP16	0
	AT5G24060	138.1491	1.9212632	2.1074445	0.3900483	4.9257057	8.41E-07	3.24E-05	NA	NA
	AT5G55930	68.040098	1.9190806	2.1431832	0.4199663	4.5696059	4.89E-06	0.000128	ATOPT1	ARABIDOPSIS THALIANA OLIGOPEPTIDE TRANSPORTER 1
	AT3G57260	1270.4218	1.9125395	2.0411559	0.3345309	5.7170786	1.08E-08	9.12E-07	AtBG2	0
	AT5G14510	16.411343	1.9113923	2.6368445	0.6076361	3.1456198	0.0016574	0.011554	NA	NA
	AT1G04200	142.93034	1.9113087	2.0826525	0.3778965	5.057757	4.24E-07	1.88E-05	NA	NA
	AT1G80150	18.048256	1.9109626	2.4469493	0.5632194	3.3929275	0.0006915	0.0060187	NA	NA

Supplemental Table 3-RNA seq data comparing *Pro35S::MYB63 Pro35S::LAC17* with wild type.

DAP.MYB63.target(1.5kb upstream TSS)	Gene	baseMean	log2FoldChange	lfcMLE	lfcSE	stat	pvalue	padj (FDR)	TAIR10 Symbol	TAIR10 Annotation (Short)
	AT3G05660	39.851437	1.9105508	2.1847591	0.454202	4.2063898	2.59E-05	0.0004841	AtRLP33	receptor like protein 33
	AT2G30470	37.115928	1.9013065	2.1959317	0.4665412	4.0753238	4.60E-05	0.0007573	HSI2	high-level expression of sugar-inducible gene 2
	AT1G27752	92.81454	1.9001209	2.0654225	0.3728338	5.0964293	3.46E-07	1.60E-05	NA	NA
	AT1G03660	22.622423	1.8969045	2.384285	0.550339	3.4467928	0.0005673	0.0051779	NA	NA
	AT2G01350	166.91944	1.8912322	2.0685609	0.3850268	4.9119499	9.02E-07	3.41E-05	QPT	quinolinate phoshoribosyltransferase
	AT1G76270	40.060895	1.8895659	2.1521115	0.4487934	4.210325	2.55E-05	0.0004782	NA	NA
	AT5G26742	3118.0055	1.8802414	1.9991381	0.3256905	5.7730919	7.78E-09	7.04E-07	AtRH3	0
	AT1G12120	429.06466	1.8783751	2.0276884	0.3594641	5.2254877	1.74E-07	9.21E-06	NA	NA
	AT1G71350	41.081911	1.8782405	2.1570652	0.4600932	4.0823045	4.46E-05	0.0007393	NA	NA
	AT2G02350	9.9298379	1.8766375	2.9109777	0.650887	2.8832003	0.0039366	0.0218549	AtPP2-B9	Phloem protein 2-B9
	AT2G46310	38.270598	1.8720782	2.2518259	0.512637	3.6518592	0.0002603	0.0028819	CRF5	cytokinin response factor 5
	AT5G18475	15.573023	1.8685243	2.4303813	0.573872	3.2559949	0.00113	0.008732	NA	NA
	AT3G13770	5.4183242	1.8595234	3.8448879	0.6858697	2.7111905	0.0067042	0.0325349	NA	NA
	AT4G04460	7.0425237	1.8578189	3.1508357	0.6672002	2.7844999	0.005361	0.0274932	NA	NA
	AT2G24645	55.65755	1.8560383	2.0977775	0.4382644	4.234974	2.29E-05	0.0004382	NA	NA
	AT3G57270	8.8913697	1.8559548	2.975413	0.6587504	2.817387	0.0048416	0.0255053	BG1	beta-1,3-glucanase 1
	AT2G01100	115.87222	1.8546313	2.0187825	0.3756524	4.937095	7.93E-07	3.11E-05	NA	NA
YES	AT1G14170	168.71831	1.851605	2.0293718	0.3887453	4.7630284	1.91E-06	6.01E-05	NA	NA
	AT1G04980	103.37499	1.8498725	2.0125473	0.3745173	4.9393518	7.84E-07	3.08E-05	ATPDI10	ARABIDOPSIS THALIANA PROTEIN DISULFIDE ISOMERASE 10
	AT4G21065	6.7506461	1.8454542	3.1437893	0.6688	2.7593515	0.0057916	0.0291702	NA	NA
	AT5G07620	29.337439	1.8435898	2.1674768	0.4880325	3.7775961	0.0001583	0.0019735	NA	NA
	AT1G31010	46.462763	1.8427976	2.0891017	0.4423005	4.1663923	3.09E-05	0.000556	OSB4	organellar single-stranded DNA binding protein 4
	AT5G61480	40.987422	1.8409943	2.1164108	0.461562	3.9886177	6.65E-05	0.0010064	PXY	PHLOEM INTERCALATED WITH XYLEM
	AT1G06390	346.3796	1.838588	2.009888	0.3842873	4.7844106	1.71E-06	5.51E-05	ATGSK1	0
	AT3G50040	12.789308	1.8348692	2.5523643	0.6121448	2.9974431	0.0027225	0.0166824	NA	NA
YES	AT2G36880	1498.1269	1.8296571	1.9317612	0.3080959	5.9385964	2.87E-09	2.91E-07	MAT3	methionine adenosyltransferase 3
	AT2G19450	104.45795	1.8290032	1.987841	0.3726829	4.9076667	9.22E-07	3.46E-05	ABX45	0
	AT4G17660	12.974863	1.8268713	2.5003155	0.6028077	3.0306038	0.0024407	0.0153787	NA	NA
	AT3G55090	17.380287	1.8249897	2.4607417	0.5961167	3.0614638	0.0022026	0.0142421	ABCG16	ATP-binding cassette G16
	AT1G47056	111.14033	1.8248593	2.0001792	0.3885536	4.6965448	2.65E-06	7.72E-05	VFB1	VIER F-box proteine 1
	AT1G78050	26.045683	1.8236831	2.2319951	0.5282595	3.4522488	0.0005559	0.0051063	PGM	phosphoglycerate/bisphosphoglycerate mutase
	AT1G11940	31.240764	1.8235467	2.3661073	0.5748323	3.1723108	0.0015123	0.0107636	NA	NA
	AT5G13830	5.628752	1.8234126	3.8785072	0.687169	2.6535142	0.0079658	0.0370362	NA	NA
	AT3G57550	437.5791	1.8229602	1.9122365	0.2904843	6.2755896	3.48E-10	4.94E-08	AGK2	guanylate kinase
	AT5G52010	57.432327	1.8218807	2.0801107	0.4524905	4.0263397	5.67E-05	0.000891	NA	NA
	AT1G47840	6.7613015	1.8207538	3.1779792	0.6733106	2.7041812	0.0068473	0.033008	HXK3	hexokinase 3
	AT5G24490	3506.421	1.8176203	1.9124811	0.2990144	6.0787051	1.21E-09	1.42E-07	NA	NA
	AT5G64580	262.14155	1.8151673	1.9485348	0.3475007	5.2234922	1.76E-07	9.28E-06	EMB3144	EMBRYO DEFECTIVE 3144
	AT2G40520	192.63838	1.8072883	1.940712	0.348012	5.1931784	2.07E-07	1.07E-05	NA	NA
	AT3G54090	337.08082	1.8051063	1.9177313	0.3235814	5.5785239	2.43E-08	1.79E-06	FLN1	fructokinase-like 1
	AT5G61380	226.42748	1.8013073	1.9204134	0.3319794	5.425961	5.76E-08	3.70E-06	APRR1	0
	AT2G44040	238.33224	1.8010613	1.95774	0.373247	4.8253873	1.40E-06	4.79E-05	NA	NA
	AT1G19540	36.868668	1.7956883	2.1240561	0.4955229	3.6238253	0.0002903	0.0031249	NA	NA

Supplemental Table 3-RNA seq data comparing *Pro35S::MYB63 Pro35S::LAC17* with wild type.

DAP.MYB63.target(1.5kb upstream TSS)	Gene	baseMean	log2FoldChange	lfcMLE	lfcSE	stat	pvalue	padj (FDR)	TAIR10 Symbol	TAIR10 Annotation (Short)
	AT1G60700	14.017267	1.7951808	2.434693	0.6003025	2.9904603	0.0027856	0.0169591	NA	NA
	AT2G42580	1116.9713	1.7931517	1.8988327	0.3157133	5.6796841	1.35E-08	1.10E-06	TTL3	tetratricopetide-repeat thioredoxin-like 3
	AT1G01680	111.37249	1.7899039	2.0050892	0.4251124	4.2104253	2.55E-05	0.0004782	ATPUB54	PLANT U-BOX 54
	AT3G60300	231.91537	1.789216	1.9436602	0.3721777	4.807424	1.53E-06	5.08E-05	NA	NA
	AT4G00231	114.06019	1.7887879	1.9282492	0.3563189	5.0201881	5.16E-07	2.20E-05	MEE50	maternal effect embryo arrest 50
	AT3G07730	10.348339	1.788113	2.6908039	0.642046	2.785023	0.0053524	0.0274692	NA	NA
	AT5G38730	16.729745	1.7871632	2.3111288	0.5706545	3.1317777	0.0017375	0.0120014	NA	NA
	AT5G40010	8.845927	1.786429	2.8693289	0.6586004	2.7124627	0.0066785	0.0324636	AATP1	AAA-ATPase 1
	AT4G36940	184.83328	1.7851292	1.9324018	0.3650339	4.8903111	1.01E-06	3.72E-05	NAPRT1	nicotinate phosphoribosyltransferase 1
YES	AT5G66120	1159.1804	1.7846654	1.8762478	0.2967069	6.0149107	1.80E-09	1.95E-07	NA	NA
	AT3G15470	103.95804	1.7835727	1.937703	0.372036	4.7940857	1.63E-06	5.35E-05	NA	NA
	AT3G13380	51.253933	1.7814061	2.0573875	0.4672227	3.8127563	0.0001374	0.0017817	BRL3	BRI1-like 3
YES	AT3G05290	338.70624	1.7812529	1.8984732	0.3313667	5.3754727	7.64E-08	4.57E-06	AtPNC1	0
	AT2G43570	1103.6646	1.7804593	1.9697534	0.4056884	4.3887362	1.14E-05	0.0002543	CHI	chitinase, putative
	AT5G59490	8.3101855	1.7776943	2.8472059	0.6588868	2.6980267	0.0069752	0.0334486	NA	NA
	AT2G28800	3866.1632	1.7774727	1.9300354	0.3714989	4.7845969	1.71E-06	5.51E-05	ALB3	ALBINO 3
	AT4G17160	10.159211	1.7772179	2.8892292	0.662513	2.6825405	0.0073065	0.0347509	ATRAB2B	0
	AT3G61570	5.5145224	1.7764701	3.2897049	0.6797029	2.6135981	0.0089594	0.040294	GC3	GOLGIN CANDIDATE 3
	AT5G04780	9.7068915	1.7764453	2.6413239	0.6388383	2.7807431	0.0054235	0.0277345	NA	NA
	AT4G15110	498.79349	1.7755708	1.886555	0.3239939	5.4802597	4.25E-08	2.88E-06	CYP97B3	cytochrome P450, family 97, subfamily B, polypeptide 3
	AT5G23810	23.586878	1.7752178	2.3648926	0.5920172	2.9985917	0.0027123	0.0166366	AAP7	amino acid permease 7
	AT3G49350	141.96506	1.772079	1.9118824	0.3579197	4.9510518	7.38E-07	2.92E-05	NA	NA
	AT1G57560	32.969855	1.7703709	2.2090353	0.5458027	3.2436098	0.0011803	0.0090373	AtMYB50	myb domain protein 50
	AT5G14620	72.095938	1.7635385	1.9309986	0.3865913	4.561764	5.07E-06	0.0001316	DMT7	0
	AT5G23240	129.56632	1.7600001	1.9567752	0.4138173	4.2530849	2.11E-05	0.0004108	DJC76	DNA J protein C76
	AT1G04900	24.525481	1.7592414	2.1546202	0.5295366	3.3222284	0.000893	0.0072883	NA	NA
	AT1G64600	11.654488	1.7571834	2.5275807	0.6267735	2.8035381	0.0050545	0.0263564	NA	NA
	AT5G60280	31.666743	1.7566348	2.0686045	0.4901306	3.5840134	0.0003384	0.0034983	LecRK-I.8	L-type lectin receptor kinase I.8
	AT5G05470	30.525805	1.7554043	2.0790068	0.4959542	3.5394487	0.000401	0.0039702	ATEIF2-A2	0
	AT4G04490	51.474603	1.7548301	2.0171714	0.4613591	3.8036098	0.0001426	0.0018278	CRK36	cysteine-rich RLK (RECEPTOR-like protein kinase) 36
	AT4G39030	95.304828	1.7525379	1.9090383	0.3770202	4.648393	3.35E-06	9.34E-05	EDS5	ENHANCED DISEASE SUSCEPTIBILITY 5
	AT2G22270	38.505712	1.7519268	2.0164506	0.4629153	3.7845512	0.000154	0.0019365	NA	NA
	AT3G07360	323.45933	1.7512722	1.87842	0.3458052	5.0643315	4.10E-07	1.84E-05	ATPUB9	ARABIDOPSIS THALIANA PLANT U-BOX 9
YES	AT1G51680	638.85214	1.7481746	1.851571	0.3161642	5.5293246	3.21E-08	2.29E-06	4CL.1	4-COUMARATE:COA LIGASE 1
	AT2G29990	91.65623	1.7466378	1.9005285	0.3750907	4.6565747	3.22E-06	9.10E-05	NDA2	alternative NAD(P)H dehydrogenase 2
	AT1G25480	59.352559	1.7462521	1.9671001	0.4335998	4.0273357	5.64E-05	0.0008888	NA	NA
	AT1G76970	159.6804	1.7439688	1.8860864	0.3632415	4.8011278	1.58E-06	5.22E-05	NA	NA
	AT4G29560	7.6246844	1.7430221	3.2638732	0.681551	2.5574345	0.0105447	0.0456356	NA	NA
	AT5G08305	6.1054285	1.7410345	3.3727499	0.6835263	2.547136	0.0108611	0.0465503	NA	NA
	AT5G52630	9.868118	1.7410101	2.7872524	0.6588415	2.6425325	0.0082289	0.0378586	MEF1	mitochondrial RNA editing factor 1
	AT5G22840	20.103781	1.7401197	2.2184315	0.5612341	3.100524	0.0019318	0.0129319	NA	NA
	AT3G13600	6.7360769	1.7397668	3.1174761	0.6770522	2.5696201	0.010181	0.0444135	NA	NA

Supplemental Table 3-RNA seq data comparing *Pro35S::MYB63 Pro35S::LAC17* with wild type.

DAP.MYB63.target(1.5kb upstream TSS)	Gene	baseMean	log2FoldChange	lfcMLE	lfcSE	stat	pvalue	padj (FDR)	TAIR10 Symbol	TAIR10 Annotation (Short)
	AT5G5355C	798.587	1.7382827	1.8327229	0.304448	5.7096213	1.13E-08	9.36E-07	ATYSL3	YELLOW STRIPE LIKE 3
	AT3G4974C	5.9408601	1.7372865	3.4566893	0.6858805	2.5329288	0.0113114	0.0479424	NA	NA
	AT5G5528C	576.86572	1.7365697	1.8429338	0.3210666	5.4087528	6.35E-08	3.99E-06	ATFTSZ1-1	ARABIDOPSIS THALIANA HOMOLOG OF BACTERIAL CYTOKINESIS Z-RING PROTEIN FTSZ 1-1
	AT3G4784C	6.4400428	1.7359307	3.0688199	0.6754669	2.5699714	0.0101707	0.0443792	NA	NA
	AT5G4626C	28.130518	1.735672	2.1615418	0.5447841	3.1859812	0.0014426	0.0103825	NA	NA
	AT4G1364C	195.41386	1.7332292	1.8592378	0.3460214	5.0090237	5.47E-07	2.30E-05	UNE16	unfertilized embryo sac 16
	AT1G4729C	135.66494	1.7325112	1.8725116	0.3618159	4.788378	1.68E-06	5.43E-05	3BETAHSD/	3beta-hydroxysteroid-dehydrogenase/decarboxylase isoform 1
	AT4G3518C	35.830346	1.7324278	2.0247477	0.481314	3.5993717	0.000319	0.0033485	LHT7	LYS/HIS transporter 7
	AT3G1319C	22.046967	1.7315873	2.1732225	0.5500956	3.1477931	0.0016451	0.0114907	NA	NA
	AT5G0989C	194.27383	1.7304385	1.8568939	0.3466984	4.991192	6.00E-07	2.46E-05	NA	NA
	AT4G3279C	224.41682	1.7293509	1.8421364	0.3299261	5.2416308	1.59E-07	8.56E-06	NA	NA
	AT5G0771C	73.486681	1.7289774	1.8869858	0.3806124	4.5426193	5.56E-06	0.0001414	NA	NA
	AT4G3591C	88.79364	1.7285466	1.9066281	0.4002847	4.3182933	1.57E-05	0.0003261	NA	NA
	AT4G3124C	39.881234	1.7273957	2.0441144	0.4957904	3.484125	0.0004937	0.0046657	AtNRX2	0
	AT4G2453C	63.619361	1.7262347	1.9399216	0.4301168	4.0134091	5.98E-05	0.0009234	NA	NA
	AT4G2154C	146.73131	1.725471	1.8852261	0.3832622	4.5020639	6.73E-06	0.0001659	SPHK1	sphingosine kinase 1
	AT2G3332C	73.955296	1.7245889	1.9113545	0.4079331	4.2276271	2.36E-05	0.0004499	NA	NA
YES	AT5G1799C	475.2853	1.7243566	1.8236317	0.3123703	5.5202329	3.39E-08	2.39E-06	pat1	PHOSPHORIBOSYLANTHRANILATE TRANSFERASE 1
	AT2G3454C	7.6078984	1.724166	2.6737306	0.650463	2.6506749	0.0080331	0.0372179	NA	NA
	AT2G1702C	73.169667	1.7241199	1.9472896	0.4380522	3.9358778	8.29E-05	0.0012037	NA	NA
	AT4G21534	82.730821	1.7239497	1.8797869	0.3791986	4.5462982	5.46E-06	0.0001396	SPHK2	sphingosine kinase 2
	AT1G1364C	62.611906	1.7236462	1.931269	0.4257452	4.0485391	5.15E-05	0.0008294	NA	NA
	AT5G4509C	8.4296437	1.7235735	2.8708586	0.6676068	2.5817194	0.0098309	0.0432826	AtPP2-A7	phloem protein 2-A7
	AT4G1065C	12.813865	1.7198727	2.3282982	0.5978575	2.8767267	0.0040182	0.0221717	NA	NA
	AT5G6652C	41.514643	1.7191139	2.1453216	0.5469774	3.1429339	0.0016726	0.0116381	NA	NA
	AT4G0050C	64.485138	1.7135893	1.8980944	0.4072814	4.2073845	2.58E-05	0.0004825	NA	NA
	AT3G1562C	25.857584	1.7129561	2.0520095	0.5084116	3.369231	0.0007538	0.0064185	UVR3	UV REPAIR DEFECTIVE 3
	AT4G3792C	156.00669	1.7113699	1.9019038	0.4129329	4.1444266	3.41E-05	0.0005963	NA	NA
	AT2G0202C	65.336582	1.7112037	2.0609536	0.5153166	3.3206844	0.000898	0.007319	AtNPF8.4	0
	AT5G1847C	236.77249	1.71101	1.833605	0.3436549	4.9788608	6.40E-07	2.58E-05	NA	NA
	AT5G0262C	107.67261	1.710754	1.9221434	0.4300322	3.9781999	6.94E-05	0.0010448	ANK1	ankyrin-like1
	AT5G5438C	382.71364	1.7103922	1.8988913	0.4114896	4.1565868	3.23E-05	0.0005735	THE1	THESEUS1
	AT2G3280C	387.11135	1.706762	1.8243533	0.3380328	5.0491011	4.44E-07	1.94E-05	AP4.3A	0
	AT2G3936C	150.06678	1.7057715	1.8316786	0.3481324	4.8997778	9.59E-07	3.57E-05	NA	NA
	AT1G2768C	61.190663	1.705609	1.9555	0.4582329	3.7221445	0.0001975	0.0023482	APL2	ADPGLC-PPase large subunit
	AT3G5677C	28.807325	1.7050535	2.0080142	0.4901085	3.4789304	0.0005034	0.0047473	NA	NA
	AT5G6143C	10.756265	1.7044787	2.4171964	0.6214726	2.7426448	0.0060947	0.0301736	ANAC100	NAC domain containing protein 100
	AT2G3060C	613.4302	1.7017053	1.873243	0.3971004	4.2853277	1.82E-05	0.0003658	NA	NA
	AT2G3363C	292.47417	1.7010938	1.8469075	0.3711148	4.5837399	4.57E-06	0.0001209	NA	NA
	AT5G2333C	49.244275	1.6998739	1.9706521	0.4729919	3.5938756	0.0003258	0.0034075	NA	NA
	AT5G5115C	237.78924	1.6995568	1.813008	0.3334801	5.0964261	3.46E-07	1.60E-05	NA	NA
	AT2G4441C	294.18976	1.6984623	1.8457916	0.3730269	4.55319	5.28E-06	0.0001354	NA	NA
	AT2G1980C	88.060064	1.6983271	1.983167	0.4822722	3.5215117	0.0004291	0.0041753	MIOX2	myo-inositol oxygenase 2

Supplemental Table 3-RNA seq data comparing *Pro35S::MYB63 Pro35S::LAC17* with wild type.

DAP.MYB63.target(1.5kb upstream TSS)	Gene	baseMean	log2FoldChange	lfcMLE	lfcSE	stat	pvalue	padj (FDR)	TAIR10 Symbol	TAIR10 Annotation (Short)
	AT4G1458C	98.027359	1.6974767	1.8684328	0.3966412	4.279628	1.87E-05	0.0003715	CIPK4	CBL-interacting protein kinase 4
	AT2G02955	17.471993	1.6967815	2.1200126	0.5462293	3.1063541	0.0018941	0.0127459	MEE12	maternal effect embryo arrest 12
	AT1G6476C	71.852123	1.6965829	1.9029539	0.427412	3.9694326	7.20E-05	0.0010774	NA	NA
	AT4G2046C	9.9386531	1.6937073	2.4689637	0.6324742	2.6779074	0.0074084	0.0350963	NA	NA
	AT5G0598C	79.354171	1.6927463	1.8731658	0.4058131	4.171246	3.03E-05	0.0005478	ATDFB	DHFS-FPGS homolog B
	AT5G6010C	16.567285	1.689141	2.2829205	0.6001013	2.81476	0.0048814	0.0256539	APRR3	pseudo-response regulator 3
	AT1G6197C	22.122576	1.6855428	2.0270702	0.5119323	3.2925109	0.000993	0.0078864	NA	NA
	AT1G7228C	118.69497	1.6844172	1.8101776	0.3498421	4.8147928	1.47E-06	4.96E-05	AERO1	endoplasmic reticulum oxidoreductins 1
	AT4G2839C	105.52905	1.6844114	1.8307783	0.3727903	4.5183883	6.23E-06	0.0001555	AAC3	ADP/ATP carrier 3
	AT5G1895C	24.34479	1.6833697	2.1904262	0.576922	2.917846	0.0035246	0.0202137	NA	NA
	AT5G1501C	11.066467	1.681786	2.4394539	0.6306469	2.666763	0.0076586	0.0359601	NA	NA
	AT3G0495C	25.608638	1.681685	2.184069	0.5762432	2.91836	0.0035188	0.0202013	NA	NA
YES	AT2G4089C	619.68632	1.6816004	1.7976578	0.3383381	4.9701777	6.69E-07	2.68E-05	CYP98A3	cytochrome P450, family 98, subfamily A, polypeptide 3
	AT1G3434C	116.56828	1.6803569	1.8925914	0.4341007	3.8708921	0.0001084	0.0014805	NA	NA
	AT1G7813C	32.511048	1.6789541	2.0295896	0.5181926	3.2400192	0.0011952	0.0091126	UNE2	unfertilized embryo sac 2
	AT2G1800C	9.8314553	1.6785341	2.709504	0.6629755	2.5318192	0.0113472	0.0480811	TAF14	TBP-associated factor 14
	AT2G3032C	50.600058	1.6776547	1.9228653	0.4580081	3.6629369	0.0002493	0.0027926	NA	NA
	AT3G2074C	31.696121	1.6774517	1.9465754	0.4733936	3.5434609	0.0003949	0.0039259	FIE	FERTILIZATION-INDEPENDENT ENDOSPERM
	AT3G03305	74.158317	1.6772709	2.006307	0.5084066	3.2990739	0.00097	0.0077675	NA	NA
	AT3G5668C	989.20501	1.6753456	1.7954661	0.3440532	4.8694377	1.12E-06	4.04E-05	NA	NA
	AT1G7537C	347.08365	1.6752677	1.7778755	0.3210864	5.2174982	1.81E-07	9.53E-06	NA	NA
	AT5G0491C	125.08486	1.6732592	1.8941676	0.4415306	3.7896784	0.0001508	0.001905	NA	NA
	AT1G7250C	173.19737	1.6726357	1.7927706	0.3440999	4.8609012	1.17E-06	4.19E-05	NA	NA
	AT1G5583C	66.84741	1.6718069	1.8640934	0.4181057	3.9985273	6.37E-05	0.0009742	NA	NA
	AT1G2805C	75.606145	1.6703234	1.8220091	0.3799072	4.3966615	1.10E-05	0.0002474	BBX13	B-box domain protein 13
	AT2G1887€	34.335594	1.6686411	2.052279	0.5341813	3.1237354	0.0017857	0.0122173	NA	NA
	AT1G6785C	76.630302	1.6681311	1.9084523	0.455578	3.6615708	0.0002507	0.0028019	NA	NA
	AT5G3520C	144.75257	1.6680352	1.8250216	0.3856778	4.3249454	1.53E-05	0.0003197	NA	NA
	AT4G3049C	349.31988	1.6653667	1.7905579	0.3510336	4.7441806	2.09E-06	6.43E-05	NA	NA
	AT5G5550C	115.526	1.6640662	1.8068165	0.3711031	4.4841078	7.32E-06	0.0001775	ATXYLT	ARABIDOPSIS THALIANA BETA-1,2-XYLOSYLTRANSFERASE
	AT4G1275C	11.219992	1.6630077	2.3467077	0.6201466	2.6816361	0.0073263	0.0348357	NA	NA
	AT4G3848C	31.763495	1.6617383	1.9292151	0.4736014	3.5087278	0.0004503	0.0043437	NA	NA
	AT5G6019C	49.573388	1.6615976	1.9126083	0.4638721	3.5820166	0.000341	0.003513	NA	NA
	AT1G3090C	94.318887	1.6592434	1.8259635	0.3961427	4.1884996	2.81E-05	0.0005148	BP80-3;3	binding protein of 80 kDa 3;3
	AT5G2544C	506.0214	1.6582076	1.7412972	0.2935211	5.6493649	1.61E-08	1.28E-06	NA	NA
	AT1G1292C	472.58143	1.6579415	1.778545	0.3461002	4.7903504	1.66E-06	5.41E-05	ERF1-2	eukaryotic release factor 1-2
	AT5G6475C	14.181019	1.6579266	2.3882177	0.6285016	2.6379038	0.008342	0.0382134	ABR1	ABA REPRESSOR1
	AT5G6424C	489.93333	1.657178	1.7592433	0.3218934	5.1482205	2.63E-07	1.30E-05	AtMC3	metacaspase 3
	AT3G5862C	306.63925	1.6568548	1.7666196	0.332392	4.9846413	6.21E-07	2.53E-05	TTL4	tetratricopetide-repeat thioredoxin-like 4
	AT4G1186C	140.09291	1.6538749	1.7719837	0.3431899	4.8191243	1.44E-06	4.88E-05	NA	NA
	AT4G3628C	33.905157	1.6535304	1.9071993	0.4655676	3.5516437	0.0003828	0.0038392	CRH1	CRT1 Homologue 1
	AT5G4684C	87.328812	1.653501	1.8661694	0.4366917	3.7864268	0.0001528	0.001926	NA	NA

Supplemental Table 3-RNA seq data comparing *Pro35S::MYB63 Pro35S::LAC17* with wild type.

DAP.MYB63.target(1.5kb upstream TSS)	Gene	baseMean	log2FoldChange	lfcMLE	lfcSE	stat	pvalue	padj (FDR)	TAIR10 Symbol	TAIR10 Annotation (Short)
	AT4G2006C	13.961027	1.6530134	2.2241637	0.5958158	2.77437	0.0055309	0.0281464	EMB1895	EMBRYO DEFECTIVE 1895
	AT5G5714C	12.832915	1.6526652	2.3870522	0.629896	2.6237111	0.0086978	0.0393825	ATPAP28	PURPLE ACID PHOSPHATASE 28
	AT2G2055C	31.3378	1.6524519	2.0240209	0.5301976	3.116672	0.001829	0.0124458	NA	NA
	AT2G21385	350.73496	1.6519651	1.7750532	0.3497179	4.7237078	2.32E-06	6.97E-05	NA	NA
	AT4G3645C	11.099309	1.6515307	2.4916267	0.6452841	2.5593851	0.0104858	0.0454786	ATMPK14	mitogen-activated protein kinase 14
	AT4G33985	127.94281	1.6481028	1.8432431	0.4230456	3.895804	9.79E-05	0.0013662	NA	NA
	AT3G5127C	281.67119	1.64647	1.7893288	0.3730752	4.4132395	1.02E-05	0.0002326	NA	NA
	AT5G5409C	16.783159	1.6444724	2.208687	0.596968	2.754708	0.0058745	0.0294065	NA	NA
	AT5G1776C	65.138365	1.6439437	1.8344269	0.4190893	3.9226575	8.76E-05	0.0012596	NA	NA
	AT5G2253C	42.103159	1.6430143	1.8594017	0.4403709	3.7309786	0.0001907	0.0022915	NA	NA
	AT2G1729C	208.95588	1.6413676	1.7713484	0.3588413	4.5740766	4.78E-06	0.0001258	ATCDPK3	ARABIDOPSIS THALIANA CALMODULIN-DOMAIN PROTEIN KINASE 3
	AT4G0215C	19.901615	1.6396833	2.0917547	0.5632395	2.9111651	0.0036008	0.0205377	ATIMPALPH	IMPORTIN ALPHA 3
YES	AT4G3420C	534.14343	1.6380646	1.739947	0.3232104	5.0681056	4.02E-07	1.81E-05	EDA9	embryo sac development arrest 9
	AT1G78915	221.67345	1.6379175	1.7606396	0.3504385	4.6739088	2.96E-06	8.47E-05	NA	NA
YES	AT1G0943C	966.02012	1.636864	1.7389073	0.3236326	5.0577851	4.24E-07	1.88E-05	ACLA-3	ATP-citrate lyase A-3
	AT2G4476C	83.260088	1.632182	1.8035853	0.4033706	4.0463586	5.20E-05	0.0008348	NA	NA
	AT1G2002C	5980.4196	1.6312751	1.7249336	0.3120876	5.2269786	1.72E-07	9.19E-06	ATLFNR2	LEAF FNR 2
	AT1G0443C	793.0426	1.6303387	1.7410061	0.3359457	4.852983	1.22E-06	4.30E-05	NA	NA
	AT3G5085C	19.235192	1.6298689	2.0364936	0.5471046	2.9790809	0.0028911	0.0174602	NA	NA
	AT5G5741C	45.946864	1.6298076	1.8426737	0.4389946	3.7125911	0.0002051	0.0024087	NA	NA
	AT5G2663C	11.564033	1.6285573	2.2977285	0.6189069	2.6313444	0.0085048	0.0387032	NA	NA
	AT5G4126C	183.83836	1.6270148	1.7520823	0.3543877	4.5910593	4.41E-06	0.0001174	BSK8	brassinosteroid-signaling kinase 8
	AT2G2893C	333.88861	1.6269775	1.733993	0.3312534	4.91158	9.03E-07	3.41E-05	APK1B	protein kinase 1B
	AT3G5313C	268.99511	1.6268282	1.7595699	0.3634683	4.4758457	7.61E-06	0.0001824	CYP97C1	CYTOCHROME P450 97C1
	AT4G3437C	114.3557	1.6246969	1.8099457	0.4170752	3.8954528	9.80E-05	0.0013671	ARI1	ARIADNE 1
	AT3G0349C	172.40594	1.62359	1.8734469	0.4675887	3.4722608	0.0005161	0.0048348	AtPEX19-1	0
	AT2G4336C	496.34814	1.6223065	1.7132616	0.3087049	5.2552022	1.48E-07	8.07E-06	BIO2	BIOTIN AUXOTROPH 2
	AT5G1922C	911.19602	1.6204117	1.7235306	0.3266144	4.9612371	7.00E-07	2.79E-05	ADG2	ADP GLUCOSE PYROPHOSPHORYLASE 2
	AT3G4664C	639.71709	1.6186669	1.6937298	0.2833759	5.7120849	1.12E-08	9.27E-07	LUX	LUX ARRHYTHMO
	AT2G4544C	129.02241	1.6161874	1.7380172	0.3512384	4.601397	4.20E-06	0.0001132	DHDPS2	dihydrodipicolinate synthase 2
	AT3G0391C	21.594946	1.6153278	2.1002025	0.5762106	2.8033637	0.0050573	0.0263599	GDH3	glutamate dehydrogenase 3
	AT5G0514C	274.97969	1.614905	1.7223403	0.3328736	4.8514061	1.23E-06	4.30E-05	NA	NA
	AT1G0451C	235.9478	1.6147211	1.7441474	0.3606818	4.4768574	7.57E-06	0.000182	MAC3A	MOS4-associated complex 3A
	AT2G0403C	312.54668	1.6120447	1.7401971	0.3595143	4.4839519	7.33E-06	0.0001775	AtHsp90.5	HEAT SHOCK PROTEIN 90.5
	AT1G4518C	29.367727	1.6097304	1.9102152	0.4987166	3.2277457	0.0012477	0.0093882	NA	NA
	AT2G3099C	167.09229	1.6088864	1.7890212	0.4140392	3.8858315	0.000102	0.0014083	NA	NA
	AT1G6398C	339.07374	1.6072347	1.8218982	0.4436163	3.6230287	0.0002912	0.0031327	NA	NA
	AT4G2995C	771.77484	1.6065231	1.6890785	0.2968996	5.410997	6.27E-08	3.95E-06	NA	NA
	AT4G3828C	49.015841	1.6063524	1.7836965	0.4107392	3.9108815	9.20E-05	0.0013059	NA	NA
	AT4G3904C	453.23625	1.6061415	1.6893594	0.2979887	5.3899406	7.05E-08	4.31E-06	NA	NA
	AT5G2526C	24.162562	1.6047499	1.9096758	0.5014002	3.2005369	0.0013717	0.0100405	NA	NA
	AT3G2501C	106.30577	1.6020145	1.7983215	0.4285204	3.7384793	0.0001851	0.0022377	AtRLP41	receptor like protein 41
	AT1G4511C	21.630329	1.6007538	2.0868104	0.5786982	2.7661288	0.0056726	0.028683	NA	NA

Supplemental Table 3-RNA seq data comparing *Pro35S::MYB63 Pro35S::LAC17* with wild type.

DAP.MYB63.target(1.5kb upstream TSS)	Gene	baseMean	log2FoldChange	lfcMLE	lfcSE	stat	pvalue	padj (FDR)	TAIR10 Symbol	TAIR10 Annotation (Short)
	AT5G08340	70.450565	1.5971724	1.7414883	0.3790543	4.2135712	2.51E-05	0.0004748	NA	NA
	AT4G09500	94.615324	1.5953644	1.7526198	0.3930519	4.0589158	4.93E-05	0.0008019	NA	NA
	AT3G05500	705.6337	1.5943804	1.6841558	0.3093425	5.1540948	2.55E-07	1.27E-05	NA	NA
	AT3G25560	44.505596	1.5939945	1.8355911	0.4637711	3.4370287	0.0005881	0.0053421	AtNIK2	0
	AT2G30530	177.34967	1.5935213	1.7098497	0.346534	4.5984551	4.26E-06	0.0001147	NA	NA
	AT4G27800	1697.436	1.5932346	1.6859807	0.3140009	5.0739808	3.90E-07	1.77E-05	PPH1	PROTEIN PHOSPHATASE 1
	AT5G03160	191.35033	1.5927271	1.69972	0.3341536	4.7664516	1.87E-06	5.93E-05	ATP58IPK	homolog of mamallian P58IPK
	AT5G50280	112.95045	1.5913644	1.7524432	0.3973439	4.0050056	6.20E-05	0.0009528	EMB1006	embryo defective 1006
	AT3G24490	36.493596	1.588734	1.8895273	0.501625	3.1671745	0.0015393	0.0108992	NA	NA
	AT3G47010	47.649981	1.5877492	1.8057514	0.4474376	3.5485381	0.0003874	0.003874	NA	NA
	AT1G76040	94.53587	1.5850347	1.7647464	0.4155939	3.8139033	0.0001368	0.0017773	CPK29	calcium-dependent protein kinase 29
	AT2G18850	51.696672	1.5841459	1.8183338	0.459903	3.4445217	0.0005721	0.005212	NA	NA
YES	AT2G46550	633.94802	1.5829546	1.7165488	0.3688383	4.2917304	1.77E-05	0.0003578	NA	NA
	AT3G12920	216.58601	1.5793101	1.7050711	0.3596865	4.3907967	1.13E-05	0.0002522	BRG3	BOI-related gene 3
	AT5G60640	856.31863	1.5742112	1.6909424	0.3490819	4.5095758	6.50E-06	0.0001608	ATPDI2	ARABIDOPSIS THALIANA PROTEIN DISULFIDE ISOMERASE 2
	AT3G16180	169.13846	1.5731895	1.6775888	0.3324423	4.7322177	2.22E-06	6.73E-05	NRT1.12	nitrate transporter 1.12
	AT3G30775	248.49244	1.5723791	1.7304561	0.3965335	3.9653121	7.33E-05	0.0010936	AT-POX	0
YES	AT5G14930	225.93012	1.5714369	1.6806383	0.33924	4.6322277	3.62E-06	9.98E-05	SAG101	senescence-associated gene 101
	AT5G35370	25.713363	1.5708458	1.9252065	0.5302805	2.9622922	0.0030536	0.0181433	NA	NA
	AT3G56580	97.642855	1.5706622	1.7412704	0.4086477	3.8435605	0.0001213	0.0016196	AtRZF1	0
	AT1G09700	23.457151	1.5704444	1.9092857	0.5230132	3.0026862	0.0026761	0.016493	DRB1	DSRNA-BINDING PROTEIN 1
	AT5G55460	375.80821	1.5700487	1.6649764	0.3192519	4.9178986	8.75E-07	3.33E-05	NA	NA
	AT3G03310	156.49467	1.5639081	1.6757548	0.3434081	4.5540803	5.26E-06	0.0001351	ATLCAT3	ARABIDOPSIS LECITHIN:CHOLESTEROL ACYLTRANSFERASE 3
	AT1G54350	353.51698	1.5636239	1.6580018	0.3190328	4.9011377	9.53E-07	3.56E-05	ABCD2	ATP-binding cassette D2
	AT1G61680	11.107926	1.5633308	2.1228281	0.6011972	2.6003628	0.0093125	0.0414988	ATTPS14	TERPENE SYNTHASE 14
	AT1G63720	39.126831	1.5610141	1.8180995	0.47794	3.2661302	0.0010903	0.0084763	NA	NA
	AT4G21180	237.97914	1.5603578	1.7114748	0.3905562	3.9952197	6.46E-05	0.0009862	ATERDJ2B	0
	AT5G57830	28.780469	1.559744	1.8996038	0.5254458	2.9684202	0.0029933	0.0178975	NA	NA
	AT5G67460	14.918187	1.5590924	2.0717159	0.5888824	2.6475444	0.0081079	0.037455	NA	NA
	AT3G59040	114.36645	1.5568132	1.7418304	0.4237464	3.6739266	0.0002389	0.0027088	NA	NA
	AT3G09490	58.578257	1.5532123	1.890287	0.5255964	2.9551427	0.0031252	0.0184777	NA	NA
	AT1G01190	33.733908	1.5510086	1.8513127	0.5057142	3.0669667	0.0021624	0.0140632	CYP78A8	cytochrome P450, family 78, subfamily A, polypeptide 8
	AT5G61210	1115.1325	1.5508332	1.643738	0.3180061	4.8767405	1.08E-06	3.92E-05	ATSNAP33	0
	AT5G22020	184.2431	1.5496287	1.654994	0.3360625	4.6111328	4.00E-06	0.0001089	NA	NA
	AT3G19960	45.272771	1.5492866	1.7982501	0.4748384	3.2627657	0.0011033	0.0085664	ATM1	myosin 1
	AT5G01340	60.543264	1.5475094	1.7297264	0.4216688	3.6699645	0.0002426	0.0027391	AtmSFC1	0
	AT5G49410	23.428223	1.5459624	1.8985414	0.5332144	2.8993262	0.0037397	0.0210931	NA	NA
	AT5G20250	2085.8806	1.5412912	1.6531426	0.3458985	4.4559066	8.35E-06	0.000196	DIN10	DARK INDUCIBLE 10
	AT5G24690	936.85562	1.5379571	1.6090277	0.2829787	5.4348856	5.48E-08	3.54E-06	NA	NA
	AT5G57010	32.06708	1.5348674	1.978324	0.5710572	2.6877649	0.0071932	0.0343296	NA	NA
	AT5G17230	1663.8984	1.5340897	1.6321261	0.3272233	4.6882043	2.76E-06	7.97E-05	PSY	PHYTOENE SYNTHASE
	AT1G30590	93.436527	1.5337243	1.6801406	0.3881537	3.9513324	7.77E-05	0.0011461	NA	NA
YES	AT5G39710	22.090652	1.5333118	1.9233551	0.5511753	2.7818953	0.0054042	0.0276691	EMB2745	EMBRYO DEFECTIVE 2745

Supplemental Table 3-RNA seq data comparing *Pro35S::MYB63 Pro35S::LAC17* with wild type.

DAP.MYB63.target(1.5kb upstream TSS)	Gene	baseMean	log2FoldChange	lfcMLE	lfcSE	stat	pvalue	padj (FDR)	TAIR10 Symbol	TAIR10 Annotation (Short)
	AT2G0322C	121.88986	1.5323909	1.6414033	0.3425919	4.4729338	7.72E-06	0.000184	ATFT1	ARABIDOPSIS THALIANA FUCOSYLTRANSFERASE 1
	AT4G0533C	350.9429	1.5321925	1.6722931	0.3815118	4.0161079	5.92E-05	0.0009176	AGD13	ARF-GAP domain 13
	AT5G0681C	38.000346	1.5319215	1.8129155	0.4965685	3.0850154	0.0020354	0.0134261	NA	NA
	AT1G8077C	234.04817	1.5310384	1.6219458	0.3166447	4.8351929	1.33E-06	4.60E-05	PDE318	pigment defective 318
	AT5G2048C	85.132326	1.5305301	1.6552562	0.363007	4.216255	2.48E-05	0.0004707	EFR	EF-TU receptor
	AT1G6385C	17.422131	1.5277017	2.0691209	0.6010812	2.5415894	0.011035	0.0470789	NA	NA
	AT1G0376C	33.627014	1.5271038	1.831539	0.5105199	2.9912721	0.0027782	0.0169312	NA	NA
	AT5G3617C	384.36664	1.5244519	1.6286606	0.3369803	4.5238603	6.07E-06	0.0001518	ATPRFB	0
YES	AT1G6593C	2422.2698	1.523907	1.5991145	0.2915786	5.2264024	1.73E-07	9.19E-06	cICDH	cytosolic NADP+-dependent isocitrate dehydrogenase
YES	AT5G1327C	19.23148	1.523811	1.8801365	0.5370135	2.8375653	0.0045459	0.024367	RARE1	REQUIRED FOR ACCD RNA EDITING 1
	AT1G1278C	932.96671	1.523269	1.6093382	0.3098582	4.9160193	8.83E-07	3.36E-05	ATUGE1	A. THALIANA UDP-GLC 4-EPIMERASE 1
	AT1G2328C	32.748708	1.5229301	1.8916222	0.5429377	2.8049811	0.005032	0.0262558	NA	NA
	AT2G4659C	43.186724	1.5213909	1.8446696	0.5226561	2.9108834	0.0036041	0.0205428	DAG2	DOF AFFECTING GERMINATION 2
	AT4G0299C	246.46842	1.5213757	1.6553356	0.3753049	4.0537055	5.04E-05	0.0008148	BSM	BELAYA SMERT
	AT5G4391C	109.55192	1.5211233	1.6675351	0.3895	3.9053228	9.41E-05	0.0013248	NA	NA
	AT2G2037C	76.257667	1.5208956	1.6581521	0.3788034	4.0150003	5.94E-05	0.0009188	AtMUR3	0
	AT1G5216C	66.256891	1.5205876	1.7178377	0.4379182	3.4723099	0.000516	0.0048348	TRZ3	tRNAse Z3
	AT4G06534	15.600461	1.5192359	1.9277243	0.5593345	2.7161493	0.0066046	0.032168	NA	NA
	AT2G3750C	118.44731	1.5178695	1.6348547	0.35449	4.2818398	1.85E-05	0.000369	NA	NA
	AT3G1379C	214.75737	1.5173374	1.612801	0.3248754	4.6705219	3.00E-06	8.58E-05	ATBFRUCT1	0
	AT1G6904C	301.94721	1.5169012	1.6322627	0.3527657	4.3000244	1.71E-05	0.0003478	ACR4	ACT domain repeat 4
	AT2G1949C	29.521649	1.5164825	1.7968141	0.4983715	3.0428754	0.0023433	0.0149007	RECA2	A. thaliana recA homolog 2
	AT2G3025C	792.36389	1.5163786	1.6201164	0.3371062	4.4982226	6.85E-06	0.0001687	ATWRKY25	0
	AT1G0404C	190.54138	1.5159696	1.6762439	0.4046709	3.7461788	0.0001795	0.0021809	NA	NA
	AT4G0957C	113.61019	1.5147164	1.6870856	0.4164734	3.637006	0.0002758	0.0030089	ATCPK4	0
	AT4G1727C	541.12965	1.51446	1.5982687	0.306939	4.9340742	8.05E-07	3.13E-05	NA	NA
	AT5G4100C	55.116712	1.5138658	1.6907337	0.4205965	3.5993302	0.000319	0.0033485	AtYSL4	0
	AT5G5478C	95.176799	1.5137791	1.6488337	0.377354	4.0115627	6.03E-05	0.0009291	NA	NA
	AT5G4499C	14.354905	1.512921	1.9376739	0.5653664	2.6760011	0.0074506	0.0352132	NA	NA
	AT2G1967C	41.229216	1.5123949	1.7082333	0.4376371	3.4558194	0.0005486	0.0050597	ATPRMT1A	ARABIDOPSIS THALIANA PROTEIN ARGININE METHYLTRANSFERASE 1A
	AT2G4012C	38.530867	1.5084597	1.7541272	0.4761712	3.1678939	0.0015355	0.0108854	NA	NA
	AT4G3767C	48.896321	1.507132	1.7120024	0.4457052	3.3814548	0.000721	0.0061946	NAGS2	N-acetyl-l-glutamate synthase 2
	AT5G5023C	74.984003	1.5066373	1.672807	0.4109877	3.6658935	0.0002465	0.0027744	NA	NA
	AT4G3142C	118.62715	1.5065698	1.6445955	0.3815585	3.9484637	7.87E-05	0.0011517	REIL1	REI1-LIKE 1
	AT3G5472C	129.74333	1.5064123	1.6421344	0.3791187	3.9734582	7.08E-05	0.0010629	AMP1	ALTERED MERISTEM PROGRAM 1
	AT4G0334C	14.8201	1.5049861	1.931925	0.5678685	2.6502369	0.0080435	0.0372437	NA	NA
	AT3G1948C	150.71914	1.5048888	1.6286418	0.3647525	4.1257807	3.69E-05	0.0006372	3-PGDH	0
	AT1G0908C	26.687404	1.504368	1.8365224	0.5280741	2.8487821	0.0043887	0.0236863	BIP3	binding protein 3
	AT1G3118C	441.78868	1.5030184	1.5948446	0.3207438	4.6860405	2.79E-06	8.03E-05	ATIMD3	ARABIDOPSIS ISOPROPYLMALATE DEHYDROGENASE 3
	AT5G1332C	132.66278	1.5026164	1.6998933	0.4404336	3.411675	0.0006457	0.005726	AtGH3.12	0

Supplemental Table 3-RNA seq data comparing *Pro35S::MYB63 Pro35S::LAC17* with wild type.

DAP.MYB63.target(1.5kb upstream TSS)	Gene	baseMean	log2FoldChange	lfcMLE	lfcSE	stat	pvalue	padj (FDR)	TAIR10 Symbol	TAIR10 Annotation (Short)
	AT5G3760C	1372.1687	1.5014427	1.5658909	0.2735996	5.4877365	4.07E-08	2.79E-06	ATGLN1;1	ARABIDOPSIS GLUTAMINE SYNTHASE 1;1
	AT3G4780C	1460.1295	1.501076	1.5746282	0.290634	5.1648335	2.41E-07	1.21E-05	NA	NA
	AT1G6315C	22.069757	1.5008796	1.827494	0.5250117	2.8587546	0.0042531	0.0230927	NA	NA
	AT5G0605C	57.367727	1.5005058	1.673666	0.4186573	3.5840906	0.0003383	0.0034983	NA	NA
	AT3G2224C	26.789665	-1.500158	-1.856033	0.5395757	-2.780255	0.0054316	0.0277679	NA	NA
	AT3G1406C	123.7589	-1.500844	-1.76481	0.4901994	-3.061701	0.0022008	0.0142359	NA	NA
	AT5G2599C	286.39745	-1.500865	-1.596372	0.3264882	-4.596996	4.29E-06	0.0001151	NA	NA
	AT1G51402	62.537745	-1.500882	-1.736019	0.4704437	-3.190354	0.001421	0.01028	NA	NA
	AT2G26695	211.76754	-1.501659	-1.596363	0.3251493	-4.618368	3.87E-06	0.0001058	NA	NA
	AT3G0440C	39502.377	-1.501724	-1.632329	0.373622	-4.019367	5.84E-05	0.0009073	emb2171	embryo defective 2171
	AT5G1405C	117.12782	-1.502347	-1.615248	0.3508501	-4.282018	1.85E-05	0.000369	NA	NA
	AT1G7202C	7096.2204	-1.502481	-1.611331	0.3456403	-4.34695	1.38E-05	0.0002947	NA	NA
	AT5G3898C	3813.4734	-1.50259	-1.591259	0.3159184	-4.756261	1.97E-06	6.16E-05	NA	NA
	AT1G6693C	37.78105	-1.50271	-1.785026	0.5009187	-2.999907	0.0027006	0.0165932	NA	NA
YES	AT3G4994C	4724.7213	-1.503878	-1.621236	0.3568533	-4.214276	2.51E-05	0.0004738	LBD38	LOB domain-containing protein 38
	AT1G1445C	4865.2843	-1.504496	-1.604136	0.332405	-4.526093	6.01E-06	0.0001504	NA	NA
	AT4G3043C	15.77838	-1.505002	-1.894641	0.5535958	-2.718594	0.006556	0.0319919	TET9	tetraspanin9
	AT3G12587	628.97469	-1.505607	-1.643897	0.3821315	-3.940022	8.15E-05	0.0011884	NA	NA
	AT2G2505C	66.288756	-1.505696	-1.661504	0.4008071	-3.75666	0.0001722	0.0021139	NA	NA
	AT3G2477C	1097.3425	-1.505963	-1.643469	0.3812422	-3.950148	7.81E-05	0.0011471	CLE41	CLAVATA3/ESR-RELATED 41
	AT5G6587C	551.27521	-1.506074	-1.65683	0.3959131	-3.804052	0.0001423	0.0018271	ATPSK5	phytosulfokine 5 precursor
	AT5G6631C	15.931081	-1.507186	-1.907008	0.5573237	-2.704329	0.0068443	0.0330052	NA	NA
	AT3G03341	51.780325	-1.507352	-1.715612	0.4484849	-3.360986	0.0007766	0.0065678	NA	NA
	AT1G0429C	938.98897	-1.50739	-1.633214	0.3671617	-4.105519	4.03E-05	0.0006839	NA	NA
	AT3G2788C	488.39125	-1.507738	-1.63029	0.3629851	-4.153718	3.27E-05	0.0005768	NA	NA
	AT4G3071C	25.274128	-1.508361	-1.879412	0.5457148	-2.76401	0.0057096	0.0288456	QWRF8	QWRF domain containing 8
	AT5G5610C	1347.7939	-1.508901	-1.638932	0.3721385	-4.054676	5.02E-05	0.0008127	NA	NA
	AT2G4694C	59.631543	-1.508937	-1.691885	0.4267547	-3.535841	0.0004065	0.0040094	NA	NA
	AT5G6091C	172.52238	-1.509793	-1.643263	0.375967	-4.015759	5.93E-05	0.0009179	AGL8	AGAMOUS-like 8
	AT2G2132C	152.17427	-1.509952	-1.677734	0.4128182	-3.657669	0.0002545	0.0028323	BBX18	B-box domain protein 18
	AT4G4008C	161.6471	-1.510092	-1.663648	0.3984461	-3.789952	0.0001507	0.001905	NA	NA
	AT2G4211C	58.002263	-1.510395	-1.80225	0.5060999	-2.984382	0.0028415	0.0172391	NA	NA
	AT4G2723C	1361.9344	-1.510458	-1.608431	0.3293561	-4.586095	4.52E-06	0.0001199	HTA2	histone H2A 2
	AT3G2813C	675.10369	-1.510595	-1.623327	0.350048	-4.315393	1.59E-05	0.0003293	UMAMIT44	Usually multiple acids move in and out Transporters 44
	AT4G28703	361.88016	-1.510607	-1.712009	0.4434094	-3.406799	0.0006573	0.0057939	NA	NA
	AT1G0777C	12735.025	-1.510627	-1.609927	0.3313272	-4.559319	5.13E-06	0.0001325	RPS15A	ribosomal protein S15A
	AT1G1392C	47.597565	-1.510767	-1.711709	0.4420247	-3.417834	0.0006312	0.0056368	NA	NA
	AT5G2015C	292.8448	-1.511015	-1.63337	0.362399	-4.169478	3.05E-05	0.0005502	ATSPX1	ARABIDOPSIS THALIANA SPX DOMAIN GENE 1
	AT3G2068C	1982.9769	-1.511827	-1.616013	0.338208	-4.470111	7.82E-06	0.0001859	NA	NA
	AT5G5734C	575.58732	-1.515796	-1.613595	0.3285676	-4.613345	3.96E-06	0.000108	NA	NA
	AT2G27402	733.62545	-1.516454	-1.606891	0.3173447	-4.77857	1.77E-06	5.65E-05	NA	NA
	AT3G5750C	45.341091	-1.516464	-1.697688	0.4245307	-3.572095	0.0003541	0.0036199	NA	NA
	AT3G53232	276.93403	-1.517208	-1.680074	0.407237	-3.725614	0.0001948	0.0023222	DVL20	DEVIL 20
	AT5G4326C	2126.9068	-1.517409	-1.601895	0.307828	-4.929405	8.25E-07	3.20E-05	NA	NA
	AT3G0887C	45.482553	-1.517435	-1.787332	0.4922258	-3.082802	0.0020506	0.0135066	LecRK-VI.1	L-type lectin receptor kinase VI.1
	AT1G7203C	1967.6291	-1.51754	-1.638814	0.3604708	-4.209882	2.56E-05	0.0004786	NA	NA

Supplemental Table 3-RNA seq data comparing *Pro35S::MYB63 Pro35S::LAC17* with wild type.

DAP.MYB63.target(1.5kb upstream TSS)	Gene	baseMean	log2FoldChange	lfcMLE	lfcSE	stat	pvalue	padj (FDR)	TAIR10 Symbol	TAIR10 Annotation (Short)
	AT5G60890	248.35925	-1.51821	-1.678719	0.4046783	-3.751646	0.0001757	0.002142	ATMYB34	0
YES	AT3G23805	896.46753	-1.51829	-1.648951	0.3718988	-4.082535	4.45E-05	0.0007392	RALFL24	ralf-like 24
	AT2G20875	110.01999	-1.519441	-1.716739	0.4385497	-3.464694	0.0005308	0.0049412	EPF1	EPIDERMAL PATTERNING FACTOR 1
	AT3G57320	701.3275	-1.519458	-1.627623	0.3429613	-4.430405	9.41E-06	0.0002181	NA	NA
	AT1G49310	77.936737	-1.519829	-1.761049	0.472427	-3.217066	0.0012951	0.0096322	NA	NA
	ATMG0069	155.29851	-1.521032	-1.714489	0.4351936	-3.495069	0.0004739	0.0045238	ORF240A	0
	AT3G07910	1762.2999	-1.522467	-1.65029	0.3680822	-4.136215	3.53E-05	0.0006124	NA	NA
	AT5G59030	4448.219	-1.523878	-1.614244	0.3166057	-4.813172	1.49E-06	4.99E-05	COPT1	copper transporter 1
	AT3G51600	20944.921	-1.523983	-1.616664	0.3201732	-4.759871	1.94E-06	6.07E-05	LTP5	lipid transfer protein 5
	AT4G31875	102.32932	-1.52417	-1.670961	0.3892636	-3.91552	9.02E-05	0.0012882	NA	NA
	AT5G60680	8838.5099	-1.524373	-1.60662	0.3035425	-5.021944	5.12E-07	2.19E-05	NA	NA
YES	AT5G59470	524.54639	-1.524959	-1.651033	0.3656192	-4.170894	3.03E-05	0.0005479	NA	NA
	AT3G60490	34.229559	-1.525885	-1.759505	0.4663183	-3.272196	0.0010672	0.0083324	NA	NA
	AT1G72750	2803.905	-1.526855	-1.652862	0.365433	-4.178207	2.94E-05	0.0005365	ATTIM23-2	translocase inner membrane subunit 23-2
	AT2G31141	188.12887	-1.526881	-1.653416	0.3658076	-4.174	2.99E-05	0.0005432	NA	NA
	AT3G22210	2538.2499	-1.526914	-1.66005	0.3739952	-4.08271	4.45E-05	0.0007392	NA	NA
	AT4G30660	2213.1721	-1.527107	-1.634175	0.3407487	-4.481623	7.41E-06	0.0001788	NA	NA
	AT5G65495	497.93912	-1.52714	-1.672546	0.3878229	-3.937726	8.23E-05	0.001197	NA	NA
	AT5G22210	1699.8033	-1.52866	-1.640233	0.3467218	-4.408896	1.04E-05	0.0002364	NA	NA
YES	AT2G35760	323.69118	-1.529292	-1.645354	0.3524193	-4.339409	1.43E-05	0.000302	NA	NA
	AT1G27045	15.636425	-1.530604	-1.96532	0.5683224	-2.693197	0.007077	0.0338682	ATHB54	homeobox protein 54
	AT1G54120	32.403075	-1.533	-1.818335	0.4987817	-3.073488	0.0021157	0.0138343	NA	NA
YES	AT1G66810	36.289961	-1.533906	-1.796194	0.4848724	-3.163525	0.0015587	0.0110122	NA	NA
	AT5G54720	48.323178	-1.53436	-1.777757	0.4718829	-3.25157	0.0011477	0.0088349	NA	NA
	AT5G57887	202.02186	-1.534535	-1.691169	0.3988303	-3.847588	0.0001193	0.0016012	NA	NA
	AT3G52910	63.770205	-1.534589	-1.719509	0.4259255	-3.602951	0.0003146	0.003317	AtGRF4	growth-regulating factor 4
	AT5G44260	616.88598	-1.534594	-1.682966	0.3903451	-3.931378	8.45E-05	0.0012235	AtTZF5	0
	AT2G43460	7763.9913	-1.537819	-1.648624	0.3448256	-4.459699	8.21E-06	0.0001934	NA	NA
	AT1G32210	3482.8773	-1.537906	-1.686705	0.390514	-3.938159	8.21E-05	0.0011962	ATDAD1	DEFENDER AGAINST APOPTOTIC DEATH 1
	AT4G01200	177.47502	-1.538347	-1.703395	0.4070231	-3.779508	0.0001571	0.0019638	NA	NA
	AT5G67070	2339.1272	-1.538642	-1.634522	0.3236185	-4.754492	1.99E-06	6.20E-05	RALFL34	ralf-like 34
	AT1G68760	435.67394	-1.538939	-1.64405	0.3368236	-4.568978	4.90E-06	0.0001282	ATNUDT1	ARABIDOPSIS THALIANA NUDIX HYDROLASE HOMOLOG 1
	AT4G02360	82.356544	-1.539129	-1.727441	0.4283664	-3.593019	0.0003269	0.0034148	NA	NA
	AT1G72520	240.1062	-1.539689	-1.776116	0.4672891	-3.294939	0.0009844	0.0078393	ATLOX4	Arabidopsis thaliana lipoxygenase 4
	AT2G13570	20.777667	-1.540944	-1.967875	0.5642103	-2.731152	0.0063113	0.0310417	NF-YB7	nuclear factor Y, subunit B7
	AT1G32760	101.65858	-1.542292	-1.725288	0.4233301	-3.643237	0.0002692	0.002962	NA	NA
	AT2G41730	327.52873	-1.542582	-1.641073	0.3269849	-4.717592	2.39E-06	7.14E-05	NA	NA
	AT2G30230	1019.3755	-1.542862	-1.688376	0.3863863	-3.993055	6.52E-05	0.000992	NA	NA
	AT2G46250	54.796666	-1.544083	-1.754066	0.4464059	-3.458922	0.0005423	0.0050172	NA	NA
	AT5G52120	89.538459	-1.544664	-1.75616	0.4477074	-3.450164	0.0005602	0.0051328	AtPP2-A14	phloem protein 2-A14
	AT3G48580	25.841585	-1.546137	-1.83056	0.4967112	-3.112749	0.0018535	0.0125621	XTH11	xyloglucan endotransglucosylase/hydrolase 11
YES	AT4G39955	65.692575	-1.546936	-1.708375	0.4020822	-3.847313	0.0001194	0.0016012	NA	NA
	AT5G64230	88.468128	-1.547534	-1.713298	0.4063804	-3.808093	0.00014	0.0018076	NA	NA
	AT4G01897	1292.7145	-1.547581	-1.686439	0.3784723	-4.08902	4.33E-05	0.0007255	NA	NA
	AT5G05960	981.38449	-1.54933	-1.655931	0.3379514	-4.584475	4.55E-06	0.0001206	NA	NA

Supplemental Table 3-RNA seq data comparing *Pro35S::MYB63 Pro35S::LAC17* with wild type.

DAP.MYB63.target(1.5kb upstream TSS)	Gene	baseMean	log2FoldChange	lfcMLE	lfcSE	stat	pvalue	padj (FDR)	TAIR10 Symbol	TAIR10 Annotation (Short)
	AT2G2796C	1989.9649	-1.549713	-1.676213	0.3637251	-4.260672	2.04E-05	0.0003992	CKS1	cyclin-dependent kinase-subunit 1
	AT5G1892C	72.134023	-1.549927	-1.762187	0.4473479	-3.464701	0.0005308	0.0049412	NA	NA
	AT2G3076C	73.847045	-1.550938	-1.731217	0.4198147	-3.694339	0.0002205	0.0025543	NA	NA
	AT3G22235	46536.584	-1.551294	-1.670585	0.3546216	-4.374506	1.22E-05	0.0002688	NA	NA
	AT3G6240C	2791.1492	-1.551541	-1.677376	0.3627409	-4.27727	1.89E-05	0.0003751	NA	NA
	AT1G1594C	13.262305	-1.551983	-2.100059	0.5997983	-2.587509	0.0096673	0.0427147	NA	NA
	AT3G2540C	143.97721	-1.552252	-1.690063	0.376482	-4.123044	3.74E-05	0.0006429	NA	NA
	AT5G6382C	15.701239	-1.552532	-2.059137	0.5896241	-2.633087	0.0084613	0.0385538	NA	NA
	AT1G7904C	494177.25	-1.553471	-1.662166	0.3404731	-4.562685	5.05E-06	0.0001313	PSBR	photosystem II subunit R
	AT4G1137C	334.00678	-1.554247	-1.682631	0.3653785	-4.2538	2.10E-05	0.0004099	RHA1A	RING-H2 finger A1A
	AT1G63245	38.658213	-1.554483	-1.863584	0.510081	-3.047522	0.0023074	0.0147034	CLE14	CLAVATA3/ESR-RELATED 14
	AT4G1486C	25.86985	-1.554853	-1.939026	0.5458689	-2.8484	0.004394	0.0237006	AtOFP11	ovate family protein 11
	AT4G34881	59.72768	-1.55561	-1.828432	0.4893912	-3.178664	0.0014796	0.0105471	NA	NA
	AT5G1093C	344.1011	-1.555757	-1.688508	0.3704505	-4.199635	2.67E-05	0.0004957	CIPK5	CBL-interacting protein kinase 5
	AT3G5965C	460.01511	-1.556605	-1.692125	0.3736323	-4.166142	3.10E-05	0.0005561	NA	NA
	AT1G2263C	3808.3965	-1.557862	-1.672429	0.347871	-4.478275	7.52E-06	0.0001811	NA	NA
	AT5G0276C	176.45162	-1.558458	-1.68168	0.3585733	-4.346273	1.38E-05	0.0002953	APD7	Arabidopsis Pp2c clade D 7
	AT3G4560C	2862.007	-1.559448	-1.667812	0.3394343	-4.594254	4.34E-06	0.0001159	TET3	tetraspanin3
	AT2G30942	239.0607	-1.55963	-1.667704	0.3388595	-4.602586	4.17E-06	0.0001129	NA	NA
	AT5G05365	93.701404	-1.560782	-1.724344	0.4029079	-3.873795	0.0001072	0.0014673	NA	NA
	AT2G0302C	16.900008	-1.562399	-2.105684	0.5989101	-2.608736	0.0090877	0.0407084	NA	NA
	AT1G0995C	30.026435	-1.565234	-1.969159	0.5538203	-2.82625	0.0047097	0.0250141	RAS1	RESPONSE TO ABA AND SALT 1
	AT5G3877C	38.583397	-1.565392	-1.82111	0.4770014	-3.281734	0.0010317	0.0081367	AtGDU7	glutamine dumper 7
	AT2G4067C	262.22856	-1.566051	-1.693137	0.3627106	-4.317633	1.58E-05	0.0003265	ARR16	response regulator 16
	AT1G6608C	123.01949	-1.566672	-1.715437	0.3869957	-4.048292	5.16E-05	0.0008294	NA	NA
	AT4G0206C	30.707874	-1.566931	-1.837507	0.4861124	-3.223393	0.0012668	0.0094924	MCM7	0
	AT2G43445	22.067308	-1.567549	-1.925154	0.5329772	-2.941118	0.0032703	0.0191345	NA	NA
	AT3G5037C	125.37211	-1.567717	-1.732006	0.4031153	-3.889005	0.0001007	0.0013929	NA	NA
	ATMG0006	969.51264	-1.568913	-1.67836	0.339963	-4.614952	3.93E-06	0.0001074	NAD5	NADH DEHYDROGENASE SUBUNIT 5
	AT2G4353C	3106.1497	-1.569288	-1.669798	0.3275555	-4.790907	1.66E-06	5.41E-05	NA	NA
	AT1G49475	47.03474	-1.569342	-1.829777	0.4798297	-3.270624	0.0010731	0.008371	NA	NA
	AT1G7466C	33.37247	-1.569784	-1.828166	0.4786282	-3.279756	0.001039	0.0081654	MIF1	mini zinc finger 1
	AT1G13245	3807.8567	-1.570106	-1.695369	0.3602196	-4.358746	1.31E-05	0.0002833	DVL4	DEVIL 4
	AT4G01335	48.132179	-1.571523	-1.762925	0.4275386	-3.675746	0.0002372	0.002693	NA	NA
	AT1G5993C	182.60428	-1.57166	-1.671854	0.3266331	-4.811698	1.50E-06	5.00E-05	NA	NA
	AT4G0106C	63.192504	-1.572867	-1.808735	0.4625972	-3.40008	0.0006737	0.005899	CPL3	CAPRICE-like MYB3
	AT2G01505	140.15729	-1.572956	-1.751509	0.4160461	-3.780726	0.0001564	0.0019583	CLE16	CLAVATA3/ESR-RELATED 16
	AT2G1446C	173.82435	-1.574204	-1.746807	0.4104921	-3.834918	0.0001256	0.0016656	NA	NA
	AT3G22142	17.95854	-1.574414	-1.973732	0.5502179	-2.861438	0.0042172	0.0229658	NA	NA
	AT5G1620C	299.6404	-1.575036	-1.687359	0.3430704	-4.590998	4.41E-06	0.0001174	NA	NA
	AT3G4632C	1073.767	-1.575059	-1.700132	0.3594548	-4.3818	1.18E-05	0.0002609	NA	NA
	ATMG0004	24.493739	-1.575132	-2.052944	0.5795034	-2.718073	0.0065663	0.0320249	ORF315	0
	AT1G2943C	717.36976	-1.575439	-1.68432	0.3385839	-4.653023	3.27E-06	9.21E-05	SAUR62	SMALL AUXIN UPREGULATED RNA 62
	AT4G3228C	418.00676	-1.575858	-1.710577	0.3707898	-4.250004	2.14E-05	0.0004147	IAA29	indole-3-acetic acid inducible 29
	AT3G5273C	5772.0521	-1.575914	-1.716412	0.3774977	-4.174631	2.98E-05	0.0005422	NA	NA
	AT2G2546C	44.747284	-1.57616	-1.784575	0.4410761	-3.573443	0.0003523	0.0036116	NA	NA
	AT5G3878C	9.6112168	-1.576584	-2.247704	0.6248043	-2.523325	0.0116251	0.0488791	NA	NA

Supplemental Table 3-RNA seq data comparing *Pro35S::MYB63 Pro35S::LAC17* with wild type.

DAP.MYB63.target(1.5kb upstream TSS)	Gene	baseMean	log2FoldChange	lfcMLE	lfcSE	stat	pvalue	padj (FDR)	TAIR10 Symbol	TAIR10 Annotation (Short)
	AT1G54630	4704.5095	-1.5789	-1.708324	0.3643887	-4.33301	1.47E-05	0.0003093	ACP3	acyl carrier protein 3
	AT3G21460	1496.4884	-1.579242	-1.687806	0.3378181	-4.674829	2.94E-06	8.44E-05	NA	NA
	AT5G02280	845.16321	-1.579326	-1.690122	0.3407979	-4.634201	3.58E-06	9.90E-05	NA	NA
	AT5G02090	245.17382	-1.579622	-1.83203	0.4740571	-3.332135	0.0008618	0.0071047	NA	NA
	ATCG00700	86.289485	-1.579948	-1.714212	0.3695764	-4.275025	1.91E-05	0.0003772	PSBN	photosystem II reaction center protein N
	AT3G44220	192.60437	-1.580589	-1.74792	0.4047993	-3.904623	9.44E-05	0.0013276	NA	NA
	AT4G15440	147.043	-1.580836	-1.71521	0.3697022	-4.275972	1.90E-05	0.0003765	CYP74B2	0
	AT5G61290	85.319976	-1.582159	-1.75012	0.4049106	-3.907428	9.33E-05	0.0013175	NA	NA
	AT1G17200	3061.4949	-1.582818	-1.685563	0.3294821	-4.803958	1.56E-06	5.15E-05	NA	NA
	AT3G59540	5431.9741	-1.582907	-1.701668	0.35088	-4.511249	6.44E-06	0.0001598	NA	NA
	AT1G60590	17.478042	-1.582919	-2.223413	0.6202442	-2.552091	0.0107079	0.0461224	NA	NA
	AT1G80520	88.275958	-1.585219	-1.822207	0.4622469	-3.429378	0.000605	0.0054565	NA	NA
	AT3G13110	2167.3033	-1.585259	-1.660516	0.2864765	-5.533644	3.14E-08	2.26E-06	ATSERAT2;2	serine acetyltransferase 2;2
	AT1G20823	178.30221	-1.585476	-1.719356	0.3688464	-4.298472	1.72E-05	0.0003494	NA	NA
	AT5G47610	36.483973	-1.586311	-1.835124	0.4697415	-3.376988	0.0007328	0.0062769	NA	NA
	AT5G25340	34.066275	-1.586795	-1.843081	0.4745324	-3.343913	0.0008261	0.0068696	NA	NA
	AT1G19910	10022.863	-1.587147	-1.717387	0.3645604	-4.353592	1.34E-05	0.0002877	ATVHA-C2	VACUOLAR-TYPE H+ ATPASE C2
	AT3G25717	912.29493	-1.587623	-1.687697	0.3252025	-4.881951	1.05E-06	3.85E-05	DVL6	DEVIL 6
	ATMG0016	501.04618	-1.588039	-1.718773	0.3649307	-4.351617	1.35E-05	0.0002892	COX2	cytochrome oxidase 2
	AT3G15540	325.04424	-1.589296	-1.701658	0.3418405	-4.649234	3.33E-06	9.34E-05	IAA19	indole-3-acetic acid inducible 19
	AT3G06890	96.077322	-1.589817	-1.786756	0.4304335	-3.693525	0.0002212	0.0025598	NA	NA
	AT1G75335	136.49484	-1.590839	-1.75363	0.3990938	-3.98613	6.72E-05	0.0010145	NA	NA
	AT3G19508	77.170277	-1.593126	-1.834357	0.4642196	-3.431837	0.0005995	0.0054208	NA	NA
YES	AT5G13930	515.19984	-1.59381	-1.734551	0.3758879	-4.24012	2.23E-05	0.0004301	ATCHS	0
	AT1G16820	367.83784	-1.593998	-1.711048	0.3474653	-4.587503	4.49E-06	0.0001192	NA	NA
	AT1G75050	18.512219	-1.595448	-2.132808	0.5945365	-2.683515	0.0072853	0.034668	NA	NA
	AT4G16750	168.58178	-1.596743	-1.74467	0.3832418	-4.166411	3.09E-05	0.000556	NA	NA
	AT1G18835	11.299687	-1.598182	-2.27067	0.6240693	-2.560905	0.01044	0.0453551	MIF3	mini zinc finger 3
	AT5G28442	77.583186	-1.598294	-1.779602	0.4156769	-3.84504	0.0001205	0.0016138	NA	NA
	AT2G24550	3301.2147	-1.599778	-1.691792	0.3123283	-5.122104	3.02E-07	1.45E-05	NA	NA
	AT5G65040	48.822688	-1.599928	-1.824109	0.4510481	-3.547135	0.0003894	0.0038903	NA	NA
	AT3G47836	3218.4442	-1.600126	-1.738234	0.3723932	-4.296874	1.73E-05	0.0003511	NA	NA
	AT4G39950	247.77384	-1.600308	-1.71652	0.3457586	-4.628395	3.69E-06	0.0001014	CYP79B2	cytochrome P450, family 79, subfamily B, polypeptide 2
	AT1G56045	8772.862	-1.601205	-1.707027	0.3320968	-4.821502	1.42E-06	4.85E-05	NA	NA
	AT2G43150	335.79129	-1.601584	-1.732633	0.3640121	-4.399809	1.08E-05	0.0002444	NA	NA
	AT1G24140	140.79987	-1.602378	-1.738451	0.3695448	-4.336086	1.45E-05	0.0003061	NA	NA
	AT4G00810	8780.5533	-1.603338	-1.728311	0.3566676	-4.495329	6.95E-06	0.0001701	NA	NA
	AT1G56290	13.170414	-1.604215	-2.138714	0.5917515	-2.710961	0.0067089	0.0325351	NA	NA
	AT4G31330	175.40132	-1.60428	-1.75741	0.3880862	-4.133825	3.57E-05	0.0006176	NA	NA
	AT2G19710	28.321715	-1.604327	-1.992868	0.5430008	-2.954557	0.0031312	0.0185007	NA	NA
	AT3G11110	570.59131	-1.604459	-1.706123	0.3259269	-4.922756	8.53E-07	3.27E-05	NA	NA
	AT5G54980	859.79102	-1.607093	-1.701587	0.3153458	-5.096287	3.46E-07	1.60E-05	NA	NA
	AT3G61840	41.470624	-1.60763	-1.851428	0.4641743	-3.463419	0.0005334	0.004957	NA	NA
	AT1G71050	335.60596	-1.608138	-1.76179	0.3883214	-4.141256	3.45E-05	0.0006022	HIPP20	heavy metal associated isoprenylated plant protein 20
	AT2G21180	402.11349	-1.608272	-1.760782	0.3870973	-4.154697	3.26E-05	0.0005755	NA	NA
	AT2G27580	1698.6989	-1.60907	-1.740465	0.3637708	-4.423307	9.72E-06	0.0002234	NA	NA
	AT3G44010	5441.981	-1.609181	-1.746356	0.3704628	-4.343704	1.40E-05	0.0002974	NA	NA
	AT4G25890	4409.778	-1.610505	-1.752605	0.3758533	-4.284929	1.83E-05	0.000366	NA	NA

Supplemental Table 3-RNA seq data comparing *Pro35S::MYB63 Pro35S::LAC17* with wild type.

DAP.MYB63.target(1.5kb upstream TSS)	Gene	baseMean	log2FoldChange	lfcMLE	lfcSE	stat	pvalue	padj (FDR)	TAIR10 Symbol	TAIR10 Annotation (Short)
	AT1G31835	204.6998	-1.611261	-1.747376	0.3687422	-4.369613	1.24E-05	0.000273	NA	NA
	AT3G55850	504.16316	-1.611643	-1.690908	0.2910675	-5.537007	3.08E-08	2.23E-06	LAF3	LONG AFTER FAR-RED 3
YES	AT3G22540	17.996488	-1.612048	-2.039691	0.5570797	-2.893747	0.0038067	0.021332	NA	NA
	AT1G30757	219.49126	-1.613445	-1.837797	0.4503411	-3.582719	0.00034	0.0035076	NA	NA
	AT1G73540	812.96692	-1.614937	-1.706145	0.3097586	-5.213536	1.85E-07	9.68E-06	atnudt21	nudix hydrolase homolog 21
	AT3G23450	603.8627	-1.615404	-1.707815	0.3114976	-5.185929	2.15E-07	1.11E-05	NA	NA
	AT5G47700	8242.3032	-1.615887	-1.772315	0.3905956	-4.136981	3.52E-05	0.000611	NA	NA
	AT4G35270	43.159562	-1.616368	-1.824006	0.4363581	-3.704225	0.000212	0.0024732	NLP2	NIN-like protein 2
	AT3G53890	8976.1401	-1.617266	-1.726688	0.3355194	-4.820188	1.43E-06	4.88E-05	NA	NA
	AT5G54145	992.58956	-1.617424	-1.735621	0.3468716	-4.662889	3.12E-06	8.83E-05	NA	NA
	AT3G25030	205.84982	-1.617485	-1.811795	0.4257154	-3.799452	0.000145	0.0018534	NA	NA
	AT5G57290	9277.1779	-1.617729	-1.78059	0.3969089	-4.07582	4.59E-05	0.0007573	NA	NA
	AT3G54120	167.52352	-1.618343	-1.787952	0.4031316	-4.014428	5.96E-05	0.0009202	NA	NA
	AT2G15318	8.6592308	-1.618865	-2.385522	0.6364148	-2.543726	0.0109677	0.046903	NA	NA
	AT3G62070	104.18683	-1.61957	-1.788488	0.4020826	-4.027954	5.63E-05	0.0008879	NA	NA
	AT2G19810	2973.3303	-1.620708	-1.766053	0.3784115	-4.282925	1.84E-05	0.0003681	AtOZF1	0
	AT5G59550	1099.7697	-1.621101	-1.720589	0.3214193	-5.04357	4.57E-07	1.98E-05	AtRDUF2	Arabidopsis thaliana RING and Domain of Unknown Function 1117 2
	AT1G51355	139.63474	-1.621652	-1.800823	0.4115949	-3.939922	8.15E-05	0.0011884	NA	NA
YES	AT1G67050	1325.0616	-1.622194	-1.705697	0.2971489	-5.459195	4.78E-08	3.17E-06	NA	NA
	AT1G07985	19.232629	-1.622476	-2.03003	0.5486451	-2.957241	0.0031041	0.0183947	NA	NA
	AT3G04290	241.23492	-1.623589	-1.855235	0.4548527	-3.569482	0.0003577	0.003644	ATLTL1	0
	AT3G49650	31.273806	-1.623655	-1.90366	0.4862266	-3.339297	0.0008399	0.0069591	NA	NA
	AT4G14010	1099.2667	-1.625185	-1.74015	0.3420259	-4.751642	2.02E-06	6.26E-05	RALFL32	ralf-like 32
	AT1G74160	45.310664	-1.625253	-1.833559	0.4357678	-3.729631	0.0001918	0.0022987	TRM4	TON1 Recruiting Motif 4
	AT1G71890	23.967477	-1.626038	-2.0632	0.5601776	-2.902718	0.0036994	0.0209619	ATSUC5	SUCROSE-PROTON SYMPORTER 5
	AT4G01410	509.40482	-1.62622	-1.72133	0.3145775	-5.169537	2.35E-07	1.19E-05	NA	NA
	AT1G19780	34.978189	-1.626366	-1.89825	0.4810442	-3.380907	0.0007225	0.0062028	ATCNGC8	cyclic nucleotide gated channel 8
	AT3G61770	1354.1618	-1.626692	-1.738796	0.3381849	-4.810067	1.51E-06	5.03E-05	NA	NA
	AT2G46380	38.136323	-1.628783	-1.86768	0.4580727	-3.555731	0.0003769	0.0037927	NA	NA
	ATMG0051	149.869	-1.629414	-1.804776	0.4072783	-4.000739	6.31E-05	0.0009668	NAD5	NADH DEHYDROGENASE SUBUNIT 5
	AT5G63500	1174.2891	-1.629805	-1.753004	0.3519601	-4.630654	3.65E-06	0.0001004	NA	NA
YES	AT1G36675	46.620068	-1.629806	-1.84252	0.4387384	-3.714756	0.0002034	0.0023973	NA	NA
	AT5G39800	630.14273	-1.629881	-1.734033	0.32714	-4.982213	6.29E-07	2.56E-05	NA	NA
	AT3G55230	9.5925907	-1.629927	-2.425508	0.6407294	-2.543862	0.0109634	0.0468958	NA	NA
	ATCG00570	13.905574	-1.630479	-2.125538	0.5775924	-2.82289	0.0047593	0.025196	PSBF	photosystem II reaction center protein F
	AT3G57450	4711.755	-1.630739	-1.783592	0.3854245	-4.231021	2.33E-05	0.0004441	NA	NA
	AT5G15520	517.66266	-1.630896	-1.777087	0.3782126	-4.312114	1.62E-05	0.000333	NA	NA
	AT5G58390	122.2872	-1.632133	-1.823692	0.4218025	-3.869424	0.0001091	0.0014871	NA	NA
	AT3G11690	636.87196	-1.632536	-1.749153	0.3434388	-4.753499	2.00E-06	6.22E-05	NA	NA
	AT5G25090	77.521204	-1.633349	-1.902517	0.4787942	-3.41138	0.0006463	0.0057294	AtENODL13	0
	AT5G16450	972.68107	-1.63474	-1.768343	0.3638647	-4.492714	7.03E-06	0.0001715	NA	NA
	AT1G23710	821.99322	-1.635637	-1.741121	0.32849	-4.979258	6.38E-07	2.58E-05	NA	NA
	AT2G37640	485.2988	-1.635658	-1.737502	0.3233795	-5.058014	4.24E-07	1.88E-05	ATEXP3	EXPANSIN 3
	AT5G44005	1091.1304	-1.636149	-1.780953	0.376289	-4.348117	1.37E-05	0.0002935	NA	NA
YES	AT2G16790	135.48423	-1.636818	-1.791391	0.3862327	-4.237906	2.26E-05	0.0004335	NA	NA
	AT4G34620	56783.354	-1.637471	-1.754819	0.3440154	-4.759876	1.94E-06	6.07E-05	SSR16	small subunit ribosomal protein 16

Supplemental Table 3-RNA seq data comparing *Pro35S::MYB63 Pro35S::LAC17* with wild type.

DAP.MYB63.target(1.5kb upstream TSS)	Gene	baseMean	log2FoldChange	lfcMLE	lfcSE	stat	pvalue	padj (FDR)	TAIR10 Symbol	TAIR10 Annotation (Short)
	AT2G2909C	35.601521	-1.638897	-1.967277	0.5117251	-3.202691	0.0013615	0.0099982	CYP707A2	cytochrome P450, family 707, subfamily A, polypeptide 2
	ATCG0005C	55.282073	-1.640122	-1.900025	0.4716047	-3.477747	0.0005056	0.0047608	RPS16	ribosomal protein S16
YES	AT2G3621C	7.3054781	-1.640637	-2.542909	0.6518654	-2.516833	0.0118415	0.0495778	SAUR45	SMALL AUXIN UPREGULATED RNA 45
	AT2G3076€	1796.3015	-1.640758	-1.791058	0.3817468	-4.298027	1.72E-05	0.0003497	NA	NA
	AT1G6735C	3410.8083	-1.640768	-1.758313	0.3439446	-4.770443	1.84E-06	5.85E-05	NA	NA
	AT5G1116C	93.114002	-1.642662	-1.795967	0.384183	-4.275728	1.91E-05	0.0003765	APT5	adenine phosphoribosyltransferase 5
	ATCG0065C	317.42534	-1.643381	-1.749949	0.3291819	-4.992318	5.97E-07	2.45E-05	RPS18	ribosomal protein S18
	AT1G1709C	95.919058	-1.64385	-1.89196	0.4640313	-3.542541	0.0003963	0.0039326	NA	NA
	AT1G6836C	28.737709	-1.644353	-2.064756	0.5513074	-2.982642	0.0028577	0.0173048	NA	NA
	AT1G7575C	9574.5937	-1.645991	-1.764031	0.3441108	-4.783317	1.72E-06	5.53E-05	GASA1	GAST1 protein homolog 1
	AT4G3355C	34.867243	-1.646235	-1.937147	0.4900105	-3.359592	0.0007806	0.0065942	NA	NA
	AT2G3203C	183.79897	-1.646874	-1.757605	0.334349	-4.925613	8.41E-07	3.24E-05	NA	NA
YES	AT3G20898	38.482071	-1.647189	-1.912073	0.4737777	-3.476713	0.0005076	0.0047743	NA	NA
	ATMG0009	887.07671	-1.647502	-1.770897	0.3505626	-4.699593	2.61E-06	7.63E-05	NA	NA
	AT5G1519C	47.569976	-1.647689	-2.039131	0.5401169	-3.050616	0.0022837	0.0145888	NA	NA
	AT5G3692C	60.558235	-1.647911	-1.899749	0.4658446	-3.537469	0.000404	0.0039877	NA	NA
	AT5G2063C	4516.9171	-1.649264	-1.800345	0.3817737	-4.320004	1.56E-05	0.0003251	ATGER3	ARABIDOPSIS THALIANA GERMIN 3
	ATMG0058	47.15802	-1.649627	-1.920095	0.4772298	-3.456673	0.0005469	0.0050463	NAD4	NADH dehydrogenase subunit 4
	AT1G1557C	17.105816	-1.649725	-2.072857	0.5520147	-2.988553	0.002803	0.0170538	CYCA2;3	CYCLIN A2;3
	AT3G1250C	13.516779	-1.650067	-2.172429	0.5836591	-2.827108	0.0046971	0.0249787	ATHCHIB	basic chitinase
	AT5G5557C	102.28193	-1.650118	-1.822334	0.4023429	-4.101273	4.11E-05	0.0006953	NA	NA
	AT5G5168C	11.295437	-1.651045	-2.554121	0.6529812	-2.528473	0.011456	0.0483677	NA	NA
	AT5G56795	14.710975	-1.65309	-2.118501	0.5657334	-2.922031	0.0034776	0.0200429	MT1B	metallothionein 1B
	AT5G2519C	1496.3066	-1.653095	-1.765379	0.3360627	-4.919008	8.70E-07	3.32E-05	ESE3	ethylene and salt inducible 3
	AT1G7563C	3880.8386	-1.65436	-1.770918	0.3414693	-4.844828	1.27E-06	4.41E-05	AVA-P4	vacuolar H+-pumping ATPase 16 kDa proteolipid subunit 4
	AT2G3887C	2489.2148	-1.655629	-1.785104	0.357139	-4.63581	3.56E-06	9.86E-05	NA	NA
YES	AT5G6221C	54.798398	-1.656009	-1.953527	0.4931997	-3.357685	0.000786	0.0066188	NA	NA
	AT5G6708C	118.20907	-1.656802	-1.986714	0.5111745	-3.241168	0.0011904	0.0090939	MAPKKK19	mitogen-activated protein kinase kinase kinase 19
	AT1G1170C	172.44257	-1.656872	-1.812103	0.384898	-4.304704	1.67E-05	0.000342	NA	NA
	AT1G3182C	125.75623	-1.657634	-1.803453	0.3750456	-4.419821	9.88E-06	0.0002262	PUT1	POLYAMINE UPTAKE TRANSPORTER 1
	AT5G35525	20.004217	-1.657762	-2.030021	0.5299956	-3.127879	0.0017607	0.0121153	NA	NA
	AT4G14819	11.577789	-1.65803	-2.501793	0.6446453	-2.572003	0.0101112	0.0441945	NA	NA
	AT2G3956C	53.605309	-1.658282	-1.886699	0.4476289	-3.704592	0.0002117	0.0024713	NA	NA
	AT2G2931C	1469.4655	-1.658365	-1.784348	0.3527328	-4.701475	2.58E-06	7.59E-05	NA	NA
	AT5G5158C	167.57668	-1.659137	-1.778031	0.3436315	-4.828246	1.38E-06	4.73E-05	NA	NA
	AT5G0302C	45.798335	-1.660036	-1.910135	0.4630493	-3.585009	0.0003371	0.003489	NA	NA
	AT1G25425	201.11191	-1.6601	-1.755976	0.3126688	-5.309454	1.10E-07	6.27E-06	CLE43	CLAVATA3/ESR-RELATED 43
	AT1G30515	27.604876	-1.660899	-2.004712	0.5165234	-3.215534	0.001302	0.0096683	NA	NA
	AT5G44565	348.74241	-1.661443	-1.811099	0.3789115	-4.384779	1.16E-05	0.000258	NA	NA
YES	AT2G3209C	3053.2592	-1.661473	-1.801526	0.3686971	-4.506336	6.60E-06	0.0001628	NA	NA
	AT5G5684C	62.114666	-1.661712	-1.907007	0.4597878	-3.614084	0.0003014	0.0032159	NA	NA

Supplemental Table 3-RNA seq data comparing *Pro35S::MYB63 Pro35S::LAC17* with wild type.

DAP.MYB63.target(1.5kb upstream TSS)	Gene	baseMean	log2FoldChange	lfcMLE	lfcSE	stat	pvalue	padj (FDR)	TAIR10 Symbol	TAIR10 Annotation (Short)
	AT4G15620	110.09926	-1.663514	-1.781433	0.3419966	-4.864126	1.15E-06	4.14E-05	NA	NA
	AT5G16250	492.06125	-1.663692	-1.784081	0.3453255	-4.817751	1.45E-06	4.91E-05	NA	NA
	AT3G48550	285.79777	-1.663901	-1.760902	0.3140182	-5.298741	1.17E-07	6.59E-06	NA	NA
	AT1G53690	34.965371	-1.664095	-1.996209	0.5112471	-3.254972	0.001134	0.0087513	NA	NA
	AT5G52220	51.589624	-1.664335	-1.859482	0.4209048	-3.954185	7.68E-05	0.0011353	NA	NA
	AT5G53486	96.84592	-1.664614	-1.802091	0.3650984	-4.559358	5.13E-06	0.0001325	NA	NA
	AT1G21550	389.51383	-1.666401	-1.769711	0.322747	-5.163181	2.43E-07	1.22E-05	NA	NA
	AT2G40205	1347.5758	-1.667214	-1.792049	0.3505299	-4.756266	1.97E-06	6.16E-05	NA	NA
	AT5G35490	2462.1871	-1.667214	-1.812466	0.3738434	-4.45966	8.21E-06	0.0001934	ATMRU1	ARABIDOPSIS MTO 1 RESPONDING UP 1
	AT2G38110	31.341451	-1.667545	-2.023982	0.5224129	-3.192006	0.0014129	0.0102503	ATGPAT6	GLYCEROL-3-PHOSPHATE sn-2-ACYLTRANSFERASE 6
	AT4G39780	442.02714	-1.667996	-1.790703	0.3477659	-4.796319	1.62E-06	5.31E-05	NA	NA
	ATCG00360	399.80204	-1.669423	-1.788862	0.3436243	-4.858278	1.18E-06	4.20E-05	YCF3	0
	AT4G09890	1048.0579	-1.669423	-1.778908	0.3309113	-5.044925	4.54E-07	1.97E-05	NA	NA
	AT1G07590	47776.347	-1.671246	-1.773503	0.321028	-5.20592	1.93E-07	1.01E-05	NA	NA
	AT4G30180	198.54094	-1.671278	-1.790014	0.3423948	-4.881143	1.05E-06	3.86E-05	NA	NA
	AT3G59900	59.252616	-1.672838	-1.872193	0.4234837	-3.950182	7.81E-05	0.0011471	ARGOS	AUXIN-REGULATED GENE INVOLVED IN ORGAN SIZE
	AT2G37530	101.71715	-1.672851	-1.830319	0.3855163	-4.339249	1.43E-05	0.000302	NA	NA
	AT1G19200	97.700089	-1.67385	-1.850585	0.40389	-4.144321	3.41E-05	0.0005963	NA	NA
	AT5G06190	93.260676	-1.675061	-1.855332	0.4069955	-4.115674	3.86E-05	0.0006601	NA	NA
	AT1G54020	18.655547	-1.676591	-2.111709	0.5534273	-3.029469	0.0024498	0.0154258	NA	NA
	AT5G50720	240.5517	-1.676767	-1.784406	0.3276437	-5.117654	3.09E-07	1.47E-05	ATHVA22E	ARABIDOPSIS THALIANA HVA22 HOMOLOGUE E
	AT1G62660	149.72773	-1.67894	-1.814014	0.3611531	-4.648832	3.34E-06	9.34E-05	NA	NA
	AT4G34720	8497.739	-1.680241	-1.757441	0.2822583	-5.95285	2.64E-09	2.74E-07	ATVHA-C1	0
	AT1G74929	52.333595	-1.680302	-1.958673	0.4794447	-3.504683	0.0004572	0.0043891	NA	NA
	AT4G27260	75.961229	-1.681645	-1.914388	0.4488651	-3.746436	0.0001794	0.0021809	GH3.5	0
	AT4G13520	7623.3207	-1.682346	-1.813534	0.3567221	-4.716125	2.40E-06	7.17E-05	SMAP1	small acidic protein 1
	AT5G38610	205.25031	-1.682593	-1.857141	0.4014192	-4.191611	2.77E-05	0.0005099	NA	NA
	AT5G44575	211.49827	-1.682739	-1.841145	0.385741	-4.362355	1.29E-05	0.0002804	NA	NA
	AT5G08040	1913.9338	-1.684342	-1.820292	0.3619645	-4.653336	3.27E-06	9.21E-05	TOM5	mitochondrial import receptor subunit TOM5 homolog
	AT1G68765	42.48442	-1.685534	-2.058325	0.5280427	-3.19204	0.0014127	0.0102503	IDA	INFLORESCENCE DEFICIENT IN ABSCISSION
	AT4G37235	8.8604382	-1.685841	-2.670951	0.6582148	-2.561232	0.0104302	0.0453248	NA	NA
	AT5G36220	149.80782	-1.687786	-1.840953	0.3799993	-4.441551	8.93E-06	0.0002085	CYP81D1	cytochrome P450, family 81, subfamily D, polypeptide 1
YES	AT3G52110	518.95034	-1.688094	-1.833991	0.3724787	-4.532055	5.84E-06	0.000147	NA	NA
	AT5G44574	12.345774	-1.688114	-2.284511	0.5998559	-2.8142	0.0048899	0.0256687	NA	NA
	AT4G37700	9.1200094	-1.689388	-2.54128	0.6439412	-2.623512	0.0087028	0.0393898	NA	NA
	AT1G62422	1057.0462	-1.689635	-1.839051	0.3761406	-4.49203	7.05E-06	0.0001718	NA	NA
YES	AT3G20865	142.70417	-1.689913	-1.84704	0.3836343	-4.40501	1.06E-05	0.0002398	AGP40	arabinogalactan protein 40
	AT4G12470	82.530948	-1.691509	-1.873608	0.4069798	-4.156248	3.24E-05	0.0005738	AZI1	azelaic acid induced 1
	AT5G02060	70.577363	-1.692905	-1.886621	0.4172318	-4.057468	4.96E-05	0.0008054	NA	NA
	AT3G03870	6512.5328	-1.694698	-1.851789	0.3836451	-4.41736	9.99E-06	0.0002285	NA	NA
	AT3G11120	4731.3366	-1.695086	-1.832329	0.362466	-4.676538	2.92E-06	8.39E-05	NA	NA
	ATCG00440	565.6045	-1.698369	-1.867391	0.3949259	-4.300474	1.70E-05	0.0003474	NDHC	0
	AT3G15680	1081.1894	-1.699341	-1.821434	0.3443252	-4.935278	8.00E-07	3.12E-05	NA	NA

Supplemental Table 3-RNA seq data comparing *Pro35S::MYB63 Pro35S::LAC17* with wild type.

DAP.MYB63.target(1.5kb upstream TSS)	Gene	baseMean	log2FoldChange	lfcMLE	lfcSE	stat	pvalue	padj (FDR)	TAIR10 Symbol	TAIR10 Annotation (Short)
	AT1G25422	14.425445	-1.700314	-2.275099	0.5930582	-2.867027	0.0041435	0.0226528	NA	NA
	AT1G03730	313.91234	-1.70062	-1.792389	0.3034242	-5.604762	2.09E-08	1.57E-06	NA	NA
	AT2G22820	24.1522	-1.702432	-2.176573	0.5646274	-3.015142	0.0025686	0.0159781	NA	NA
	AT4G11360	2507.8308	-1.703545	-1.791608	0.2977327	-5.721726	1.05E-08	8.97E-07	RHA1B	RING-H2 finger A1B
	AT4G29240	115.09465	-1.703691	-1.842349	0.3629368	-4.69418	2.68E-06	7.79E-05	NA	NA
	AT1G04250	1334.6304	-1.703891	-1.794183	0.3010472	-5.659882	1.51E-08	1.22E-06	AXR3	AUXIN RESISTANT 3
	AT1G24577	218.98641	-1.705116	-1.840598	0.3592399	-4.746454	2.07E-06	6.37E-05	NA	NA
	AT3G15353	106345.49	-1.705119	-1.840161	0.3590961	-4.748363	2.05E-06	6.34E-05	ATMT3	0
	AT3G27027	137.06304	-1.705181	-1.867912	0.3878426	-4.39658	1.10E-05	0.0002474	NA	NA
	AT5G52190	1041.9311	-1.705435	-1.815075	0.3280297	-5.199027	2.00E-07	1.04E-05	NA	NA
	AT5G65080	12.489255	-1.706315	-2.426399	0.6237123	-2.735741	0.006224	0.0306875	AGL68	AGAMOUS-like 68
	AT4G21740	834.19709	-1.706398	-1.86134	0.3802971	-4.487012	7.22E-06	0.0001757	NA	NA
	AT4G15800	4819.6951	-1.706489	-1.80952	0.3191297	-5.347321	8.93E-08	5.22E-06	RALFL33	ralf-like 33
	AT1G68945	1744.1956	-1.706604	-1.826158	0.3405916	-5.010706	5.42E-07	2.29E-05	NA	NA
	AT3G13275	126.54975	-1.70688	-1.885373	0.4024509	-4.241212	2.22E-05	0.0004285	NA	NA
	AT1G11100	6.8349283	-1.70753	-2.694554	0.6556399	-2.604371	0.0092043	0.041159	NA	NA
	AT5G24570	604.91165	-1.708052	-1.815482	0.3248373	-5.258177	1.45E-07	7.99E-06	NA	NA
	ATCG00640	263.30882	-1.708657	-1.83809	0.3520401	-4.853585	1.21E-06	4.29E-05	RPL33	ribosomal protein L33
	AT5G48490	7986.9469	-1.709627	-1.805975	0.3095064	-5.523722	3.32E-08	2.35E-06	NA	NA
	AT1G64780	17.240881	-1.710233	-2.372905	0.6139011	-2.785844	0.0053389	0.0274342	AMT1;2	ammonium transporter 1;2
	AT2G41410	7935.2419	-1.711072	-1.863577	0.3774318	-4.533462	5.80E-06	0.0001462	NA	NA
	AT2G26020	509.83746	-1.712716	-1.921194	0.4279755	-4.001901	6.28E-05	0.0009629	PDF1.2b	plant defensin 1.2b
	AT2G18328	2874.7435	-1.713311	-1.832948	0.3401371	-5.037119	4.73E-07	2.04E-05	ATRL4	RAD-like 4
	AT2G15050	2495.8136	-1.713527	-1.825817	0.3308358	-5.179386	2.23E-07	1.14E-05	LTP	lipid transfer protein
	AT5G14550	821.07287	-1.713594	-1.866441	0.3774803	-4.539559	5.64E-06	0.0001433	NA	NA
	AT1G44830	70.89654	-1.714466	-1.949088	0.4470313	-3.835225	0.0001254	0.0016647	NA	NA
	AT4G35060	95.30427	-1.716348	-1.843347	0.3483823	-4.926623	8.37E-07	3.24E-05	HIPP25	heavy metal associated isoprenylated plant protein 25
	AT2G18010	46.836467	-1.716875	-2.000817	0.4786281	-3.587075	0.0003344	0.0034738	SAUR10	SMALL AUXIN UPREGULATED RNA 10
	AT1G61890	1346.0219	-1.71735	-1.869035	0.3759663	-4.56783	4.93E-06	0.0001287	NA	NA
	AT1G78020	9321.6925	-1.717392	-1.810382	0.3040149	-5.64904	1.61E-08	1.28E-06	NA	NA
	AT3G25290	63.048577	-1.717884	-1.905773	0.409777	-4.19224	2.76E-05	0.000509	NA	NA
	AT1G53887	9.6888553	-1.718556	-2.574086	0.6414308	-2.679254	0.0073786	0.0350012	NA	NA
	AT1G18265	111.02137	-1.718783	-1.866031	0.3707718	-4.635689	3.56E-06	9.86E-05	NA	NA
	AT4G15630	1048.4808	-1.718976	-1.82335	0.319879	-5.373832	7.71E-08	4.60E-06	NA	NA
	AT5G41761	585.21995	-1.719148	-1.834828	0.3345884	-5.138099	2.78E-07	1.36E-05	NA	NA
	AT1G76960	5029.7375	-1.720397	-1.878763	0.3825259	-4.497464	6.88E-06	0.0001688	NA	NA
	AT3G47500	192.07927	-1.72095	-1.861318	0.363321	-4.736721	2.17E-06	6.62E-05	CDF3	cycling DOF factor 3
	AT3G13175	132.23599	-1.722099	-1.965355	0.4527351	-3.803768	0.0001425	0.0018278	NA	NA
	AT2G35960	1365.4951	-1.722547	-1.819881	0.3098383	-5.559503	2.71E-08	1.97E-06	NHL12	NDR1/HIN1-like 12
	AT4G37800	7098.0318	-1.722743	-1.842185	0.3390981	-5.080369	3.77E-07	1.72E-05	XTH7	xyloglucan endotransglucosylase/hydrolase 7
	AT2G17710	457.77485	-1.723473	-1.8662	0.3658131	-4.711348	2.46E-06	7.31E-05	NA	NA
	AT5G67140	424.52142	-1.725445	-1.833784	0.3246049	-5.315525	1.06E-07	6.10E-06	NA	NA
	AT2G28710	119.65733	-1.725693	-1.902829	0.3993918	-4.320801	1.55E-05	0.0003243	NA	NA
	AT2G21970	3735.9775	-1.72814	-1.852938	0.345128	-5.007244	5.52E-07	2.31E-05	42249	stress enhanced protein 2
	AT2G31940	41.333761	-1.730529	-2.017663	0.4790357	-3.612525	0.0003032	0.0032277	NA	NA
	AT4G35783	95.631418	-1.730691	-1.944114	0.4295875	-4.028727	5.61E-05	0.000887	DVL17	DEVIL 17
	AT2G18970	7.1136987	-1.731258	-2.746127	0.6566109	-2.636658	0.0083727	0.0383054	NA	NA
	AT3G52630	294.84095	-1.73187	-1.935846	0.4223905	-4.100163	4.13E-05	0.0006973	NA	NA
	AT5G56490	5.2348475	-1.734601	-3.437002	0.6858052	-2.52929	0.0114293	0.0482927	AtGulLO4	0

Supplemental Table 3-RNA seq data comparing *Pro35S::MYB63 Pro35S::LAC17* with wild type.

DAP.MYB63.target(1.5kb upstream TSS)	Gene	baseMean	log2FoldChange	lfcMLE	lfcSE	stat	pvalue	padj (FDR)	TAIR10 Symbol	TAIR10 Annotation (Short)
	AT5G4695C	6.1431132	-1.734974	-3.615527	0.6876881	-2.522909	0.0116389	0.0489141	NA	NA
	AT5G4096C	219.51238	-1.737	-1.947289	0.4268502	-4.069344	4.71E-05	0.0007745	NA	NA
	AT3G1664C	106480.81	-1.737312	-1.834655	0.3087122	-5.62761	1.83E-08	1.40E-06	TCTP	translationally controlled tumor protein
	AT4G1298C	3032.8321	-1.737771	-1.887972	0.3725975	-4.663938	3.10E-06	8.82E-05	NA	NA
	AT5G4910C	662.31771	-1.739157	-1.85368	0.3314877	-5.246521	1.55E-07	8.40E-06	NA	NA
	AT3G1352C	4245.828	-1.740101	-1.845744	0.3199071	-5.439394	5.35E-08	3.47E-06	AGP12	arabinogalactan protein 12
	ATCG0105C	67.425528	-1.740405	-1.964352	0.4363939	-3.988152	6.66E-05	0.0010067	NDHD	0
	AT1G5029C	478.08165	-1.740501	-1.914472	0.3955696	-4.399986	1.08E-05	0.0002444	NA	NA
	AT2G3847C	839.79004	-1.741238	-1.863433	0.3408522	-5.108485	3.25E-07	1.53E-05	ATWRKY33	WRKY DNA-BINDING PROTEIN 33
	AT5G1013C	5.7722511	-1.741488	-4.097685	0.6886592	-2.52881	0.011445	0.0483475	NA	NA
	AT4G1027C	85.668667	-1.741655	-1.934217	0.4113244	-4.234261	2.29E-05	0.0004391	NA	NA
	AT4G37608	109.77432	-1.742038	-1.930036	0.4075142	-4.274791	1.91E-05	0.0003772	NA	NA
	AT5G0237C	1001.6443	-1.742771	-1.891684	0.3707447	-4.70073	2.59E-06	7.60E-05	NA	NA
	AT1G6548C	65.382121	-1.745657	-1.931739	0.4054227	-4.30577	1.66E-05	0.0003408	FT	FLOWERING LOCUS T
	AT3G0318C	253.60206	-1.745695	-2.022655	0.4727445	-3.692682	0.0002219	0.0025632	NA	NA
	AT3G1871C	163.92576	-1.748634	-1.886982	0.3585661	-4.876742	1.08E-06	3.92E-05	ATPUB29	ARABIDOPSIS THALIANA PLANT U-BOX 29
	AT1G6134C	242.08093	-1.749153	-2.017453	0.4669246	-3.746115	0.0001796	0.0021809	AtFBS1	0
	AT2G4508C	23.720202	-1.749158	-2.388112	0.6054168	-2.88918	0.0038625	0.0215431	cycp3;1	cyclin p3;1
	AT5G0155C	9.2420195	-1.749963	-2.666456	0.6460488	-2.708717	0.0067544	0.0326865	LecRK-VI.3	L-type lectin receptor kinase VI.3
	AT2G4053C	392.4118	-1.750937	-1.861506	0.3253387	-5.38189	7.37E-08	4.46E-06	NA	NA
	AT5G6046C	779.72306	-1.751268	-1.964033	0.4277125	-4.094498	4.23E-05	0.0007126	NA	NA
	AT2G3775C	100.6207	-1.751714	-2.066368	0.4932848	-3.551122	0.0003836	0.0038447	NA	NA
	AT1G78476	50.023505	-1.752174	-2.011356	0.4599226	-3.809715	0.0001391	0.0017986	NA	NA
	AT5G6665C	184.09956	-1.752954	-1.880344	0.3456926	-5.070847	3.96E-07	1.79E-05	NA	NA
	AT3G1869C	1476.7857	-1.754427	-1.90224	0.3685761	-4.760012	1.94E-06	6.07E-05	MKS1	MAP kinase substrate 1
	AT2G33855	1303.6163	-1.755561	-1.87248	0.3331537	-5.269522	1.37E-07	7.58E-06	NA	NA
	AT3G21351	29.704982	-1.757072	-2.190048	0.5450185	-3.223875	0.0012647	0.0094804	NA	NA
	AT3G0382C	969.27543	-1.757282	-1.848885	0.2988012	-5.881108	4.08E-09	3.99E-07	SAUR29	SMALL AUXIN UP RNA 29
	AT1G16515	50.883327	-1.758558	-2.018626	0.4595732	-3.826503	0.00013	0.0017135	NA	NA
	AT3G1903C	3501.563	-1.758692	-1.871311	0.3275066	-5.369943	7.88E-08	4.67E-06	NA	NA
	AT5G57123	25.56025	-1.76269	-2.172645	0.5352274	-3.293348	0.00099	0.0078803	NA	NA
	AT5G09225	1120.623	-1.763683	-1.939008	0.3947682	-4.467642	7.91E-06	0.0001873	NA	NA
	AT2G3593C	47.906296	-1.764358	-1.98745	0.4331782	-4.073053	4.64E-05	0.0007636	AtPUB23	0
	AT1G2946C	933.60599	-1.764992	-1.874708	0.3231886	-5.461182	4.73E-08	3.16E-06	SAUR65	SMALL AUXIN UPREGULATED RNA 65
	ATCG0068C	1498.9131	-1.766039	-1.879024	0.3273462	-5.39502	6.85E-08	4.24E-06	PSBB	photosystem II reaction center protein B
	AT3G1642C	104.56322	-1.76605	-1.915218	0.3687442	-4.789363	1.67E-06	5.41E-05	JAL30	JACALIN-RELATED LECTIN 30
	AT2G0248C	16.12603	-1.766314	-2.273971	0.5683643	-3.107715	0.0018854	0.0127064	STI	STICHEL
	AT4G3460C	174.34217	-1.7672	-1.902622	0.3536915	-4.996444	5.84E-07	2.42E-05	NA	NA
	AT1G1460C	234.73436	-1.767436	-1.923145	0.3752702	-4.70977	2.48E-06	7.35E-05	NA	NA
	AT3G5629C	2741.7377	-1.767559	-1.904526	0.3557331	-4.968778	6.74E-07	2.69E-05	NA	NA
	AT2G0130C	183.29729	-1.767672	-1.898372	0.348493	-5.072332	3.93E-07	1.78E-05	NA	NA
	AT3G2560C	2017.2798	-1.768172	-1.886634	0.3340424	-5.293255	1.20E-07	6.77E-06	NA	NA
YES	AT4G0297C	3525.1231	-1.76901	-1.888832	0.3356534	-5.270349	1.36E-07	7.57E-06	AT7SL-1	7SL RNA1
	AT5G1760C	451.7063	-1.769087	-1.914933	0.3650899	-4.845619	1.26E-06	4.40E-05	NA	NA
	AT1G53541	125.02476	-1.769463	-1.981544	0.4247978	-4.165424	3.11E-05	0.0005573	NA	NA
	AT1G7619C	41.485206	-1.769486	-2.033752	0.4611903	-3.836781	0.0001247	0.0016567	SAUR56	SMALL AUXIN UPREGULATED RNA 56
	AT1G7581C	548.84483	-1.77011	-1.928857	0.3782561	-4.679659	2.87E-06	8.27E-05	NA	NA

Supplemental Table 3-RNA seq data comparing *Pro35S::MYB63 Pro35S::LAC17* with wild type.

DAP.MYB63.target(1.5kb upstream TSS)	Gene	baseMean	log2FoldChange	lfcMLE	lfcSE	stat	pvalue	padj (FDR)	TAIR10 Symbol	TAIR10 Annotation (Short)
	AT1G55265	95.566431	-1.770112	-1.958617	0.4053046	-4.367362	1.26E-05	0.0002751	NA	NA
	AT4G22470	36.787909	-1.770669	-2.072516	0.4834511	-3.66256	0.0002497	0.002795	NA	NA
	AT4G15920	2798.275	-1.772095	-1.898875	0.3437274	-5.155525	2.53E-07	1.26E-05	AtSWEET17	0
	AT4G01895	62.563367	-1.773059	-2.007057	0.4404178	-4.025857	5.68E-05	0.0008919	NA	NA
	AT1G47400	249.3621	-1.774384	-2.020825	0.4500152	-3.942941	8.05E-05	0.0011764	NA	NA
	AT5G15150	34.555893	-1.774501	-2.058146	0.4727205	-3.753806	0.0001742	0.0021323	ATHB-3	homeobox 3
	AT4G24380	216.77851	-1.77476	-1.966097	0.407678	-4.353337	1.34E-05	0.0002877	NA	NA
	AT5G20635	122.83548	-1.775786	-2.049766	0.4676902	-3.796927	0.0001465	0.0018673	AGG3	Arabidopsis G protein gamma subunit 3
	AT1G11340	48.670053	-1.776278	-1.994751	0.4286929	-4.143474	3.42E-05	0.0005974	NA	NA
YES	AT1G02205	91.520112	-1.778909	-2.04136	0.459958	-3.867546	0.0001099	0.0014975	CER1	ECERIFERUM 1
	AT1G14200	1109.8537	-1.779545	-1.911529	0.3490627	-5.098066	3.43E-07	1.60E-05	NA	NA
	AT3G16900	11.072732	-1.779659	-2.465234	0.6098101	-2.918382	0.0035185	0.0202013	NA	NA
	AT5G43620	299.1335	-1.780289	-2.008953	0.4368596	-4.075197	4.60E-05	0.0007573	NA	NA
	AT1G29440	867.05264	-1.784964	-1.882658	0.3054137	-5.844412	5.08E-09	4.85E-07	SAUR63	SMALL AUXIN UP RNA 63
	AT1G72910	548.81708	-1.785	-1.875248	0.2947427	-6.056132	1.39E-09	1.57E-07	NA	NA
	AT4G11521	236.73657	-1.785393	-1.885732	0.3089089	-5.779674	7.48E-09	6.85E-07	NA	NA
	AT5G59080	4963.8569	-1.785728	-1.943573	0.3761061	-4.747938	2.06E-06	6.34E-05	NA	NA
	AT1G55260	584.41549	-1.786625	-1.9472	0.3786151	-4.718843	2.37E-06	7.11E-05	LTPG6	glycosylphosphatidylinositol-anchored lipid protein transfer 6
	AT3G27860	6.6847064	-1.786774	-3.807304	0.6882115	-2.596257	0.0094245	0.0418718	NA	NA
	AT2G29995	270.70189	-1.786996	-1.920798	0.3502834	-5.101571	3.37E-07	1.58E-05	NA	NA
	AT3G44020	1492.9431	-1.791736	-2.007568	0.4263259	-4.202739	2.64E-05	0.0004904	NA	NA
	AT2G45830	44.368934	-1.791906	-2.074923	0.4705991	-3.807713	0.0001403	0.001808	DTA2	downstream target of AGL15 2
	AT5G11140	25.233164	-1.792476	-2.193921	0.5285293	-3.391442	0.0006953	0.006044	NA	NA
	AT1G71340	1468.4116	-1.792899	-1.944943	0.3695898	-4.85105	1.23E-06	4.30E-05	AtGDPD4	0
	AT2G22880	455.21173	-1.793849	-2.769572	0.6541954	-2.742069	0.0061054	0.0302099	NA	NA
	AT3G04720	13388.09	-1.794171	-1.926142	0.3478829	-5.1574	2.50E-07	1.25E-05	AtPR4	0
	AT2G12462	34.264948	-1.794497	-2.12061	0.4938301	-3.633835	0.0002792	0.0030406	NA	NA
	AT3G05935	913.48666	-1.795219	-1.92381	0.3438515	-5.220914	1.78E-07	9.38E-06	NA	NA
	AT5G17870	22171.373	-1.795351	-1.932006	0.3530417	-5.085379	3.67E-07	1.68E-05	PSRP6	plastid-specific 50S ribosomal protein 6
	AT4G18980	29.678941	-1.796795	-2.271305	0.555711	-3.233326	0.0012236	0.0092809	AtS40-3	AtS40-3
	ATCG00330	366.49285	-1.797083	-1.961741	0.3815874	-4.709494	2.48E-06	7.35E-05	RPS14	chloroplast ribosomal protein S14
	AT3G14770	575.03622	-1.797397	-1.94749	0.3671073	-4.896108	9.78E-07	3.62E-05	AtSWEET2	0
	AT1G52270	172.77949	-1.797487	-1.950541	0.3697717	-4.861073	1.17E-06	4.19E-05	NA	NA
	AT2G43290	2462.7154	-1.79872	-1.933347	0.3504725	-5.132271	2.86E-07	1.39E-05	MSS3	multicopy suppressors of snf4 deficiency in yeast 3
	AT2G01008	24.040302	-1.80211	-2.352616	0.5782467	-3.116508	0.0018301	0.0124458	NA	NA
	AT5G26790	30.066934	-1.802265	-2.155031	0.5066986	-3.556878	0.0003753	0.0037804	NA	NA
	AT2G34490	60.87453	-1.803437	-2.029989	0.4323126	-4.171604	3.02E-05	0.0005478	CYP710A2	cytochrome P450, family 710, subfamily A, polypeptide 2
	AT1G72230	404.12828	-1.804288	-2.007872	0.4153229	-4.344302	1.40E-05	0.0002973	NA	NA
	AT5G12940	1459.7226	-1.80515	-1.946599	0.3574207	-5.050492	4.41E-07	1.93E-05	NA	NA
	AT5G59350	368.37608	-1.805738	-1.943077	0.3526925	-5.119865	3.06E-07	1.46E-05	NA	NA
	AT2G17660	19.516067	-1.806439	-2.578934	0.6251786	-2.889477	0.0038588	0.0215294	NA	NA
	AT5G67060	8.2131021	-1.807	-3.208188	0.6760263	-2.672974	0.0075182	0.0354118	HEC1	HECATE 1
	AT1G01840	82.498432	-1.80706	-2.137885	0.4965347	-3.639342	0.0002733	0.0029889	NA	NA
YES	AT1G55330	22646.734	-1.808573	-1.918816	0.320482	-5.64329	1.67E-08	1.31E-06	AGP21	arabinogalactan protein 21
	AT3G04980	8.6566359	-1.809823	-3.094787	0.6717022	-2.694383	0.0070519	0.0337716	NA	NA
	ATMG0137	323.01527	-1.809991	-1.935659	0.3390556	-5.338332	9.38E-08	5.47E-06	ORF111D	0

Supplemental Table 3-RNA seq data comparing *Pro35S::MYB63 Pro35S::LAC17* with wild type.

DAP.MYB63.target(1.5kb upstream TSS)	Gene	baseMean	log2FoldChange	lfcMLE	lfcSE	stat	pvalue	padj (FDR)	TAIR10 Symbol	TAIR10 Annotation (Short)
	AT5G0323C	704.70651	-1.810634	-1.958868	0.3640667	-4.973359	6.58E-07	2.64E-05	NA	NA
	AT4G1674C	20.514296	-1.810822	-2.490805	0.6082893	-2.976909	0.0029117	0.0175491	ATTPS03	terpene synthase 03
	AT5G6202C	383.92843	-1.810975	-1.935124	0.3372432	-5.369937	7.88E-08	4.67E-06	AT-HSFB2A	ARABIDOPSIS THALIANA HEAT SHOCK TRANSCRIPTION FACTOR B2A
	AT2G1931C	2272.6941	-1.811107	-1.924836	0.3246806	-5.578119	2.43E-08	1.79E-06	NA	NA
	AT2G31085	6.3501943	-1.811197	-3.615352	0.6853137	-2.642874	0.0082206	0.0378472	AtCLE6	0
	AT3G0866C	70.968991	-1.812003	-1.981502	0.3843217	-4.714808	2.42E-06	7.19E-05	NA	NA
	AT2G2840C	1025.3186	-1.812148	-1.959861	0.3634122	-4.98648	6.15E-07	2.51E-05	NA	NA
	AT1G3026C	1176.0002	-1.81225	-1.986351	0.3892667	-4.655549	3.23E-06	9.13E-05	NA	NA
	AT5G1155C	298.11149	-1.81267	-1.958787	0.3615167	-5.01407	5.33E-07	2.26E-05	NA	NA
YES	AT4G2902C	814.5587	-1.813167	-2.499053	0.6120325	-2.962533	0.0030512	0.0181351	NA	NA
	AT1G24148	1521.0737	-1.81425	-1.972503	0.3739863	-4.851112	1.23E-06	4.30E-05	NA	NA
	AT2G2200C	54.623926	-1.815475	-2.089415	0.4632214	-3.919239	8.88E-05	0.0012725	PROPEP6	elicitor peptide 6 precursor
	ATMG0117	328.60108	-1.816775	-1.957941	0.3559437	-5.104108	3.32E-07	1.56E-05	ATP6-2	0
	AT1G44414	27.191722	-1.817522	-2.263239	0.5436333	-3.343287	0.0008279	0.0068819	NA	NA
YES	AT1G6682C	1616.7251	-1.8201	-1.925264	0.3129153	-5.816589	6.01E-09	5.64E-07	NA	NA
	ATCG0022C	7441.4399	-1.820201	-1.960573	0.355014	-5.127124	2.94E-07	1.42E-05	PSBM	photosystem II reaction center protein M
	AT1G1116C	5.2222185	-1.820953	-4.000728	0.6882944	-2.645603	0.0081546	0.0376503	NA	NA
	AT1G4950C	18165.667	-1.821015	-1.932524	0.3211258	-5.670722	1.42E-08	1.15E-06	NA	NA
	AT4G0183C	10.350194	-1.821762	-2.86734	0.6548895	-2.781786	0.0054061	0.0276691	ABCB5	ATP-binding cassette B5
	AT1G4970C	111.03562	-1.821935	-2.104816	0.4684994	-3.888874	0.0001007	0.0013929	NA	NA
	AT5G0876C	2181.6967	-1.822302	-1.993267	0.3855073	-4.727024	2.28E-06	6.88E-05	NA	NA
	AT2G4261C	1669.0875	-1.824513	-1.903953	0.2753727	-6.625612	3.46E-11	6.23E-09	LSH10	LIGHT SENSITIVE HYPOCOTYLS 10
	AT2G2454C	96.704332	-1.826265	-1.983234	0.371217	-4.91967	8.67E-07	3.32E-05	AFR	ATTENUATED FAR-RED RESPONSE
	AT1G1064C	19.181663	-1.826401	-2.298783	0.5516231	-3.310958	0.0009298	0.0075271	NA	NA
	AT2G2121C	1823.9187	-1.826559	-1.950436	0.3358094	-5.439274	5.35E-08	3.47E-06	SAUR6	SMALL AUXIN UPREGULATED RNA 6
	AT5G1558C	50.140115	-1.828245	-2.079164	0.4473505	-4.086829	4.37E-05	0.0007317	LNG1	LONGIFOLIA1
	AT1G6650C	496.48651	-1.829974	-2.033769	0.4131752	-4.429051	9.46E-06	0.0002187	NA	NA
	AT2G1745C	9522.9991	-1.82999	-1.959082	0.3416448	-5.356409	8.49E-08	4.98E-06	RHA3A	RING-H2 finger A3A
	AT1G6923C	79.701316	-1.83143	-2.096342	0.4560761	-4.015625	5.93E-05	0.0009179	SP1L2	SPIRAL1-like2
	AT1G0120C	88.190984	-1.832917	-2.067021	0.4356476	-4.207339	2.58E-05	0.0004825	ATRAB-A3	ARABIDOPSIS RAB GTPASE HOMOLOG A3
	AT5G2227C	1650.0966	-1.83329	-1.993616	0.3743841	-4.896817	9.74E-07	3.62E-05	NA	NA
	AT1G65295	813.29787	-1.83374	-1.998804	0.3788682	-4.840048	1.30E-06	4.51E-05	NA	NA
	AT3G51325	32.755713	-1.835891	-2.128173	0.4715964	-3.892928	9.90E-05	0.0013765	NA	NA
	AT3G63088	86.883827	-1.838084	-2.085397	0.4438776	-4.14097	3.46E-05	0.0006022	DVL14	DEVIL 14
	AT2G2995C	43.569279	-1.83836	-2.101307	0.4534115	-4.054508	5.02E-05	0.0008127	ELF4-L1	ELF4-like 1
YES	AT2G3385C	52.637774	-1.83877	-2.280132	0.5418982	-3.393202	0.0006908	0.0060169	NA	NA
	AT1G4501C	261.07593	-1.841258	-1.974959	0.3457079	-5.326052	1.00E-07	5.80E-06	NA	NA
	AT2G35345	18.03589	-1.844502	-2.537668	0.6076275	-3.03558	0.0024007	0.0151749	NA	NA
	AT4G1165C	26.08416	-1.845011	-2.597263	0.6190929	-2.980185	0.0028807	0.0174208	ATOSM34	osmotin 34
	AT1G29465	1185.0913	-1.845716	-1.995565	0.3628068	-5.087325	3.63E-07	1.67E-05	NA	NA
	AT5G2102C	25848.498	-1.845984	-2.030305	0.3955356	-4.667049	3.06E-06	8.71E-05	NA	NA
	AT5G6604C	15182.464	-1.84605	-1.995175	0.3621055	-5.0981	3.43E-07	1.60E-05	STR16	sulfurtransferase protein 16
	AT5G01015	915.78799	-1.846908	-1.978126	0.3426207	-5.390533	7.02E-08	4.31E-06	NA	NA
	AT1G73325	232.89046	-1.84697	-2.648335	0.6294094	-2.934449	0.0033414	0.0194332	NA	NA
	AT3G4962C	57.434741	-1.847495	-2.103443	0.4485409	-4.118899	3.81E-05	0.0006534	DIN11	DARK INDUCIBLE 11
	AT3G2256C	11.474164	-1.848026	-2.82617	0.6469668	-2.856447	0.0042841	0.0232402	NA	NA

Supplemental Table 3-RNA seq data comparing *Pro35S::MYB63 Pro35S::LAC17* with wild type.

DAP.MYB63.target(1.5kb upstream TSS)	Gene	baseMean	log2FoldChange	lfcMLE	lfcSE	stat	pvalue	padj (FDR)	TAIR10 Symbol	TAIR10 Annotation (Short)
	AT5G03850	6341.7042	-1.848573	-1.995536	0.3596454	-5.139988	2.75E-07	1.35E-05	NA	NA
	AT3G28857	69.731701	-1.849524	-2.278541	0.5359385	-3.451002	0.0005585	0.0051195	PRE5	Paclobutrazol Resistance 5
	AT3G58850	1657.0423	-1.850767	-1.977443	0.3371514	-5.489426	4.03E-08	2.77E-06	HLH2	0
	AT1G52905	335.03324	-1.850814	-2.032461	0.3925202	-4.715207	2.41E-06	7.19E-05	NA	NA
	ATCG00170	51.49888	-1.852012	-2.265184	0.5294445	-3.498028	0.0004687	0.0044762	RPOC2	0
	AT4G38825	145.73499	-1.852243	-2.055806	0.4105589	-4.511515	6.44E-06	0.0001598	SAUR13	SMALL AUXIN UPREGULATED RNA 13
	AT3G05937	262.21741	-1.853029	-2.003301	0.3623857	-5.113417	3.16E-07	1.50E-05	NA	NA
	AT1G12845	1135.477	-1.853853	-1.987149	0.3444135	-5.382637	7.34E-08	4.46E-06	NA	NA
	AT1G70000	352.09898	-1.853878	-2.025703	0.383324	-4.836319	1.32E-06	4.58E-05	NA	NA
	AT1G08180	317.1028	-1.853934	-1.981272	0.337446	-5.494016	3.93E-08	2.72E-06	NA	NA
	AT1G53170	440.63007	-1.85752	-1.949685	0.292136	-6.358408	2.04E-10	3.00E-08	ATERF-8	0
	AT2G21800	9.5316657	-1.85778	-2.75812	0.6372318	-2.915392	0.0035524	0.0203361	ATEME1A	essential meiotic endonuclease 1A
	AT1G19210	7.1389508	-1.858129	-3.912304	0.6877485	-2.701756	0.0068974	0.0331817	NA	NA
	AT4G29905	10119.355	-1.858156	-1.993715	0.346645	-5.360399	8.30E-08	4.89E-06	NA	NA
	AT1G59970	409.07656	-1.858323	-2.023572	0.3768433	-4.931288	8.17E-07	3.17E-05	NA	NA
	AT4G02075	1027.9699	-1.860118	-2.001153	0.3523716	-5.278853	1.30E-07	7.28E-06	PIT1	pitchoun 1
	AT2G18340	13.235793	-1.860141	-2.500281	0.594289	-3.130028	0.0017479	0.0120639	NA	NA
	AT4G29700	34.069214	-1.860539	-2.190975	0.4907692	-3.791067	0.00015	0.0018996	NA	NA
YES	AT5G35480	8368.8647	-1.861062	-2.01485	0.3655967	-5.090477	3.57E-07	1.64E-05	NA	NA
	AT3G09220	6.0638433	-1.862972	-5.234363	0.6866657	-2.71307	0.0066663	0.0324159	LAC7	laccase 7
	AT3G47965	231.73196	-1.865954	-2.049272	0.3925746	-4.753119	2.00E-06	6.22E-05	NA	NA
	AT1G62370	44.097877	-1.867467	-2.161944	0.4700952	-3.97253	7.11E-05	0.0010662	NA	NA
	AT1G62770	28.747659	-1.867853	-2.180537	0.4798154	-3.892857	9.91E-05	0.0013765	NA	NA
	AT1G52618	364.37326	-1.868138	-2.07489	0.4119999	-4.534316	5.78E-06	0.000146	NA	NA
	AT3G52360	1011.0599	-1.868829	-2.033234	0.3752799	-4.979827	6.36E-07	2.58E-05	NA	NA
	AT4G37230	11.418903	-1.87091	-2.706384	0.6285289	-2.97665	0.0029142	0.0175581	NA	NA
	AT4G12970	478.82646	-1.871494	-2.112265	0.4371704	-4.280926	1.86E-05	0.0003698	EPFL9	0
	AT2G15020	54.659578	-1.871977	-2.14852	0.4592226	-4.076404	4.57E-05	0.0007569	NA	NA
	ATCG00300	162.16661	-1.872263	-2.039164	0.3770576	-4.965458	6.85E-07	2.73E-05	YCF9	0
	AT2G38480	631.0896	-1.875015	-2.003478	0.3372282	-5.560076	2.70E-08	1.97E-06	NA	NA
	AT2G25820	13.417736	-1.875314	-2.945195	0.6550137	-2.863015	0.0041963	0.0228671	ESE2	ethylene and salt inducible 2
	AT2G42540	2400.9407	-1.875362	-2.057227	0.3908018	-4.798754	1.60E-06	5.27E-05	COR15	0
	AT4G25470	600.97065	-1.875612	-2.049609	0.3835744	-4.889825	1.01E-06	3.72E-05	ATCBF2	0
	AT3G57770	319.81771	-1.877422	-2.025381	0.3580325	-5.24372	1.57E-07	8.49E-06	NA	NA
	AT1G76955	495.50507	-1.878432	-2.012254	0.3429312	-5.477577	4.31E-08	2.91E-06	NA	NA
	AT1G20190	2233.9788	-1.878733	-2.090166	0.4150285	-4.526758	5.99E-06	0.0001501	ATEXP11	0
	AT1G11112	22.324878	-1.880684	-2.37201	0.5541281	-3.393951	0.0006889	0.0060063	NA	NA
	AT5G67390	663.15508	-1.882573	-2.06027	0.3863597	-4.872591	1.10E-06	3.99E-05	NA	NA
	AT4G23420	195.03499	-1.883465	-2.063069	0.3876552	-4.85861	1.18E-06	4.20E-05	NA	NA
	AT2G31945	126.89376	-1.883664	-2.182625	0.4721625	-3.989441	6.62E-05	0.0010038	NA	NA
YES	AT3G17130	94.465249	-1.884269	-2.08091	0.4019588	-4.687717	2.76E-06	7.98E-05	NA	NA
	AT2G26975	2841.4313	-1.886422	-1.983589	0.2972355	-6.346556	2.20E-10	3.20E-08	COPT6	copper transporter 6
	AT4G11910	46.488045	-1.88709	-2.274584	0.51508	-3.663683	0.0002486	0.002788	NA	NA
	AT4G08685	3015.5563	-1.887251	-2.02132	0.3426607	-5.507637	3.64E-08	2.55E-06	SAH7	0
	AT1G69690	1201.8753	-1.891775	-2.031916	0.3488856	-5.422335	5.88E-08	3.76E-06	AtTCP15	0
	AT5G43580	103.57291	-1.892005	-3.711487	0.6863677	-2.756548	0.0058415	0.0292986	UPI	UNUSUAL SERINE PROTEASE INHIBITOR
	AT2G29170	392.27404	-1.892354	-2.032443	0.3486132	-5.428234	5.69E-08	3.66E-06	NA	NA
	AT5G03130	115.18764	-1.893912	-2.08188	0.3940121	-4.806734	1.53E-06	5.09E-05	NA	NA
	AT4G21620	2268.7845	-1.894912	-2.026261	0.3390107	-5.589535	2.28E-08	1.70E-06	NA	NA
	AT3G05890	113.24543	-1.895604	-2.26697	0.5080248	-3.731322	0.0001905	0.0022899	RCI2B	RARE-COLD-INDUCIBLE 2B

Supplemental Table 3-RNA seq data comparing *Pro35S::MYB63 Pro35S::LAC17* with wild type.

DAP.MYB63.target(1.5kb upstream TSS)	Gene	baseMean	log2FoldChange	lfcMLE	lfcSE	stat	pvalue	padj (FDR)	TAIR10 Symbol	TAIR10 Annotation (Short)
	AT5G5139C	384.65293	-1.898869	-2.051174	0.3607553	-5.263592	1.41E-07	7.79E-06	NA	NA
	AT4G1591C	396.07554	-1.899011	-2.155962	0.4456101	-4.261598	2.03E-05	0.000398	ATDI21	drought-induced 21
	AT5G0188C	153.14312	-1.89964	-2.039973	0.3479759	-5.459114	4.79E-08	3.17E-06	DAFL2	DAF-Like gene 2
	AT4G2978C	189.83799	-1.899971	-2.012073	0.3155873	-6.020429	1.74E-09	1.90E-07	NA	NA
	AT2G3570C	29.388352	-1.900586	-2.490572	0.5818441	-3.266488	0.0010889	0.0084692	ATERF38	ERF FAMILY PROTEIN 38
	AT3G5602C	4061.9181	-1.900637	-2.064399	0.3720713	-5.10826	3.25E-07	1.53E-05	NA	NA
	AT1G6147C	114.67408	-1.90154	-2.074185	0.3795216	-5.010362	5.43E-07	2.29E-05	NA	NA
YES	AT5G4050C	2498.6256	-1.902498	-2.107586	0.4079496	-4.663562	3.11E-06	8.82E-05	NA	NA
	AT3G1066C	8.3129964	-1.90259	-3.018044	0.6555375	-2.902336	0.0037039	0.0209768	ATCPK2	0
YES	AT4G3877C	1544.0883	-1.906017	-2.029255	0.3288559	-5.795905	6.80E-09	6.28E-07	ATPRP4	ARABIDOPSIS THALIANA PROLINE-RICH PROTEIN 4
	AT5G0676C	26.555632	-1.906359	-2.34834	0.533889	-3.570703	0.000356	0.003633	AtLEA4-5	Late Embryogenesis Abundant 4-5
	AT5G4458C	10429.807	-1.907354	-2.078896	0.3788212	-5.03497	4.78E-07	2.06E-05	NA	NA
	AT4G0501C	155.45916	-1.908384	-2.162293	0.4424908	-4.312821	1.61E-05	0.0003324	AtFBS3	0
	AT2G4408C	854.25749	-1.911479	-2.13767	0.4232377	-4.516326	6.29E-06	0.0001566	ARL	ARGOS-like
	AT4G3481C	43.510333	-1.913733	-2.251355	0.4891533	-3.912337	9.14E-05	0.0013001	SAUR5	SMALL AUXIN UPREGULATED RNA 5
YES	AT5G1441C	507.99757	-1.914206	-2.09445	0.3859271	-4.96002	7.05E-07	2.80E-05	NA	NA
YES	AT4G1521C	61.092729	-1.915063	-2.232473	0.4795692	-3.993299	6.52E-05	0.0009918	AT-BETA-AI	0
	AT3G0877C	240.24793	-1.915255	-3.040815	0.6605034	-2.899689	0.0037353	0.0210931	LTP6	lipid transfer protein 6
	AT3G0384C	366.46994	-1.918018	-2.018643	0.2995416	-6.403178	1.52E-10	2.42E-08	SAUR27	SMALL AUXIN UP RNA 27
	AT5G0179C	82.306592	-1.918038	-2.123509	0.4058041	-4.726511	2.28E-06	6.88E-05	NA	NA
	AT1G3464C	536.21912	-1.921349	-2.129482	0.408592	-4.702365	2.57E-06	7.57E-05	NA	NA
	AT1G55675	2975.2789	-1.921985	-2.073637	0.358468	-5.361664	8.25E-08	4.87E-06	NA	NA
	AT4G08555	184.46454	-1.924287	-2.268387	0.4928588	-3.904338	9.45E-05	0.0013282	NA	NA
	AT1G2945C	1190.121	-1.925049	-2.036531	0.3132055	-6.146281	7.93E-10	1.00E-07	SAUR64	SMALL AUXIN UPREGULATED RNA 64
	AT1G30135	88.237349	-1.925856	-2.253207	0.4835213	-3.982981	6.81E-05	0.0010263	JAZ8	jasmonate-zim-domain protein 8
	AT5G4046C	90.978622	-1.926297	-2.228452	0.469915	-4.099246	4.14E-05	0.0006994	NA	NA
	AT1G2623C	547.85842	-1.927327	-2.120377	0.3958337	-4.869031	1.12E-06	4.04E-05	Cpn60beta4	chaperonin-60beta4
	AT2G1965C	53.522592	-1.929852	-2.236605	0.4713767	-4.094075	4.24E-05	0.0007132	NA	NA
	AT2G32765	100.12808	-1.931116	-2.213654	0.4582097	-4.21448	2.50E-05	0.0004738	ATSUMO5	0
	AT1G6193C	22.005749	-1.933288	-2.492155	0.5696506	-3.393814	0.0006893	0.0060064	NA	NA
	AT1G6610C	18127.461	-1.933916	-2.102918	0.3743003	-5.16675	2.38E-07	1.20E-05	NA	NA
	AT2G4351C	1069.3269	-1.936256	-2.178795	0.4327507	-4.474299	7.67E-06	0.0001832	ATTI1	trypsin inhibitor protein 1
	AT1G0207C	6.0357683	-1.937646	-4.22446	0.6878164	-2.817098	0.004846	0.0255199	NA	NA
	AT1G3252C	638.22817	-1.942879	-2.071017	0.3316537	-5.858156	4.68E-09	4.49E-07	NA	NA
YES	AT4G1555C	30.9017	-1.947536	-2.272318	0.4792158	-4.064007	4.82E-05	0.0007903	IAGLU	indole-3-acetate beta-D-glucosyltransferase
YES	AT3G6055C	38.199645	-1.948881	-2.322919	0.5035996	-3.869901	0.0001089	0.0014853	CYCP3;2	cyclin p3;2
	AT2G4778C	93.513411	-1.950138	-2.139265	0.390179	-4.99806	5.79E-07	2.41E-05	NA	NA
	AT2G3795C	423.8123	-1.950511	-2.113269	0.3669159	-5.315962	1.06E-07	6.10E-06	NA	NA
	AT1G07135	2969.2974	-1.950521	-2.124049	0.3770814	-5.172679	2.31E-07	1.18E-05	NA	NA
	AT2G39705	606.50191	-1.952319	-2.106278	0.3582254	-5.449976	5.04E-08	3.30E-06	DVL11	DEVIL 11
	AT5G6532C	7.3598394	-1.95433	-3.496025	0.6759727	-2.891138	0.0038385	0.0214425	NA	NA
	AT5G2258C	3669.2942	-1.954541	-2.100065	0.3496739	-5.589611	2.28E-08	1.70E-06	NA	NA
YES	AT2G3854C	22641.151	-1.956944	-2.092608	0.3390624	-5.771635	7.85E-09	7.04E-07	ATLTP1	ARABIDOPSIS THALIANA LIPID TRANSFER PROTEIN 1
	AT5G6330C	12.618647	-1.957109	-2.722872	0.6114749	-3.200637	0.0013712	0.0100405	NA	NA
	AT4G3356C	55.036442	-1.958128	-2.197956	0.427967	-4.575418	4.75E-06	0.0001254	NA	NA
	AT5G3333C	15.044132	-1.962344	-2.644206	0.5963575	-3.29055	0.0009999	0.0079369	NA	NA

Supplemental Table 3-RNA seq data comparing *Pro35S::MYB63 Pro35S::LAC17* with wild type.

DAP.MYB63.target(1.5kb upstream TSS)	Gene	baseMean	log2FoldChange	lfcMLE	lfcSE	stat	pvalue	padj (FDR)	TAIR10 Symbol	TAIR10 Annotation (Short)
	AT2G06255	245.82291	-1.962894	-2.108925	0.3492384	-5.620498	1.90E-08	1.45E-06	ELF4-L3	ELF4-like 3
	AT5G06790	217.28504	-1.964154	-2.085275	0.3218566	-6.102576	1.04E-09	1.25E-07	NA	NA
	ATCG00630	15350.496	-1.964233	-2.12374	0.3628927	-5.412712	6.21E-08	3.94E-06	PSAJ	0
	AT1G74670	26317.896	-1.965048	-2.125265	0.3635175	-5.405647	6.46E-08	4.02E-06	GASA6	GA-stimulated Arabidopsis 6
	AT1G65490	3440.6433	-1.96514	-2.169463	0.4020394	-4.887927	1.02E-06	3.75E-05	NA	NA
YES	AT1G09500	85.729341	-1.965678	-2.429416	0.5390322	-3.64668	0.0002657	0.0029296	NA	NA
	AT5G09530	8.0248004	-1.967579	-3.247522	0.6631357	-2.967084	0.0030064	0.0179517	PELPK1	Pro-Glu-Leu|Ile|Val-Pro-Lys 1
	AT5G46115	15.843981	-1.9686	-2.669584	0.5987996	-3.287578	0.0010105	0.0080058	NA	NA
	AT1G61165	12.110152	-1.97152	-2.762703	0.6134863	-3.213633	0.0013107	0.0097175	NA	NA
	AT3G23230	42.840985	-1.972128	-2.254245	0.4530824	-4.352691	1.34E-05	0.0002882	AtERF98	0
	ATCG00210	52.101964	-1.972359	-2.230705	0.4388296	-4.49459	6.97E-06	0.0001704	YCF6	0
YES	AT5G26730	5.8086791	-1.973707	-4.119568	0.6858772	-2.877638	0.0040066	0.0221212	NA	NA
	AT2G45450	618.08468	-1.974101	-2.165586	0.3906751	-5.05305	4.35E-07	1.91E-05	ZPR1	LITTLE ZIPPER 1
	AT2G36220	2275.5755	-1.977919	-2.129741	0.3542474	-5.583439	2.36E-08	1.76E-06	NA	NA
	AT1G19670	3041.0692	-1.979248	-2.128634	0.3517188	-5.62736	1.83E-08	1.40E-06	ATCLH1	chlorophyllase 1
	AT3G28925	5.8977515	-1.98481	-4.241667	0.6872074	-2.888225	0.0038742	0.0215885	NA	NA
	AT2G27385	14970.827	-1.985021	-2.15803	0.3738727	-5.309349	1.10E-07	6.27E-06	NA	NA
	AT5G24190	8.5133783	-1.985726	-3.347829	0.6671829	-2.976285	0.0029176	0.0175672	NA	NA
YES	AT1G72240	185.77923	-1.990682	-2.208425	0.4097084	-4.858777	1.18E-06	4.20E-05	NA	NA
	AT5G42210	59.710903	-1.993151	-2.227849	0.4212207	-4.731844	2.22E-06	6.73E-05	NA	NA
	AT3G19550	363.71218	-1.993651	-2.222317	0.4178928	-4.770724	1.84E-06	5.85E-05	NA	NA
	AT4G12880	1336.4153	-1.994363	-2.216125	0.4129738	-4.829273	1.37E-06	4.71E-05	AtENODL19	0
	AT3G43720	5134.8336	-1.995248	-2.159917	0.3653711	-5.46088	4.74E-08	3.16E-06	LTPG2	glycosylphosphatidylinositol-anchored lipid protein transfer 2
	AT5G24105	356.36171	-1.995617	-2.256529	0.4397294	-4.538284	5.67E-06	0.0001439	AGP41	arabinogalactan protein 41
	AT2G42150	7.1658254	-1.996545	-3.470123	0.6716941	-2.972402	0.0029548	0.0177249	NA	NA
	AT1G33055	62.003	-1.997083	-2.257861	0.4382648	-4.556797	5.19E-06	0.0001336	NA	NA
	AT1G25560	10576.995	-2.001046	-2.171353	0.3701526	-5.406003	6.44E-08	4.02E-06	AtTEM1	0
	AT1G12672	13.311766	-2.001497	-2.652908	0.5850218	-3.421235	0.0006234	0.0055862	NA	NA
	AT1G29640	16.654114	-2.002853	-2.639932	0.5823544	-3.439235	0.0005834	0.0053014	NA	NA
	AT1G70270	60.486359	-2.004794	-4.57753	0.6887315	-2.91085	0.0036045	0.0205428	NA	NA
	AT1G02470	110.16197	-2.006431	-2.267969	0.4385669	-4.574972	4.76E-06	0.0001255	NA	NA
	AT2G04800	8.6980019	-2.006912	-3.339077	0.6640432	-3.022261	0.0025089	0.0156774	NA	NA
	AT5G54530	215.07816	-2.009225	-2.232584	0.4125268	-4.870532	1.11E-06	4.03E-05	NA	NA
	AT1G29490	43.833426	-2.010398	-2.488406	0.5387656	-3.731488	0.0001904	0.0022899	SAUR68	SMALL AUXIN UPREGULATED 68
	AT3G11090	816.4765	-2.012008	-2.13115	0.3162669	-6.36174	1.99E-10	2.97E-08	LBD21	LOB domain-containing protein 21
	AT2G20835	133.20036	-2.01358	-2.200577	0.3832231	-5.254327	1.49E-07	8.09E-06	NA	NA
YES	AT3G48020	183.14147	-2.014482	-2.235128	0.4098924	-4.914661	8.89E-07	3.37E-05	NA	NA
	AT1G79770	67.605126	-2.015196	-2.215156	0.3932327	-5.12469	2.98E-07	1.43E-05	NA	NA
	AT3G25700	87.253902	-2.015626	-2.209899	0.3887793	-5.184498	2.17E-07	1.11E-05	NA	NA
	AT4G01080	86.188466	-2.015633	-2.255032	0.4230234	-4.764826	1.89E-06	5.97E-05	TBL26	TRICHOME BIREFRINGENCE-LIKE 26
	AT4G11211	827.08607	-2.015657	-2.21417	0.3931579	-5.126839	2.95E-07	1.42E-05	NA	NA
	AT2G27830	5949.2055	-2.017735	-2.161046	0.3426278	-5.889	3.89E-09	3.83E-07	NA	NA
	AT5G49170	316.1239	-2.019293	-2.207613	0.3842012	-5.255823	1.47E-07	8.07E-06	NA	NA
	AT5G20670	737.45642	-2.021492	-2.156693	0.3336819	-6.058142	1.38E-09	1.56E-07	NA	NA
	AT3G12870	66.561189	-2.022542	-2.350426	0.4753431	-4.254909	2.09E-05	0.0004083	NA	NA
	AT3G17120	1017.0123	-2.022792	-2.182256	0.3582435	-5.646417	1.64E-08	1.29E-06	NA	NA
	AT2G18969	25.41299	-2.023006	-2.444162	0.5159496	-3.920937	8.82E-05	0.0012656	NA	NA
	AT3G22415	169.92731	-2.024024	-2.255457	0.4171108	-4.852486	1.22E-06	4.30E-05	NA	NA

Supplemental Table 3-RNA seq data comparing *Pro35S::MYB63 Pro35S::LAC17* with wild type.

DAP.MYB63.target(1.5kb upstream TSS)	Gene	baseMean	log2FoldChange	lfcMLE	lfcSE	stat	pvalue	padj (FDR)	TAIR10 Symbol	TAIR10 Annotation (Short)
	AT3G56360	16794.871	-2.024459	-2.224	0.3934017	-5.146036	2.66E-07	1.31E-05	NA	NA
	AT3G61900	33.371486	-2.024637	-2.365419	0.4799543	-4.218394	2.46E-05	0.0004667	SAUR33	SMALL AUXIN UPREGULATED RNA 33
	AT2G15830	188.03914	-2.025088	-2.21711	0.3865794	-5.238478	1.62E-07	8.68E-06	NA	NA
	AT2G14247	1398.7488	-2.025322	-2.269964	0.4266749	-4.746757	2.07E-06	6.37E-05	NA	NA
	AT5G25880	6.4361478	-2.026984	-5.37224	0.6884217	-2.944393	0.0032359	0.01897	ATNADP-M	Arabidopsis thaliana NADP-malic enzyme 3
	AT3G16690	189.88523	-2.029714	-2.257711	0.4141458	-4.900964	9.54E-07	3.56E-05	AtSWEET16	0
	AT3G46900	77.196567	-2.033645	-2.220625	0.3814516	-5.331331	9.75E-08	5.65E-06	COPT2	copper transporter 2
	AT5G24165	10503.294	-2.04055	-2.228566	0.3826222	-5.333066	9.66E-08	5.61E-06	NA	NA
	AT3G19660	549.53138	-2.04572	-2.184117	0.3352981	-6.101198	1.05E-09	1.25E-07	NA	NA
	AT1G07610	1882.0693	-2.045859	-2.182773	0.3338471	-6.128133	8.89E-10	1.10E-07	MT1C	metallothionein 1C
	AT1G32928	716.25107	-2.046745	-2.346713	0.4591676	-4.457513	8.29E-06	0.0001951	NA	NA
	AT1G53543	16.505477	-2.049359	-2.851451	0.6111833	-3.3531	0.0007991	0.006695	NA	NA
	AT1G76650	720.66331	-2.049649	-2.234634	0.3792031	-5.405149	6.48E-08	4.02E-06	CML38	calmodulin-like 38
	AT2G42975	813.71427	-2.054632	-2.20062	0.3425764	-5.997587	2.00E-09	2.14E-07	NA	NA
	AT1G09180	51.985363	-2.054766	-2.305545	0.4267174	-4.815285	1.47E-06	4.96E-05	ATSAR1	SECRETION-ASSOCIATED RAS 1
	AT1G77570	70.117771	-2.055008	-2.284039	0.4120977	-4.986701	6.14E-07	2.51E-05	AtREN1	0
	AT1G52827	538.22438	-2.056336	-2.226049	0.3651865	-5.630919	1.79E-08	1.39E-06	ATCDT1	0
	AT3G29035	267.80731	-2.058249	-2.278961	0.406756	-5.060156	4.19E-07	1.87E-05	ANAC059	Arabidopsis NAC domain containing protein 59
	AT2G30432	98.599659	-2.065288	-2.291338	0.409602	-5.042181	4.60E-07	1.99E-05	TCL1	TRICHOMELESS1
	AT5G57760	171.11943	-2.06628	-2.326656	0.4331531	-4.770322	1.84E-06	5.85E-05	NA	NA
	AT5G39610	69.136598	-2.070433	-2.321865	0.4260114	-4.860042	1.17E-06	4.20E-05	ANAC092	Arabidopsis NAC domain containing protein 92
	AT4G16590	10.816114	-2.071208	-3.02118	0.6300176	-3.28754	0.0010107	0.0080058	ATCSLA01	cellulose synthase-like A01
	AT5G41050	1842.376	-2.074086	-2.234334	0.3552086	-5.839065	5.25E-09	4.98E-07	NA	NA
	AT5G37760	12.163196	-2.076125	-2.930488	0.6153248	-3.374031	0.0007408	0.0063267	NA	NA
	AT2G19970	29.492763	-2.077943	-2.569507	0.5374878	-3.866028	0.0001106	0.0015045	NA	NA
	AT5G24770	281.33677	-2.078494	-2.335414	0.4303829	-4.829407	1.37E-06	4.71E-05	ATVSP2	0
	ATMG0067	255.80494	-2.080434	-2.230506	0.3445446	-6.038212	1.56E-09	1.73E-07	ORF275	0
	AT1G48745	402.53847	-2.081551	-2.258601	0.3697927	-5.628968	1.81E-08	1.40E-06	NA	NA
	AT3G04640	2608.2295	-2.081742	-2.265042	0.3754158	-5.545163	2.94E-08	2.13E-06	NA	NA
	AT4G36110	242.00831	-2.082305	-2.251577	0.3627187	-5.740825	9.42E-09	8.16E-07	SAUR9	SMALL AUXIN UPREGULATED RNA 9
	AT1G77870	105.66614	-2.082341	-2.342888	0.4318631	-4.821761	1.42E-06	4.85E-05	MUB5	membrane-anchored ubiquitin-fold protein 5 precursor
	ATCG01020	60877.656	-2.084401	-2.256852	0.3657538	-5.698919	1.21E-08	9.92E-07	RPL32	ribosomal protein L32
	AT4G30130	59.176611	-2.08651	-2.385351	0.4534227	-4.601689	4.19E-06	0.0001132	NA	NA
	AT4G26288	150.11298	-2.087078	-2.273813	0.3772723	-5.532022	3.17E-08	2.27E-06	NA	NA
	AT4G15730	7.7009438	-2.090451	-3.515361	0.6643307	-3.146703	0.0016512	0.0115202	NA	NA
	AT5G23820	2404.8065	-2.095338	-2.237645	0.3360024	-6.236082	4.49E-10	6.13E-08	ML3	MD2-related lipid recognition 3
	AT2G42840	113.12678	-2.099007	-2.451341	0.4815484	-4.358871	1.31E-05	0.0002833	PDF1	protodermal factor 1
	AT3G06895	40.529752	-2.099992	-2.383582	0.44344	-4.735685	2.18E-06	6.63E-05	NA	NA
	AT1G11850	1131.121	-2.105008	-2.303749	0.3863945	-5.447821	5.10E-08	3.33E-06	NA	NA
	AT3G23240	15.112642	-2.107471	-2.819216	0.5890577	-3.577699	0.0003466	0.0035593	ATERF1	ETHYLENE RESPONSE FACTOR 1
	AT5G46295	80.860344	-2.109205	-2.553147	0.5194121	-4.060755	4.89E-05	0.000797	NA	NA
	AT4G27654	114.4667	-2.109847	-6.268977	0.6855445	-3.077622	0.0020866	0.0136886	NA	NA
	AT3G50800	253.12554	-2.110897	-2.244977	0.3260968	-6.473222	9.59E-11	1.59E-08	NA	NA

Supplemental Table 3-RNA seq data comparing *Pro35S::MYB63 Pro35S::LAC17* with wild type.

DAP.MYB63.target(1.5kb upstream TSS)	Gene	baseMean	log2FoldChange	lfcMLE	lfcSE	stat	pvalue	padj (FDR)	TAIR10 Symbol	TAIR10 Annotation (Short)
	AT3G15210	5600.5402	-2.112874	-2.269039	0.3485526	-6.061851	1.35E-09	1.53E-07	ATERF-4	ETHYLENE RESPONSIVE ELEMENT BINDING FACTOR 4
	AT2G32020	31.388762	-2.116258	-2.457205	0.4727725	-4.476271	7.60E-06	0.0001823	NA	NA
	AT3G21260	218.44961	-2.123277	-2.325487	0.3874381	-5.480301	4.25E-08	2.88E-06	GLTP3	GLYCOLIPID TRANSFER PROTEIN 3
	AT2G46650	5110.5435	-2.123641	-2.235877	0.3006928	-7.062493	1.64E-12	3.77E-10	ATCB5-C	ARABIDOPSIS CYTOCHROME B5 ISOFORM C
	AT4G28290	830.18999	-2.1297	-2.315941	0.3742296	-5.690891	1.26E-08	1.04E-06	NA	NA
	AT1G11120	34.808175	-2.134395	-2.499503	0.4826131	-4.422581	9.75E-06	0.0002236	NA	NA
	AT5G24660	1134.2806	-2.136079	-2.266846	0.3209942	-6.654572	2.84E-11	5.22E-09	LSU2	RESPONSE TO LOW SULFUR 2
	AT5G61160	10.715474	-2.137754	-3.41993	0.6558056	-3.259738	0.0011152	0.0086374	AACT1	anthocyanin 5-aromatic acyltransferase 1
	ATCG00730	459.77499	-2.143169	-2.360305	0.3975873	-5.390438	7.03E-08	4.31E-06	PETD	photosynthetic electron transfer D
	AT1G17147	966.7281	-2.145801	-2.352904	0.3898838	-5.503694	3.72E-08	2.59E-06	NA	NA
	AT3G06868	128.39223	-2.152918	-2.333222	0.3667793	-5.869793	4.36E-09	4.23E-07	NA	NA
	AT1G66400	287.35425	-2.159824	-2.352965	0.3774891	-5.721554	1.06E-08	8.97E-07	CML23	calmodulin like 23
	AT3G15760	1210.2947	-2.160616	-2.346151	0.3714262	-5.81708	5.99E-09	5.64E-07	NA	NA
	AT1G10585	62.557255	-2.161701	-2.52089	0.4784789	-4.517861	6.25E-06	0.0001557	NA	NA
	AT1G78410	828.56165	-2.162358	-2.440199	0.4368751	-4.949602	7.44E-07	2.93E-05	NA	NA
	AT2G07798	21.618618	-2.163365	-2.792564	0.5688036	-3.80336	0.0001427	0.0018283	NA	NA
	AT3G55860	39.630985	-2.166854	-2.558249	0.492473	-4.399944	1.08E-05	0.0002444	NA	NA
	AT1G47370	272.45252	-2.167632	-2.332409	0.3523411	-6.152084	7.65E-10	9.77E-08	NA	NA
	AT4G36500	9531.5899	-2.172541	-2.364971	0.3763574	-5.772549	7.81E-09	7.04E-07	NA	NA
	AT5G18020	804.70923	-2.173841	-2.325272	0.3396015	-6.401154	1.54E-10	2.42E-08	SAUR20	SMALL AUXIN UP RNA 20
	AT1G61795	445.16142	-2.174189	-2.352167	0.3638368	-5.975725	2.29E-09	2.41E-07	NA	NA
	AT1G77885	270.03716	-2.175802	-2.446634	0.4313451	-5.044227	4.55E-07	1.98E-05	NA	NA
	AT2G27310	976.6754	-2.176165	-2.395401	0.3967527	-5.484941	4.14E-08	2.82E-06	NA	NA
	AT4G16515	544.85565	-2.177144	-2.372473	0.3781315	-5.757637	8.53E-09	7.54E-07	CLEL 6	CLE-like 6
	AT1G20470	102.60066	-2.185371	-2.380952	0.3770115	-5.796562	6.77E-09	6.28E-07	SAUR60	SMALL AUXIN UPREGULATED RNA 60
	AT5G60220	14.616419	-2.186719	-3.099623	0.6179128	-3.53888	0.0004018	0.0039735	TET4	tetraspanin4
	AT5G64660	1609.3994	-2.186922	-2.407775	0.3972043	-5.505787	3.68E-08	2.57E-06	ATCMPG2	0
	AT3G20490	128.83286	-2.197884	-2.429498	0.403279	-5.450034	5.04E-08	3.30E-06	NA	NA
	AT3G29140	114.47693	-2.200185	-2.388643	0.3703948	-5.940106	2.85E-09	2.91E-07	NA	NA
	AT5G21940	21160.419	-2.202444	-2.387608	0.3682136	-5.98143	2.21E-09	2.34E-07	NA	NA
	AT1G69490	149.25061	-2.206493	-2.497164	0.4402709	-5.011671	5.40E-07	2.29E-05	ANAC029	Arabidopsis NAC domain containing protein 29
	AT2G37130	185.62611	-2.207039	-2.378386	0.3556642	-6.205403	5.46E-10	7.18E-08	NA	NA
	AT3G47295	2478.2359	-2.208412	-2.383031	0.3587215	-6.156341	7.44E-10	9.58E-08	NA	NA
	AT3G14380	8.6857587	-2.208882	-3.393989	0.6430752	-3.434874	0.0005928	0.0053739	NA	NA
	AT3G28500	84.582997	-2.210706	-2.486743	0.4307373	-5.132378	2.86E-07	1.39E-05	NA	NA
	AT5G40630	19.354143	-2.215447	-2.86441	0.5680108	-3.90036	9.60E-05	0.0013461	NA	NA
	AT5G61600	1096.9695	-2.2167	-2.333981	0.3007729	-7.370011	1.71E-13	5.04E-11	ERF104	ethylene response factor 104
	AT1G68840	9147.4108	-2.220637	-2.41334	0.3731376	-5.951255	2.66E-09	2.76E-07	AtRAV2	0
	AT4G26950	12.894251	-2.222909	-3.080823	0.6066009	-3.664533	0.0002478	0.0027805	NA	NA
	AT5G18050	564.26329	-2.224533	-2.413353	0.3694546	-6.021128	1.73E-09	1.90E-07	SAUR22	SMALL AUXIN UP RNA 22
	AT5G59820	851.20364	-2.224737	-2.499876	0.4304295	-5.168643	2.36E-07	1.20E-05	AtZAT12	0
	AT4G23600	476.61019	-2.225893	-2.394807	0.352379	-6.31676	2.67E-10	3.85E-08	CORI3	CORONATINE INDUCED 1
	AT4G28700	16.81056	-2.226633	-2.941267	0.5827016	-3.821223	0.0001328	0.0017391	AMT1;4	ammonium transporter 1;4

Supplemental Table 3-RNA seq data comparing *Pro35S::MYB63 Pro35S::LAC17* with wild type.

DAP.MYB63.target(1.5kb upstream TSS)	Gene	baseMean	log2FoldChange	lfcMLE	lfcSE	stat	pvalue	padj (FDR)	TAIR10 Symbol	TAIR10 Annotation (Short)
	AT3G5524C	2874.7764	-2.228078	-2.380157	0.3367077	-6.617246	3.66E-11	6.53E-09	NA	NA
	AT2G3460C	424.35521	-2.229125	-2.547223	0.4544094	-4.905543	9.32E-07	3.49E-05	JAZ7	jasmonate-zim-domain protein 7
	AT5G5374C	194.77643	-2.232403	-2.455891	0.3951247	-5.649869	1.61E-08	1.28E-06	NA	NA
	AT4G2192C	66.69248	-2.235268	-2.611814	0.48088	-4.648287	3.35E-06	9.34E-05	NA	NA
	AT3G0385C	367.58633	-2.238251	-2.429657	0.3704535	-6.041921	1.52E-09	1.70E-07	SAUR26	SMALL AUXIN UP RNA 26
	AT5G4948C	967.79519	-2.242381	-2.412922	0.3527015	-6.357731	2.05E-10	3.00E-08	ATCP1	Ca2+-binding protein 1
	AT2G0285C	131.48318	-2.243696	-2.429476	0.3650655	-6.14601	7.95E-10	1.00E-07	ARPN	plantacyanin
	AT1G4800C	36.300131	-2.246589	-2.587936	0.4623958	-4.858584	1.18E-06	4.20E-05	AtMYB112	myb domain protein 112
	AT5G0495C	71.973352	-2.250432	-2.674972	0.5007816	-4.493838	7.00E-06	0.0001708	ATNAS1	ARABIDOPSIS THALIANA NICOTIANAMINE SYNTHASE 1
	AT5G5932C	281.24653	-2.252509	-3.290124	0.6346608	-3.549154	0.0003865	0.0038671	LTP3	lipid transfer protein 3
	AT3G2766C	6.6954818	-2.253704	-4.343226	0.6778668	-3.3247	0.0008851	0.0072538	OLE3	OLEOSIN 3
	AT1G6254C	19.478448	-2.262727	-3.000018	0.5840049	-3.874501	0.0001068	0.0014642	FMO GS-OX	flavin-monooxygenase glucosinolate S-oxygenase 2
	AT2G2718C	25.016547	-2.264324	-2.976128	0.5795837	-3.906812	9.35E-05	0.0013198	NA	NA
	AT5G1808C	591.20264	-2.268012	-2.419711	0.3335469	-6.79968	1.05E-11	2.15E-09	SAUR24	small auxin up RNA 24
	AT1G05065	120.75447	-2.275009	-2.53193	0.4142606	-5.491734	3.98E-08	2.75E-06	CLE20	CLAVATA3/ESR-RELATED 20
	AT3G0886C	21.923774	-2.277285	-3.056673	0.5912339	-3.85175	0.0001173	0.0015772	PYD4	PYRIMIDINE 4
	AT1G26945	844.92719	-2.279661	-2.459137	0.3579372	-6.368886	1.90E-10	2.86E-08	KDR	KIDARI
	AT2G07774	291.61346	-2.2822	-2.52658	0.4062358	-5.617919	1.93E-08	1.46E-06	NA	NA
	ATMG0122	36.366313	-2.283164	-2.681554	0.4854804	-4.702897	2.56E-06	7.57E-05	ORF113	0
	AT3G20395	8.7016456	-2.283522	-3.767531	0.6595758	-3.462106	0.000536	0.0049664	NA	NA
	AT5G10946	110.43574	-2.287875	-2.655332	0.473444	-4.832408	1.35E-06	4.66E-05	NA	NA
	AT1G1871C	60.040707	-2.289335	-2.731865	0.5036627	-4.545372	5.48E-06	0.00014	AtMYB47	myb domain protein 47
	AT5G1516C	250.6278	-2.291868	-2.489027	0.3711952	-6.174294	6.65E-10	8.62E-08	BHLH134	BASIC HELIX-LOOP-HELIX PROTEIN 134
	AT1G5075C	28.338007	-2.298327	-2.834835	0.5337465	-4.306028	1.66E-05	0.0003408	NA	NA
YES	AT3G1672C	3767.7344	-2.298923	-2.54692	0.4079361	-5.635497	1.75E-08	1.37E-06	ATL2	TOXICOS EN LEVADURA 2
	AT5G1803C	919.24436	-2.309912	-2.481745	0.3493539	-6.611955	3.79E-11	6.70E-09	NA	NA
	AT3G2937C	356.62568	-2.31107	-2.624155	0.4454577	-5.188079	2.12E-07	1.10E-05	P1R3	P1R3
	AT2G21185	1100.3967	-2.319539	-2.457825	0.3171874	-7.312836	2.62E-13	7.25E-11	NA	NA
	AT1G01453	6.9068873	-2.320137	-4.395325	0.6753895	-3.435257	0.000592	0.005369	NA	NA
	AT2G0299C	292.20824	-2.321721	-2.566678	0.4038434	-5.749063	8.97E-09	7.81E-07	ATRNS1	RIBONUCLEASE 1
	AT1G52342	2002.3619	-2.326601	-2.560535	0.3964491	-5.8686	4.39E-09	4.24E-07	NA	NA
	AT3G2076C	942.09778	-2.327039	-2.558248	0.3944385	-5.899624	3.64E-09	3.63E-07	NA	NA
	AT5G4563C	80.660411	-2.328315	-2.804175	0.5135053	-4.534159	5.78E-06	0.000146	NA	NA
	AT1G1257C	29.711116	-2.335134	-2.991028	0.5622079	-4.153506	3.27E-05	0.0005768	NA	NA
	AT4G27652	741.30034	-2.335784	-2.598799	0.4147767	-5.631426	1.79E-08	1.39E-06	NA	NA
YES	AT1G2402C	1118.5595	-2.337872	-2.586479	0.4054817	-5.765666	8.13E-09	7.22E-07	MLP423	MLP-like protein 423
	AT2G2869C	8.654603	-2.343564	-4.207621	0.6720724	-3.487071	0.0004883	0.0046318	NA	NA
	AT2G34655	1540.3959	-2.344934	-2.526486	0.3554438	-6.597201	4.19E-11	7.26E-09	NA	NA
	AT5G5407C	802.80328	-2.347772	-2.565644	0.3834985	-6.121985	9.24E-10	1.13E-07	AT-HSFA9	ARABIDOPSIS THALIANA HEAT SHOCK TRANSCRIPTION FACTOR A9
	AT2G25625	61.723744	-2.351742	-2.647816	0.4312882	-5.452832	4.96E-08	3.27E-06	NA	NA
	AT5G1021C	8.2271333	-2.35176	-4.107695	0.6674716	-3.523386	0.0004261	0.004154	NA	NA
	AT5G01075	127.2046	-2.354601	-2.544443	0.3608632	-6.524914	6.80E-11	1.15E-08	NA	NA
	AT1G5288C	723.81862	-2.356139	-2.537986	0.3548766	-6.639319	3.15E-11	5.74E-09	ANAC018	Arabidopsis NAC domain containing protein 18

Supplemental Table 3-RNA seq data comparing *Pro35S::MYB63 Pro35S::LAC17* with wild type.

DAP.MYB63.target(1.5kb upstream TSS)	Gene	baseMean	log2FoldChange	lfcMLE	lfcSE	stat	pvalue	padj (FDR)	TAIR10 Symbol	TAIR10 Annotation (Short)
	AT2G39030	119.73088	-2.357016	-2.619285	0.4119095	-5.722169	1.05E-08	8.97E-07	NATA1	N-acetyltransferase activity 1
	AT3G61190	333.01016	-2.357134	-2.537126	0.3529801	-6.67781	2.43E-11	4.55E-09	BAP1	BON association protein 1
	AT4G33040	153.80966	-2.359008	-2.538019	0.3518104	-6.705338	2.01E-11	3.83E-09	NA	NA
	AT3G14395	13.760496	-2.359023	-3.506033	0.6319393	-3.732989	0.0001892	0.0022794	NA	NA
	AT1G68240	37.918029	-2.360358	-2.75044	0.4759685	-4.959062	7.08E-07	2.81E-05	NA	NA
	AT5G24155	27.088411	-2.360894	-2.945548	0.5418993	-4.356702	1.32E-05	0.0002855	NA	NA
	AT2G40610	1942.4077	-2.361322	-2.576353	0.3806061	-6.204111	5.50E-10	7.18E-08	ATEXP8	0
	AT3G19380	1974.8667	-2.361357	-2.555934	0.3650078	-6.469333	9.84E-11	1.61E-08	PUB25	plant U-box 25
	AT3G49820	6.8317842	-2.364978	-4.40321	0.6730874	-3.513627	0.000442	0.0042758	NA	NA
	AT1G76610	68.981392	-2.365707	-2.639073	0.4172733	-5.669442	1.43E-08	1.16E-06	NA	NA
	AT2G25810	12.023887	-2.366691	-3.617814	0.6414868	-3.689383	0.0002248	0.0025869	TIP4;1	tonoplast intrinsic protein 4;1
	AT1G35560	1370.3992	-2.367544	-2.570601	0.3711752	-6.378507	1.79E-10	2.73E-08	AtTCP23	0
	AT1G76600	3064.0526	-2.36934	-2.490163	0.295858	-8.00837	1.16E-15	5.82E-13	NA	NA
	AT3G25905	30.96127	-2.375529	-3.062829	0.5667694	-4.191349	2.77E-05	0.0005099	CLE27	CLAVATA3/ESR-RELATED 27
	AT5G37770	6259.8322	-2.388344	-2.555892	0.3407443	-7.009195	2.40E-12	5.27E-10	CML24	CALMODULIN-LIKE 24
	AT5G17220	220.81146	-2.389823	-2.657003	0.4132086	-5.783574	7.31E-09	6.72E-07	ATGSTF12	ARABIDOPSIS THALIANA GLUTATHIONE S-TRANSFERASE PHI 12
	AT2G05540	988.13139	-2.392072	-2.649576	0.4074191	-5.871281	4.32E-09	4.21E-07	NA	NA
	AT1G27730	2374.2236	-2.393161	-2.51785	0.2987259	-8.011227	1.14E-15	5.82E-13	STZ	salt tolerance zinc finger
	AT2G25735	3804.4589	-2.395393	-2.584939	0.3588184	-6.67578	2.46E-11	4.57E-09	NA	NA
	AT4G25490	242.63785	-2.396577	-2.670488	0.4166835	-5.751552	8.84E-09	7.74E-07	ATCBF1	0
	AT5G18010	230.79468	-2.402197	-2.654588	0.4028637	-5.962804	2.48E-09	2.60E-07	SAUR19	small auxin up RNA 19
	AT1G02820	1340.0671	-2.403478	-2.567872	0.3369148	-7.133786	9.76E-13	2.35E-10	AtLEA3	0
	AT4G21970	29.825305	-2.404388	-2.852719	0.4958553	-4.848971	1.24E-06	4.33E-05	NA	NA
	AT4G39675	48.700838	-2.404418	-2.767738	0.4609488	-5.216237	1.83E-07	9.57E-06	NA	NA
	AT1G04330	24.509032	-2.408517	-3.082589	0.5611798	-4.291882	1.77E-05	0.0003578	NA	NA
	AT5G56550	4405.6826	-2.414151	-2.559645	0.3187919	-7.572812	3.65E-14	1.34E-11	ATOXS3	OXIDATIVE STRESS 3
	AT1G70985	177.05006	-2.414714	-2.660027	0.3973045	-6.077742	1.22E-09	1.42E-07	NA	NA
	AT1G28370	358.15523	-2.422439	-2.666391	0.3963052	-6.11256	9.80E-10	1.18E-07	ATERF11	ERF DOMAIN PROTEIN 11
	AT4G27657	185.10739	-2.4323	-2.630702	0.3627732	-6.70474	2.02E-11	3.83E-09	NA	NA
	AT5G52050	95.529723	-2.44926	-2.797864	0.4526592	-5.410826	6.27E-08	3.95E-06	NA	NA
	AT1G68238	802.89938	-2.44929	-2.634894	0.3521042	-6.956149	3.50E-12	7.59E-10	NA	NA
	AT3G17790	678.47972	-2.455042	-2.699227	0.3944587	-6.223825	4.85E-10	6.52E-08	ATACP5	0
	AT1G29510	889.95216	-2.455405	-2.598776	0.3142247	-7.814171	5.53E-15	2.49E-12	SAUR67	SMALL AUXIN UPREGULATED RNA 67
	AT3G21500	8.6229744	-2.461485	-4.781542	0.6775809	-3.632754	0.0002804	0.0030489	DXL1	DXS-like 1
	AT5G18060	488.7529	-2.463886	-2.722455	0.4030867	-6.112545	9.81E-10	1.18E-07	SAUR23	SMALL AUXIN UP RNA 23
	AT3G03190	406.3494	-2.469663	-2.647208	0.3440884	-7.177409	7.10E-13	1.78E-10	ATGSTF11	glutathione S-transferase F11
	AT5G10990	6.8112431	-2.485794	-5.455966	0.6827769	-3.640712	0.0002719	0.0029821	SAUR69	SMALL AUXIN UPREGULATED RNA 69
	AT3G61930	11.428267	-2.492273	-4.526916	0.6713079	-3.712564	0.0002052	0.0024087	NA	NA
	AT1G01250	291.0468	-2.50177	-2.678935	0.3415863	-7.323975	2.41E-13	6.89E-11	NA	NA
	AT5G38120	161.32068	-2.511498	-2.68145	0.3348203	-7.501032	6.33E-14	2.00E-11	4CL8	0
	AT1G29290	227.89639	-2.522742	-2.831758	0.42797	-5.89467	3.75E-09	3.72E-07	NA	NA
	AT1G73500	3083.4593	-2.522882	-2.665432	0.3097595	-8.14465	3.80E-16	2.14E-13	ATMKK9	0
	AT5G10625	27.808794	-2.5255	-3.406564	0.5922749	-4.264067	2.01E-05	0.0003941	NA	NA
YES	AT4G33790	36.069329	-2.526397	-3.157676	0.5429993	-4.652672	3.28E-06	9.21E-05	CER4	ECERIFERUM 4
	AT1G35210	664.09625	-2.526935	-2.747309	0.3736076	-6.763607	1.35E-11	2.67E-09	NA	NA
	AT4G27360	9.9250908	-2.529271	-3.947029	0.6426032	-3.935977	8.29E-05	0.0012037	NA	NA

Supplemental Table 3-RNA seq data comparing *Pro35S::MYB63 Pro35S::LAC17* with wild type.

DAP.MYB63.target(1.5kb upstream TSS)	Gene	baseMean	log2FoldChange	lfcMLE	lfcSE	stat	pvalue	padj (FDR)	TAIR10 Symbol	TAIR10 Annotation (Short)
	AT1G19960	6628.5427	-2.53829	-2.760989	0.3747698	-6.772931	1.26E-11	2.56E-09	NA	NA
	AT2G24692	30.429763	-2.541245	-3.033202	0.5014663	-5.067629	4.03E-07	1.81E-05	NA	NA
	AT1G02340	162.85814	-2.551067	-2.891754	0.4421285	-5.769967	7.93E-09	7.07E-07	FBI1	0
	AT4G10290	18.162502	-2.557123	-3.457106	0.5919882	-4.31955	1.56E-05	0.0003254	NA	NA
	AT1G49780	1613.8387	-2.577203	-2.86844	0.4153761	-6.204506	5.49E-10	7.18E-08	PUB26	plant U-box 26
	AT5G53905	376.24074	-2.580024	-2.854217	0.4049078	-6.371881	1.87E-10	2.83E-08	NA	NA
	AT3G24450	42.741568	-2.58048	-3.102204	0.5103081	-5.056711	4.27E-07	1.88E-05	NA	NA
	AT3G21890	61.531967	-2.584896	-3.021291	0.4794941	-5.390882	7.01E-08	4.31E-06	BBX31	B-box domain protein 31
	AT4G17490	1106.7133	-2.597545	-2.785369	0.3450816	-7.527335	5.18E-14	1.70E-11	ATERF6	ethylene responsive element binding factor 6
	AT4G37140	13.298851	-2.612003	-4.046066	0.6417333	-4.070232	4.70E-05	0.0007722	ATMES20	ARABIDOPSIS THALIANA METHYL ESTERASE 20
	AT2G45570	18.871047	-2.615675	-3.491556	0.5836514	-4.48157	7.41E-06	0.0001788	CYP76C2	cytochrome P450, family 76, subfamily C, polypeptide 2
	AT5G53200	49.668533	-2.62087	-2.976454	0.4427694	-5.919267	3.23E-09	3.24E-07	TRY	TRIPTYCHON
	AT5G24780	104.42583	-2.627735	-2.952009	0.4285907	-6.131105	8.73E-10	1.09E-07	ATVSP1	0
	AT1G10586	10.972264	-2.636825	-4.111091	0.6415268	-4.110234	3.95E-05	0.0006726	NA	NA
	AT5G49525	124.06907	-2.641146	-2.848072	0.3559983	-7.418985	1.18E-13	3.54E-11	NA	NA
	AT1G06080	288.23457	-2.642952	-2.988618	0.4390218	-6.020093	1.74E-09	1.90E-07	ADS1	delta 9 desaturase 1
	AT3G56220	18.805196	-2.652456	-3.492966	0.575171	-4.611596	4.00E-06	0.0001088	NA	NA
	AT3G27650	72.255474	-2.656707	-3.142826	0.4938808	-5.379247	7.48E-08	4.49E-06	LBD25	LOB domain-containing protein 25
	AT5G09980	38.19083	-2.665952	-3.230325	0.5159308	-5.167266	2.38E-07	1.20E-05	PROPEP4	elicitor peptide 4 precursor
	AT3G20470	725.71762	-2.682905	-2.906501	0.3664663	-7.321015	2.46E-13	6.93E-11	ATGRP-5	0
	AT4G30450	604.41043	-2.68855	-2.922596	0.3732057	-7.203938	5.85E-13	1.53E-10	NA	NA
	AT3G44260	3163.7459	-2.716845	-3.69124	0.6017531	-4.514884	6.34E-06	0.0001575	AtCAF1a	CCR4- associated factor 1a
	AT3G29000	606.07044	-2.719186	-3.071595	0.4377788	-6.211324	5.25E-10	7.01E-08	NA	NA
	AT1G33102	119.36327	-2.719342	-2.987551	0.3914232	-6.947319	3.72E-12	7.99E-10	NA	NA
	AT2G01280	19.431685	-2.723528	-3.538518	0.5656966	-4.814469	1.48E-06	4.96E-05	MEE65	maternal effect embryo arrest 65
	AT4G12080	121.80287	-2.726669	-2.954588	0.3657606	-7.454793	9.00E-14	2.75E-11	AHL1	AT-hook motif nuclear-localized protein 1
	AT1G05835	280.40942	-2.736642	-3.143345	0.4604663	-5.943197	2.80E-09	2.88E-07	NA	NA
	AT5G55410	28.444933	-2.7525	-3.732182	0.5938958	-4.634651	3.58E-06	9.90E-05	NA	NA
	AT3G20362	33.423016	-2.757778	-3.371375	0.522192	-5.281158	1.28E-07	7.21E-06	NA	NA
	AT1G66370	7.7654108	-2.75824	-5.620063	0.6731893	-4.097272	4.18E-05	0.0007047	AtMYB113	myb domain protein 113
	AT5G22250	1091.7217	-2.76689	-3.014245	0.3774665	-7.33016	2.30E-13	6.68E-11	AtCAF1b	CCR4- associated factor 1b
	AT5G54585	1232.2622	-2.771109	-2.94827	0.327022	-8.473769	2.38E-17	1.65E-14	NA	NA
	AT3G09870	58.018714	-2.77177	-3.086411	0.4127506	-6.715363	1.88E-11	3.64E-09	SAUR48	SMALL AUXIN UPREGULATED RNA 48
	AT3G30460	63.687573	-2.79548	-3.144105	0.4281407	-6.529348	6.61E-11	1.12E-08	NA	NA
	AT4G14695	9.347565	-2.79881	-4.886505	0.6581578	-4.252491	2.11E-05	0.0004114	NA	NA
	AT5G54490	703.84336	-2.821263	-3.049701	0.3619773	-7.794033	6.49E-15	2.78E-12	PBP1	pinoid-binding protein 1
	AT1G17020	14.470078	-2.836136	-4.15154	0.6194302	-4.57862	4.68E-06	0.0001237	ATSRG1	SENESCENCE-RELATED GENE 1
	AT5G21960	21.154749	-2.836882	-3.68435	0.5650571	-5.020523	5.15E-07	2.20E-05	NA	NA
	AT5G55620	2593.9987	-2.837373	-2.992064	0.3047909	-9.309245	1.29E-20	1.29E-17	NA	NA
	AT1G69880	35.999018	-2.853358	-3.724034	0.5710332	-4.996833	5.83E-07	2.42E-05	ATH8	thioredoxin H-type 8
YES	AT5G02580	52.767417	-2.868782	-3.607897	0.546829	-5.246213	1.55E-07	8.40E-06	NA	NA
	AT4G27280	2533.8332	-2.889552	-3.01737	0.277289	-10.42072	1.99E-25	3.59E-22	NA	NA
	AT3G10930	1237.259	-2.893983	-3.207659	0.4082879	-7.088094	1.36E-12	3.18E-10	NA	NA
	AT2G17300	128.45398	-2.905028	-3.178526	0.3839413	-7.566332	3.84E-14	1.38E-11	NA	NA

Supplemental Table 3-RNA seq data comparing *Pro35S::MYB63 Pro35S::LAC17* with wild type.

DAP.MYB63.target(1.5kb upstream TSS)	Gene	baseMean	log2FoldChange	lfcMLE	lfcSE	stat	pvalue	padj (FDR)	TAIR10 Symbol	TAIR10 Annotation (Short)
	AT3G23170	1144.9489	-2.907607	-3.05599	0.295793	-9.829872	8.37E-23	1.08E-19	NA	NA
	AT1G34060	93.093567	-2.910935	-3.367148	0.4675505	-6.225926	4.79E-10	6.49E-08	NA	NA
	AT4G30460	274.29894	-2.915046	-3.184276	0.3817135	-7.636736	2.23E-14	8.73E-12	NA	NA
	AT2G38210	213.41033	-2.931721	-3.188414	0.3732165	-7.855282	3.99E-15	1.89E-12	PDX1L4	putative PDX1-like protein 4
	AT5G51190	103.61222	-2.941586	-3.204914	0.3759044	-7.825356	5.06E-15	2.34E-12	NA	NA
	AT5G53980	7.4806703	-2.960192	-8.793799	0.6814708	-4.343829	1.40E-05	0.0002974	ATHB52	homeobox protein 52
	AT4G15320	9.6697442	-2.972139	-5.944449	0.6686673	-4.444869	8.79E-06	0.0002056	ATCSLB06	cellulose synthase-like B6
	AT2G44578	66.528129	-2.977975	-3.436607	0.4624035	-6.44021	1.19E-10	1.94E-08	NA	NA
	AT1G33760	277.9001	-2.987737	-3.314456	0.4088467	-7.307719	2.72E-13	7.42E-11	NA	NA
	AT1G72920	729.32911	-2.993114	-3.225977	0.3557749	-8.412945	4.00E-17	2.40E-14	NA	NA
	AT5G66800	79.482067	-3.004	-3.415085	0.4440478	-6.765037	1.33E-11	2.67E-09	NA	NA
	AT1G12610	44.458353	-3.010286	-3.580251	0.4947388	-6.084596	1.17E-09	1.38E-07	DDF1	DWARF AND DELAYED FLOWERING 1
	AT3G26960	2676.4418	-3.038121	-3.217262	0.3155883	-9.626849	6.16E-22	7.40E-19	NA	NA
	AT5G59510	17.599048	-3.065378	-4.434473	0.6120771	-5.008156	5.50E-07	2.30E-05	DVL18	DEVIL 18
	AT5G36910	478.45039	-3.070396	-3.324985	0.3652023	-8.407383	4.19E-17	2.44E-14	THI2.2	thionin 2.2
	AT2G47270	107.25646	-3.105582	-3.45319	0.4107014	-7.561656	3.98E-14	1.38E-11	UPB1	UPBEAT1
	AT1G32920	9941.5756	-3.142127	-3.407537	0.3686939	-8.522318	1.56E-17	1.14E-14	NA	NA
	AT3G19350	70.079601	-3.196189	-3.816331	0.500378	-6.38755	1.69E-10	2.60E-08	MPC	maternally expressed pab C-terminal
	AT5G17350	255.61813	-3.227208	-3.56137	0.3996103	-8.075886	6.70E-16	3.66E-13	NA	NA
	AT3G53250	22.307036	-3.280679	-4.542955	0.5931353	-5.531081	3.18E-08	2.28E-06	SAUR57	SMALL AUXIN UPREGULATED RNA 57
	AT4G13195	186.91678	-3.280934	-3.530528	0.3509325	-9.34919	8.83E-21	9.36E-18	CLE44	CLAVATA3/ESR-RELATED 44
	AT1G59950	10.777003	-3.29427	-9.323984	0.6751912	-4.879019	1.07E-06	3.89E-05	NA	NA
	AT2G37430	251.85819	-3.323874	-3.7342	0.4282902	-7.760797	8.44E-15	3.46E-12	ZAT11	zinc finger of Arabidopsis thaliana 11
	AT4G24570	2542.663	-3.337363	-3.565679	0.3367747	-9.909778	3.77E-23	5.23E-20	DIC2	dicarboxylate carrier 2
	AT5G50335	869.19138	-3.344565	-3.64362	0.3770697	-8.869885	7.32E-19	6.60E-16	NA	NA
	AT2G34925	82.634343	-3.394058	-3.879477	0.4488138	-7.562285	3.96E-14	1.38E-11	CLE42	CLAVATA3/ESR-RELATED 42
	AT5G47230	699.83052	-3.398252	-3.756885	0.4032714	-8.426711	3.56E-17	2.21E-14	ATERF-5	ETHYLENE RESPONSIVE ELEMENT BINDING FACTOR- 5
	AT3G28770	91.691534	-3.420487	-3.922457	0.4538076	-7.537306	4.80E-14	1.63E-11	NA	NA
	AT1G05575	1668.6077	-3.439458	-3.71409	0.3597364	-9.561049	1.17E-21	1.31E-18	NA	NA
	AT5G13170	257.02	-3.452961	-8.155753	0.6824918	-5.059344	4.21E-07	1.88E-05	AtSWEET15	0
	AT4G13395	1406.4106	-3.474276	-3.913253	0.4330479	-8.022845	1.03E-15	5.48E-13	DVL10	DEVIL 10
	AT1G56650	49.816637	-3.481282	-4.219535	0.5116775	-6.803665	1.02E-11	2.11E-09	ATMYB75	MYB DOMAIN PROTEIN 75
	AT2G37030	45.424485	-3.616151	-4.259402	0.4801969	-7.530559	5.05E-14	1.69E-11	SAUR46	SMALL AUXIN UPREGULATED RNA 46
	AT3G11480	41.433255	-3.881345	-5.493274	0.5994383	-6.474969	9.48E-11	1.58E-08	ATBSMT1	0
	AT1G74930	1691.1291	-4.109283	-4.392917	0.3376036	-12.17192	4.39E-34	3.95E-30	ORA47	0
	AT2G44840	326.46668	-4.340355	-4.865814	0.4220449	-10.28411	8.31E-25	1.25E-21	ATERF13	ETHYLENE-RESPONSIVE ELEMENT BINDING FACTOR 13
	AT5G04340	4383.2978	-4.368571	-4.889829	0.4230205	-10.32709	5.31E-25	8.71E-22	C2H2	0
	AT4G37290	116.92904	-4.506379	-5.413155	0.4991502	-9.028102	1.75E-19	1.66E-16	NA	NA
	AT3G49580	271.94608	-4.603195	-5.125511	0.4105529	-11.21219	3.55E-29	8.00E-26	LSU1	RESPONSE TO LOW SULFUR 1
	AT2G18210	328.29451	-4.998477	-5.626183	0.4256029	-11.74446	7.54E-32	3.40E-28	NA	NA

Supplementary table 4: List of primers used in this study

(A) Primers used for generating *Pro35S:MYB58* and *Pro35S:MYB63* lines

MYB58_ORF_F	AAAAAGCAGGCTtgtcaggatcacacacacca
MYB58_ORF_F	AGAAAGCTGGGTtcgacgtttcttcgttgttg
MYB63_ORF_F	AAAAAGCAGGCTgagaatggggaagggaagag
MYB63_ORF_R	AGAAAGCTGGGTacctttccatcatacatattgtca

(B) Primers used for quantitative real time PCR

APT1 RT F	TGCTGCAATCCGACTACTTG
APT1 RT R	AATCCCACGCAAAATGTCTC
MYB58 RT F	GCGAGTTTACGGGGATGTTA
MYB58 RT R	GTTCGCTTTCCAGGTGCTTA
MYB63 RT F	GGGTTCCAACCAACAAGATG
MYB63 RT R	GCTCTTGAGCCTGAGCTGTT
