# Supplementary material for: Dwarfism of high‐monolignol Arabidopsis plants is rescued by ectopic LACCASE overexpression
Source: Plant Direct. 2020 Sep 28;4(9):e00265. doi: 10.1002/pld3.265 (PMC7520647; doi:10.1002/pld3.265)
Supplement: Supplementary file 3 — Supplementary Material [file PLD3-4-e00265-s003.pdf]

Reviewer Comments:
Reviewer #1:
Comments to the Author (Required):
Authors have made the corrections. Thank you.

Reviewer #2:
Comments to the Author (Required):
All previous suggestions for minor changes are satisfied in this version.

Reviewer #3:
Comments to the Author (Required):
Congratulations, the manuscript has improved markedly.
A minor comment: line 699 "concnetration" should be "concentration", and "10uM" should be "10 uM"

Transferred Reviewer Comments:
